# Remapping the Synthetic Landscape of α‐Tertiary Alkylamines via Dual‐Functional Catechol

**DOI:** 10.1002/anie.9109858

**Published:** 2026-07-21

**Authors:** Chen‐Yan Cai, Yuzuru Kanda, Tian Qin

**Affiliations:** ^1^ Department of Biochemistry The University of Texas Southwestern Medical Center Dallas Texas USA; ^2^ Novartis Biomedical Research Cambridge Massachusetts USA

**Keywords:** catechol activation, ketimines, organoboron reagents, three‐component assembly, α‐tertiary amines

## Abstract

α‐Tertiary alkylamines are privileged motifs with a strong propensity to be bioactive, rendering their efficient synthesis fundamentally important across diverse areas of chemistry. Despite recent advances in aliphatic amine preparation, the direct construction of hindered *α*‐tertiary amines remains a long‐standing challenge. Here, we report a three‐component assembly that exploits the dual role of catechol to enable the modular synthesis of these motifs from readily available, bench‐stable reactants. This method exhibits exceptional functional‐group tolerance and provides access to a variety of synthetically demanding molecular architectures, including consecutive quaternary centers as well as vicinal aminoether and diamine fragments. This approach, therefore, represents a valuable supplement to the chemist's toolbox for *α*‐tertiary alkylamines, offering streamlined routes to complex targets and significant possibilities in drug discovery.

## Graphical abstract

A three‐component assembly strategy has been developed for the modular synthesis of hindered *α*‐tertiary aliphatic amines from readily available precursors through unlocking the dual role of catechol. The practicality of this methodology has been demonstrated in over 90 examples, including compounds that are otherwise difficult to access, along with five synthetic applications that streamline the preparation of intermediates for medicinally relevant targets.

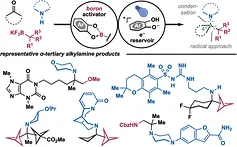

## 1 Introduction

Aliphatic amines are ubiquitous structural motifs found across a broad range of areas, including pharmaceuticals, agrochemicals, and specialty chemicals [1, 2, 3, 4]. The substituents directly attached to nitrogen provide opportunities for modulation and precise tuning of their properties [5, 6]. Notably, tertiary alkyl substitution can enhance three‐dimensional diversity, modulate lipophilicity, and improve oxidative metabolic stability, rendering the construction of nitrogen‐substituted quaternary centers a highly desirable synthetic objective [7, 8, 9, 10, 11]. However, the sterically congested environment continues to impede their synthetic accessibility [12, 13, 14, 15]. Moreover, modern synthetic chemistry faces increasing demands for efficiency and generality. These challenges are especially pronounced in early drug discovery, where the complexity of structure–activity relationships in highly context‐dependent hit‐to‐lead development requires the rapid assembly of combinatorial libraries from simple, readily available building blocks to accelerate the identification of candidates with favorable biological and physicochemical profiles [16, 17, 18, 19].

**FIGURE 1:**
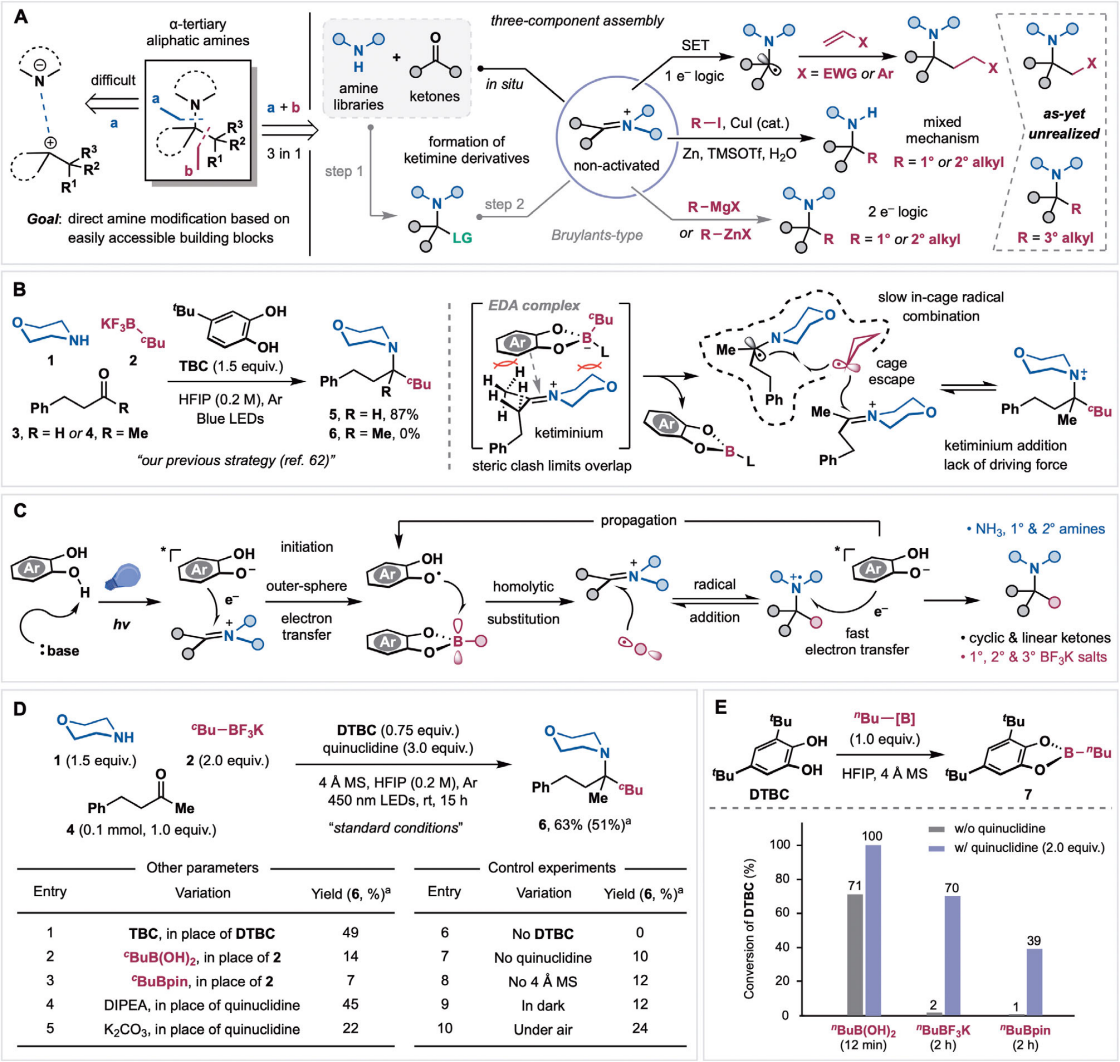
Three‐component assembly of *α*‐tertiary amines. (A) Retrosynthesis and synthetic development. (B) Preliminary results and anticipated transformation challenges. (C) Mechanistic design. (D) Reaction development. ^a^Yield based on ^1^H NMR analysis of the reaction crude using mesitylene as the internal standard. Numbers in parentheses refer to yields of isolated **6**. (E) Effect of organoboron reagents and quinuclidine on transesterification conversion. LG, leaving group; EWG, electron‐withdrawing group; TMS, trimethylsilyl; *^t^* Bu, tertiary butyl; HFIP, hexafluoro‐2‐propanol; LED, light‐emitting diode; MS, molecular sieves; DIPEA, *N*,*N*‐diisopropylethylamine; *^n^* Bu, normal butyl; Bpin, pinacol boronate.

The direct alkylation of nitrogen nucleophiles from established amine libraries via classical S_N_2 reaction [20] to construct C(*sp^3^*)─N bonds is widely applied in the pharmaceutical industry, but sterically encumbered tertiary halides generally fall outside its practical scope (Figure 1A, left). Alternative strategies have been developed following this bond‑disconnection logic, exploiting planar carbocations (S_N_1) [21, 22, 23, 24], electrophilic nitrogen sources [25, 26, 27], and nitrene species via direct C─H insertion [28, 29, 30] or radical‑mediated pathways [31, 32, 33, 34, 35, 36]. Nonetheless, these approaches typically require at least one prefunctionalized precursor, thereby introducing additional steps.

Three‐component assembly strategies [37] that use in situ‐generated ketimine derivatives as the key intermediate offer greater efficiency and modularity [38] (Figure 1A, right). However, unlike aldehyde‐derived imines [39, 40], replacing the hydrogen with an alkyl group increases steric hindrance while reducing electrophilicity, thereby disfavoring imine formation and subsequent transformations [41, 42]. Substantial progress has thus relied on engineered precursors [43, 44, 45, 46, 47, 48, 49, 50, 51, 52]. Direct utilization of readily available and ubiquitous precursors represents a highly desirable yet challenging goal. As a notable advance, the Bruylants reaction [53], although widely cited in the patent literature, relies on basic organometallic reagents that require a two‐step protocol, exhibit restricted functional group tolerance, and have a limited substrate scope. More recently, Gaunt and coworkers elegantly identified a zinc‐mediated mode of alkyl iodide activation that enables the one‐pot synthesis of secondary amines bearing an *α*‐quaternary center [54]. Another strategy involves single‐electron reduction of the in situ‐formed ketimine to generate nucleophilic *α*‐amino carbon radicals, which add to activated, typically terminal, olefins to yield the targeted amines [55, 56, 57, 58]. Despite ongoing progress toward *α*‐tertiary alkylamines, existing methods remain inadequate to meet the ideal criteria for practical applications.

Therefore, development of robust methods for the direct utilization of vast existing libraries of amine sources to liberate a wide array of *α*‐tertiary aliphatic amines in a modular fashion, with a product scope that includes contiguous quaternary centers, vicinal amino ethers, and diamine motifs, is highly desirable. Here, we harness the versatile reactivity of bench‐stable potassium alkyltrifluoroborate salts [59, 60, 61] to provide a general and practical solution to this challenge, with readily accessible ketones serving as the third modular component. Significantly, 3,5‐di‐*tert*‐butylcatechol (**DTBC**) was applied as a dual‐functional activator in combination with a quinuclidine base additive.

## 2 Results and Discussion

Motivated by our recent advances in modular alkylamine synthesis [62], we set out to extend this strategy to more challenging *α*‐tertiary amines. However, the application of this chemistry to the formation of these sterically hindered compounds poses several substantial challenges (Figure 1B, right): the sluggish electron donor–acceptor (EDA) interactions; the slow in‐cage radical combination leading to radical escape; the inefficient trapping of escaped radicals by nonactivated ketiminium species in the absence of an effective driving force. Unsurprisingly, simply replacing aldehydes with ketones caused the reaction to derail (Figure 1B, left). In this scenario, a mechanism design that is expected to be less sensitive to steric congestion has been outlined in Figure 1C. We hypothesized that the reducing ability of catechol could be boosted after deprotonation and subsequent photoexcitation [63, 64, 65], which ought to be competent to drive the reversible radical addition toward productive bond formation. The resulting catechol radical then propagates the chain through attacking the boron atom of *B*‐alkylcatecholboranes to deliver another portion of the alkyl radical, a process pioneered by the Renaud group [66, 67]. The proposed mechanism involves catechol activating otherwise less radical‐viable organoboron species [68] via reversible transesterification—a necessary step that, in turn, reduces the effective concentration of free catechol—with the observed exchange rate depending on the type of boron reagent and the base used as a promoter (Figure 1E).

Common reagents, morpholine (**1**), potassium cyclobutyltrifluoroborate (**2**), and 4‑phenyl‑2‑butanone (**4**) were chosen and employed as model reaction partners. As an initial attempt to validate our mechanistic hypothesis, we introduced a base additive under the previously established conditions described in Figure 1B and observed encouraging formation of product **6** in 11% assay yield (see Table S1). By adding molecular sieves to elevate the concentration of alkyl–ketiminium ion, replacing 4‐*tert*‐butylcatechol (**TBC**) with **DTBC**, using quinuclidine as the base additive, and modulating the reaction's stoichiometry, the desired amine product **6** was obtained in an optimal assay yield of 63% under 450 nm LED irradiation (Figure 1D). Hexafluoroisopropanol (HFIP) has been identified as an optimal solvent, likely due to its ability to accelerate ketiminium formation [69, 70].

The interplay between the steric and electronic properties of catechol, as well as its solubility in the HFIP solvent, accounts for its observed reactivity (Figure 1D, entry 1; Table S3). Exploration of other organoboron reagents (entries 2 and 3) proved ineffective, presumably due to the unfavorable concentration of free **DTBC**, and water generated during the conversion of boronic acids to **DTBC** esters probably also contributed to the reduced yield. Most of the evaluated organic and inorganic bases enhanced the reaction conversion, albeit to varying extents (Figure 1D, entries 4 and 5; Table S4). Omission of **DTBC** completely abolished the reaction (entry 6), and only a small amount of reaction occurs in the absence of quinuclidine, molecular sieves, or light (entries 7–9). Moderate yield erosion under atmospheric conditions necessitates an inert atmosphere to preserve yield and reproducibility (entry 10).

**FIGURE 2:**
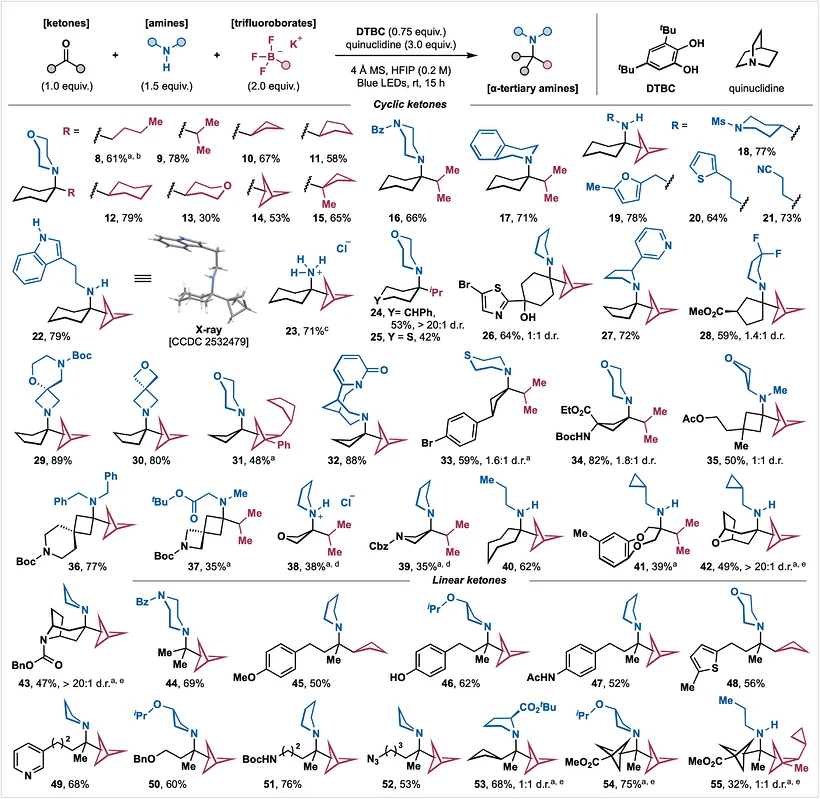
Substrate scope for the three‐component assembly. Conditions: ketone (0.1 mmol), HFIP (0.5 mL). The yield represents the purified product. ^a^RBF_3_K (3.0 equiv.). ^b^ **DTBC** (1.0 equiv.), reaction time: 24 h. ^c^NH_3_ (0.1 mL; 4 M in methanol), no quinuclidine. ^d^3‐Methylbenzene‐1,2‐diol in place of **DTBC**, 420 nm LEDs. ^e^ **TBC** (1.0 equiv.) in place of **DTBC**, reaction time: 24 h. Bz, benzoyl; Ms, mesyl; Boc, *tert*‐butoxy carbonyl; Ac, acetyl; Cbz, benzyloxycarbonyl; *^i^* Pr, isopropyl; Bn, benzyl.

**FIGURE 3:**
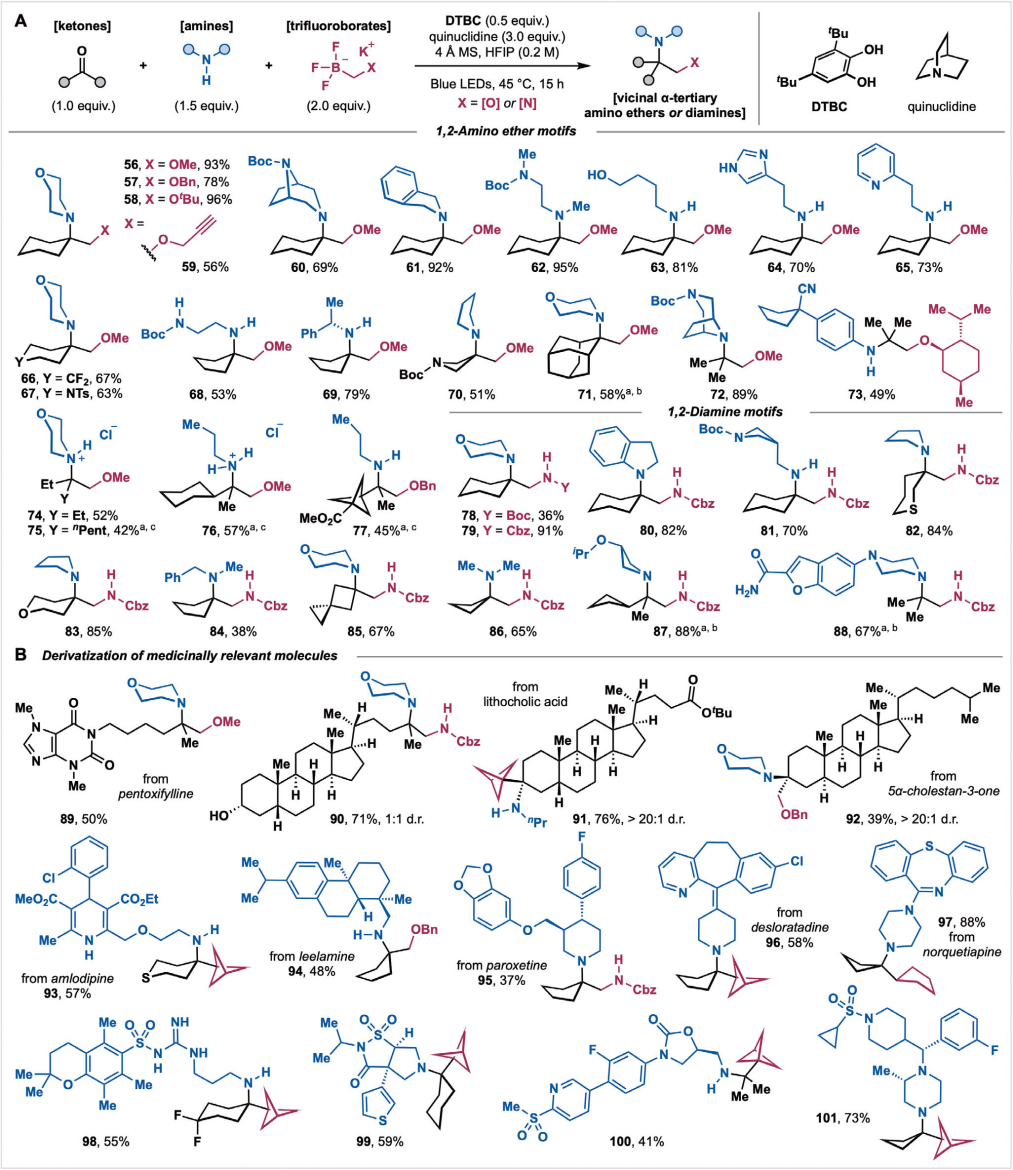
Substrate scope for the three‐component assembly, continued. (A) Scope of vicinal aminoethers and diamines. Conditions: ketone (0.1 mmol), HFIP (0.5 mL). The yield represents the purified product. ^a^RBF_3_K (3.0 equiv.). ^b^ **DTBC** (0.75 equiv.). ^c^ **TBC** (1.0 equiv.) in place of **DTBC**, reaction time: 24 h. Ts, tosyl. (B) Late‐stage derivatization of complex molecules. See the Supporting Information for details.

With the optimized conditions established, the scope of this method was next evaluated (Figures 2 and 3). Cyclic ketones were initially investigated, and substrates containing six‐membered (**8**–**26**), five‐membered (**27**–**30**), and four‐membered (**31**–**39**) rings efficiently delivered the desired α‐tertiary alkylamines. Notably, relatively acidic ketones such as 3‐oxetanone (**38**) and 3‐azetidinone (**39**) were also well tolerated. Those bearing sterically more congested seven‐membered rings (**40** and **41**) and bridged heterocyclic systems (**42** and **43**) were also well tolerated. Acyclic ketones (**44**–**55**) decorated with distal functional groups were likewise competent substrates. In addition, bicyclo[1.1.1]pentane‐substituted ketones [7] (**54** and **55**) afforded the corresponding products, which are otherwise difficult to access but can now be prepared in a single step from commercially available building blocks.

Moreover, in addition to the primary‐, secondary‐, and tertiary‐alkylamines shown in Figure 2, this protocol enables the efficient synthesis of sterically congested α‐tertiary amines containing vicinal amino ether (**56**–**77**) and diamine (**78**–**88**) subunits (Figure 3A).

Remarkably, a wide range of unprotected and polar functional groups—including free alcohols (e.g., **26**), aryl halides (e.g., **33**), free phenol (**46**), alkyl azide (**52**), and terminal alkyne (**59**)—as well as aromatic heterocycles commonly used in medicinal chemistry, such as furan (**19**), thiophenes (e.g., **20**), *N*‐unprotected indole (**22**) and thiazole (**26**), pyridines (e.g., **27**), and imidazole (**64**)—were tolerated under these conditions, demonstrating the robustness of the method.

It is worth noting that solutions of gaseous amines enable access to hindered primary amines (**23**) and dimethylamino‐containing products (**86**), a widely exploited class in medicinal chemistry [71]. The broad substrate compatibility observed for primary and unsubstituted amines likely arises from acidic activation of the neutral ketimine by the HFIP [70] solvent or by Lewis‐acidic boron species [72] formed in the reaction medium.

To further demonstrate the synthetic utility and versatility of our approach, we adapted it for the late‐stage functionalization of advanced structures (Figure 3B). Compounds bearing carbonyl groups, such as pentoxifylline (**89**), lithocholic acid derivatives (**90** and **91**), and 5α‐cholestan‐3‐one (**92**), were successfully functionalized with alkyl and amino substituents in moderate‐to‐good yields. Moreover, amine‐containing molecules—including amlodipine (**93**), leelamine (**94**), paroxetine (**95**), desloratadine (**96**), norquetiapine (**97**), and medicinally relevant intermediates (**98**–**101**)—underwent efficient alkylation to directly install a C(*sp^3^*)‐rich quaternary center in a highly chemo‐selective manner.

**FIGURE 4:**
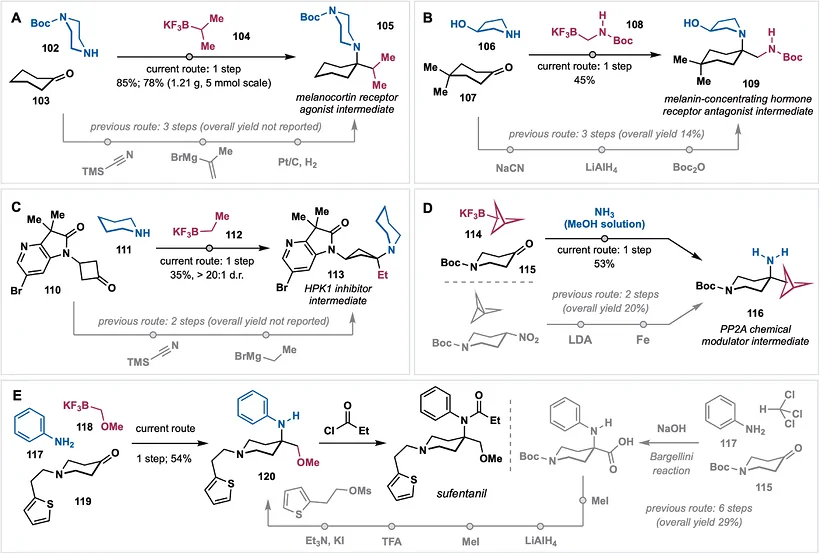
Synthetic applications. (A) Melanocortin receptor agonist intermediate **105**. (B) Melanin‐concentrating hormone receptor antagonist intermediate **109**. (C) HPK1 inhibitor intermediate **113**. (D) PP2A chemical modulator intermediate **116**. (E) Sufentanil intermediate **120**. See the Supporting Information for details.

Furthermore, as illustrated in Figure 4, we present five examples demonstrating how our approach can streamline the synthesis of hindered amine intermediates for drug candidates: (i) The melanocortin receptor agonist intermediate **105** has previously been synthesized from *N*‐Boc‐piperazine **102** and cyclohexanone **103** in three steps [73]. Alternatively, direct fragment assembly from the same starting material without using toxic and organometallic reagents, coupled with commercial trifluoroborate **104**, delivers the target in a single operation (85% yield) and remains efficient upon scale‐up to 5 mmol (78% yield). (ii) The melanin‐concentrating hormone receptor antagonist intermediate **109** is known to be prepared from (*S*)‐pyrrolidin‐3‐ol **106** and ketone **107** in 14% yield over three steps [74]. Our three‐component synthesis enables rapid access to **109** in a single step from the same starting material and **108**, affording an improved yield of 45%. (iii) The HPK1 inhibitor intermediate **113** was previously prepared using the standard Bruylants procedure [75]; in contrast, it can now be made in a single step without the need for toxic cyanide reagents. (iv) As an intermediate of a chemical modulator of PP2A, compound **116** was previously prepared via a two‐step route: addition of the alkyl lithium, generated by LDA deprotonation of *N*‐Boc 4‐nitropiperidine, to highly strained [1.1.1]propellane, followed by Fe‐mediated reduction, giving an overall yield of 20% [76]. Under our milder one‐step procedure, **116** was synthesized from commercial feedstocks in 53% isolated yield. (v) The recent patented synthesis of sufentanil—a painkiller used in anesthesia, labor and delivery, and for severe pain—proceeds in six steps, in which the quaternary center is constructed via the Bargellini reaction, affording the key intermediate **120** in 29% overall yield [77]. We present an alternative one‐step route to the same target, starting from aniline **117** and commercial trifluoroborate **118**, affording the known compound **119** in 54% yield.

**FIGURE 5:**
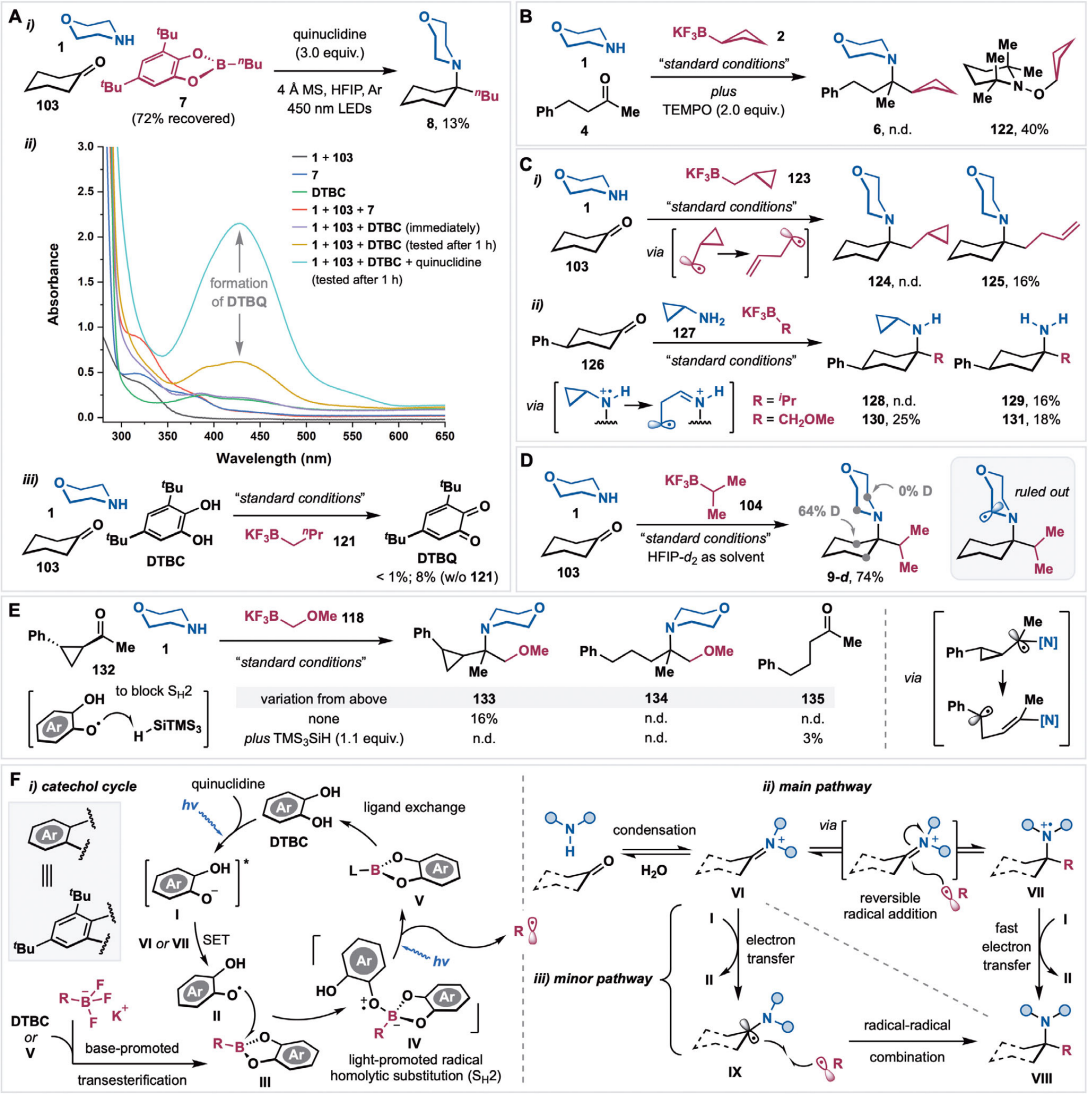
Mechanistic studies. (A) UV−vis spectroscopy analysis. (B) Radical trap experiment. (C) Radical clock experiments. (D) Excluding a stepwise interception process of aminium radical cations. (E) Probing the involvement of α‐amino carbon‐centered radicals. (F) Proposed reaction mechanism. n.d., not detected. See the Supporting Information for details.

Mechanistic studies were next performed to support the proposed three‐component assembly mechanism. UV–vis analysis (Figure 5A) indicates the formation of EDA complexes between the ketiminium ion and *B*‐alkylcatecholborane **7** or **DTBC**, as evidenced by the observation of the corresponding charge‐transfer (CT) bands [78, 79]. However, in each case, the CT band nearly overlaps with the corresponding local band within the wavelength range of the applied light source (∼400–500 nm), which indicates that the EDA complex may not be responsible for product formation. Subsequent exposure of **7**—in place of **DTBC** and **121**—to standard conditions afforded the amine products in 13% yield, further highlighting the inefficient electron transfer from CT band irradiation (Figure 5A‐i). Moreover, UV–vis spectrometry of an HFIP solution containing morpholine **1**, cyclohexanone **103**, and **DTBC**, which was stored in the dark for 1 h before analysis, showed the formation of 3,5‐di‐*tert*‐butyl‐*o*‐benzoquinone (**DTBQ**), and a stronger absorbance was observed in the presence of quinuclidine. These results illustrate that, in the absence of light, spontaneous electron transfer occurs between the ketiminium ion and **DTBC**, which exhibits higher reducing ability after deprotonation. Meanwhile, **DTBQ** was also detected under the standard conditions in the absence of trifluoroborate **121** (Figure 5A‐iii), whereas the interception of catechol radicals by *B*‑alkylcatecholborane species is presumably responsible for preventing the second electron transfer.

Next, the formation of alkyl radicals from alkyl organoboron species was confirmed by detecting adduct **122** in the presence of 2,2,6,6‐tetramethylpiperidin‐1‐oxyl (TEMPO) as a radical trap (Figure 5B), as well as ring‐opening product **125** when cyclopropylmethyl trifluoroborate **123** was used as the reaction partner (Figure 5C–i). To corroborate our proposed radical addition to the ketiminium intermediate pathway, ketone **126**, cyclopropylamine **127**—as a probe for aminium radical cations [80, 81]—and two different types of trifluoroborates were subjected to the standard reaction conditions (Figure 5C‐ii). In each case, the corresponding ring‑opened products (**129** and **131**) were isolated, but the detection of cyclopropyl product **130** suggests the possible co‑existence of a radical–radical combination pathway, which is shown to depend on the nature of the alkyl trifluoroborates employed. Furthermore, the observation of ring‐opened ketone product **135** (Figure 5E) underscores the involvement of *α*‐amino carbon‐centered radicals, for which the addition of (TMS)_3_SiH was employed to facilitate the hydrogen atom transfer event. However, the possible involvement of a radical‐chain mechanism cannot be ruled out.

Given the possibility that the aminium radical cation could undergo deprotonation by quinuclidine followed by polarity‐matched hydrogen atom transfer with **DTBC** [82, 83], we conducted a deuterium‐labeling experiment using HFIP‐*d*_2_ as the solvent (Figure 5D). No deuterium incorporation was detected at the *α*‐position to nitrogen, thereby excluding this pathway. On the other hand, because the aminium radical cations (*E* ^0^ ∼ 0.75 V vs. SCE) [84] generally have a higher reduction potential than the alkyl ketiminium ions (*E*_p_^red^ ∼ −1.68 V vs. SCE) [57], electron transfer from **DTBC** to the former should be feasible. However, the low conversion observed in the control experiments indicates that both light and quinuclidine are indispensable for rendering this transformation productive (entries 7 and 9; Figure 1D).

A plausible mechanism is proposed based on the above results (Figure 5F). The process is initiated by single‐electron transfer (SET) between the in situ–generated ketiminium ion **VI** and the photoexcited **DTBC** anion **I**, affording the *α*‐amino radical **IX** and the oxygen‐centered **DTBC** radical **II**. Subsequent coordination of intermediate **II** to *B*‐alkylcatecholborane **III** furnishes adduct **IV**, which liberates the alkyl radical upon light‐promoted homolytic cleavage of the B─C bond. Upon ligand substitution, the resulting boron‐ate complex **V** further liberates **DTBC** via reversible ligand exchange; alternatively, **III** may also form via direct transesterification of the alkyl trifluoroborate precursor with **V**. Finally, the amine product **VIII** is delivered via addition of the alkyl radical to **VI**, followed by a rapid SET event with **I**; in parallel, radical–radical cross‐coupling between **IX** and the alkyl radical constitutes a minor pathway.

## 3 Conclusion

In conclusion, we describe a mechanistically distinct three‐component assembly that exploits the dual functionality of catechol to enable the efficient, modular synthesis of hindered *α*‐tertiary aliphatic amines from readily available, bench‐stable precursors. This synthetic platform displays broad functional‐group tolerance and expands chemical space by enabling access to previously inaccessible substrate classes. This capability is expected to be highly valuable to chemists in both academic and industrial settings, as demonstrated by the late‐stage functionalization of advanced molecules and streamlined synthetic routes to pharmaceutically relevant intermediates.

## Conflicts of Interest

The authors declare no conflicts of interest.

## Supporting information




**Supporting File**: anie73649‐sup‐0001‐SuppMat.pdf.

## Data Availability

The data that support the findings of this study are available in the Supporting Information of this article.
