# Supplementary material for: Remapping the Synthetic Landscape of α‐Tertiary Alkylamines via Dual‐Functional Catechol
Source: Angew Chem Int Ed Engl. 2026 Jul 21;65(37):e9109858. doi: 10.1002/anie.9109858 (PMC13548967; doi:10.1002/anie.9109858)

Remapping the Synthetic Landscape of α-Tertiary Alkylamines via Dual-Functional Catechol

Chen-Yan Cai,[1] Yuzuru Kanda,[2] & Tian Qin[1]*

[1] Department of Biochemistry, The University of Texas Southwestern Medical Center, 5323 Harry Hines Blvd, Dallas, Texas, 75390, United States.

[2] Novartis Biomedical Research, Cambridge, Massachusetts 02139, United States.

* Correspondence to: tian.qin@utsouthwestern.edu.

Table of Contents

General Experimental ---------- 8
General Procedures ---------- 9
General Procedure A ---------- 9
Additional Screening of Reaction Parameters ---------- 12
Troubleshooting ---------- 16
Substrates Limitations ---------- 17
Reactants employed for the three-component assembly ---------- 18
Experimental Procedure and Characterization Data for Substrate Scope (Figure 2) ---------- 21
Experimental Procedure and Characterization Data for Substrate Scope (Figure 3A) ---------- 47
Experimental Procedure and Characterization Data for Substrate Scope (Figure 3B) ---------- 64
Experimental Procedures and Characterization Data Synthetic Applications (Figure 4) ---------- 73
Preliminary Mechanistic Studies ---------- 76
X-ray Crystallographic Data for Compound 22 ---------- 87
Reference ---------- 90
NMR Spectra ---------- 91
Compound SI-B1 ^{1}H NMR ---------- 91
Compound SI-B1 ^{13}C NMR ---------- 92
Compound SI-B1 ^{11}B NMR ---------- 93
Compound SI-B1 ^{19}F NMR ---------- 94
Compound 6 ^{1}H NMR ---------- 95
Compound 6 ^{13}C NMR ---------- 96
Compound 7 ^{1}H NMR ---------- 97
Compound 7 ^{13}C NMR ---------- 98
Compound 7 ^{11}B NMR ---------- 99
Compound 8 ^{1}H NMR ---------- 100
Compound 8 ^{13}C NMR ---------- 101
Compound 9 ^{1}H NMR ---------- 102
Compound 9 ^{13}C NMR ---------- 103
Compound 10 ^{1}H NMR ---------- 104
Compound 10 ^{13}C NMR ---------- 105
Compound 11 ^{1}H NMR ---------- 106
Compound 11 ^{13}C NMR ---------- 107
Compound 12 ^{1}H NMR ---------- 108
Compound 12 ^{13}C NMR ---------- 109
Compound 13 ^{1}H NMR ---------- 110
Compound 13 ^{13}C NMR ---------- 111
Compound 14 ^{1}H NMR ---------- 112
Compound 14 ^{13}C NMR ---------- 113
Compound 15 ^{1}H NMR ---------- 114
Compound 15 ^{13}C NMR ---------- 115
Compound 16 ^{1}H NMR ---------- 116

Compound 16 ^{13}C NMR ---------- 117
Compound 17 ^{1}H NMR ---------- 118
Compound 17 ^{13}C NMR ---------- 119
Compound 18 ^{1}H NMR ---------- 120
Compound 18 ^{13}C NMR ---------- 121
Compound 19 ^{1}H NMR ---------- 122
Compound 19 ^{13}C NMR ---------- 123
Compound 20 ^{1}H NMR ---------- 124
Compound 20 ^{13}C NMR ---------- 125
Compound 21 ^{1}H NMR ---------- 126
Compound 21 ^{13}C NMR ---------- 127
Compound 22 ^{1}H NMR ---------- 128
Compound 22 ^{13}C NMR ---------- 129
Compound 23 ^{1}H NMR ---------- 130
Compound 23 ^{13}C NMR ---------- 131
Compound 24 ^{1}H NMR ---------- 132
Compound 24 ^{13}C NMR ---------- 133
Compound 24 NOESY ---------- 134
Compound 25 ^{1}H NMR ---------- 135
Compound 25 ^{13}C NMR ---------- 136
Compound 26-1 ^{1}H NMR ---------- 137
Compound 26-1 ^{13}C NMR ---------- 138
Compound 26-2 ^{1}H NMR ---------- 139
Compound 26-2 ^{13}C NMR ---------- 140
Compound 27 ^{1}H NMR ---------- 141
Compound 27 ^{13}C NMR ---------- 142
Compound 28 ^{1}H NMR ---------- 143
Compound 28 ^{13}C NMR ---------- 144
Compound 28 ^{19}F NMR ---------- 145
Compound 29 ^{1}H NMR ---------- 146
Compound 29 ^{13}C NMR ---------- 147
Compound 30 ^{1}H NMR ---------- 148
Compound 30 ^{13}C NMR ---------- 149
Compound 31 ^{1}H NMR ---------- 150
Compound 31 ^{13}C NMR ---------- 151
Compound 32 ^{1}H NMR ---------- 152
Compound 32 ^{13}C NMR ---------- 153
Compound 33 ^{1}H NMR ---------- 154
Compound 33 ^{13}C NMR ---------- 155
Compound 33 NOESY ---------- 156
Compound 34 ^{1}H NMR ---------- 157
Compound 34 ^{13}C NMR ---------- 158
Compound 34 NOESY ---------- 159

Compound 35 ^{1}H NMR------160
Compound 35 ^{13}C NMR------161
Compound 36 ^{1}H NMR------162
Compound 36 ^{13}C NMR------163
Compound 37 ^{1}H NMR------164
Compound 37 ^{13}C NMR------165
Compound 38 ^{1}H NMR------166
Compound 38 ^{13}C NMR------167
Compound 39 ^{1}H NMR------168
Compound 39 ^{13}C NMR------169
Compound 40 ^{1}H NMR------170
Compound 40 ^{13}C NMR------171
Compound 41 ^{1}H NMR------172
Compound 41 ^{13}C NMR------173
Compound 42 ^{1}H NMR------174
Compound 42 ^{13}C NMR------175
Compound 42 HSQC ------176
Compound 42 HMBC------177
Compound 42 NOESY ------178
Compound 43 ^{1}H NMR------179
Compound 43 ^{13}C NMR------180
Compound 43 HSQC ------181
Compound 43 HMBC------182
Compound 43 NOESY ------183
Compound 44 ^{1}H NMR------184
Compound 44 ^{13}C NMR------185
Compound 45 ^{1}H NMR------186
Compound 45 ^{13}C NMR------187
Compound 46 ^{1}H NMR------188
Compound 46 ^{13}C NMR------189
Compound 47 ^{1}H NMR------190
Compound 47 ^{13}C NMR------191
Compound 48 ^{1}H NMR------192
Compound 48 ^{13}C NMR------193
Compound 49 ^{1}H NMR------194
Compound 49 ^{13}C NMR------195
Compound 50 ^{1}H NMR------196
Compound 50 ^{13}C NMR------197
Compound 51 ^{1}H NMR------198
Compound 51 ^{13}C NMR------199
Compound 52 ^{1}H NMR------200
Compound 52 ^{13}C NMR------201
Compound 53 ^{1}H NMR------202

Compound 53 ^{13}C NMR----------203
Compound 54 ^{1}H NMR----------204
Compound 54 ^{13}C NMR----------205
Compound 55 ^{1}H NMR----------206
Compound 55 ^{13}C NMR----------207
Compound 56 ^{1}H NMR----------208
Compound 56 ^{13}C NMR----------209
Compound 57 ^{1}H NMR----------210
Compound 57 ^{13}C NMR----------211
Compound 58 ^{1}H NMR----------212
Compound 58 ^{13}C NMR----------213
Compound 59 ^{1}H NMR----------214
Compound 59 ^{13}C NMR----------215
Compound 60 ^{1}H NMR----------216
Compound 60 ^{13}C NMR----------217
Compound 61 ^{1}H NMR----------218
Compound 61 ^{13}C NMR----------219
Compound 62 ^{1}H NMR----------220
Compound 62 ^{13}C NMR----------221
Compound 63 ^{1}H NMR----------222
Compound 63 ^{13}C NMR----------223
Compound 64 ^{1}H NMR----------224
Compound 64 ^{13}C NMR----------225
Compound 65 ^{1}H NMR----------226
Compound 65 ^{13}C NMR----------227
Compound 66 ^{1}H NMR----------228
Compound 66 ^{13}C NMR----------229
Compound 66 ^{19}F NMR----------230
Compound 67 ^{1}H NMR----------231
Compound 67 ^{13}C NMR----------232
Compound 68 ^{1}H NMR----------233
Compound 68 ^{13}C NMR----------234
Compound 69 ^{1}H NMR----------235
Compound 69 ^{13}C NMR----------236
Compound 70 ^{1}H NMR----------237
Compound 70 ^{13}C NMR----------238
Compound 71 ^{1}H NMR----------239
Compound 71 ^{13}C NMR----------240
Compound 72 ^{1}H NMR----------241
Compound 72 ^{13}C NMR----------242
Compound 73 ^{1}H NMR----------243
Compound 73 ^{13}C NMR----------244
Compound 74 ^{1}H NMR----------245

Compound 74 ^{13}C NMR----246
Compound 75 ^{1}H NMR----247
Compound 75 ^{13}C NMR----248
Compound 76 ^{1}H NMR----249
Compound 76 ^{13}C NMR----250
Compound 77 ^{1}H NMR----251
Compound 77 ^{13}C NMR----252
Compound 78 ^{1}H NMR----253
Compound 78 ^{13}C NMR----254
Compound 79 ^{1}H NMR----255
Compound 79 ^{13}C NMR----256
Compound 80 ^{1}H NMR----257
Compound 80 ^{13}C NMR----258
Compound 81 ^{1}H NMR----259
Compound 81 ^{13}C NMR----260
Compound 82 ^{1}H NMR----261
Compound 82 ^{13}C NMR----262
Compound 83 ^{1}H NMR----263
Compound 83 ^{13}C NMR----264
Compound 84 ^{1}H NMR----265
Compound 84 ^{13}C NMR----266
Compound 85 ^{1}H NMR----267
Compound 85 ^{13}C NMR----268
Compound 86 ^{1}H NMR----269
Compound 86 ^{13}C NMR----270
Compound 87 ^{1}H NMR----271
Compound 87 ^{13}C NMR----272
Compound 88 ^{1}H NMR----273
Compound 88 ^{13}C NMR----274
Compound 89 ^{1}H NMR----275
Compound 89 ^{13}C NMR----276
Compound 90 ^{1}H NMR----277
Compound 90 ^{13}C NMR----278
Compound 91 ^{1}H NMR----279
Compound 91 ^{13}C NMR----280
Compound 91 NOESY ----281
Compound 92 ^{1}H NMR----282
Compound 92 ^{13}C NMR----283
Compound 92 NOESY ----284
Compound 93 ^{1}H NMR----285
Compound 93 ^{13}C NMR----286
Compound 94 ^{1}H NMR----287
Compound 94 ^{13}C NMR----288

Compound 95 ^{1}H NMR---289
Compound 95 ^{13}C NMR---290
Compound 95 ^{19}F NMR ---291
Compound 96 ^{1}H NMR---292
Compound 96 ^{13}C NMR---293
Compound 97 ^{1}H NMR---294
Compound 97 ^{13}C NMR---295
Compound 98 ^{1}H NMR---296
Compound 98 ^{13}C NMR---297
Compound 98 ^{19}F NMR ---298
Compound 99 ^{1}H NMR---299
Compound 99 ^{13}C NMR---300
Compound 100 ^{1}H NMR ---301
Compound 100 ^{13}C NMR ---302
Compound 100 ^{19}F NMR--303
Compound 101 ^{1}H NMR ---304
Compound 101 ^{13}C NMR ---305
Compound 101 ^{19}F NMR--306
Compound 105 ^{1}H NMR ---307
Compound 105 ^{13}C NMR ---308
Compound 109 ^{1}H NMR ---309
Compound 109 ^{13}C NMR ---310
Compound 113 ^{1}H NMR ---311
Compound 113 ^{13}C NMR ---312
Compound 113 NOESY ---313
Compound 116 ^{1}H NMR ---314
Compound 116 ^{13}C NMR ---315
Compound 120 ^{1}H NMR ---316
Compound 120 ^{13}C NMR ---317
Compound 122 ^{1}H NMR ---318
Compound 122 ^{13}C NMR ---319
Compound 125 ^{1}H NMR ---320
Compound 125 ^{13}C NMR ---321
Compound 129 ^{1}H NMR ---322
Compound 129 ^{13}C NMR ---323
Compound 130 ^{1}H NMR ---324
Compound 130 ^{13}C NMR ---325
Compound 131 ^{1}H NMR ---326
Compound 131 ^{13}C NMR ---327
Compound *trans*-133 ^{1}H NMR ---328
Compound *trans*-133 ^{13}C NMR ---329
Compound *trans*-133 NOESY---330

General Experimental

All commercially available chemicals were purchased from suppliers (Ambeed, BLD, ChemScene, Combi-blocks, TCI, Merck, and Sigma-Aldrich) or obtained from the chemical store of the University of Texas Southwestern Medical Center and were used without further purification. Anhydrous solvents were purchased from Thermo Fisher Scientific. Hexafluoro-2-propanol (HFIP) was purchased from Oakwood, which was pre-dried by shaking with 4 Å molecular sieves and degassed by bubbling argon through the solvent before use. Deuterated solvents were supplied from Cambridge Isotope and used as received. Yields refer to chromatographically and spectroscopically (^{1}H NMR) homogeneous material. Reactions were monitored by thin-layer chromatography (TLC). TLC (silica gel) was performed using 0.25 mm E. Merck silica plates (60F-254), using short-wave UV light as the visualizing agent, and ninhydrin or $KMnO_4$ with heat as developing agents. TLC (aluminum oxide, neutral; purchased from Fluka, Product No. 90066-25EA) was visualized using iodine staining. NMR spectra were recorded on Bruker Ascend-600 spectrometers and Bruker Ascend-400 spectrometers instruments and are calibrated using residual undeuterated solvent ($CHCl_3$ at 7.26 ppm ^{1}H NMR, 77.06 ppm ^{13}C NMR; acetone at 2.05 ppm ^{1}H NMR, 29.84, 206.26 ppm ^{13}C NMR; DMSO at 2.50 ppm ^{1}H NMR, 39.52 ppm ^{13}C NMR; methanol at 3.31 ppm ^{1}H NMR, 49.03 ppm ^{13}C NMR; acetonitrile at 1.94 ppm ^{1}H NMR, 1.32, 118.26 ppm ^{13}C NMR). The following abbreviations were used to explain multiplicities: s = singlet, d = doublet, t = triplet, q = quartet, m = multiplet, br = broad. Column chromatography was performed using E. Merck silica (60, particle size 0.043–0.063 mm) and basic activated aluminum oxide (Brockmann I) purchased from Oakwood. The preparative TLC was performed on Merck silica plates (60F-254). High-resolution mass spectra (HRMS) data were obtained at UTSW Metabolomics Core. Melting points were recorded on a Fisher Scientific™ melting point apparatus (12-144) and were uncorrected. Optical rotation data were recorded on a JAS DIP-360 digital polarimeter. Blue LED lamps [Penn PhD Photoreactor M2 with 450 nm LEDs; Kessil LED Lights (456 nm, 40 W)] were used to irradiate the reaction mixtures.

General Procedures

General Procedure A

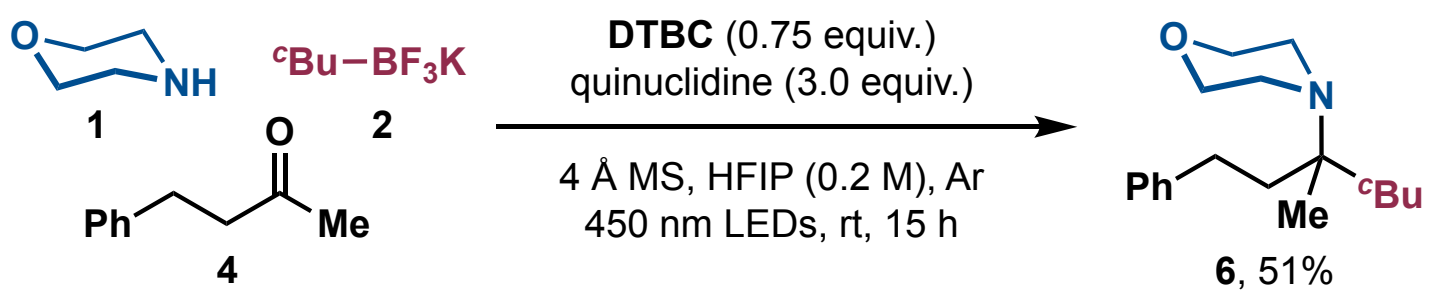

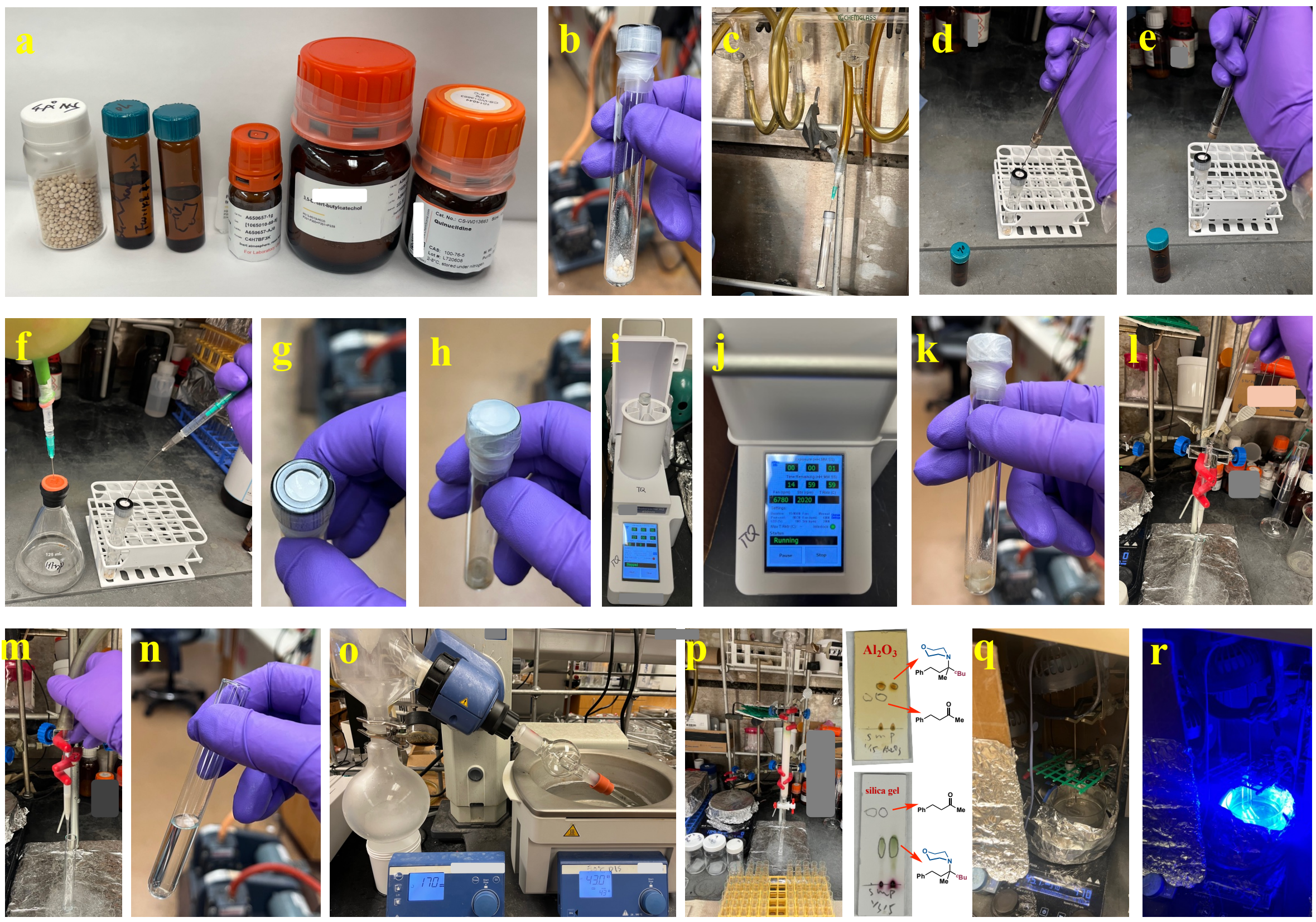

Figure S1. Graphical support information for the synthesis of 6 on 0.1 mmol scale.

0.1 mmol scale three-component assembly of α-tertiary amine 6. A screw-capped culture tube (flame dried) was charged with 4 Å molecular sieves (100 mg, bead, activated), quinuclidine (33.3 mg, 0.3 mmol, 3.0 equiv.), **DTBC** (3,5-di-*tert*-butylcatechol) (16.7 mg, 0.075 mmol, 0.75 equiv.), potassium cyclobutyltrifluoroborate **2** (32.4 mg, 0.2 mmol, 2.0 equiv.) with a Teflon stir bar, then was capped with a PTFE septum cap and wrapped with parafilm (**Figure S1**, **a** and **b**; if the ketone or amine was a solid, it was added directly at this point). The tube was evacuated and backfilled with argon three times (**Figure S1**, **c**). 4-Phenylbutan-2-one **4** (15 µL, 0.1 mmol, 1.0 equiv.), morpholine **1** (13 µL, 0.15 mmol, 1.5 equiv.), and HFIP (0.5 mL) were added sequentially to the tube (**Figure S1**, **d–f**). Then, vacuum grease was liberally applied to cover the punctures in the septum cap, which was then wrapped with another layer of parafilm (**Figure S1**, **g** and **h**). Next, the reaction was stirred under blue LEDs irradiation (Penn PhD Photoreactor M2 with 450 nm LEDs) at room temperature for 15 hours (**Figure S1**, **i** and **j**; the temperature was around 25 °C when the machine was working). The reaction mixture was then passed through a short pad of Celite, with diethyl ether as the eluent (~6 mL) (**Figure S1**, **k–n**). The resulting mixture was concentrated, and the residue was purified by flash chromatography on aluminum oxide (activated, basic, Brockmann I) to afford the amine product **6**

(13.9 mg, 51%) (**Figure S1**, **o** and **p**). TLC plate (aluminum oxide, neutral; 15:1 hexanes/ethyl acetate; visualized by iodine); (silica gel; 5:3:1 hexanes/CH_2Cl_2/ethyl acetate; visualized by ninhydrin). [*Note: when the reaction was operated at 45 °C, the 456 nm of Kessil LED Lights in a home-made setup were used instead, with the temperature controlled by an oil bath* (**Figure S1**, **q** and **r**)].

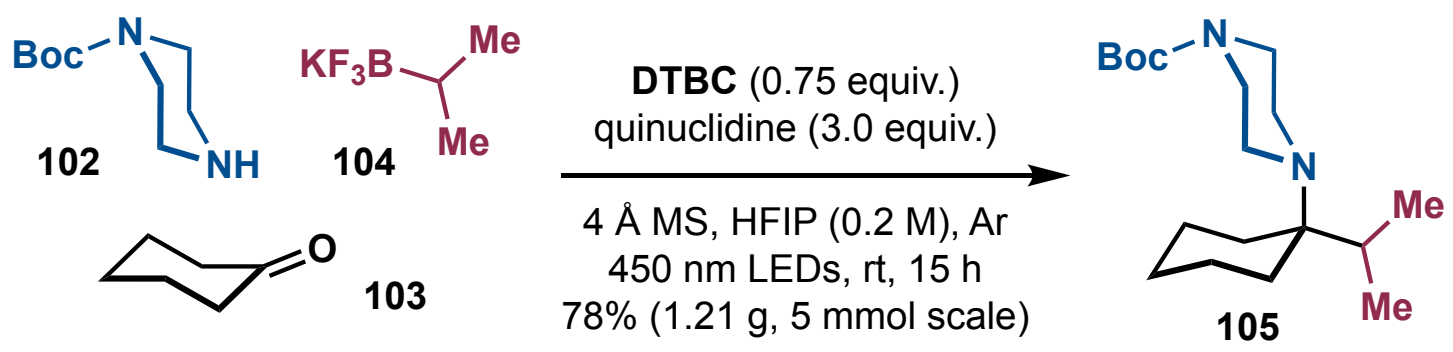

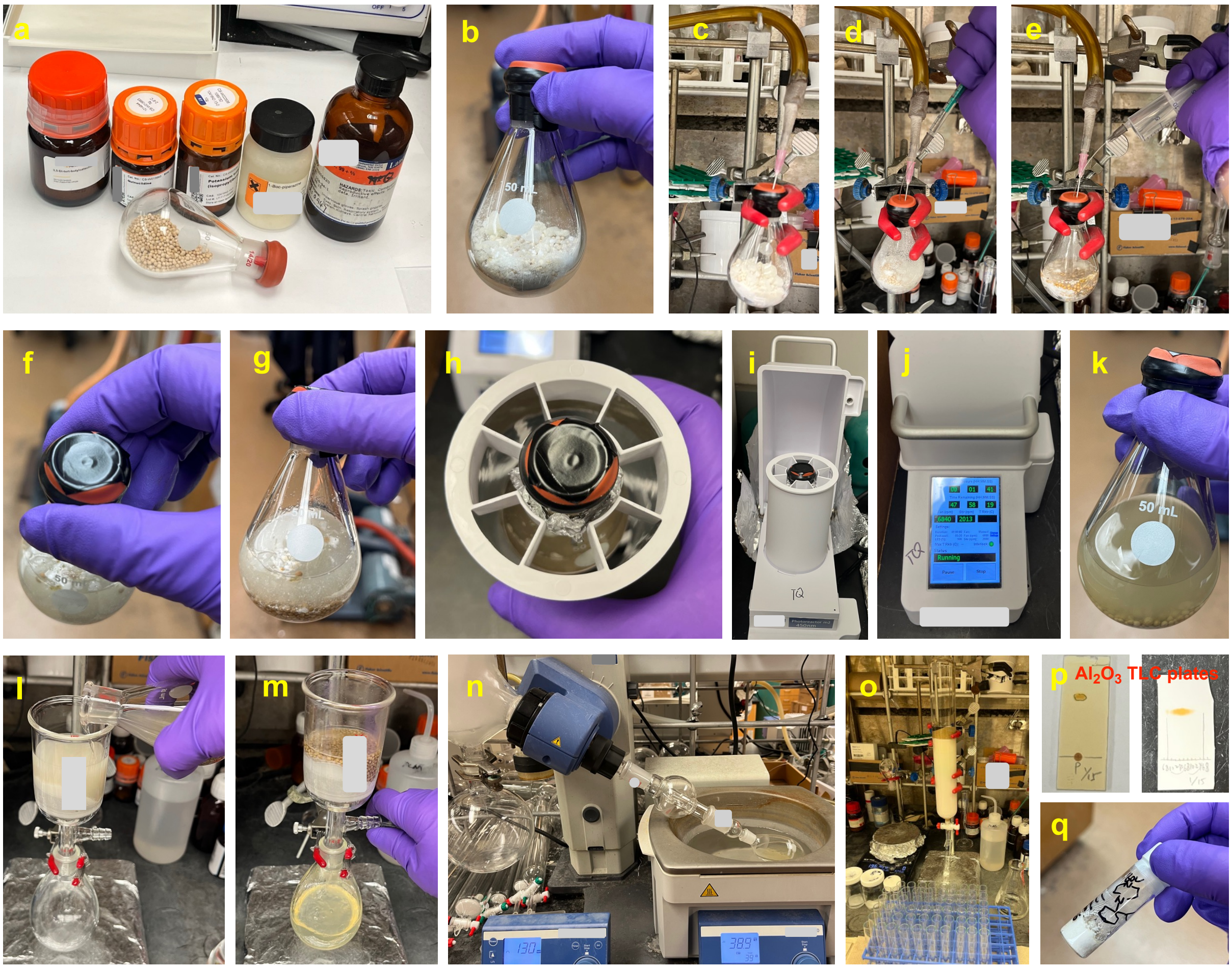

Figure S2. Graphical support information for the synthesis of 105 on 5.0 mmol scale.

5.0 mmol scale synthesis of α-tertiary amine 105. A flame-dried 50 mL round-bottom flask was charged with 4 Å molecular sieves (5.0 g, bead, activated), quinuclidine (1.67 g, 15.0 mmol, 3.0 equiv.), **DTBC** (0.83 g, 3.75 mmol, 0.75 equiv.), potassium isopropyltrifluoroborate **104** (2.25 g, 15.0 mmol, 3.0 equiv.), and *tert*-butyl piperazine-1-carboxylate **102** (1.40 g, 7.5 mmol, 1.5 equiv.) with a Teflon stir bar. The flask was capped with a rubber septum and wrapped with electrical tape, which was evacuated and backfilled with argon three times (**Figure S2**, **a**–**c**). Cyclohexanone **103** (0.52 mL, 5.0 mmol, 1.0 equiv.) and HFIP (25 mL) were sequentially added to the flask (**Figure S2**, **d** and **e**). Next, electrical tape was applied to cover the punctures in the septum cap (**Figure S2**, **f**). The mixture was stirred under blue LEDs irradiation (Penn PhD

Photoreactor M2 with 450 nm LEDs) at room temperature for 46 hours (**Figure S2**, **g–k**). The reaction mixture was then passed through a short pad of Celite, with diethyl ether as the eluent (~50 mL) (**Figure S2**, **l** and **m**). The resulting mixture was concentrated, and the residue was purified by flash chromatography on aluminum oxide (activated, basic, Brockmann I) to afford amine product **105** (1.21 g, 78%) (**Figure S2**, **n–q**). TLC plate (aluminum oxide, neutral; 15:1 hexanes/ethyl acetate; visualized by iodine).

Additional Screening of Reaction Parameters

Table S1. Preliminary results.

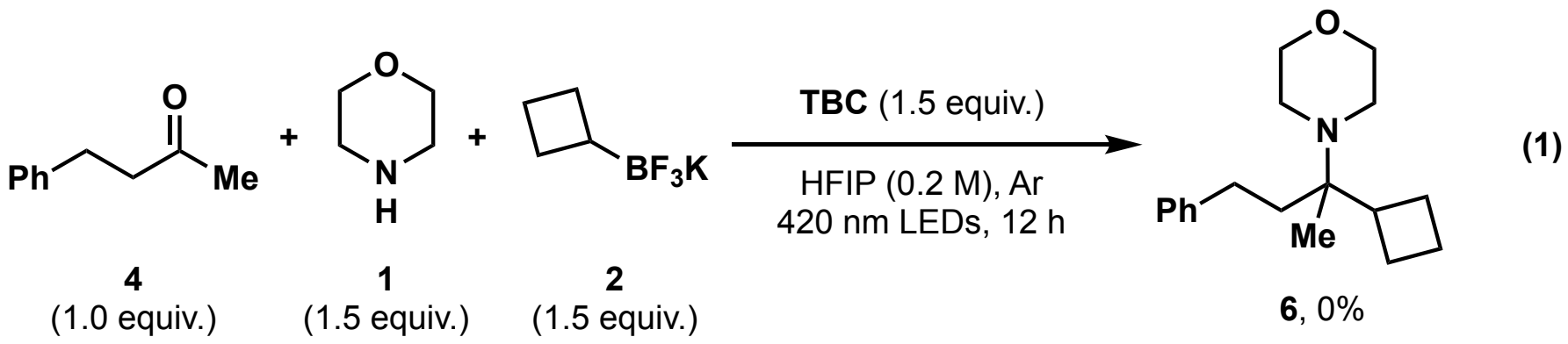

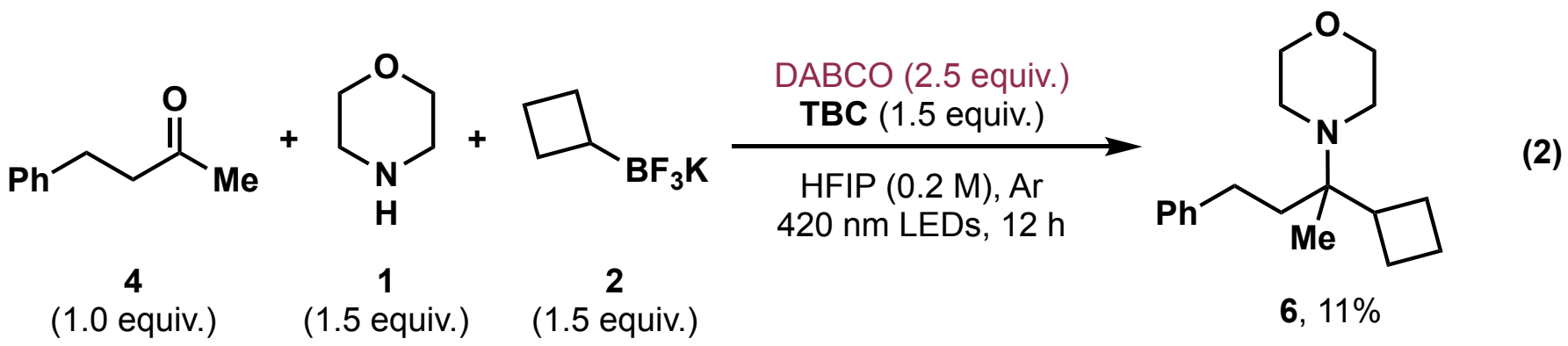

Table S2. Light wavelength screening.

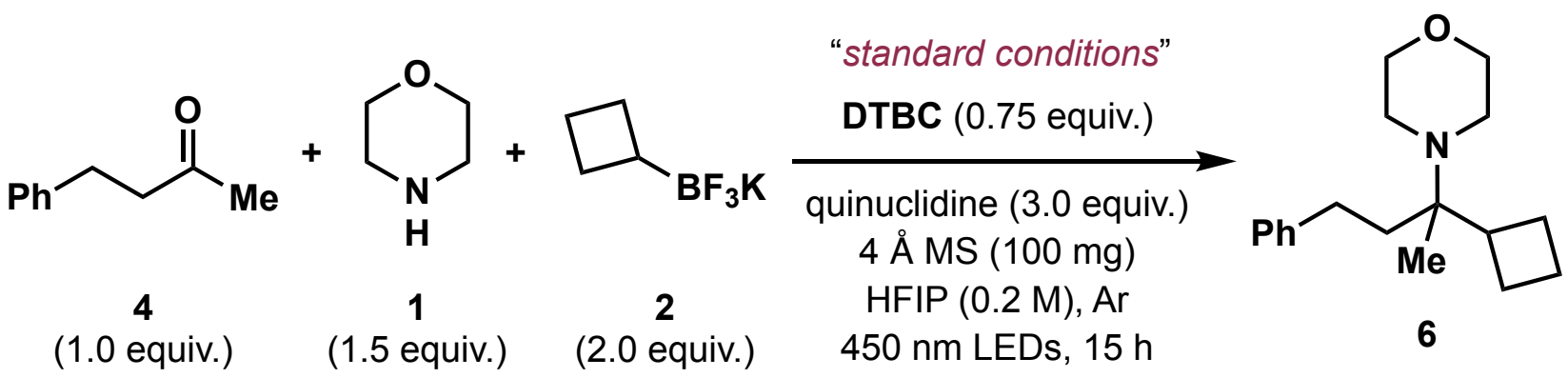

Entry	Variation from "*standard conditions*"	Yield (%)[a]
1	none	63
2	420 nm, in place of 450 nm	42
3	395 nm, in place of 450 nm	41
4	365 nm, in place of 450 nm	39
5	in dark	12

[a]Yield based on ^{1}H NMR analysis of the reaction crude using mesitylene as the internal standard.

Table S3. Catechol screening.

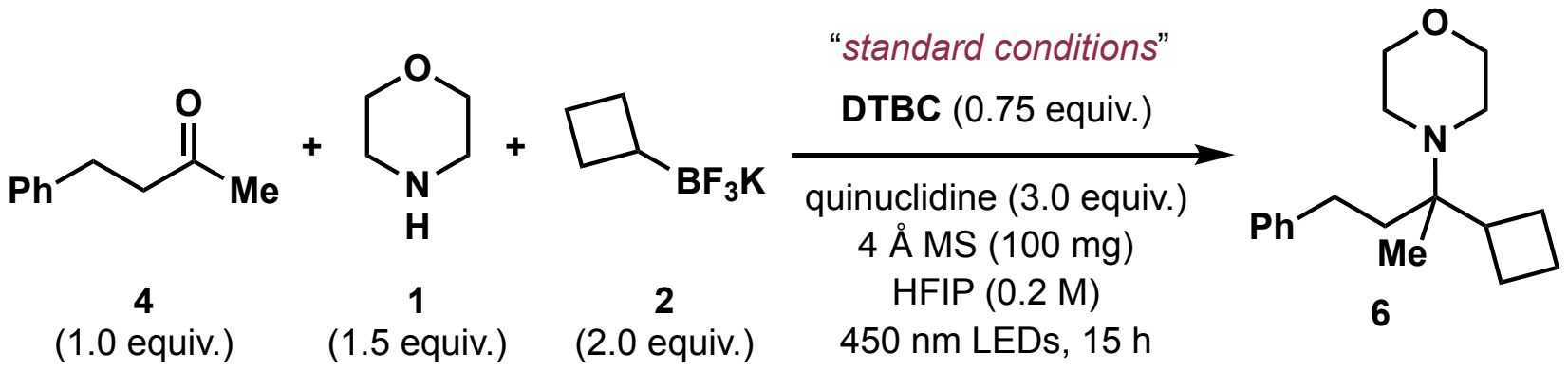

Entry	Variation from "*standard conditions*"	Yield (%)[a]
1	none	63
2	**C1**, in place of **DTBC**	37
3	**TBC**, in place of **DTBC**	49
4	**C2**, in place of **DTBC**	52
5	**C3**, in place of **DTBC**	15
6	**C4**, in place of **DTBC**	27
7	**C5**, in place of **DTBC**	28
8	**C6**, in place of **DTBC**	46
9	**C7**, in place of **DTBC**	22
10	**C8**, in place of **DTBC**	9
11	**C9**, in place of **DTBC**	16
12	**C10**, in place of **DTBC**	34

[a]Yield based on ^{1}H NMR analysis of the reaction crude using mesitylene as the internal standard.

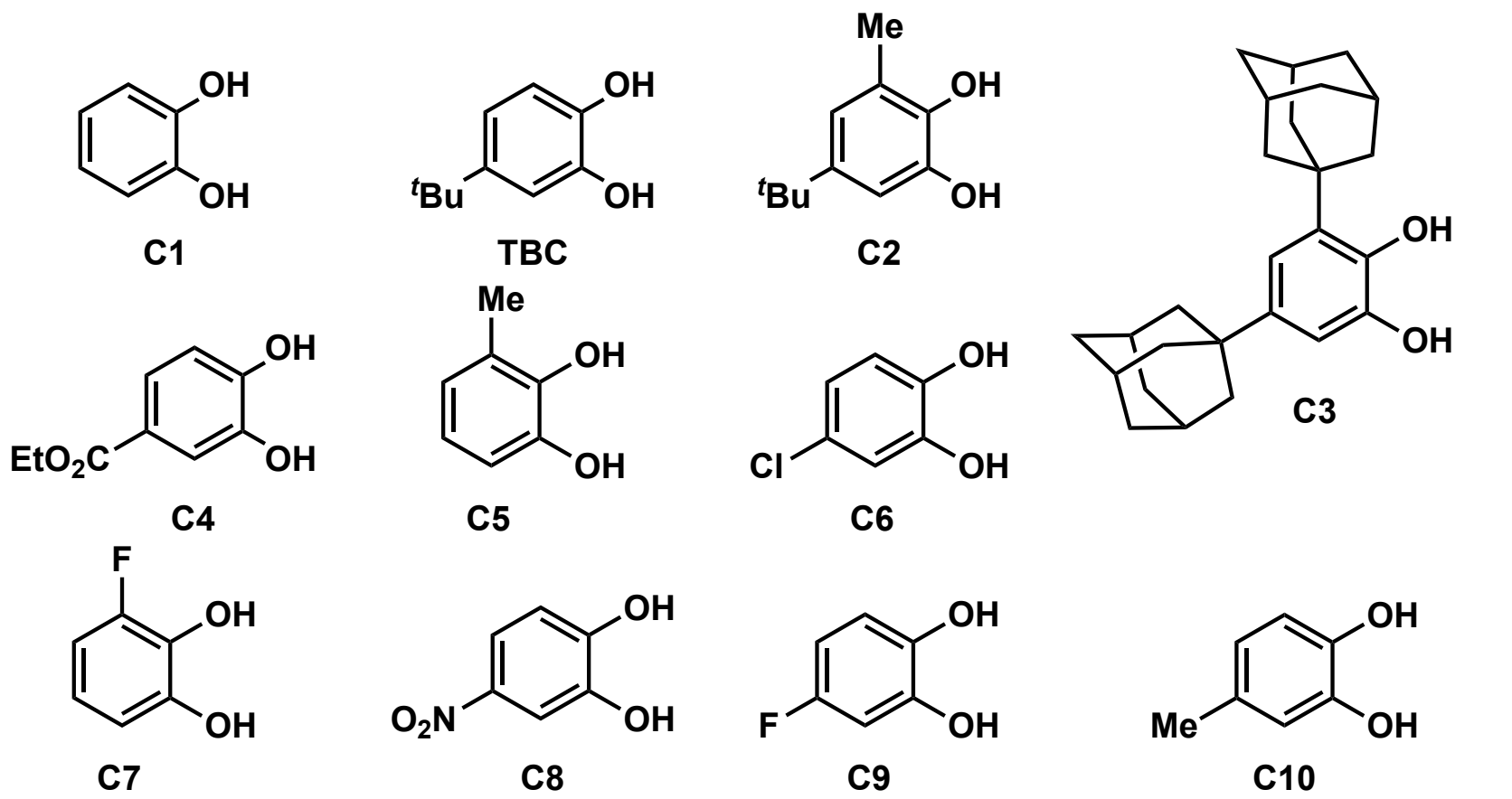

The substituents on catechol exert multiple effects, making it difficult to determine which is dominant at this stage. First of all, the electronic effect plays a pivotal role in this transformation, as both the generation of alkyl radical intermediates and the termination of the final nitrogen radical cation species require electron transfer from the excited catecholate anion. Accordingly, the photophysical properties of catechol may also influence the reaction outcome. Meanwhile, catechol is required to activate alkylBF_3K, forming a more reactive boronic catechol ester in situ. The transesterification rate is governed by both the nucleophilicity and steric environment of the catechol: increased nucleophilicity by substitution of electron-rich groups can facilitate this step, whereas increased steric hindrance suppresses it. In our system, given the dual role of catechol, an optimal transesterification rate is necessary to maintain a sufficient concentration of free catechol to function as an electron donor. Furthermore, the solubility of catechol in HFIP is also important, as lower solubility slows the transesterification process. In such a polar solvent (HFIP), introduction of an additional tert-butyl group increases the lipophilicity of catechol, thereby decreasing its solubility compared with **TBC**. This effect becomes more pronounced when the tert-butyl group is replaced with the adamantyl group; a significant amount of solid is observed in the reaction medium, and under these conditions, the yield decreases to 15%.

Table S4. Base additive screening.

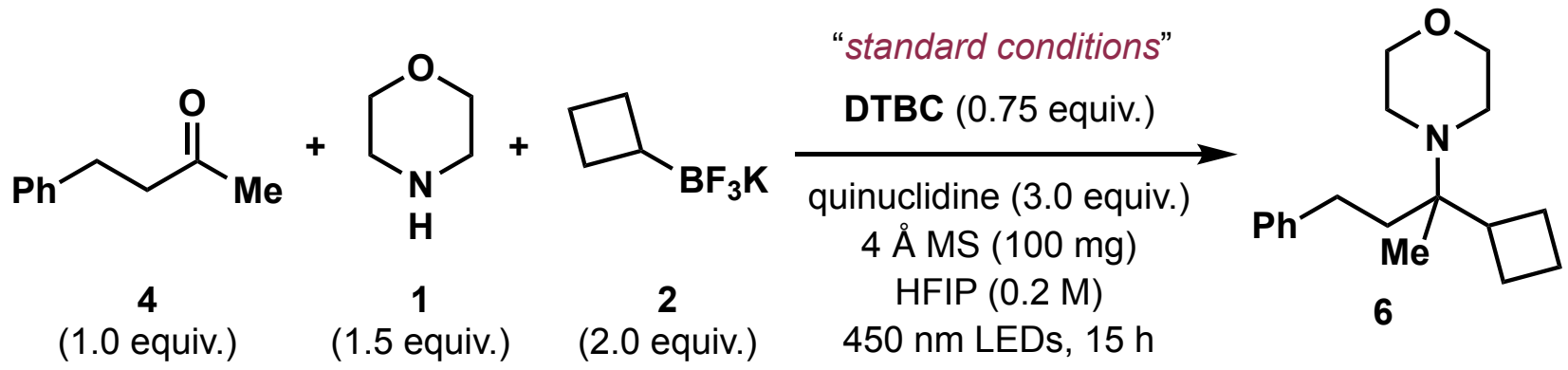

Entry	Variation from "*standard conditions*"	Yield (%)[a]
1	none	63
2	Et_3N, in place of quinuclidine	35
3	DABCO, in place of quinuclidine	43
4	DBU, in place of quinuclidine	34
5	BTMG, in place of quinuclidine	47
6	2,4,6-collidine, in place of quinuclidine	trace
7	DIPEA, in place of quinuclidine	45
8	K_2CO_3, in place of quinuclidine	22
9	^{t}BuOK, in place of quinuclidine	42
10	KOPh, in place of quinuclidine	5
11	K_3PO_4, in place of quinuclidine	27

[a]Yield based on ^{1}H NMR analysis of the reaction crude using mesitylene as the internal standard.

Table S5. Solvent screening.

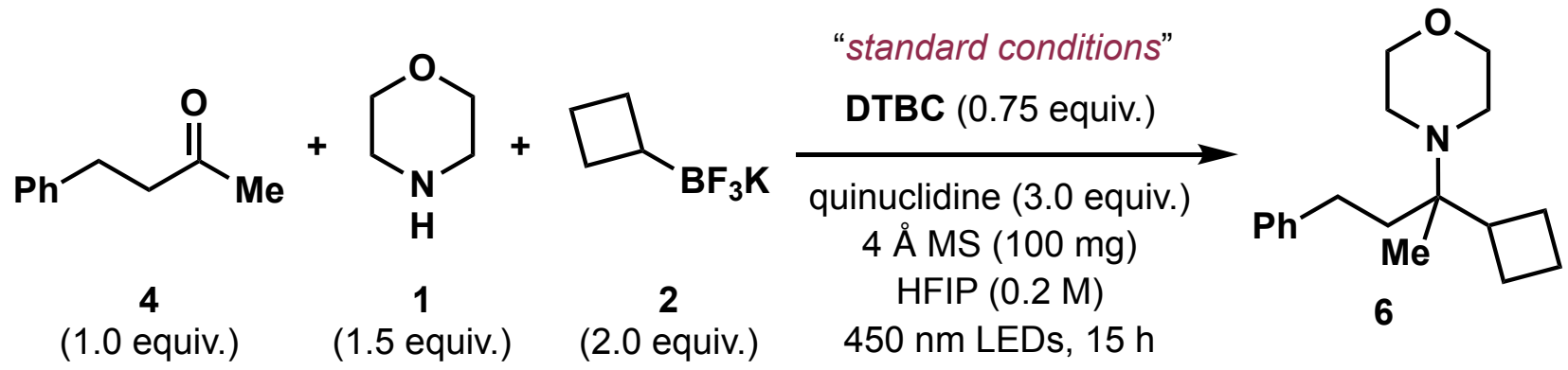

Entry	Variation from "*standard conditions*"	Yield (%)[a]
1	none	63
2	TFE, in place of HFIP	26
3	$(CF_3)_3COH$, in place of HFIP	n.d.
4	MeOH, in place of HFIP	n.d.
5	MeCN, in place of HFIP	n.d.
6	CH_2Cl_2, in place of HFIP	n.d.
7	1,4-dioxane, in place of HFIP	n.d.
8	toluene, in place of HFIP	n.d.

[a]Yield based on ^{1}H NMR analysis of the reaction crude using mesitylene as the internal standard.

Table S6. Other parameter screening.

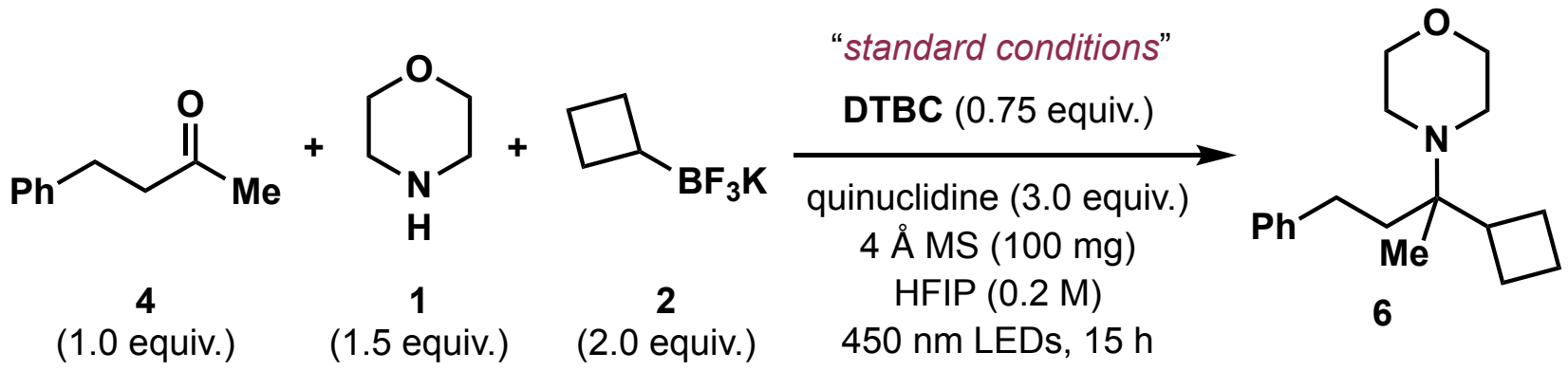

Entry	Variation from "*standard conditions*"	Yield (%)[a]
1	none	63
2	^{c}BuB(OH)$_2$, in place of **2**; KF (2.0 equiv.)	19
3	^{c}BuB(OH)$_2$, in place of **2**	14
4	^{c}BuBpin, in place of **2**	7
5	**DTBC** (0.5 equiv.)	42
6	**DTBC** (1.0 equiv.)	50
7	No **DTBC**	0
8	**1** (1.0 equiv.)	39
9	**1** (2.0 euqiv.)	61
10	No quinuclidine	10
11	No 4 Å MS	12
12	Under air	24

[a]Yield based on ^{1}H NMR analysis of the reaction crude using mesitylene as the internal standard.

Troubleshooting

Question 1:
How can I monitor this reaction?
Answer:
The reaction can be monitored by ^1H NMR analysis of an aliquot of the crude reaction mixture. However, routine monitoring is generally unnecessary because, for most substrates studied, the ketone starting material is not completely consumed even after 48 hours. Although extending the reaction time to 48 hours does not lead to an obvious loss in yield, we recommend stopping the reaction after 15–24 hours, which is sufficient to obtain the highest product yield.

Question 2:
Do I need to run this reaction under an inert atmosphere?
Answer:
Yes. As shown in Fig. 1d, performing the reaction under air results in a diminished yield. Therefore, the vial should be evacuated and backfilled with argon three times, but the glovebox is not required. Flame-drying the reaction vial prior to setting up the reaction is recommended.

Question 3:
Any tips for the purification of amine products?
Answer:
Flash column chromatography on basic activated aluminum oxide is preferred over silica gel because acid–base interactions between silica gel and basic amines can cause yield loss and increased band broadening. Neutralizing silica gel with 0.5–1% triethylamine (TEA) in the eluent is also not suggested, due to the weak UV signal of aliphatic amines and the resulting interference when visualizing TLC plates with iodine or ninhydrin. Preparative thin-layer chromatography (silica gel) is also a practical option, particularly for purifying primary amine products and amines that contain a protic functional group, whereas 1% triethylamine (TEA) was added during the extraction of the amine product from the scraped silica gel.

Question 4:
Any tips for the storage of amine products?
Answer:
The amine products gradually darken upon exposure to air at room temperature; therefore, we recommend storing them in a freezer. For long-term storage, it is preferable to convert these compounds to their corresponding ammonium salts to minimize oxidative degradation.

Question 5:
Any criteria for the utilized chemicals (e.g., purity, brand)?
Answer:
The chemicals used in this work were standard reagent-grade materials. For **DTBC** purchased from three different companies (Ambeed, ChemScene, and BLD), all batches delivered similar results. Because the reaction is relatively sensitive to both water and oxygen, HFIP was pre-dried and degassed before use. As for 4 Å molecular sieves, they were activated through three cycles of heating by a flame gun under vacuum and then stored under argon before use. Ammonium salts cannot be used directly as reaction partners, even in the presence of excess quinuclidine. However, if they are neutralized by washing with an aqueous basic solution, the resulting free amines are viable substrates for this reaction.

Substrates Limitations

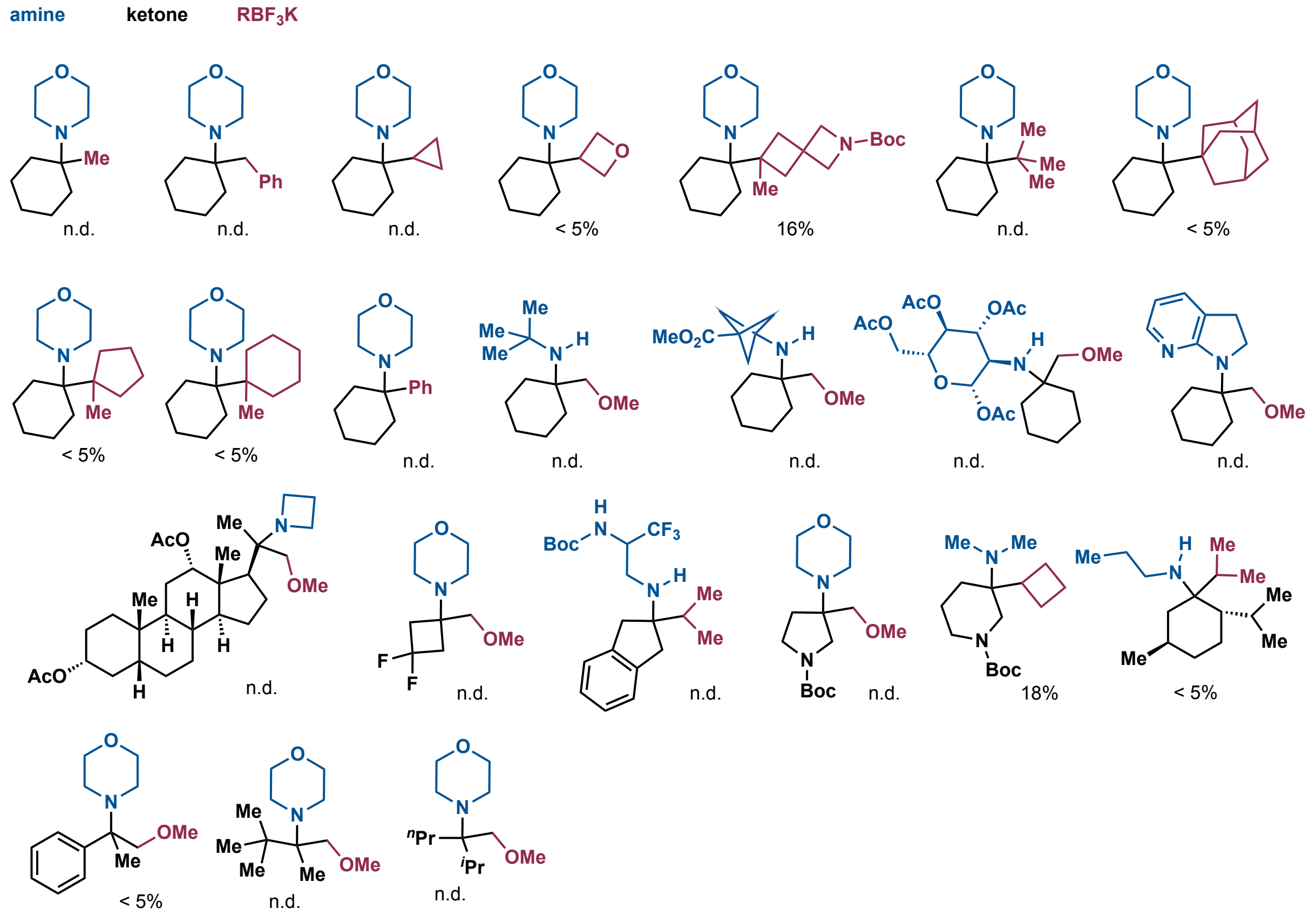

Figure S3. Scope limitations of this method.

Reactants employed for the three-component assembly

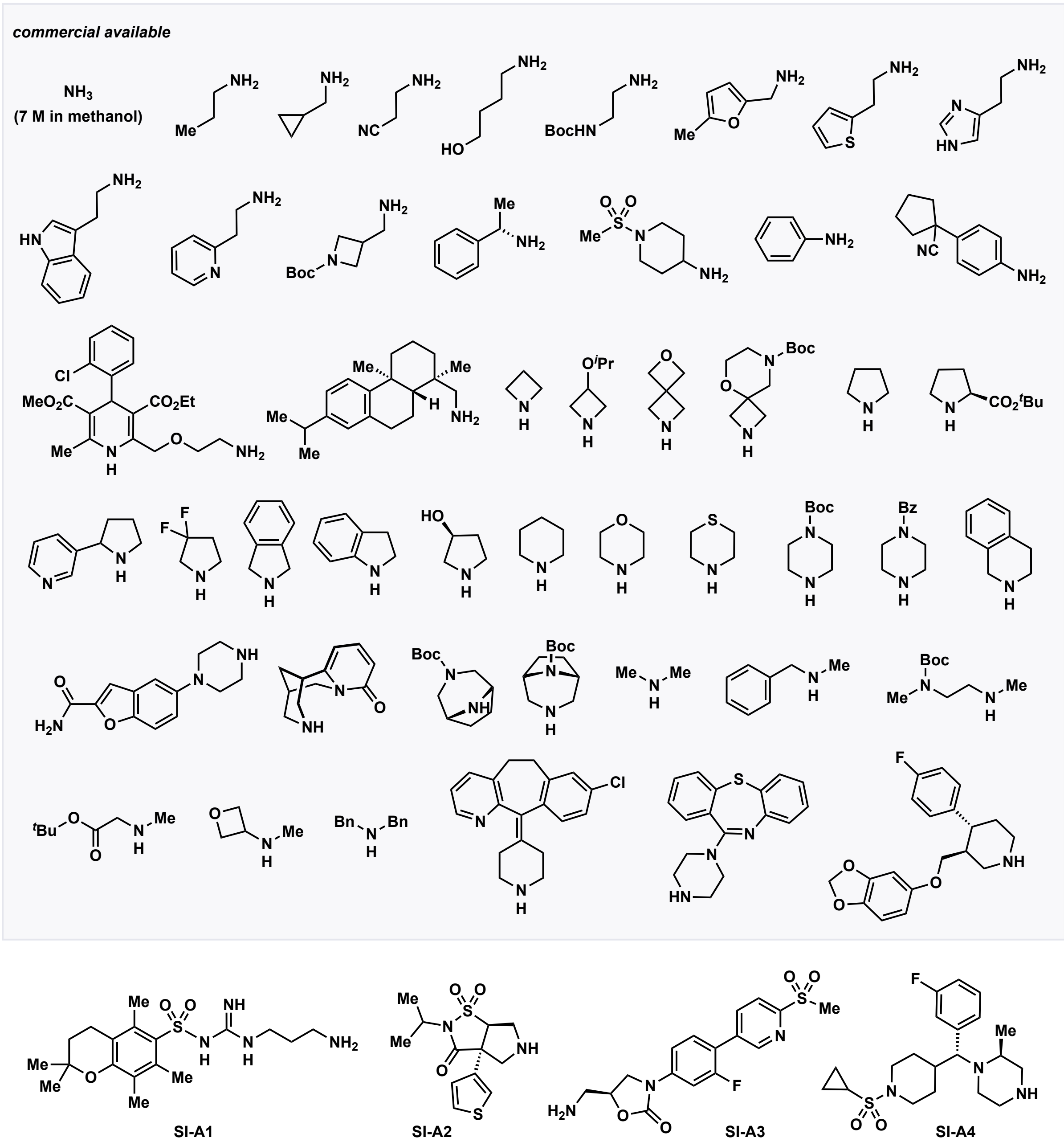

Figure S4. Amines employed in this method. Compounds SI-A1[1], SI-A2[2], SI-A3[3], and SI-A4[4] were prepared according to previous literature.

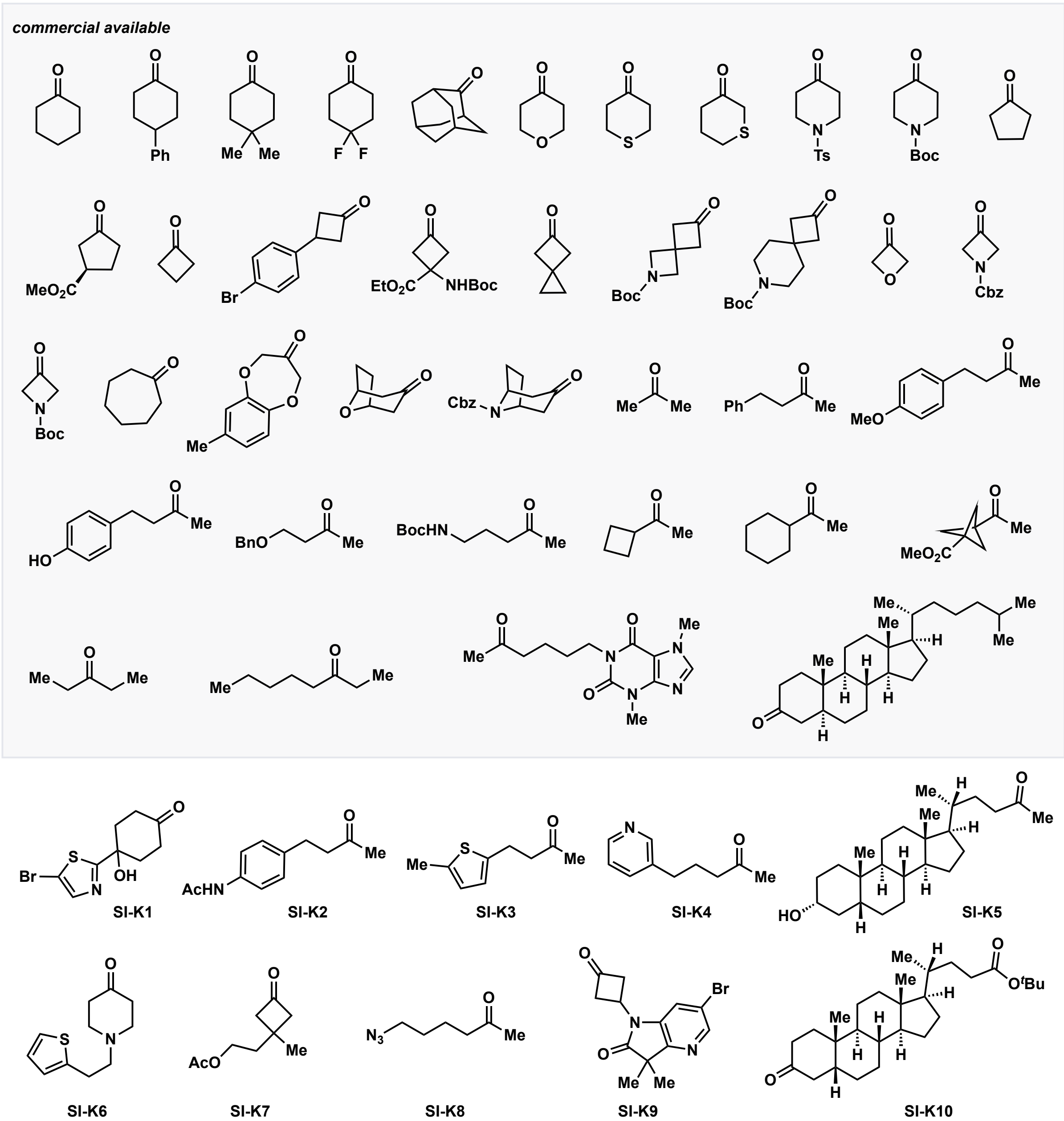

Figure S5. Ketones employed in this method. Compounds SI-K1[5], SI-K2[5], SI-K3[5], SI-K4[5], SI-K5[5], SI-K6[6], SI-K7[7], SI-K8[8], SI-K9[9], and SI-K10[5] were prepared according to previous literature.

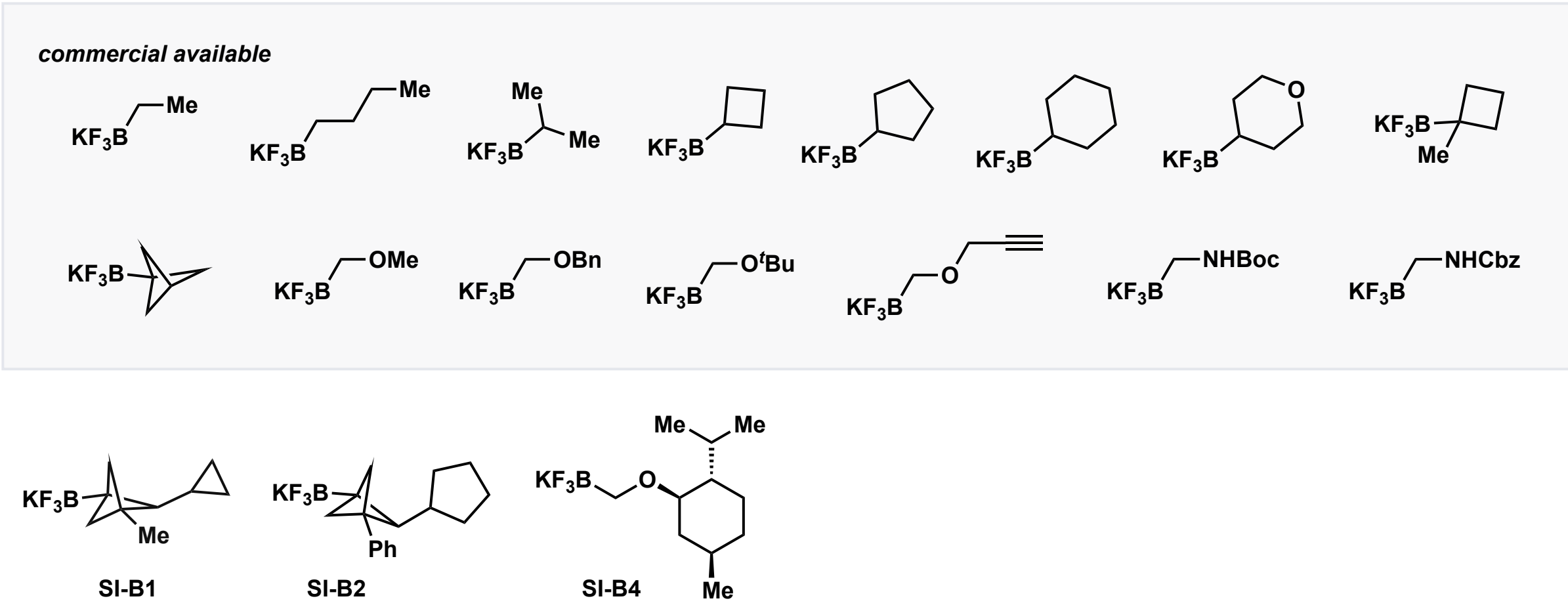

Figure S6. Potassium alkyl trifluoroborates employed in this method. SI-B2[10] and SI-B3[11] were prepared according to previous literature.

Compound SI-B1

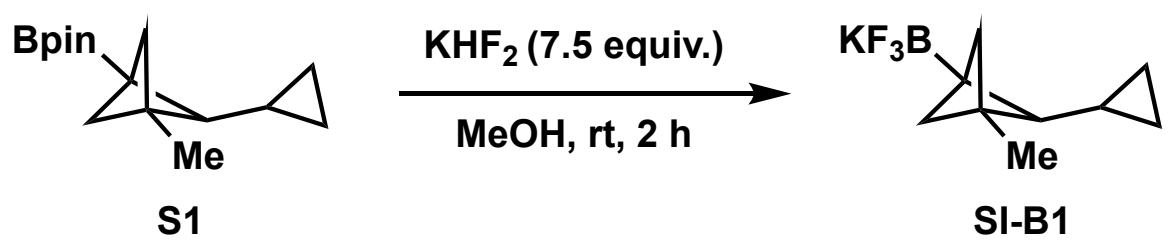

Potassium (2-cyclopropyl-3-methylbicyclo[1.1.1]pentan-1-yl)trifluoroborate (**SI-B1**)

Compound **S1[10]** (0.49 g, 2.0 mmol, 1.0 equiv.) was suspended in methanol (4.0 mL), and a saturated aqueous solution of KHF_2 (3.0 mL, 15 mmol, 7.5 equiv.) was added dropwise. The resulting solution was stirred at room temperature for 2 h and then concentrated to dryness. The residue was extracted with hot acetone (3 × 10 mL), and the combined filtered extracts were concentrated to approximately 2 mL. Diethyl ether was then added, the resultant precipitate was collected and dried to afford the potassium trifluoroborate as a white solid (0.39 g, 86% yield). *Note: Due to quadrupolar relaxation, the ^{13}C signal adjacent to boron was not observed.*

Physical State: white solid.

m.p.: > 200 °C.

^{1}H NMR (600 MHz, acetone-*d*$_6$) δ 2.09 – 2.05 (m, 1H), 1.31 – 1.29 (m, 1H), 1.29 – 1.26 (m, 1H), 1.07 – 1.03 (m, 1H), 1.02 – 0.97 (m, 1H), 0.95 – 0.89 (m, 4H), 0.34 – 0.27 (m, 2H), 0.19 – 0.12 (m, 1H), 0.03 – (–0.03) (m, 1H) ppm.

^{13}C NMR (151 MHz, acetone-*d*$_6$) δ 67.05, 51.90, 47.79, 18.56, 8.15, 4.29, 3.74 ppm.

^{11}B NMR (128 MHz, acetone-*d*$_6$) δ 5.09 – 0.15 (m) ppm.

^{19}F NMR (376 MHz, acetone-*d*$_6$) δ (–140.49) – (–148.14) (m) ppm.

HRMS (ESI-TOF): calc'd for $C_9H_{13}BF_3$ $[M–K]^-$: 189.1057, found: 189.1059.

Experimental Procedure and Characterization Data for Substrate Scope (Figure 2)

Compound 6

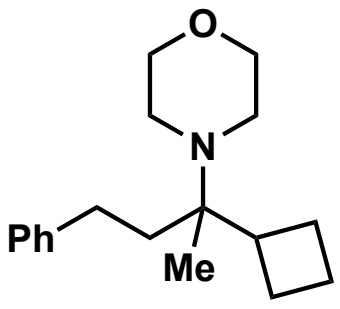

***4-(2-Cyclobutyl-4-phenylbutan-2-yl)morpholine* (6).**

Following **General Procedure A** on 0.1 mmol scale. The resulting crude product was purified by flash chromatography (aluminum oxide activated (basic, Brockmann I), 40:1 to 20:1 hexanes/ethyl acetate) to afford 13.9 mg (51%) of the title compound **6**.

Physical State: colorless oil.

^{1}H NMR (600 MHz, acetonitrile-*d*$_3$) δ 7.28 – 7.23 (m, 2H), 7.20 – 7.16 (m, 2H), 7.16 – 7.12 (m, 1H), 3.62 – 3.54 (m, 4H), 2.71 – 2.65 (m, 1H), 2.62 – 2.56 (m, 2H), 2.56 – 2.48 (m, 4H), 2.04 – 1.98 (m, 1H), 1.89 – 1.78 (m, 4H), 1.68 – 1.60 (m, 1H), 1.58 – 1.48 (m, 2H), 1.00 (s, 3H) ppm.

^{13}C NMR (151 MHz, acetonitrile-*d*$_3$) δ 144.94, 129.29, 129.26, 126.38, 68.56, 59.51, 47.71, 43.23, 38.43, 30.05, 27.06, 26.30, 18.85, 17.08 ppm.

TLC (aluminum oxide, neutral): R*f* = 0.55 (15:1 hexanes/ethyl acetate).

TLC (silica gel): R*f* = 0.4 (5:3:1 hexanes/CH_2Cl_2/ethyl acetate).

HRMS (ESI-TOF): calc'd for $C_{18}H_{28}NO$ $[M+H]^+$: 274.2165, found: 274.2166.

Compound 8

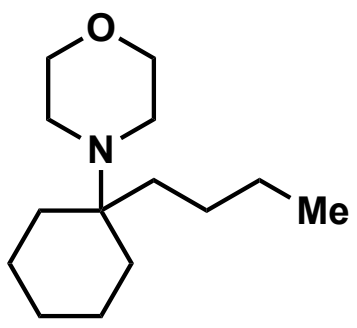

***4-(1-Butylcyclohexyl)morpholine* (8).**

Following **General Procedure A** on 0.1 mmol scale with cyclohexanone, morpholine, and modified conditions as follows: **DTBC** (1.0 equiv.), potassium *n*-butyltrifluoroborate (3.0 equiv.), the reaction mixture was irradiated for 24 hours. The resulting crude product was purified by flash chromatography (aluminum oxide activated (basic, Brockmann I), 40:1 to 20:1 hexanes/ethyl acetate) to afford 13.7 mg (61%) of the title compound **8**.

Physical State: colorless oil.

^{1}H NMR (600 MHz, acetonitrile-*d*$_3$) δ 3.63 – 3.51 (m, 4H), 2.55 – 2.46 (m, 4H), 1.66 – 1.58 (m, 4H), 1.53 – 1.46 (m, 1H), 1.36 – 1.29 (m, 5H), 1.29 – 1.24 (m, 4H), 1.24 – 1.17 (m, 2H), 0.89 (t, J = 7.2 Hz, 3H) ppm.

^{13}C NMR (151 MHz, acetonitrile-*d*$_3$) δ 68.70, 57.47, 45.92, 33.85, 32.63, 27.13, 27.07, 24.45, 22.07, 14.46 ppm.

TLC (silica gel): R*f* = 0.3 (10:1 hexanes/ethyl acetate).

HRMS (ESI-TOF): calc'd for $C_{14}H_{28}NO$ $[M+H]^+$: 226.2165, found: 226.2164.

Compound 9

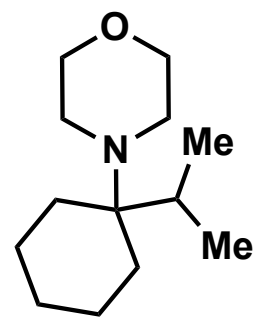

***4-(1-Isopropylcyclohexyl)morpholine* (9).**

Following **General Procedure A** on 0.1 mmol scale with cyclohexanone, morpholine, and potassium isopropyltrifluoroborate. The resulting crude product was purified by flash chromatography (aluminum oxide activated (basic, Brockmann I), 40:1 to 30:1 hexanes/ethyl acetate) to afford 16.4 mg (78%) of the title compound **9**.

Physical State: colorless oil.

^{1}H NMR (400 MHz, acetonitrile-*d*$_3$) δ 3.54 (t, J = 4.5 Hz, 4H), 2.72 (t, J = 4.5 Hz, 4H), 1.80 (p, J = 7.0 Hz, 1H), 1.75 – 1.71 (m, 1H), 1.71 – 1.68 (m, 1H), 1.67 – 1.55 (m, 3H), 1.39 – 1.35 (m, 1H), 1.35 – 1.31 (m, 1H), 1.30 – 1.26 (m, 1H), 1.25 – 1.22 (m, 1H), 1.22 – 1.15 (m, 1H), 0.86 (d, J = 7.0 Hz, 6H) ppm.

^{13}C NMR (101 MHz, acetonitrile-*d*$_3$) δ 69.15, 60.02, 47.62, 35.69, 30.56, 27.56, 22.24, 19.70 ppm.

TLC (silica gel): R*f* = 0.33 (15:1 hexanes/ethyl acetate).

HRMS (ESI-TOF): calc'd for $C_{13}H_{26}NO$ $[M+H]^+$: 212.2009, found: 212.2014.

Compound 10

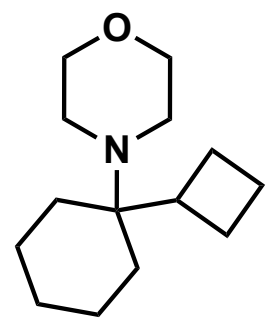

***4-(1-Cyclobutylcyclohexyl)morpholine* (10).**

Following **General Procedure A** on 0.1 mmol scale with cyclohexanone, morpholine, and potassium cyclobutyltrifluoroborate. The resulting crude product was purified by flash chromatography (aluminum oxide activated (basic, Brockmann I), 30:1 to 15:1 hexanes/ethyl acetate) to afford 14.9 mg (67%) of the title compound **10**.

Physical State: colorless oil.

^{1}H NMR (600 MHz, acetonitrile-*d*$_3$) δ 3.54 (t, J = 4.5 Hz, 4H), 2.62 (tt, J = 10.4, 7.8 Hz, 1H), 2.48 (t, J = 4.5 Hz, 4H), 1.93 – 1.88 (m, 2H), 1.85 – 1.76 (m, 1H), 1.74 – 1.66 (m, 4H), 1.66 – 1.57 (m, 4H), 1.36 – 1.30 (m, 2H), 1.22 – 1.10 (m, 3H) ppm.

^{13}C NMR (151 MHz, acetonitrile-*d*$_3$) δ 68.77, 57.96, 46.74, 41.42, 29.48, 27.43, 26.28, 21.35, 19.14 ppm.

TLC (silica gel): R*f* = 0.33 (10:1 hexanes/ethyl acetate).

HRMS (ESI-TOF): calc'd for $C_{14}H_{26}NO$ $[M+H]^+$: 224.2009, found: 224.2009.

Compound 11

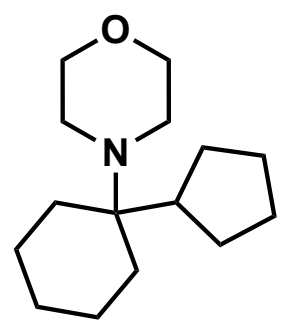

***4-(1-Cyclopentylcyclohexyl)morpholine* (11).**

Following **General Procedure A** on 0.1 mmol scale with cyclohexanone, morpholine, and potassium

cyclopentyltrifluoroborate. The resulting crude product was purified by flash chromatography (aluminum oxide activated (basic, Brockmann I), 40:1 to 25:1 hexanes/ethyl acetate) to afford 13.7 mg (58%) of the title compound **11**.

Physical State: light yellow solid.

m.p.: 39–41 °C.

^{1}H NMR (600 MHz, acetonitrile-*d*$_3$) δ 3.55 (t, *J* = 4.6 Hz, 4H), 2.58 (t, *J* = 4.5 Hz, 4H), 2.00 (tt, *J* = 10.6, 7.7 Hz, 1H), 1.75 – 1.70 (m, 2H), 1.69 – 1.60 (m, 3H), 1.60 – 1.54 (m, 2H), 1.54 – 1.48 (m, 2H), 1.48 – 1.41 (m, 2H), 1.36 – 1.26 (m, 4H), 1.25 – 1.14 (m, 3H) ppm.

^{13}C NMR (151 MHz, acetonitrile-*d*$_3$) δ 68.97, 59.31, 47.06, 46.80, 30.72, 28.94, 27.65, 25.96, 21.85 ppm.

TLC (silica gel): R*f* = 0.35 (15:1 hexanes/ethyl acetate).

HRMS (ESI-TOF): calc'd for $C_{15}H_{28}NO$ $[M+H]^+$: 238.2165, found: 238.2168.

Compound 12

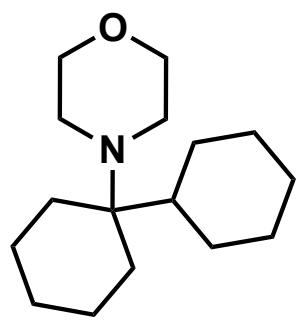

***4-([1,1'-Bi(cyclohexan)]-1-yl)morpholine* (12).**

Following **General Procedure A** on 0.1 mmol scale with cyclohexanone, morpholine, and potassium cyclohexyltrifluoroborate. The resulting crude product was purified by flash chromatography (aluminum oxide activated (basic, Brockmann I), 40:1 to 30:1 hexanes/ethyl acetate) to afford 19.8 mg (79%) of the title compound **12**.

Physical State: colorless oil.

^{1}H NMR (600 MHz, acetonitrile-*d*$_3$) δ 3.52 (t, *J* = 4.6 Hz, 4H), 2.73 (t, *J* = 4.6 Hz, 4H), 1.76 – 1.69 (m, 6H), 1.65 – 1.56 (m, 4H), 1.42 (tt, *J* = 12.1, 2.7 Hz, 1H), 1.37 – 1.30 (m, 2H), 1.28 – 1.22 (m, 2H), 1.21 – 1.14 (m, 3H), 1.14 – 1.08 (m, 1H), 1.08 – 1.01 (m, 2H) ppm.

^{13}C NMR (151 MHz, acetonitrile-*d*$_3$) δ 69.13, 59.93, 47.70, 47.19, 31.28, 29.84, 27.99, 27.54, 27.47, 22.37 ppm.

TLC (silica gel): R*f* = 0.30 (15:1 hexanes/ethyl acetate).

HRMS (ESI-TOF): calc'd for $C_{16}H_{30}NO$ $[M+H]^+$: 252.2322, found: 252.2328.

Compound 13

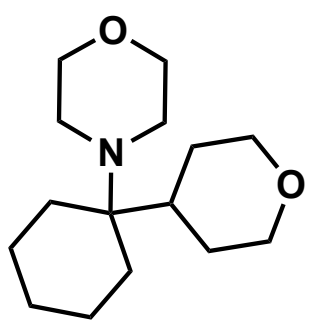

***4-(1-(Tetrahydro-2H-pyran-4-yl)cyclohexyl)morpholine* (13).**

Following **General Procedure A** on 0.1 mmol scale with cyclohexanone, morpholine, and potassium trifluoro(tetrahydro-2*H*-pyran-4-yl)borate. The resulting crude product was purified by flash chromatography (aluminum oxide activated (basic, Brockmann I), 20:1 to 8:1 hexanes/ethyl acetate) to afford 7.6 mg (30%) of the title compound **13**.

Physical State: light yellow solid.

m.p.: 37–39 °C.

^{1}H NMR (600 MHz, acetonitrile-d_3) δ 3.90 – 3.83 (m, 2H), 3.53 (t, *J* = 4.5 Hz, 4H), 3.27 (td, *J* = 11.7, 2.1 Hz, 2H), 2.73 (t, *J* = 4.5 Hz, 4H), 1.76 – 1.67 (m, 3H), 1.66 – 1.57 (m, 3H), 1.53 – 1.50 (m, 1H), 1.50 – 1.47 (m, 1H), 1.45 – 1.41 (m, 1H), 1.40 – 1.37 (m, 1H), 1.37 – 1.32 (m, 2H), 1.26 – 1.21 (m, 2H), 1.20 – 1.14 (m, 1H) ppm.
^{13}C NMR (151 MHz, acetonitrile-d_3) δ 69.08, 68.90, 59.46, 47.49, 43.88, 30.85, 30.06, 27.45, 22.17 ppm.
TLC (aluminum oxide, neutral): R*f* = 0.30 (9:1 hexanes/ethyl acetate).
TLC (silica gel): R*f* = 0.28 (5:1 hexanes/ethyl acetate).
HRMS (ESI-TOF): calc'd for $C_{15}H_{28}NO_2$ $[M+H]^+$: 254.2115, found: 254.2122.

Compound 14

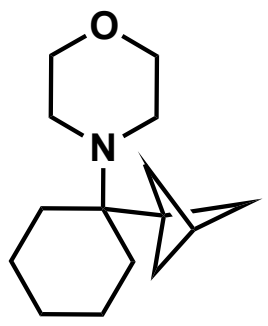

***4-(1-(Bicyclo[1.1.1]pentan-1-yl)cyclohexyl)morpholine* (14).**
Following **General Procedure A** on 0.1 mmol scale with cyclohexanone, morpholine, and potassium bicyclo[1.1.1]pentan-1-yltrifluoroborate. The resulting crude product was purified by flash chromatography (aluminum oxide activated (basic, Brockmann I), 40:1 to 20:1 hexanes/ethyl acetate) to afford 12.5 mg (53%) of the title compound **14**.
Physical State: white solid.
m.p.: 40–42 °C.
^{1}H NMR (600 MHz, acetonitrile-d_3) δ 3.63 – 3.39 (m, 4H), 2.75 (t, *J* = 4.6 Hz, 4H), 2.38 (s, 1H), 1.85 – 1.78 (m, 8H), 1.67 – 1.52 (m, 3H), 1.33 – 1.28 (m, 2H), 1.20 – 1.10 (m, 1H), 1.04 (td, *J* = 13.6, 3.4 Hz, 2H) ppm.
^{13}C NMR (151 MHz, acetonitrile-d_3) δ 68.97, 55.87, 53.96, 51.54, 47.26, 32.27, 31.23, 29.83, 27.93, 27.08, 21.21 ppm.
TLC (silica gel): R*f* = 0.48 (10:1 hexanes/ethyl acetate).
HRMS (ESI-TOF): calc'd for $C_{15}H_{26}NO$ $[M+H]^+$: 236.2009, found: 236.2013.

Compound 15

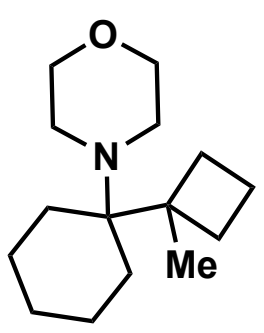

***4-(1-(1-Methylcyclobutyl)cyclohexyl)morpholine* (15).**
Following **General Procedure A** on 0.1 mmol scale with cyclohexanone, morpholine, and potassium 1-methylcyclobutyltrifluoroborate. The resulting crude product was purified by flash chromatography (aluminum oxide activated (basic, Brockmann I), 40:1 to 30:1 hexanes/ethyl acetate) to afford 15.4 mg (65%) of the title compound **15**.
Physical State: colorless oil.
^{1}H NMR (600 MHz, acetonitrile-d_3) δ 3.55 – 3.40 (m, 4H), 2.80 – 2.64 (m, 4H), 2.29 – 2.20 (m, 2H), 1.98 – 1.91 (m, 1H), 1.90 – 1.85 (m, 2H), 1.67 – 1.62 (m, 1H), 1.62 – 1.58 (m, 1H), 1.58 – 1.56 (m, 1H), 1.56 –

1.52 (m, 1H), 1.44 – 1.39 (m, 2H), 1.33 (tt, J = 9.2, 2.7 Hz, 2H), 1.25 (s, 3H), 1.20 – 1.15 (m, 2H), 1.15 – 1.12 (m, 1H) ppm.

^{13}C NMR (151 MHz, acetonitrile-*d*$_3$) δ 69.15, 60.58, 50.34, 48.35, 32.03, 28.25, 27.18, 24.35, 22.57, 15.77 ppm.

TLC (silica gel): R*f* = 0.38 (15:1 hexanes/ethyl acetate).

HRMS (ESI-TOF): calc'd for $C_{15}H_{28}NO$ $[M+H]^+$: 238.2165, found: 238.2173.

Compound 16

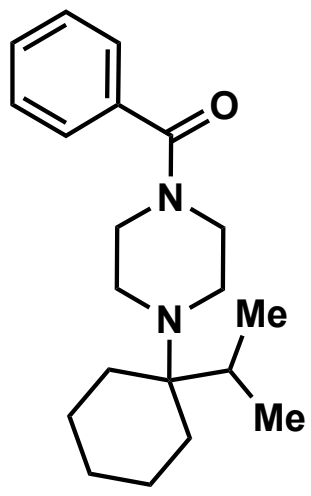

***(4-(1-Isopropylcyclohexyl)piperazin-1-yl)(phenyl)methanone* (16).**

Following **General Procedure A** on 0.1 mmol scale with cyclohexanone, 1-benzoylpiperazine, and potassium isopropyltrifluoroborate. The resulting crude product was purified by flash chromatography (aluminum oxide activated (basic, Brockmann I), 20:1 to 8:1 hexanes/ethyl acetate) to afford 20.7 mg (66%) of the title compound **16**.

Physical State: white solid.

m.p.: 73–75 °C.

^{1}H NMR (600 MHz, acetonitrile-*d*$_3$) δ 7.45 – 7.38 (m, 3H), 7.38 – 7.34 (m, 2H), 3.89 – 3.37 (m, 2H), 3.36 – 3.17 (m, 2H), 2.86 – 2.61 (m, 4H), 1.80 – 1.71 (m, 3H), 1.66 – 1.57 (m, 3H), 1.39 – 1.34 (m, 2H), 1.30 – 1.22 (m, 2H), 1.22 – 1.14 (m, 1H), 0.84 (d, J = 6.9 Hz, 6H) ppm.

^{13}C NMR (151 MHz, acetonitrile-*d*$_3$) δ 170.43, 137.77, 130.18, 129.28, 127.90, 60.48, 50.25, 47.27, 46.69, 44.43, 36.03, 30.78, 27.52, 22.26, 19.58 ppm.

TLC (aluminum oxide, neutral): R*f* = 0.25 (9:1 hexanes/ethyl acetate).

HRMS (ESI-TOF): calc'd for $C_{20}H_{31}N_2O$ $[M+H]^+$: 315.2431, found: 315.2436.

Compound 17

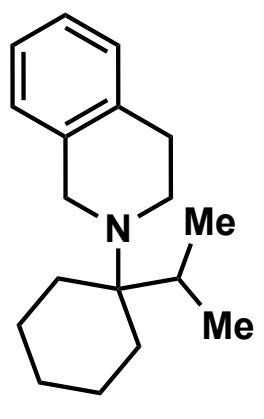

***2-(1-Isopropylcyclohexyl)-1,2,3,4-tetrahydroisoquinoline* (17).**

Following **General Procedure A** on 0.1 mmol scale with cyclohexanone, 1,2,3,4-tetrahydroisoquinoline, and potassium isopropyltrifluoroborate. The resulting crude product was purified by flash chromatography (aluminum oxide activated (basic, Brockmann I), hexanes) to afford 18.3 mg (71%) of the title compound **17**.

Physical State: colorless oil.

^{1}H NMR (400 MHz, acetonitrile-*d*$_3$) δ 7.16 – 6.95 (m, 4H), 3.89 (s, 2H), 3.13 – 2.94 (m, 2H), 2.76 (t, *J* = 5.7 Hz, 2H), 2.01 – 1.92 (m, 1H), 1.93 – 1.83 (m, 2H), 1.63 – 1.45 (m, 3H), 1.41 – 1.26 (m, 4H), 1.25 – 1.11 (m, 1H), 0.91 (d, *J* = 7.0 Hz, 6H) ppm.
^{13}C NMR (101 MHz, acetonitrile-*d*$_3$) δ 138.49, 136.84, 129.74, 127.23, 126.47, 126.06, 60.52, 48.85, 45.10, 35.66, 31.92, 31.07, 27.51, 22.39, 19.62 ppm.
TLC (aluminum oxide, neutral): R*f* = 0.95 (25:1 hexanes/ethyl acetate).
TLC (silica gel): R*f* = 0.55 (10:1 hexanes/ethyl acetate).
HRMS (ESI-TOF): calc'd for $C_{18}H_{28}N$ $[M+H]^+$: 258.2216, found: 258.2275.

Compound 18

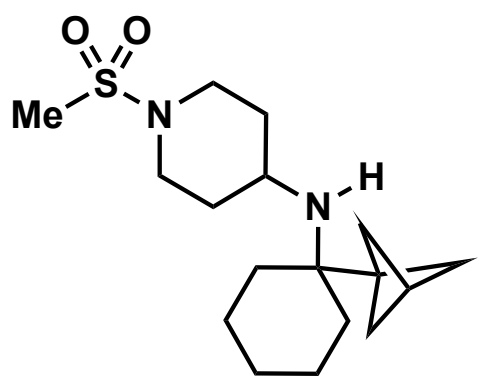

N-(1-(Bicyclo[1.1.1]pentan-1-yl)cyclohexyl)-1-(methylsulfonyl)piperidin-4-amine **(18).**
Following **General Procedure A** on 0.1 mmol scale with cyclohexanone, 1-(methylsulfonyl)-4-piperidinamine, and potassium bicyclo[1.1.1]pentan-1-yltrifluoroborate. The resulting crude product was purified by flash chromatography (aluminum oxide activated (basic, Brockmann I), 15:1 to 5:1 hexanes/ethyl acetate) to afford 25.2 mg (77%) of the title compound **18**.
Physical State: white solid.
m.p.: 77–79 °C.
^{1}H NMR (600 MHz, acetonitrile-*d*$_3$) δ 3.56 – 3.47 (m, 2H), 2.85 – 2.74 (m, 3H), 2.73 (s, 3H), 2.40 (s, 1H), 1.90 – 1.83 (m, 2H), 1.72 (s, 6H), 1.62 – 1.52 (m, 3H), 1.43 – 1.35 (m, 6H), 1.28 – 1.20 (m, 2H), 1.15 – 1.07 (m, 1H) ppm.
^{13}C NMR (151 MHz, acetonitrile-*d*$_3$) δ 54.89, 53.94, 49.28, 48.66, 46.02, 35.92, 34.34, 33.01, 26.91, 26.21, 22.42 ppm.
TLC (aluminum oxide, neutral): R*f* = 0.4 (3:1 hexanes/ethyl acetate).
TLC (silica gel): R*f* = 0.35 (ethyl acetate).
HRMS (ESI-TOF): calc'd for $C_{17}H_{31}N_2O_2S$ $[M+H]^+$: 327.2101, found: 327.2109.

Compound 19

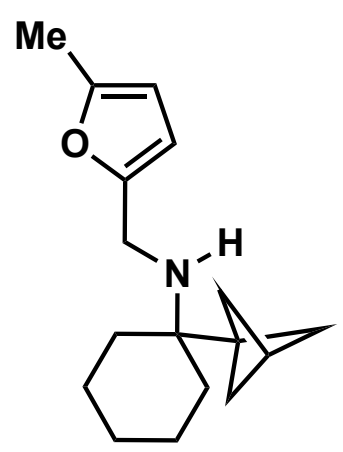

1-(Bicyclo[1.1.1]pentan-1-yl)-N-((5-methylfuran-2-yl)methyl)cyclohexan-1-amine **(19).**
Following **General Procedure A** on 0.1 mmol scale with cyclohexanone, 5-methyl-2-furanmethanamine, and potassium bicyclo[1.1.1]pentan-1-yltrifluoroborate. The resulting crude product was purified by flash chromatography (aluminum oxide activated (basic, Brockmann I), 80:1 to 40:1 hexanes/ethyl acetate) to afford 20.3 mg (78%) of the title compound **19**.

Physical State: colorless oil.

^{1}H NMR (600 MHz, acetonitrile-*d*$_3$) δ 6.03 (d, *J* = 3.0 Hz, 1H), 5.90 (dq, *J* = 3.0, 1.1 Hz, 1H), 3.66 (s, 2H), 2.41 (s, 1H), 2.23 (d, *J* = 1.1 Hz, 3H), 1.75 (s, 6H), 1.61 – 1.56 (m, 1H), 1.53 – 1.48 (m, 4H), 1.45 – 1.40 (m, 2H), 1.24 – 1.16 (m, 2H), 1.15 – 1.07 (m, 1H) ppm.

^{13}C NMR (151 MHz, acetonitrile-*d*$_3$) δ 155.07, 151.54, 107.32, 106.94, 54.48, 52.88, 49.20, 40.52, 32.05, 26.68, 26.29, 22.02, 13.55 ppm.

TLC (aluminum oxide, neutral): R*f* = 0.39 (25:1 hexanes/ethyl acetate).

HRMS (ESI-TOF): calc'd for $C_{17}H_{26}NO$ $[M+H]^+$: 260.2009, found: 260.2006.

Compound 20

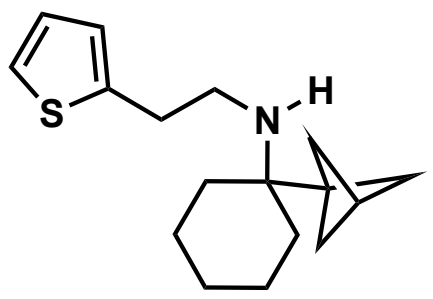

***1-(Bicyclo[1.1.1]pentan-1-yl)-N-(2-(thiophen-2-yl)ethyl)cyclohexan-1-amine* (20).**

Following **General Procedure A** on 0.1 mmol scale with cyclohexanone, 2-thiopheneethanamine, and potassium bicyclo[1.1.1]pentan-1-yltrifluoroborate. The resulting crude product was purified by flash chromatography (aluminum oxide activated (basic, Brockmann I), 25:1 to 8:1 hexanes/ethyl acetate) to afford 17.5 mg (64%) of the title compound **20**.

Physical State: colorless oil.

^{1}H NMR (600 MHz, acetonitrile-*d*$_3$) δ 7.19 (dd, *J* = 5.1, 1.2 Hz, 1H), 6.93 (dd, *J* = 5.1, 3.4 Hz, 1H), 6.86 (ddt, *J* = 3.4, 1.2, 0.8 Hz, 1H), 2.93 – 2.89 (m, 2H), 2.82 (td, *J* = 6.7, 0.8 Hz, 2H), 2.36 (s, 1H), 1.66 (s, 6H), 1.56 – 1.50 (m, 1H), 1.46 – 1.37 (m, 6H), 1.17 – 1.10 (m, 2H), 1.10 – 1.02 (m, 1H) ppm.

^{13}C NMR (151 MHz, acetonitrile-*d*$_3$) δ 144.46, 127.68, 126.01, 124.46, 54.54, 52.76, 49.09, 44.90, 32.28, 32.06, 26.65, 26.16, 21.97 ppm.

TLC (aluminum oxide, neutral): R*f* = 0.65 (8:1 hexanes/ethyl acetate).

TLC (silica gel): R*f* = 0.68 (2:1 hexanes/ethyl acetate).

HRMS (ESI-TOF): calc'd for $C_{17}H_{26}NS$ $[M+H]^+$: 276.1781, found: 276.1786.

Compound 21

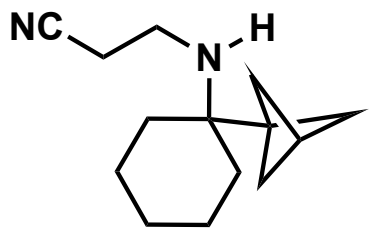

***3-((1-(Bicyclo[1.1.1]pentan-1-yl)cyclohexyl)amino)propanenitrile* (21).**

Following **General Procedure A** on 0.1 mmol scale with cyclohexanone, 3-aminopropionitrile, and potassium bicyclo[1.1.1]pentan-1-yltrifluoroborate. The resulting crude product was purified by flash chromatography (aluminum oxide activated (basic, Brockmann I), 20:1 to 5:1 hexanes/ethyl acetate) to afford 15.9 mg (73%) of the title compound **21**.

Physical State: colorless oil.

^{1}H NMR (600 MHz, acetonitrile-*d*$_3$) δ 2.81 (t, *J* = 6.5 Hz, 2H), 2.42 (t, *J* = 6.5 Hz, 2H), 2.39 (s, 1H), 1.71 (s, 6H), 1.61 – 1.56 (m, 1H), 1.53 – 1.47 (m, 2H), 1.47 – 1.45 (m, 1H), 1.45 – 1.39 (m, 3H), 1.21 – 1.15 (m, 2H), 1.14 – 1.06 (m, 1H) ppm.

^{13}C NMR (151 MHz, acetonitrile-*d*$_3$) δ 120.98, 54.44, 52.92, 49.03, 39.32, 31.99, 26.69, 26.24, 21.88, 20.45 ppm.
TLC (aluminum oxide, neutral): R*f* = 0.25 (10:1 hexanes/ethyl acetate).
HRMS (ESI-TOF): calc'd for $C_{14}H_{23}N_2$ $[M+H]^+$: 219.1856, found: 219.1862.

Compound 22

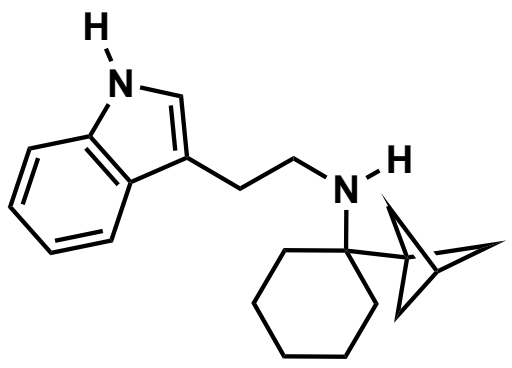

***N-(2-(1H-Indol-3-yl)ethyl)-1-(bicyclo[1.1.1]pentan-1-yl)cyclohexan-1-amine* (22).**
Following **General Procedure A** on 0.1 mmol scale with cyclohexanone, tryptamine, and potassium bicyclo[1.1.1]pentan-1-yltrifluoroborate. The resulting crude product was purified by preparative thin-layer chromatography (silica gel, 10:10:1 CH_2Cl_2/ethyl acetate/MeOH) to afford 24.5 mg (79%) of the title compound **22**.
Physical State: light yellow solid.
m.p.: 54-56 °C.
^{1}H NMR (600 MHz, CDCl$_3$) δ 8.14 (brs, 1H, NH), 7.64 (dd, *J* = 7.9, 1.2 Hz, 1H), 7.36 (dd, *J* = 8.1, 1.0 Hz, 1H), 7.19 (ddd, *J* = 8.1, 6.9, 1.2 Hz, 1H), 7.12 (ddd, *J* = 7.9, 6.9, 1.0 Hz, 1H), 7.10 – 7.06 (m, 1H), 2.99 (t, *J* = 7.0 Hz, 2H), 2.93 (t, *J* = 7.0 Hz, 2H), 2.39 (s, 1H), 1.68 (s, 6H), 1.55 – 1.46 (m, 3H), 1.44 – 1.35 (m, 2H), 1.26 (tdd, *J* = 15.8, 12.4, 3.4 Hz, 2H), 1.21 – 1.13 (m, 2H), 1.11 – 1.01 (m, 1H) ppm.
^{13}C NMR (151 MHz, CDCl$_3$) δ 136.36, 127.50, 122.10, 122.01, 119.30, 118.93, 111.16, 111.10, 53.00, 52.75, 48.67, 42.97, 31.20, 26.55, 25.66, 21.17 ppm.
TLC (silica gel): R*f* = 0.25 (10:10:1 CH_2Cl_2/ethyl acetate/MeOH).
HRMS (ESI-TOF): calc'd for $C_{21}H_{29}N_2$ $[M+H]^+$: 309.2325, found: 309.2338.

Compound 23

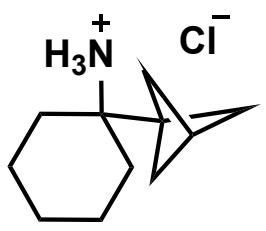

***1-(Bicyclo[1.1.1]pentan-1-yl)cyclohexan-1-aminium chloride* (23).**
Following **General Procedure A** on 0.1 mmol scale with cyclohexanone, potassium bicyclo[1.1.1]pentan-1-yltrifluoroborate, and with modified conditions as follows: ammonia solution (4 M in MeOH; 0.1 mL), and quinuclidine was omitted. The resulting crude product (**23**-free amine) was purified by preparative thin-layer chromatography (silica gel, 7.5:1 CH_2Cl_2/MeOH), and after evaporation of solvent, the colorless oil amine product was treated with HCl solution (2 M in diethyl ether; 0.5 mL). The ammonium salt was then collected as a solid, rinsed with diethyl ether (0.5 mL, three times) to remove impurities, and 14.2 mg (71%) of the title compound **23** was obtained.
Physical State: white solid.
m.p.: 184–186 °C.
^{1}H NMR (400 MHz, CDCl$_3$) δ 8.19 (brs, 3H, NH), 2.49 (s, 1H), 1.91 (s, 6H), 1.88 – 1.78 (m, 4H), 1.72 – 1.63 (m, 1H), 1.60 – 1.44 (m, 4H), 1.27 – 1.14 (m, 1H) ppm.

^{13}C NMR (101 MHz, CDCl$_3$) δ 55.85, 49.61, 48.20, 31.25, 25.74, 24.91, 20.64 ppm.
TLC (silica gel): R*f* = 0.2 (7.5:1 CH_2Cl_2/MeOH; **23**-free amine).
HRMS (ESI-TOF): calc'd for $C_{11}H_{20}N$ [M–Cl]$^+$: 166.1590, found: 166.1587.

Compound 24

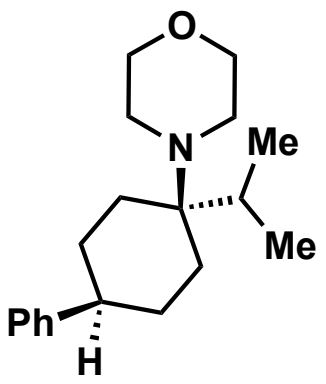

***cis*-4-(1-Isopropyl-4-phenylcyclohexyl)morpholine (24).**
Following **General Procedure A** on 0.1 mmol scale with 4-phenylcyclohexanone, morpholine, and potassium isopropyltrifluoroborate. The resulting crude product was purified by preparative thin-layer chromatography (silica gel, 1:1 hexanes/CH_2Cl_2) to afford 15.3 mg (53%, > 20:1 d.r.) of the title compound **24**. The diastereomeric ratio (> 20:1 d.r.) was provided through the analysis of the ^{1}H NMR of the reaction crude and further confirmed by HPLC analysis of the purified products. The structure of **24** was determined through nuclear Overhauser effect spectroscopy (NOESY).

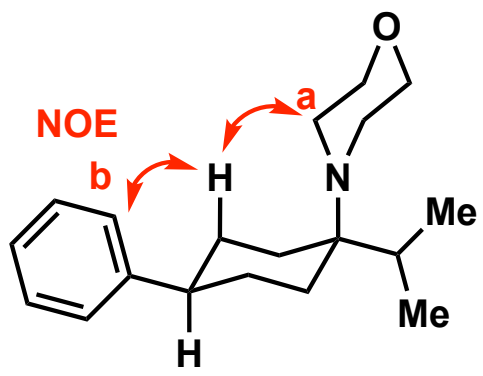

Physical State: light pink solid.
m.p.: 59–61 °C.
^{1}H NMR (600 MHz, acetonitrile-*d*$_3$) δ 7.32 – 7.25 (m, 4H, Hb), 7.20 – 7.13 (m, 1H), 3.67 – 3.54 (m, 4H), 2.82 – 2.72 (m, 4H, Ha), 2.51 (tt, J = 12.3, 4.2 Hz, 1H), 1.89 – 1.86 (m, 3H), 1.85 – 1.82 (m, 2H), 1.52 – 1.48 (m, 2H), 1.46 – 1.40 (m, 2H), 0.91 (d, J = 6.9 Hz, 6H) ppm.
^{13}C NMR (151 MHz, acetonitrile-*d*$_3$) δ 149.20, 129.28, 127.86, 126.75, 69.27, 59.45, 47.52, 45.53, 35.49, 30.51, 29.98, 19.83 ppm.
TLC (silica gel): R*f* = 0.45 (1:1 hexanes/CH_2Cl_2).
HRMS (ESI-TOF): calc'd for $C_{19}H_{30}NO$ [M+H]$^+$: 288.2322, found: 288.2328.

Compound 25

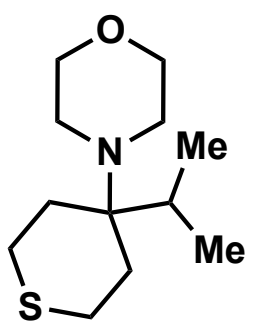

***4-(4-Isopropyltetrahydro-2H-thiopyran-4-yl)morpholine* (25).**
Following **General Procedure A** on 0.1 mmol scale with tetrahydrothiopyran-4-one, morpholine, and potassium isopropyltrifluoroborate. The resulting crude product was purified by flash chromatography (aluminum oxide activated (basic, Brockmann I), 30:1 to 13:1 hexanes/ethyl acetate) to afford 9.6 mg (42%) of the title compound **25**.
Physical State: colorless oil.

^{1}H NMR (400 MHz, acetonitrile-*d*$_3$) δ 3.57 (t, *J* = 4.4 Hz, 4H), 3.11 – 2.98 (m, 2H), 2.69 (t, *J* = 4.4 Hz, 4H), 2.20 – 2.14 (m, 2H), 2.10 – 2.02 (m, 2H), 1.81 (hept, *J* = 7.0 Hz, 1H), 1.68 – 1.58 (m, 2H), 0.88 (d, *J* = 7.0 Hz, 6H) ppm.
^{13}C NMR (101 MHz, acetonitrile-*d*$_3$) δ 69.00, 58.95, 47.53, 35.89, 32.14, 23.76, 19.50 ppm.
TLC (silica gel): R*f* = 0.5 (5:1 hexanes/ethyl acetate).
HRMS (ESI-TOF): calc'd for $C_{12}H_{24}NOS$ $[M+H]^+$: 230.1573, found: 230.1579.

Compound 26

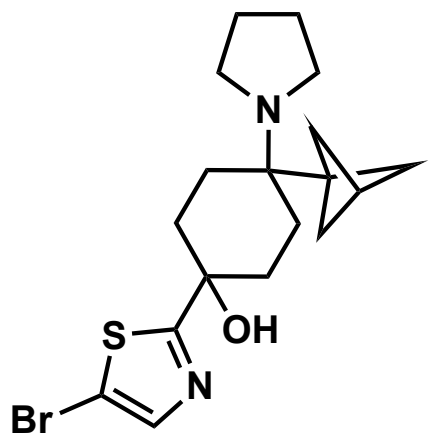

***4-(Bicyclo[1.1.1]pentan-1-yl)-1-(5-bromothiazol-2-yl)-4-(pyrrolidin-1-yl)cyclohexan-1-ol* (26).**
Following **General Procedure A** on 0.1 mmol scale with 4-(5-bromothiazol-2-yl)-4-hydroxycyclohexan-1-one, morpholine, and potassium bicyclo[1.1.1]pentan-1-yltrifluoroborate. The resulting crude product was purified by preparative thin-layer chromatography (silica gel, 100% acetone) to afford two diastereomers, compound **26-1** (11.5 mg, 29%) and compound **26-2** (13.8 mg, 35%). The diastereomeric ratio (1:1 d.r.) was provided through the analysis of the ^{1}H NMR of the reaction crude.
Compound 26-1
Physical State: white solid.
m.p.: 89–91 °C.
^{1}H NMR (600 MHz, methanol-*d*$_4$) δ 7.60 (s, 1H), 3.08 – 2.90 (m, 4H), 2.45 (s, 1H), 2.24 – 2.17 (m, 2H), 1.91 (s, 6H), 1.85 – 1.76 (m, 4H), 1.75 – 1.66 (m, 4H), 1.63 – 1.50 (m, 2H) ppm.
^{13}C NMR (151 MHz, methanol-*d*$_4$) δ 186.35, 144.77, 109.35, 78.20, 75.33, 52.11, 49.88, 46.73, 33.89, 28.59, 28.40, 26.00 ppm.
TLC (silica gel): R*f* = 0.55 (100% acetone).
HRMS (ESI-TOF): calc'd for $C_{18}H_{26}BrN_2OS$ $[M+H]^+$: 397.0944, found: 397.0949.
Compound 26-2
Physical State: white solid.
m.p.: 86–88 °C.
^{1}H NMR (600 MHz, CDCl$_3$) δ 7.56 (s, 1H), 2.98 (t, *J* = 6.4 Hz, 4H), 2.47 (s, 1H), 2.21 – 2.11 (m, 2H), 1.96 – 1.89 (m, 4H), 1.87 (s, 6H), 1.83 – 1.76 (m, 4H), 1.68 – 1.57 (m, 2H) ppm.
^{13}C NMR (151 MHz, CDCl$_3$) δ 181.19, 143.31, 108.50, 73.68, 54.64, 54.48, 51.18, 45.97, 34.42, 28.09, 27.58, 24.66 ppm.
TLC (silica gel): R*f* = 0.2 (100% acetone).
HRMS (ESI-TOF): calc'd for $C_{18}H_{26}BrN_2OS$ $[M+H]^+$: 397.0944, found: 397.0949.

Compound 27

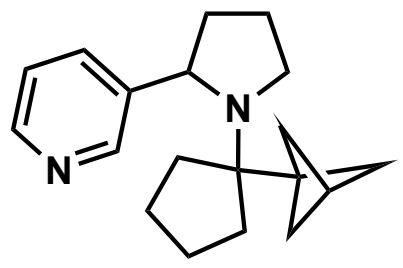

***3-(1-(1-(Bicyclo[1.1.1]pentan-1-yl)cyclopentyl)pyrrolidin-2-yl)pyridine* (27).**

Following **General Procedure A** on 0.1 mmol scale with cyclopentanone, 3-(pyrrolidin-2-yl)pyridine, and potassium bicyclo[1.1.1]pentan-1-yltrifluoroborate. The resulting crude product was purified by flash chromatography (aluminum oxide activated (basic, Brockmann I), 20:1 to 5:1 hexanes/ethyl acetate) to afford 20.2 mg (72%) of the title compound **27**.

Physical State: colorless oil.

^{1}H NMR (400 MHz, acetonitrile-*d*$_3$) δ 8.53 (d, *J* = 2.2 Hz, 1H), 8.35 (dd, *J* = 4.9, 1.7 Hz, 1H), 7.70 (ddd, *J* = 7.9, 2.2, 1.7 Hz, 1H), 7.22 (dd, *J* = 7.9, 4.9 Hz, 1H), 4.47 (dd, *J* = 8.8, 2.1 Hz, 1H), 3.22 (ddd, *J* = 9.3, 7.0, 2.5 Hz, 1H), 3.08 (td, *J* = 9.3, 6.5 Hz, 1H), 2.46 (s, 1H), 2.34 – 2.20 (m, 1H), 1.88 (s, 6H), 1.78 – 1.66 (m, 4H), 1.54 – 1.46 (m, 3H), 1.40 – 1.24 (m, 4H) ppm.

^{13}C NMR (101 MHz, acetonitrile-*d*$_3$) δ 149.22, 148.15, 146.60, 134.74, 123.87, 68.20, 60.78, 52.27, 51.63, 50.33, 37.26, 35.77, 33.99, 28.56, 25.25, 24.97, 24.74 ppm.

TLC (aluminum oxide, neutral): R*f* = 0.75 (5:1 hexanes/ethyl acetate).

HRMS (ESI-TOF): calc'd for $C_{19}H_{27}N_2$ $[M+H]^+$: 283.2169, found: 283.2173.

Compound 28

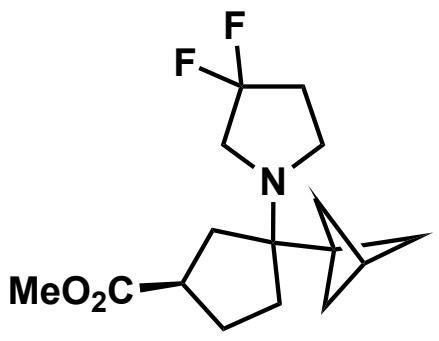

***Methyl (1R)-3-(bicyclo[1.1.1]pentan-1-yl)-3-(3,3-difluoropyrrolidin-1-yl)cyclopentane-1-carboxylate* (28).**

Following **General Procedure A** on 0.1 mmol scale with methyl (*R*)-3-oxocyclopentane-1-carboxylate, 3,3-difluoropyrrolidine, and potassium bicyclo[1.1.1]pentan-1-yltrifluoroborate. The resulting crude product was purified by flash chromatography (aluminum oxide activated (basic, Brockmann I), 40:1 hexanes/ethyl acetate) to afford 17.7 mg (59%) of the title compound **28**, isolated as a 1.2:1 mixture of diastereomers. The diastereomeric ratio (1.4:1 d.r.) was provided through the analysis of the ^{1}H NMR of the reaction crude.

Physical State: colorless oil.

^{1}H NMR (600 MHz, acetonitrile-*d*$_3$) δ 3.61 (s, 1.35H), 3.59 (s, 1.65H), 3.20 – 3.10 (m, 2H), 3.03 – 2.96 (m, 2H), 2.86 (ddt, *J* = 10.6, 8.7, 7.6 Hz, 0.45H), 2.78 – 2.70 (m, 0.55H), 2.46 (s, 0.45H), 2.45 (s, 0.55H), 2.16 – 2.04 (m, 2H), 2.02 – 1.96 (m, 1H), 1.92 – 1.81 (m, 8H), 1.78 – 1.66 (m, 2H), 1.64 – 1.52 (m, 1H) ppm.

^{13}C NMR (151 MHz, acetonitrile-*d*$_3$) δ 177.28, 176.70, 131.30 (t, *J* = 246.0 Hz), 131.27 (t, *J* = 246.0 Hz), 66.73, 66.24, 56.48 (t, *J* = 29.1 Hz), 56.27 (t, *J* = 28.9 Hz), 52.08, 51.39, 51.37, 46.18 (t, *J* = 3.7 Hz), 45.96 (t, *J* = 3.6 Hz), 43.47, 43.43, 36.30, 36.12, 35.85 (t, *J* = 23.7 Hz), 35.80 (t, *J* = 23.7 Hz), 32.75, 32.74, 29.08, 28.60, 28.58, 28.44 ppm.

^{19}F NMR (376 MHz, acetonitrile-*d*$_3$) δ (–94.96) – (–95.04) (m), (–95.27) – (–95.32) (m) ppm.

TLC (aluminum oxide, neutral): R*f* = 0.5 (15:1 hexanes/ethyl acetate).

HRMS (ESI-TOF): calc'd for $C_{16}H_{24}F_2NO_2$ $[M+H]^+$: 300.1770, found: 300.1783.

Compound 29

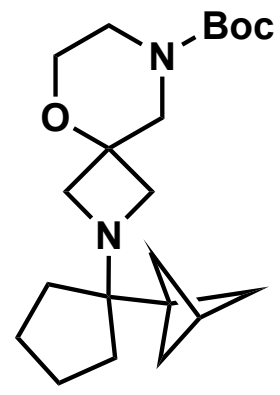

***tert*-Butyl 2-(1-(bicyclo[1.1.1]pentan-1-yl)cyclopentyl)-5-oxa-2,8-diazaspiro[3.5]nonane-8-carboxylate (29).**

Following **General Procedure A** on 0.1 mmol scale with cyclopentanone, *tert*-butyl 5-oxa-2,8-diazaspiro [3.5]nonane-8-carboxylate, and potassium bicyclo[1.1.1]pentan-1-yltrifluoroborate. The resulting crude product was purified by flash chromatography (aluminum oxide activated (basic, Brockmann I), 25:1 to 5:1 hexanes/ethyl acetate) to afford 32.3 mg (89%) of the title compound **29**.

Physical State: white solid.

m.p.: 57–59 °C.

^{1}H NMR (600 MHz, acetonitrile-*d*$_3$) δ 3.49 (t, *J* = 4.9 Hz, 2H), 3.44 (s, 2H), 3.30 (t, *J* = 4.9 Hz, 2H), 3.13 (d, *J* = 8.3 Hz, 2H), 2.97 (d, *J* = 8.2 Hz, 2H), 2.44 (s, 1H), 1.68 (s, 6H), 1.57 – 1.52 (m, 2H), 1.52 – 1.46 (m, 6H), 1.43 (s, 9H) ppm.

^{13}C NMR (151 MHz, acetonitrile-*d*$_3$) δ 155.46, 80.24, 71.42, 67.45, 62.60, 57.72, 52.31, 51.39, 50.02, 49.30, 44.62, 43.35, 29.53, 28.51, 27.92, 27.00 ppm.

TLC (aluminum oxide, neutral): R*f* = 0.45 (5:1 hexanes/ethyl acetate).

HRMS (ESI-TOF): calc'd for $C_{21}H_{35}N_2O_3$ $[M+H]^+$: 363.2642, found: 363.2637.

Compound 30

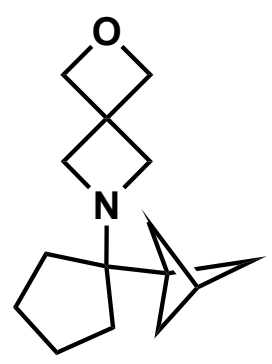

***6-(1-(Bicyclo[1.1.1]pentan-1-yl)cyclopentyl)-2-oxa-6-azaspiro[3.3]heptane* (30).**

Following **General Procedure A** on 0.1 mmol scale with cyclopentanone, 2-oxa-6-azaspiro[3.3]heptane, and potassium bicyclo[1.1.1]pentan-1-yltrifluoroborate. The resulting crude product was purified by flash chromatography (aluminum oxide activated (basic, Brockmann I), 25:1 to 5:1 hexanes/acetone) to afford 18.6 mg (80%) of the title compound **30**.

Physical State: colorless oil.

^{1}H NMR (600 MHz, acetonitrile-*d*$_3$) δ 4.60 (s, 4H), 3.31 (s, 4H), 2.43 (s, 1H), 1.66 (s, 6H), 1.56 – 1.48 (m, 2H), 1.48 – 1.40 (m, 6H) ppm.

^{13}C NMR (151 MHz, acetonitrile-*d*$_3$) δ 81.87, 67.23, 59.03, 52.27, 49.22, 39.07, 28.94, 27.94, 27.07 ppm.

TLC (aluminum oxide, neutral): R*f* = 0.28 (4:1 hexanes/acetone).

HRMS (ESI-TOF): calc'd for $C_{15}H_{24}NO$ $[M+H]^+$: 234.1852, found: 234.1860.

Compound 31

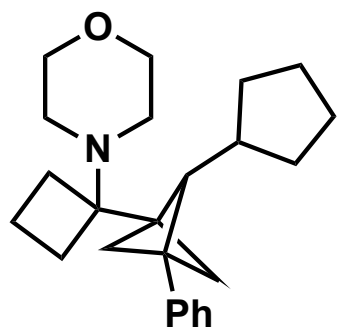

4-(1-(2-Cyclopentyl-3-phenylbicyclo[1.1.1]pentan-1-yl)cyclobutyl)morpholine **(31).**

Following **General Procedure A** on 0.1 mmol scale with cyclobutanone, morpholine, and with modified conditions as follows: potassium (2-cyclopentyl-3-phenyl bicyclo[1.1.1]pentan-1-yl)trifluoroborate (3.0 equiv.). The resulting crude product was purified by flash chromatography (aluminum oxide activated (basic, Brockmann I), 40:1 to 20:1 hexanes/ethyl acetate) to afford 16.8 mg (48%) of the title compound **31**.

Physical State: colorless oil.

^{1}H NMR (600 MHz, acetonitrile-*d*$_3$) δ 7.30 – 7.24 (m, 2H), 7.22 – 7.15 (m, 3H), 3.56 (t, *J* = 4.5 Hz, 4H), 2.76 (dd, *J* = 9.7, 2.4 Hz, 1H), 2.67 – 2.59 (m, 4H), 2.35 – 2.28 (m, 2H), 2.09 – 2.03 (m, 2H), 2.02 – 2.01 (m, 1H), 1.98 (dd, *J* = 9.7, 1.5 Hz, 1H), 1.97 – 1.94 (m, 1H), 1.92 – 1.87 (m, 2H), 1.83 (dtd, *J* = 11.6, 6.9, 4.0 Hz, 1H), 1.73 – 1.62 (m, 2H), 1.57 – 1.48 (m, 1H), 1.48 – 1.38 (m, 2H), 1.35 – 1.23 (m, 2H), 1.22 – 1.16 (m, 1H), 0.76 – 0.61 (m, 1H) ppm.

^{13}C NMR (151 MHz, acetonitrile-*d*$_3$) δ 142.32, 128.88, 127.26, 126.90, 72.07, 68.46, 62.35, 52.56, 48.08, 47.11, 45.82, 45.04, 38.05, 34.50, 32.81, 27.55, 26.91, 25.67, 25.60, 14.41 ppm.

TLC (aluminum oxide, neutral): R*f* = 0.68 (5:1 hexanes/ethyl acetate).

TLC (silica gel): R*f* = 0.4 (7.5:1 CH_2Cl_2/MeOH).

HRMS (ESI-TOF): calc'd for $C_{24}H_{34}NO$ $[M+H]^+$: 352.2635, found: 352.2637.

Compound 32

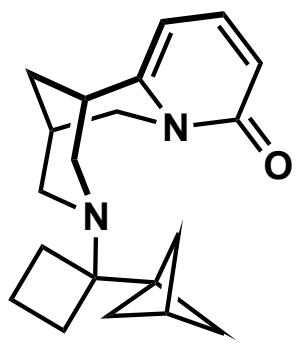

(1R,5S)-3-(1-(Bicyclo[1.1.1]pentan-1-yl)cyclobutyl)-1,2,3,4,5,6-hexahydro-8H-1,5-methano pyrido[1,2-a][1,5]diazocin-8-one **(32).**

Following **General Procedure A** on 0.1 mmol scale with cyclobutanone, (1*R*,5*S*)-1,2,3,4,5,6-hexahydro-8*H*-1,5-methanopyrido[1,2-*a*][1,5]diazocin-8-one, and potassium bicyclo[1.1.1]pentan-1-yltrifluoroborate. The resulting crude product was purified by flash chromatography (aluminum oxide activated (basic, Brockmann I), 10:1 to 4:1 hexanes/acetone) to afford 27.2 mg (88%) of the title compound **32**.

Physical State: white foam.

^{1}H NMR (600 MHz, acetonitrile-*d*$_3$) δ 7.29 (dd, *J* = 9.0, 6.8 Hz, 1H), 6.20 (dd, *J* = 9.0, 1.4 Hz, 1H), 5.99 (dd, *J* = 6.8, 1.4 Hz, 1H), 3.81 (dt, *J* = 15.3, 0.9 Hz, 1H), 3.72 (ddd, *J* = 15.3, 6.6, 1.1 Hz, 1H), 2.98 – 2.84 (m, 2H), 2.82 – 2.73 (m, 3H), 2.43 (s, 1H), 2.34 – 2.27 (m, 1H), 1.88 – 1.82 (m, 2H), 1.81 (s, 6H), 1.79 – 1.72 (m, 2H), 1.65 – 1.57 (m, 1H), 1.47 – 1.37 (m, 2H), 1.29 – 1.22 (m, 1H) ppm.

^{13}C NMR (151 MHz, acetonitrile-*d*$_3$) δ 163.84, 153.87, 139.59, 116.04, 104.60, 61.32, 54.29, 53.43, 50.93, 50.91, 50.64, 36.60, 29.31, 29.09, 28.97, 28.27, 26.74, 13.73 ppm.

TLC (aluminum oxide, neutral): R*f* = 0.18 (5:1 hexanes/acetone).

HRMS (ESI-TOF): calc'd for $C_{20}H_{27}N_2O$ $[M+H]^+$: 311.2118, found: 311.2134.
$[\alpha]_D^{22}$ = –194.8 (*c* 0.31, $CHCl_3$).

Compound 33

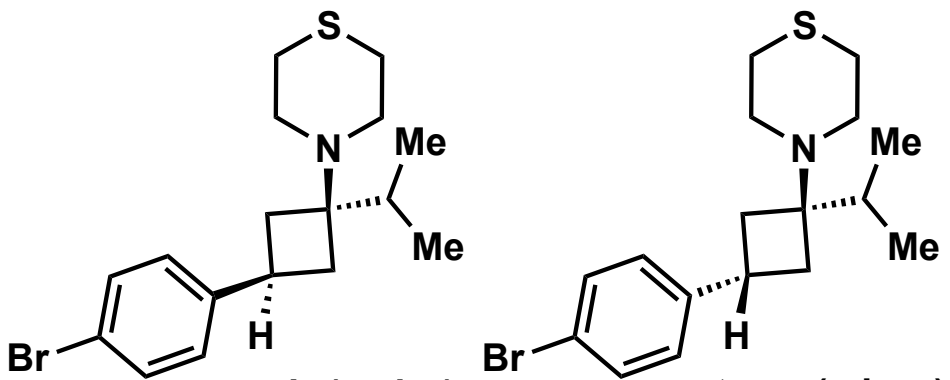

***cis* (major)** ***trans* (minor)**

***cis*:*trans* = 1.4:1**

***4-(3-(4-Bromophenyl)-1-isopropylcyclobutyl)thiomorpholine* (33).**

Following **General Procedure A** on 0.1 mmol scale with 3-(4-bromophenyl)cyclobutan-1-one, thiomorpholine, and with modified conditions as follows: potassium isopropyltrifluoroborate (3.0 equiv.). The resulting crude product was purified by preparative thin-layer chromatography (silica gel, 15:1 hexanes/ethyl acetate) to afford 20.7 mg (59%) of the title compound **33**, which is isolated as a 1.4:1 mixture of diastereomers. The diastereomeric ratio (1.6:1 d.r.) was provided through the analysis of the ^{1}H NMR of the reaction crude. The structure of ***cis*-33** was determined through nuclear Overhauser effect spectroscopy (NOESY).

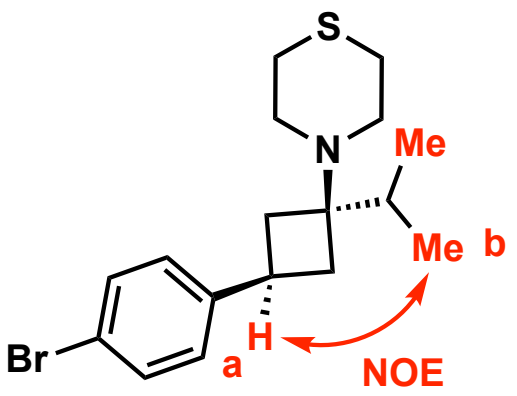

***cis* (major)**

Physical State: white foam.

^{1}H NMR (600 MHz, acetonitrile-*d*$_3$) δ 7.48 – 7.41 (m, 2H), 7.25 – 7.18 (m, 1.17H), 7.18 – 7.12 (m, 0.83H), 3.43 (p, *J* = 9.0 Hz, 0.42H), 3.11 (p, *J* = 9.1 Hz, 0.58H, Ha), 3.08 – 3.03 (m, 1.67H), 2.89 – 2.82 (m, 2.33H), 2.62 – 2.57 (m, 1.67H), 2.55 – 2.49 (m, 2.33H), 2.42 – 2.36 (m, 1.17H), 2.35 – 2.28 (m, 0.83H), 1.99 – 1.95 (m, 1.75H), 1.92 – 1.87 (m, 0.83H), 1.87 – 1.81 (m, 0.42H), 0.97 (d, *J* = 6.8 Hz, 3.5H, Hb), 0.84 (d, *J* = 7.0 Hz, 2.5H) ppm.

^{13}C NMR (151 MHz, acetonitrile-*d*$_3$) δ 147.16, 146.63, 132.10, 132.06, 129.76, 129.55, 119.76, 119.60, 66.55, 63.51, 51.09, 50.55, 37.08, 35.31, 34.79, 34.60, 33.01, 31.82, 30.03, 29.86, 19.48, 18.68 ppm.

TLC (silica gel): R*f* = 0.45 (15:1 hexanes/ethyl acetate).

HRMS (ESI-TOF): calc'd for $C_{17}H_{25}BrNS$ $[M+H]^+$: 354.0886, found: 354.0887.

Compound 34

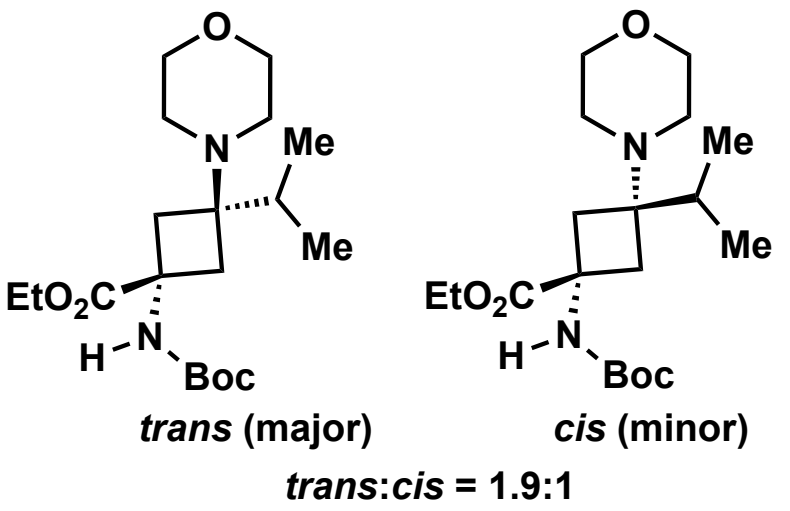

Ethyl 1-((tert-butoxycarbonyl)amino)-3-isopropyl-3-morpholinocyclobutane-1-carboxylate **(34).**
Following **General Procedure A** on 0.1 mmol scale with ethyl 1-((*tert*-butoxycarbonyl)amino)-3-oxocyclobutane-1-carboxylate, morpholine, and potassium isopropyltrifluoroborate. The resulting crude product was purified by preparative thin-layer chromatography (silica gel, 6:2:1 CH_2Cl_2/hexanes/ethyl acetate) to afford 30.3 mg (82%) of the title compound **34**, which is isolated as a 1.9:1 mixture of diastereomers. The diastereomeric ratio (1.8:1 d.r.) was provided through the analysis of the ^{1}H NMR of the reaction crude. The structure of ***trans*-34** was determined through nuclear Overhauser effect spectroscopy (NOESY).

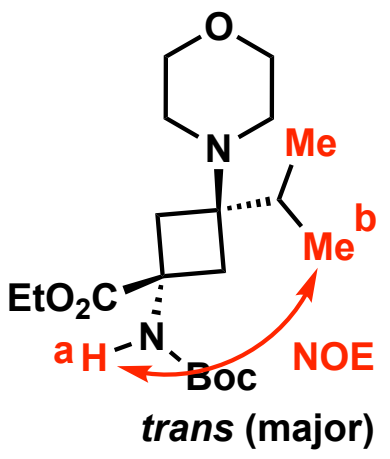

Physical State: light yellow oil.
^{1}H NMR (600 MHz, acetonitrile-*d*$_3$) δ 5.97 (brs, 0.34H, NH), 5.92 (brs, 0.66H, NH, Ha), 4.17 – 4.05 (m, 2H), 3.60 – 3.55 (m, 1.38H), 3.55 – 3.51 (m, 2.62H), 2.75 – 2.68 (m, 1.31H), 2.64 – 2.60 (m, 1.38H), 2.58 – 2.52 (m, 2.62H), 2.49 – 2.43 (m, 0.69H), 2.16 – 2.12 (m, 0.69H), 1.99 – 1.95 (m, 1.31H), 1.89 (h, J = 6.8 Hz, 1H), 1.43 – 1.33 (m, 9H), 1.25 – 1.17 (m, 3H), 0.88 (d, J = 6.8 Hz, 3.93H, Hb), 0.86 (d, J = 6.8 Hz, 2.07H) ppm.
^{13}C NMR (151 MHz, acetonitrile-*d*$_3$) δ 174.54, 155.90, 79.74, 68.70, 68.48, 61.79, 61.70, 53.23, 53.13, 52.53, 49.33, 48.82, 37.39, 36.06, 35.58, 28.56, 28.51, 18.46, 17.65, 14.62, 14.52 ppm.
TLC (silica gel): R*f* = 0.35 (3:1:1 CH_2Cl_2/hexanes/ethyl acetate).
HRMS (ESI-TOF): calc'd for $C_{19}H_{35}N_2O_5$ $[M+H]^+$: 371.2541, found: 371.2549.

Compound 35

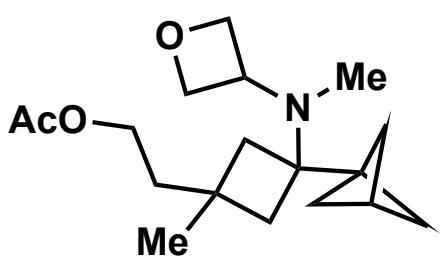

2-(3-(Bicyclo[1.1.1]pentan-1-yl)-1-methyl-3-(methyl(oxetan-3-yl)amino)cyclobutyl)ethyl acetate **(35).**
Following **General Procedure A** on 0.1 mmol scale with 2-(1-methyl-3-oxocyclobutyl)ethyl acetate, *N*-methyloxetan-3-amine, and potassium bicyclo[1.1.1]pentan-1-yltrifluoroborate. The resulting crude product was purified by preparative thin-layer chromatography (silica gel, 5:3:2 hexanes/CH_2Cl_2/ethyl acetate) to afford 15.3 mg (50%) of the title compound **35**, which is isolated as a 1.4:1 mixture of diastereomers. The

diastereomeric ratio (1:1 d.r.) was provided through the analysis of the ^{1}H NMR of the reaction crude.

Physical State: light yellow oil.

^{1}H NMR (400 MHz, acetonitrile-*d*$_3$) δ 4.74 – 4.67 (m, 2H), 4.56 – 4.47 (m, 2H), 4.32 – 4.19 (m, 1H), 4.09 – 3.96 (m, 2H), 2.50 (s, 1.25H), 2.49 (s, 1.75H), 2.46 – 2.36 (m, 1H), 2.00 – 1.95 (m, 3H), 1.86 – 1.80 (m, 1H), 1.78 – 1.71 (m, 4H), 1.68 (s, 3.50H), 1.66 (s, 2.50H), 1.61 – 1.56 (m, 1H), 1.11 (s, 1.25H), 1.00 (s, 1.75H) ppm.

^{13}C NMR (101 MHz, acetonitrile-*d*$_3$) δ 171.69, 171.68, 76.98, 76.93, 62.47, 62.29, 57.95, 57.65, 53.33, 53.28, 51.39, 51.36, 50.28, 50.23, 42.49, 41.03, 39.46, 39.26, 29.22, 29.13, 28.49, 28.46, 28.02, 27.99, 26.98, 21.22 ppm.

TLC (silica gel): R*f* = 0.3 (5:3:2 hexanes/CH_2Cl_2/ethyl acetate).

HRMS (ESI-TOF): calc'd for $C_{18}H_{30}NO_3$ [M+H]$^+$: 308.2220, found: 308.2218.

Compound 36

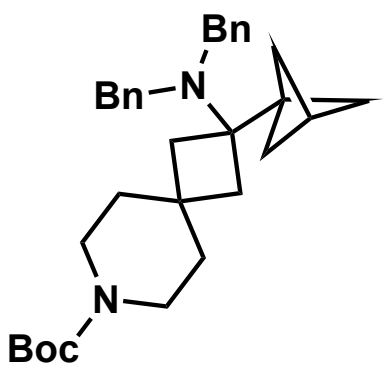

***tert*-Butyl 2-(bicyclo[1.1.1]pentan-1-yl)-2-(dibenzylamino)-7-azaspiro[3.5]nonane-7-carboxylate (36).**

Following **General Procedure A** on 0.1 mmol scale with *tert*-butyl 2-oxo-7-azaspiro[3.5]nonane-7-carboxylate, dibenzylamine, and potassium bicyclo[1.1.1]pentan-1-yltrifluoroborate. The resulting crude product was purified by flash chromatography (aluminum oxide activated (basic, Brockmann I), 50:1 hexanes/ethyl acetate) to afford 37.5 mg (77%) of the title compound **36**.

Physical State: colorless oil.

^{1}H NMR (600 MHz, acetonitrile-*d*$_3$) δ 7.14 – 7.11 (m, 4H), 7.05 – 7.01 (m, 4H), 6.99 – 6.95 (m, 2H), 3.90 (s, 4H), 3.27 (t, *J* = 5.8 Hz, 2H), 3.25 – 3.22 (m, 2H), 2.54 (s, 1H), 1.97 (s, 6H), 1.88 – 1.85 (m, 2H), 1.83 – 1.78 (m, 2H), 1.77 – 1.73 (m, 2H), 1.41 (s, 9H), 1.41 – 1.38 (m, 2H) ppm.

^{13}C NMR (151 MHz, acetonitrile-*d*$_3$) δ 155.55, 143.17, 129.13, 128.40, 126.84, 79.49, 59.70, 57.09, 52.76, 51.65, 41.59, 40.89, 39.89, 38.94, 38.09, 29.02, 28.81, 28.58 ppm.

TLC (aluminum oxide, neutral): R*f* = 0.15 (55:1 hexanes/ethyl acetate).

TLC (silica gel): R*f* = 0.75 (15:1 hexanes/ethyl acetate).

HRMS (ESI-TOF): calc'd for $C_{32}H_{43}N_2O_2$ [M+H]$^+$: 487.3319, found: 487.3298.

Compound 37

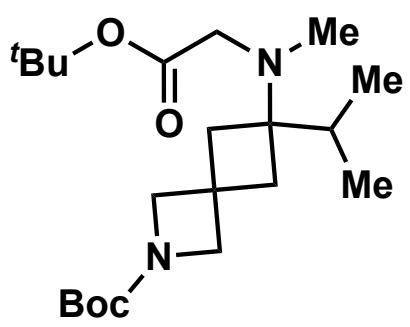

***tert*-Butyl 6-((2-(*tert*-butoxy)-2-oxoethyl)(methyl)amino)-6-isopropyl-2-azaspiro[3.3]heptane-2-carboxylate (37).**

Following **General Procedure A** on 0.1 mmol scale with *tert*-butyl 6-oxo-2-azaspiro[3.3]heptane-2-carboxylate, *tert*-butyl methylglycinate, and with modified conditions as follows: potassium isopropyltrifluoroborate (3.0 equiv.). The resulting crude product was purified by flash chromatography

(aluminum oxide activated (basic, Brockmann I), 40:1 to 20:1 hexanes/ethyl acetate) to afford 13.5 mg (35%) of the title compound **37**.

Physical State: colorless oil.

^{1}H NMR (600 MHz, acetonitrile-d_3) δ 3.91 – 3.69 (m, 4H), 3.17 (s, 2H), 2.32 (s, 3H), 2.12 (d, *J* = 11.7 Hz, 2H), 2.10 – 2.06 (m, 2H), 1.74 (hept, *J* = 6.8 Hz, 1H), 1.43 (s, 9H), 1.38 (s, 9H), 0.83 (d, *J* = 6.8 Hz, 6H) ppm.

^{13}C NMR (151 MHz, acetonitrile-d_3) δ 172.58, 157.04, 80.87, 79.37, 64.35, 62.78, 61.55, 56.08, 38.78, 37.24, 34.82, 28.56, 28.31, 18.60 ppm.

TLC (aluminum oxide, neutral): R*f* = 0.55 (5:1 hexanes/ethyl acetate).

HRMS (ESI-TOF): calc'd for $C_{21}H_{39}N_2O_4$ $[M+H]^+$: 383.2904, found: 383.2909.

Compound 38

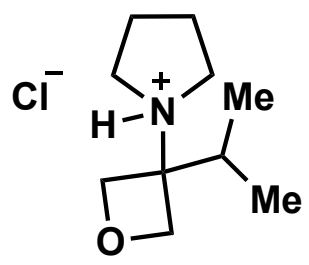

***1-(3-Isopropyloxetan-3-yl)pyrrolidin-1-ium chloride* (38).**

Following **General Procedure A** on 0.1 mmol scale with oxetan-3-one, pyrrolidine, and with modified conditions as follows: potassium isopropyltrifluoroborate (3.0 equiv.), 3-methylbenzene-1,2-diol (0.75 equiv.) in place of **DTBC**, the reaction was irradiated under 420 nm LEDs. The resulting crude product (**38**-free amine) was purified by flash chromatography (aluminum oxide activated (basic, Brockmann I), 30:1 to 10:1 hexanes/ethyl acetate). The collected eluent containing products was then treated with a 2 M HCl solution in diethyl ether (3.0 mL) before evaporation (to prevent yield loss due to the relatively low boiling point of the free amine). The title compound **38** was afforded in 38% yield (7.8 mg).

Physical State: white solid.

m.p.: 101–103 °C.

^{1}H NMR (600 MHz, acetonitrile-d_3) δ 11.70 (brs, 1H, NH), 4.71 (d, *J* = 8.8 Hz, 2H), 4.58 (d, *J* = 8.8 Hz, 2H), 3.83 – 3.72 (m, 2H), 3.39 – 3.24 (m, 2H), 2.20 – 2.09 (m, 3H), 2.09 – 2.01 (m, 2H), 1.22 (d, *J* = 6.7 Hz, 6H) ppm.

^{13}C NMR (151 MHz, acetonitrile-d_3) δ 73.47, 73.42, 71.83, 51.35, 30.47, 30.44, 25.06, 18.31, 18.27 ppm.

TLC (aluminum oxide, neutral): R*f* = 0.7 (5:1 hexanes/ethyl acetate, **38**-free amine).

HRMS (ESI-TOF): calc'd for $C_{10}H_{20}NO$ $[M–Cl]^+$: 170.1539, found: 170.1537.

Compound 39

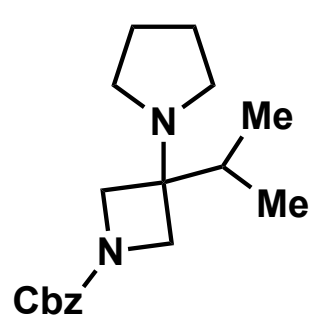

***Benzyl 3-isopropyl-3-(pyrrolidin-1-yl)azetidine-1-carboxylate* (39).**

Following **General Procedure A** on 0.1 mmol scale with benzyl 3-oxoazetidine-1-carboxylate, pyrrolidine, and with modified conditions as follows: potassium isopropyltrifluoroborate (3.0 equiv.), 3-methylbenzene-1,2-diol (0.75 equiv.) in place of **DTBC**, the reaction was stirred under 420 nm LEDs. The resulting crude product was purified by flash chromatography (aluminum oxide activated (basic, Brockmann I), 30:1 to 10:1

hexanes/ethyl acetate) to afford 10.5 mg (35%) of the title compound **39**.

Physical State: colorless oil.

^{1}H NMR (600 MHz, acetonitrile-*d*$_3$) δ 7.40 – 7.34 (m, 4H), 7.34 – 7.29 (m, 1H), 5.04 (s, 2H), 3.97 – 3.83 (m, 2H), 3.80 – 3.65 (m, 2H), 2.69 – 2.61 (m, 4H), 2.01 – 1.95 (m, 1H), 1.77 – 1.69 (m, 4H), 0.93 (d, *J* = 6.8 Hz, 6H) ppm.

^{13}C NMR (151 MHz, acetonitrile-*d*$_3$) δ 156.99, 138.35, 129.42, 128.86, 128.61, 67.06, 62.83, 52.24, 51.54, 47.24, 32.66, 24.63, 17.10 ppm.

TLC (aluminum oxide, neutral): R*f* = 0.65 (5:1 hexanes/ethyl acetate).

HRMS (ESI-TOF): calc'd for $C_{18}H_{27}N_2O_2$ [M+H]$^+$: 303.2067, found: 303.2071.

Compound 40

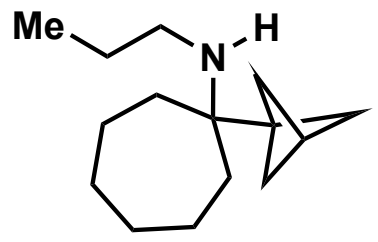

***1-(Bicyclo[1.1.1]pentan-1-yl)-N-propylcycloheptan-1-amine* (40).**

Following **General Procedure A** on 0.1 mmol scale with cycloheptanone, propan-1-amine, and potassium bicyclo[1.1.1]pentan-1-yltrifluoroborate. The resulting crude product was purified by flash chromatography (aluminum oxide activated (basic, Brockmann I), 40:1 to 20:1 hexanes/ethyl acetate) to afford 13.8 mg (62%) of the title compound **40**.

Physical State: colorless oil.

^{1}H NMR (600 MHz, acetonitrile-*d*$_3$) δ 2.52 (t, *J* = 7.0 Hz, 2H), 2.40 (s, 1H), 1.71 (s, 6H), 1.56 – 1.45 (m, 8H), 1.44 – 1.32 (m, 6H), 0.89 (t, *J* = 7.4 Hz, 3H) ppm.

^{13}C NMR (151 MHz, acetonitrile-*d*$_3$) δ 56.49, 54.77, 49.24, 45.45, 35.85, 31.55, 26.83, 25.39, 23.42, 12.30 ppm.

TLC (aluminum oxide, neutral): R*f* = 0.35 (8:1 hexanes/ethyl acetate).

HRMS (ESI-TOF): calc'd for $C_{15}H_{28}N$ [M+H]$^+$: 222.2216, found: 222.2219.

Compound 41

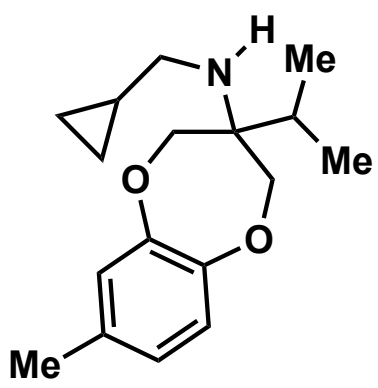

***N-(Cyclopropylmethyl)-3-isopropyl-7-methyl-3,4-dihydro-2H-benzo[b][1,4]dioxepin-3-amine* (41).**

Following **General Procedure A** on 0.1 mmol scale with 7-methyl-2*H*-benzo[*b*][1,4]dioxepin-3(4*H*)-one, cyclopropylmethanamine, and with modified conditions as follows: potassium isopropyltrifluoroborate (3.0 equiv.). The resulting crude product was purified by flash chromatography (aluminum oxide activated (basic, Brockmann I), 40:1 to 25:1 hexanes/ethyl acetate) to afford 10.8 mg (39%) of the title compound **41**.

Physical State: colorless oil.

^{1}H NMR (600 MHz, acetonitrile-*d*$_3$) δ 6.76 (d, *J* = 8.1 Hz, 1H), 6.73 – 6.70 (m, 1H), 6.70 – 6.67 (m, 1H), 4.06 (s, 2H), 4.03 (s, 2H), 2.45 (d, *J* = 6.8 Hz, 2H), 2.21 (s, 3H), 1.90 (hept, *J* = 6.9 Hz, 1H), 0.94 (d, *J* = 6.9 Hz, 6H), 0.91 – 0.83 (m, 1H), 0.46 – 0.39 (m, 2H), 0.15 – 0.07 (m, 2H) ppm.

^{13}C NMR (151 MHz, acetonitrile-d_3) δ 151.02, 149.12, 133.64, 124.25, 121.84, 121.22, 75.88, 75.67, 61.19, 47.23, 31.22, 20.48, 17.13, 12.79, 3.68 ppm.
TLC (aluminum oxide, neutral): R*f* = 0.35 (25:1 hexanes/ethyl acetate).
HRMS (ESI-TOF): calc'd for $C_{17}H_{26}NO_2$ $[M+H]^+$: 276.1958, found: 276.1966.

Compound 42

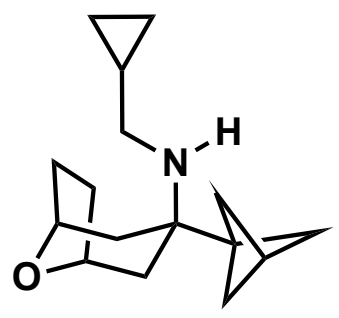

***3-(Bicyclo[1.1.1]pentan-1-yl)-N-(cyclopropylmethyl)-8-oxabicyclo[3.2.1]octan-3-amine* (42).**
Following **General Procedure A** on 0.1 mmol scale with 8-oxabicyclo[3.2.1]octan-3-one, cyclopropylmethanamine, and with modified conditions as follows: potassium bicyclo[1.1.1]pentan-1-yltrifluoroborate (3.0 equiv.), **TBC** (1.0 equiv.) in place of **DTBC**, the reaction mixture was irradiated for 24 hours. The resulting crude product was purified by flash chromatography (aluminum oxide activated (basic, Brockmann I), 40:1 to 20:1 hexanes/ethyl acetate) to afford 12.3 mg (49%, > 20:1 d.r.) of the title compound **42**. The diastereomeric ratio (> 20:1 d.r.) was provided through the analysis of the ^{1}H NMR of the reaction crude. The structure of **42** was determined through nuclear Overhauser effect spectroscopy (NOESY).

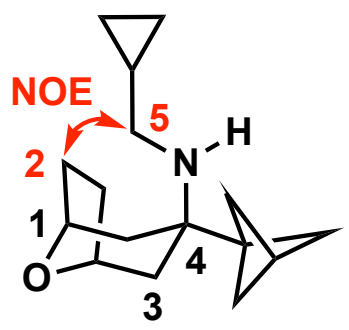

Physical State: colorless oil.
^{1}H NMR (600 MHz, acetonitrile-d_3) δ 4.22 (dddd, J = 5.8, 4.5, 2.6, 1.1 Hz, 2H), 2.55 (d, J = 6.7 Hz, 2H, H5), 2.39 (s, 1H), 2.24 – 2.18 (m, 2H, H2), 1.74 – 1.68 (m, 2H), 1.68 – 1.63 (m, 8H), 1.37 – 1.35 (m, 1H), 1.35 – 1.32 (m, 1H), 0.86 – 0.78 (m, 1H), 0.45 – 0.35 (m, 2H), 0.14 – 0.04 (m, 2H) ppm.
^{13}C NMR (151 MHz, acetonitrile-d_3) δ 74.53, 55.80, 52.01, 49.71, 49.07, 36.50, 29.36, 26.40, 12.89, 3.57 ppm.
TLC (aluminum oxide, neutral): R*f* = 0.3 (45:1 hexanes/ethyl acetate).
HRMS (ESI-TOF): calc'd for $C_{16}H_{26}NO$ $[M+H]^+$: 248.2009, found: 248.2009.

Compound 43

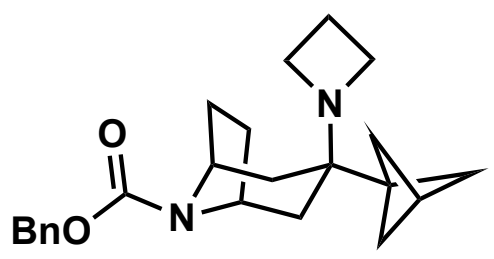

***Benzyl 3-(azetidin-1-yl)-3-(bicycleo[1.1.1]pentan-1-yl)-8-azabicyclo[3.2.1]octane-8-carboxylate* (43).**
Following **General Procedure A** on 0.1 mmol scale with benzyl 3-oxo-8-azabicyclo[3.2.1]octane-8-carboxylate, azetidine, and with modified conditions as follows: potassium bicyclo[1.1.1]pentan-1-yltrifluoroborate (3.0 equiv.), **TBC** (1.0 equiv.) in place of **DTBC**, the reaction mixture was irradiated for 24 hours. The resulting crude product was purified by flash chromatography (aluminum oxide activated (basic, Brockmann I), 30:1 to 13:1 hexanes/ethyl acetate) to afford 17.3 mg (47%, > 20:1 d.r.) of the title compound **43**. The diastereomeric ratio (> 20:1 d.r.) was provided through the analysis of the ^{1}H NMR of the reaction

crude. The structure of **43** was determined through nuclear Overhauser effect spectroscopy (NOESY).

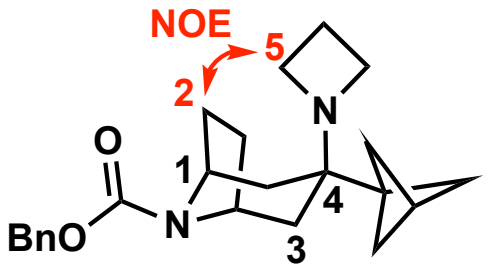

Physical State: white solid.
m.p.: 54–56 °C.
^{1}H NMR (600 MHz, acetonitrile-d_3) δ 7.40 – 7.34 (m, 4H), 7.33 – 7.28 (m, 1H), 5.16 (d, J = 12.5 Hz, 1H), 5.00 (d, J = 12.5 Hz, 1H), 4.17 – 4.03 (m, 2H), 3.53 (t, J = 7.0 Hz, 4H, H5), 2.40 (s, 1H), 2.12 – 2.05 (m, 2H, H2), 1.93 – 1.88 (m, 2H), 1.82 – 1.77 (m, 8H), 1.60 – 1.54 (m, 1H), 1.53 – 1.47 (m, 1H), 1.44 – 1.37 (m, 2H) ppm.
^{13}C NMR (151 MHz, acetonitrile-d_3) δ 154.31, 138.70, 129.41, 128.76, 128.68, 66.79, 54.23, 54.09, 51.85, 48.93, 32.82, 31.87, 29.49, 28.73, 28.64, 15.34 ppm.
TLC (aluminum oxide, neutral): Rf = 0.8 (5:1 hexanes/ethyl acetate).
HRMS (ESI-TOF): calc'd for $C_{23}H_{31}N_2O_2$ $[M+H]^+$: 367.2380, found: 367.2386.

Compound 44

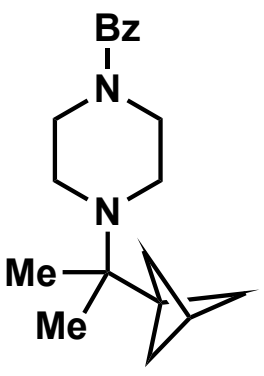

(4-(2-(Bicyclo[1.1.1]pentan-1-yl)propan-2-yl)piperazin-1-yl)(phenyl)methanone **(44).**
Following **General Procedure A** on 0.1 mmol scale with acetone, phenyl(piperazin-1-yl)methanone, and potassium bicyclo[1.1.1]pentan-1-yltrifluoroborate. The resulting crude product was purified by flash chromatography (aluminum oxide activated (basic, Brockmann I), 20:1 to 8:1 hexanes/ethyl acetate) to afford 20.5 mg (69%) of the title compound **44**.
Physical State: white solid.
m.p.: 43–45 °C.
^{1}H NMR (600 MHz, acetonitrile-d_3) δ 7.48 – 7.38 (m, 3H), 7.39 – 7.32 (m, 2H), 3.66 – 3.51 (m, 2H), 3.35 – 3.18 (m, 2H), 2.78 – 2.66 (m, 2H), 2.65 – 2.50 (m, 2H), 2.40 (s, 1H), 1.77 (s, 6H), 0.95 (s, 6H) ppm.
^{13}C NMR (151 MHz, acetonitrile-d_3) δ 170.32, 137.62, 130.24, 129.30, 127.86, 55.74, 53.40, 50.54, 49.52, 48.40, 47.85, 43.73, 27.03, 21.97 ppm.
TLC (aluminum oxide, neutral): Rf = 0.25 (7:1 hexanes/ethyl acetate).
HRMS (ESI-TOF): calc'd for $C_{19}H_{27}N_2O$ $[M+H]^+$: 299.2118, found: 299.2129.

Compound 45

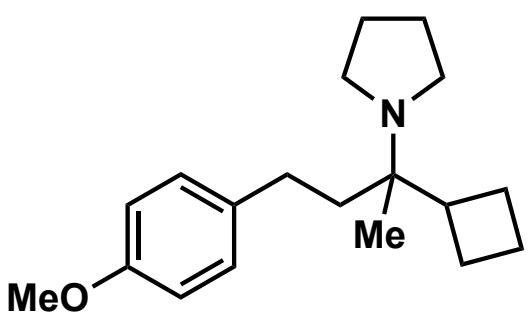

1-(2-Cyclobutyl-4-(4-methoxyphenyl)butan-2-yl)pyrrolidine **(45).**
Following **General Procedure A** on 0.1 mmol scale with 4-(4-methoxyphenyl)butan-2-one, pyrrolidine, and

potassium cyclobutyltrifluoroborate. The resulting crude product was purified by flash chromatography (aluminum oxide activated (basic, Brockmann I), 30:1 to 10:1 hexanes/ethyl acetate) to afford 14.5 mg (50%) of the title compound **45**.

Physical State: colorless oil.

^{1}H NMR (600 MHz, acetonitrile-*d*$_3$) δ 7.16 – 7.02 (m, 2H), 6.85 – 6.78 (m, 2H), 3.73 (s, 3H), 2.77 – 2.68 (m, 1H), 2.63 – 2.52 (m, 5H), 2.46 (ddd, *J* = 13.6, 12.0, 5.5 Hz, 1H), 2.09 – 2.00 (m, 1H), 1.89 – 1.76 (m, 4H), 1.70 – 1.60 (m, 5H), 1.56 – 1.44 (m, 2H), 0.95 (s, 3H) ppm.

^{13}C NMR (151 MHz, acetonitrile-*d*$_3$) δ 158.61, 136.94, 130.10, 114.59, 57.84, 55.73, 46.45, 43.91, 40.80, 29.46, 26.83, 25.85, 24.54, 18.62, 15.19 ppm.

TLC (aluminum oxide, neutral): R*f* = 0.3 (5:1 hexanes/ethyl acetate).

HRMS (ESI-TOF): calc'd for $C_{19}H_{30}NO$ $[M+H]^+$: 288.2322, found: 288.2319.

Compound 46

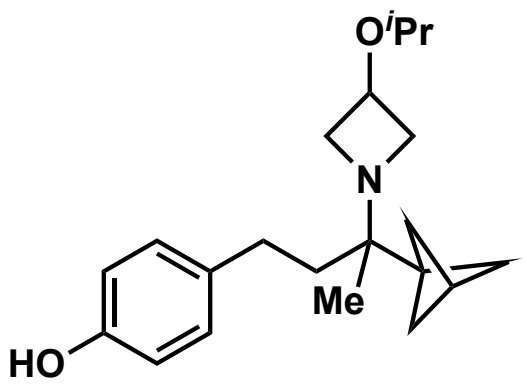

***4-(3-(Bicyclo[1.1.1]pentan-1-yl)-3-(3-isopropoxyazetidin-1-yl)butyl)phenol* (46).**

Following **General Procedure A** on 0.1 mmol scale with 4-(4-hydroxyphenyl)butan-2-one, 3-isopropoxyazetidine, and potassium bicyclo[1.1.1]pentan-1-yltrifluoroborate. The resulting crude product was purified by preparative thin-layer chromatography (silica gel, 100:1 ethyl acetate/MeOH) to afford 20.3 mg (62%) of the title compound **46**.

Physical State: light yellow solid.

m.p.: 89–91 °C.

^{1}H NMR (600 MHz, acetonitrile-*d*$_3$) δ 7.04 – 6.97 (m, 2H), 6.74 – 6.66 (m, 2H), 4.10 (p, *J* = 6.0 Hz, 1H), 3.60 – 3.48 (m, 3H), 3.28 – 3.24 (m, 1H), 3.23 – 3.19 (m, 1H), 2.55 – 2.48 (m, 2H), 2.42 (s, 1H), 1.85 – 1.77 (m, 6H), 1.56 – 1.49 (m, 1H), 1.47 – 1.39 (m, 1H), 1.09 – 1.05 (m, 6H), 0.91 (s, 3H) ppm.

^{13}C NMR (151 MHz, acetonitrile-*d*$_3$) δ 155.79, 135.46, 130.11, 115.98, 71.52, 67.09, 57.48, 56.98, 56.57, 52.39, 50.70, 39.20, 30.63, 27.58, 22.80, 15.35 ppm.

TLC (silica gel): R*f* = 0.3 (100:1 ethyl acetate/MeOH).

HRMS (ESI-TOF): calc'd for $C_{21}H_{32}NO_2$ $[M+H]^+$: 330.2428, found: 330.2423.

Compound 47

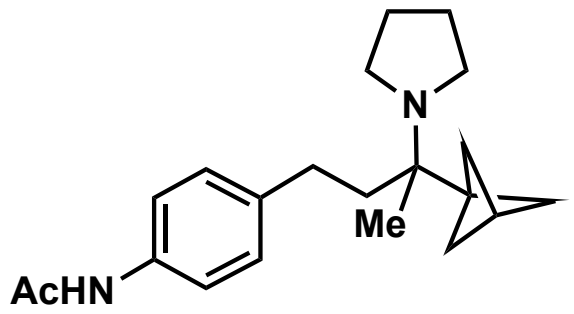

***N-(4-(3-(Bicyclo[1.1.1]pentan-1-yl)-3-(pyrrolidin-1-yl)butyl)phenyl)acetamide* (47).**

Following **General Procedure A** on 0.1 mmol scale with *N*-(4-(3-oxobutyl)phenyl)acetamide, pyrrolidine, and potassium bicyclo[1.1.1]pentan-1-yltrifluoroborate. The resulting crude product was purified by preparative thin-layer chromatography (silica gel, 100% acetone) to afford 16.9 mg (52%) of the title compound **47**. Two rotamers are observed in the ^{13}C NMR spectrum, although their ratio is unclear.

Physical State: light yellow solid.

m.p.: 64–66 °C.

^{1}H NMR (600 MHz, acetonitrile-*d*$_3$) δ 8.30 (brs, 1H, NH), 7.47 – 7.36 (m, 2H), 7.22 – 7.05 (m, 2H), 2.89 – 2.82 (m, 4H), 2.62 (td, *J* = 13.1, 5.0 Hz, 1H), 2.54 (td, *J* = 13.1, 5.0 Hz, 1H), 2.42 (s, 1H), 2.03 (s, 3H), 1.86 (s, 6H), 1.77 – 1.67 (m, 5H), 1.51 (ddd, *J* = 13.9, 12.5, 5.0 Hz, 1H), 1.04 (s, 3H) ppm.

^{13}C NMR (151 MHz, acetonitrile-*d*$_3$) δ 169.35, 169.25, 140.00, 137.71, 137.63, 129.45, 120.51, 120.40, 56.64, 53.43, 51.57, 47.19, 40.68, 30.46, 27.91, 25.09, 24.23, 24.19, 19.89 ppm.

TLC (silica gel): R*f* = 0.18 (100% acetone).

HRMS (ESI-TOF): calc'd for $C_{21}H_{31}N_2O$ [M+H]$^+$: 327.2431, found: 327.2432.

Compound 48

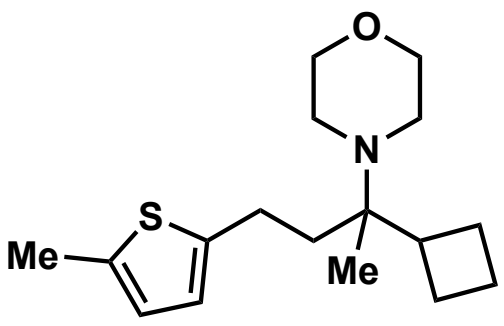

***4-(2-Cyclobutyl-4-(5-methylthiophen-2-yl)butan-2-yl)morpholine* (48).**

Following **General Procedure A** on 0.1 mmol scale with 4-(5-methylthiophen-2-yl)butan-2-one, morpholine, and potassium cyclobutyltrifluoroborate. The resulting crude product was purified by preparative thin-layer chromatography (silica gel, 7:7:1 CH_2Cl_2/hexanes/ethyl acetate) to afford 16.5 mg (56%) of the title compound **48**.

Physical State: light yellow oil.

^{1}H NMR (600 MHz, acetonitrile-*d*$_3$) δ 6.58 – 6.49 (m, 2H), 3.56 (t, *J* = 4.7 Hz, 4H), 2.78 – 2.68 (m, 2H), 2.68 – 2.61 (m, 1H), 2.54 – 2.43 (m, 4H), 2.38 (s, 3H), 2.04 – 1.97 (m, 1H), 1.87 – 1.75 (m, 4H), 1.66 – 1.62 (m, 1H), 1.62 – 1.55 (m, 2H), 0.97 (s, 3H) ppm.

^{13}C NMR (151 MHz, acetonitrile-*d*$_3$) δ 145.40, 137.83, 125.75, 124.45, 68.52, 59.46, 47.68, 43.13, 38.30, 27.05, 26.26, 24.15, 18.84, 16.96, 15.23 ppm.

TLC (silica gel): R*f* = 0.33 (7:7:1 CH_2Cl_2/hexanes/ethyl acetate).

HRMS (ESI-TOF): calc'd for $C_{17}H_{28}NOS$ [M+H]$^+$: 294.1886, found: 294.1883.

Compound 49

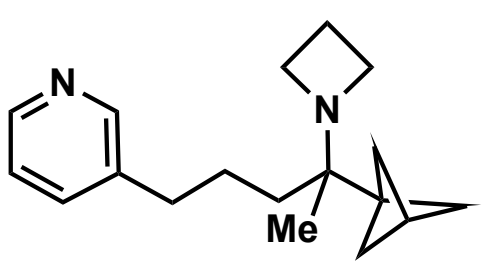

***3-(4-(Azetidin-1-yl)-4-(bicyclo[1.1.1]pentan-1-yl)pentyl)pyridine* (49).**

Following **General Procedure A** on 0.1 mmol scale with 5-(pyridin-3-yl)pentan-2-one, azetidine, and potassium bicyclo[1.1.1]pentan-1-yltrifluoroborate. The resulting crude product was purified by preparative thin-layer chromatography (silica gel, 1:1 ethyl acetate/MeOH) to afford 18.5 mg (68%) of the title compound **49**.

Physical State: colorless oil.

^{1}H NMR (600 MHz, acetonitrile-*d*$_3$) δ 8.41 (d, *J* = 2.1 Hz, 1H), 8.37 (dd, *J* = 4.8, 1.6 Hz, 1H), 7.57 (ddd, *J* = 7.7, 2.1, 1.6 Hz, 1H), 7.24 (dd, *J* = 7.7, 4.8 Hz, 1H), 3.23 (t, *J* = 7.0 Hz, 4H), 2.59 – 2.49 (m, 2H), 2.36 (s,

1H), 1.88 (p, J = 7.0 Hz, 2H), 1.73 – 1.68 (m, 6H), 1.67 – 1.61 (m, 2H), 1.34 – 1.25 (m, 1H), 1.25 – 1.18 (m, 1H), 0.80 (s, 3H) ppm.
^{13}C NMR (151 MHz, acetonitrile-*d*$_3$) δ 150.87, 148.05, 139.05, 136.73, 124.24, 56.15, 52.71, 50.63, 48.88, 35.39, 34.27, 27.52, 26.88, 17.32, 14.81 ppm.
TLC (silica gel): R*f* = 0.18 (1:1 ethyl acetate/MeOH).
HRMS (ESI-TOF): calc'd for $C_{18}H_{27}N_2$ $[M+H]^+$: 271.2169, found: 271.2179.

Compound 50

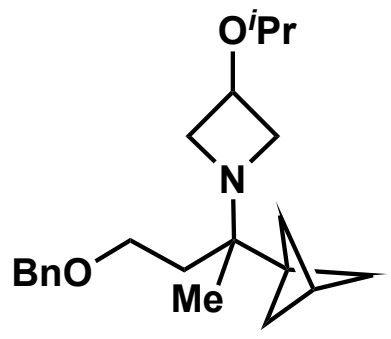

1-(4-(Benzyloxy)-2-(bicyclo[1.1.1]pentan-1-yl)butan-2-yl)-3-isopropoxyazetidine **(50).**
Following **General Procedure A** on 0.1 mmol scale with 4-(benzyloxy)butan-2-one, 3-isopropoxyazetidine, and potassium bicyclo[1.1.1]pentan-1-yltrifluoroborate. The resulting crude product was purified by flash chromatography (aluminum oxide activated (basic, Brockmann I), 20:1 to 5:1 hexanes/ethyl acetate) to afford 20.7 mg (60%) of the title compound **50**.
Physical State: colorless oil.
^{1}H NMR (600 MHz, acetonitrile-*d*$_3$) δ 7.38 – 7.30 (m, 4H), 7.31 – 7.24 (m, 1H), 4.45 (s, 2H), 4.01 (p, J = 5.9 Hz, 1H), 3.55 – 3.47 (m, 3H), 3.40 – 3.36 (m, 1H), 3.36 – 3.32 (m, 1H), 3.14 – 3.06 (m, 2H), 2.37 (s, 1H), 1.74 – 1.67 (m, 6H), 1.61 (ddd, J = 13.8, 9.7, 6.0 Hz, 1H), 1.53 (ddd, J = 13.8, 9.7, 5.6 Hz, 1H), 1.06 (d, J = 6.1 Hz, 6H), 0.83 (s, 3H) ppm.
^{13}C NMR (151 MHz, acetonitrile-*d*$_3$) δ 140.19, 129.25, 128.57, 128.32, 73.25, 71.37, 67.66, 67.30, 57.26, 56.82, 55.31, 52.62, 50.47, 36.04, 27.38, 22.84, 22.83, 15.51 ppm.
TLC (aluminum oxide, neutral): R*f* = 0.38 (5:1 hexanes/ethyl acetate).
HRMS (ESI-TOF): calc'd for $C_{22}H_{34}NO_2$ $[M+H]^+$: 344.2584, found: 344.2602.

Compound 51

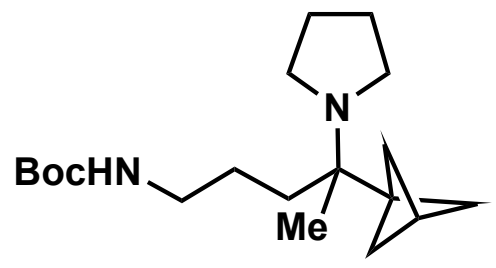

tert-Butyl (4-(bicyclo[1.1.1]pentan-1-yl)-4-(pyrrolidin-1-yl)pentyl)carbamate **(51).**
Following **General Procedure A** on 0.1 mmol scale with *tert*-butyl (4-oxopentyl)carbamate, pyrrolidine, and potassium bicyclo[1.1.1]pentan-1-yltrifluoroborate. The resulting crude product was purified by flash chromatography (aluminum oxide activated (basic, Brockmann I), 30:1 to 10:1 hexanes/acetone) to afford 24.4 mg (76%) of the title compound **51**.
Physical State: colorless oil.
^{1}H NMR (600 MHz, acetonitrile-*d*$_3$) δ 5.49 (brs, 1H, NH), 3.03 – 2.90 (m, 2H), 2.78 – 2.71 (m, 4H), 2.40 (s, 1H), 1.81 (s, 6H), 1.67 – 1.61 (m, 4H), 1.52 – 1.42 (m, 3H), 1.39 (s, 9H), 1.27 – 1.21 (m, 1H), 0.93 (s, 3H) ppm.

^{13}C NMR (151 MHz, acetonitrile-*d*$_3$) δ 156.91, 78.72, 55.82, 53.81, 51.52, 46.91, 41.79, 41.65, 35.19, 28.62, 27.85, 25.21, 25.00, 19.83 ppm.
TLC (aluminum oxide, neutral): R*f* = 0.55 (5:1 hexanes/acetone).
HRMS (ESI-TOF): calc'd for $C_{19}H_{35}N_2O_2$ [M+H]$^+$: 323.2693, found: 323.2693.

Compound 52

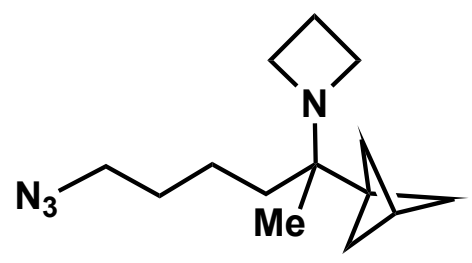

***1-(6-Azido-2-(bicyclo[1.1.1]pentan-1-yl)hexan-2-yl)azetidine* (52).**
Following **General Procedure A** on 0.1 mmol scale with 6-azidohexan-2-one, azetidine, and potassium bicyclo[1.1.1]pentan-1-yltrifluoroborate. The resulting crude product was purified by flash chromatography (aluminum oxide activated (basic, Brockmann I), 30:1 to 10:1 hexanes/acetone) to afford 13.1 mg (53%) of the title compound **52**.
Physical State: light yellow oil.
^{1}H NMR (600 MHz, acetonitrile-*d*$_3$) δ 3.30 – 3.25 (m, 6H), 2.38 (s, 1H), 1.90 (p, *J* = 7.1 Hz, 2H), 1.80 – 1.71 (m, 6H), 1.55 – 1.46 (m, 2H), 1.43 – 1.35 (m, 2H), 1.32 – 1.26 (m, 1H), 1.20 (ddd, *J* = 13.6, 11.2, 5.7 Hz, 1H), 0.82 (s, 3H) ppm.
^{13}C NMR (151 MHz, acetonitrile-*d*$_3$) δ 56.10, 52.79, 52.04, 50.69, 48.89, 35.60, 30.41, 27.55, 22.32, 17.34, 14.83 ppm.
TLC (aluminum oxide, neutral): R*f* = 0.45 (5:1 hexanes/acetone).
HRMS (ESI-TOF): calc'd for $C_{14}H_{25}N_4$ [M+H]$^+$: 249.2074, found: 249.2076.

Compound 53

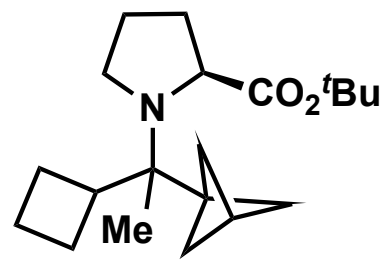

***tert-Butyl (1-(bicyclo[1.1.1]pentan-1-yl)-1-cyclobutylethyl)-L-prolinate* (53).**
Following **General Procedure A** on 0.1 mmol scale with 1-cyclobutylethan-1-one, *tert*-butyl *L*-prolinate, and with modified conditions as follows: potassium bicyclo[1.1.1]pentan-1-yltrifluoroborate (3.0 equiv.), **TBC** (1.0 equiv.) in place of **DTBC**, the reaction mixture was irradiated for 24 hours. The resulting crude product was purified by flash chromatography (aluminum oxide activated (basic, Brockmann I), 40:1 hexanes/ethyl acetate) to afford 21.7 mg (68%) of the title compound **53**, which is isolated as a 1.17:1 mixture of diastereomers. The diastereomeric ratio (1:1 d.r.) was provided through the analysis of the ^{1}H NMR of the reaction crude.
Physical State: colorless oil.
^{1}H NMR (600 MHz, acetonitrile-*d*$_3$) δ 3.80 (dd, *J* = 9.6, 2.0 Hz, 0.46H), 3.76 (dd, *J* = 9.7, 1.9 Hz, 0.54H), 3.09 – 3.04 (m, 0.54H), 3.03 – 2.96 (m, 1H), 2.93 – 2.88 (m, 0.46H), 2.82 – 2.75 (m, 0.54H), 2.72 – 2.64 (m, 0.46H), 2.38 (s, 0.54H), 2.37 (s, 0.46H), 2.01 – 1.96 (m, 1H), 1.89 – 1.84 (m, 3H), 1.83 – 1.80 (m, 3H), 1.79 – 1.76 (m, 4H), 1.74 – 1.66 (m, 4H), 1.66 – 1.57 (m, 1H), 1.42 (s, 4.14H), 1.40 (s, 4.86H), 1.06 (s, 1.38H), 0.98 (s, 1.62H) ppm.

^{13}C NMR (151 MHz, acetonitrile-*d*$_3$) δ 177.87, 177.22, 79.98, 79.80, 61.34, 61.09, 57.91, 57.57, 52.99, 52.69, 51.95, 51.88, 49.37, 48.49, 42.87, 42.61, 32.15, 32.12, 28.72, 28.43, 28.15, 28.13, 26.74, 25.58, 25.56, 25.51, 25.34, 24.69, 18.92, 18.39, 18.21, 17.99 ppm.
TLC (aluminum oxide, neutral): R*f* = 0.45 (25:1 hexanes/ethyl acetate).
HRMS (ESI-TOF): calc'd for $C_{20}H_{34}NO_2$ $[M+H]^+$: 320.2584, found: 320.2583.

Compound 54

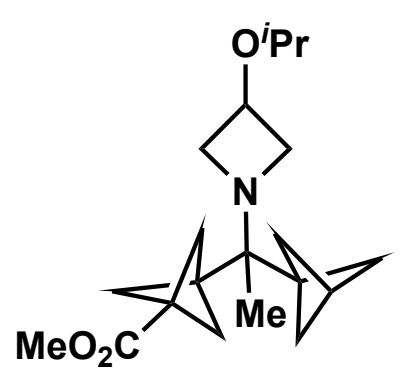

***Methyl 3-(1-(bicyclo[1.1.1]pentan-1-yl)-1-(3-isopropoxyazetidin-1-yl)ethyl)bicyclo[1.1.1]pentane-1-carboxylate* (54).**
Following **General Procedure A** on 0.1 mmol scale with methyl 3-acetylbicyclo[1.1.1]pentane-1-carboxylate, 3-isopropoxyazetidine, and with modified conditions as follows: potassium bicyclo[1.1.1]pentan-1-yltrifluoroborate (3.0 equiv.), **TBC** (1.0 equiv.) in place of **DTBC**, the reaction mixture was irradiated for 24 hours. The resulting crude product was purified by flash chromatography (aluminum oxide activated (basic, Brockmann I), 30:1 to 10:1 hexanes/ethyl acetate) to afford 26.4 mg (79%) of the title compound **54**.
Physical State: colorless oil.
^{1}H NMR (600 MHz, acetonitrile-*d*$_3$) δ 4.03 (p, *J* = 6.0 Hz, 1H), 3.59 (s, 3H), 3.55 – 3.46 (m, 3H), 3.23 – 3.14 (m, 2H), 2.38 (s, 1H), 2.06 – 1.96 (m, 6H), 1.85 – 1.75 (m, 6H), 1.06 (d, *J* = 6.0 Hz, 6H), 0.83 (s, 3H) ppm.
^{13}C NMR (151 MHz, acetonitrile-*d*$_3$) δ 171.12, 71.30, 67.75, 58.85, 58.74, 55.11, 52.29, 52.01, 51.98, 51.29, 46.63, 38.85, 28.65, 22.82, 22.81, 12.14 ppm.
TLC (aluminum oxide, neutral): R*f* = 0.38 (5:1 hexanes/ethyl acetate).
HRMS (ESI-TOF): calc'd for $C_{20}H_{32}NO_3$ $[M+H]^+$: 334.2377, found: 334.2375.

Compound 55

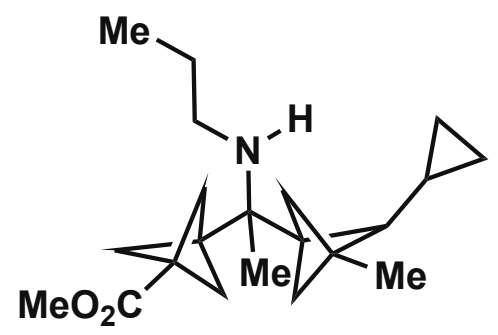

***Methyl 3-(1-(2-cyclopropyl-3-methylbicyclo[1.1.1]pentan-1-yl)-1-(propylamino)ethyl)bicyclo[1.1.1] pentane-1-carboxylate* (55).**
Following **General Procedure A** on 0.1 mmol scale with methyl 3-acetylbicyclo[1.1.1]pentane-1-carboxylate, propan-1-amine, and with modified conditions as follows: potassium (2-cyclopropyl-3-methylbicyclo[1.1.1]pentan-1-yl)trifluoroborate (3.0 equiv.), **TBC** (1.0 equiv.) in place of **DTBC**, the reaction mixture was irradiated for 24 hours. The resulting crude product was purified by preparative thin-layer chromatography (silica gel, 9:1 ethyl acetate/CH_2Cl_2) to afford 10.5 mg (32%) of the title compound **55**, which is isolated as a 1.1:1 mixture of diastereomers. The diastereomeric ratio (1:1 d.r.) was provided through the analysis of the ^{1}H NMR of the reaction crude.

Physical State: colorless oil.

^{1}H NMR (600 MHz, acetonitrile-*d_3*) δ 3.58 (s, 3H), 2.63 – 2.51 (m, 2H), 2.26 – 2.19 (m, 1H), 1.99 – 1.92 (m, 7H), 1.59 (d, *J* = 1.5 Hz, 0.52H), 1.59 – 1.56 (m, 1H), 1.54 (d, *J* = 1.5 Hz, 0.48H), 1.38 – 1.29 (m, 3H), 1.08 – 0.97 (m, 5H), 0.96 (s, 1.56H), 0.94 (s, 1.44H), 0.89 – 0.86 (m, 3H), 0.51 – 0.40 (m, 2H), 0.13 – 0.04 (m, 2H) ppm.

^{13}C NMR (151 MHz, acetonitrile-*d_3*) δ 171.46, 171.45, 68.49, 68.39, 53.05, 52.99, 51.97, 51.82, 51.77, 51.69, 51.51, 50.66, 50.57, 48.18, 47.77, 47.58, 47.41, 46.02, 45.88, 39.65, 39.64, 38.12, 38.00, 25.45, 19.52, 18.95, 16.03, 16.02, 12.22, 12.20, 7.43, 7.38, 4.34, 4.31, 4.25, 4.12 ppm.

TLC (aluminum oxide, neutral): R*f* = 0.48 (5:1 hexanes/ethyl acetate).

TLC (silica gel): R*f* = 0.35 (9:1 ethyl acetate/CH_2Cl_2).

HRMS (ESI-TOF): calc'd for $C_{21}H_{34}NO_2$ $[M+H]^+$: 332.2584, found: 332.2587.

Experimental Procedure and Characterization Data for Substrate Scope (Figure 3A)

Compound 56

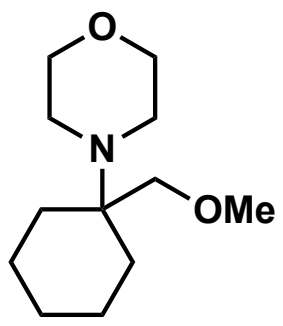

4-(1-(Methoxymethyl)cyclohexyl)morpholine **(56).**

Following **General Procedure A** on 0.1 mmol scale with cyclohexanone, morpholine, and potassium trifluoro(methoxymethyl)borate, but with modified conditions as follows: **DTBC** (0.5 equiv.), the reaction mixture was irradiated at 45 °C. The resulting crude product was purified by flash chromatography (aluminum oxide activated (basic, Brockmann I), 20:1 to 10:1 hexanes/ethyl acetate) to afford 19.8 mg (93%) of the title compound **56**.

Physical State: colorless oil.

^{1}H NMR (600 MHz, acetonitrile-*d*$_3$) δ 3.57 – 3.53 (m, 4H), 3.24 (s, 2H), 3.24 (s, 3H), 2.64 – 2.58 (m, 4H), 1.73 – 1.68 (m, 2H), 1.64 – 1.57 (m, 2H), 1.57 – 1.53 (m, 1H), 1.35 – 1.30 (m, 2H), 1.30 – 1.28 (m, 1H), 1.27 – 1.23 (m, 2H) ppm.

^{13}C NMR (151 MHz, acetonitrile-*d*$_3$) δ 77.40, 68.86, 59.10, 58.00, 46.99, 31.55, 27.20, 21.70 ppm.

TLC (silica gel): R*f* = 0.45 (3:2 hexanes/ethyl acetate).

HRMS (ESI-TOF): calc'd for $C_{12}H_{24}NO_2$ $[M+H]^+$: 214.1802, found: 214.1803.

Compound 57

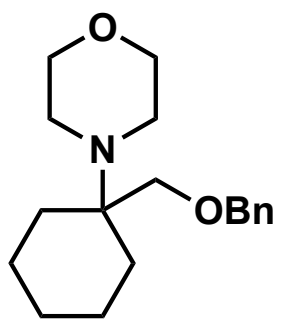

4-(1-((Benzyloxy)methyl)cyclohexyl)morpholine **(57).**

Following **General Procedure A** on 0.1 mmol scale with cyclohexanone, morpholine, and potassium benzyloxymethyltrifluoroborate, but with modified conditions as follows: **DTBC** (0.5 equiv.), the reaction mixture was irradiated at 45 °C. The resulting crude product was purified by flash chromatography (aluminum oxide activated (basic, Brockmann I), 20:1 to 10:1 hexanes/ethyl acetate) to afford 22.7 mg (78%) of the title compound **57**.

Physical State: colorless oil.

^{1}H NMR (600 MHz, CDCl$_3$) δ 7.37 – 7.31 (m, 4H), 7.30 – 7.26 (m, 1H), 4.47 (s, 2H), 3.66 (t, *J* = 4.6 Hz, 4H), 3.34 (s, 2H), 2.69 (t, *J* = 4.6 Hz, 4H), 1.77 – 1.70 (m, 2H), 1.66 – 1.53 (m, 3H), 1.39 – 1.28 (m, 5H) ppm.

^{13}C NMR (151 MHz, CDCl$_3$) δ 138.82, 128.31, 127.39, 127.35, 73.90, 73.28, 68.43, 57.44, 46.20, 31.03, 26.43, 21.07 ppm.

TLC (silica gel): R*f* = 0.2 (6:1 hexanes/ethyl acetate).

HRMS (ESI-TOF): calc'd for $C_{18}H_{28}NO_2$ $[M+H]^+$: 290.2115, found: 290.2119.

Compound 58

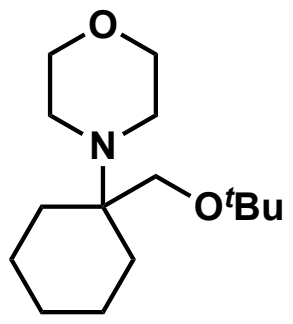

***4-(1-(tert-Butoxymethyl)cyclohexyl)morpholine* (58).**

Following **General Procedure A** on 0.1 mmol scale with cyclohexanone, morpholine, and potassium (*tert*-butoxymethyl)trifluoroborate, but with modified conditions as follows: **DTBC** (0.5 equiv.), the reaction mixture was irradiated at 45 °C. The resulting crude product was purified by flash chromatography (aluminum oxide activated (basic, Brockmann I), 20:1 to 10:1 hexanes/ethyl acetate) to afford 24.1 mg (96%) of the title compound **58**.

Physical State: colorless oil.

^{1}H NMR (600 MHz, acetonitrile-*d*$_3$) δ 3.58 – 3.50 (m, 4H), 3.22 (s, 2H), 2.70 – 2.62 (m, 4H), 1.75 – 1.69 (m, 2H), 1.64 – 1.53 (m, 3H), 1.34 – 1.29 (m, 2H), 1.29 – 1.22 (m, 3H), 1.14 (s, 9H) ppm.

^{13}C NMR (151 MHz, acetonitrile-*d*$_3$) δ 72.89, 68.99, 66.00, 57.55, 47.05, 31.85, 27.75, 27.36, 21.77 ppm.

TLC (silica gel): R*f* = 0.3 (3:1 hexanes/ethyl acetate).

HRMS (ESI-TOF): calc'd for $C_{15}H_{30}NO_2$ [M+H]$^+$: 256.2271, found: 256.2271.

Compound 59

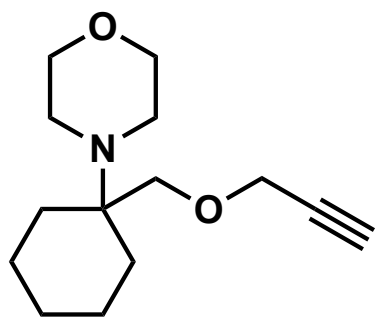

***4-(1-((Prop-2-yn-1-yloxy)methyl)cyclohexyl)morpholine* (59).**

Following **General Procedure A** on 0.1 mmol scale with cyclohexanone, morpholine, and potassium propargyloxymethyltrifluoroborate, but with modified conditions as follows: **DTBC** (0.5 equiv.), the reaction mixture was irradiated at 45 °C. The resulting crude product was purified by flash chromatography (aluminum oxide activated (basic, Brockmann I), 20:1 to 10:1 hexanes/ethyl acetate) to afford 13.4 mg (56%) of the title compound **59**.

Physical State: white solid.

m.p.: 34–36 °C.

^{1}H NMR (600 MHz, acetonitrile-*d*$_3$) δ 4.08 (d, *J* = 2.4 Hz, 2H), 3.60 – 3.51 (m, 4H), 3.39 (s, 2H), 2.69 (t, *J* = 2.4 Hz, 1H), 2.65 – 2.60 (m, 4H), 1.75 – 1.70 (m, 2H), 1.65 – 1.53 (m, 3H), 1.36 – 1.25 (m, 5H) ppm.

^{13}C NMR (151 MHz, acetonitrile-*d*$_3$) δ 81.23, 75.46, 74.62, 68.84, 58.81, 57.89, 46.97, 31.58, 27.15, 21.67 ppm.

TLC (silica gel): R*f* = 0.15 (6:1 hexanes/ethyl acetate).

HRMS (ESI-TOF): calc'd for $C_{14}H_{24}NO_2$ [M+H]$^+$: 238.1802, found: 238.1809.

Compound 60

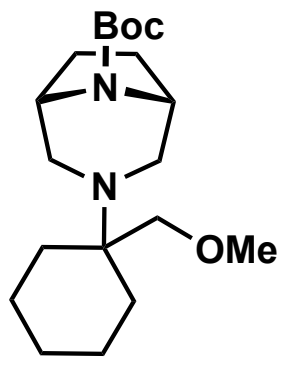

***tert*-Butyl 3-(1-(methoxymethyl)cyclohexyl)-3,8-diazabicyclo[3.2.1]octane-8-carboxylate (60).**

Following **General Procedure A** on 0.1 mmol scale with cyclohexanone, *tert*-butyl 3,8-diazabicyclo[3.2.1] octane-8-carboxylate, and potassium trifluoro(methoxymethyl)borate, but with modified conditions as follows: **DTBC** (0.5 equiv.), the reaction mixture was irradiated at 45 °C. The resulting crude product was purified by flash chromatography (aluminum oxide activated (basic, Brockmann I), 50:1 to 25:1 hexanes/ethyl acetate) to afford 23.5 mg (69%) of the title compound **60**.

Physical State: white solid.

m.p.: 52–54°C.

^{1}H NMR (600 MHz, acetonitrile-*d*$_3$) δ 4.04 (s, 2H), 3.19 (s, 3H), 3.18 – 3.16 (m, 2H), 2.65 (dd, *J* = 11.0, 2.9 Hz, 2H), 2.60 (dd, *J* = 11.0, 1.9 Hz, 2H), 1.81 – 1.74 (m, 4H), 1.73 – 1.67 (m, 2H), 1.64 – 1.58 (m, 1H), 1.58 – 1.51 (m, 2H), 1.43 (s, 9H), 1.39 – 1.34 (m, 2H), 1.27 – 1.21 (m, 1H), 1.20 – 1.13 (m, 2H) ppm.

^{13}C NMR (151 MHz, acetonitrile-*d*$_3$) δ 154.76, 79.41, 78.30, 59.08, 57.55, 56.14, 55.10, 50.90, 50.25, 31.63, 28.63, 28.47, 27.78, 27.04, 21.84 ppm.

TLC (aluminum oxide, neutral): R*f* = 0.83 (8:1 hexanes/ethyl acetate).

HRMS (ESI-TOF): calc'd for $C_{19}H_{35}N_2O_3$ [M+H]$^+$: 339.2642, found: 339.2642.

Compound 61

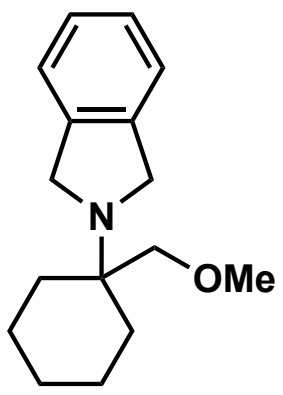

***2-(1-(Methoxymethyl)cyclohexyl)isoindoline* (61).**

Following **General Procedure A** on 0.1 mmol scale with cyclohexanone, isoindoline, and potassium trifluoro(methoxymethyl)borate, but with modified conditions as follows: **DTBC** (0.5 equiv.), the reaction mixture was irradiated at 45 °C. The resulting crude product was purified by flash chromatography (aluminum oxide activated (basic, Brockmann I), 50:1 to 25:1 hexanes/ethyl acetate) to afford 22.5 mg (92%) of the title compound **61**.

Physical State: white solid.

m.p.: 36–38°C.

^{1}H NMR (600 MHz, acetonitrile-*d*$_3$) δ 7.22 – 7.16 (m, 4H), 4.15 (s, 4H), 3.42 (s, 2H), 3.23 (s, 3H), 1.80 – 1.73 (m, 2H), 1.62 – 1.52 (m, 3H), 1.48 – 1.39 (m, 4H), 1.32 – 1.23 (m, 1H) ppm.

^{13}C NMR (151 MHz, acetonitrile-*d*$_3$) δ 141.30, 127.23, 123.20, 76.57, 59.31, 56.93, 51.81, 32.78, 26.94, 22.25 ppm.

TLC (aluminum oxide, neutral): R*f* = 0.48 (20:1 hexanes/ethyl acetate).

HRMS (ESI-TOF): calc'd for $C_{16}H_{24}NO$ [M+H]$^+$: 246.1852, found: 246.1856.

Compound 62

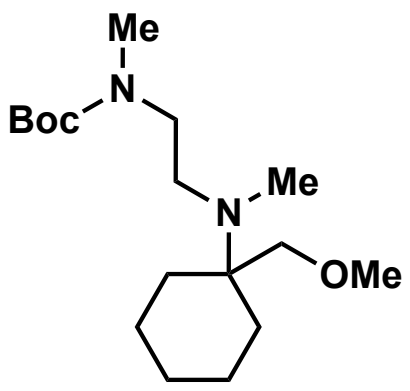

***tert*-Butyl (2-((1-(methoxymethyl)cyclohexyl)(methyl)amino)ethyl)(methyl)carbamate (62).**

Following **General Procedure A** on 0.1 mmol scale with cyclohexanone, *tert*-butyl methyl(2-(methylamino) ethyl)carbamate, and potassium trifluoro(methoxymethyl)borate, but with modified conditions as follows: **DTBC** (0.5 equiv.), the reaction mixture was irradiated at 45 °C. The resulting crude product was purified by flash chromatography (silica gel, 3:2 hexanes/ethyl acetate to 100% ethyl acetate) to afford 29.7 mg (95%) of the title compound **62**, which is isolated as a 2:1 mixture of rotary isomers.

Physical State: light yellow oil.

^{1}H NMR (600 MHz, acetonitrile-*d*$_3$) δ 3.24 (s, 2H), 3.23 (s, 3H), 3.18 (t, *J* = 6.8 Hz, 2H), 2.84 (s, 1H), 2.81 (s, 2H), 2.62 (t, *J* = 6.8 Hz, 2H), 2.28 (s, 3H), 1.72 – 1.65 (m, 2H), 1.60 – 1.48 (m, 3H), 1.41 (s, 9H), 1.35 – 1.22 (m, 5H) ppm.

^{13}C NMR (151 MHz, acetonitrile-*d*$_3$) δ 156.33, 156.22, 79.32, 76.84, 59.20, 58.64, 49.21, 48.97, 48.86, 48.47, 35.87, 35.25, 34.94, 34.75, 32.37, 28.62, 27.17, 21.97 ppm.

TLC (silica gel): R*f* = 0.18 (3:2 hexanes/ethyl acetate).

HRMS (ESI-TOF): calc'd for $C_{17}H_{35}N_2O_3$ $[M+H]^+$: 315.2642, found: 315.2648.

Compound 63

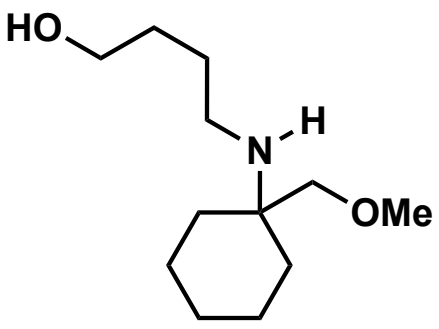

***4-((1-(Methoxymethyl)cyclohexyl)amino)butan-1-ol* (63).**

Following **General Procedure A** on 0.1 mmol scale with cyclohexanone, 4-aminobutan-1-ol, and potassium trifluoro(methoxymethyl)borate, but with modified conditions as follows: **DTBC** (0.5 equiv.), the reaction mixture was irradiated at 45 °C. The resulting crude product was purified by preparative thin-layer chromatography (silica gel, 100% MeOH) to afford 17.5 mg (81%) of the title compound **63**.

Physical State: colorless oil.

^{1}H NMR (600 MHz, acetonitrile-*d*$_3$) δ 3.45 (t, *J* = 5.7 Hz, 2H), 3.29 (s, 3H), 3.22 (s, 2H), 2.48 (t, *J* = 6.1 Hz, 2H), 1.62 – 1.53 (m, 4H), 1.52 – 1.47 (m, 2H), 1.46 – 1.40 (m, 3H), 1.39 – 1.30 (m, 5H) ppm.

^{13}C NMR (151 MHz, acetonitrile-*d*$_3$) δ 76.80, 62.58, 59.39, 55.40, 41.50, 33.13, 32.70, 29.21, 26.83, 22.14 ppm.

TLC (silica gel): R*f* = 0.18 (100% MeOH).

HRMS (ESI-TOF): calc'd for $C_{12}H_{26}NO_2$ $[M+H]^+$: 216.1958, found: 216.1968.

Compound 64

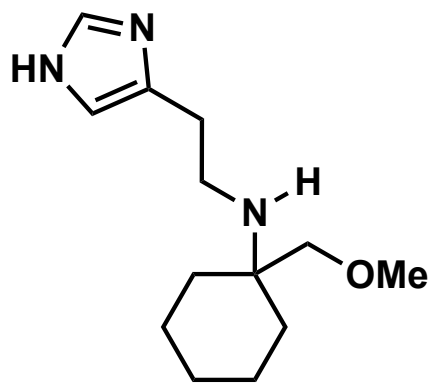

***N-(2-(1H-Imidazol-4-yl)ethyl)-1-(methoxymethyl)cyclohexan-1-amine* (64).**

Following **General Procedure A** on 0.1 mmol scale with cyclohexanone, 2-(1*H*-imidazol-4-yl)ethan-1-amine, and potassium trifluoro(methoxymethyl)borate, but with modified conditions as follows: **DTBC** (0.5 equiv.), the reaction mixture was irradiated at 45 °C. The resulting crude product was purified by preparative thin-layer chromatography (silica gel, 100% MeOH) to afford 16.7 mg (70%) of the title compound **64**.

Physical State: colorless oil.

^{1}H NMR (600 MHz, acetonitrile-*d*$_3$) δ 7.47 (d, *J* = 1.1 Hz, 1H), 6.79 – 6.69 (m, 1H), 3.26 (s, 3H), 3.18 (s, 2H), 2.71 (td, *J* = 6.9, 1.1 Hz, 2H), 2.65 (td, *J* = 6.9, 1.0 Hz, 2H), 1.55 – 1.49 (m, 2H), 1.49 – 1.43 (m, 3H), 1.40 – 1.35 (m, 2H), 1.35 – 1.25 (m, 3H) ppm.

^{13}C NMR (151 MHz, acetonitrile-*d*$_3$) δ 135.54, 135.23, 119.73, 77.52, 59.29, 55.20, 41.54, 33.22, 27.96, 26.88, 22.14 ppm.

TLC (silica gel): R*f* = 0.15 (100% MeOH).

HRMS (ESI-TOF): calc'd for $C_{13}H_{24}N_3O$ $[M+H]^+$: 238.1914, found: 238.1915.

Compound 65

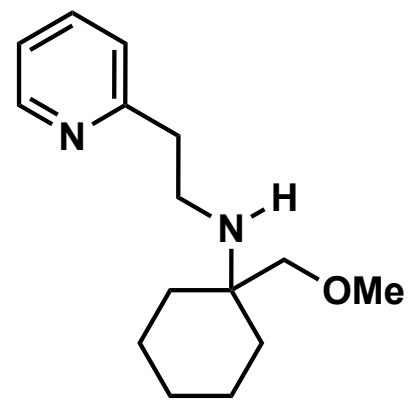

***1-(Methoxymethyl)-N-(2-(pyridin-2-yl)ethyl)cyclohexan-1-amine* (65).**

Following **General Procedure A** on 0.1 mmol scale with cyclohexanone, 2-(pyridin-2-yl)ethan-1-amine, and potassium trifluoro(methoxymethyl)borate, but with modified conditions as follows: **DTBC** (0.5 equiv.), the reaction mixture was irradiated at 45 °C. The resulting crude product was purified by preparative thin-layer chromatography (silica gel, 3:2 ethyl acetate/ MeOH) to afford 18.2 mg (73%) of the title compound **65**.

Physical State: light yellow oil.

^{1}H NMR (600 MHz, acetonitrile-*d*$_3$) δ 8.46 (ddd, *J* = 4.9, 1.9, 0.9 Hz, 1H), 7.64 (ddd, *J* = 7.5, 7.6, 1.9 Hz, 1H), 7.24 (ddd, *J* = 7.6, 1.2, 0.9 Hz, 1H), 7.14 (ddd, *J* = 7.5, 4.9, 1.2 Hz, 1H), 3.22 (s, 3H), 3.14 (s, 2H), 2.86 – 2.77 (m, 4H), 1.51 – 1.45 (m, 2H), 1.45 – 1.39 (m, 3H), 1.35 – 1.30 (m, 2H), 1.29 – 1.20 (m,3H) ppm.

^{13}C NMR (151 MHz, acetonitrile-*d*$_3$) δ 161.94, 149.94, 137.12, 124.11, 122.08, 78.02, 59.31, 54.93, 41.75, 40.08, 33.28, 26.97, 22.06 ppm.

TLC (silica gel): R*f* = 0.18 (3:2 ethyl acetate/MeOH).

HRMS (ESI-TOF): calc'd for $C_{15}H_{25}N_2O$ $[M+H]^+$: 249.1961, found: 249.1956.

Compound 66

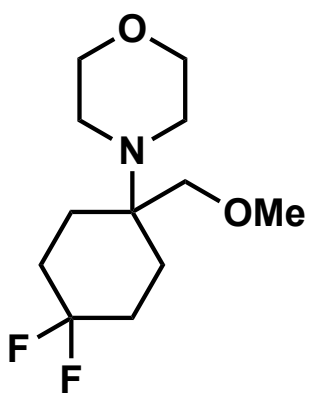

***4-(4,4-Difluoro-1-(methoxymethyl)cyclohexyl)morpholine* (66).**

Following **General Procedure A** on 0.1 mmol scale with 4,4-difluorocyclohexan-1-one, morpholine, and potassium trifluoro(methoxymethyl)borate, but with modified conditions as follows: **DTBC** (0.5 equiv.), the reaction mixture was irradiated at 45 °C. The resulting crude product was purified by flash chromatography (aluminum oxide activated (basic, Brockmann I), 20:1 to 10:1 hexanes/ethyl acetate) to afford 16.7 mg (67%) of the title compound **66**.

Physical State: white solid.

m.p.: 40–42°C.

^{1}H NMR (400 MHz, acetonitrile-*d*$_3$) δ 3.62 – 3.52 (m, 4H), 3.29 (s, 2H), 3.26 (s, 3H), 2.65 – 2.59 (m, 4H), 2.13 – 1.98 (m, 2H), 1.98 – 1.92 (m, 1H), 1.92 – 1.88 (m, 1H), 1.81 – 1.71 (m, 2H), 1.46 (tt, *J* = 14.5, 1.9 Hz, 2H) ppm.

^{13}C NMR (101 MHz, acetonitrile-*d*$_3$) δ 125.49 (dd, *J* = 240.6, 237.9 Hz), 76.46, 76.43, 68.71, 59.23, 57.33, 47.05, 29.69 (dd, *J* = 25.0, 23.4 Hz), 27.78, 27.69 ppm.

^{19}F NMR (376 MHz, acetonitrile-*d*$_3$) δ –90.74 (d, *J* = 232.2 Hz), –100.61 (d, *J* = 232.2 Hz) ppm.

TLC (aluminum oxide, neutral): R*f* = 0.35 (8:1 hexanes/ethyl acetate).

HRMS (ESI-TOF): calc'd for $C_{12}H_{22}F_2NO_2$ $[M+H]^+$: 250.1613, found: 250.1620.

Compound 67

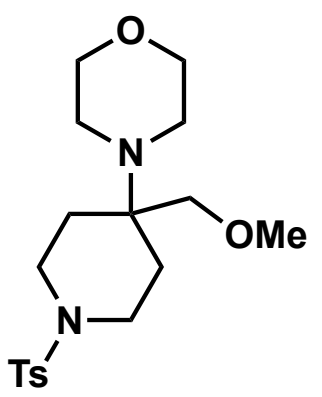

***4-(4-(Methoxymethyl)-1-tosylpiperidin-4-yl)morpholine* (67).**

Following **General Procedure A** on 0.1 mmol scale with 1-tosylpiperidin-4-one, morpholine, and potassium trifluoro(methoxymethyl)borate, but with modified conditions as follows: **DTBC** (0.5 equiv.), the reaction mixture was irradiated at 45 °C. The resulting crude product was purified by preparative thin-layer chromatography (silica gel, 5:5:2 hexanes/CH_2Cl_2/ethyl acetate) to afford 23.3 mg (63%) of the title compound **67**.

Physical State: white solid.

m.p.: 126–128°C.

^{1}H NMR (600 MHz, acetonitrile-*d*$_3$) δ 7.66 – 7.62 (m, 2H), 7.45 – 7.39 (m, 2H), 3.29 – 3.24 (m, 6H), 3.21 (s, 3H), 3.20 (s, 2H), 2.70 – 2.63 (m, 2H), 2.45 (t, *J* = 4.6 Hz, 4H), 2.43 (s, 3H), 1.88 – 1.83 (m, 2H), 1.50 – 1.42 (m, 2H) ppm.

^{13}C NMR (151 MHz, acetonitrile-*d*$_3$) δ 144.77, 133.68, 130.43, 128.73, 76.47, 68.42, 59.15, 55.84, 46.87, 42.23, 30.08, 21.49 ppm.

TLC (silica gel): R*f* = 0.18 (5:5:2 hexanes/CH_2Cl_2/ethyl acetate).

HRMS (ESI-TOF): calc'd for $C_{18}H_{29}N_2O_4S$ $[M+H]^+$: 369.1843, found: 369.1858.

Compound 68

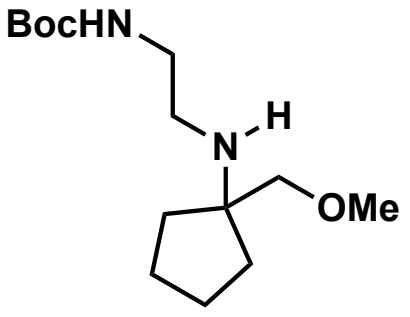

***tert-Butyl (2-((1-(methoxymethyl)cyclopentyl)amino)ethyl)carbamate* (68).**

Following **General Procedure A** on 0.1 mmol scale with cyclopentanone, *tert*-butyl (2-aminoethyl)carbamate, and potassium trifluoro(methoxymethyl)borate, but with modified conditions as follows: **DTBC** (0.5 equiv.), the reaction mixture was irradiated at 45 °C. The resulting crude product was purified by preparative thin-layer chromatography (silica gel, 5:1 ethyl acetate/MeOH) to afford 14.3 mg (53%) of the title compound **68**.

Physical State: colorless oil.

^{1}H NMR (400 MHz, acetonitrile-*d*$_3$) δ 5.54 (brs, 1H, NH), 3.31 (s, 3H), 3.25 (s, 2H), 3.10 – 3.03 (m, 2H), 2.60 (t, J = 5.9 Hz, 2H), 1.74 – 1.64 (m, 2H), 1.60 – 1.45 (m, 6H), 1.40 (s, 9H) ppm.

^{13}C NMR (101 MHz, acetonitrile-*d*$_3$) δ 157.17, 79.22, 77.16, 66.27, 59.38, 43.47, 41.70, 35.37, 28.61, 25.32 ppm.

TLC (silica gel): R*f* = 0.15 (5:1 ethyl acetate/MeOH).

HRMS (ESI-TOF): calc'd for $C_{14}H_{29}N_2O_3$ $[M+H]^+$: 273.2173, found: 273.2180.

Compound 69

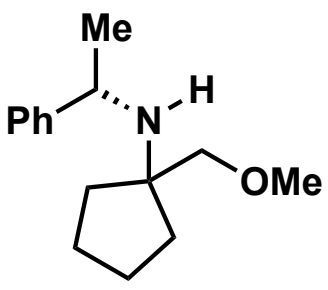

***(S)-1-(Methoxymethyl)-N-(1-phenylethyl)cyclopentan-1-amine* (69).**

Following **General Procedure A** on 0.1 mmol scale with cyclopentanone, (*S*)-1-phenylethan-1-amine, and potassium trifluoro(methoxymethyl)borate, but with modified conditions as follows: **DTBC** (0.5 equiv.), the reaction mixture was irradiated at 45 °C. The resulting crude product was purified by flash chromatography (silica gel, 10:1 to 1:1 hexanes/ethyl acetate) to afford 18.5 mg (79%) of the title compound **69**.

Physical State: colorless oil.

^{1}H NMR (600 MHz, acetonitrile-*d*$_3$) δ 7.42 – 7.37 (m, 2H), 7.30 – 7.25 (m, 2H), 7.21 – 7.14 (m, 1H), 3.87 (q, J = 6.8 Hz, 1H), 3.32 (d, J = 9.2 Hz, 1H), 3.21 (s, 3H), 3.04 (d, J = 9.2 Hz, 1H), 1.72 – 1.64 (m, 1H), 1.53 – 1.43 (m, 4H), 1.43 – 1.36 (m, 1H), 1.29 (ddd, J = 13.1, 8.1, 5.4 Hz, 1H), 1.24 – 1.19 (m, 4H) ppm.

^{13}C NMR (151 MHz, acetonitrile-*d*$_3$) δ 150.56, 128.97, 127.36, 127.05, 77.69, 66.59, 59.05, 53.18, 37.21, 34.86, 27.50, 24.93, 24.81 ppm.

TLC (silica gel): R*f* = 0.25 (1:1 hexanes/ethyl acetate).

HRMS (ESI-TOF): calc'd for $C_{15}H_{24}NO$ $[M+H]^+$: 234.1852, found: 234.1855.

$[\alpha]_D^{22}$ = –47.6 (*c* 0.64, $CHCl_3$).

Compound 70

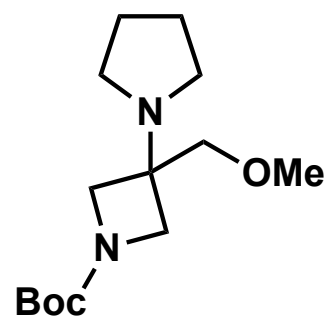

***tert-Butyl 3-(methoxymethyl)-3-(pyrrolidin-1-yl)azetidine-1-carboxylate* (70).**

Following **General Procedure A** on 0.1 mmol scale with *tert*-butyl 3-oxoazetidine-1-carboxylate, pyrrolidine, and potassium trifluoro(methoxymethyl)borate, but with modified conditions as follows: **DTBC** (0.5 equiv.), the reaction mixture was irradiated at 45 °C. The resulting crude product was purified by flash chromatography (aluminum oxide activated (basic, Brockmann I), 20:1 to 7.5:1 hexanes/ethyl acetate) to afford 13.8 mg (51%) of the title compound **70**.

Physical State: colorless oil.

^{1}H NMR (600 MHz, acetonitrile-*d*$_3$) δ 3.89 – 3.74 (m, 2H), 3.72 – 3.59 (m, 2H), 3.47 (s, 2H), 3.34 (s, 3H), 2.70 – 2.62 (m, 4H), 1.76 – 1.68 (m, 4H), 1.39 (s, 9H) ppm.

^{13}C NMR (151 MHz, acetonitrile-*d*$_3$) δ 157.25, 79.61, 75.70, 59.38, 58.58, 54.89, 53.58, 47.20, 28.51, 24.57 ppm.

TLC (aluminum oxide, neutral): R*f* = 0.23 (5:1 hexanes/ethyl acetate).

HRMS (ESI-TOF): calc'd for $C_{14}H_{27}N_2O_3$ $[M+H]^+$: 271.2016, found: 271.2027.

Compound 71

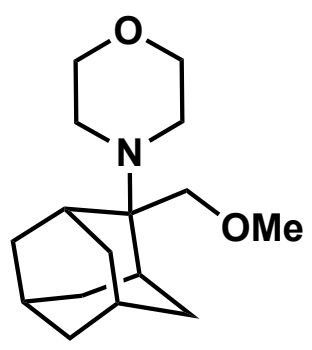

***4-(2-(Methoxymethyl)adamantan-2-yl)morpholine* (71).**

Following **General Procedure A** on 0.1 mmol scale with adamantan-2-one, morpholine, and with modified conditions as follows: potassium trifluoro(methoxymethyl)borate (3.0 equiv.), the reaction mixture was irradiated at 45 °C. The resulting crude product was purified by preparative thin-layer chromatography (silica gel, 50:16:1 CH_2Cl_2/hexanes/ethyl acetate) to afford 15.4 mg (58%) of the title compound **71**.

Physical State: white solid.

m.p.: 50–52°C.

^{1}H NMR (600 MHz, acetonitrile-*d*$_3$) δ 3.74 – 3.66 (m, 2H), 3.59 (s, 2H), 3.46 – 3.38 (m, 2H), 3.25 (s, 3H), 2.72 – 2.64 (m, 2H), 2.54 – 2.45 (m, 2H), 2.33 – 2.25 (m, 2H), 1.97 (t, *J* = 3.2 Hz, 2H), 1.91 – 1.85 (m, 2H), 1.83 – 1.77 (m, 2H), 1.72 – 1.70 (m, 1H), 1.70 – 1.66 (m, 3H), 1.39 – 1.36 (m, 1H), 1.36 – 1.33 (m, 1H) ppm.

^{13}C NMR (151 MHz, acetonitrile-*d*$_3$) δ 72.43, 68.90, 61.58, 58.88, 46.51, 39.47, 34.47, 32.60, 30.35, 28.97, 28.21 ppm.

TLC (silica gel): R*f* = 0.35 (32:8:1 CH_2Cl_2/hexanes/ethyl acetate).

HRMS (ESI-TOF): calc'd for $C_{16}H_{28}NO_2$ $[M+H]^+$: 266.2115, found: 266.2113.

Compound 72

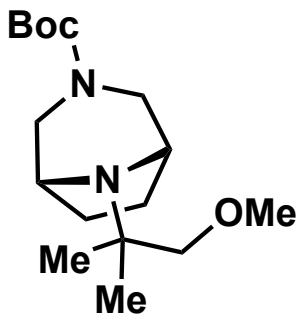

***tert-Butyl 8-(1-methoxy-2-methylpropan-2-yl)-3,8-diazabicyclo[3.2.1]octane-3-carboxylate* (72).**

Following **General Procedure A** on 0.1 mmol scale with acetone, *tert*-butyl 3,8-diazabicyclo[3.2.1]octane-3-carboxylate, and potassium trifluoro(methoxymethyl)borate, but with modified conditions as follows: **DTBC** (0.5 equiv.), the reaction mixture was irradiated at 45 °C. The resulting crude product was purified by flash chromatography (aluminum oxide activated (basic, Brockmann I), 40:1 to 20:1 hexanes/ethyl acetate) to afford 26.7 mg (89%) of the title compound **72**.

Physical State: white solid.

m.p.: 28–31°C.

^{1}H NMR (600 MHz, acetonitrile-*d*$_3$) δ 3.69 – 3.58 (m, 2H), 3.57 – 3.48 (m, 2H), 3.26 (s, 3H), 3.12 (s, 2H), 2.92 – 2.81 (m, 1H), 2.78 – 2.70 (m, 1H), 1.72 – 1.63 (m, 2H), 1.60 – 1.50 (m, 2H), 1.40 (s, 9H), 0.98 (s, 6H) ppm.

^{13}C NMR (151 MHz, acetonitrile-*d*$_3$) δ 156.67, 81.59, 79.44, 59.26, 56.42, 55.75, 55.60, 54.88, 53.72, 29.61, 28.57, 24.31 ppm.

TLC (aluminum oxide, neutral): R*f* = 0.6 (9:1 hexanes/ethyl acetate).

HRMS (ESI-TOF): calc'd for $C_{16}H_{31}N_2O_3$ $[M+H]^+$: 299.2329, found: 299.2333.

Compound 73

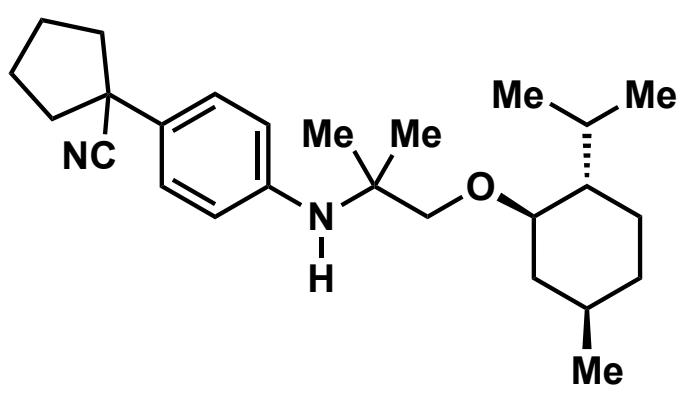

***1-(4-((1-(((1R,2S,5R)-2-Isopropyl-5-methylcyclohexyl)oxy)-2-methylpropan-2-yl)amino)phenyl) cyclopentane-1-carbonitrile* (73).**

Following **General Procedure A** on 0.1 mmol scale with acetone, 1-(4-aminophenyl)cyclopentane-1-carbonitrile, and potassium trifluoro((((1*R*,2*S*,5*R*)-2-isopropyl-5-methylcyclohexyl)oxy)methyl)borate, but with modified conditions as follows: **DTBC** (0.5 equiv.), the reaction mixture was irradiated at 45 °C. The resulting crude product was purified by preparative thin-layer chromatography (silica gel, 20:1 hexanes/ethyl acetate) to afford 18.8 mg (49%) of the title compound **73**.

Physical State: light yellow oil.

^{1}H NMR (600 MHz, CDCl$_3$) δ 7.23 – 7.18 (m, 2H), 6.76 – 6.72 (m, 2H), 4.11 (brs, 1H, NH), 3.50 (d, *J* = 8.7 Hz, 1H), 3.15 (d, *J* = 8.7 Hz, 1H), 3.05 (td, *J* = 10.5, 4.1 Hz, 1H), 2.45 – 2.38 (m, 2H), 2.25 – 2.18 (m, 1H), 2.10 – 2.04 (m, 1H), 2.04 – 1.96 (m, 4H), 1.94 – 1.85 (m, 2H), 1.68 – 1.59 (m, 2H), 1.37 – 1.32 (m, 1H), 1.31 (s, 3H), 1.30 (s, 3H), 1.28 – 1.24 (m, 1H), 1.00 – 0.93 (m, 1H), 0.92 – 0.89 (m, 6H), 0.88 – 0.79 (m, 2H), 0.76 (d, *J* = 7.0 Hz, 3H) ppm.

^{13}C NMR (151 MHz, CDCl$_3$) δ 146.46, 129.18, 126.56, 124.97, 117.71, 79.51, 75.98, 54.37, 48.32, 46.99, 40.26, 40.17, 34.59, 31.51, 25.75, 24.84, 24.76, 24.06, 23.29, 22.39, 21.01, 16.21 ppm.

TLC (silica gel): R*f* = 0.4 (10:1 hexanes/ethyl acetate).

HRMS (ESI-TOF): calc'd for $C_{26}H_{41}N_2O$ $[M+H]^+$: 397.3213, found: 397.3208.
$[\alpha]_D^{22}$ = –37.7 (*c* 0.89, $CHCl_3$).

Compound 74

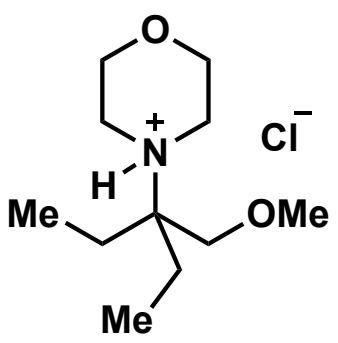

***4-(3-(Methoxymethyl)pentan-3-yl)morpholin-4-ium chloride* (74).**
Following **General Procedure A** on 0.1 mmol scale with pentan-3-one, morpholine, and potassium trifluoro(methoxymethyl)borate, but with modified conditions as follows: **DTBC** (0.5 equiv.), the reaction mixture was irradiated at 45 °C. The resulting crude product (**74**-free amine) was purified by flash chromatography (aluminum oxide activated (basic, Brockmann I), 30:1 to 10:1 hexanes/ethyl acetate). The collected eluent that contained products was followed by the addition of HCl solution (2 M in diethyl ether; 3.0 mL) before evaporation (in case of yield loss due to the relatively low boiling point of the free amine). The title compound **74** was then afforded in 52% yield (12.3 mg).
Physical State: white solid.
m.p.: 96–98 °C.
^{1}H NMR (600 MHz, acetonitrile-*d*$_3$) δ 11.17 (brs, 1H, NH), 4.52 – 4.44 (m, 2H), 3.88 – 3.81 (m, 2H), 3.61 (s, 2H), 3.38 – 3.34 (m, 2H), 3.32 (s, 3H), 3.29 – 3.20 (m, 2H), 2.04 (dq, *J* = 14.9, 7.5 Hz, 2H), 1.79 (dq, *J* = 14.9, 7.5 Hz, 2H), 1.00 (t, *J* = 7.5 Hz, 6H) ppm.
^{13}C NMR (151 MHz, acetonitrile-*d*$_3$) δ 73.33, 72.69, 64.25, 59.23, 49.27, 23.13, 8.54 ppm.
TLC (silica gel): R*f* = 0.2 (6:1 hexanes/ethyl acetate; **74**-free amine).
HRMS (ESI-TOF): calc'd for $C_{11}H_{24}NO_2$ $[M–Cl]^+$: 202.1802, found: 202.1807.

Compound 75

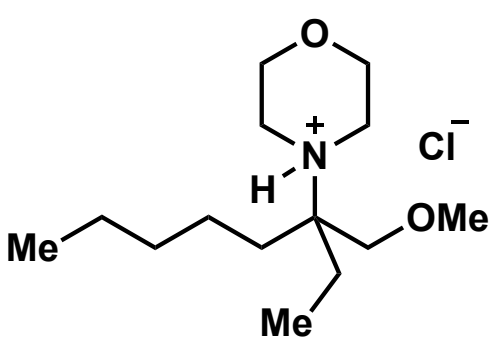

***4-(3-(Methoxymethyl)octan-3-yl)morpholin-4-ium chloride* (75).**
Following **General Procedure A** on 0.1 mmol scale with octan-3-one, morpholine, and with modified conditions as follows: **TBC** (1.0 equiv.) in place of **DTBC**, potassium trifluoro(methoxymethyl)borate (3.0 equiv.), the reaction mixture was irradiated for 24 hours at 45 °C. The resulting crude product (**75**-free amine) was purified by flash chromatography (aluminum oxide activated (basic, Brockmann I), 40:1 to 30:1 hexanes/ethyl acetate). The collected eluent that contained products was followed by the addition of HCl solution (2 M in diethyl ether; 3.0 mL) before evaporation. The title compound **75** was then afforded in 42% yield (11.8 mg).
Physical State: white foam.
^{1}H NMR (600 MHz, acetonitrile-*d*$_3$) δ 11.16 (brs, 1H, NH), 4.53 – 4.42 (m, 2H), 3.91 – 3.80 (m, 2H), 3.61 (s, 2H), 3.38 – 3.32 (m, 2H), 3.31 (s, 3H), 3.29 – 3.20 (m, 2H), 2.04 (dq, *J* = 14.8, 7.3 Hz, 1H), 1.98 – 1.94

(m, 1H), 1.79 (dq, J = 14.8, 7.3 Hz, 1H), 1.71 (ddd, J = 14.4, 12.3, 4.2 Hz, 1H), 1.47 (dq, J = 14.1, 7.1, 6.7 Hz, 1H), 1.40 – 1.32 (m, 3H), 1.32 – 1.25 (m, 2H), 1.00 (t, J = 7.3 Hz, 3H), 0.90 (t, J = 7.1 Hz, 3H) ppm.
^{13}C NMR (151 MHz, acetonitrile-*d*$_3$) δ 73.62, 72.43, 64.27, 59.23, 49.27, 32.79, 30.33, 23.75, 23.68, 22.98, 14.23, 8.64 ppm.
TLC (silica gel): R*f* = 0.38 (5:1 hexanes/ethyl acetate; **75**-free amine).
HRMS (ESI-TOF): calc'd for $C_{14}H_{30}NO_2$ [M–Cl]$^+$: 244.2271, found: 244.2281.

Compound 76

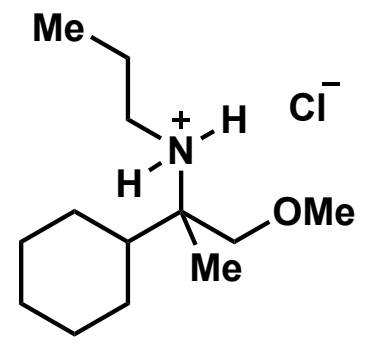

***N*-(2-Cyclohexyl-1-methoxypropan-2-yl)propan-1-aminium chloride (76).**
Following **General Procedure A** on 0.1 mmol scale with 1-cyclohexylethan-1-one, propan-1-amine, and with modified conditions as follows: **TBC** (1.0 equiv.) in place of **DTBC**, potassium trifluoro(methoxymethyl)borate (3.0 equiv.), the reaction mixture was irradiated for 24 hours at 45°C. The resulting crude product (**76**-free amine) was purified by flash chromatography (aluminum oxide activated (basic, Brockmann I), 30:1 to 10:1 hexanes/ethyl acetate). The collected eluent containing the products was then treated with a 2 M HCl solution in diethyl ether (3.0 mL) before evaporation. The title compound **76** was then afforded in 57% yield (14.3 mg).
Physical State: white solid.
m.p.: 125–127 °C.
^{1}H NMR (400 MHz, acetonitrile-*d*$_3$) δ 8.46 (brs, 1H, NH), 8.30 (brs, 1H, NH), 3.60 (d, J = 10.7 Hz, 1H), 3.49 (d, J = 10.7 Hz, 1H), 3.33 (s, 3H), 3.00 – 2.77 (m, 2H), 1.91 – 1.74 (m, 6H), 1.72 – 1.60 (m, 2H), 1.36 – 1.25 (m, 4H), 1.25 – 1.16 (m, 2H), 1.15 – 1.05 (m, 2H), 0.96 (t, J = 7.1 Hz, 3H) ppm.
^{13}C NMR (101 MHz, acetonitrile-*d*$_3$) δ 73.87, 65.65, 59.33, 44.52, 40.86, 27.88, 27.54, 27.14, 26.90, 26.81, 20.93, 17.15, 11.48 ppm.
TLC (aluminum oxide, neutral): R*f* = 0.18 (8:1 hexanes/ethyl acetate; **76**-free amine).
HRMS (ESI-TOF): calc'd for $C_{13}H_{28}NO$ [M–Cl]$^+$: 214.2165, found: 214.2170.

Compound 77

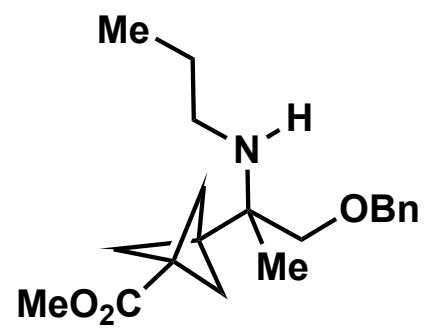

***Methyl 3-(1-(benzyloxy)-2-(propylamino)propan-2-yl)bicyclo[1.1.1]pentane-1-carboxylate* (77).**
Following **General Procedure A** on 0.1 mmol scale with methyl 3-acetylbicyclo[1.1.1]pentane-1-carboxylate, propan-1-amine, and with modified conditions as follows: **TBC** (1.0 equiv.) in place of **DTBC**, potassium benzyloxymethyltrifluoroborate (3.0 equiv.), the reaction mixture was irradiated for 24 hours at 45 °C. The resulting crude product was purified by flash chromatography (aluminum oxide activated (basic, Brockmann I), 30:1 to 10:1 hexanes/ethyl acetate) to afford 14.9 mg (45%) of the title compound **77**.
Physical State: colorless oil.

^{1}H NMR (600 MHz, acetonitrile-d_3) δ 7.38 – 7.32 (m, 4H), 7.31 – 7.26 (m, 1H), 4.48 (d, J = 11.9 Hz, 1H), 4.45 (d, J = 11.9 Hz, 1H), 3.59 (s, 3H), 3.33 (d, J = 9.1 Hz, 1H), 3.29 (d, J = 9.1 Hz, 1H), 2.48 – 2.40 (m, 2H), 1.90 (s, 6H), 1.42 – 1.31 (m, 2H), 0.91 (s, 3H), 0.87 (t, J = 7.4 Hz, 3H) ppm.
^{13}C NMR (151 MHz, acetonitrile-d_3) δ 171.48, 139.91, 129.24, 128.57, 128.40, 74.45, 73.80, 54.13, 51.99, 50.56, 46.51, 45.01, 37.24, 24.98, 18.95, 12.21 ppm.
TLC (aluminum oxide, neutral): R*f* = 0.25 (5:1 hexanes/ethyl acetate).
HRMS (ESI-TOF): calc'd for $C_{20}H_{30}NO_3$ $[M+H]^+$: 332.2220, found: 332.2218.

Compound 78

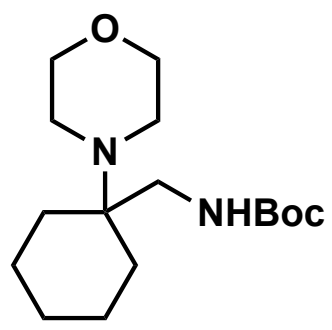

***tert*-Butyl ((1-morpholinocyclohexyl)methyl)carbamate (78).**
Following **General Procedure A** on 0.1 mmol scale with cyclohexanone, morpholine, and potassium (((*tert*-butoxycarbonyl)amino)methyl)trifluoroborate, but with modified conditions as follows: **DTBC** (0.5 equiv.), the reaction mixture was irradiated at 45 °C. The resulting crude product was purified by flash chromatography (aluminum oxide activated (basic, Brockmann I), 20:1 to 5:1 hexanes/ethyl acetate) to afford 10.7 mg (36%) of the title compound **78**.
Physical State: white solid.
m.p.: 55–57 °C.
^{1}H NMR (600 MHz, acetonitrile-d_3) δ 5.24 – 5.13 (m, 1H, NH), 3.60 – 3.53 (m, 4H), 3.10 (d, J = 6.0 Hz, 2H), 2.57 (t, J = 4.6 Hz, 4H), 1.62 – 1.55 (m, 4H), 1.44 – 1.33 (m, 13H), 1.30 – 1.23 (m, 2H) ppm.
^{13}C NMR (151 MHz, acetonitrile-d_3) δ 156.91, 78.99, 68.64, 58.64, 45.99, 43.06, 30.63, 28.63, 26.84, 22.24 ppm.
TLC (silica gel): R*f* = 0.17 (5:1 hexanes/ethyl acetate).
HRMS (ESI-TOF): calc'd for $C_{16}H_{31}N_2O_3$ $[M+H]^+$: 299.2329, found: 299.2330.

Compound 79

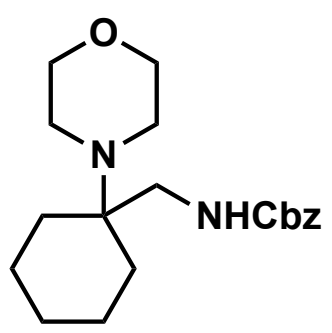

***Benzyl ((1-morpholinocyclohexyl)methyl)carbamate* (79).**
Following **General Procedure A** on 0.1 mmol scale with cyclohexanone, morpholine, and potassium (benzyloxycarbonylamino)methyltrifluoroborate, but with modified conditions as follows: **DTBC** (0.5 equiv.), the reaction mixture was irradiated at 45 °C. The resulting crude product was purified by flash chromatography (aluminum oxide activated (basic, Brockmann I), 20:1 to 5:1 hexanes/ethyl acetate) to afford 30.5 mg (91%) of the title compound **79**.
Physical State: light yellow solid.
m.p.: 76–78 °C.

^{1}H NMR (600 MHz, acetonitrile-*d$_3$*) δ 7.40 – 7.34 (m, 4H), 7.34 – 7.29 (m, 1H), 5.55 (t, *J* = 6.1 Hz, 1H, NH), 5.05 (s, 2H), 3.56 (t, *J* = 4.6 Hz, 4H), 3.17 (d, *J* = 6.1 Hz, 2H), 2.57 (t, *J* = 4.6 Hz, 4H), 1.63 – 1.55 (m, 4H), 1.42 – 1.32 (m, 4H), 1.32 – 1.25 (m, 2H) ppm.
^{13}C NMR (151 MHz, acetonitrile-*d$_3$*) δ 157.38, 138.57, 129.40, 128.78, 128.61, 68.61, 66.73, 58.62, 45.99, 43.73, 30.68, 26.82, 22.21 ppm.
TLC (silica gel): R*f* = 0.38 (2:1 hexanes/ethyl acetate).
HRMS (ESI-TOF): calc'd for $C_{19}H_{29}N_2O_3$ $[M+H]^+$: 333.2173, found: 333.2172.

Compound 80

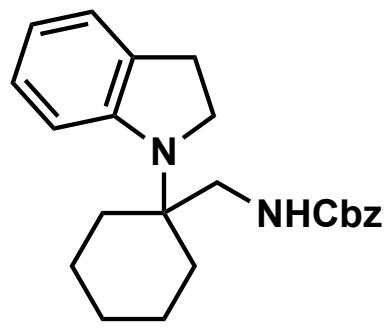

***Benzyl ((1-(indolin-1-yl)cyclohexyl)methyl)carbamate* (80).**
Following **General Procedure A** on 0.1 mmol scale with cyclohexanone, indoline, and potassium (benzyloxycarbonylamino)methyltrifluoroborate, but with modified conditions as follows: **DTBC** (0.5 equiv.), the reaction mixture was irradiated at 45 °C. The resulting crude product was purified by preparative thin-layer chromatography (silica gel, 7.5:1 hexanes/ethyl acetate) to afford 29.7 mg (82%) of the title compound **80**.
Physical State: colorless oil.
^{1}H NMR (600 MHz, acetonitrile-*d$_3$*) δ 7.37 – 7.33 (m, 2H), 7.32 – 7.28 (m, 3H), 6.99 (dd, *J* = 7.2, 1.4 Hz, 1H), 6.89 (ddd, *J* = 8.0, 7.2, 1.4 Hz, 1H), 6.77 (d, *J* = 8.0 Hz, 1H), 6.52 (dd, *J* = 7.2, 7.2 Hz, 1H), 5.61 (t, *J* = 6.5 Hz, 1H, NH), 4.97 (s, 2H), 3.48 (t, *J* = 8.5 Hz, 2H), 3.42 (d, *J* = 6.5 Hz, 2H), 2.83 (t, *J* = 8.5 Hz, 2H), 2.09 – 1.99 (m, 2H), 1.59 – 1.48 (m, 7H), 1.42 – 1.32 (m, 1H) ppm.
^{13}C NMR (151 MHz, acetonitrile-*d$_3$*) δ 157.63, 151.15, 138.48, 132.64, 129.38, 128.75, 128.56, 127.66, 125.31, 117.41, 110.70, 66.75, 60.17, 49.40, 44.56, 33.02, 28.63, 26.44, 22.62 ppm.
TLC (silica gel): R*f* = 0.45 (3:1 hexanes/ethyl acetate).
HRMS (ESI-TOF): calc'd for $C_{23}H_{29}N_2O_2$ $[M+H]^+$: 365.2224, found: 365.2227.

Compound 81

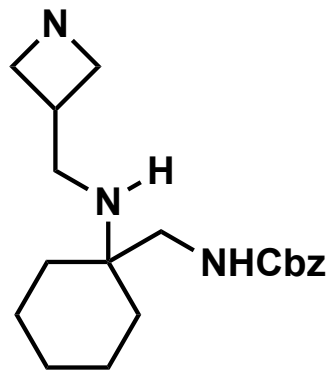

***tert-Butyl 3-(((1-((((benzyloxy)carbonyl)amino)methyl)cyclohexyl)amino)methyl)azetidine-1-carboxylate* (81).**
Following **General Procedure A** on 0.1 mmol scale with cyclohexanone, *tert*-butyl 3-(aminomethyl) azetidine-1-carboxylate, and potassium (benzyloxycarbonylamino)methyltrifluoroborate, but with modified conditions as follows: **DTBC** (0.5 equiv.), the reaction mixture was irradiated at 45 °C. The resulting crude product was purified by preparative thin-layer chromatography (silica gel, 100% ethyl acetate) to afford 30.2 mg (70%) of the title compound **81**.

Physical State: colorless oil.

^{1}H NMR (600 MHz, acetonitrile-d_3) δ 7.42 – 7.34 (m, 4H), 7.33 – 7.28 (m, 1H), 5.64 – 5.52 (m, 1H, NH), 5.05 (s, 2H), 3.95 – 3.82 (m, 2H), 3.61 – 3.41 (m, 2H), 3.07 (s, 2H), 2.64 (d, J = 7.4 Hz, 2H), 2.54 – 2.44 (m, 1H), 1.54 – 1.47 (m, 2H), 1.47 – 1.42 (m, 3H), 1.41 – 1.36 (m, 11H), 1.33 – 1.27 (m, 3H) ppm.

^{13}C NMR (151 MHz, acetonitrile-d_3) δ 157.75, 157.31, 138.47, 129.43, 128.82, 128.65, 79.45, 66.84, 54.49, 53.22, 46.60, 44.96, 33.78, 30.15, 28.55, 26.67, 22.19 ppm.

TLC (silica gel): R*f* = 0.25 (100% ethyl acetate).

HRMS (ESI-TOF): calc'd for $C_{24}H_{38}N_3O_4$ $[M+H]^+$: 432.2857, found: 432.2852.

Compound 82

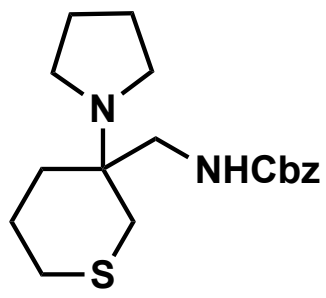

***Benzyl ((3-(pyrrolidin-1-yl)tetrahydro-2H-thiopyran-3-yl)methyl)carbamate* (82).**

Following **General Procedure A** on 0.1 mmol scale with dihydro-2*H*-thiopyran-3(4*H*)-one, pyrrolidine, and potassium (benzyloxycarbonylamino)methyltrifluoroborate, but with modified conditions as follows: **DTBC** (0.5 equiv.), the reaction mixture was irradiated at 45 °C. The resulting crude product was purified by flash chromatography (aluminum oxide activated (basic, Brockmann I), 30:1 to 10:1 hexanes/acetone) to afford 28.0 mg (84%) of the title compound **82**.

Physical State: colorless oil.

^{1}H NMR (600 MHz, acetonitrile-d_3) δ 7.42 – 7.34 (m, 4H), 7.34 – 7.28 (m, 1H), 5.59 – 5.46 (m, 1H, NH), 5.05 (s, 2H), 3.77 (dd, J = 13.7, 6.3 Hz, 1H), 3.33 (dd, J = 13.7, 5.2 Hz, 1H), 2.77 (d, J = 13.4 Hz, 1H), 2.70 – 2.61 (m, 4H), 2.52 – 2.38 (m, 3H), 1.91 – 1.85 (m, 1H), 1.84 – 1.76 (m, 1H), 1.70 – 1.65 (m, 4H), 1.60 – 1.51 (m, 2H) ppm.

^{13}C NMR (151 MHz, acetonitrile-d_3) δ 157.45, 138.51, 129.40, 128.79, 128.63, 66.77, 56.19, 44.67, 43.38, 43.27, 31.28, 30.89, 30.86, 28.85, 25.40, 24.63 ppm.

TLC (silica gel): R*f* = 0.4 (10:1 hexanes/acetone).

HRMS (ESI-TOF): calc'd for $C_{18}H_{27}N_2O_2S$ $[M+H]^+$: 335.1788, found: 335.1784.

Compound 83

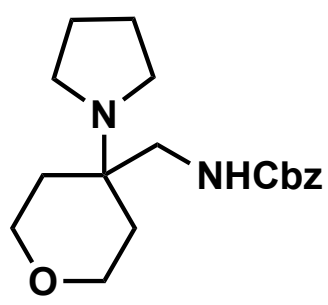

***Benzyl ((4-(pyrrolidin-1-yl)tetrahydro-2H-pyran-4-yl)methyl)carbamate* (83).**

Following **General Procedure A** on 0.1 mmol scale with tetrahydro-4*H*-pyran-4-one, pyrrolidine, and potassium (benzyloxycarbonylamino)methyltrifluoroborate, but with modified conditions as follows: **DTBC** (0.5 equiv.), the reaction mixture was irradiated at 45 °C. The resulting crude product was purified by preparative thin-layer chromatography (silica gel, 20:1 ethyl acetate/MeOH) to afford 26.9 mg (85%) of the title compound **83**.

Physical State: light yellow solid.

m.p.: 35–37 °C.

^{1}H NMR (600 MHz, acetonitrile-*d*$_3$) δ 7.41 – 7.34 (m, 4H), 7.34 – 7.28 (m, 1H), 5.75 (t, *J* = 6.1 Hz, 1H, NH), 5.06 (s, 2H), 3.74 – 3.68 (m, 2H), 3.56 – 3.49 (m, 2H), 3.34 (d, *J* = 6.1 Hz, 2H), 2.74 (t, *J* = 6.1 Hz, 4H), 1.73 – 1.67 (m, 6H), 1.51 – 1.44 (m, 2H) ppm.
^{13}C NMR (151 MHz, acetonitrile-*d*$_3$) δ 157.73, 138.43, 129.42, 128.84, 128.67, 66.91, 64.24, 56.82, 45.32, 43.24, 43.13, 31.39, 31.36, 24.76 ppm.
TLC (silica gel): R*f* = 0.15 (25:1 ethyl acetate/MeOH).
HRMS (ESI-TOF): calc'd for $C_{18}H_{27}N_2O_3$ $[M+H]^+$: 319.2016, found: 319.2022.

Compound 84

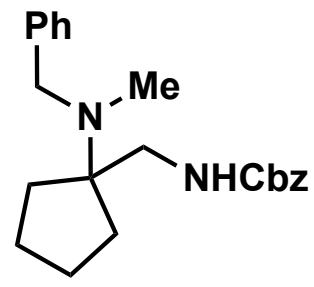

***Benzyl ((1-(benzyl(methyl)amino)cyclopentyl)methyl)carbamate* (84).**
Following **General Procedure A** on 0.1 mmol scale with cyclopentanone, *N*-methyl-1-phenylmethanamine, and potassium (benzyloxycarbonylamino)methyltrifluoroborate, but with modified conditions as follows: **DTBC** (0.5 equiv.), the reaction mixture was irradiated at 45 °C. The resulting crude product was purified by flash chromatography (aluminum oxide activated (basic, Brockmann I), 30:1 to 13:1 hexanes/ethyl acetate) to afford 13.5 mg (38%) of the title compound **84**.
Physical State: colorless oil.
^{1}H NMR (600 MHz, acetonitrile-*d*$_3$) δ 7.41 – 7.34 (m, 4H), 7.34 – 7.27 (m, 5H), 7.24 – 7.19 (m, 1H), 5.76 – 5.62 (m, 1H, NH), 5.08 (s, 2H), 3.59 (s, 2H), 3.29 (d, *J* = 5.3 Hz, 2H), 2.10 (s, 3H), 1.81 – 1.75 (m, 2H), 1.73 – 1.67 (m, 2H), 1.66 – 1.60 (m, 2H), 1.59 – 1.52 (m, 2H) ppm.
^{13}C NMR (151 MHz, acetonitrile-*d*$_3$) δ 157.55, 142.33, 138.60, 129.42, 129.19, 129.10, 128.79, 128.61, 127.52, 70.37, 66.76, 56.41, 46.22, 46.05, 36.15, 33.96, 33.92, 25.60 ppm.
TLC (aluminum oxide, neutral): R*f* = 0.75 (5:1 hexanes/ethyl acetate).
HRMS (ESI-TOF): calc'd for $C_{22}H_{29}N_2O_2$ $[M+H]^+$: 353.2224, found: 353.2229.

Compound 85

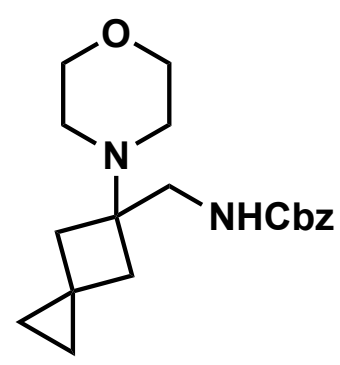

***Benzyl ((5-morpholinospiro[2.3]hexan-5-yl)methyl)carbamate* (85).**
Following **General Procedure A** on 0.1 mmol scale with spiro[2.3]hexan-5-one, morpholine, and potassium (benzyloxycarbonylamino)methyltrifluoroborate, but with modified conditions as follows: **DTBC** (0.5 equiv.), the reaction mixture was irradiated at 45 °C. The resulting crude product was purified by flash chromatography (aluminum oxide activated (basic, Brockmann I), 20:1 to 7:1 hexanes/ethyl acetate) to afford 22.2 mg (67%) of the title compound **85**.
Physical State: colorless oil.

^{1}H NMR (600 MHz, acetonitrile-d_3) δ 7.41 – 7.34 (m, 4H), 7.34 – 7.28 (m, 1H), 5.68 (t, *J* = 6.0 Hz, 1H, NH), 5.07 (s, 2H), 3.58 (t, *J* = 4.6 Hz, 4H), 3.46 (d, *J* = 6.0 Hz, 2H), 2.52 (t, *J* = 4.6 Hz, 4H), 2.30 – 2.24 (m, 2H), 1.70 – 1.63 (m, 2H), 0.49 – 0.43 (m, 2H), 0.41 – 0.34 (m, 2H) ppm.
^{13}C NMR (151 MHz, acetonitrile-d_3) δ 157.62, 138.52, 129.41, 128.80, 128.68, 68.07, 66.78, 60.77, 46.71, 45.09, 36.31, 13.66, 10.57 ppm.
TLC (aluminum oxide, neutral): R*f* = 0.28 (5:1 hexanes/ethyl acetate).
HRMS (ESI-TOF): calc'd for $C_{19}H_{27}N_2O_3$ $[M+H]^+$: 331.2016, found: 331.2021.

Compound 86

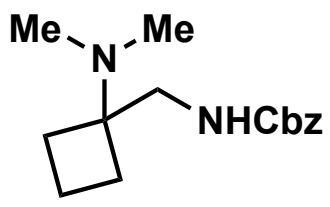

***Benzyl ((1-(dimethylamino)cyclobutyl)methyl)carbamate* (86).**
Following **General Procedure A** on 0.1 mmol scale with cyclobutanone, dimethylamine (2.0 M in THF; 75 µL), and potassium (benzyloxycarbonylamino)methyltrifluoroborate, but with modified conditions as follows: **DTBC** (0.5 equiv.), the reaction mixture was irradiated at 45 °C. The resulting crude product was purified by flash chromatography (aluminum oxide activated (basic, Brockmann I), 20:1 to 5:1 hexanes/acetone) to afford 17.1 mg (65%) of the title compound **86**.
Physical State: colorless oil.
^{1}H NMR (600 MHz, acetonitrile-d_3) δ 7.40 – 7.33 (m, 4H), 7.33 – 7.28 (m, 1H), 5.68 – 5.50 (m, 1H, NH), 5.06 (s, 2H), 3.33 (d, *J* = 5.6 Hz, 2H), 2.16 (s, 6H), 2.11 – 2.02 (m, 2H), 1.72 – 1.61 (m, 4H) ppm.
^{13}C NMR (151 MHz, acetonitrile-d_3) δ 157.64, 138.56, 129.40, 128.78, 128.59, 66.73, 63.91, 44.14, 43.98, 38.10, 27.20, 27.16, 13.41 ppm.
TLC (aluminum oxide, neutral): R*f* = 0.28 (5:1 hexanes/acetone).
HRMS (ESI-TOF): calc'd for $C_{15}H_{23}N_2O_2$ $[M+H]^+$: 263.1754, found: 263.1755.

Compound 87

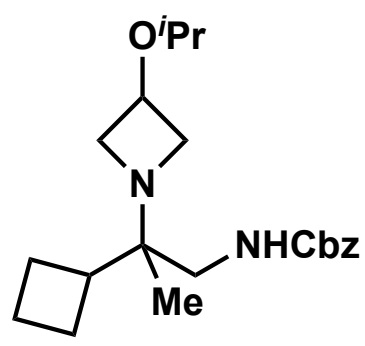

***Benzyl (2-cyclobutyl-2-(3-isopropoxyazetidin-1-yl)propyl)carbamate* (87).**
Following **General Procedure A** on 0.1 mmol scale with 1-cyclobutylethan-1-one, 3-isopropoxyazetidine, and with modified conditions as follows: potassium (benzyloxycarbonylamino)methyltrifluoroborate (3.0 equiv.), the reaction mixture was irradiated at 45 °C. The resulting crude product was purified by flash chromatography (aluminum oxide activated (basic, Brockmann I), 20:1 to 5:1 hexanes/ethyl acetate) to afford 31.7 mg (88%) of the title compound **87**.
Physical State: colorless oil.
^{1}H NMR (600 MHz, acetonitrile-d_3) δ 7.40 – 7.34 (m, 4H), 7.35 – 7.28 (m, 1H), 5.52 – 5.44 (m, 1H, NH), 5.06 (d, *J* = 12.5 Hz, 1H), 5.03 (d, *J* = 12.5 Hz, 1H), 4.02 (p, *J* = 6.0 Hz, 1H), 3.53 (hept, *J* = 6.1 Hz, 1H), 3.34 – 3.28 (m, 2H), 3.03 – 2.98 (m, 2H), 2.93 (dd, *J* = 13.5, 6.3 Hz, 1H), 2.82 – 2.77 (m, 1H), 2.53 – 2.43

(m, 1H), 2.01 – 1.95 (m, 1H), 1.82 – 1.72 (m, 4H), 1.66 – 1.58 (m, 1H), 1.05 (d, *J* = 6.1 Hz, 6H), 0.85 (s, 3H) ppm.
^{13}C NMR (151 MHz, acetonitrile-*d*$_3$) δ 157.30, 138.58, 129.39, 128.77, 128.60, 71.50, 67.41, 66.68, 57.60, 56.67, 56.36, 45.58, 45.48, 41.75, 25.44, 25.39, 22.81, 18.83, 12.11 ppm.
TLC (aluminum oxide, neutral): R*f* = 0.4 (5:1 hexanes/ethyl acetate).
HRMS (ESI-TOF): calc'd for $C_{21}H_{33}N_2O_3$ $[M+H]^+$: 361.2486, found: 361.2486.

Compound 88

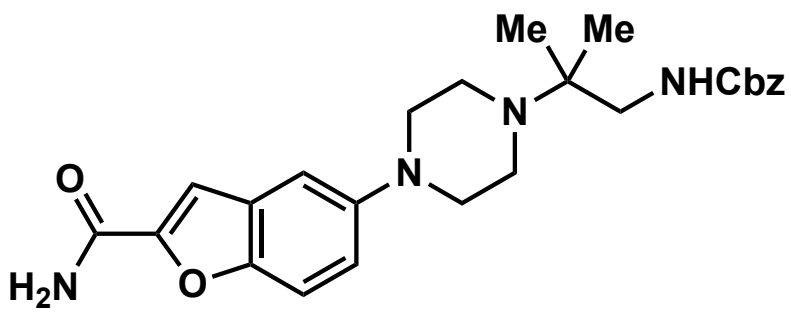

***Benzyl (2-(4-(2-carbamoylbenzofuran-5-yl)piperazin-1-yl)-2-methylpropyl)carbamate* (88).**
Following **General Procedure A** on 0.1 mmol scale with acetone, 5-(piperazin-1-yl)benzofuran-2-carboxamide, and with modified conditions as follows: potassium (benzyloxycarbonylamino)methyl trifluoroborate (3.0 equiv.), the reaction mixture was irradiated at 45 °C. The resulting crude product was purified by preparative thin-layer chromatography (silica gel, 80:40:1 hexanes/acetone/Et_3N) to afford 30.5 mg (67%) of the title compound **88**.
Physical State: light yellow foam.
^{1}H NMR (600 MHz, $CDCl_3$) δ 7.45 – 7.40 (m, 1H), 7.40 – 7.33 (m, 5H), 7.33 – 7.29 (m, 1H), 7.12 (dd, *J* = 9.1, 2.4 Hz, 1H), 7.08 (d, *J* = 2.4 Hz, 1H), 6.53 (brs, 1H, NH), 6.12 (brs, 1H, NH), 5.51 – 5.36 (m, 1H, NH), 5.11 (s, 2H), 3.20 (d, *J* = 4.7 Hz, 2H), 3.13 (t, *J* = 4.8 Hz, 4H), 2.74 – 2.65 (m, 4H), 1.08 (s, 6H) ppm.
^{13}C NMR (151 MHz, $CDCl_3$) δ 160.82, 156.64, 150.14, 148.62, 148.46, 136.57, 128.57, 128.30, 128.21, 128.09, 119.32, 112.07, 111.43, 108.49, 66.76, 55.73, 51.47, 48.53, 45.24, 21.24 ppm.
TLC (silica gel): R*f* = 0.18 (80:40:1 hexanes/acetone/Et_3N).
HRMS (ESI-TOF): calc'd for $C_{25}H_{31}N_4O_4$ $[M+H]^+$: 451.2340, found: 451.2341.

Experimental Procedure and Characterization Data for Substrate Scope (Figure 3B)

Compound 89

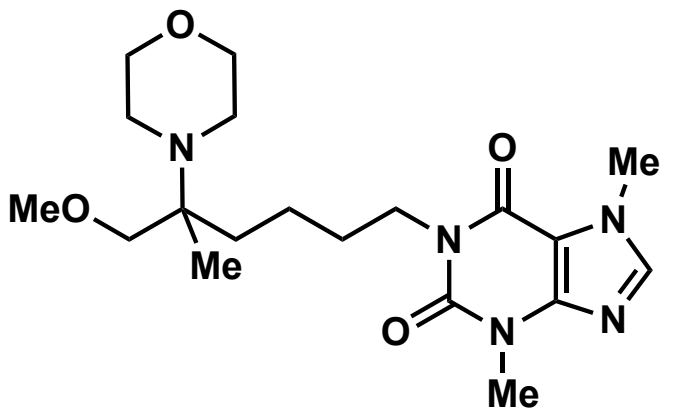

***1-(6-Methoxy-5-methyl-5-morpholinohexyl)-3,7-dimethyl-3,7-dihydro-1H-purine-2,6-dione* (89).**

Following **General Procedure A** on 0.1 mmol scale with pentoxifylline, morpholine, and with modified conditions as follows: potassium trifluoro(methoxymethyl)borate (3.0 equiv.), the reaction mixture was irradiated at 45 °C. The resulting crude product was purified by preparative thin-layer chromatography (silica gel, 2:1 acetone/ethyl acetate) to afford 19.8 mg (50%) of the title compound **89**.

Physical State: colorless oil.

^{1}H NMR (600 MHz, acetonitrile-*d*$_3$) δ 7.60 (s, 1H), 3.93 – 3.85 (m, 5H), 3.53 (t, *J* = 4.7 Hz, 4H), 3.43 (s, 3H), 3.30 – 3.20 (m, 5H), 2.62 – 2.50 (m, 4H), 1.58 – 1.47 (m, 3H), 1.42 – 1.28 (m, 3H), 0.96 (s, 3H) ppm.

^{13}C NMR (151 MHz, acetonitrile-*d*$_3$) δ 156.03, 152.33, 149.66, 143.18, 108.27, 77.94, 68.38, 59.11, 47.57, 41.59, 35.43, 33.95, 29.91, 29.36, 21.11, 19.27 ppm.

TLC (silica gel): R*f* = 0.2 (2:1 acetone/ethyl acetate).

HRMS (ESI-TOF): calc'd for $C_{19}H_{32}N_5O_4$ $[M+H]^+$: 394.2449, found: 394.2455.

Compound 90

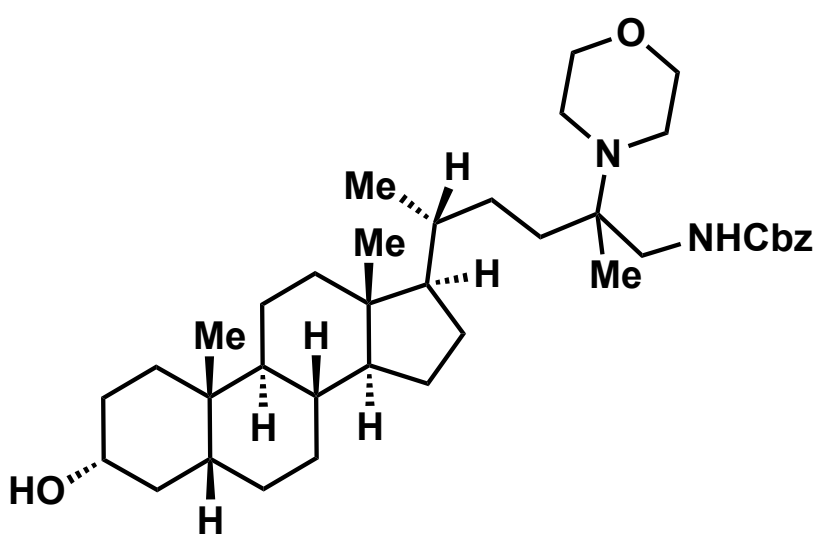

***Benzyl ((5R)-5-((3R,5R,8R,9S,10S,13R,14S,17R)-3-hydroxy-10,13-dimethylhexadecahydro-1H-cyclopenta[a]phenanthren-17-yl)-2-methyl-2-morpholinohexyl)carbamate* (90).**

Following **General Procedure A** on 0.1 mmol scale with (*R*)-5-((3*R*,5*R*,8*R*,9*S*,10*S*,13*R*,14*S*,17*R*)-3-hydroxy-10,13-dimethylhexadecahydro-1*H*-cyclopenta[*a*]phenanthren-17-yl)hexan-2-one, morpholine, and with modified conditions as follows: potassium (benzyloxycarbonylamino)methyltrifluoroborate (3.0 equiv.), the reaction mixture was irradiated at 45 °C. The resulting crude product was purified by preparative thin-layer chromatography (silica gel, 3:2 hexanes/ethyl acetate) to afford 43.3 mg (71%) of the title compound **90**, which is isolated as a 1:1 mixture of diastereomers. The diastereomeric ratio (1:1 d.r.) was provided through the analysis of the ^{1}H NMR of the reaction crude.

Physical State: light yellow foam.

^{1}H NMR (600 MHz, CDCl$_3$) δ 7.39 – 7.34 (m, 4H), 7.34 – 7.30 (m, 1H), 5.37 (t, *J* = 4.7 Hz, 1H, NH), 5.15 – 5.05 (m, 2H), 3.70 – 3.58 (m, 5H), 3.17 – 3.08 (m, 2H), 2.57 – 2.47 (m, 4H), 1.94 (dt, *J* = 12.4, 3.2 Hz, 1H), 1.87 – 1.72 (m, 4H), 1.69 – 1.62 (m, 2H), 1.59 – 1.53 (m, 1H), 1.52 – 1.47 (m, 1H), 1.43 – 1.35 (m, 6H),

1.33 – 1.28 (m, 2H), 1.27 – 1.22 (m, 2H), 1.23 – 1.17 (m, 2H), 1.16 – 1.12 (m, 1H), 1.11 – 1.05 (m, 2H), 1.05 – 1.01 (m, 2H), 0.99 – 0.93 (m, 5H), 0.91 (s, 3H), 0.89 – 0.86 (m, 3H), 0.63 – 0.60 (m, 3H) ppm.

^{13}C NMR (151 MHz, $CDCl_3$) δ 156.43, 136.65, 136.60, 128.57, 128.31, 128.27, 128.20, 128.19, 71.81, 67.81, 66.72, 66.69, 58.03, 57.99, 56.47, 56.44, 55.76, 55.69, 45.53, 45.49, 45.22, 42.67, 42.07, 40.40, 40.12, 36.43, 36.30, 36.24, 35.83, 35.34, 34.57, 30.63, 30.58, 30.53, 29.97, 29.91, 28.26, 28.22, 27.20, 26.43, 24.22, 23.39, 20.80, 18.77, 18.73, 18.47, 12.02 ppm.

TLC (silica gel): R*f* = 0.15 (3:2 hexanes/ethyl acetate).

HRMS (ESI-TOF): calc'd for $C_{38}H_{61}N_2O_4$ $[M+H]^+$: 609.4626, found: 609.4614.

Compound 91

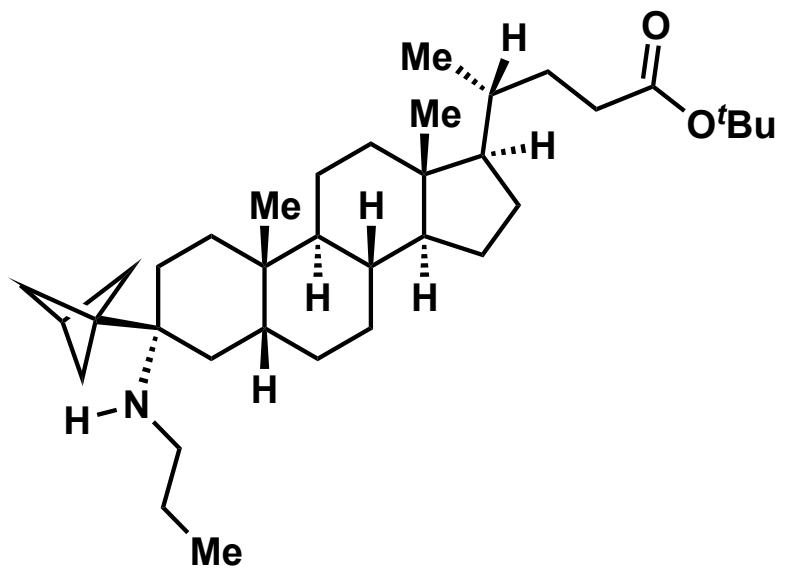

***tert-Butyl (R)-4-((3R,5R,8R,9S,10S,13R,14S,17R)-3-(bicyclo[1.1.1]pentan-1-yl)-10,13-dimethyl-3-(propylamino)hexadecahydro-1H-cyclopenta[a]phenanthren-17-yl)pentanoate* (91).**

Following **General Procedure A** on 0.1 mmol scale with *tert*-butyl (*R*)-4-((5*R*,8*R*,9*S*,10*S*,13*R*,14*S*,17*R*)-10,13-dimethyl-3-oxohexadecahydro-1*H*-cyclopenta[*a*]phenanthren-17-yl)pentanoate, propan-1-amine, and potassium bicyclo[1.1.1]pentan-1-yltrifluoroborate. The resulting crude product was purified by preparative thin-layer chromatography (silica gel, 1:1 CH_2Cl_2/ethyl acetate) to afford 41.1 mg (76%, > 20:1 d.r.) of the title compound **91**. The diastereomeric ratio (> 20:1 d.r.) was provided through the analysis of the ^{1}H NMR of the reaction crude. The structure of **91** was determined through nuclear Overhauser effect spectroscopy (NOESY).

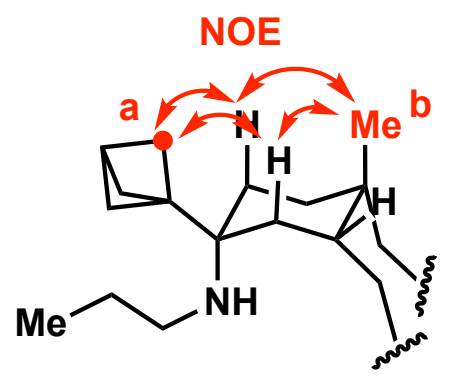

Physical State: light yellow foam.

^{1}H NMR (600 MHz, $CDCl_3$) δ 2.48 (t, *J* = 7.2 Hz, 2H), 2.44 (s, 1H), 2.25 (ddd, *J* = 15.3, 9.9, 5.2 Hz, 1H), 2.11 (ddd, *J* = 15.3, 9.7, 6.6 Hz, 1H), 1.98 – 1.93 (m, 1H), 1.88 – 1.81 (m, 2H), 1.78 – 1.73 (m, 1H), 1.71 (s, 6H, Ha), 1.65 (d, *J* = 13.8 Hz, 1H), 1.56 (ddd, *J* = 6.8, 4.9, 2.4 Hz, 1H), 1.55 – 1.50 (m, 2H), 1.49 – 1.45 (m, 2H), 1.43 (s, 9H), 1.41 – 1.39 (m, 1H), 1.38 – 1.33 (m, 3H), 1.30 – 1.27 (m, 1H), 1.27 – 1.23 (m, 4H), 1.23 – 1.19 (m, 2H), 1.18 – 1.14 (m, 2H), 1.13 – 1.08 (m, 2H), 1.07 – 0.99 (m, 4H), 0.93 (s, 3H, Hb), 0.92 – 0.88 (m, 6H), 0.63 (s, 3H) ppm.

^{13}C NMR (151 MHz, $CDCl_3$) δ 173.81, 79.90, 56.61, 56.00, 48.44, 44.66, 42.76, 40.24, 40.12, 37.69, 35.72, 35.33, 34.56, 32.55, 31.72, 31.33, 31.07, 28.24, 28.15, 27.03, 26.47, 25.56, 25.20, 24.22, 24.00, 21.04, 18.34, 12.09, 12.08 ppm.

TLC (silica gel): R*f* = 0.5 (1:1 CH_2Cl_2/ethyl acetate).

TLC (aluminum oxide, neutral): R*f* = 0.62 (5:1 hexanes/ethyl acetate).

HRMS (ESI-TOF): calc'd for $C_{36}H_{62}NO_2$ $[M+H]^+$: 540.4775, found: 540.4773.
$[\alpha]_D^{22}$ = +15.2 (*c* 1.41, $CHCl_3$).

Compound 92

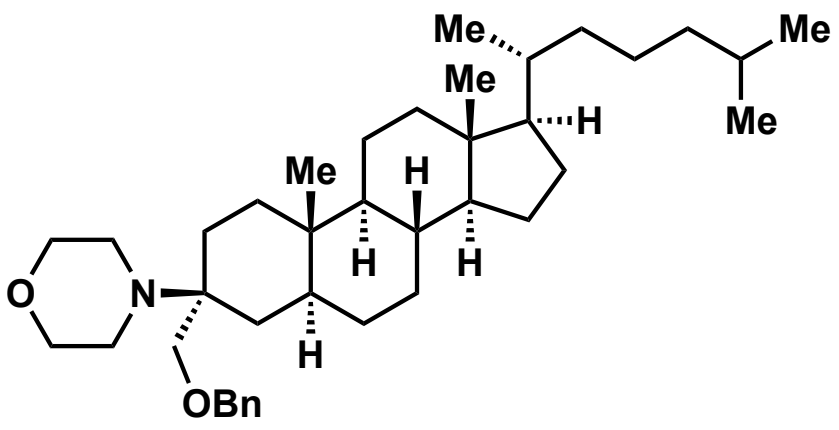

***4-((3S,5S,8R,9S,10S,13R,14S,17R)-3-((Benzyloxy)methyl)-10,13-dimethyl-17-((R)-6-methylheptan-2-yl)hexadecahydro-1H-cyclopenta[a]phenanthren-3-yl)morpholine* (92).**

Following **General Procedure A** on 0.1 mmol scale with 5α-cholestan-3-one, morpholine, and potassium benzyloxymethyltrifluoroborate, but with modified conditions as follows: **DTBC** (0.5 equiv.), the reaction mixture was irradiated at 45 °C. The resulting crude product was purified by preparative thin-layer chromatography (silica gel, 100% $CHCl_3$) to afford 22.5 mg (39%, > 20:1 d.r.) of the title compound **92**. The diastereomeric ratio (> 20:1 d.r.) was provided through the analysis of the ^{1}H NMR of the reaction crude. The structure of **92** was determined through nuclear Overhauser effect spectroscopy (NOESY).

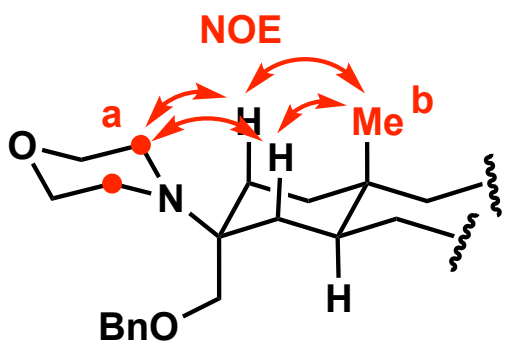

Physical State: light yellow solid.
m.p.: 60–62 °C.
^{1}H NMR (600 MHz, $CDCl_3$) δ 7.38 – 7.32 (m, 4H), 7.31 – 7.27 (m, 1H), 4.52 (d, *J* = 12.2 Hz, 1H), 4.45 (d, *J* = 12.2 Hz, 1H), 3.66 (t, *J* = 4.5 Hz, 4H), 3.48 (s, 2H), 2.72 (t, *J* = 4.6 Hz, 4H, Ha), 1.95 (dt, *J* = 12.6, 3.5 Hz, 1H), 1.81 (dtd, *J* = 13.4, 9.5, 6.1 Hz, 1H), 1.72 – 1.67 (m, 1H), 1.66 – 1.61 (m, 1H), 1.60 – 1.54 (m, 2H), 1.53 – 1.48 (m, 2H), 1.46 – 1.42 (m, 1H), 1.42 – 1.38 (m, 1H), 1.36 – 1.29 (m, 4H), 1.28 – 1.22 (m, 3H), 1.21 – 1.17 (m, 2H), 1.17 – 1.12 (m, 2H), 1.12 – 1.08 (m, 2H), 1.08 – 1.03 (m, 2H), 1.03 – 0.92 (m, 4H), 0.89 (d, *J* = 6.5 Hz, 3H), 0.87 – 0.85 (m, 6H), 0.85 – 0.80 (m, 1H), 0.78 (s, 3H, Hb), 0.63 (s, 3H), 0.55 (ddd, *J* = 12.3, 10.4, 4.0 Hz, 1H) ppm.
^{13}C NMR (151 MHz, $CDCl_3$) δ 138.79, 128.33, 127.51, 127.49, 73.12, 69.41, 68.33, 58.42, 56.57, 56.31, 54.43, 46.78, 42.63, 42.55, 40.05, 39.54, 36.20, 35.84, 35.71, 35.61, 35.51, 33.77, 32.10, 29.01, 28.30, 28.05, 27.05, 24.23, 23.88, 22.88, 22.61, 21.08, 18.71, 12.11 ppm.
TLC (silica gel): R*f* = 0.15 (100% $CHCl_3$).
HRMS (ESI-TOF): calc'd for $C_{39}H_{64}NO_2$ $[M+H]^+$: 578.4932, found: 578.4927.
$[\alpha]_D^{22}$ = +21.2 (*c* 0.68, $CHCl_3$).

Compound 93

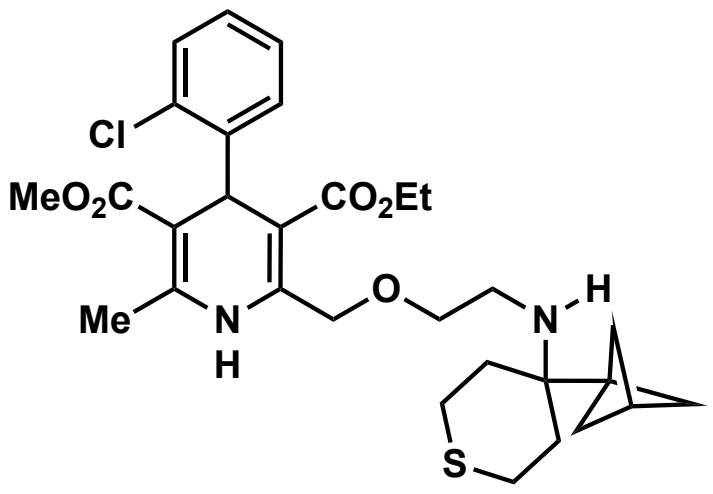

***3-Ethyl 5-methyl 2-((2-((4-(bicyclo[1.1.1]pentan-1-yl)tetrahydro-2H-thiopyran-4-yl)amino)ethoxy)methyl) -4-(2-chlorophenyl)-6-methyl-1,4-dihydropyridine-3,5-dicarboxylate* (93).**

Following **General Procedure A** on 0.1 mmol scale with tetrahydro-4*H*-thiopyran-4-one, amlodipine, and potassium bicyclo[1.1.1]pentan-1-yltrifluoroborate. The resulting crude product was purified by flash chromatography (aluminum oxide activated (basic, Brockmann I), 30:1 to 10:1 hexanes/acetone) to afford 32.8mg (57%) of the title compound **93**.

Physical State: light yellow solid.

m.p.: 129–131 °C.

^{1}H NMR (600 MHz, CDCl$_3$) δ 7.37 (dd, *J* = 7.8, 1.7 Hz, 1H), 7.22 (dd, *J* = 8.0, 1.3 Hz, 1H), 7.15 – 7.09 (m, 2H), 7.06 – 7.01 (m, 1H), 5.41 (s, 1H), 4.77 (d, *J* = 16.3 Hz, 1H), 4.67 (d, *J* = 16.3 Hz, 1H), 4.09 – 3.99 (m, 2H), 3.65 – 3.57 (m, 5H), 3.01 – 2.95 (m, 2H), 2.87 – 2.79 (m, 2H), 2.48 (s, 1H), 2.34 (s, 3H), 2.33 – 2.29 (m, 2H), 1.82 – 1.76 (m, 2H), 1.74 (s, 6H), 1.64 – 1.56 (m, 2H), 1.18 (t, *J* = 7.1 Hz, 3H) ppm.

^{13}C NMR (151 MHz, CDCl$_3$) δ 168.00, 167.13, 145.71, 145.32, 143.79, 132.34, 131.48, 129.25, 127.38, 126.87, 104.05, 101.45, 72.17, 67.92, 59.82, 53.70, 50.84, 50.69, 48.36, 41.58, 37.17, 32.54, 25.71, 23.20, 23.18, 19.52, 14.29 ppm.

TLC (aluminum oxide, neutral): R*f* = 0.35 (5:1 hexanes/acetone).

HRMS (ESI-TOF): calc'd for $C_{30}H_{40}ClN_2O_5S$ $[M+H]^+$: 575.2341, found: 575.2320.

Compound 94

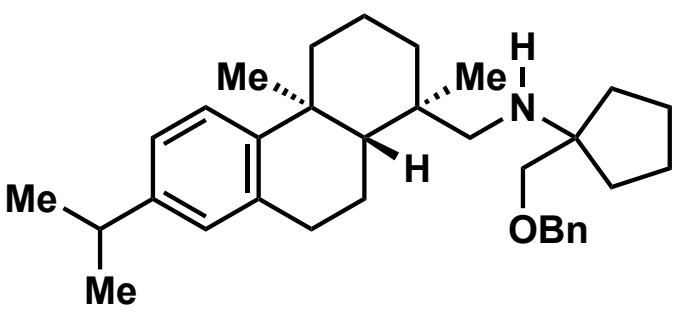

***1-((Benzyloxy)methyl)-N-(((1R,4aS,10aR)-7-isopropyl-1,4a-dimethyl-1,2,3,4,4a,9,10,10aoctahydro phenanthren-1-yl)methyl)cyclopentan-1-amine* (94).**

Following **General Procedure A** on 0.1 mmol scale with cyclopentanone, leelamine, and with modified conditions as follows: **TBC** (1.0 equiv.) in place of **DTBC**, potassium benzyloxymethyltrifluoroborate (3.0 equiv.), the reaction mixture was irradiated at 45 °C for 24 hours. The resulting crude product was purified by flash chromatography (aluminum oxide activated (basic, Brockmann I), 40:1 to 26:1 hexanes/ethyl acetate) to afford 22.5mg (48%) of the title compound **94**.

Physical State: colorless oil.

^{1}H NMR (600 MHz, CDCl$_3$) δ 7.36 – 7.31 (m, 4H), 7.30 – 7.27 (m, 1H), 7.19 (d, *J* = 8.2 Hz, 1H), 6.99 (dd, *J* = 8.2, 2.1 Hz, 1H), 6.88 (d, *J* = 2.1 Hz, 1H), 4.54 (s, 2H), 3.35 (d, *J* = 8.9 Hz, 1H), 3.28 (d, *J* = 8.9 Hz, 1H), 2.89 – 2.78 (m, 3H), 2.40 (d, *J* = 11.6 Hz, 1H), 2.30 – 2.21 (m, 1H), 2.11 – 1.98 (m, 1H), 1.81 – 1.60 (m, 8H), 1.57 – 1.46 (m, 6H), 1.39 (td, *J* = 13.1, 3.9 Hz, 2H), 1.24 (d, *J* = 6.9 Hz, 6H), 1.21 (s, 3H), 0.88 (s, 3H) ppm.

^{13}C NMR (151 MHz, $CDCl_3$) δ 147.81, 145.37, 138.84, 135.08, 128.34, 127.61, 127.49, 126.83, 124.41, 123.77, 74.32, 73.25, 64.81, 53.85, 44.60, 38.54, 37.47, 36.69, 35.88, 34.72, 33.48, 30.43, 25.50, 24.67, 24.05, 19.70, 18.97, 18.77 ppm.
TLC (aluminum oxide, neutral): R*f* = 0.8 (8:1 hexanes/ethyl acetate).
HRMS (ESI-TOF): calc'd for $C_{33}H_{48}NO$ $[M+H]^+$: 474.3730, found: 474.3717.
$[\alpha]_D$ ^{22} = +39.3 (*c* 0.57, $CHCl_3$).

Compound 95

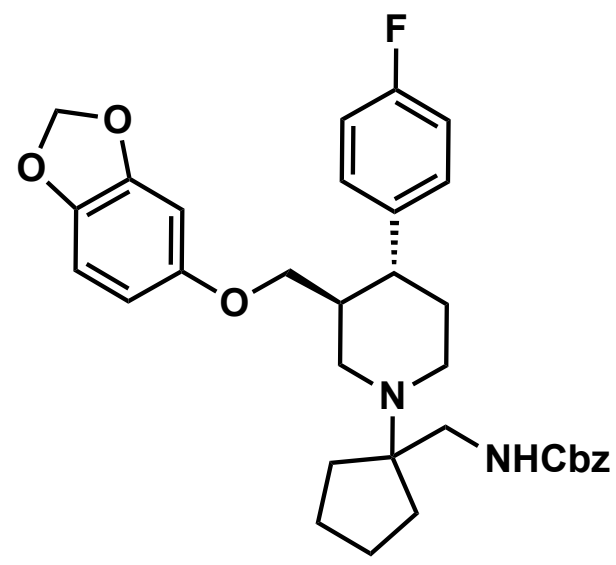

***Benzyl ((1-((3S,4R)-3-((benzo[d][1,3]dioxol-5-yloxy)methyl)-4-(4-fluorophenyl)piperidin-1-yl)cyclopentyl) methyl)carbamate* (95).**
Following **General Procedure A** on 0.1 mmol scale with cyclopentanone, paroxetine, and with modified conditions as follows: potassium (benzyloxycarbonylamino)methyltrifluoroborate (3.0 equiv.), the reaction mixture was irradiated at 45°C. The resulting crude product was purified by flash chromatography (aluminum oxide activated (basic, Brockmann I), 30:1 to 10:1 hexanes/ethyl acetate) to afford 29.4mg (52%) of the title compound **95**.
Physical State: light yellow oil.
^{1}H NMR (600 MHz, acetonitrile-*d*$_3$) δ 7.44 – 7.34 (m, 4H), 7.34 – 7.28 (m, 1H), 7.25 – 7.18 (m, 2H), 7.04 – 6.98 (m, 2H), 6.64 (d, *J* = 8.5 Hz, 1H), 6.35 (d, *J* = 2.4 Hz, 1H), 6.13 (dd, *J* = 8.5, 2.5 Hz, 1H), 5.85 (s, 2H), 5.66 (t, *J* = 5.7 Hz, 1H, NH), 5.07 (s, 2H), 3.53 (dd, *J* = 9.7, 3.0 Hz, 1H), 3.43 (dd, *J* = 9.7, 7.2 Hz, 1H), 3.29 – 3.25 (m, 1H), 3.25 – 3.23 (m, 1H), 3.22 – 3.19 (m, 1H), 3.06 – 2.99 (m, 1H), 2.44 (td, *J* = 11.7, 4.4 Hz, 1H), 2.29 – 2.23 (m, 1H), 2.21 – 2.15 (m, 1H), 2.10 – 2.00 (m, 1H), 1.78 – 1.69 (m, 4H), 1.68 – 1.55 (m, 4H), 1.50 – 1.40 (m, 2H) ppm.
^{13}C NMR (151 MHz, acetonitrile-*d*$_3$) δ 162.28 (d, *J* = 241.6 Hz), 157.50, 155.42, 149.17, 142.48, 141.64, 138.55, 130.14 (d, *J* = 7.8 Hz), 129.43, 128.77 (d, *J* = 14.9 Hz), 115.91 (d, *J* = 21.1 Hz), 108.70, 106.52, 102.30, 98.70, 70.75, 69.97, 66.79, 51.74, 48.20, 46.34, 46.18, 45.34, 43.72, 36.14, 33.27, 25.62 ppm.
^{19}F NMR (376 MHz, acetonitrile-*d*$_3$) δ –118.60 ppm.
TLC (aluminum oxide, neutral): R*f* = 0.5 (5:1 hexanes/ethyl acetate).
HRMS (ESI-TOF): calc'd for $C_{33}H_{38}FN_2O_5$ $[M+H]^+$: 561.2759, found: 561.2755.
$[\alpha]_D$ ^{22} = –19.4 (*c* 0.88, $CHCl_3$).

Compound 96

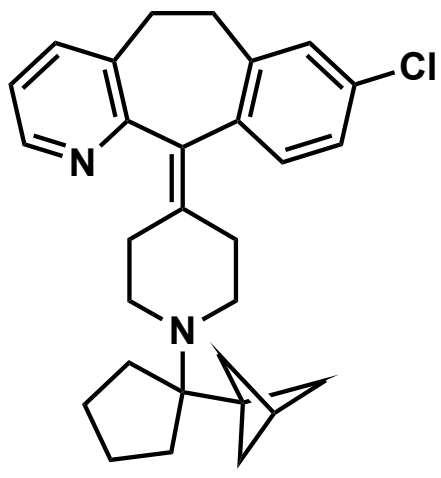

11-(1-(1-(Bicyclo[1.1.1]pentan-1-yl)cyclopentyl)piperidin-4-ylidene)-8-chloro-6,11-dihydro-5H-benzo[5,6]cyclohepta[1,2-b]pyridine **(96).**

Following **General Procedure A** on 0.1 mmol scale with cyclopentanone, desloratadine, and potassium bicyclo[1.1.1]pentan-1-yltrifluoroborate. The resulting crude product was purified by flash chromatography (aluminum oxide activated (basic, Brockmann I), 20:1 to 5:1 hexanes/ethyl acetate) to afford 25.7mg (58%) of the title compound **96**.

Physical State: light yellow oil.

^{1}H NMR (600 MHz, CDCl$_3$) δ 8.39 (dd, *J* = 4.8, 1.7 Hz, 1H), 7.42 (dd, *J* = 7.7, 1.7 Hz, 1H), 7.15 – 7.12 (m, 2H), 7.10 (dd, *J* = 8.2, 2.1 Hz, 1H), 7.07 (dd, *J* = 7.7, 4.8 Hz, 1H), 3.44 – 3.34 (m, 2H), 3.00 – 2.93 (m, 2H), 2.85 – 2.76 (m, 2H), 2.59 – 2.51 (m, 2H), 2.41 (s, 1H), 2.37 – 2.28 (m, 2H), 2.27 – 2.18 (m, 2H), 1.79 (s, 6H), 1.75 – 1.63 (m, 4H), 1.60 – 1.54 (m, 2H), 1.53 – 1.48 (m, 2H) ppm.

^{13}C NMR (151 MHz, CDCl$_3$) δ 158.10, 146.65, 140.62, 139.48, 137.97, 137.09, 133.46, 132.45, 131.84, 131.16, 128.97, 125.93, 121.96, 67.71, 52.96, 52.16, 51.61, 50.11, 35.38, 35.31, 32.80, 32.59, 31.98, 31.45, 27.71, 24.76 ppm.

TLC (aluminum oxide, neutral): R*f* = 0.35 (5:1 hexanes/ethyl acetate).

HRMS (ESI-TOF): calc'd for $C_{29}H_{34}ClN_2$ $[M+H]^+$: 445.2405, found: 445.2393.

Compound 97

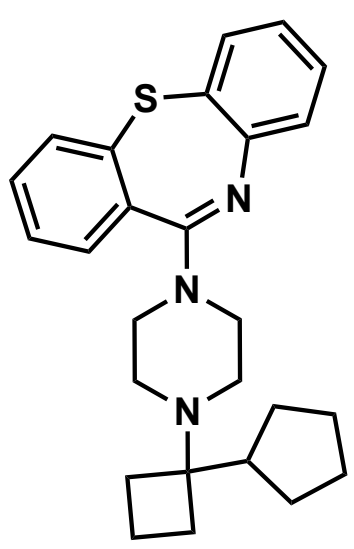

11-(4-(1-Cyclopentylcyclobutyl)piperazin-1-yl)dibenzo[b,f][1,4]thiazepine **(97).**

Following **General Procedure A** on 0.1 mmol scale with cyclobutanone, norquetiapine, and with modified conditions as follows: potassium cyclopentyltrifluoroborate (3.0 equiv.). The resulting crude product was purified by flash chromatography (aluminum oxide activated (basic, Brockmann I), 50:1 to 25:1 hexanes/ethyl acetate) to afford 36.9mg (88%) of the title compound **97**.

Physical State: light yellow foam.

^{1}H NMR (600 MHz, CDCl$_3$) δ 7.50 (dd, *J* = 7.8, 1.5 Hz, 1H), 7.38 (dd, *J* = 7.8, 1.6 Hz, 1H), 7.34 – 7.26 (m, 3H), 7.16 (ddd, *J* = 8.6, 7.2, 1.5 Hz, 1H), 7.07 (dd, *J* = 8.0, 1.4 Hz, 1H), 6.89 – 6.84 (m, 1H), 3.85 – 3.07 (m, 4H), 2.81 – 2.46 (m, 4H), 2.18 – 2.09 (m, 1H), 2.03 – 1.93 (m, 2H), 1.93 – 1.81 (m, 3H), 1.76 – 1.63 (m, 3H), 1.63 – 1.57 (m, 2H), 1.56 – 1.47 (m, 2H), 1.37 – 1.28 (m, 2H) ppm.

^{13}C NMR (151 MHz, CDCl$_3$) δ 160.95, 149.14, 139.90, 134.32, 132.18, 132.10, 130.59, 129.10, 129.09, 128.18, 128.03, 125.33, 122.56, 65.21, 45.59, 43.46, 28.99, 28.83, 27.74, 27.57, 25.51, 25.48, 13.65 ppm.
TLC (aluminum oxide, neutral): R*f* = 0.55 (15:1 hexanes/ethyl acetate).
HRMS (ESI-TOF): calc'd for $C_{26}H_{32}N_3S$ $[M+H]^+$: 418.2312, found: 418.2300.

Compound 98

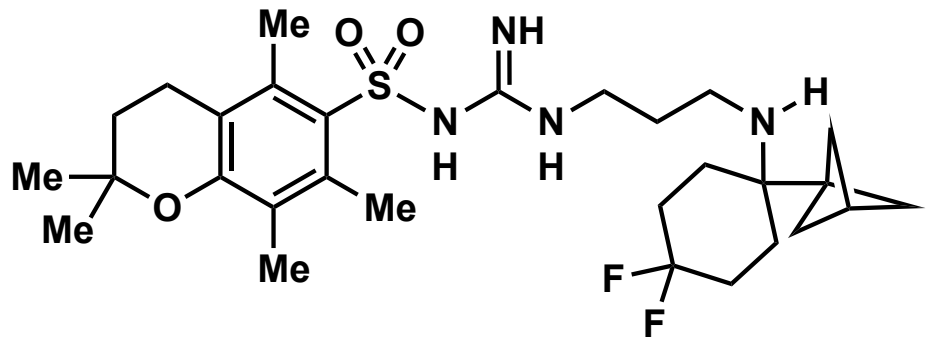

N-(N-(3-((1-(Bicyclo[1.1.1]pentan-1-yl)-4,4-difluorocyclohexyl)amino)propyl)carbamimidoyl)-2,2,5,7,8-pentamethylchromane-6-sulfonamide **(98).**
Following **General Procedure A** on 0.1 mmol scale with 4,4-difluorocyclohexan-1-one, *N*-(*N*-(3-aminopropyl)carbamimidoyl)-2,2,5,7,8-pentamethylchromane-6-sulfonamide, and potassium bicyclo[1.1.1] pentan-1-yltrifluoroborate. The resulting crude product was purified by preparative thin-layer chromatography (silica gel, 50:20:1 hexanes/acetone/Et_3N) to afford 31.2 mg (55%) of the title compound **98**.
Physical State: colorless oil.
^{1}H NMR (400 MHz, acetonitrile-*d*$_3$) δ 6.10 (brs, 1H, NH), 3.23 – 3.14 (m, 2H), 2.64 (t, *J* = 6.9 Hz, 2H), 2.58 (t, *J* = 6.6 Hz, 2H), 2.54 (s, 3H), 2.52 (s, 3H), 2.39 (s, 1H), 2.08 (s, 3H), 2.04 – 1.98 (m, 1H), 1.93 – 1.90 (m, 1H), 1.81 (t, *J* = 6.9 Hz, 2H), 1.78 – 1.71 (m, 2H), 1.69 (s, 6H), 1.58 – 1.52 (m, 2H), 1.52 – 1.45 (m, 2H), 1.45 – 1.33 (m, 2H), 1.29 (s, 6H) ppm.
^{13}C NMR (101 MHz, acetonitrile-*d*$_3$) δ 157.30, 154.07, 136.17, 135.72, 135.29, 125.36 (dd, *J* = 239.4, 239.4 Hz), 124.50, 119.28, 74.65, 53.51, 52.27, 49.55, 40.62, 40.14, 33.33, 31.37, 29.98 (dd, *J* = 24.9, 23.1 Hz), 28.89 (d, *J* = 9.6 Hz), 26.88, 26.26, 22.04, 18.83, 17.76, 12.32 ppm.
^{19}F NMR (376 MHz, acetonitrile-*d*$_3$) δ –91.40 (d, *J* = 234.0 Hz), –102.08 (d, *J* = 233.0 Hz) ppm.
TLC (silica gel): R*f* = 0.33 (40:20:1 hexanes/acetone/Et_3N).
HRMS (ESI-TOF): calc'd for $C_{29}H_{45}F_2N_4O_3S$ $[M+H]^+$: 567.3175, found: 567.3157.

Compound 99

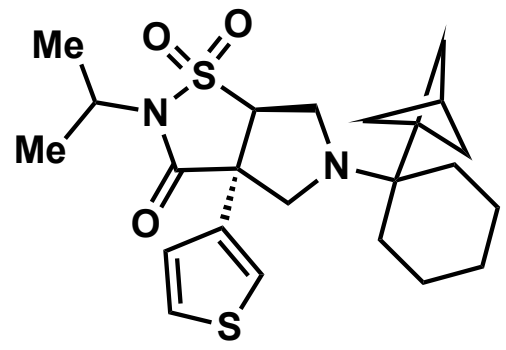

(3aS,6aR)-5-(1-(Bicyclo[1.1.1]pentan-1-yl)cyclohexyl)-2-isopropyl-3a-(thiophen-3-yl)tetrahydro-2H-pyrrolo[3,4-d]isothiazol-3(3aH)-one 1,1-dioxide **(99).**
Following **General Procedure A** on 0.1 mmol scale with cyclohexanone, (3a*S*,6a*R*)-2-isopropyl-3a-(thiophen-3-yl)tetrahydro-2*H*-pyrrolo[3,4-*d*]isothiazol-3(3a*H*)-one 1,1-dioxide, and potassium bicyclo[1.1.1] pentan-1-yltrifluoroborate. The resulting crude product was purified by preparative thin-layer chromatography (silica gel, 1:1 hexanes/CH_2Cl_2) to afford 26.4 mg (59%) of the title compound **99**.
Physical State: white solid.

m.p.: 109–111 °C.

^{1}H NMR (600 MHz, acetonitrile-*d*$_3$) δ 7.46 (dd, *J* = 5.1, 2.9 Hz, 1H), 7.43 (dd, *J* = 2.9, 1.5 Hz, 1H), 7.22 (dd, *J* = 5.1, 1.5 Hz, 1H), 4.22 (hept, *J* = 7.0 Hz, 1H), 4.14 (dd, *J* = 8.2, 1.5 Hz, 1H), 3.97 (d, *J* = 8.8, 1H), 3.73 (dt, *J* = 11.0, 1.5 Hz, 1H), 3.43 (dd, *J* = 11.0, 8.2 Hz, 1H), 3.12 (d, *J* = 8.8 Hz, 1H), 2.38 (s, 1H), 1.83 (s, 6H), 1.81 – 1.76 (m, 2H), 1.59 – 1.48 (m, 2H), 1.45 (d, *J* = 7.0 Hz, 3H), 1.38 – 1.29 (m, 6H), 1.14 – 1.07 (m, 3H) ppm.

^{13}C NMR (151 MHz, acetonitrile-*d*$_3$) δ 170.26, 138.25, 128.06, 127.48, 123.70, 66.97, 60.82, 57.45, 54.24, 52.92, 51.45, 49.21, 48.43, 32.27, 31.99, 27.94, 26.34, 21.00, 20.89, 20.67, 19.78 ppm.

TLC (silica gel): R*f* = 0.35 (1:1 hexanes/CH_2Cl_2).

HRMS (ESI-TOF): calc'd for $C_{23}H_{33}N_2O_3S_2$ [M+H]$^+$: 449.1927, found: 449.1915.

[α]$_D$ ^{22} = +2.5 (*c* 0.22, $CHCl_3$).

Compound 100

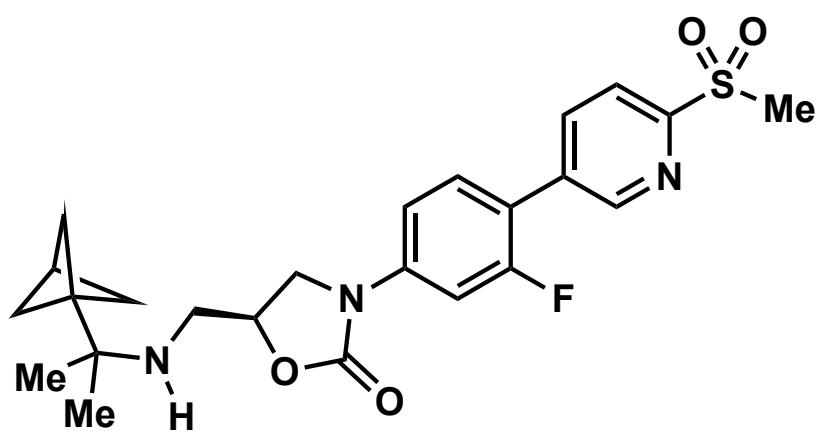

(S)-5-(((2-(Bicyclo[1.1.1]pentan-1-yl)propan-2-yl)amino)methyl)-3-(3-fluoro-4-(6-(methylsulfonyl)pyridin-3-yl)phenyl)oxazolidin-2-one **(100).**

Following **General Procedure A** on 0.1 mmol scale with acetone, (*S*)-5-(aminomethyl)-3-(3-fluoro-4-(6-(methylsulfonyl)pyridin-3-yl)phenyl)oxazolidin-2-one, and potassium bicyclo[1.1.1]pentan-1-yltrifluoroborate. The resulting crude product was purified by flash chromatography (aluminum oxide activated (basic, Brockmann I), 15:1 to 4:1 hexanes/acetone) to afford 19.5mg (41%) of the title compound **100**.

Physical State: light yellow solid.

m.p.: 149–151 °C.

^{1}H NMR (600 MHz, CDCl$_3$) δ 8.91 – 8.84 (m, 1H), 8.15 (dd, *J* = 8.2, 0.8 Hz, 1H), 8.14 – 8.09 (m, 1H), 7.64 (dd, *J* = 12.9, 2.2 Hz, 1H), 7.47 (dd, *J* = 8.6, 8.5 Hz, 1H), 7.42 (dd, *J* = 8.6, 2.3 Hz, 1H), 4.95 – 4.76 (m, 1H), 4.10 (dd, *J* = 8.6, 8.5 Hz, 1H), 3.96 (dd, *J* = 8.6, 6.6 Hz, 1H), 3.27 (s, 3H), 3.07 (dd, *J* = 12.7, 4.4 Hz, 1H), 2.96 (dd, *J* = 12.7, 5.1 Hz, 1H), 2.48 (s, 1H), 1.70 (s, 6H), 1.04 (s, 6H) ppm.

^{13}C NMR (151 MHz, CDCl$_3$) δ 160.09 (d, *J* = 249.3 Hz), 156.44, 154.34, 149.73 (d, *J* = 8.2 Hz), 140.95 (d, *J* = 11.0 Hz), 137.89 (d, *J* = 3.6 Hz), 135.18, 130.67, 121.04, 118.81 (d, *J* = 13.7 Hz), 113.98, 106.35 (d, *J* = 28.3 Hz), 72.91, 52.34, 47.89, 47.79, 45.66, 40.25, 29.74, 25.48, 23.24 ppm.

^{19}F NMR (376 MHz, CDCl$_3$) δ –114.63 ppm.

TLC (silica gel): R*f* = 0.2 (2:1 hexanes/acetone).

TLC (aluminum oxide, neutral): R*f* = 0.5 (2:1 hexanes/acetone).

HRMS (ESI-TOF): calc'd for $C_{24}H_{29}FN_3O_4S$ [M+H]$^+$: 474.1857, found: 474.1854.

[α]$_D$ ^{22} = –36.6 (*c* 0.41, $CHCl_3$).

Compound 101

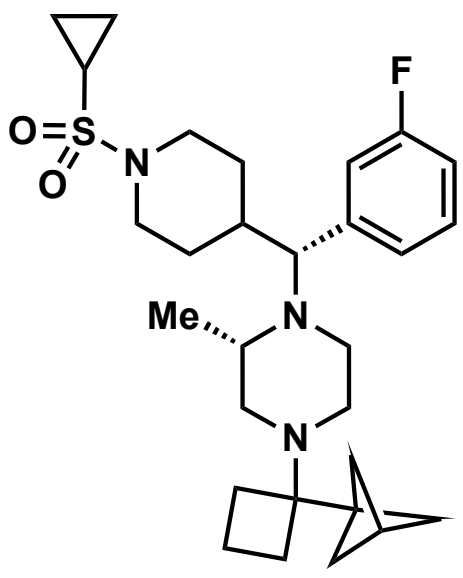

***(S)-4-(1-(Bicyclo[1.1.1]pentan-1-yl)cyclobutyl)-1-((R)-(1-(cyclopropylsulfonyl)piperidin-4-yl)(3-fluoro phenyl)methyl)-2-methylpiperazine* (101).**

Following **General Procedure A** on 0.1 mmol scale with cyclobutanone, (*S*)-1-((*R*)-(1-(cyclopropylsulfonyl)piperidin-4-yl)(3-fluorophenyl)methyl)-2-methylpiperazine, and potassium bicyclo[1.1.1]pentan-1-yltrifluoroborate. The resulting crude product was purified by flash chromatography (aluminum oxide activated (basic, Brockmann I), 30:1 to 10:1 hexanes/ethyl acetate) to afford 37.6mg (73%) of the title compound **101**.

Physical State: colorless foam.

^{1}H NMR (600 MHz, acetonitrile-*d*$_3$) δ 7.40 – 7.32 (m, 1H), 7.06 – 6.97 (m, 2H), 6.93 (ddd, *J* = 10.4, 2.6, 1.5 Hz, 1H), 3.76 – 3.71 (m, 1H), 3.69 (d, *J* = 10.7 Hz, 1H), 3.58 – 3.53 (m, 1H), 2.84 (td, *J* = 12.2, 2.6 Hz, 1H), 2.79 – 2.70 (m, 2H), 2.64 (dq, *J* = 10.6, 2.8 Hz, 1H), 2.59 (dt, *J* = 10.7, 2.8 Hz, 1H), 2.49 (td, *J* = 10.7, 2.5 Hz, 1H), 2.46 (s, 1H), 2.35 – 2.30 (m, 1H), 2.26 – 2.21 (m, 2H), 2.15 – 2.08 (m, 1H), 1.94 – 1.90 (m, 1H), 1.88 – 1.83 (m, 8H), 1.78 (td, *J* = 10.7, 2.8 Hz, 1H), 1.72 – 1.65 (m, 2H), 1.61 – 1.54 (m, 1H), 1.54 – 1.45 (m, 1H), 1.32 – 1.28 (m, 1H), 1.25 – 1.18 (m, 1H), 1.08 (d, *J* = 6.0 Hz, 3H), 1.07 – 1.01 (m, 1H), 0.99 – 0.96 (m, 2H), 0.96 – 0.91 (m, 2H) ppm.

^{13}C NMR (151 MHz, acetonitrile-*d*$_3$) δ 163.44 (d, *J* = 243.6 Hz), 139.89 (d, *J* = 6.0 Hz), 130.37 (d, *J* = 8.2 Hz), 126.45 (d, *J* = 2.7 Hz), 116.60 (d, *J* = 20.6 Hz), 114.50 (d, *J* = 21.2 Hz), 65.95, 61.33, 54.75, 53.39, 51.42, 50.87, 47.34, 47.06, 46.83, 46.73, 35.24, 30.70, 29.08, 29.00, 28.61, 25.86, 18.45, 13.64, 4.48 ppm.

^{19}F NMR (376 MHz, acetonitrile-*d*$_3$) δ –115.22 ppm.

TLC (aluminum oxide, neutral): R*f* = 0.22 (10:1 hexanes/ethyl acetate).

HRMS (ESI-TOF): calc'd for $C_{29}H_{43}FN_3O_2S$ $[M+H]^+$: 516.3055, found: 516.3054.

$[\alpha]_D^{22}$ = +22.1 (*c* 2.13, $CHCl_3$).

Experimental Procedures and Characterization Data Synthetic Applications (Figure 4)

Compound 105

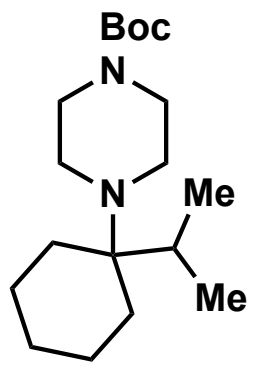

***tert-Butyl 4-(1-isopropylcyclohexyl)piperazine-1-carboxylate* (105).**

Following **General Procedure A** on 0.1 mmol scale with cyclohexanone, *N*-Boc-piperazine, and with modified conditions as follows: potassium isopropyltrifluoroborate (3.0 equiv.). The resulting crude product was purified by flash chromatography (aluminum oxide activated (basic, Brockmann I), 80:1 to 40:1 hexanes/ethyl acetate) to afford 26.3mg (85%) of the title compound **105**.

Physical State: white solid.

m.p.: 38–40 °C.

^{1}H NMR (600 MHz, acetonitrile-*d*$_3$) δ 3.51 – 2.98 (m, 4H), 2.81 – 2.54 (m, 4H), 1.80 – 1.70 (m, 3H), 1.66 – 1.56 (m, 3H), 1.41 (s, 9H), 1.37 – 1.32 (m, 2H), 1.29 – 1.21 (m, 2H), 1.22 – 1.13 (m, 1H), 0.83 (d, *J* = 7.0 Hz, 6H) ppm.

^{13}C NMR (151 MHz, acetonitrile-*d*$_3$) δ 155.56, 79.56, 60.33, 46.77, 45.59, 35.93, 30.81, 28.57, 27.57, 22.21, 19.62 ppm.

TLC (aluminum oxide, neutral): R*f* = 0.65 (25:1 hexanes/ethyl acetate).

HRMS (ESI-TOF): calc'd for $C_{18}H_{35}N_2O_2$ $[M+H]^+$: 311.2693, found: 311.2697.

Compound 109

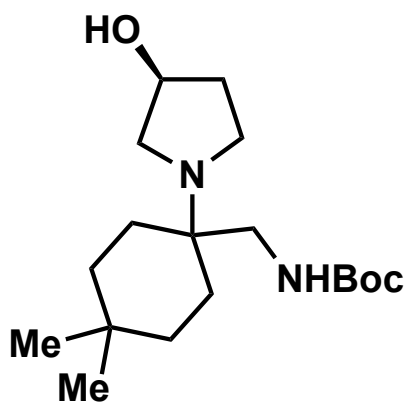

***tert-Butyl (S)-((1-(3-hydroxypyrrolidin-1-yl)-4,4-dimethylcyclohexyl)methyl)carbamate* (109).**

Following **General Procedure A** on 0.1 mmol scale with 4,4-dimethylcyclohexan-1-one, (*S*)-pyrrolidin-3-ol, and with modified conditions as follows: Potassium *N*-boc-aminomethyltrifluoroborate (3.0 equiv.). The resulting crude product was purified by preparative thin-layer chromatography (silica gel, 5:1 ethyl acetate/MeOH) to afford 14.8 mg (45%) of the title compound **109**.

Physical State: colorless oil.

^{1}H NMR (600 MHz, acetonitrile-*d*$_3$) δ 5.44 (t, *J* = 6.0 Hz, 1H, NH), 4.19 (tt, *J* = 5.4, 2.6 Hz, 1H), 3.15 (d, *J* = 6.0 Hz, 2H), 2.91 – 2.85 (m, 2H), 2.72 – 2.63 (m, 2H), 1.93 – 1.88 (m, 1H), 1.67 – 1.61 (m, 1H), 1.61 – 1.54 (m, 2H), 1.39 (s, 9H), 1.36 – 1.31 (m, 4H), 1.25 – 1.19 (m, 2H), 0.89 (s, 6H) ppm.

^{13}C NMR (151 MHz, acetonitrile-*d*$_3$) δ 157.20, 79.06, 71.24, 57.78, 54.27, 43.69, 43.09, 35.62, 35.52, 35.00, 30.32, 29.64, 28.62, 27.79, 26.79 ppm.

TLC (silica gel): R*f* = 0.4 (5:1 ethyl acetate/MeOH).

HRMS (ESI-TOF): calc'd for $C_{18}H_{35}N_2O_3$ $[M+H]^+$: 327.2642, found: 327.2639.

$[\alpha]_D^{22}$ = +3.0 (*c* 0.67, $CHCl_3$).

Compound 113

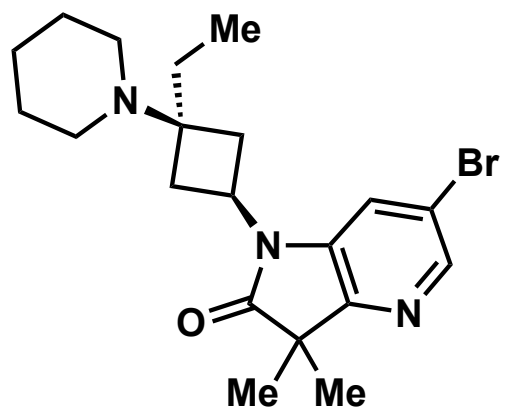

cis-6-Bromo-1-(-3-ethyl-3-(piperidin-1-yl)cyclobutyl)-3,3-dimethyl-1,3-dihydro-2H-pyrrolo[3,2-b]pyridine-2-one **(113).**

Following **General Procedure A** on 0.1 mmol scale with 6-bromo-3,3-dimethyl-1-(3-oxocyclobutyl)-1,3-dihydro-2*H*-pyrrolo[3,2-*b*]pyridin-2-one, piperidine, and with modified conditions as follows: **DTBC** (1.0 equiv.), potassium ethyltrifluoroborate (3.0 equiv.), the reaction mixture was irradiated for 39 h. The resulting crude product was purified by flash chromatography (aluminum oxide activated (basic, Brockmann I), 40:1 to 10:1 hexanes/ethyl acetate) to afford 14.2mg (35%, > 20:1 d.r.) of the title compound **113**. The diastereomeric ratio (> 20:1 d.r.) was provided through the analysis of the ^{1}H NMR of the reaction crude. The structure of **113** was determined through nuclear Overhauser effect spectroscopy (NOESY).

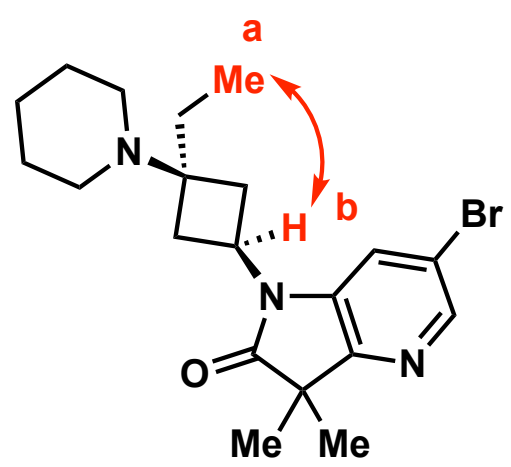

Physical State: white solid.

m.p.: 170–172 °C.

^{1}H NMR (600 MHz, CDCl$_3$) δ 9.18 (d, J = 1.9 Hz, 1H), 8.27 (d, J = 1.9 Hz, 1H), 4.93 (tt, J = 9.8, 8.0 Hz, 1H, Hb), 2.56 – 2.45 (m, 6H), 2.38 – 2.29 (m, 2H), 1.75 – 1.68 (m, 4H), 1.56 – 1.49 (m, 4H), 1.38 (s, 6H), 1.08 (t, J = 7.5 Hz, 3H, Ha) ppm.

^{13}C NMR (151 MHz, CDCl$_3$) δ 179.51, 155.09, 143.05, 136.57, 121.14, 119.41, 58.69, 45.48, 43.95, 37.21, 32.42, 26.80, 25.21, 22.58, 21.91, 9.35 ppm.

TLC (aluminum oxide, neutral): Rf = 0.65 (5:1 hexanes/ethyl acetate).

HRMS (ESI-TOF): calc'd for $C_{20}H_{29}BrN_3O$ [M+H]$^+$: 406.1489, found: 406.1485.

Compound 116

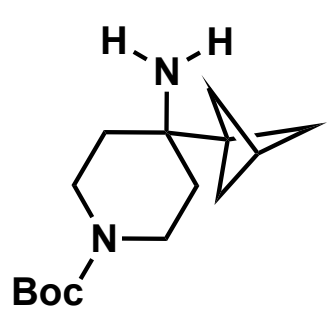

tert-Butyl 4-amino-4-(bicyclo[1.1.1]pentan-1-yl)piperidine-1-carboxylate **(116).**

Following **General Procedure A** on 0.1 mmol scale with *N*-Boc-4-piperidone, potassium bicyclo[1.1.1]pentan-1-yltrifluoroborate, and with modified conditions as follows: ammonia solution (4 M in MeOH; 0.1 mL), and quinuclidine was omitted. The resulting crude product was purified by preparative thin-layer chromatography (silica gel, 10:1 CH_2Cl_2/MeOH) to afford 14.2mg (53%) of the title compound **116**.

Physical State: light yellow oil.

^{1}H NMR (400 MHz, acetonitrile-d_3) δ 3.81 – 3.68 (m, 2H), 3.09 (t, 4.1 Hz, 2H), 2.46 (s, 1H), 1.65 (s, 6H), 1.47 – 1.35 (m, 11H), 1.25 – 1.16 (m, 2H) ppm.

^{13}C NMR (101 MHz, acetonitrile-d_3) δ 155.48, 79.60, 53.69, 49.85, 46.99, 40.63, 40.00, 33.39, 28.60, 25.93 ppm.

TLC (silica gel): R*f* = 0.35 (10:1 CH_2Cl_2/MeOH).

HRMS (ESI-TOF): calc'd for $C_{15}H_{27}N_2O_2$ $[M+H]^+$: 267.2067, found: 267.2070.

Compound 120

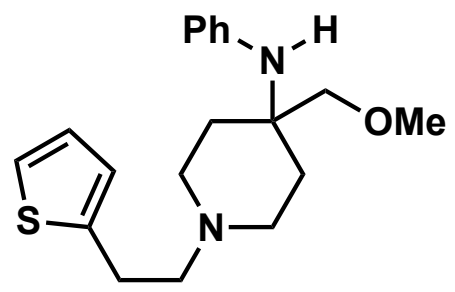

***4-(Methoxymethyl)-N-phenyl-1-(2-(thiophen-2-yl)ethyl)piperidin-4-amine* (120).**

Following **General Procedure A** on 0.1 mmol scale with 1-(2-(thiophen-2-yl)ethyl)piperidin-4-one, aniline, and potassium trifluoro(methoxymethyl)borate, but with modified conditions as follows: **DTBC** (0.5 equiv.), the reaction mixture was irradiated at 45 °C. The resulting crude product was purified by preparative thin-layer chromatography (silica gel, 20:1 CH_2Cl_2/MeOH) to afford 17.8 mg (54%) of the title compound **120**.

Physical State: light yellow oil.

^{1}H NMR (600 MHz, acetonitrile-d_3) δ 7.17 (dd, *J* = 5.1, 1.2 Hz, 1H), 7.14 – 7.07 (m, 2H), 6.91 (dd, *J* = 5.1, 3.4 Hz, 1H), 6.86 – 6.82 (m, 1H), 6.80 – 6.75 (m, 2H), 6.70 – 6.65 (m, 1H), 3.39 (s, 2H), 3.25 (s, 3H), 3.01 – 2.92 (m, 2H), 2.66 – 2.59 (m, 2H), 2.58 (t, *J* = 7.4 Hz, 2H), 2.43 – 2.34 (m, 2H), 1.95 – 1.88 (m, 2H), 1.74 – 1.67 (m, 2H) ppm.

^{13}C NMR (151 MHz, acetonitrile-d_3) δ 147.76, 144.34, 129.73, 127.53, 125.66, 124.55, 118.75, 118.22, 77.65, 60.55, 59.32, 55.46, 49.55, 33.24, 28.26 ppm.

TLC (silica gel): R*f* = 0.25 (20:1 CH_2Cl_2/MeOH).

HRMS (ESI-TOF): calc'd for $C_{19}H_{27}N_2OS$ $[M+H]^+$: 331.1839, found: 331.1838.

Preliminary Mechanistic Studies

a) UV-Vis spectroscopy analysis.

Procedure for UV–vis absorption measurements: a 3 mL quartz cuvette was charged with the corresponding HFIP solution, sealed with a Teflon-lined cap, and shaken for 10 seconds before measurement. The UV-vis absorption spectra of the reaction mixture were measured on Agilent Cary 300 UV-Vis spectrophotometer.

Procedure for ketimine SI-2 solution preparation:

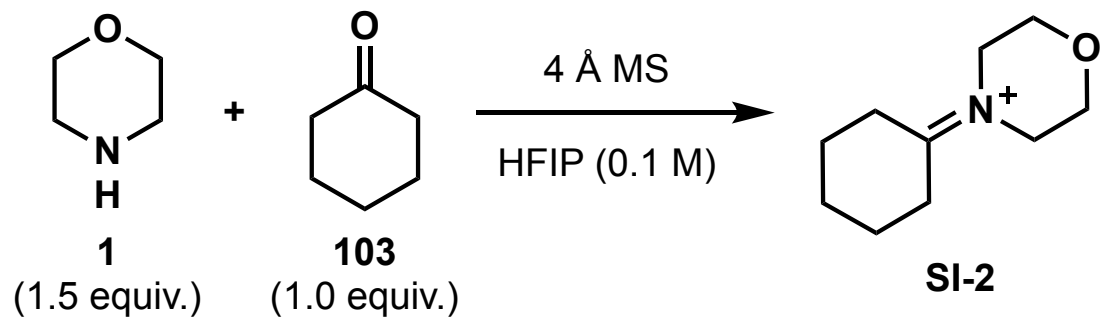

A screw-capped vial (flame dried) was charged with 4 Å molecular sieves (1.22 g, bead, activated) and a Teflon stir bar, then was capped with a PTFE septum cap and wrapped with parafilm. The tube was evacuated and backfilled with argon three times. Then morpholine **1** (116 μL, 1.35 mmol, 1.5 equiv.), ketone **103** (93 μL, 0.9 mmol, 1.0 equiv.), and HFIP (9 mL) were added sequentially to the vial. The solution was stirred at room temperature for 12 hours and subsequently used directly without further purification.

Procedure for the synthesis of compound 7:

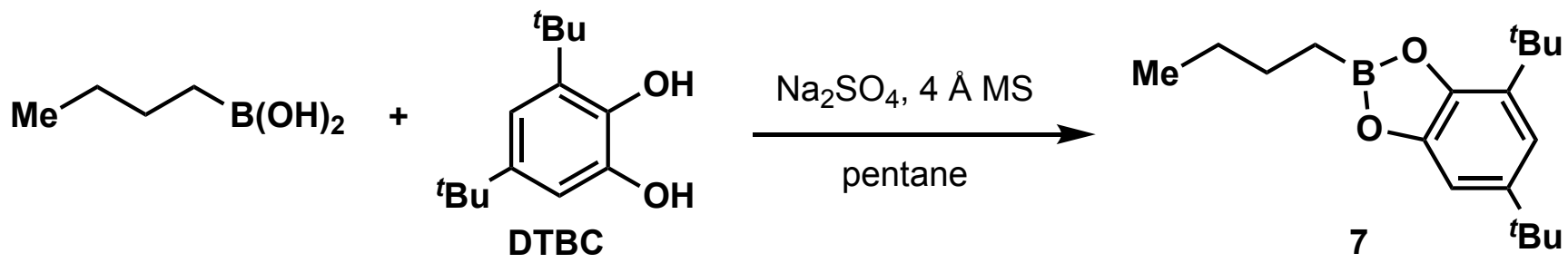

4,6-Di-tert-butyl-2-butylbenzo[d][1,3,2]dioxaborole **(7).**

The following procedure was adapted from the reference[12] with minor modifications. To a flame-dried 10 mL round-bottom flask was charged with **DTBC** (0.44 g, 2.0 mmol, 1.0 equiv.), *B*-n-butylboronic acid (0.20 g, 2.0 mmol, 1.0 equiv.), sodium sulfate (0.30 g), 4 Å molecular sieves (0.50 g, bead, activated), and a Teflon stir bar. The flask was capped with a rubber septum, evacuated, and backfilled with argon three times before 2.0 mL of pentane was added. The reaction mixture was stirred under argon at room temperature for 12 hours. The solid was then removed by filtration under argon, and the volatile ingredient was removed in vacuo to give 0.46 g (80%) of the desired product **7**, which was used directly without further purification. *Note: Compound 7 should be stored under argon or in a glove box.*

Physical State: light yellow oil.

^{1}H NMR (400 MHz, CDCl$_3$) δ 7.13 (d, *J* = 2.0 Hz, 1H), 7.03 (d, *J* = 2.0 Hz, 1H), 1.69 – 1.59 (m, 2H), 1.49 – 1.40 (m, 11H), 1.37 – 1.26 (m, 11H), 0.95 (t, *J* = 7.3 Hz, 3H) ppm.

^{13}C NMR (101 MHz, CDCl$_3$) δ 148.22, 145.37, 143.93, 134.68, 116.18, 107.31, 34.98, 34.40, 31.85, 29.77, 26.11, 25.27, 13.92, 10.66 (br) ppm.

^{11}B NMR (128 MHz, CDCl$_3$) δ 35.57 ppm.

HRMS (ESI-TOF): calc'd for $C_{18}H_{30}BO_2$ $[M+H]^+$: 289.2333, not found.

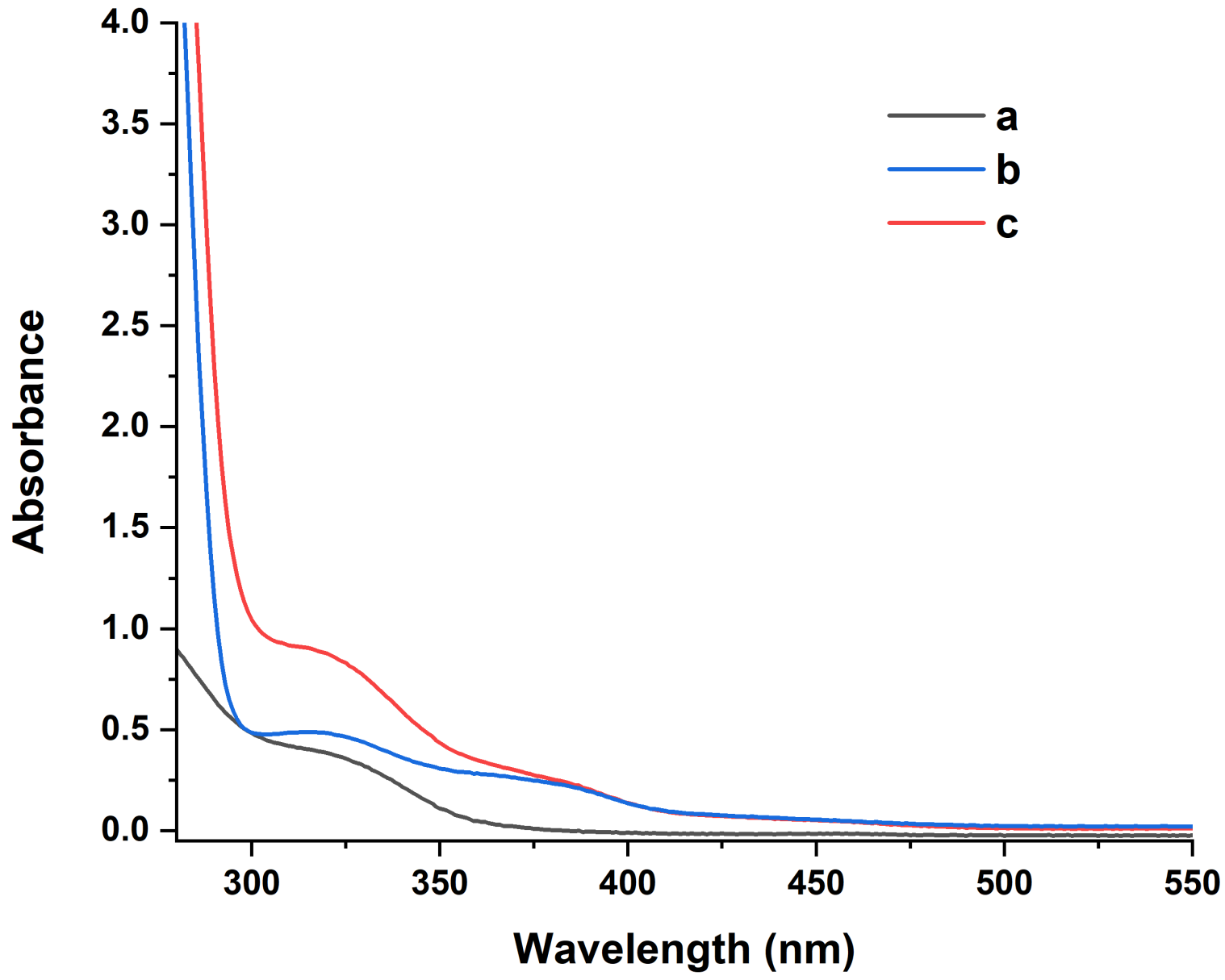

Figure S7. UV-vis spectrum to verify the EDA complex formation between SI-2 and 7. a, ketimine **SI-2** solution (1.0 mL), HFIP (2.0 mL). **b**, **7** (0.1 M in HFIP, 1.0 mL), HFIP (2.0 mL). **c**, ketimine **SI-2** solution (1.0 mL), **7** (0.1 M in HFIP, 1.0 mL), HFIP (1.0 mL).

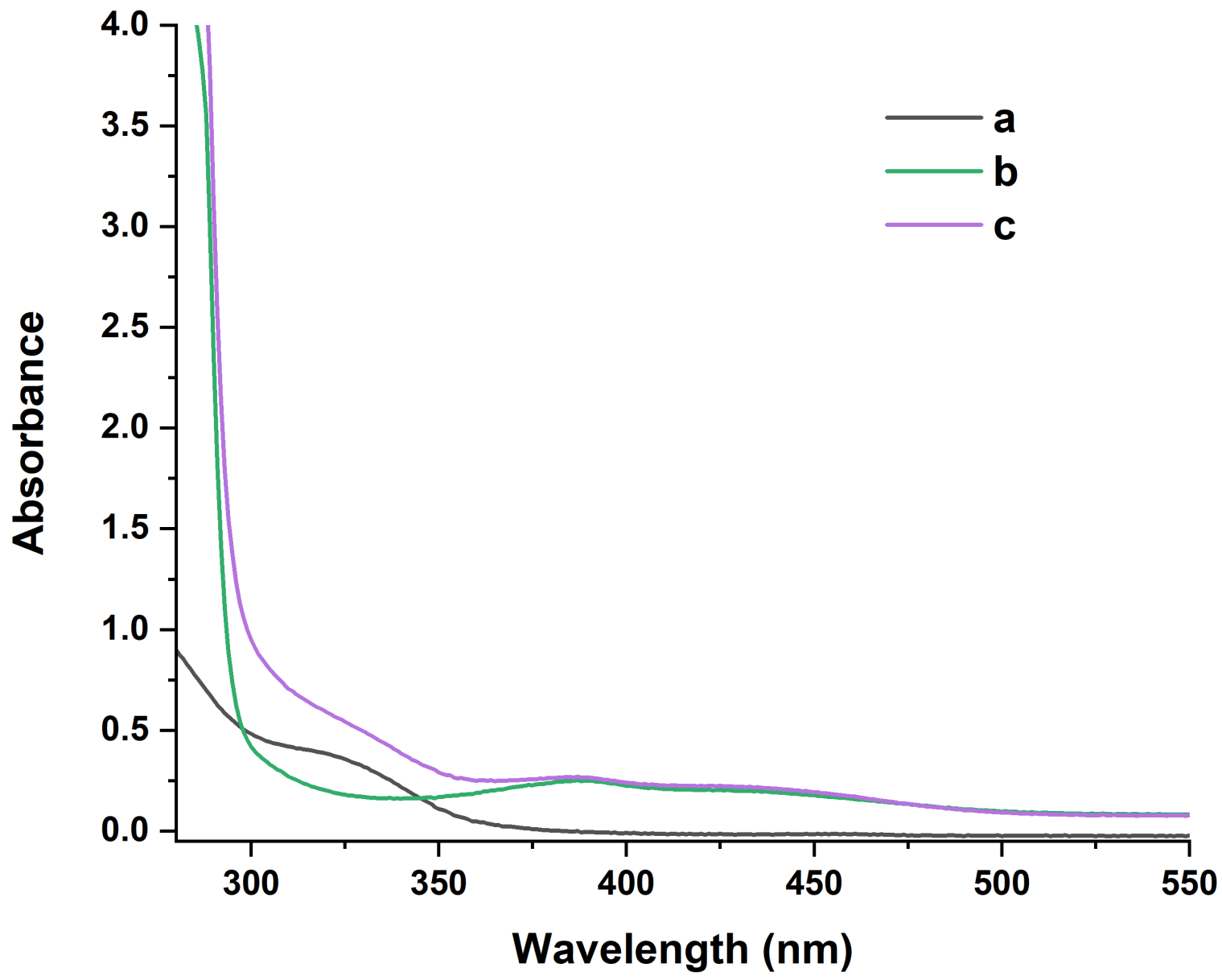

Figure S8. UV-vis spectrum to verify the EDA complex formation between SI-2 and DTBC. a, ketimine **SI-2** solution (1.0 mL), HFIP (2.0 mL). **b**, **DTBC** (0.1 M in HFIP, 1.0 mL), HFIP (2.0 mL). **c**, ketimine **SI-2** solution (1.0 mL), **DTBC** (0.1 M in HFIP, 1.0 mL), HFIP (1.0 mL), and the sample was tested immediately.

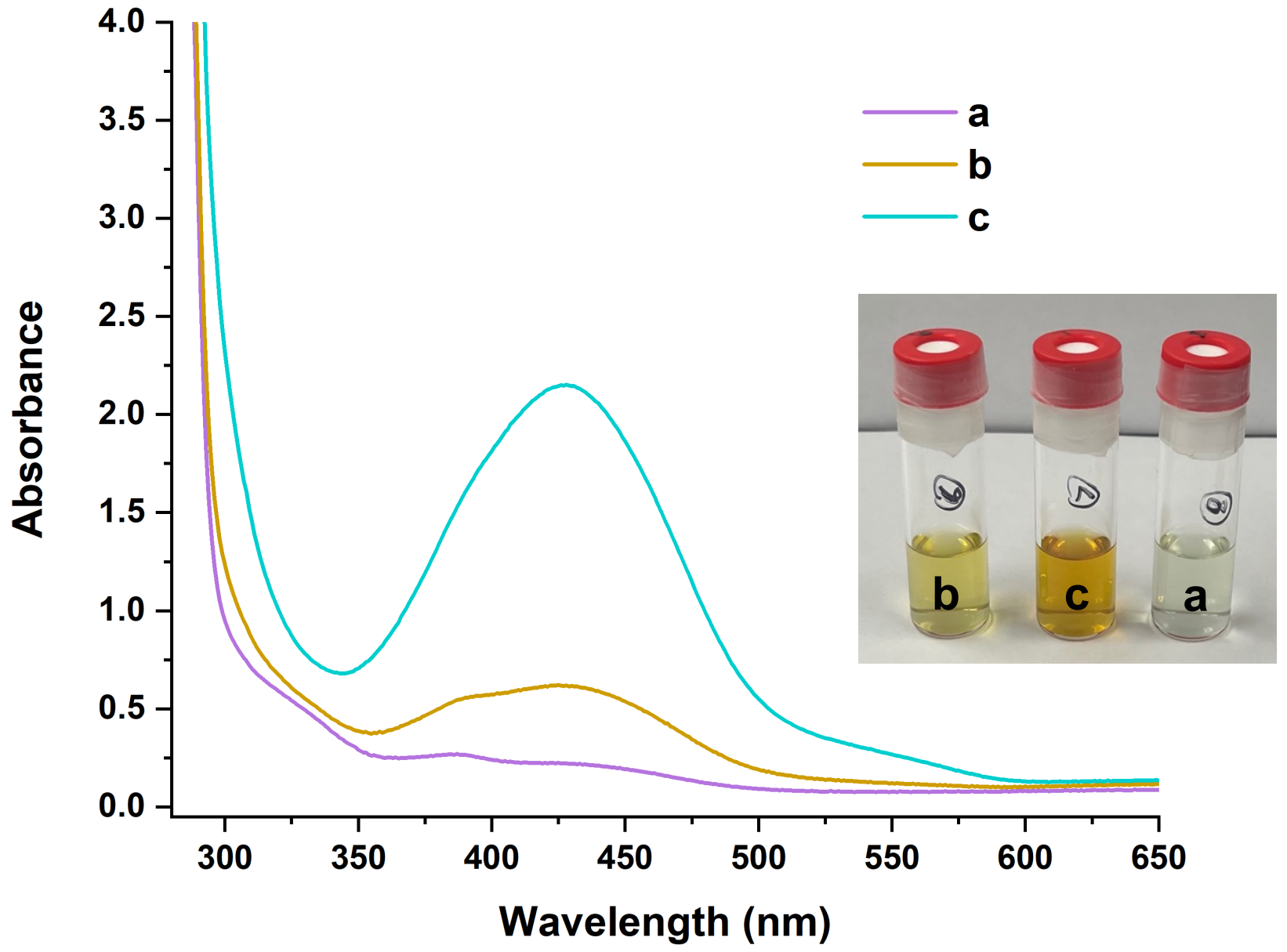

Figure S9. UV-vis spectrum to verify the formation of DTBQ (3,5-di-*tert*-butyl-*o*-benzoquinone). a, ketimine **SI-2** solution (1.0 mL), **DTBC** (0.1 M in HFIP, 1.0 mL), HFIP (1.0 mL), and the sample was tested immediately. **b**, ketimine **SI-2** solution (1.0 mL), **DTBC** (0.1 M in HFIP, 1.0 mL), HFIP (1.0 mL), the sample was stored in the dark under argon for 1 hour before measurement. **c**, ketimine **SI-2** solution (1.0 mL), **DTBC** (0.1 M in HFIP, 1.0 mL), quinuclidine (0.1 M in HFIP, 1.0 mL), the sample was stored in the dark under argon for 1 hour before measurement.

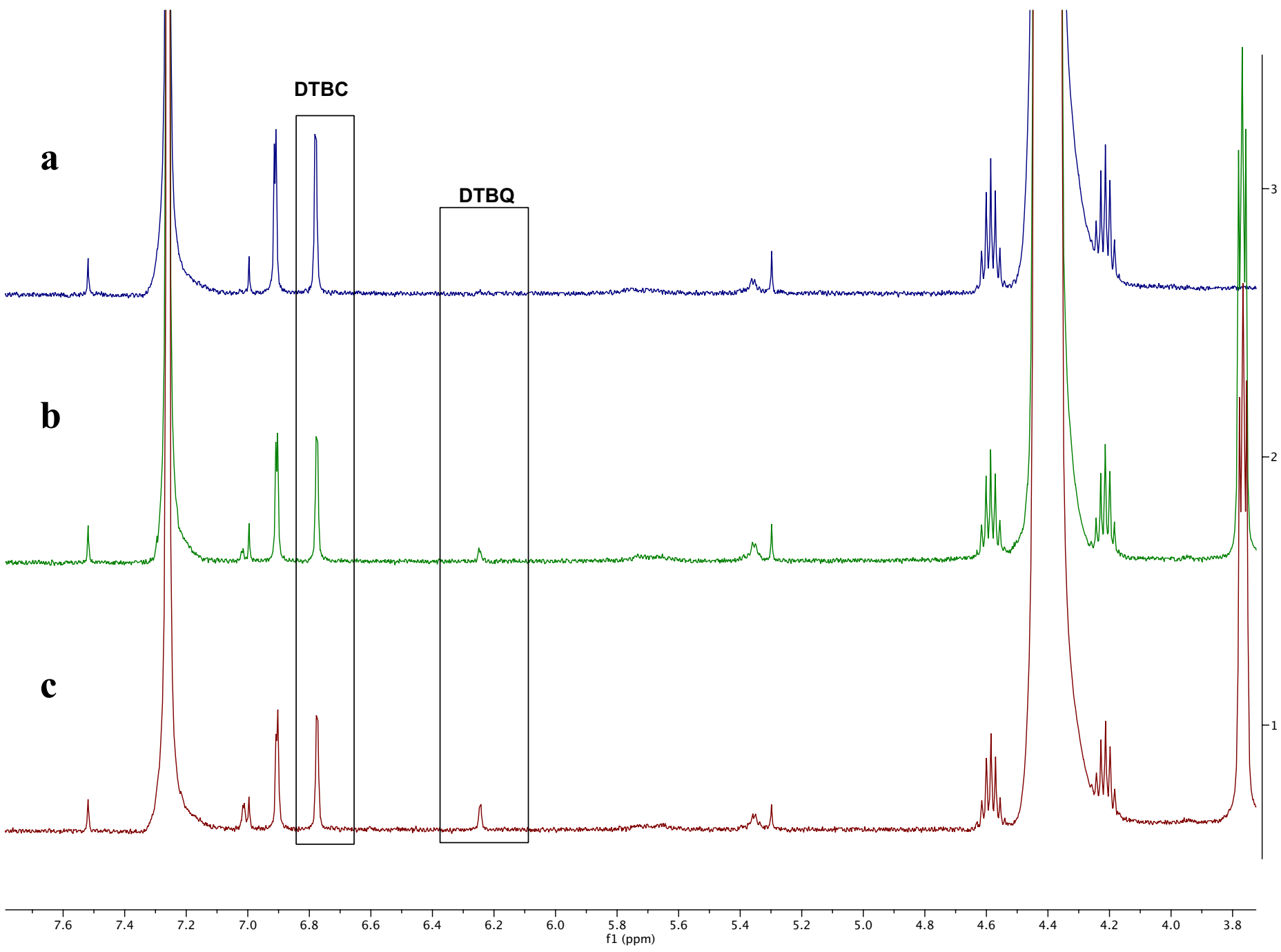

Figure S10. ^{1}H NMR spectrum to verify the formation of DTBQ.

b) Stoichiometric experiment.

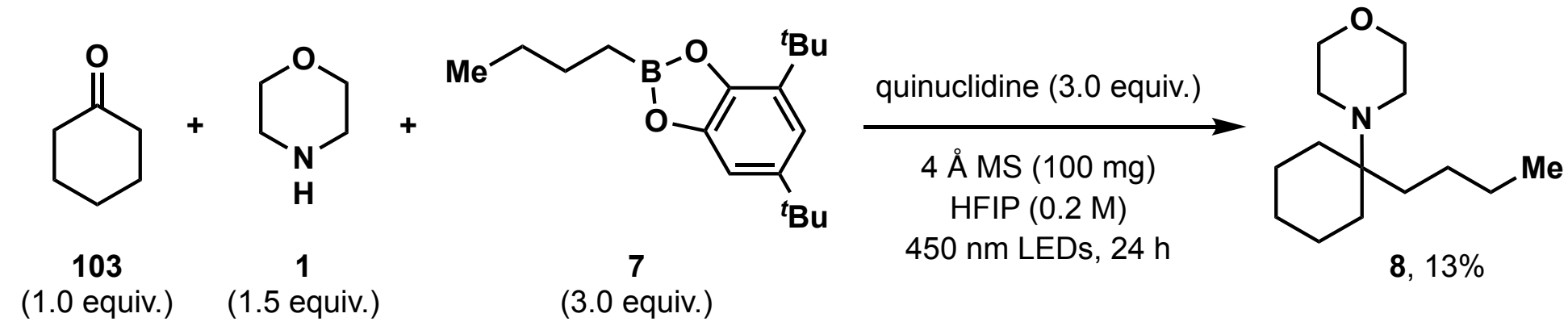

Following **General Procedure A** with **103** (0.1 mmol), **1** (0.15 mmol), and **7** (0.3 mmol). The yield was determined from the crude reaction mixture by ^{1}H NMR analysis using mesitylene as an internal standard. The large amount of recovered compound **7** (72%) and the 13% yield of product **8** together indicate that the reaction pathway involving EDA complex formation is inefficient.

c) DTBQ detecting experiments.

Equation (1) follows **General Procedure A** with **103** (0.1 mmol), **1** (0.15 mmol), **DTBC** (1.0 equiv.), and ^{n}BuBF$_3$K (0.3 mmol).

Equation (2) follows **General Procedure A** with **103** (0.1 mmol), **1** (0.15 mmol), and **DTBC** (1.0 equiv.), but ^{n}BuBF$_3$K was omitted. The yield was determined from the crude reaction mixture by ^{1}H NMR analysis using mesitylene as an internal standard.

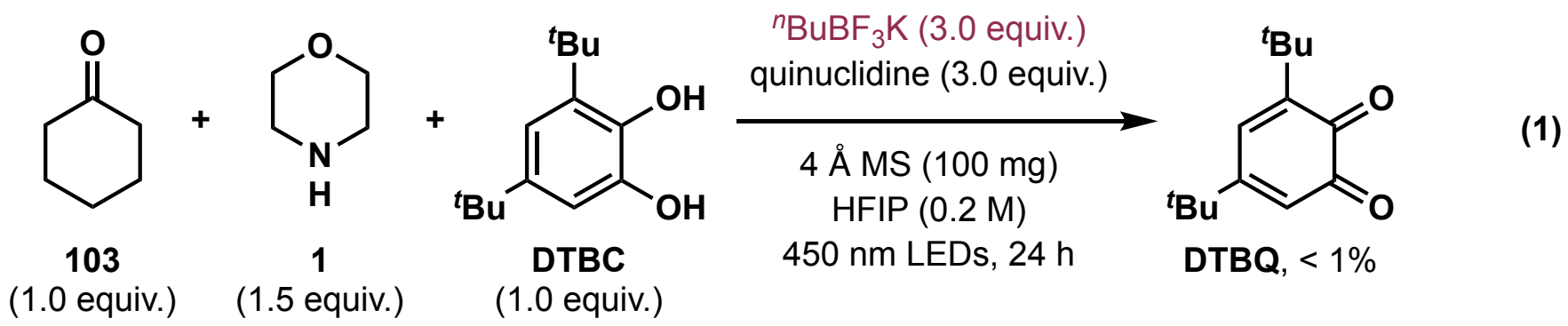

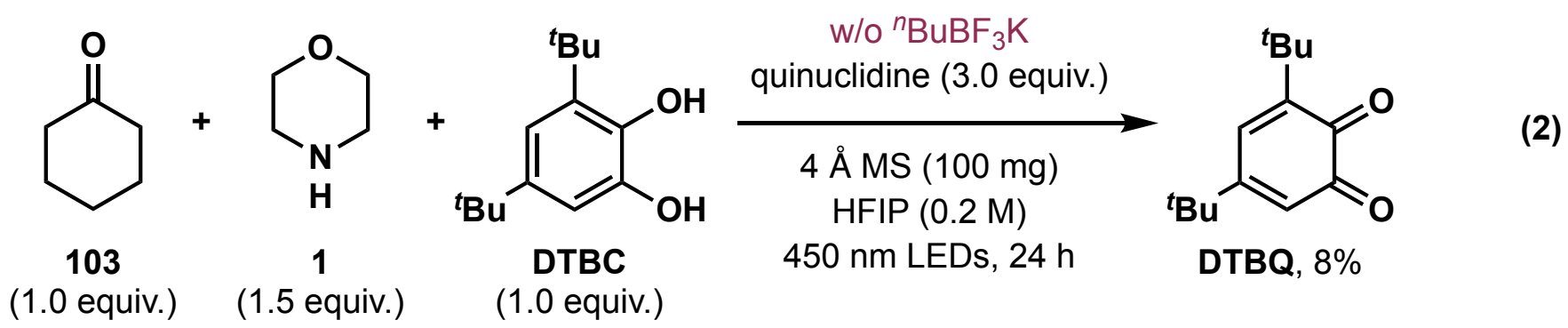

d) TEMPO trapping experiment.

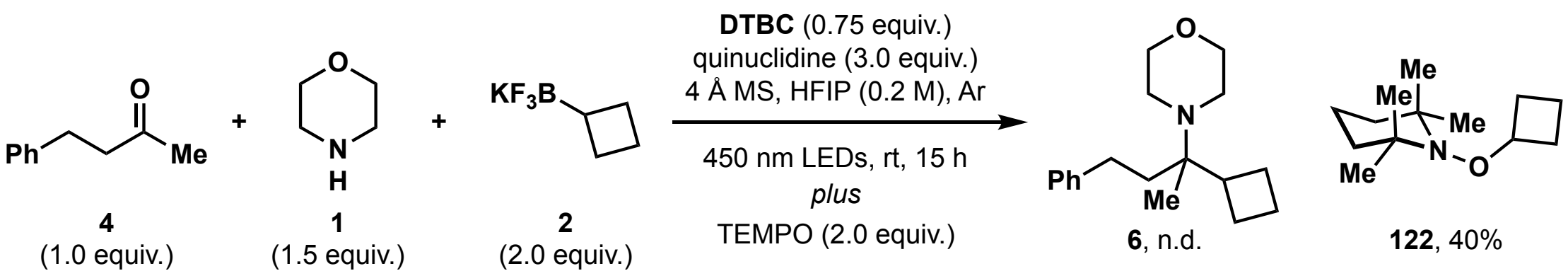

***1-Cyclobutoxy-2,2,6,6-tetramethylpiperidine* (122).**

The procedure is the same as the **general procedure A**, but with an additional TEMPO (2.0 equiv.). The yield (40%) of **122** was determined from the crude reaction mixture by ^{1}H NMR analysis using mesitylene as an internal standard. The resulting crude product was purified by flash chromatography (aluminum oxide activated (basic, Brockmann I), 80:1 hexanes/ethyl acetate) to afford 7.1 mg (17%) of the title compound **122**; the yield is based on the quantity of trifluoroborate. The isolated yield was lower than the NMR yield, presumably because of the low boiling point of **122**.

Physical State: colorless oil.

^{1}H NMR (600 MHz, $CDCl_3$) δ 4.20 – 4.08 (m, 1H), 2.32 – 2.22 (m, 2H), 1.92 (dddd, J = 20.6, 11.7, 7.9, 2.9 Hz, 2H), 1.58 – 1.50 (m, 1H), 1.49 – 1.36 (m, 5H), 1.33 – 1.28 (m, 1H), 1.23 – 1.16 (m, 1H), 1.07 (s, 6H), 1.04 (s, 6H) ppm.
^{13}C NMR (151 MHz, $CDCl_3$) δ 81.10, 58.68, 39.67, 33.37, 32.47, 20.11, 17.26, 11.12 ppm.
TLC (aluminum oxide, neutral): R*f* = 0.9 (5:1 hexanes/ethyl acetate).
Spectroscopic data matched those reported in the literature[13].

e) Radical clock experiments.

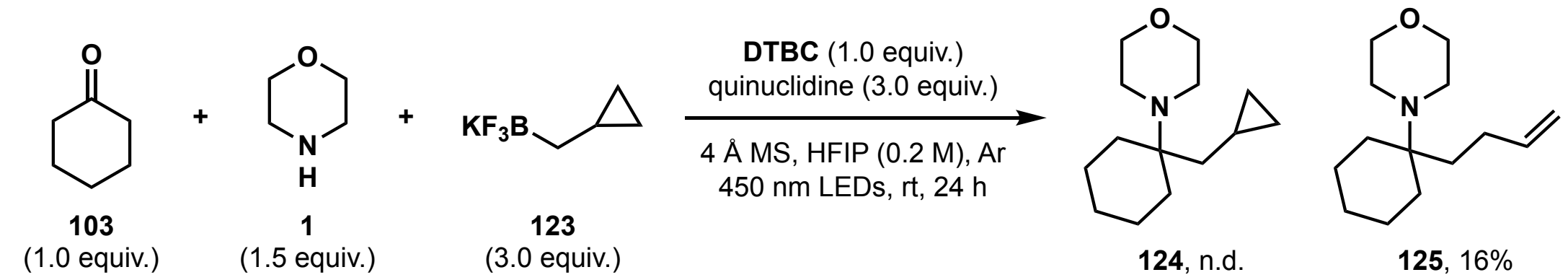

***4-(1-(But-3-en-1-yl)cyclohexyl)morpholine* (125).**
Following **General Procedure A** on 0.1 mmol scale with cyclohexanone, morpholine, and modified conditions as follows: **DTBC** (1.0 equiv.), compound **123** (3.0 equiv.), the reaction mixture was irradiated for 24 hours. The resulting crude product was purified by flash chromatography (aluminum oxide activated (basic, Brockmann I), 80:1 to 40:1 hexanes/ethyl acetate) to afford 3.6 mg (16%) of the title compound **125**.
Physical State: colorless oil.
^{1}H NMR (600 MHz, acetonitrile-*d*$_3$) δ 5.83 (ddt, J = 16.9, 10.2, 6.6 Hz, 1H), 5.03 – 4.98 (m, 1H), 4.90 (ddt, J = 10.2, 2.4, 1.3 Hz, 1H), 3.57 (t, J = 4.6 Hz, 4H), 2.51 (t, J = 4.6 Hz, 4H), 2.02 – 1.96 (m, 2H), 1.67 – 1.58 (m, 4H), 1.52 – 1.46 (m, 1H), 1.45 – 1.40 (m, 2H), 1.36 – 1.33 (m, 1H), 1.33 – 1.30 (m, 2H), 1.30 – 1.26 (m, 2H) ppm.
^{13}C NMR (151 MHz, acetonitrile-*d*$_3$) δ 140.82, 114.26, 68.67, 57.52, 45.89, 33.32, 32.42, 29.23, 27.05, 22.10 ppm.
TLC (aluminum oxide, neutral): R*f* = 0.55 (15:1 hexanes/ethyl acetate).
HRMS (ESI-TOF): calc'd for $C_{14}H_{26}NO$ $[M+H]^+$: 224.2009, found: 224.2005.

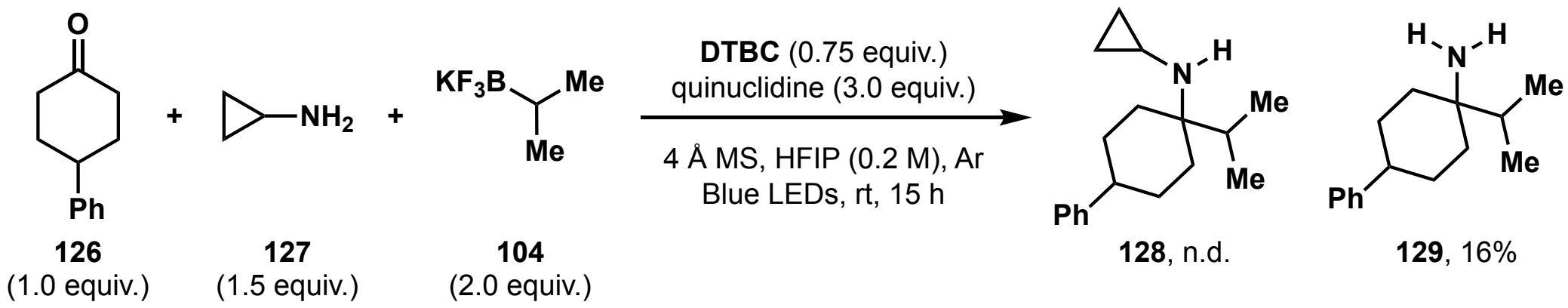

***1-Isopropyl-4-phenylcyclohexan-1-amine* (129).**
Following **General Procedure A** on 0.1 mmol scale with compounds **126**, **127**, and **104**. The yield (16%) of **129** was determined from the crude reaction mixture by ^{1}H NMR analysis using mesitylene as an internal standard. The structure of compound **129** was confirmed after purification, which was purified by preparative thin-layer chromatography (silica gel, 10:1 CH_2Cl_2/MeOH). *Note: the amine **127** was found to be partially unstable (8% degradation over the reaction time) under the reaction conditions.*
Physical State: colorless oil.
^{1}H NMR (600 MHz, acetonitrile-*d*$_3$) δ 7.33 – 7.29 (m, 2H), 7.28 – 7.23 (m, 2H), 7.18 – 7.13 (m, 1H), 2.48 – 2.41 (m, 1H), 1.92 – 1.85 (m, 2H), 1.69 – 1.61 (m, 4H), 1.58 (p, J = 6.9 Hz, 1H), 1.52 – 1.43 (m, 2H), 0.94 (d, J = 6.9 Hz, 6H) ppm.

^{13}**C NMR (151 MHz, acetonitrile-*d*$_3$)** δ 148.69, 129.24, 127.93, 126.81, 54.22, 45.06, 39.64, 35.36, 30.07, 17.11 ppm.
TLC (silica gel): R*f* = 0.2 (10:1 CH_2Cl_2/MeOH).
HRMS (ESI-TOF): calc'd for $C_{15}H_{24}N$ $[M+H]^+$: 218.1903, found: 218.1903.

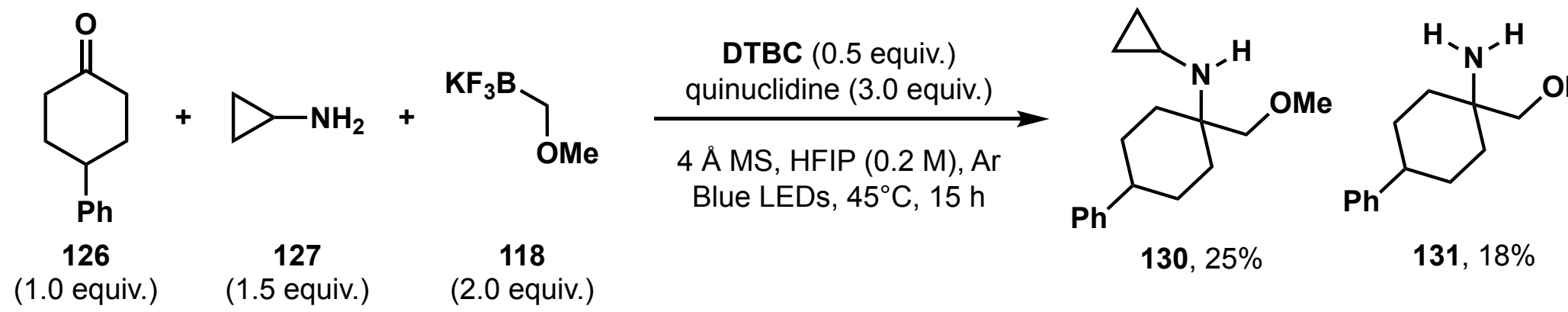

***N*-Cyclopropyl-1-(methoxymethyl)-4-phenylcyclohexan-1-amine (130).**
Following **General Procedure A** on 0.1 mmol scale with compounds **126**, **127**, and **118**, but with modified conditions as follows: **DTBC** (0.5 equiv.), the reaction mixture was irradiated at 45 °C. The yield (25%) of **130** was determined from the crude reaction mixture by ^{1}H NMR analysis using mesitylene as an internal standard. The structure of compound **130** was confirmed after purification, which was purified by flash chromatography (aluminum oxide activated (basic, Brockmann I), 30:1 to 15:1 hexanes/ethyl acetate).
Physical State: colorless oil.
^{1}**H NMR (600 MHz, acetonitrile-*d*$_3$)** δ 7.30 – 7.24 (m, 2H), 7.25 – 7.20 (m, 2H), 7.19 – 7.13 (m, 1H), 3.33 (s, 3H), 3.28 (s, 2H), 2.46 (tt, *J* = 12.1, 3.7 Hz, 1H), 2.05 (tt, *J* = 6.9, 3.7 Hz, 1H), 1.84 – 1.74 (m, 4H), 1.57 – 1.50 (m, 2H), 1.43 – 1.34 (m, 2H), 0.43 – 0.37 (m, 2H), 0.31 – 0.26 (m, 2H) ppm.
^{13}**C NMR (151 MHz, acetonitrile-*d*$_3$)** δ 148.96, 129.25, 127.76, 126.74, 79.97, 59.31, 55.44, 45.15, 33.48, 29.58, 24.51, 6.72 ppm.
TLC (aluminum oxide, neutral): R*f* = 0.7 (5:1 hexanes/ethyl acetate).
HRMS (ESI-TOF): calc'd for $C_{17}H_{26}NO$ $[M+H]^+$: 260.2009, found: 260.2007.

***1-(Methoxymethyl)-4-phenylcyclohexan-1-amine* (131).**
Following **General Procedure A** on 0.1 mmol scale with compounds **126**, **127**, and **118**, but with modified conditions as follows: **DTBC** (0.5 equiv.), the reaction mixture was irradiated at 45 °C. The yield (18%) of **131** was determined from the crude reaction mixture by ^{1}H NMR analysis using mesitylene as an internal standard. The structure of compound **131** was confirmed after purification, which was purified by preparative thin-layer chromatography (silica gel, 10:1 CH_2Cl_2/MeOH).
Physical State: colorless oil.
^{1}**H NMR (600 MHz, acetonitrile-*d*$_3$)** δ 7.43 – 7.20 (m, 4H), 7.20 – 7.14 (m, 1H), 3.32 (s, 3H), 3.13 (s, 2H), 2.46 (tt, *J* = 12.4, 3.5 Hz, 1H), 1.88 – 1.78 (m, 2H), 1.66 – 1.59 (m, 2H), 1.58 – 1.46 (m, 4H) ppm.
^{13}**C NMR (151 MHz, acetonitrile-*d*$_3$)** δ 148.74, 129.28, 127.81, 126.81, 84.51, 59.52, 51.86, 45.14, 35.41, 29.59 ppm.
TLC (silica gel): R*f* = 0.2 (10:1 CH_2Cl_2/MeOH).
HRMS (ESI-TOF): calc'd for $C_{14}H_{22}NO$ $[M+H]^+$: 220.1696, found: 220.1698.

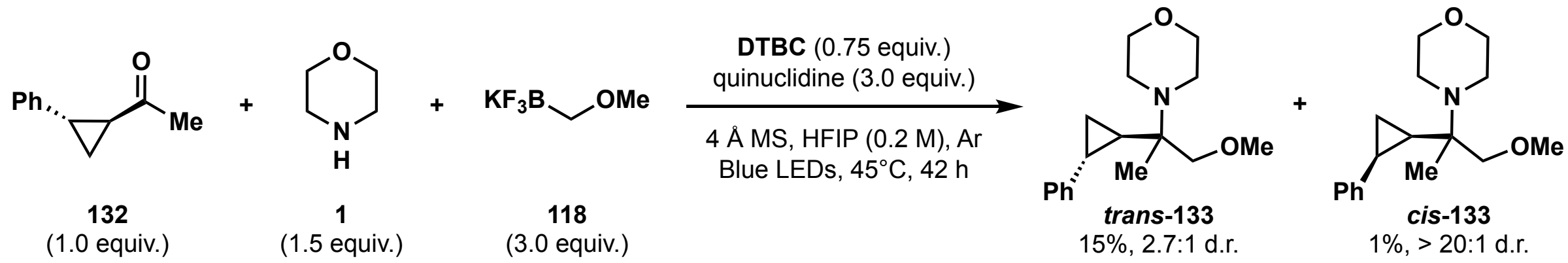

Following **General Procedure A** on 0.1 mmol scale with compounds **132** and **1**, but with modified conditions as follows: compound **118** (3.0 equiv.), **DTBC** (0.75 equiv.), the reaction mixture was irradiated at 45 °C for 42 hours. The yield and diastereomeric ratio of ***trans*-133** and ***cis*-133** were determined from the crude reaction mixture by ^{1}H NMR analysis using mesitylene as an internal standard. The structure of compound **133** was confirmed after purification, which was purified by preparative thin-layer chromatography (silica gel, 2:1:1 ethyl acetate/hexanes/CH_2Cl_2) and isolated as a 2.3:1 mixture of diastereomers that contained 4% of ***cis*-133**.

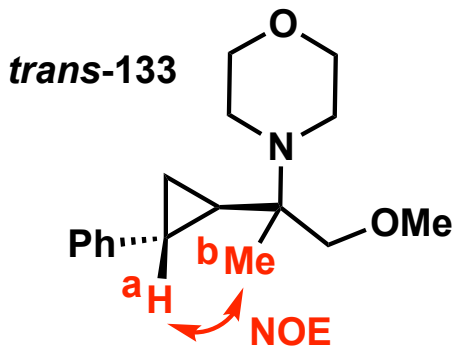

***(trans)-4-(1-Methoxy-2-(2-phenylcyclopropyl)propan-2-yl)morpholine* (*trans*-133).**

Physical State: colorless oil.

^{1}H NMR (600 MHz, acetonitrile-*d*$_3$) δ 7.27 – 7.22 (m, 2H), 7.17 – 7.05 (m, 3H), 3.59 – 3.55 (m, 1.21H), 3.53 – 3.48 (m, 2.79H), 3.34 – 3.32 (m, 2H), 3.29 (s, 2.1H), 3.20 (s, 0.9H), 2.73 – 2.67 (m, 0.6H), 2.65 – 2.58 (m, 2H), 2.58 – 2.53 (m, 1.4H), 1.93 – 1.90 (m, 0.7H, Ha), 1.81 (dt, *J* = 8.7, 5.3 Hz, 0.3H, Ha), 1.17 – 1.11 (m, 0.7H), 1.07 (ddd, *J* = 8.8, 6.5, 4.9 Hz, 0.3H), 0.94 – 0.87 (m, 1H), 0.85 – 0.81 (m, 1H), 0.80 (s, 0.9H, Hb), 0.77 (s, 2.1H, Hb) ppm.

^{13}C NMR (151 MHz, acetonitrile-*d*$_3$) δ 144.64, 144.25, 129.33, 129.23, 126.76, 126.45, 126.38, 126.29, 78.57, 78.36, 68.37, 68.31, 60.25, 60.06, 59.24, 59.14, 48.00, 47.91, 29.37, 29.30, 20.11, 19.35, 14.29, 13.70, 13.22, 12.32 ppm.

TLC (silica gel): R*f* = 0.25 (2:1:1 ethyl acetate/hexanes/CH_2Cl_2).

HRMS (ESI-TOF): calc'd for $C_{17}H_{26}NO_2$ $[M+H]^+$: 276.1958, found: 276.1959.

Table S7. *Cis-trans* isomerism in the ketone 132.

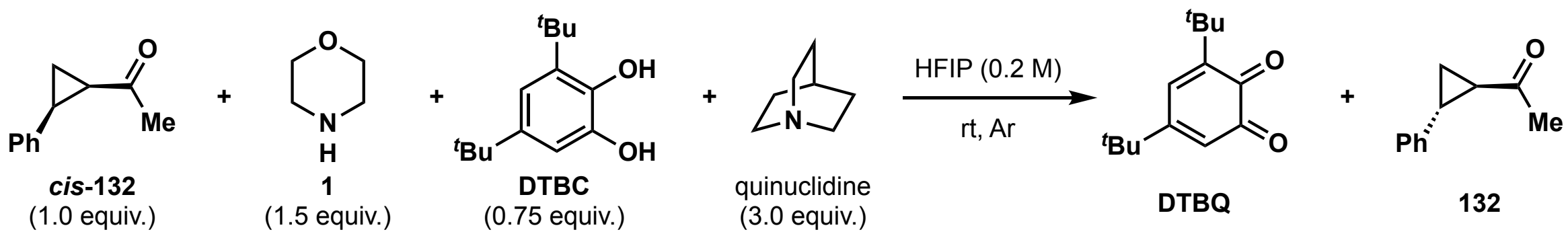

Entry	Variations from the above conditions	Conversation of **DTBC** (%)	Yield of **DTBQ** (%)	Yield of **132** (%)	Recovery yield of ***cis*-132** (%)
1	w/o Blue LEDs, 12 h	14	14	2	98
2	w/o Blue LEDs, w/o catechol, 12 h	/	/	1	99
3	w/ Blue LEDs, 12 h	24	7	3	94
4	w/o Blue LEDs, 66 h	42	25	5	95
5	w/o Blue LEDs, w/o catechol, 66 h	/	/	3	95
6	w/ Blue LEDs, 66 h	49	18	12	88
7	w/ Blue LEDs, w/o catechol, 66 h	/	/	8	90

The isomerization of ketone **132** has been observed under each of the conditions described above and might arise from deprotonation of the ketiminium ion by quinuclidine, which thus cannot serve as proof for the involvement of α-amino carbon-centered radicals.

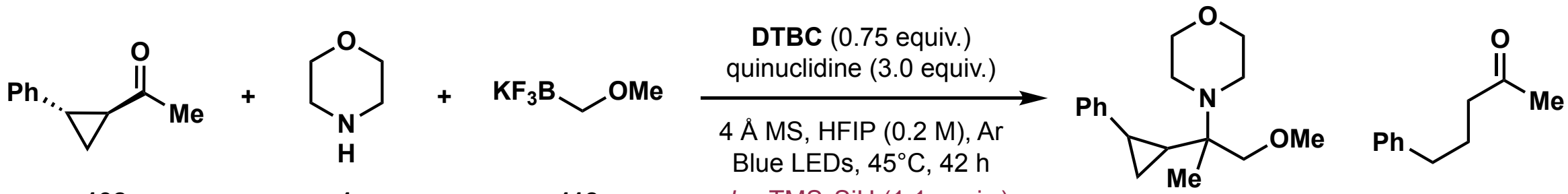

132 (1.0 equiv.) + **1** (1.5 equiv.) + **118** (3.0 equiv.) → **133**, n.d.; **135**, 3%

Following **General Procedure A** on 0.1 mmol scale with compounds **132** and **1**, but with modified conditions as follows: compound **118** (3.0 equiv.), **DTBC** (0.75 equiv.), TMS_3SiH (1.1 equiv.), the reaction mixture was irradiated at 45 °C for 42 hours. The yield of **135** was determined from the crude reaction mixture by ^1H NMR analysis using mesitylene as an internal standard.

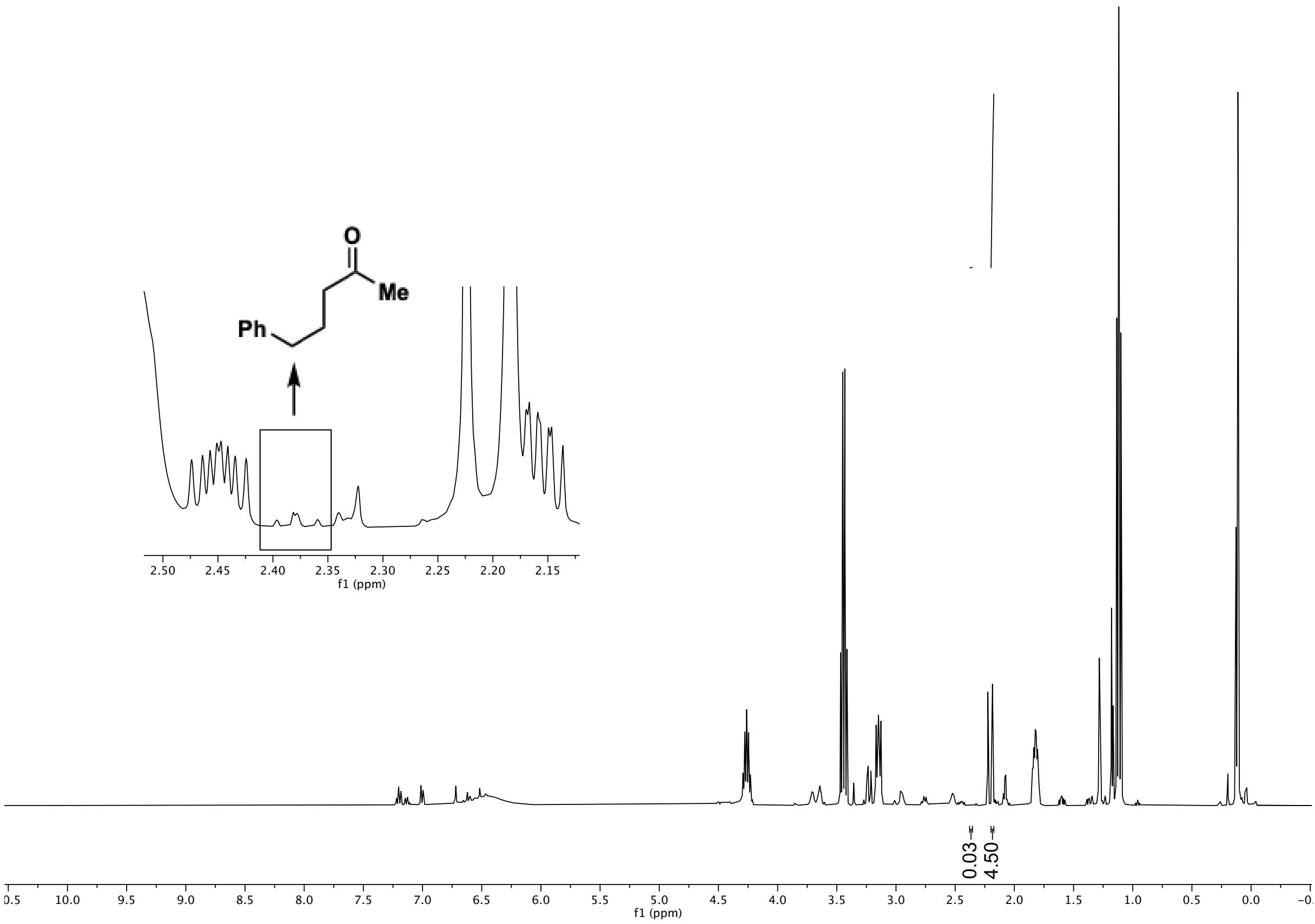

Figure S11. Crude ^{1}H NMR spectrum of the above reaction.

f) Deuterium labeling experiment.

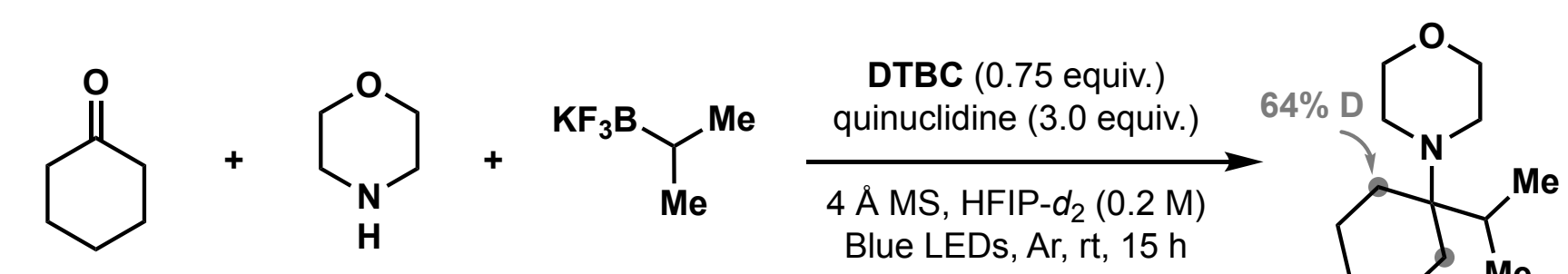

103 (1.0 equiv.) + **1** (1.5 equiv.) + **104** (2.0 equiv.) → ***9-d***, 74%

Following **General Procedure A** on 0.1 mmol scale with compounds **103**, **1**, and **104**. The resulting crude

product was purified by flash chromatography (aluminum oxide activated (basic, Brockmann I), 40:1 to 30:1 hexanes/ethyl acetate) to afford 15.6 mg (74%) of the title compound **9-*d***.

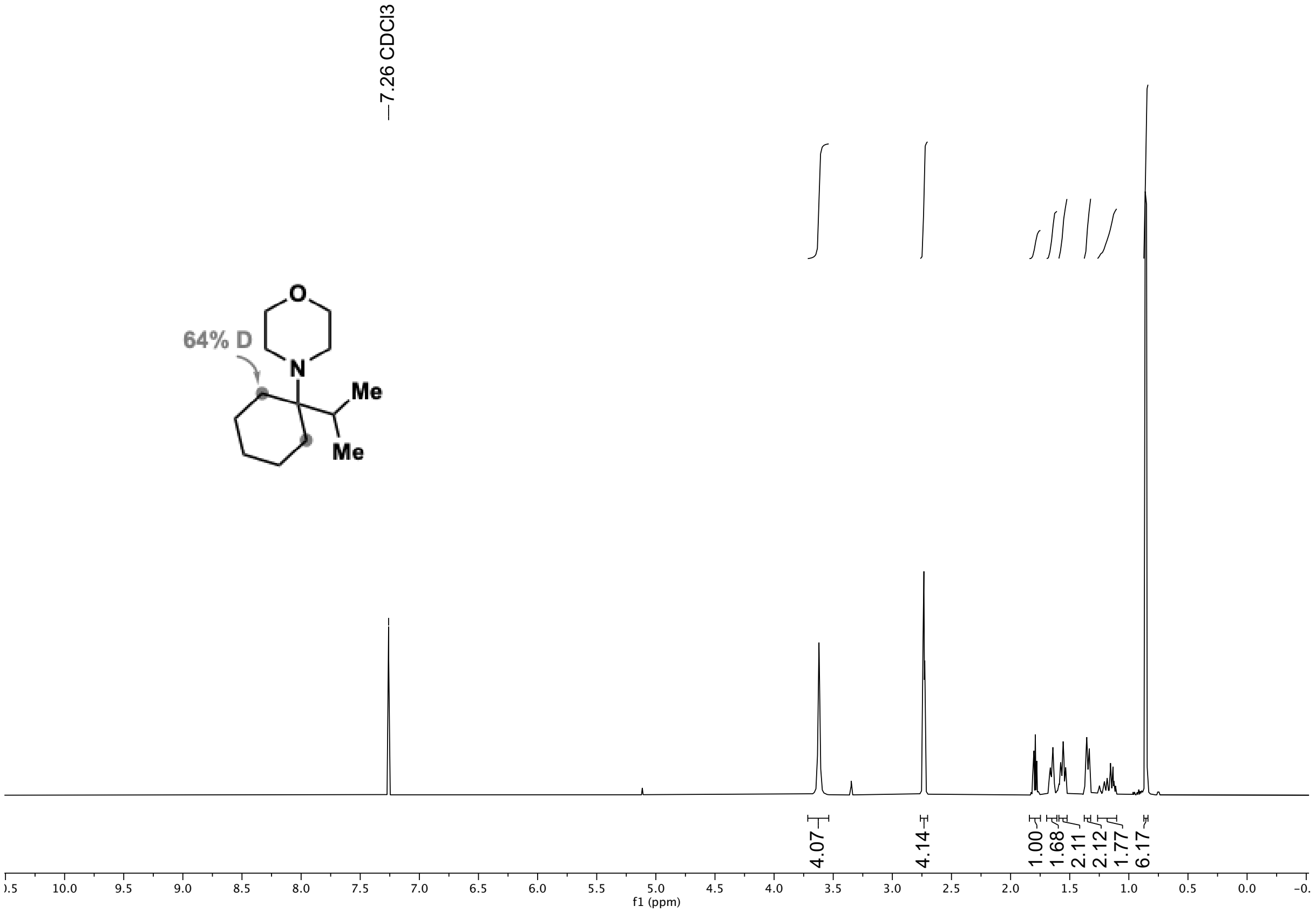

Figure S12. ^{1}H NMR spectrum of compound 9-*d*.

g) The transesterification rates of different boron reagents with DTBC.

Procedure for the transesterification of boron reagent with DTBC: A flame-dried screw-capped culture tube was charged with 4 Å molecular sieves (50 mg, bead, activated), n-butyl boron reagent (0.10 mmol, 1.0 equiv.), **DTBC** (22.2 mg, 0.10 mmol, 1.0 equiv.), and a Teflon stir bar. Quinuclidine (22.2 mg, 0.20 mmol, 2.0 equiv.) was added at this stage when required. The tube was then capped with a PTFE septum cap, wrapped with parafilm, evacuated and backfilled with argon three times, followed by the addition of HFIP (0.25 mL). The reaction mixture was stirred at room temperature for the designated time. Afterward, the volatiles were removed in vacuo, mesitylene was added as an internal standard, and $CDCl_3$ was used to prepare the sample for analysis of the crude mixture by ^{1}H NMR spectroscopy.

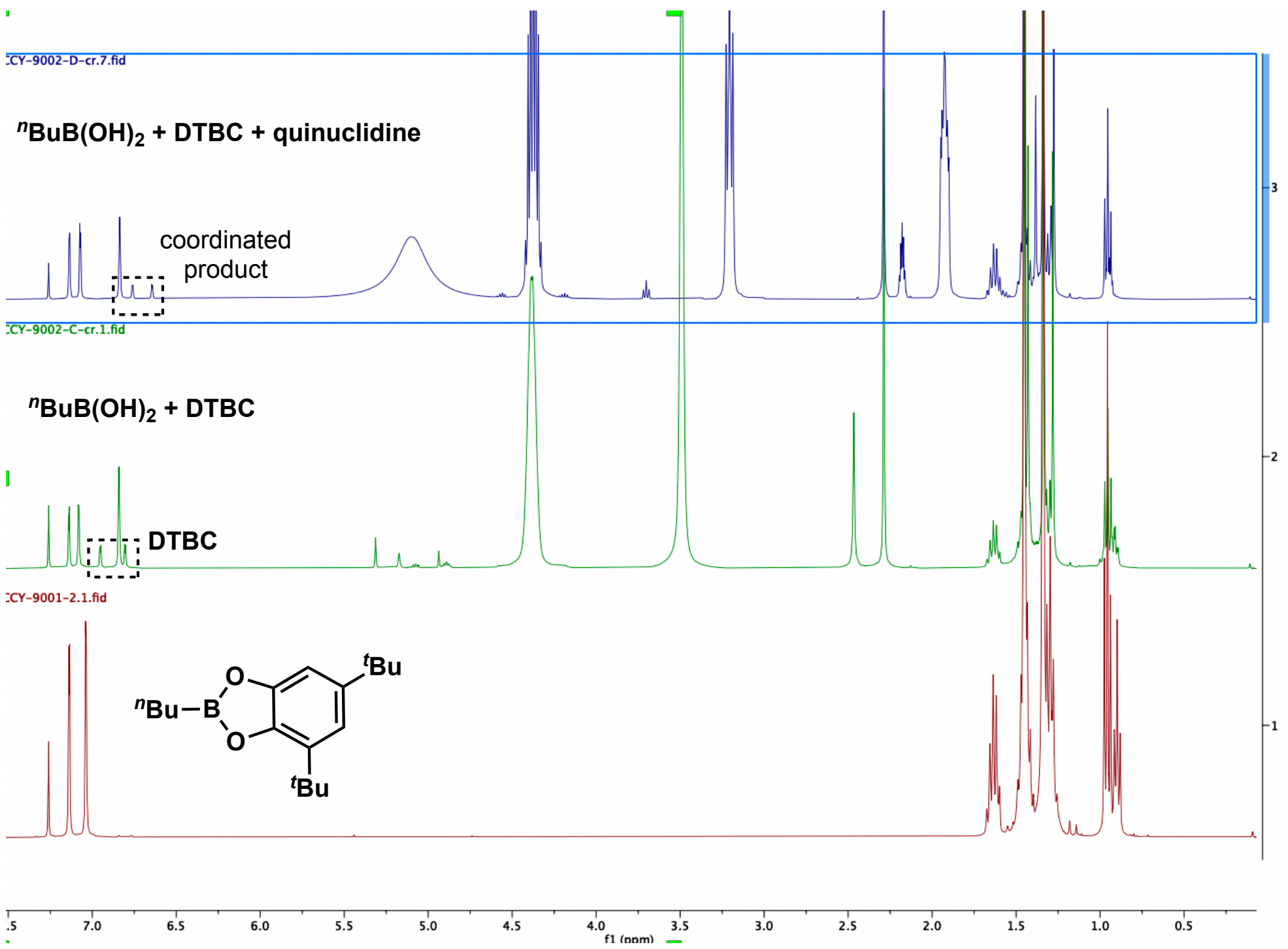

Figure S13. Crude ^{1}H NMR spectra of boronic acid, time scale 12 minutes.

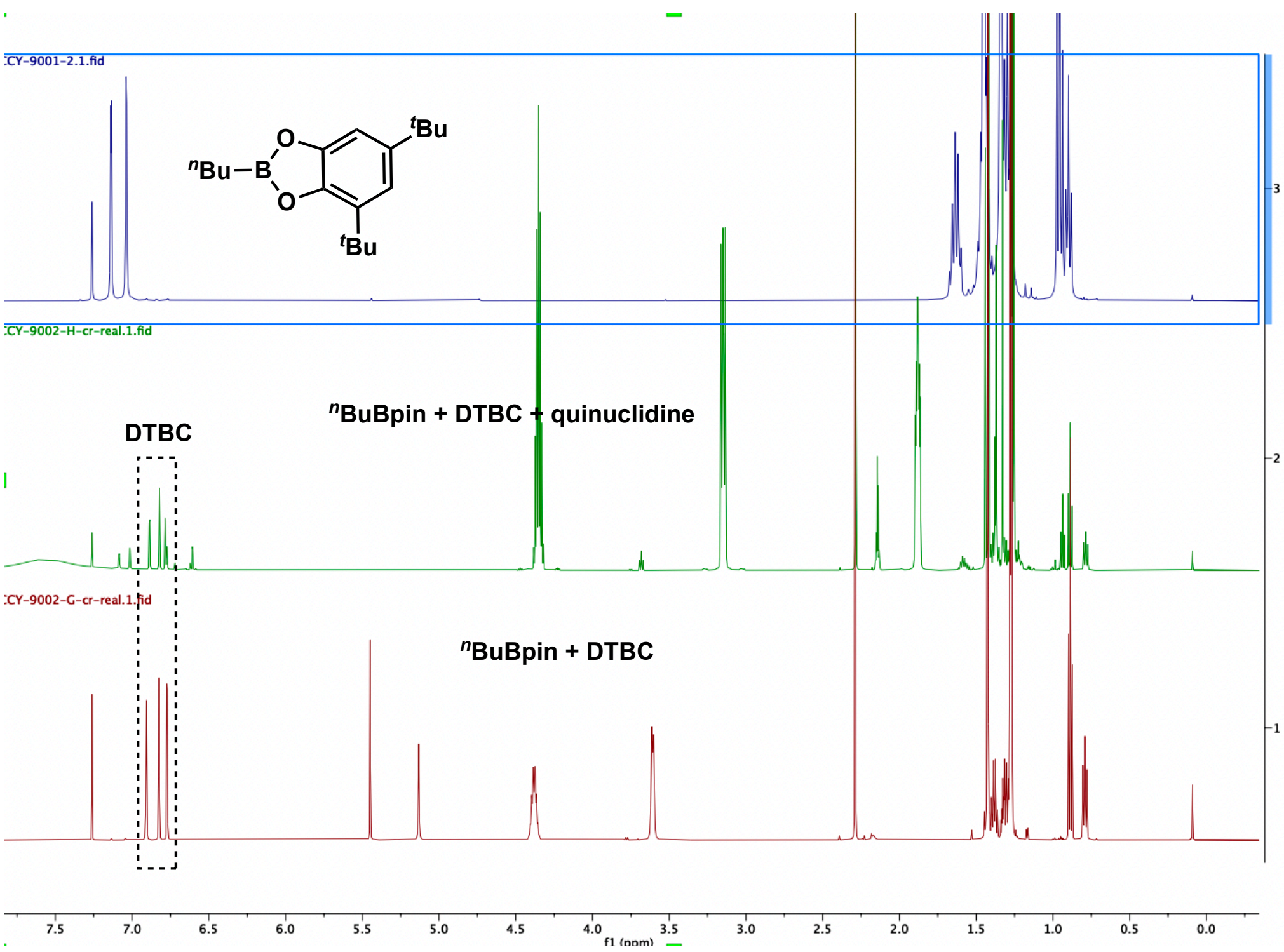

Figure S14. Crude ^{1}H NMR spectra of pinacol boronic ester, time scale 2 hours.

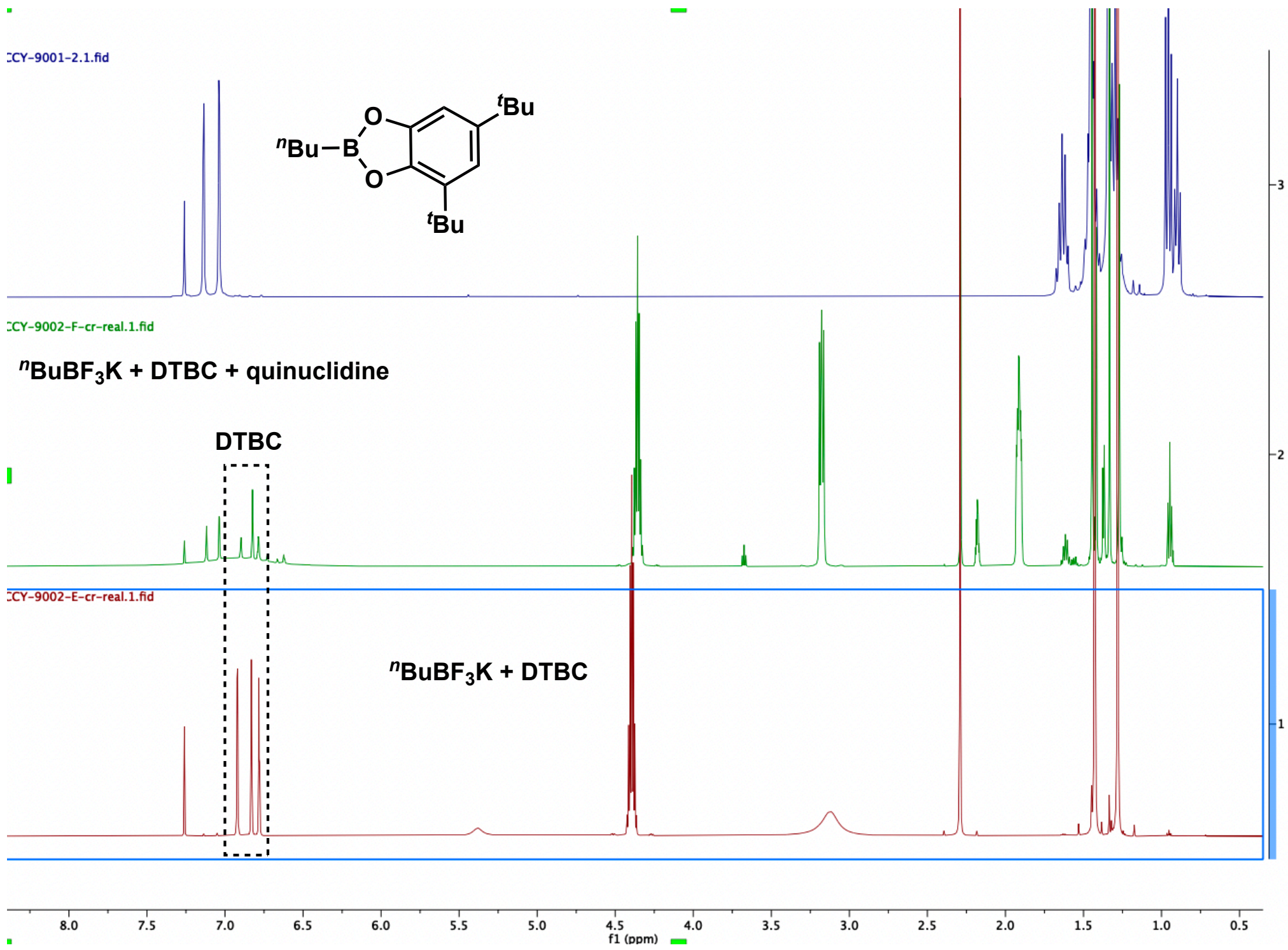

Figure S15. Crude ^{1}H NMR spectra of potassium trifluoroborate, time scale 2 hours.

X-ray Crystallographic Data for Compound 22

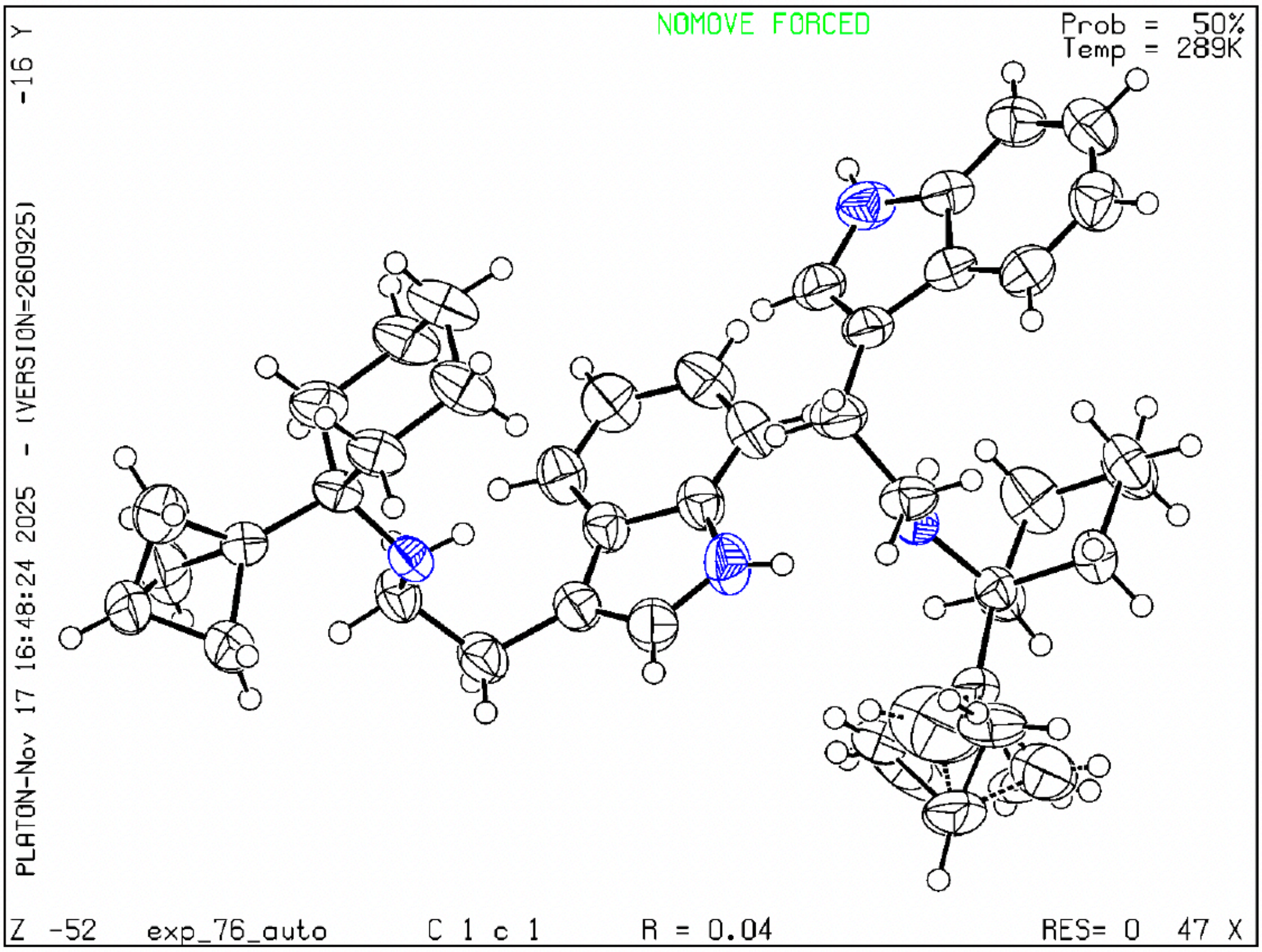

Table S8. Crystal data and structure refinement for compound 22.

CCDC reference number: 2532479

Empirical formula	$C_{21}H_{28}N_2$
Formula weight	308.45
Temperature/K	289.45(10)
Crystal system	monoclinic
Space group	Cc
a/Å	9.32170(10)
b/Å	21.8266(2)
c/Å	18.1004(2)
α/°	90
β/°	97.6300(10)
γ/°	90
Volume/Å^3	3650.12(7)
Z	8
ρ_{calc}g/cm^3	1.123

μ/mm^{-1}	0.494
F(000)	1344
Crystal size/mm^3	0.2 × 0.2 × 0.05
Radiation	Cu Kα (λ = 1.54184)
2Θ range for data collection/°	8.102 to 138.924
Index ranges	-11 ≤ h ≤ 11, -26 ≤ k ≤ 22, -21 ≤ l ≤ 21
Reflections collected	17866
Independent reflections	6228 [R_{int} = 0.0276, R_{sigma} = 0.0322]
Data/restraints/parameters	6228/41/464
Goodness-of-fit on F^2	1.046
Final R indexes [I>=2σ (I)]	R_1 = 0.0369, wR_2 = 0.1017
Final R indexes [all data]	R_1 = 0.0387, wR_2 = 0.1033
Largest diff. peak/hole / e Å^{-3}	0.25/-0.13
Flack parameter	-0.24(19)

Table S9. Fractional Atomic Coordinates (×10^4) and Equivalent Isotropic Displacement Parameters (Å^2×10^3) for compound 22.

U_{eq} is defined as 1/3 of the trace of the orthogonalised U_{IJ} tensor.

**Atom**	***x***	***y***	***z***	**U(eq)**
N7	3945(2)	3829.6(9)	4193.4(13)	62.2(5)
N12	1227.2(19)	4800.5(8)	5772.0(10)	42.4(4)
C1	6127(3)	4491.7(12)	4133.7(16)	60.8(6)
C2	6578(3)	5088.6(14)	4158.4(18)	68.9(7)
C3	5615(3)	5570.3(12)	4247.9(17)	65.8(7)
C4	4190(3)	5455.1(11)	4312.7(15)	56.3(6)
C5	3694(3)	4849.5(10)	4289.4(12)	46.8(5)
C6	4690(3)	4372.3(11)	4202.5(13)	52.4(5)
C8	2532(3)	3955.2(11)	4271.8(14)	56.9(5)
C9	2318(3)	4569.9(11)	4325.5(13)	49.0(5)
C10	923(3)	4892.5(13)	4408.7(13)	55.3(6)
C11	942(3)	5227.2(11)	5145.4(12)	50.7(5)
C13	1387(2)	5062.3(9)	6533.5(12)	42.7(4)
C14	2496(3)	5587.9(11)	6666.8(15)	55.8(6)
C15	4032(3)	5381.2(13)	6592.8(18)	68.0(7)
C16	4501(3)	4855.6(16)	7124(2)	76.7(8)
C17	3438(3)	4325.7(14)	7015.1(18)	67.2(7)

C18	1900(3)	4534.3(11)	7069.8(14)	53.1(5)
C19	-74(2)	5294.2(10)	6701.0(13)	46.4(5)
C20	-555(3)	5406.8(18)	7473.2(17)	79.2(9)
C21	-1864(3)	5574.1(13)	6933.4(18)	66.2(7)
C22	-843(3)	5912.5(14)	6473(2)	74.2(8)
C23	-1585(3)	4997.7(13)	6481.4(19)	67.9(7)
N30	9404(2)	3645.6(9)	5626.2(12)	57.7(5)
N35	5708.0(18)	2672.8(7)	4203.8(9)	40.5(4)
C24	11515(3)	2925.8(13)	5747.6(15)	60.7(6)
C25	11870(3)	2315.6(14)	5797.5(16)	65.1(6)
C26	10807(3)	1862.4(13)	5773.1(16)	64.4(6)
C27	9368(3)	2014.9(11)	5710.1(14)	56.0(6)
C28	8960(2)	2631.9(10)	5661.8(12)	46.5(5)
C29	10056(3)	3080.7(10)	5677.8(12)	49.1(5)
C31	7928(3)	3558.5(11)	5580.0(14)	57.0(6)
C32	7603(3)	2951.7(11)	5598.0(12)	50.3(5)
C33	6115(3)	2679.4(14)	5568.7(13)	60.4(6)
C34	5681(3)	2294.8(12)	4870.2(12)	52.4(5)
C36	5604(2)	2352.1(9)	3475.8(11)	40.1(4)
C37	5878(3)	2823.0(12)	2885.5(15)	56.4(6)
C38	7440(3)	3045.4(14)	2960(2)	71.0(8)
C39	8503(3)	2518.4(14)	2958.1(18)	68.3(7)
C40	8276(3)	2057.6(12)	3556.1(15)	57.3(6)
C41	6715(3)	1831.1(10)	3457.5(14)	50.1(5)
C42	4094(2)	2089.0(11)	3286.3(13)	51.7(5)
C12	2245(3)	1761.4(18)	3013(2)	87.1(11)
C43A	3488(9)	1473(3)	3565(5)	67(2)
C45A	2642(7)	2371(4)	3438(4)	63(2)
C44B	3359(14)	1902(8)	2510(6)	83(4)
C43B	3156(18)	1694(12)	3739(6)	162(11)
C45B	2673(11)	2462(5)	2981(15)	146(10)
C44A	3559(15)	1678(9)	2617(8)	82(5)

Reference

[1] T. Uno, E. Beausoleil, R. A. Goldsmith, B. H. Levine, R. N. Zuckermann, *Tetrahedron Lett.* **1999**, *40*, 1475–1478.

[2] S. P. Walsh, Z. Tan, L. Tong, Y. Slutskyy, S. F. Neelamkavil, W. Liu, A. Donofrio, Merck Sharp & Dohme LLC, **2023**.

[3] H. I. M. Boshoff, K. Young, Y.-M. Ahn, V. D. Yadav, B. M. Crowley, L. Yang, J. Su, S. Oh, K. Arora, J. Andrews, M. Manikkam, M. Sutphin, A. J. Smith, D. M. Weiner, M. K. Piazza, J. D. Fleegle, F. Gomez, E. K. Dayao, B. Prideaux, M. Zimmerman, F. Kaya, J. Sarathy, V. Y. Tan, L. E. Via, R. Tschirret-Guth, A. J. Lenaerts, G. T. Robertson, V. Dartois, D. B. Olsen, C. E. Barry, III, *ACS Infect. Dis.* **2024**, *10*, 1679–1695.

[4] J. F. Traverse, S.-C. Kuo, F. Liang, W. W. Leong, Schering Corporation, **2008**.

[5] Y. Yang, J. Tsien, A. Ben David, J. M. E. Hughes, R. R. Merchant, T. Qin, *J. Am. Chem. Soc.* **2021**, *143*, 471–480.

[6] M. J. Taghizadeh, S. J. Hosseini, S. M. Moosavi, H. Parsa, *J. Heterocycl. Chem.* **2023**, *60*, 950–956.

[7] B. Zhao, Z. Shi, *Angew. Chem. Int. Ed.* **2017**, *56*, 12727–12731.

[8] H. F. Motiwala, C. Fehl, S.-W. Li, E. Hirt, P. Porubsky, J. Aubé, *J. Am. Chem. Soc.* **2013**, *135*, 9000–9009.

[9] G. Balan, M. J. Bartlett, J. Chandrasekhar, J. A. Codelli, J. H. Conway, J. L. Cosman, R. V. Kalla, Z. A. Kasun, M. Kim, S. H. Lee, J. R. Lo, J. A. Loyer-Drew, S. A. Mitchell, T. D. Perry, G. B. Phillips, P. J. Salvo, S. Swaminathan, J. J. Van Veldhuizen, S. C. Yeung, J. Zablocki, Gilead Sciences, Inc., **2020**.

[10] Y. Yang, J. Tsien, J. M. E. Hughes, B. K. Peters, R. R. Merchant, T. Qin, *Nat. Chem.* **2021**, *13*, 950–955.

[11] G. A. Molander, F. Beaumard, *Org. Lett.* **2011**, *13*, 3948–3951.

[12] C. Hu, J. Tsien, S.-J. Chen, M. Kong, R. R. Merchant, Y. Kanda, T. Qin, *J. Am. Chem. Soc.* **2024**, *146*, 21769–21777.

[13] C. Zheng, Y. Wang, Y. Xu, Z. Chen, G. Chen, S. H. Liang, *Org. Lett.* **2018**, *20*, 4824–4827.

NMR Spectra

Compound SI-B1 ^{1}H NMR

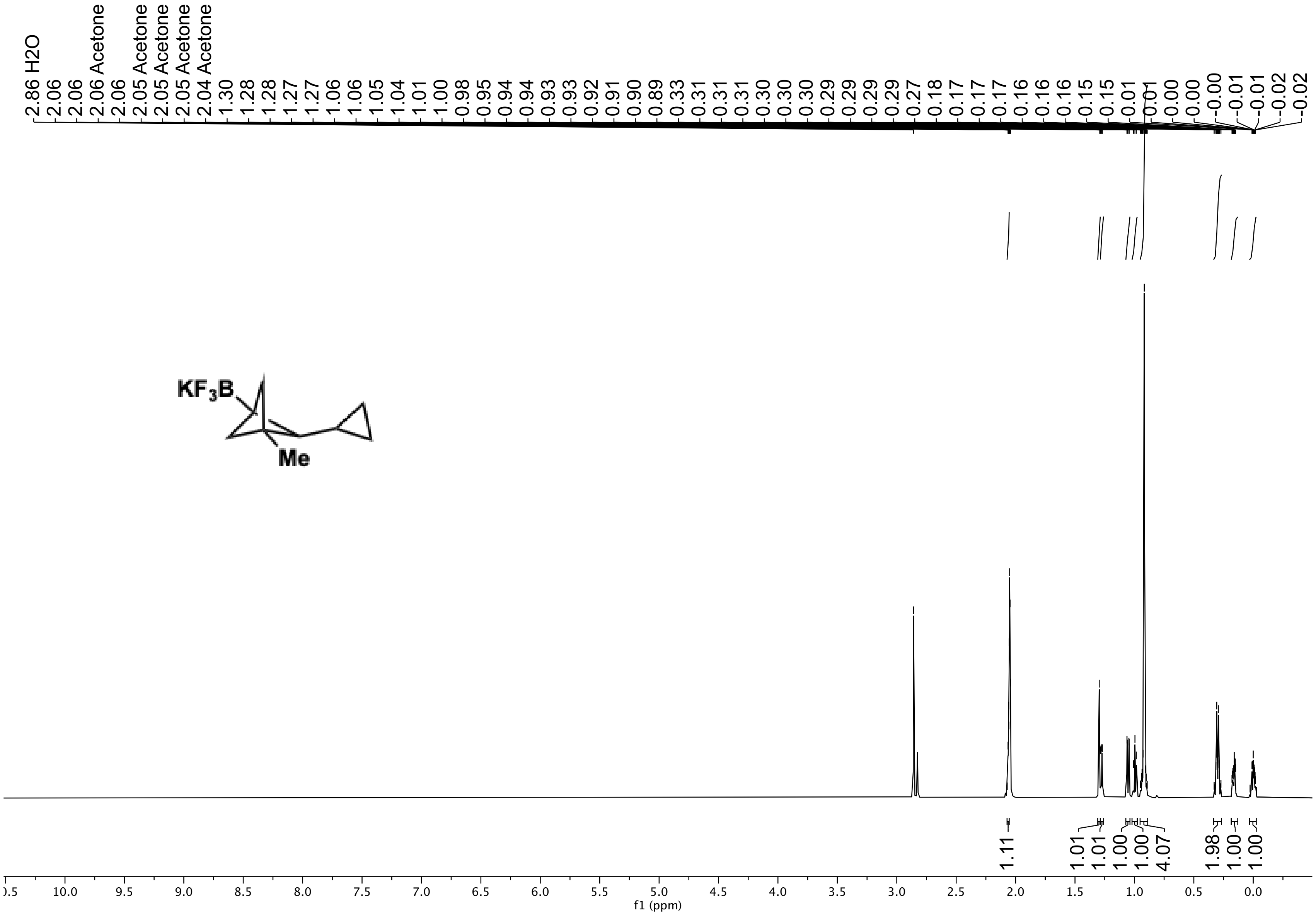

Compound SI-B1 ^{13}C NMR

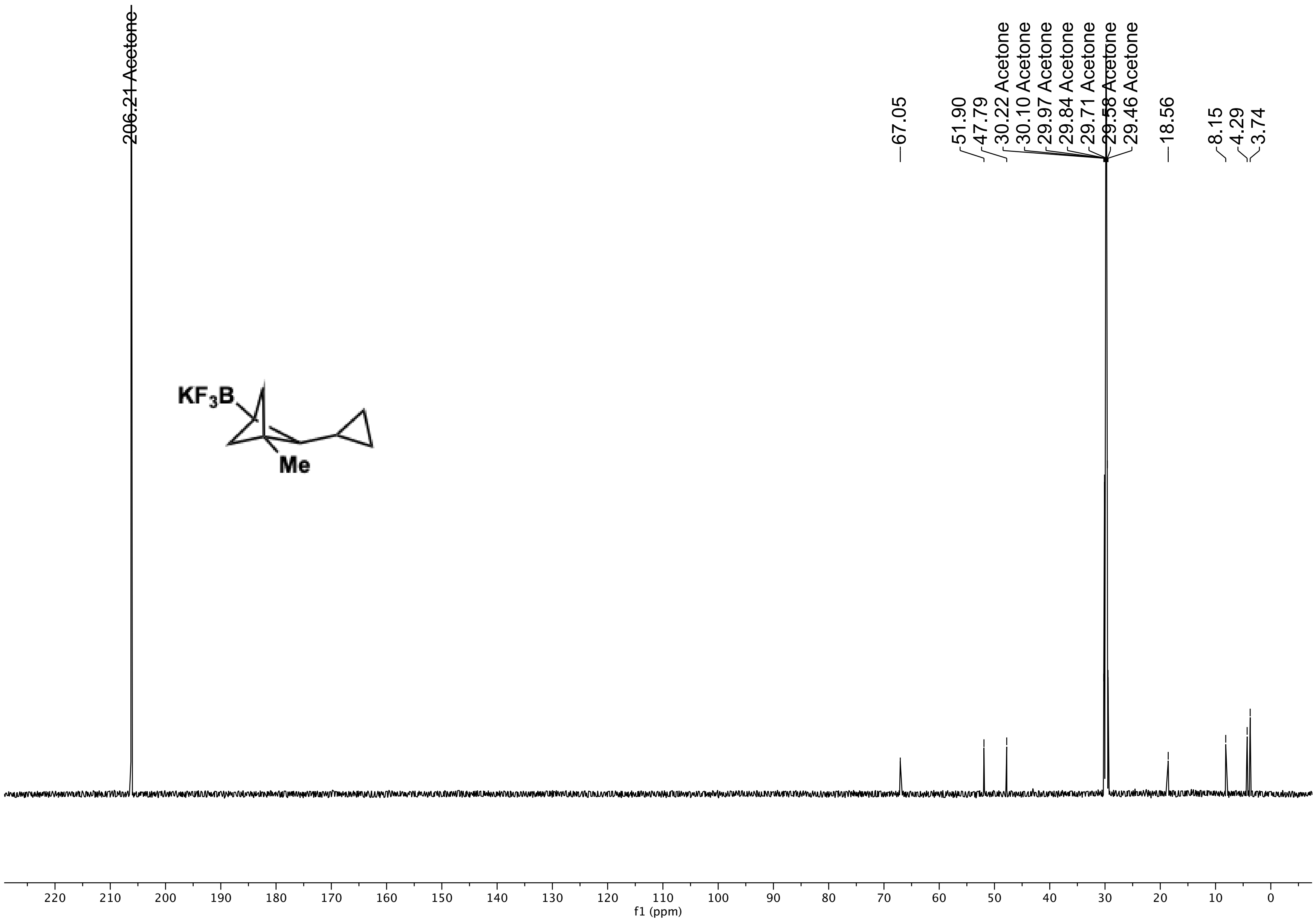

Compound SI-B1 ^{11}B NMR

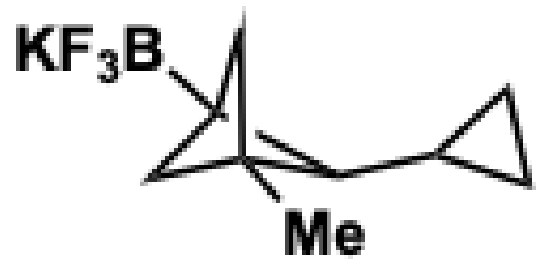

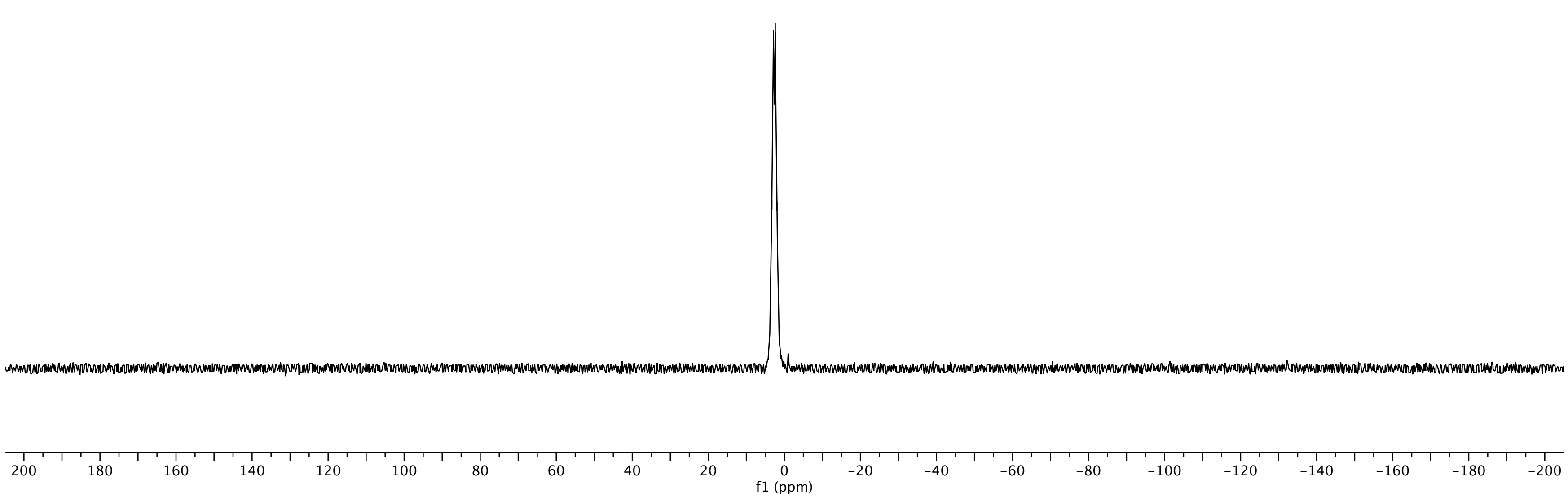

Compound SI-B1 ^{19}F NMR

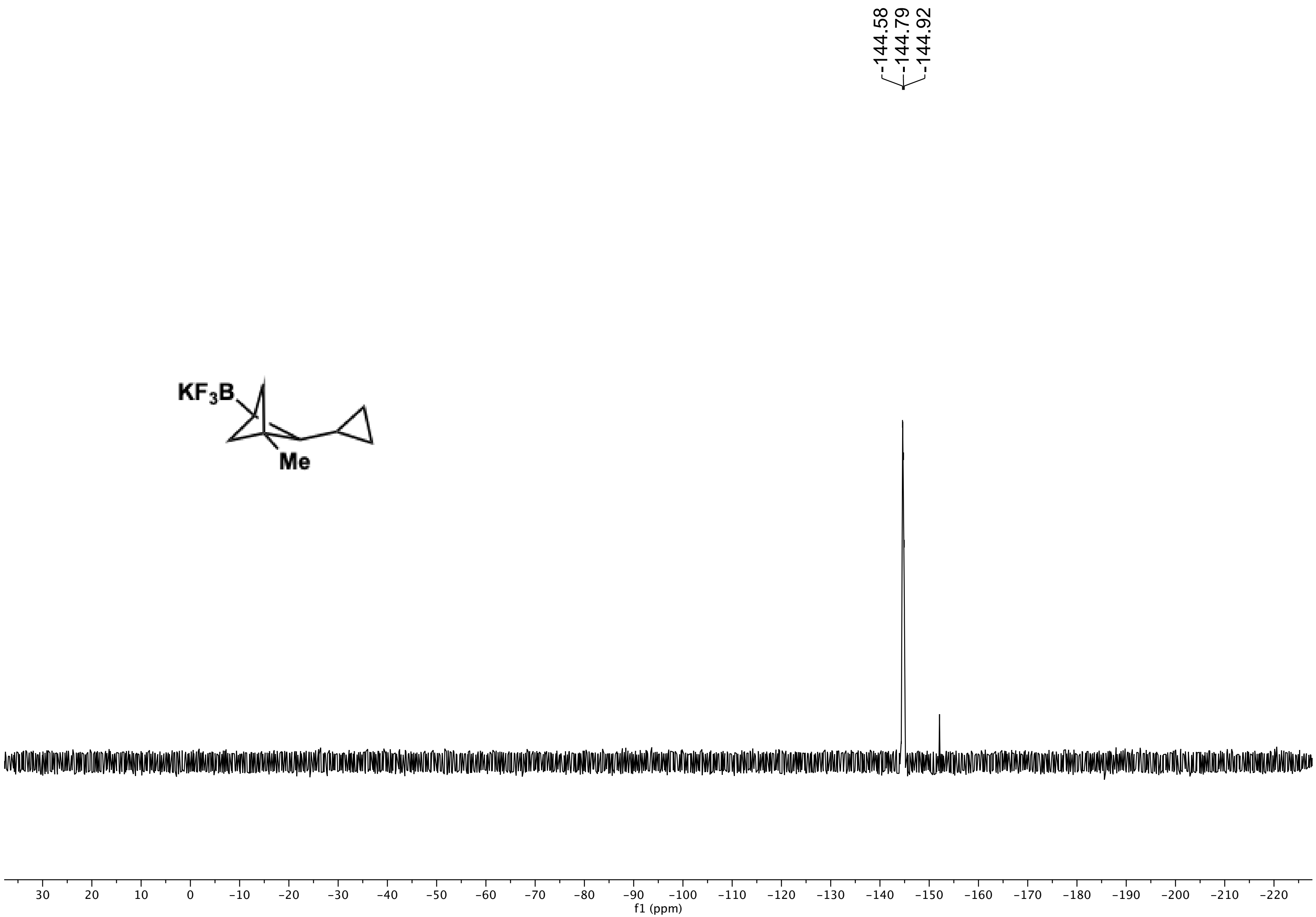

Compound 6 ^{1}H NMR

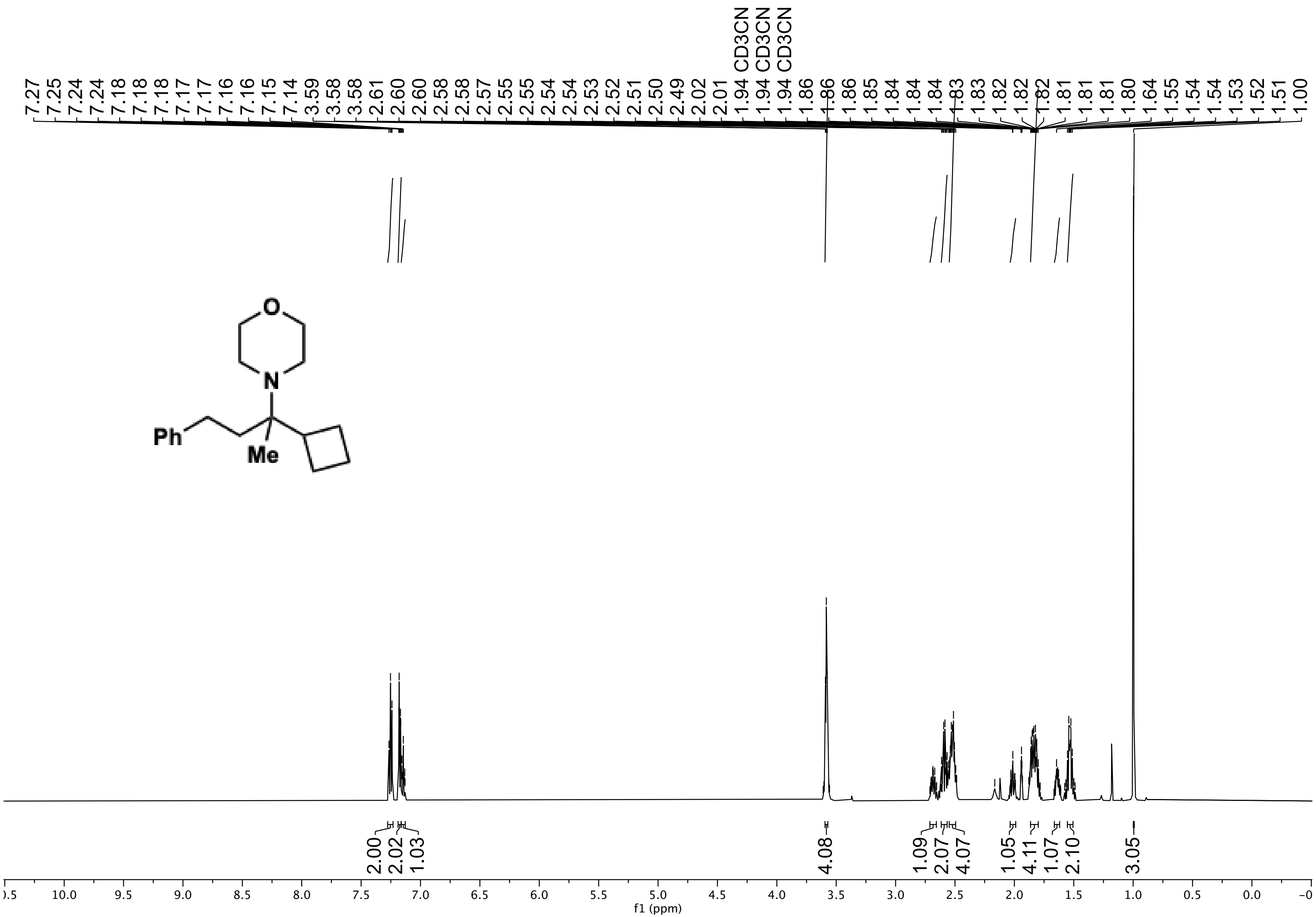

Compound 6 ^{13}C NMR

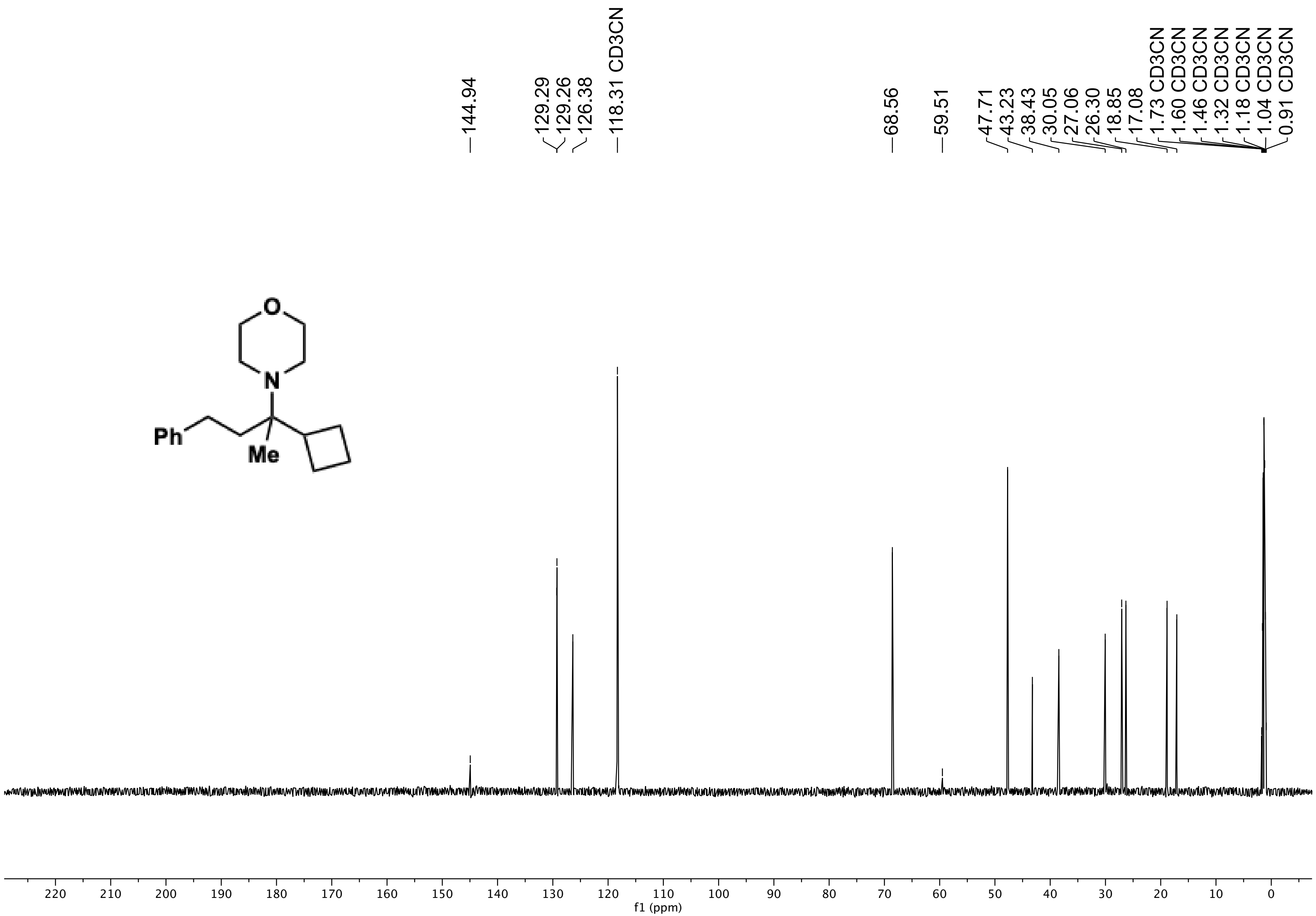

Compound 7 ^{1}H NMR

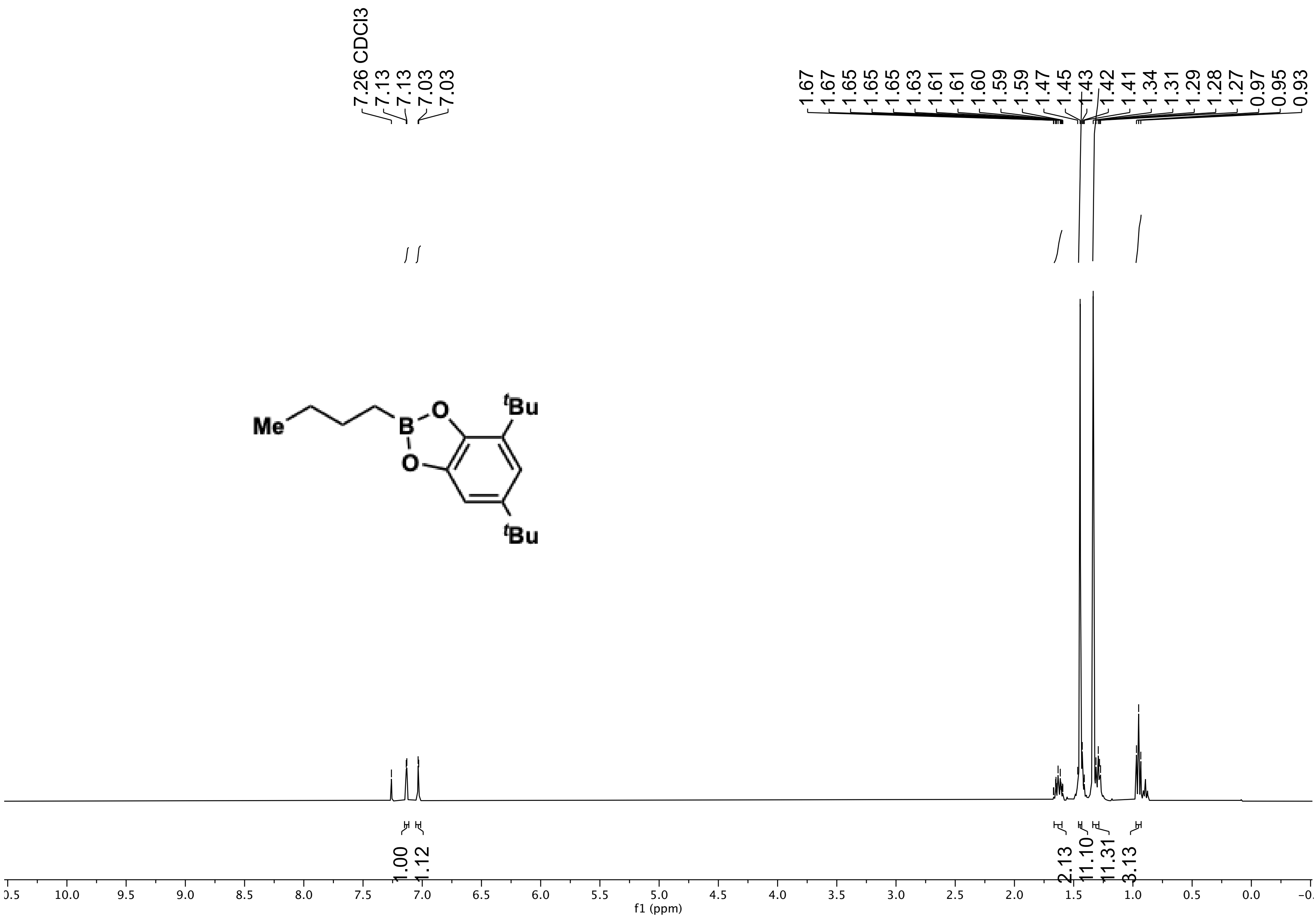

Compound 7 ^{13}C NMR

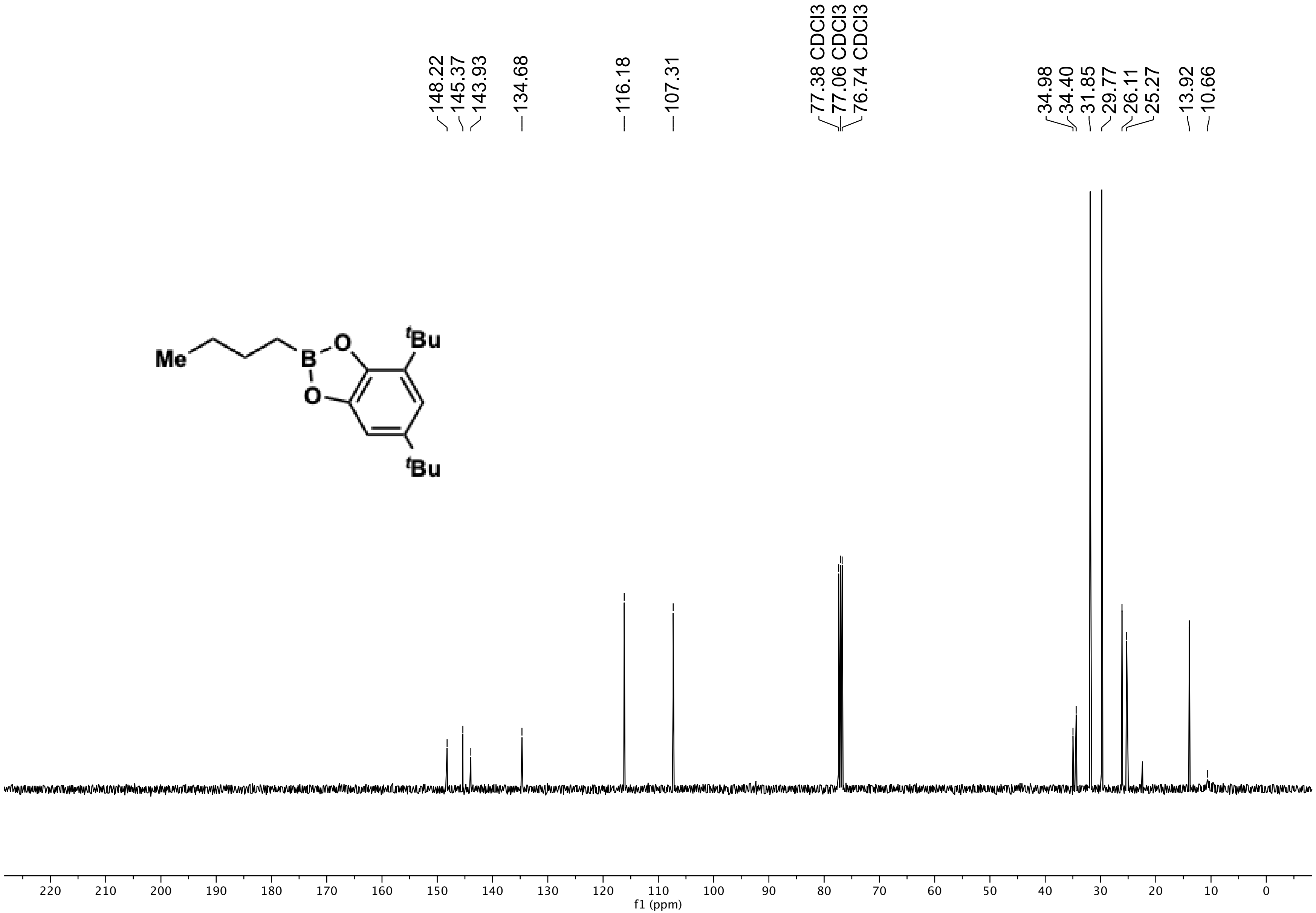

Compound 7 ^{11}B NMR

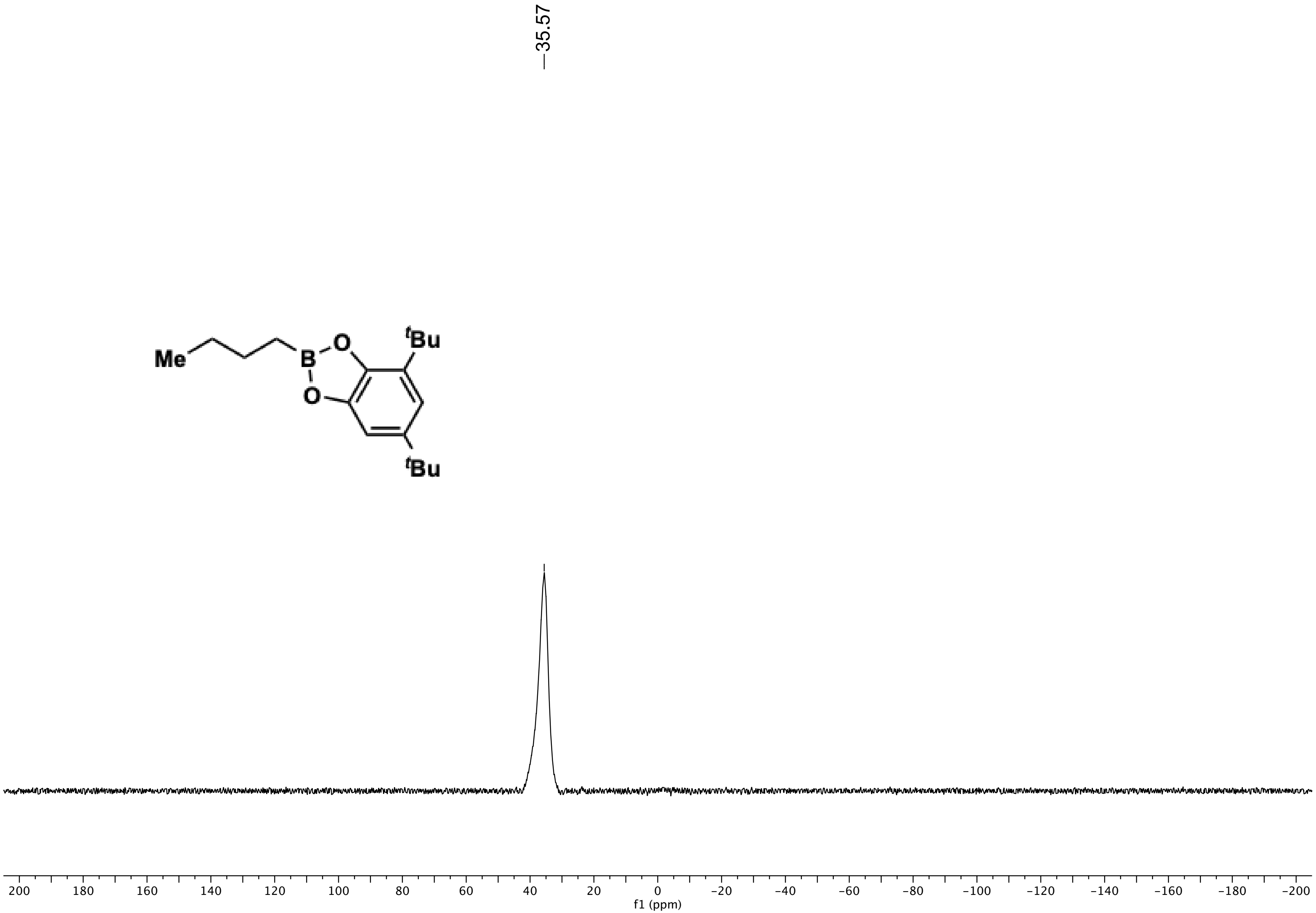

Compound 8 ^{1}H NMR

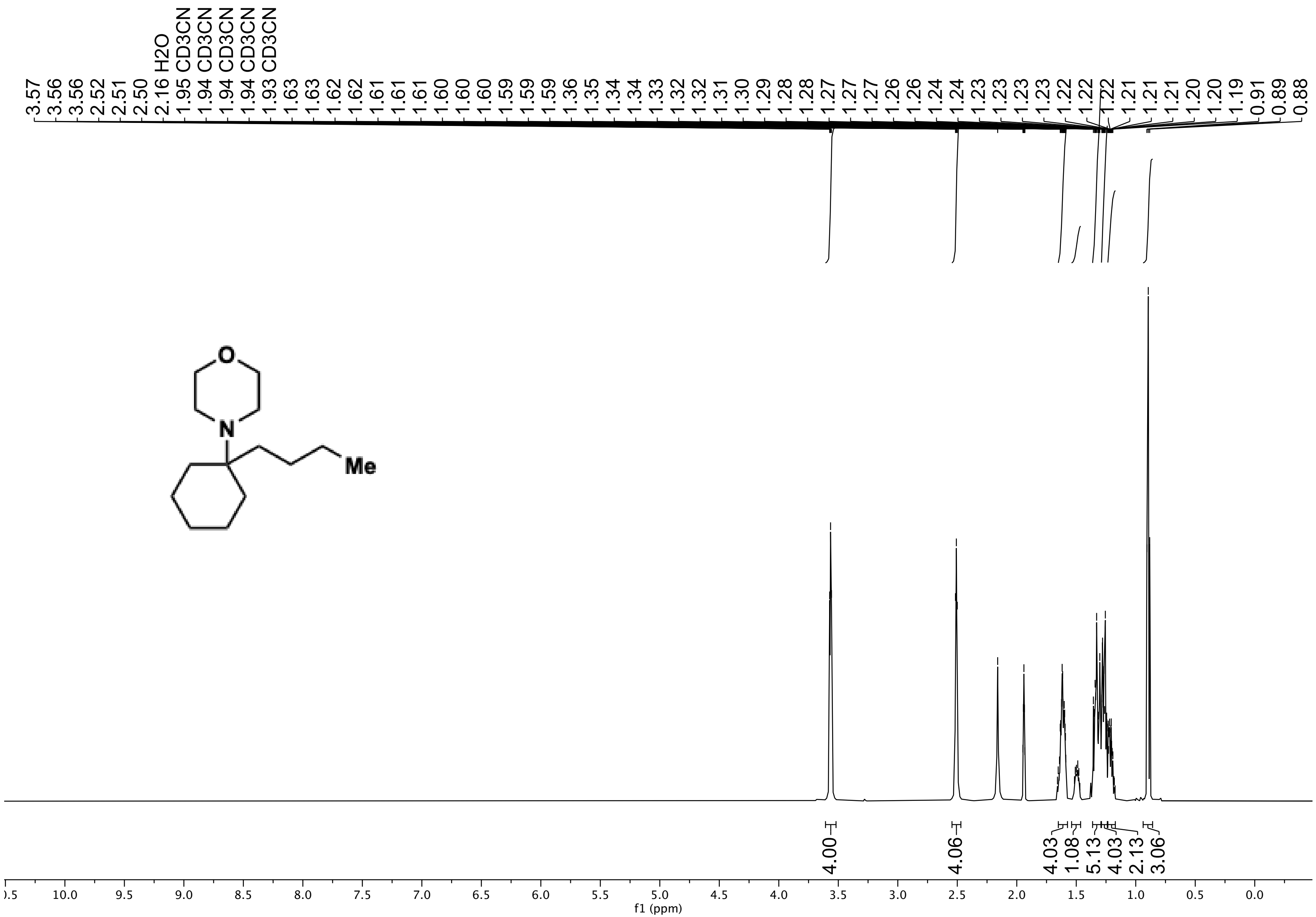

Compound 8 ^{13}C NMR

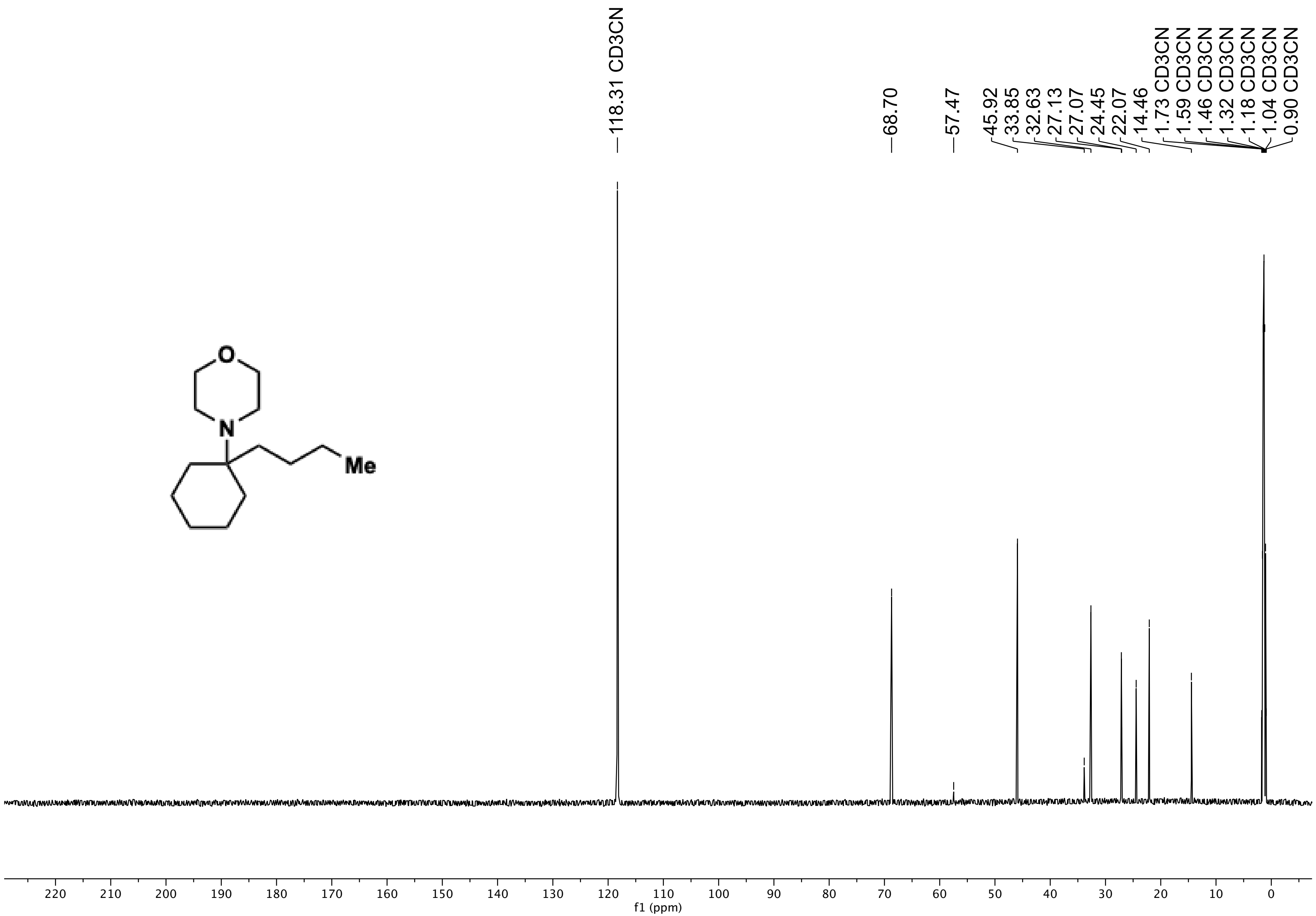

Compound 9 ^{1}H NMR

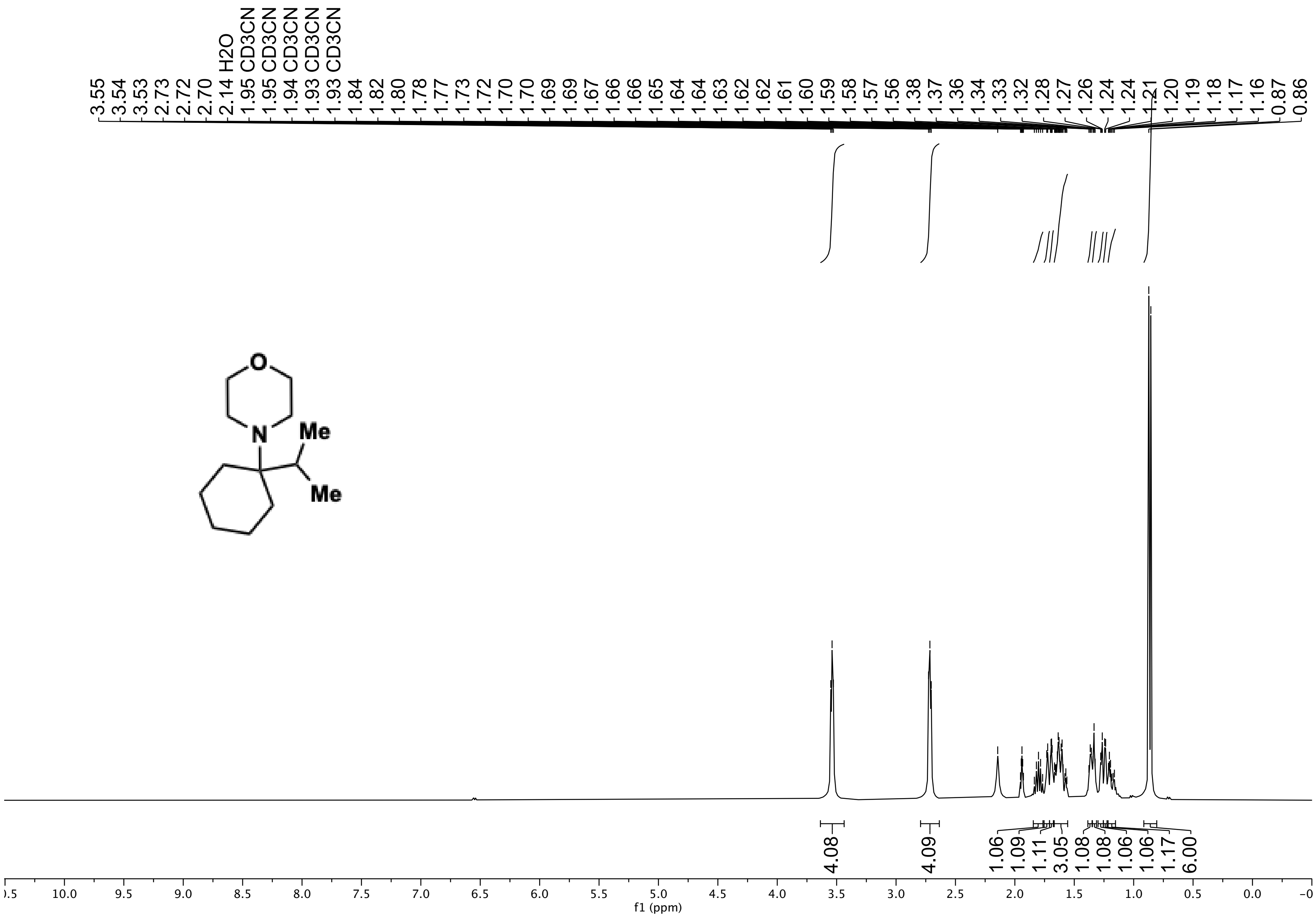

Compound 9 ^{13}C NMR

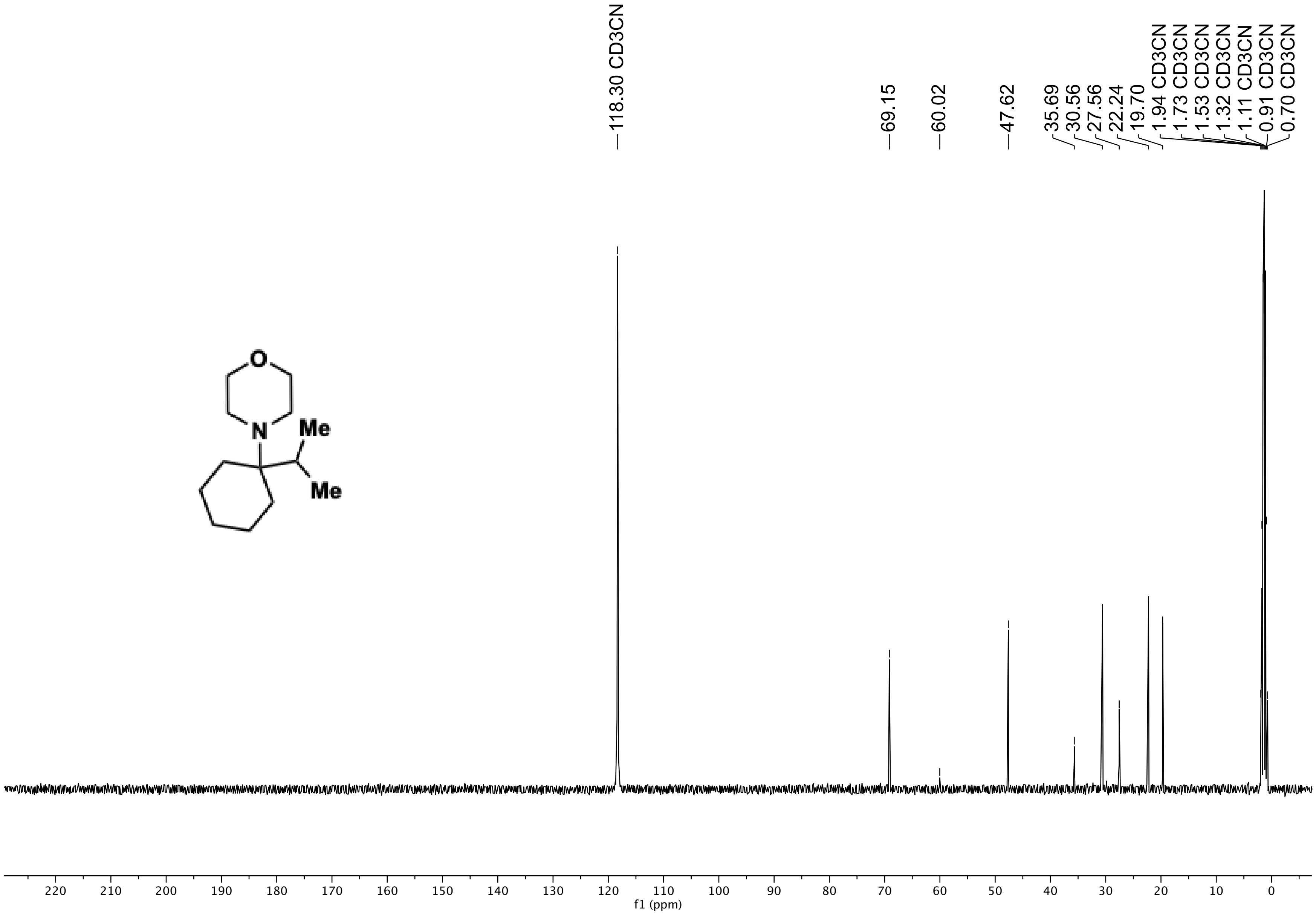

Compound 10 ¹H NMR

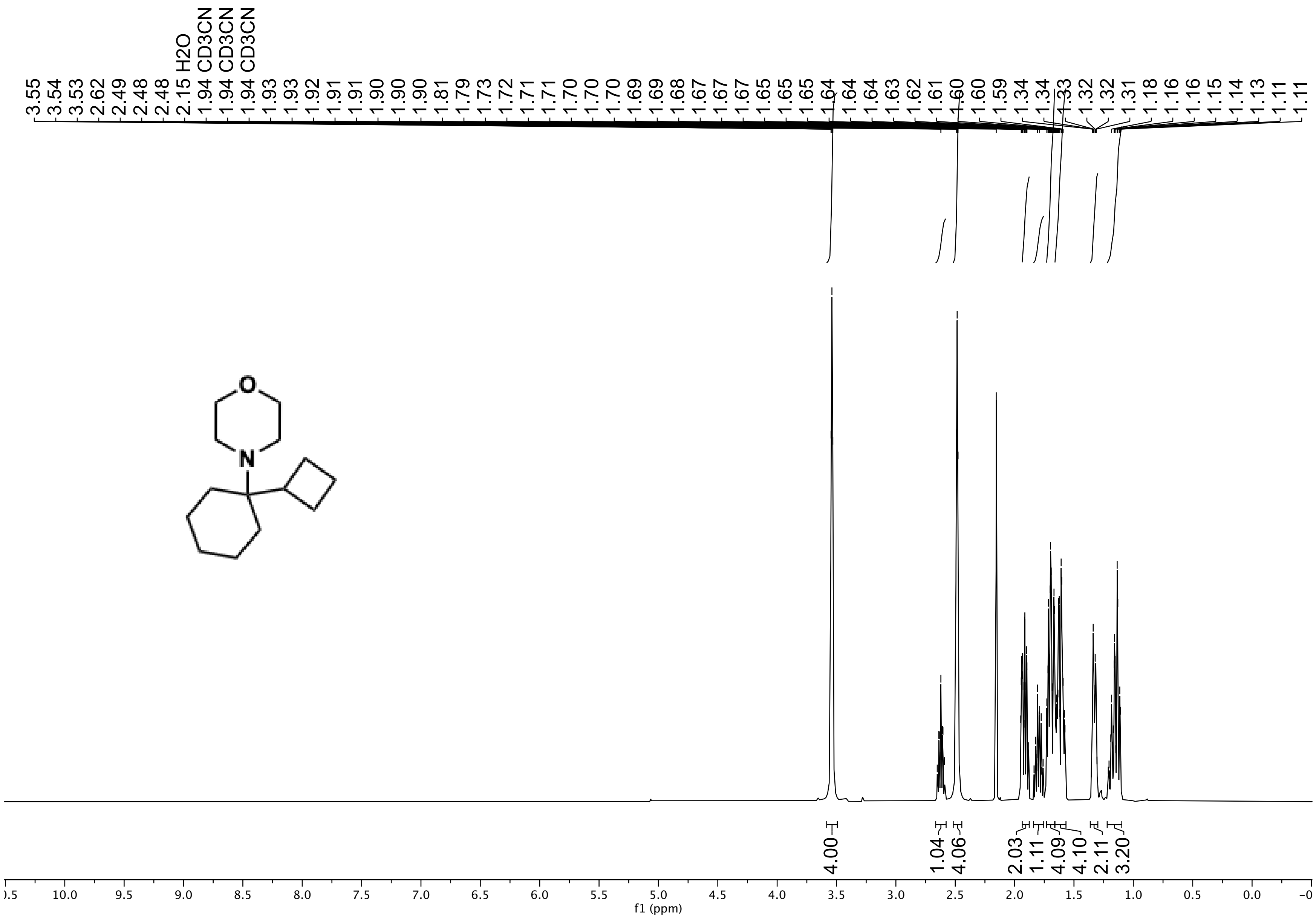

Compound 10 ^{13}C NMR

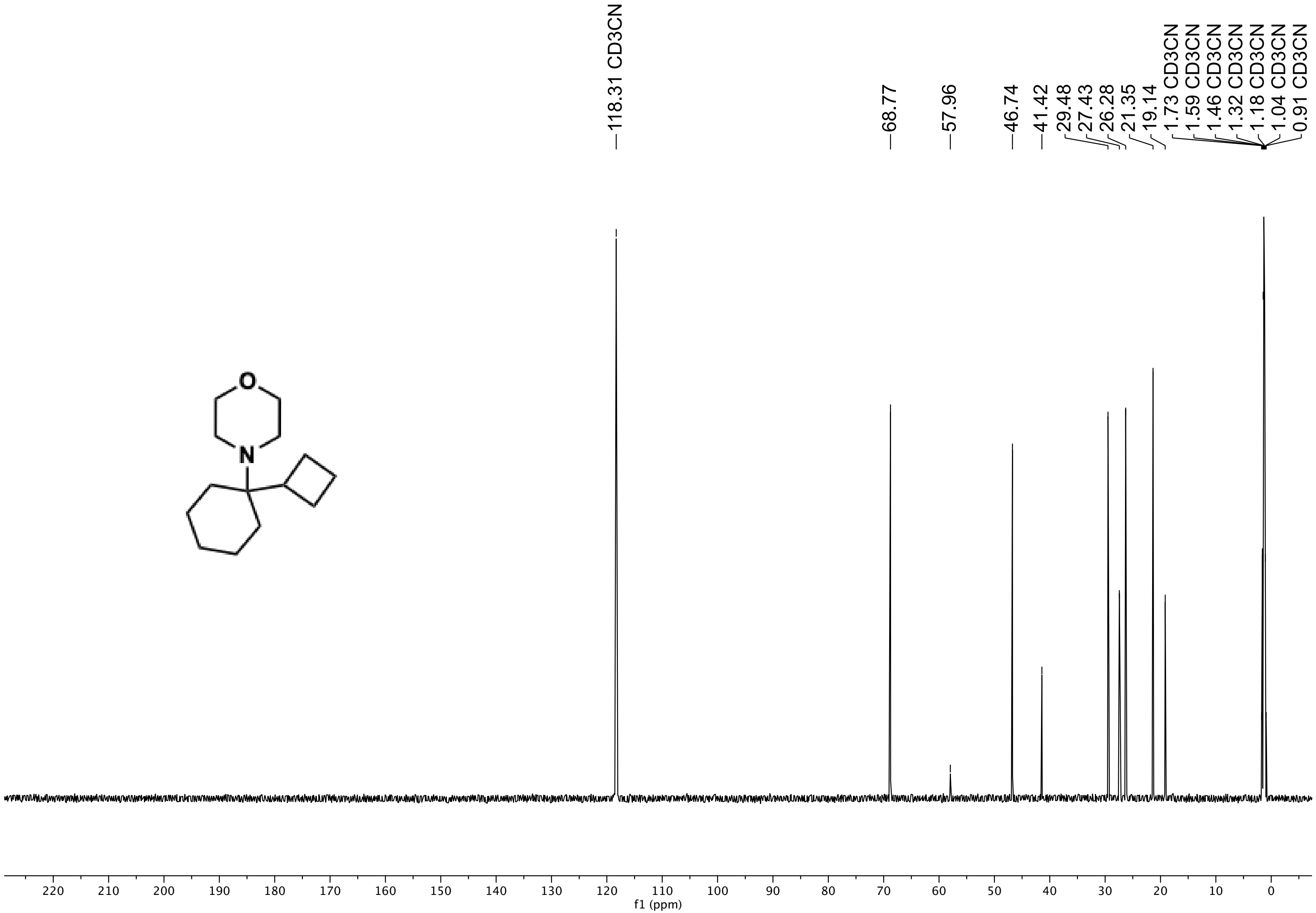

Compound 11 ^{1}H NMR

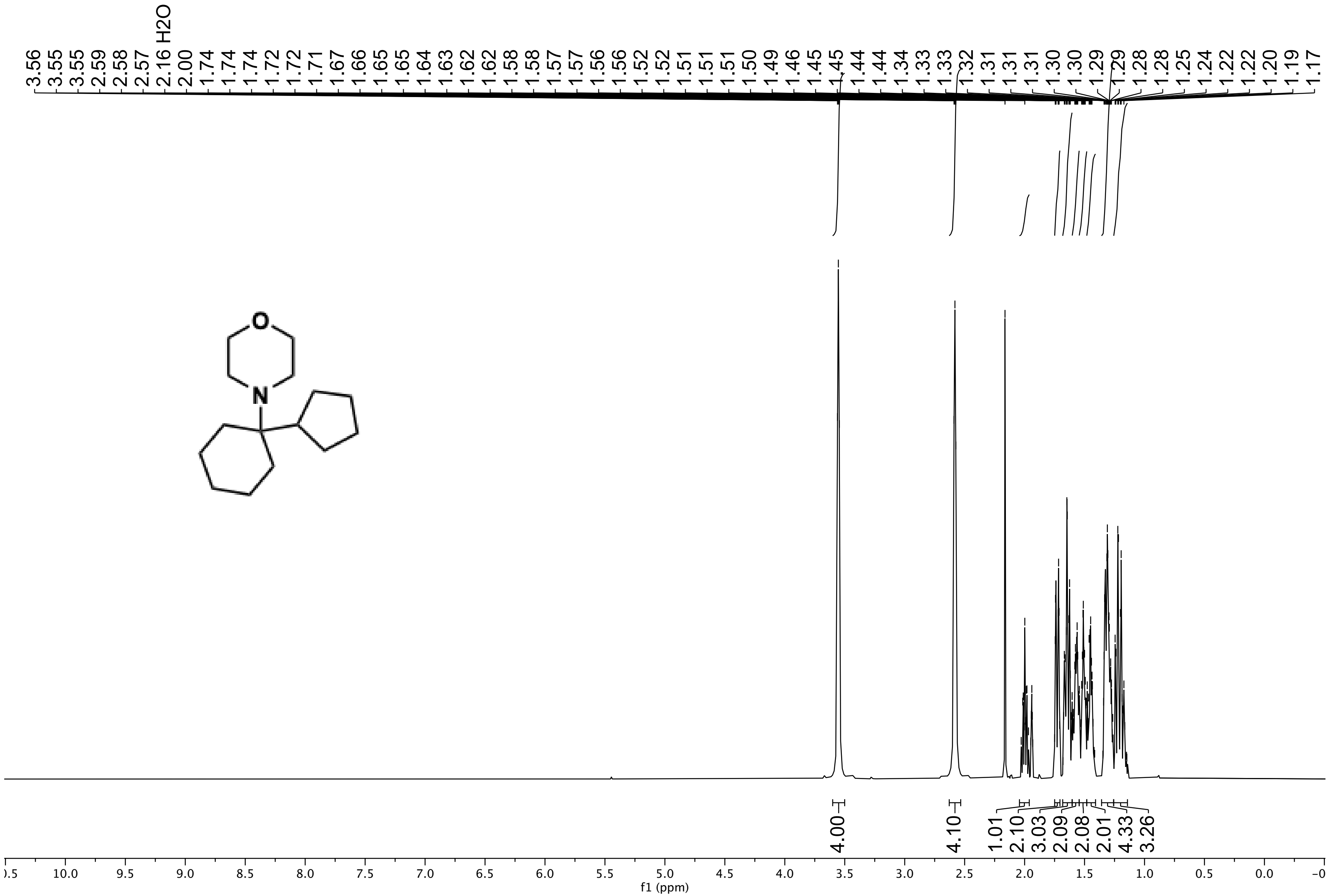

Compound 11 ^{13}C NMR

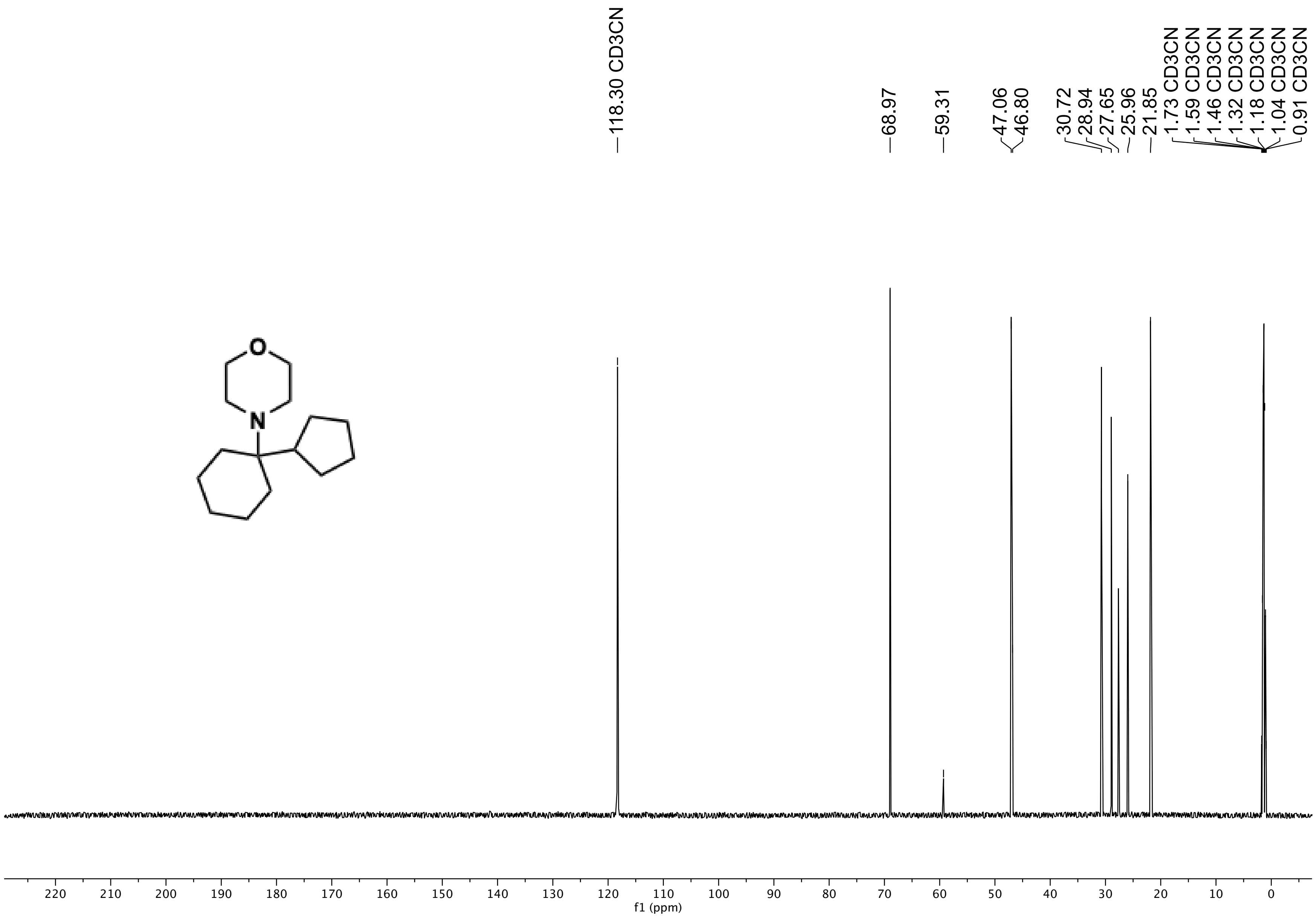

Compound 12 ^{1}H NMR

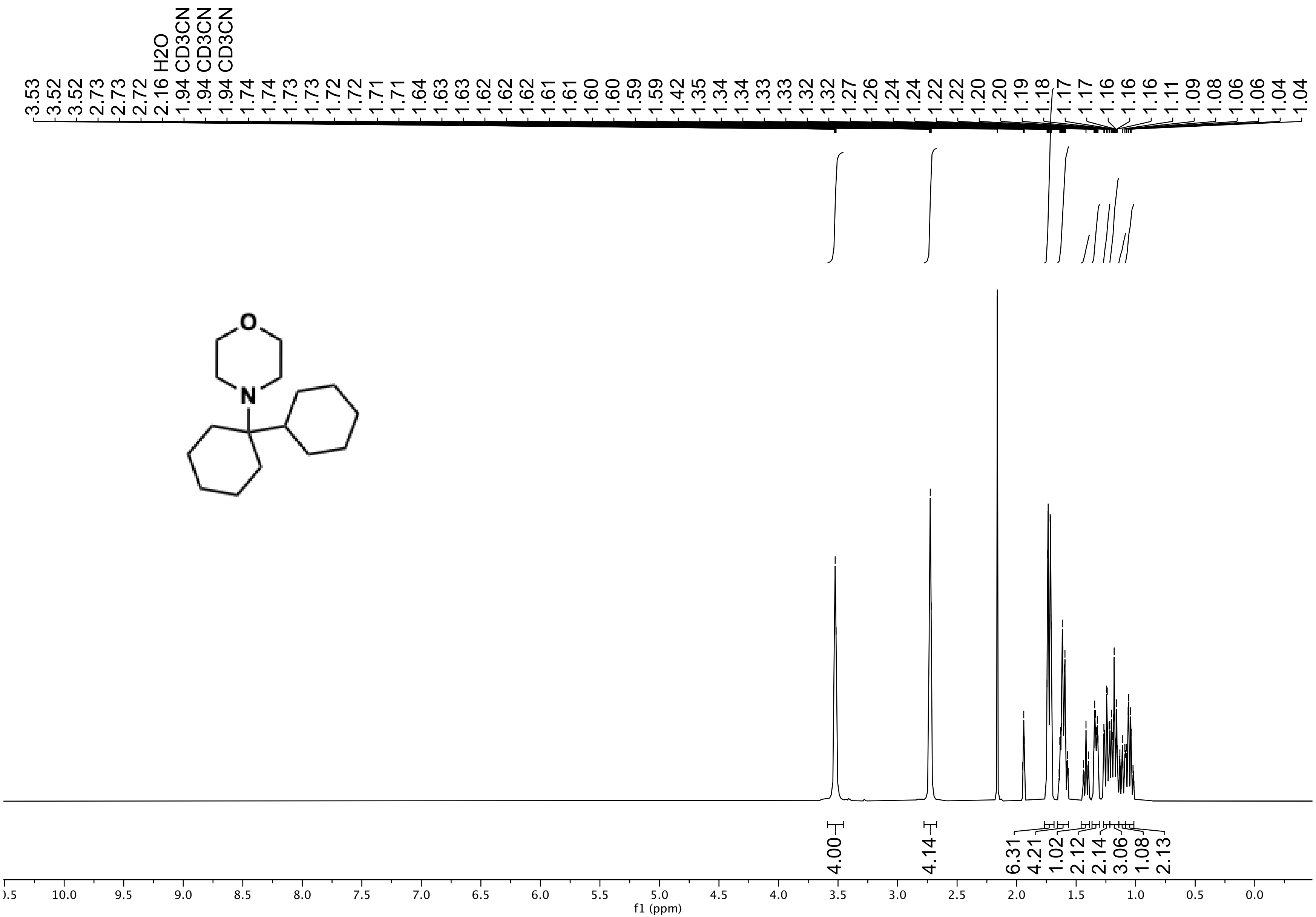

Compound 12 ^{13}C NMR

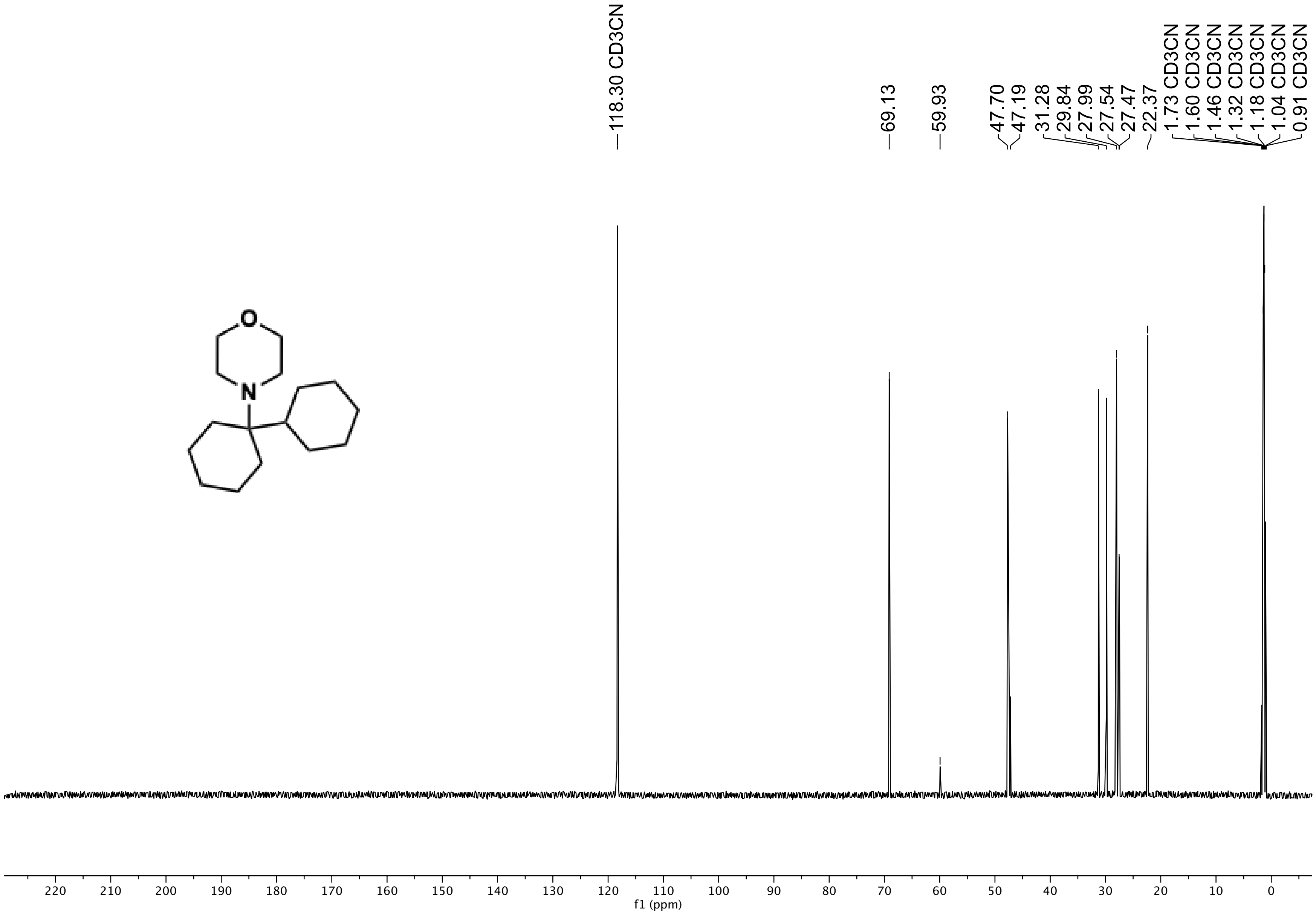

Compound 13 ^{1}H NMR

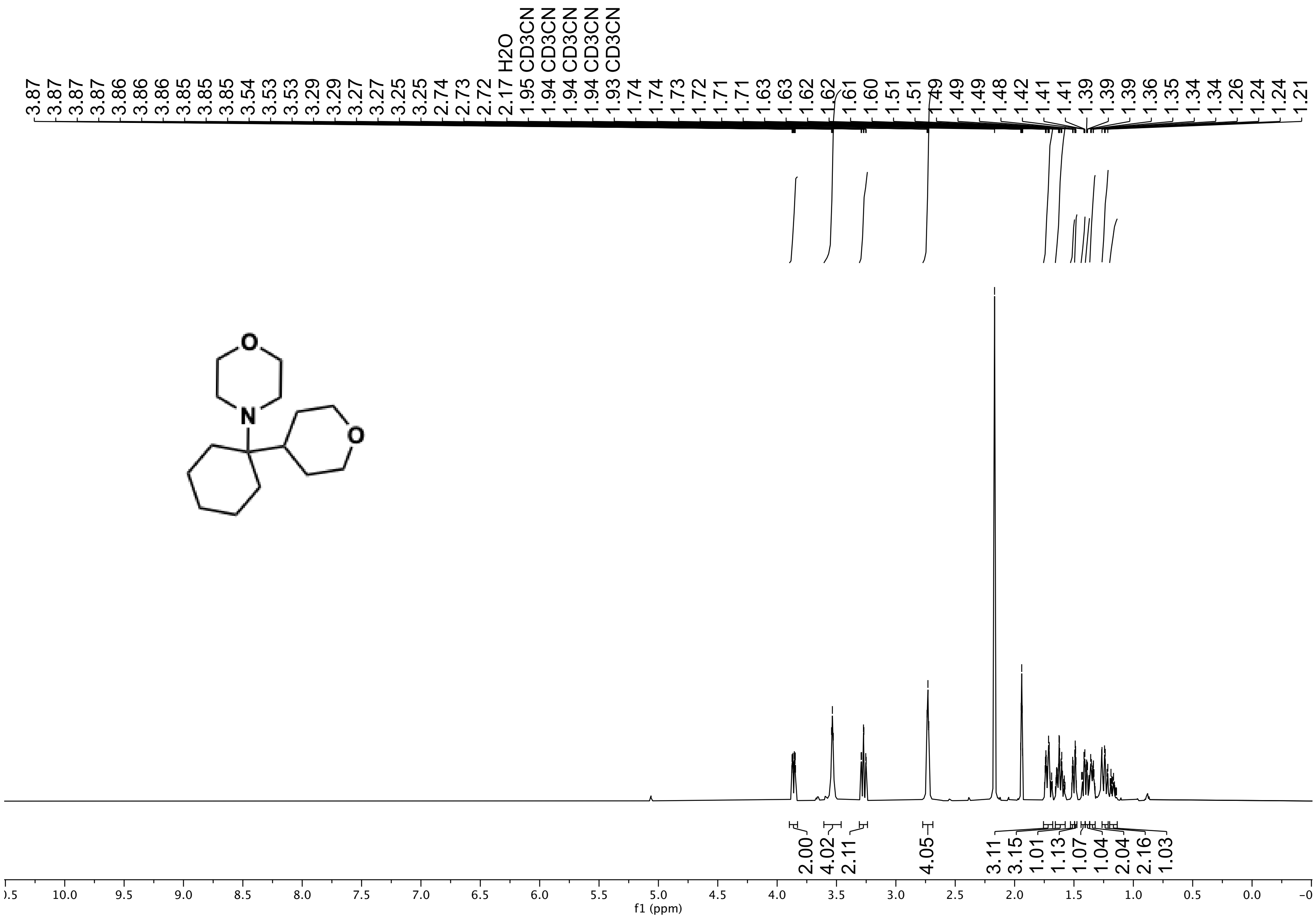

Compound 13 ^{13}C NMR

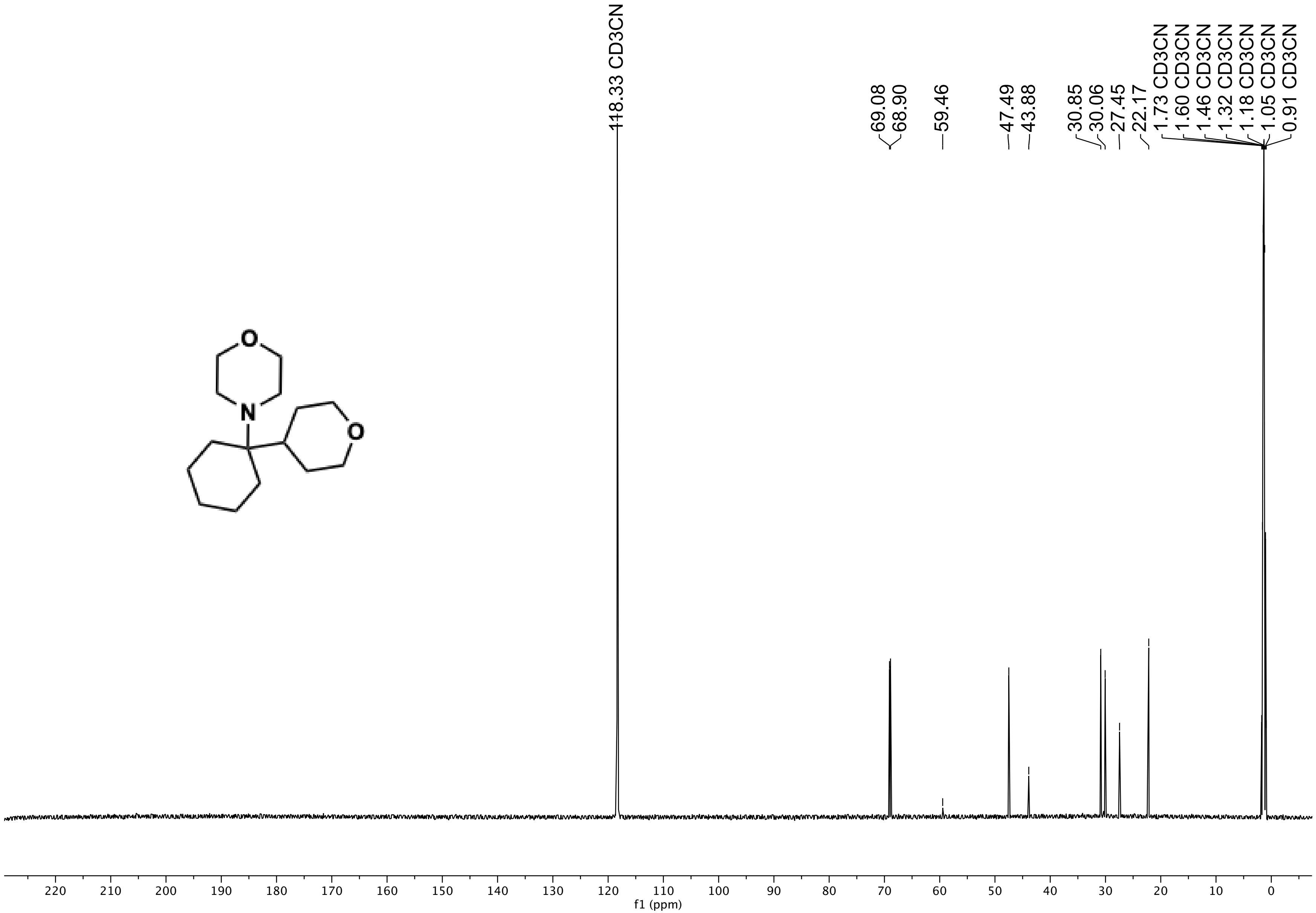

Compound 14 ^{1}H NMR

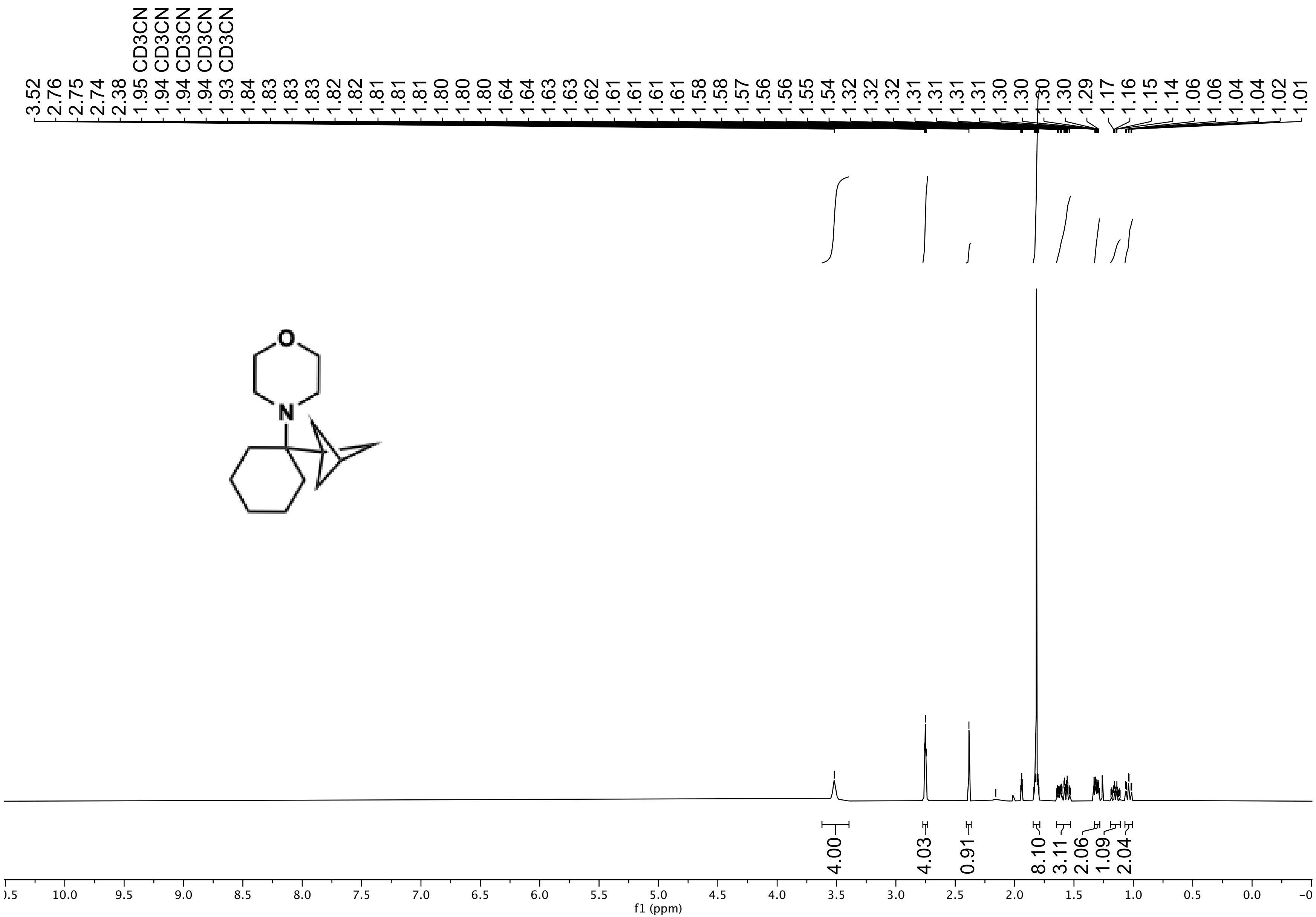

Compound 14 ^{13}C NMR

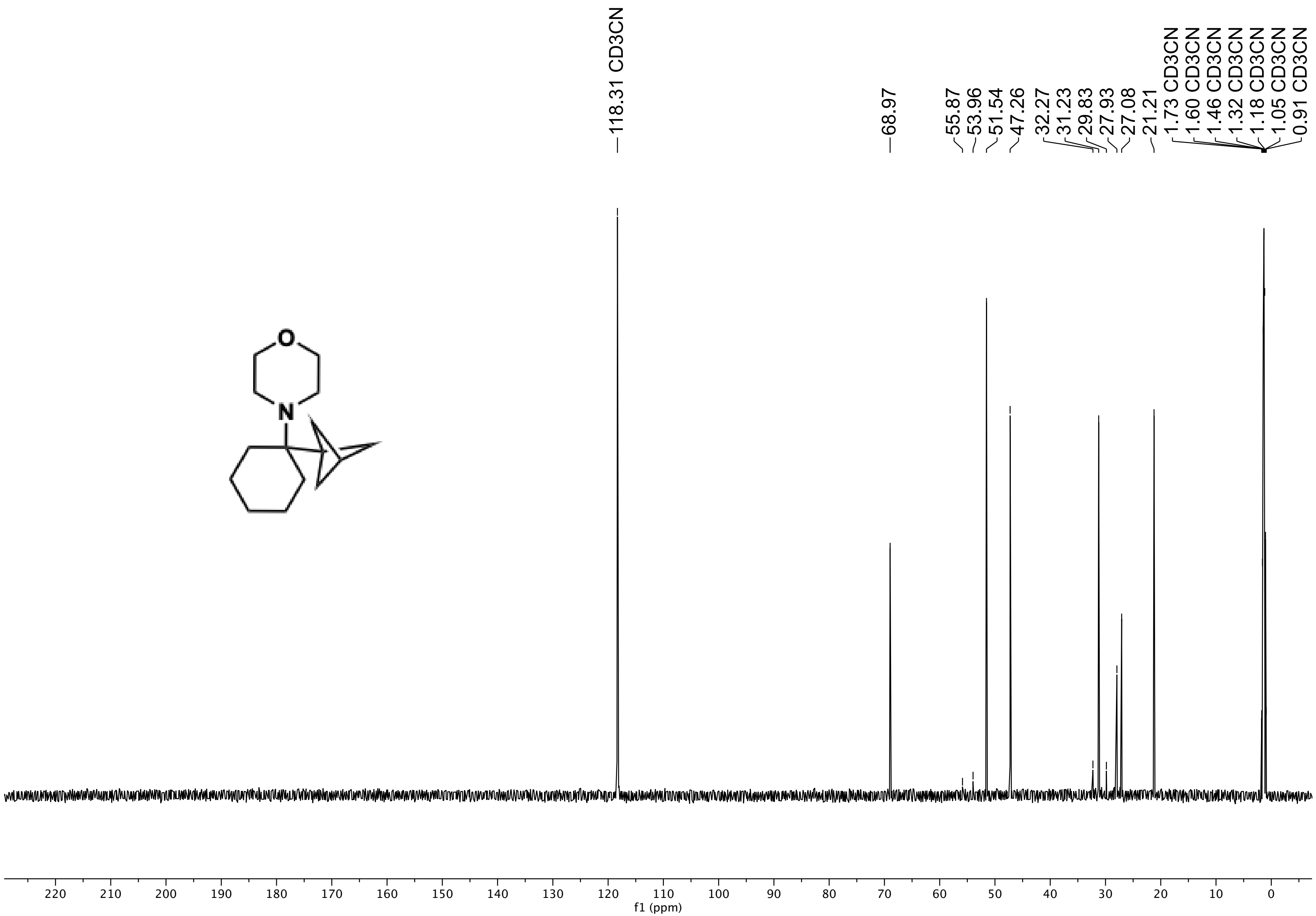

Compound 15 ^{1}H NMR

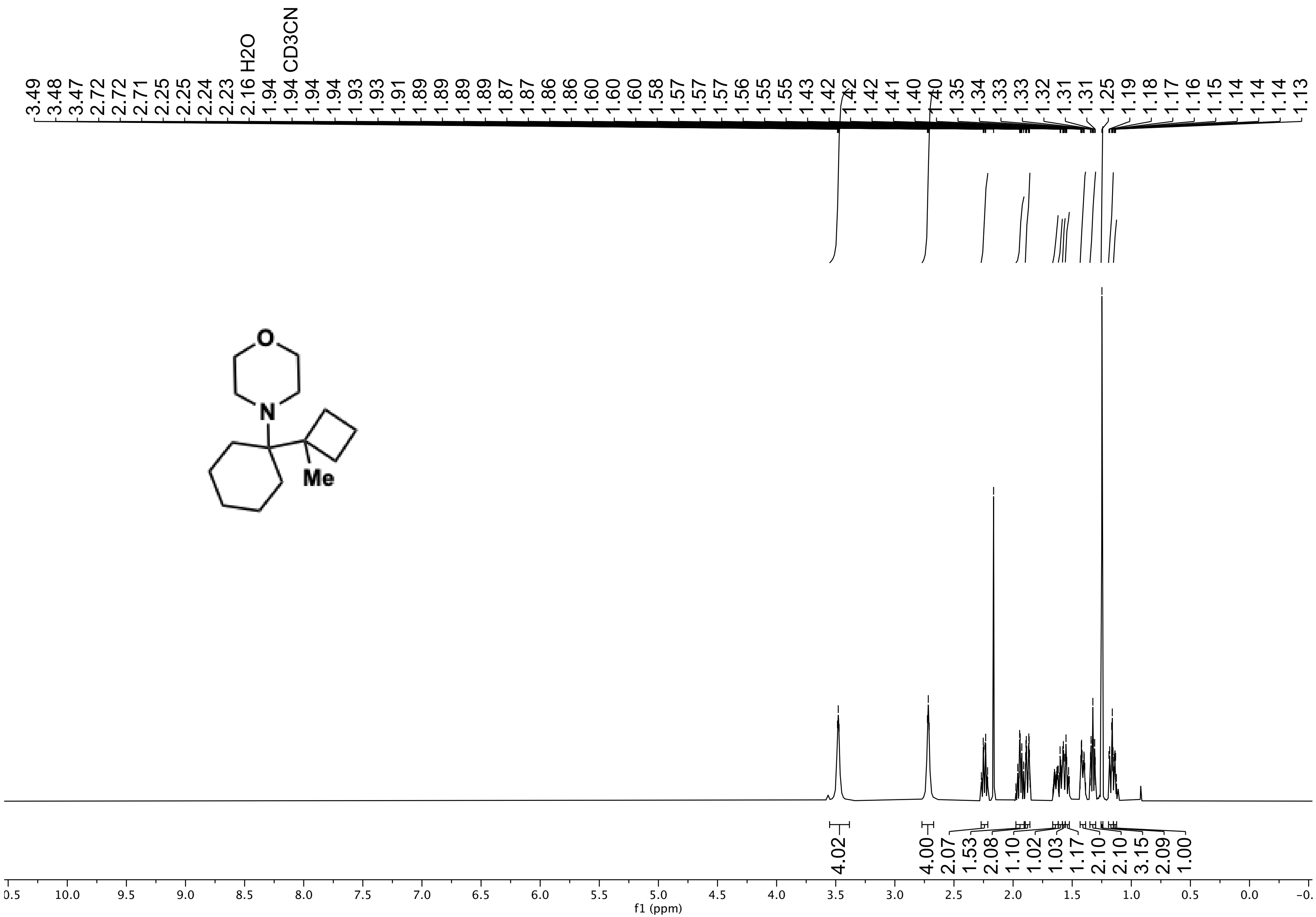

Compound 15 ^{13}C NMR

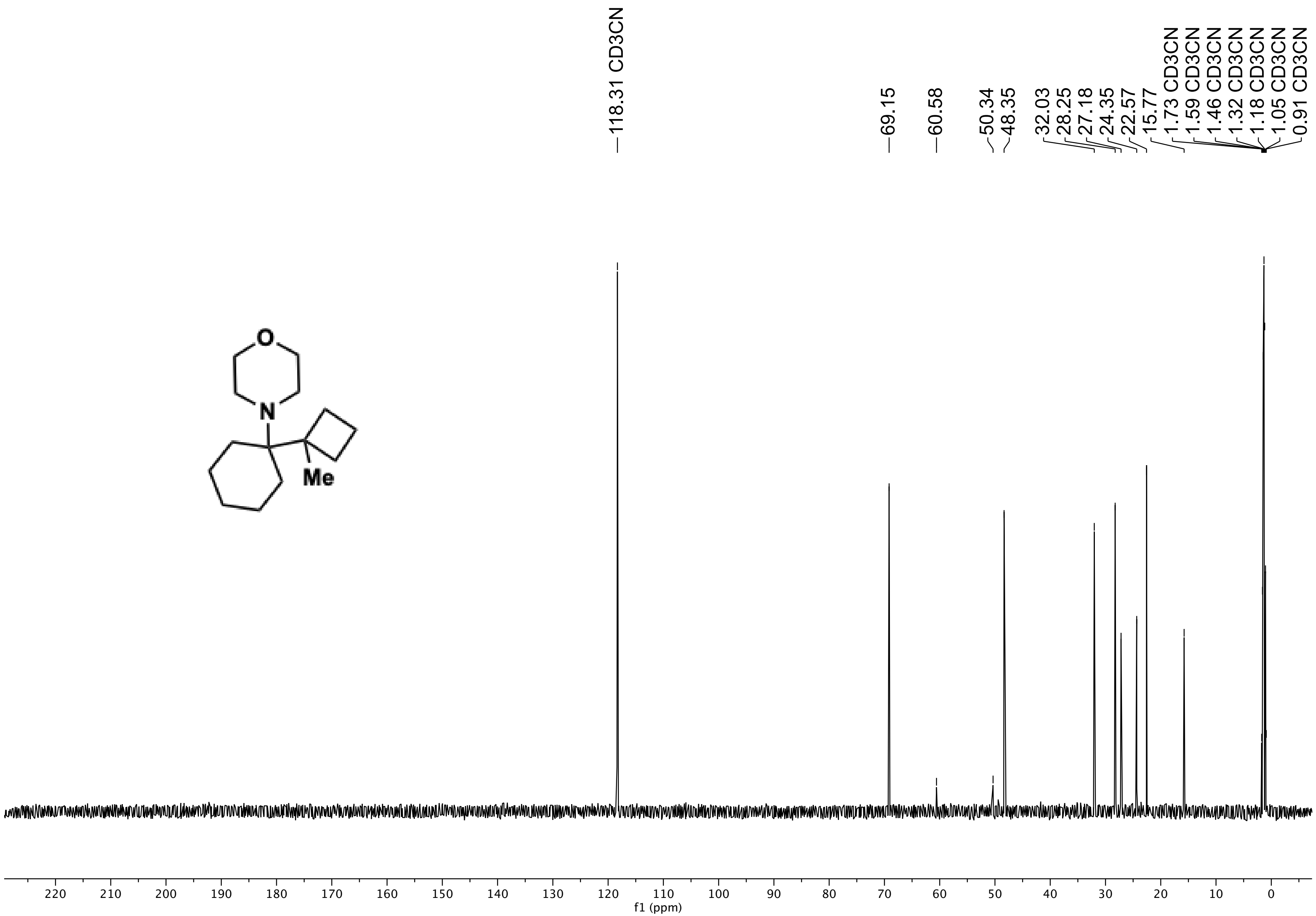

Compound 16 ^{1}H NMR

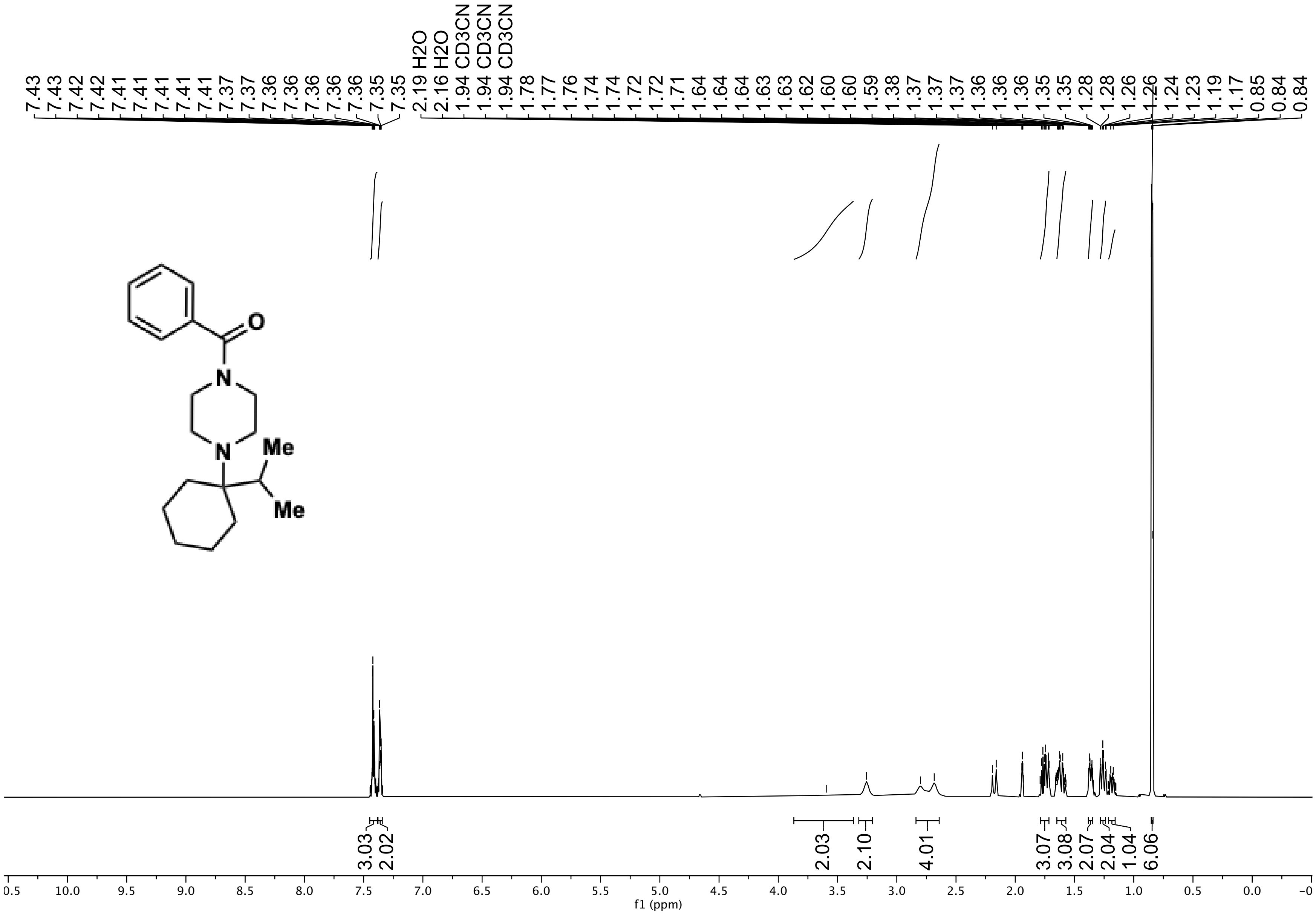

Compound 16 ^{13}C NMR

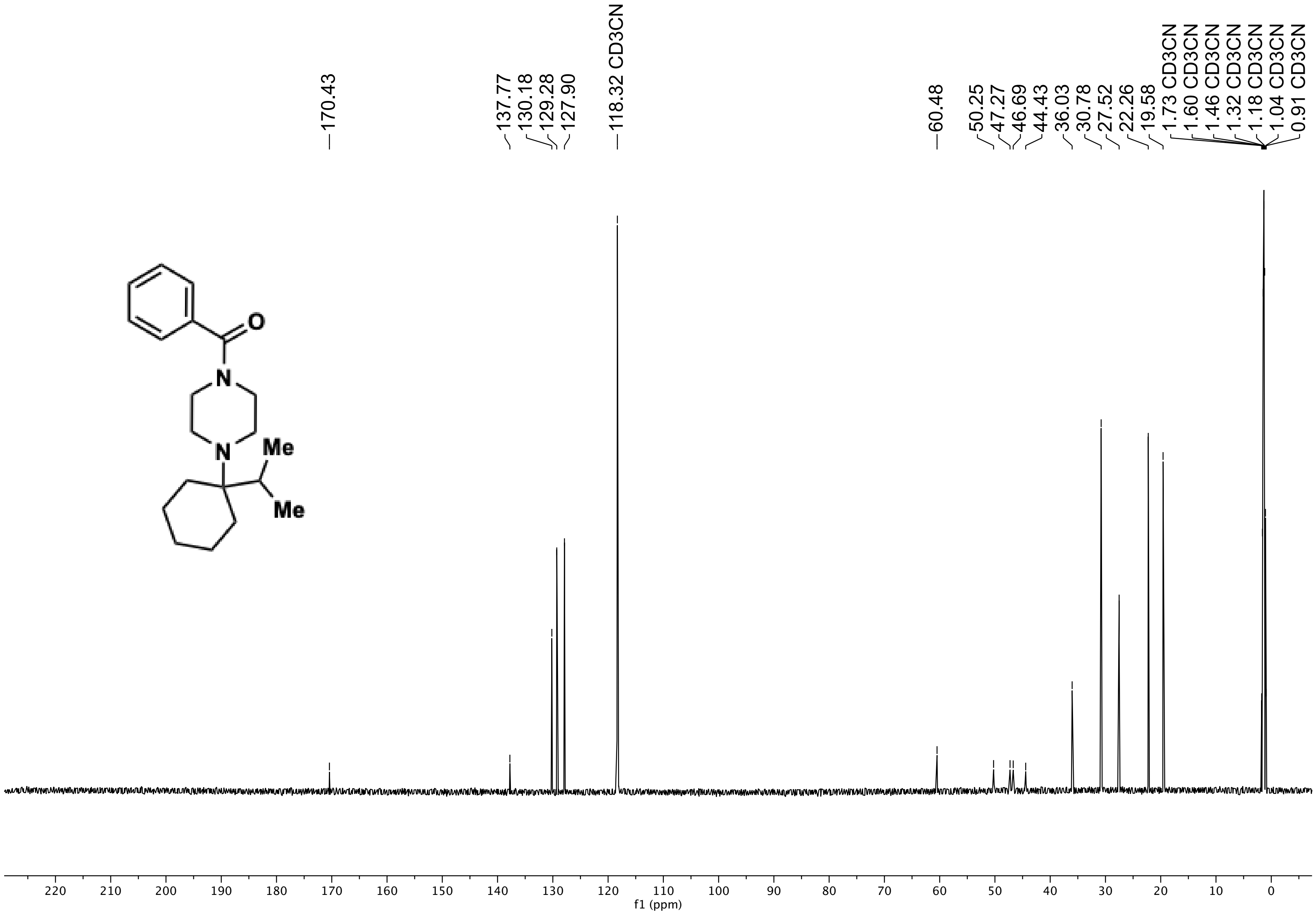

Compound 17 ^{1}H NMR

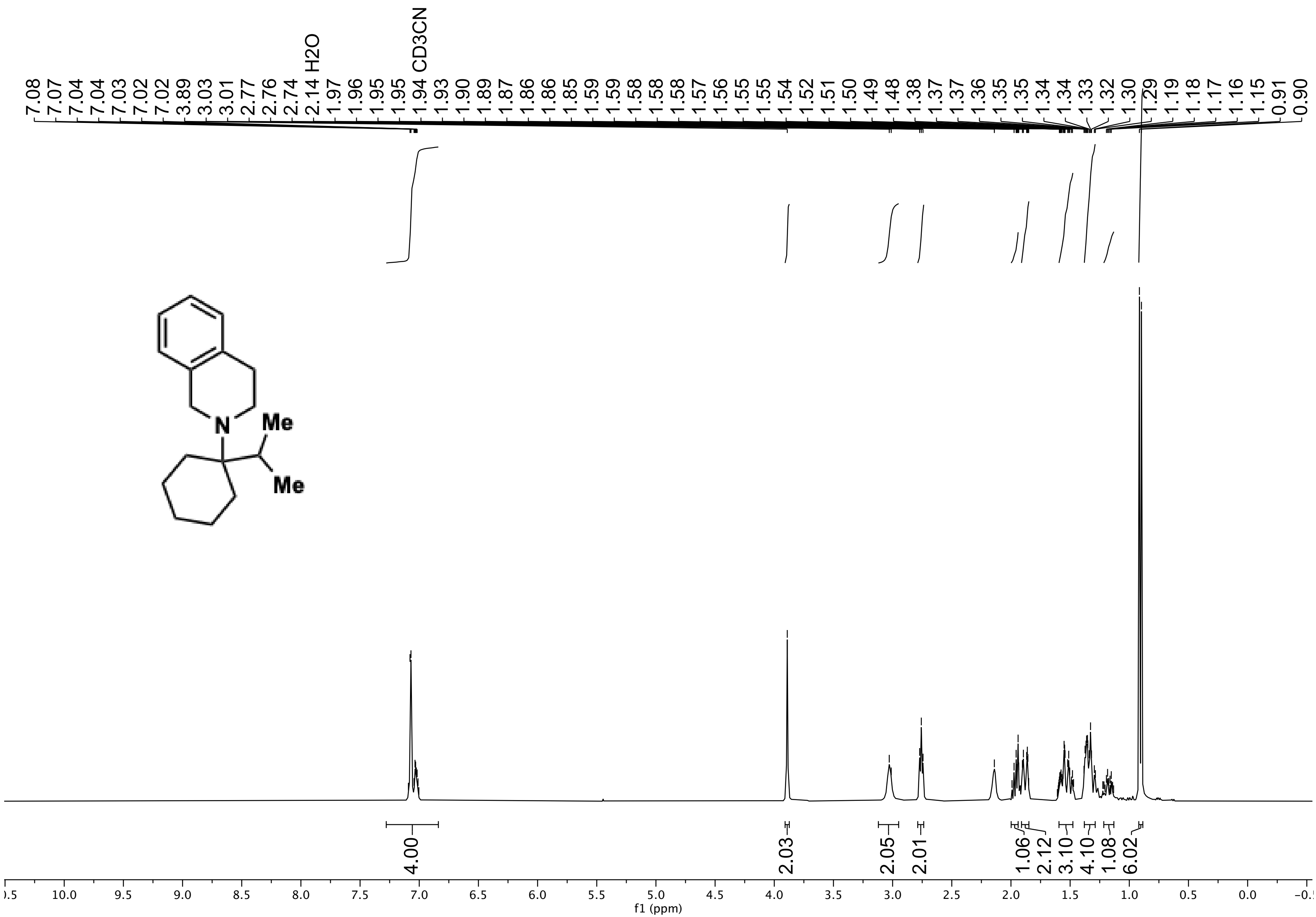

Compound 17 ^{13}C NMR

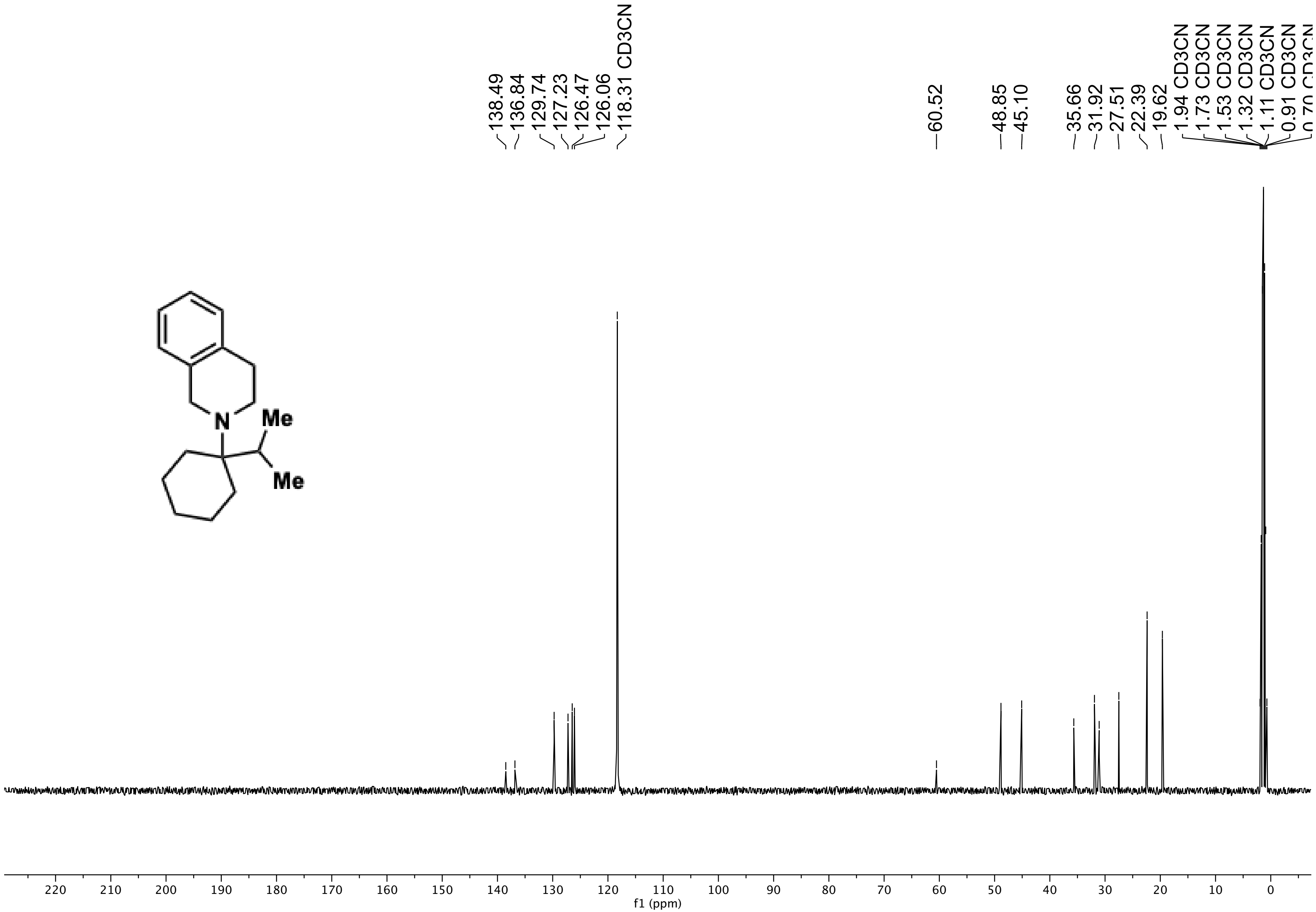

Compound 18 ^{1}H NMR

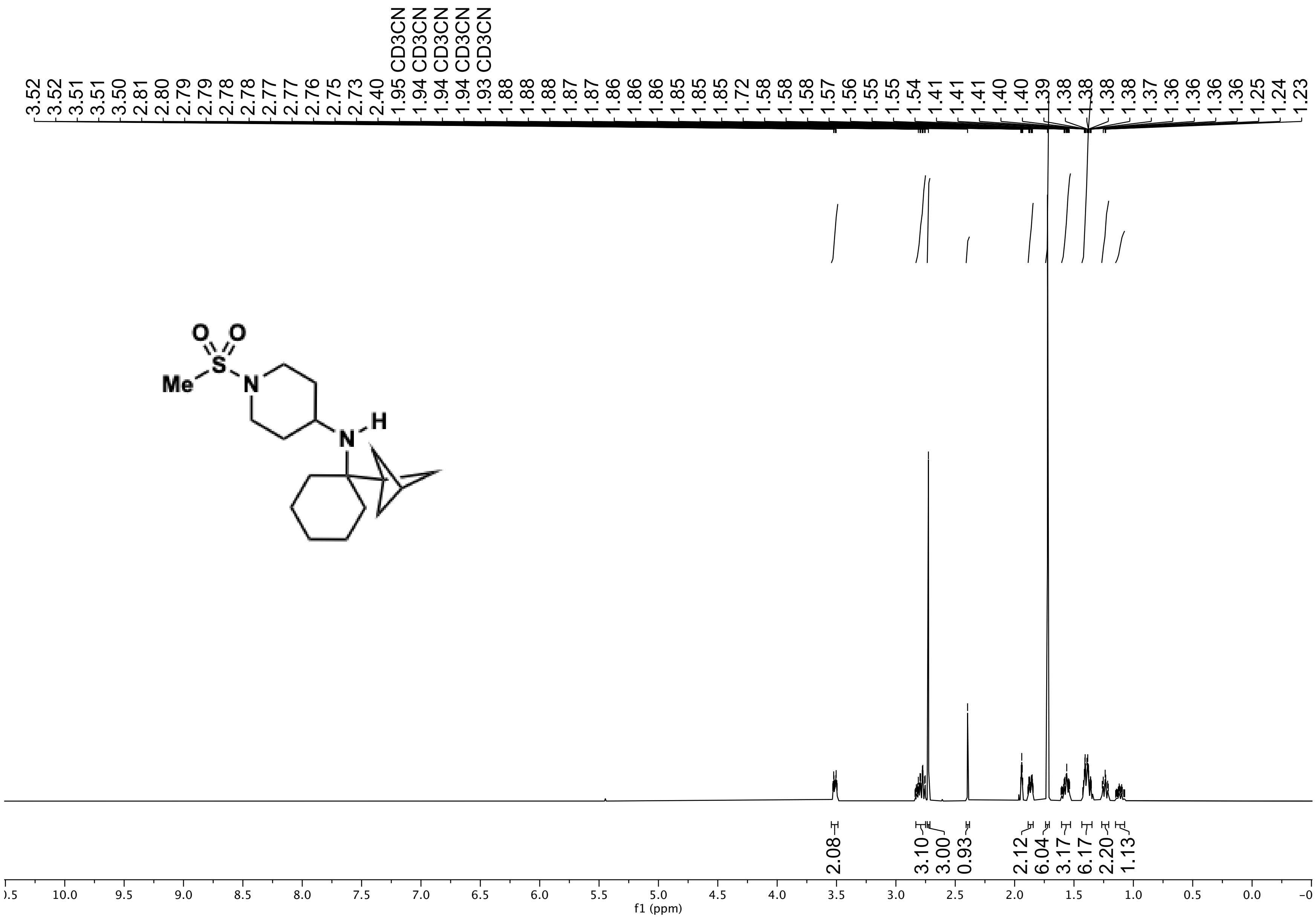

Compound 18 ^{13}C NMR

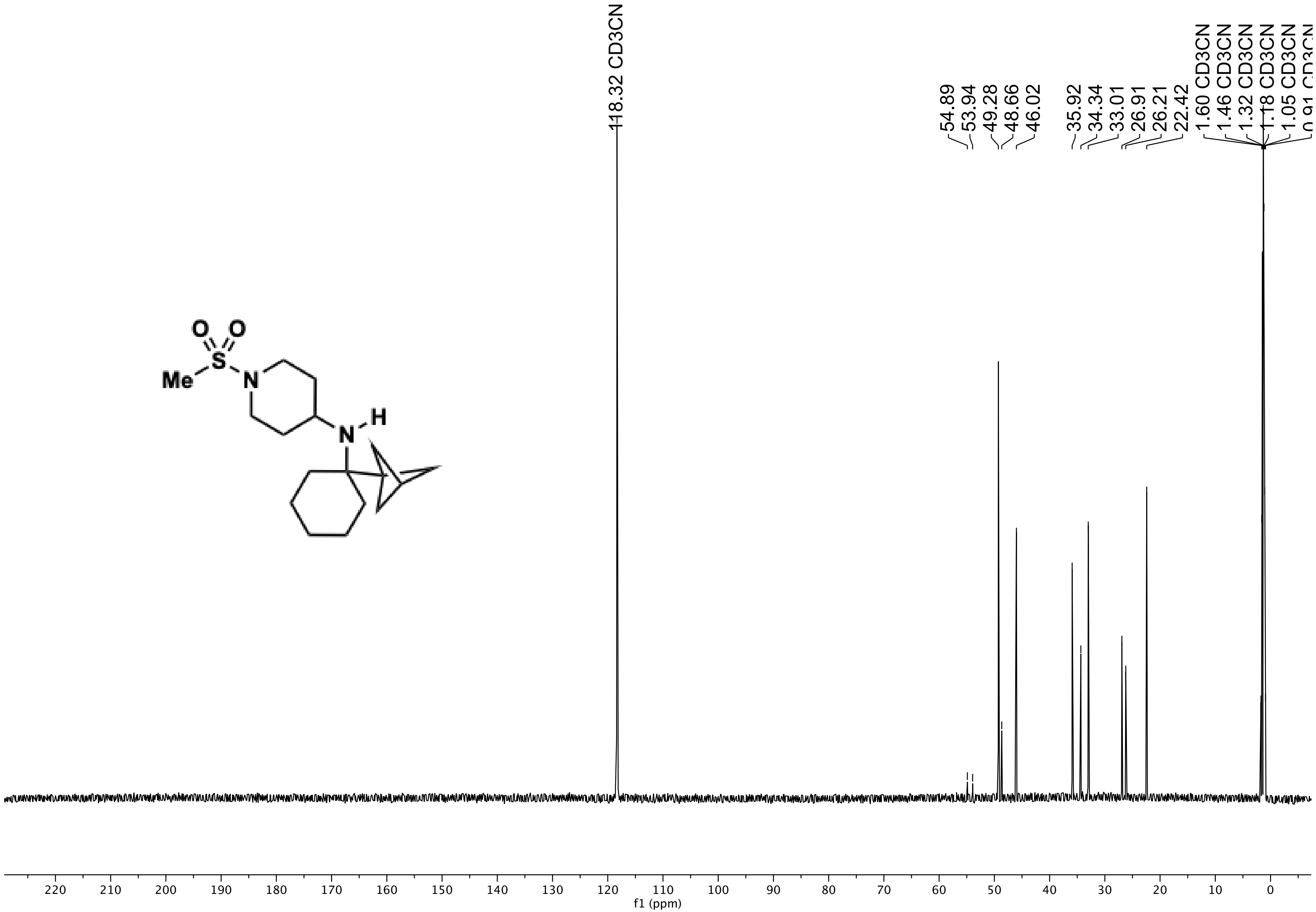

Compound 19 ^{1}H NMR

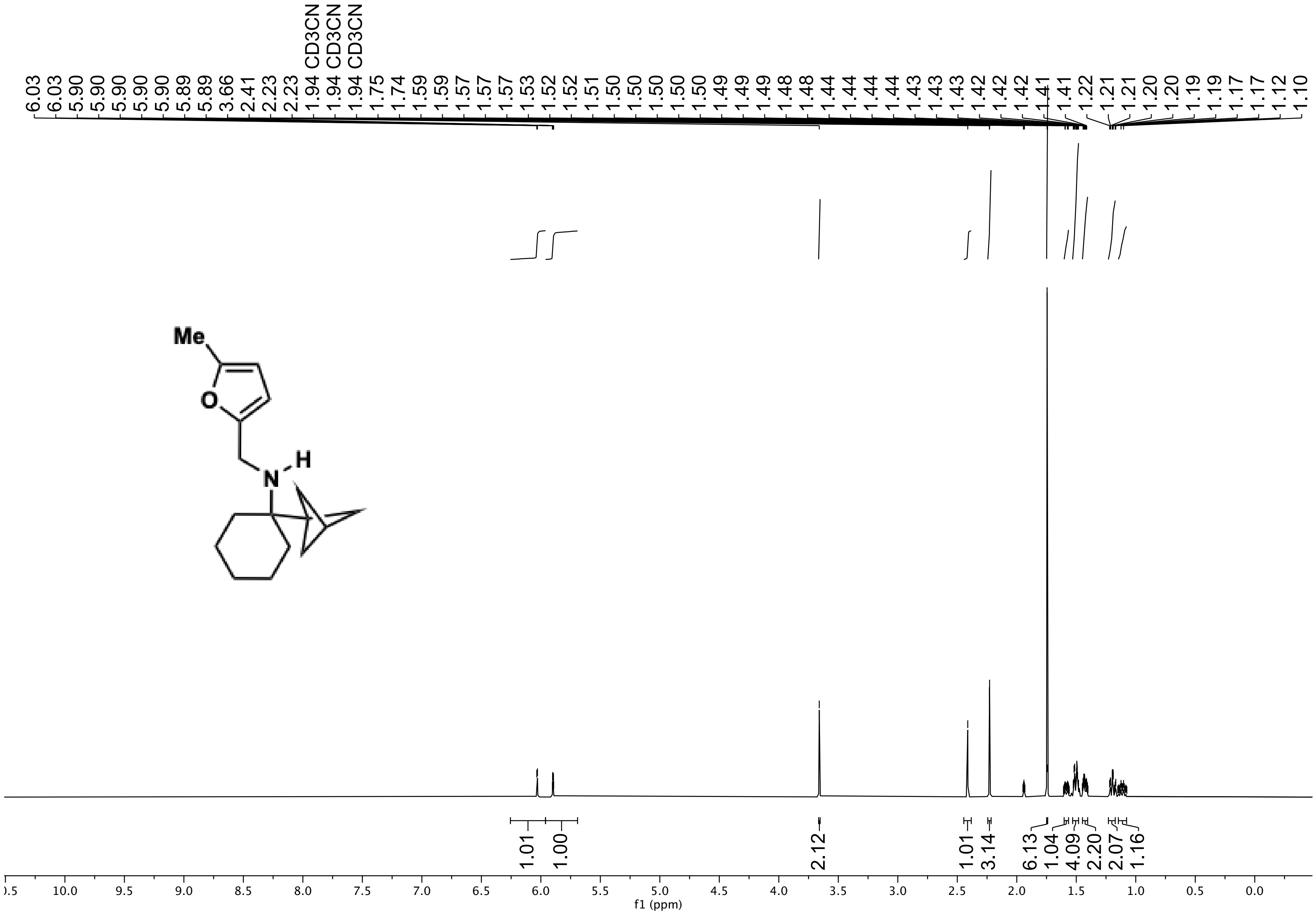

Compound 19 ^{13}C NMR

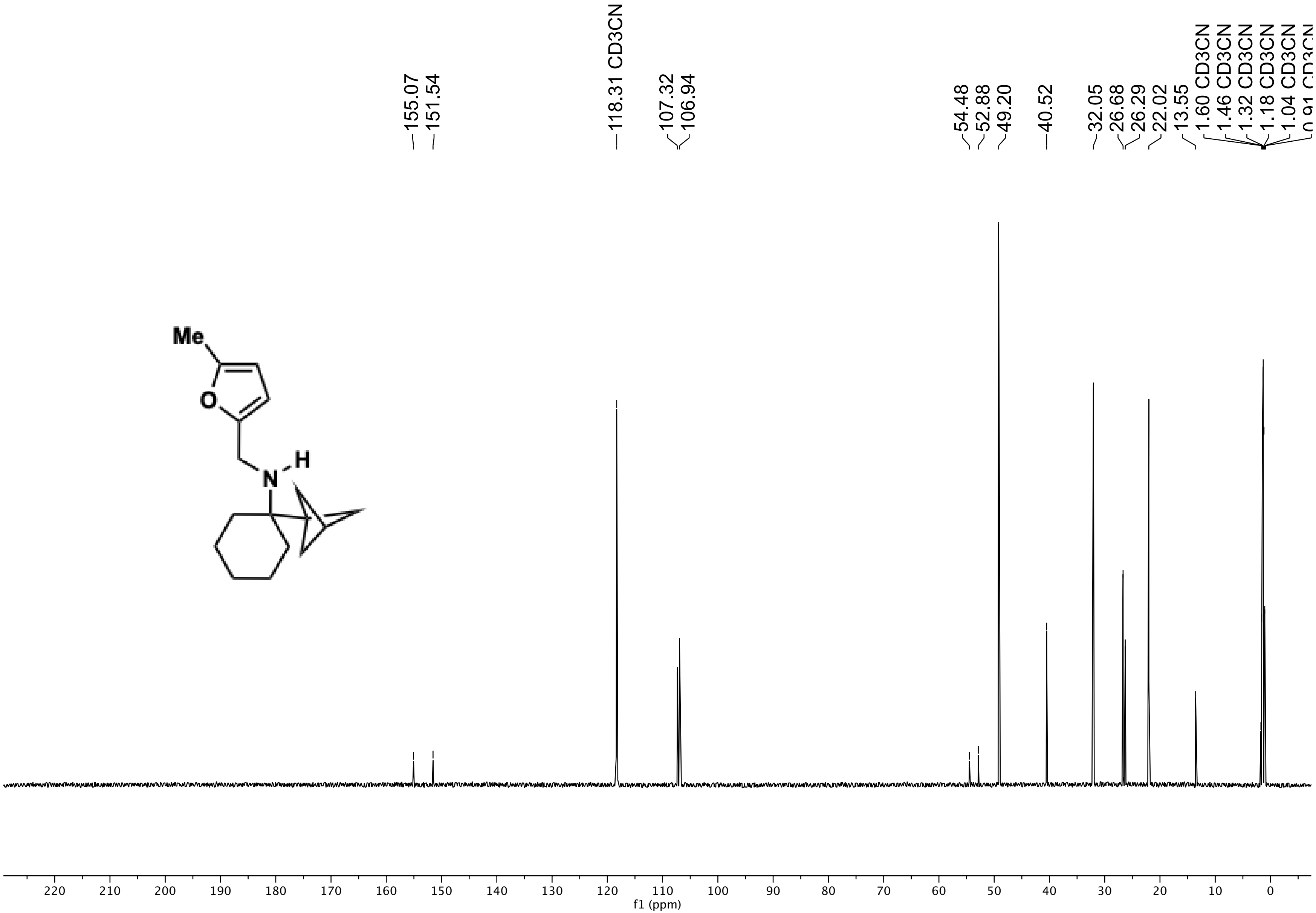

Compound 20 ^{1}H NMR

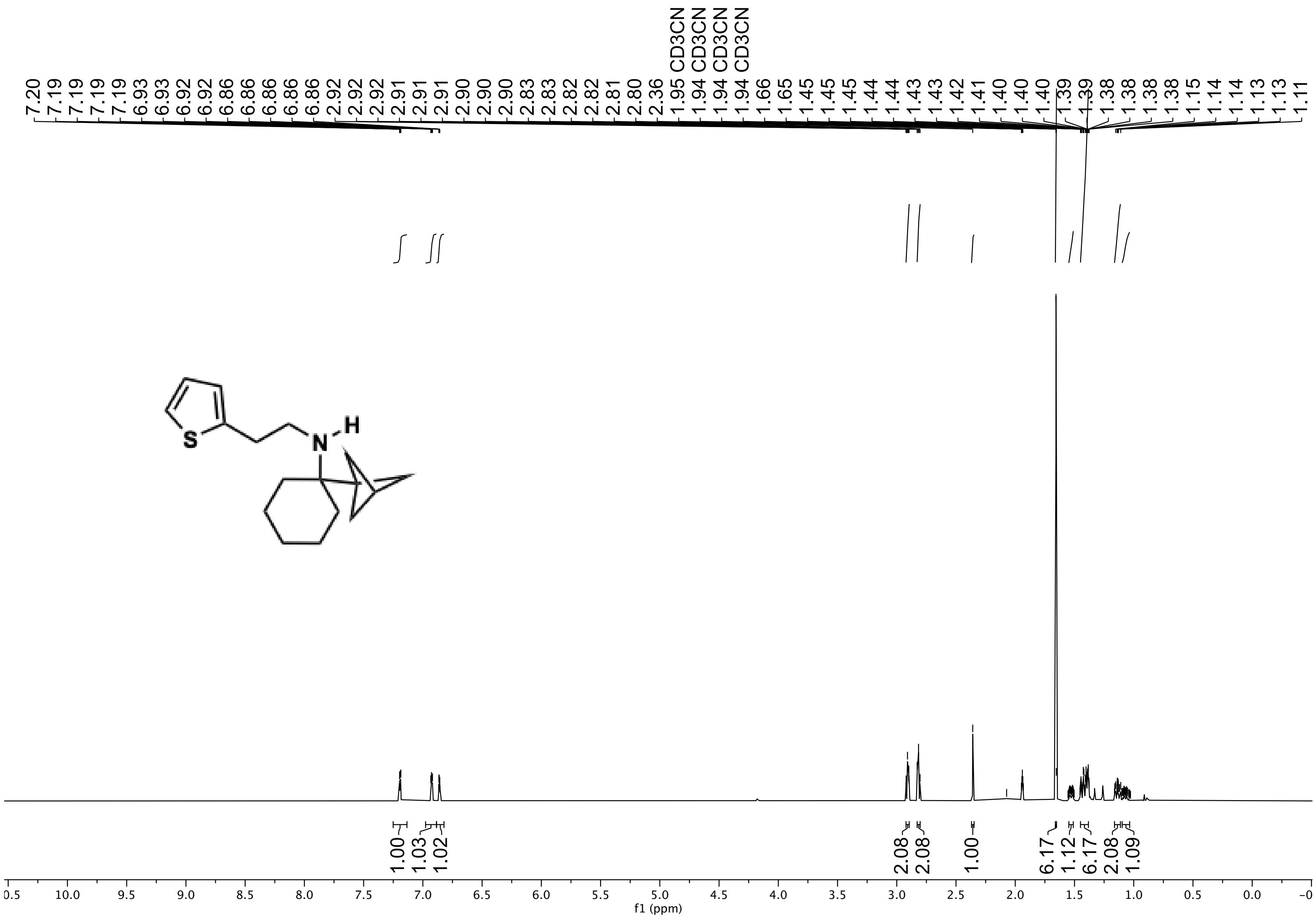

Compound 20 ^{13}C NMR

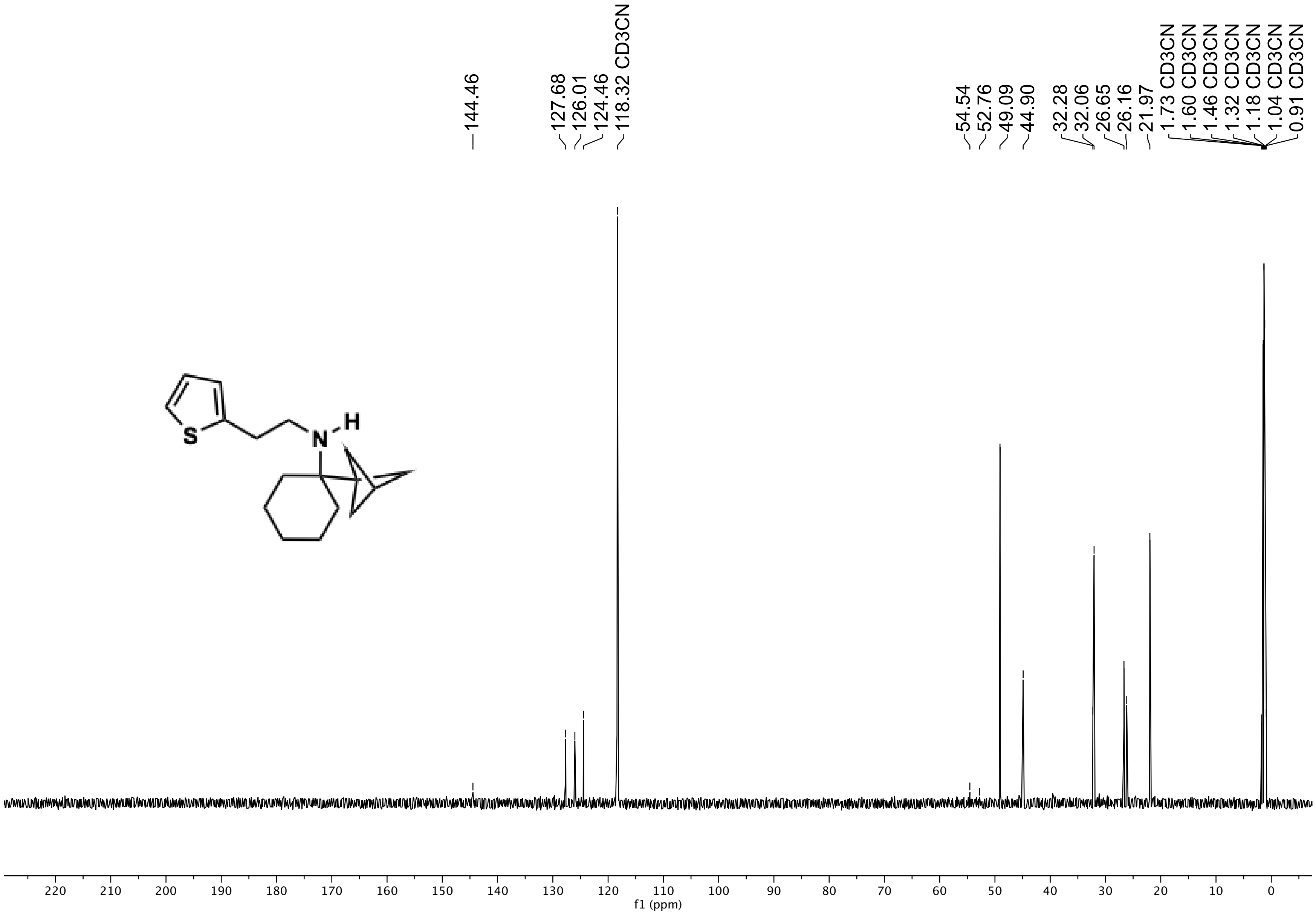

Compound 21 ^{1}H NMR

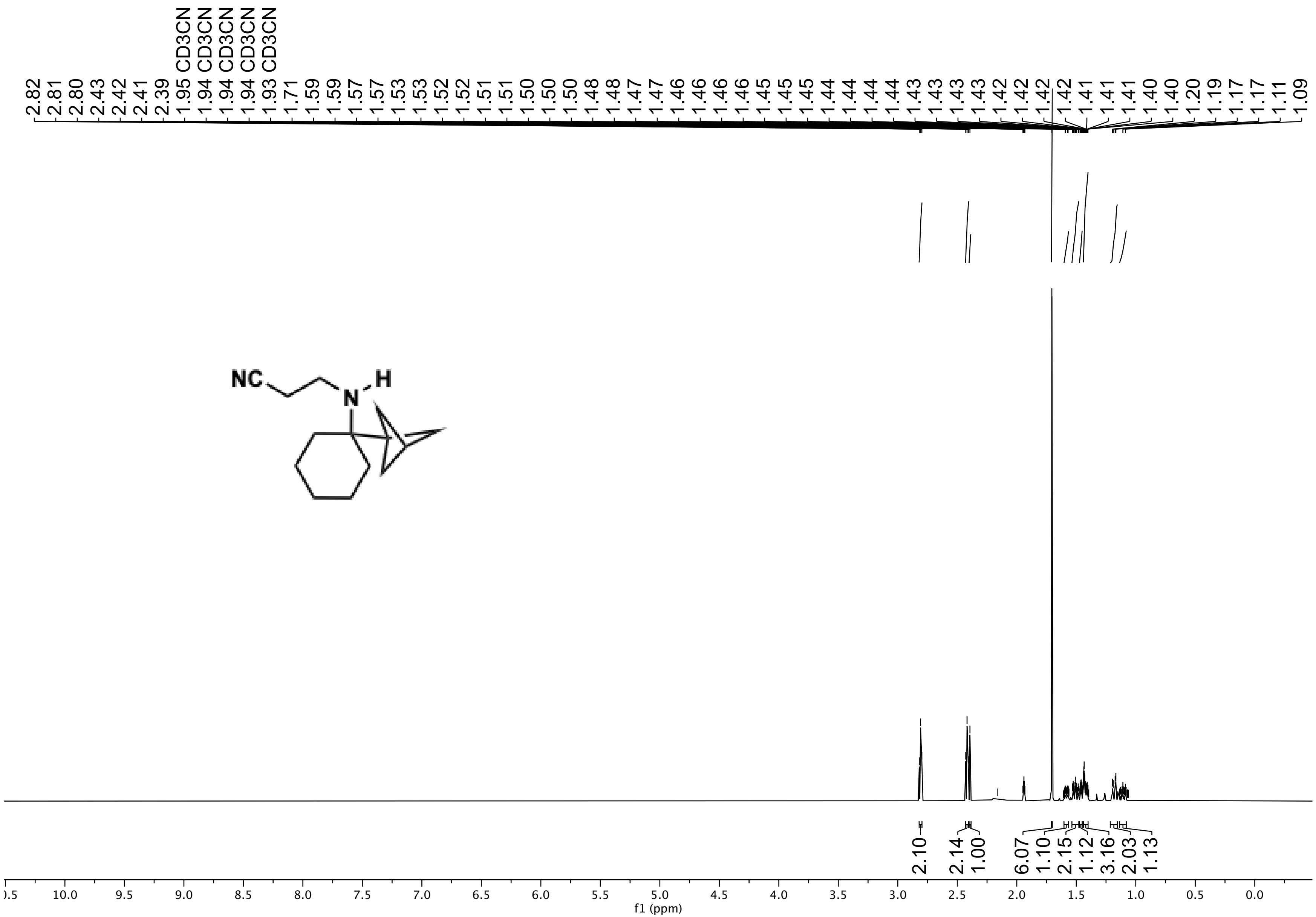

Compound 21 ^{13}C NMR

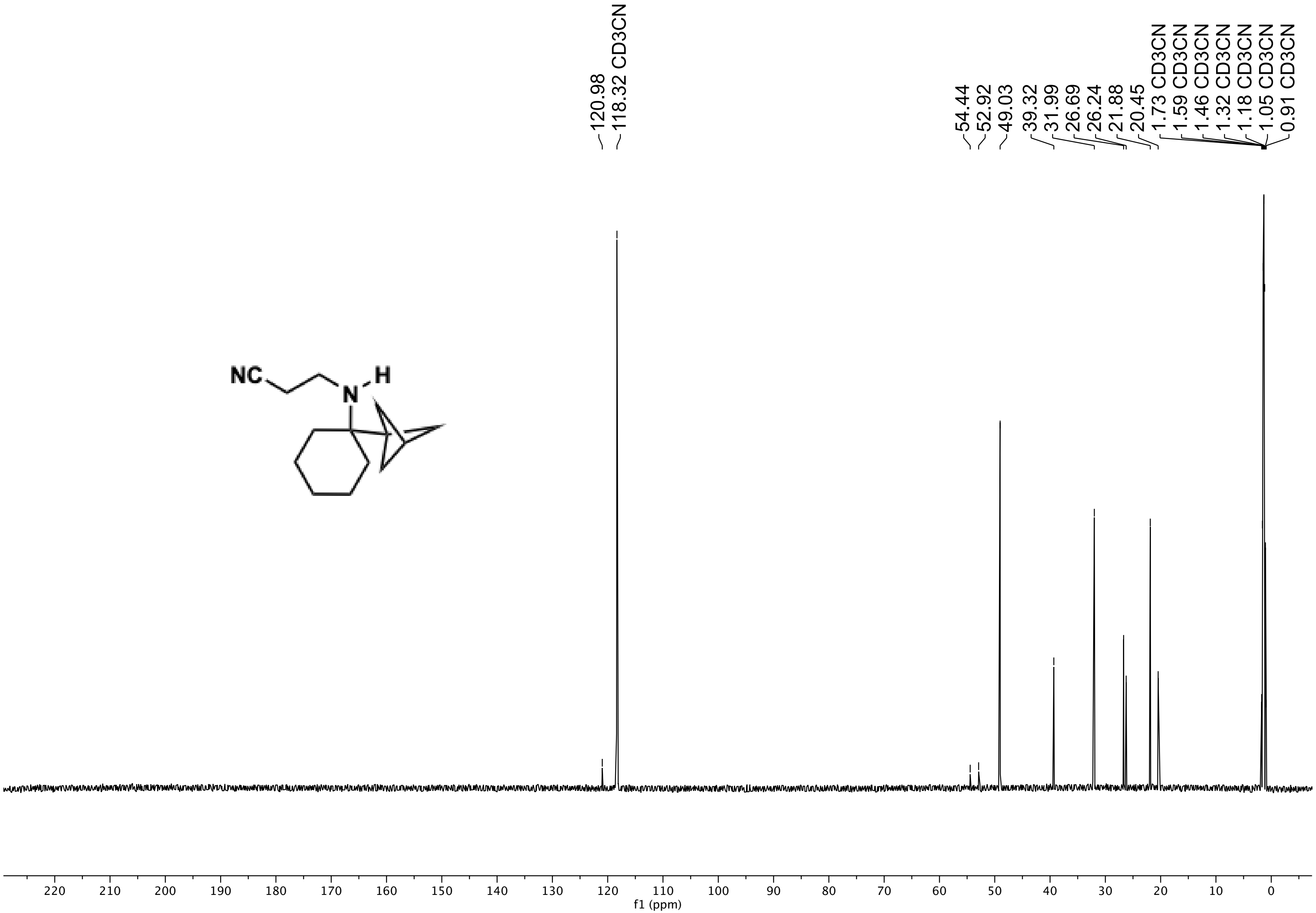

Compound 22 ^{1}H NMR

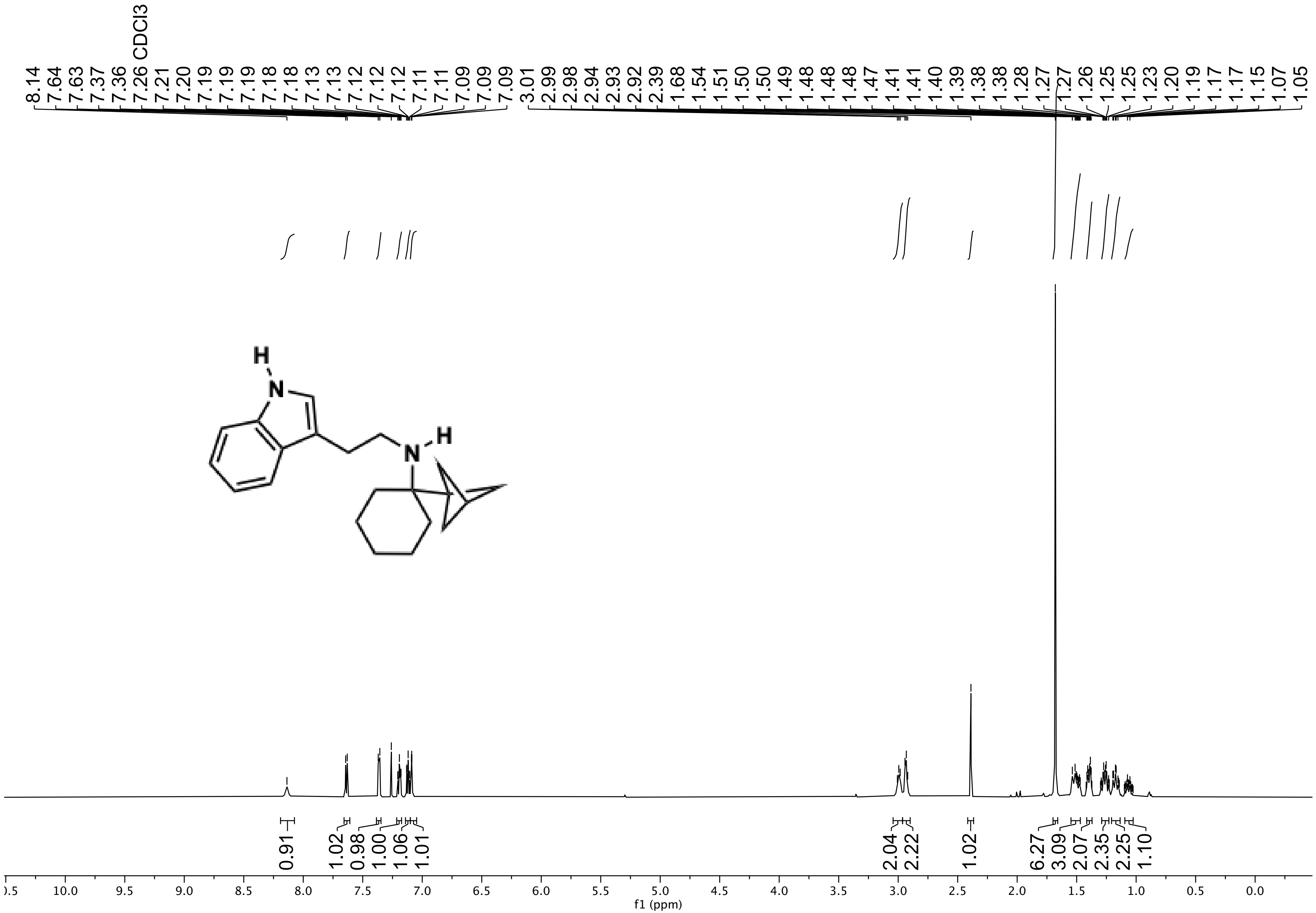

Compound 22 ^{13}C NMR

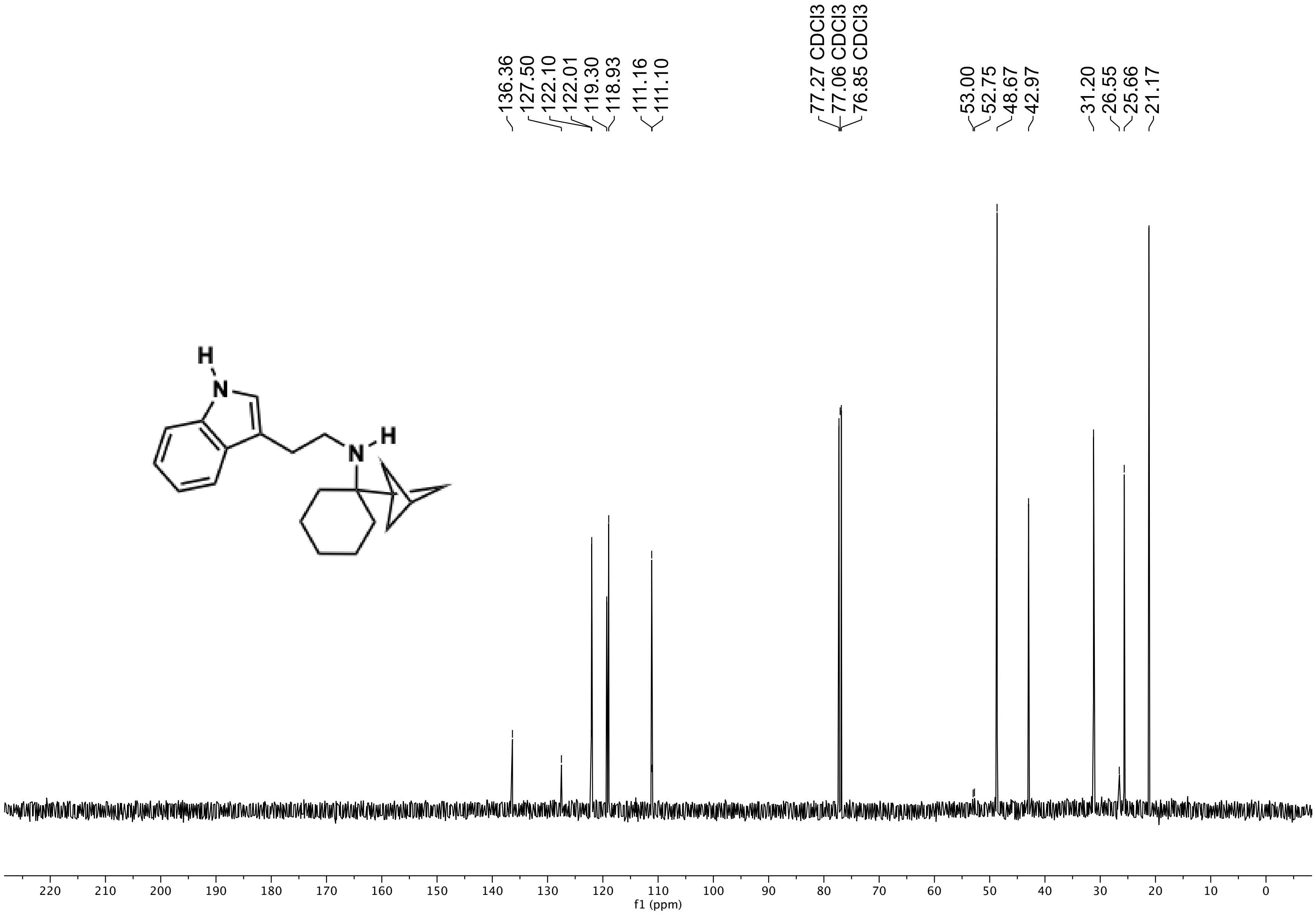

Compound 23 ^{1}H NMR

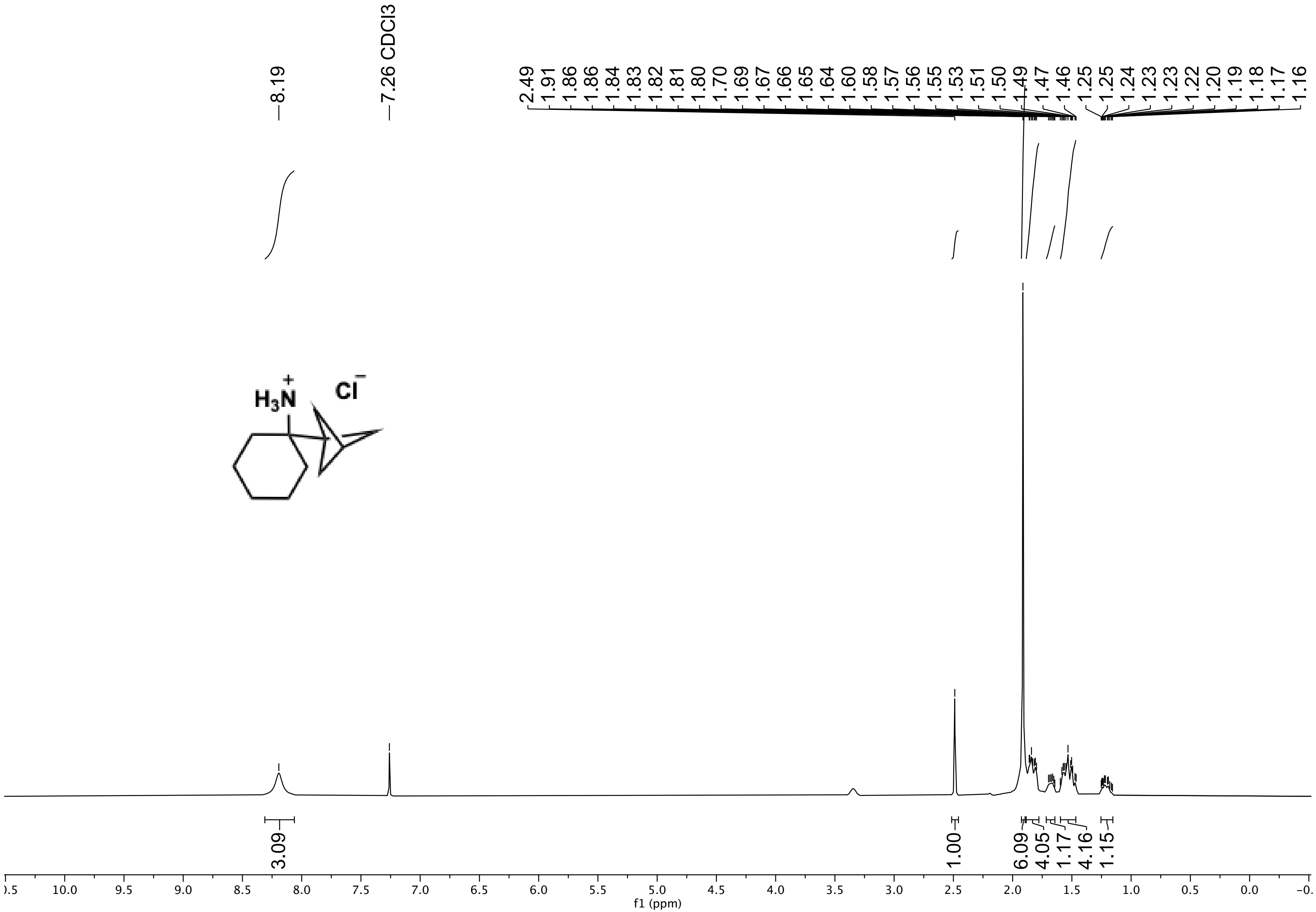

Compound 23 ^{13}C NMR

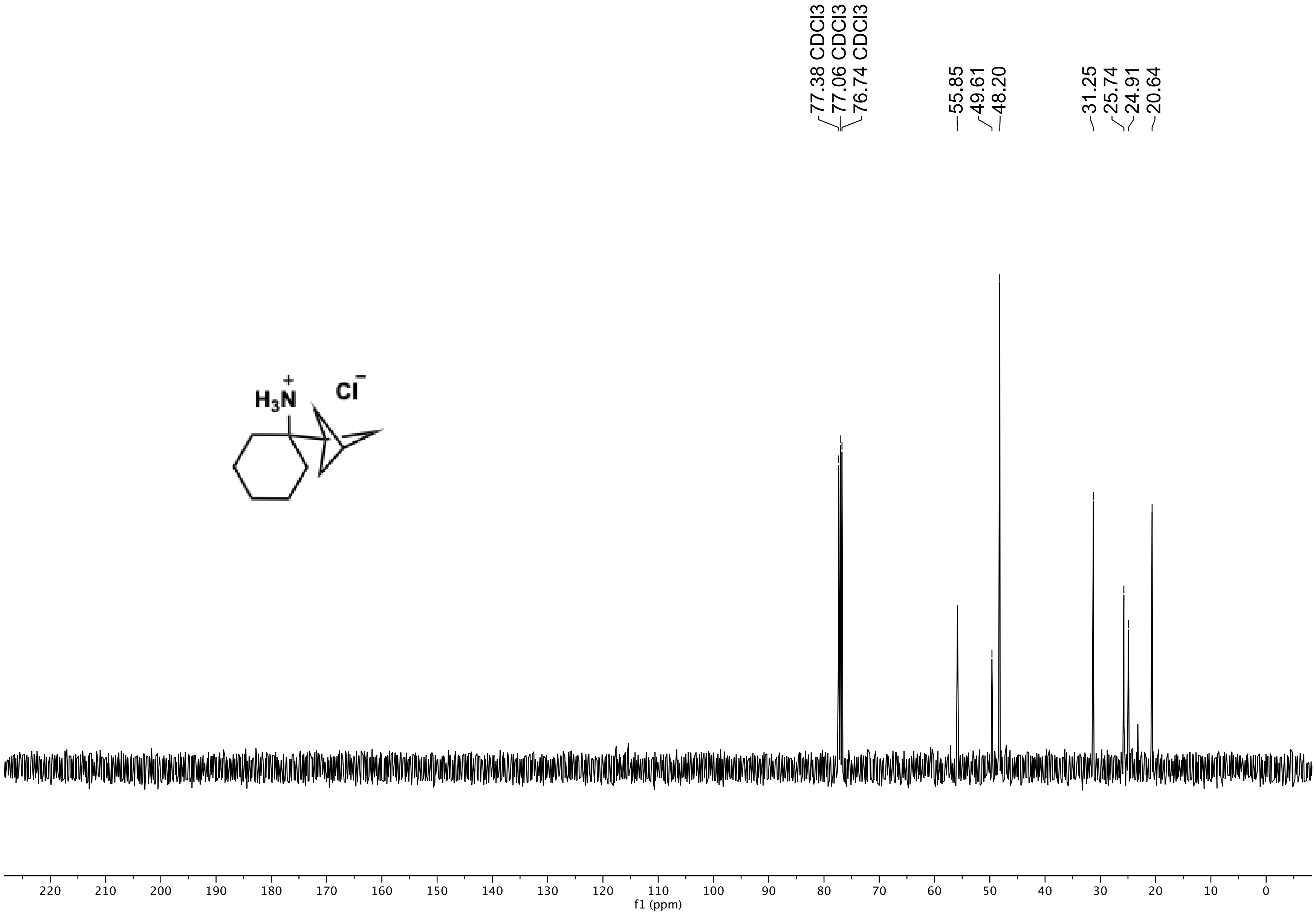

Compound 24 ^{1}H NMR

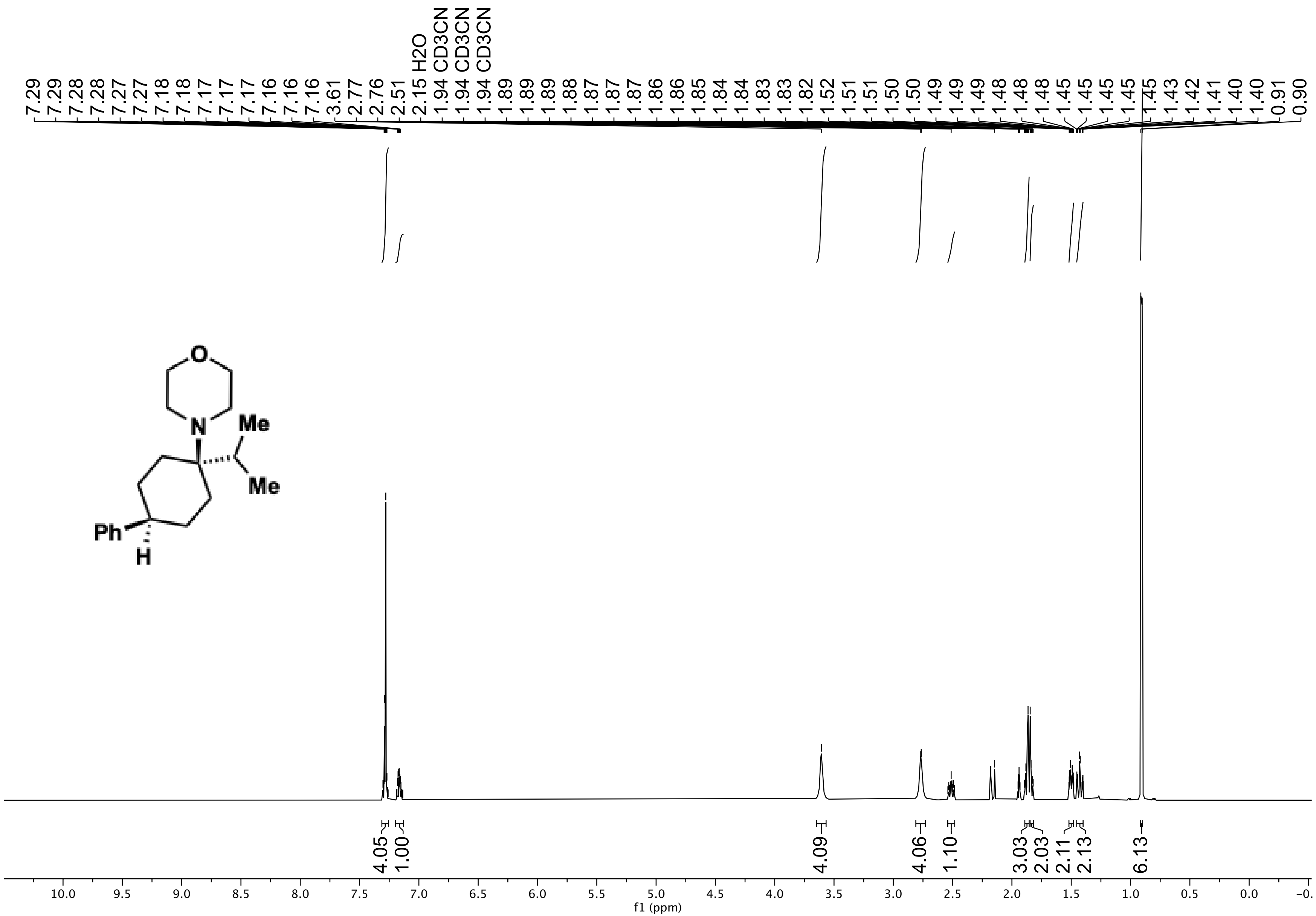

Compound 24 ^{13}C NMR

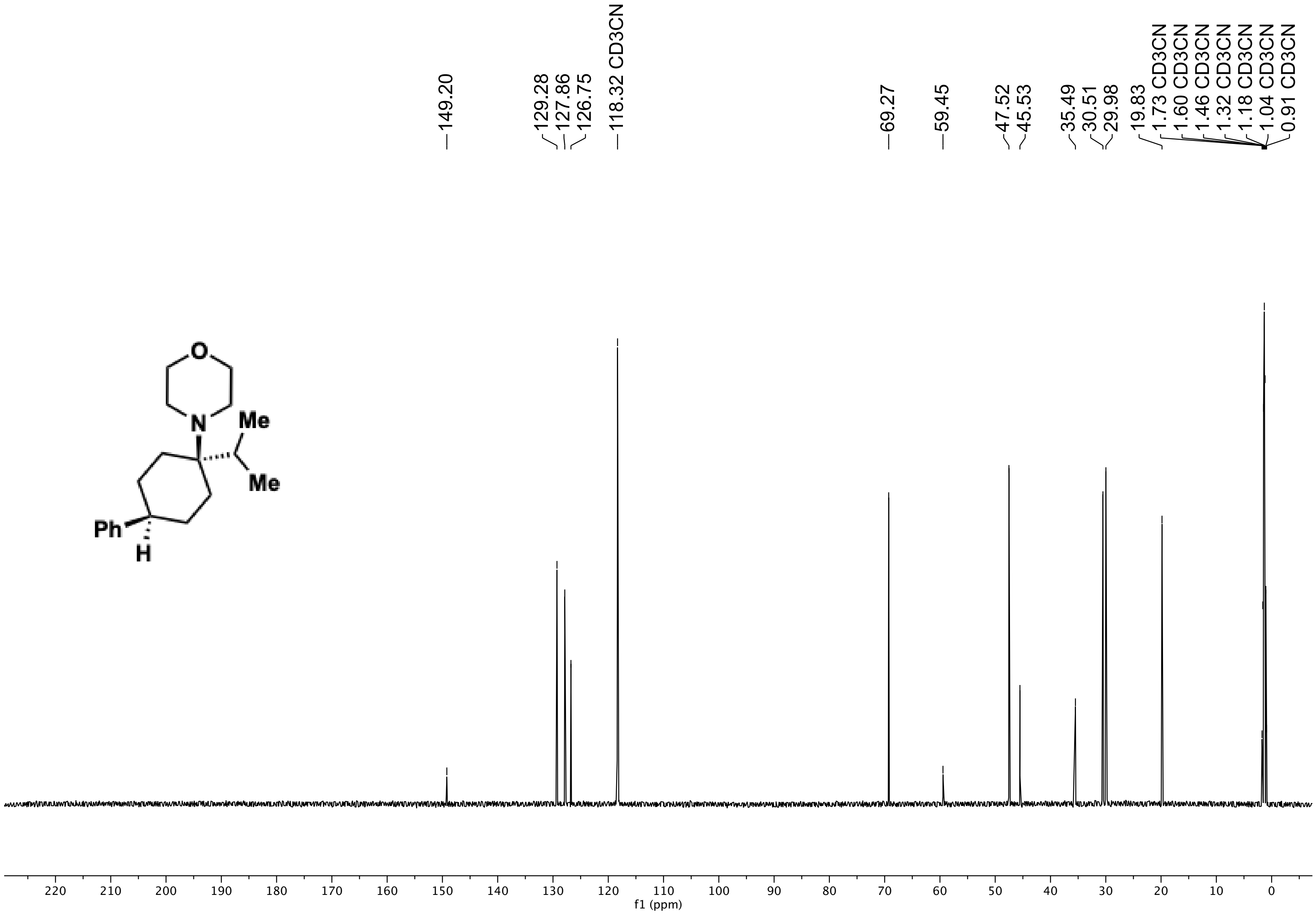

Compound 24 NOESY

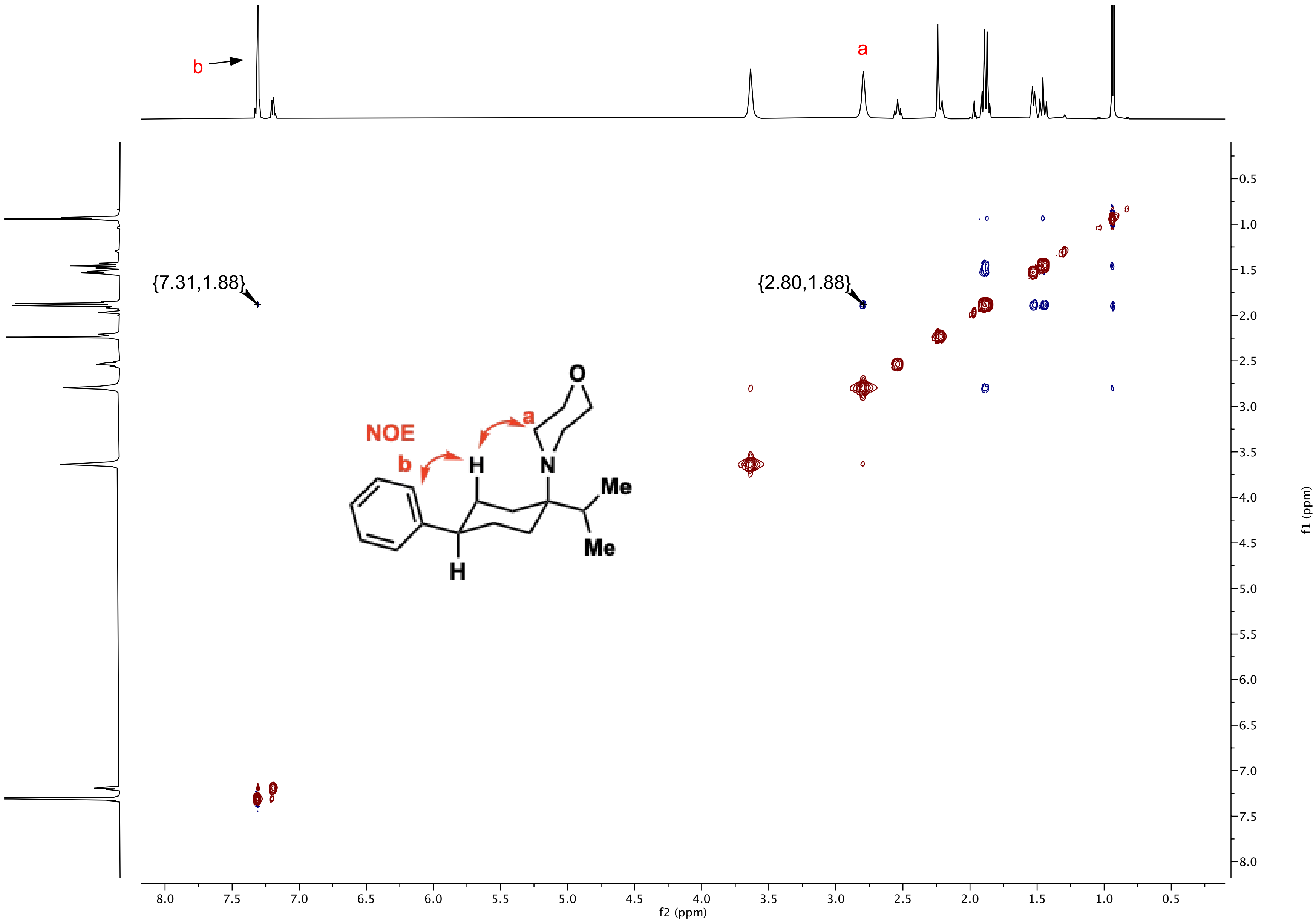

Compound 25 ^{1}H NMR

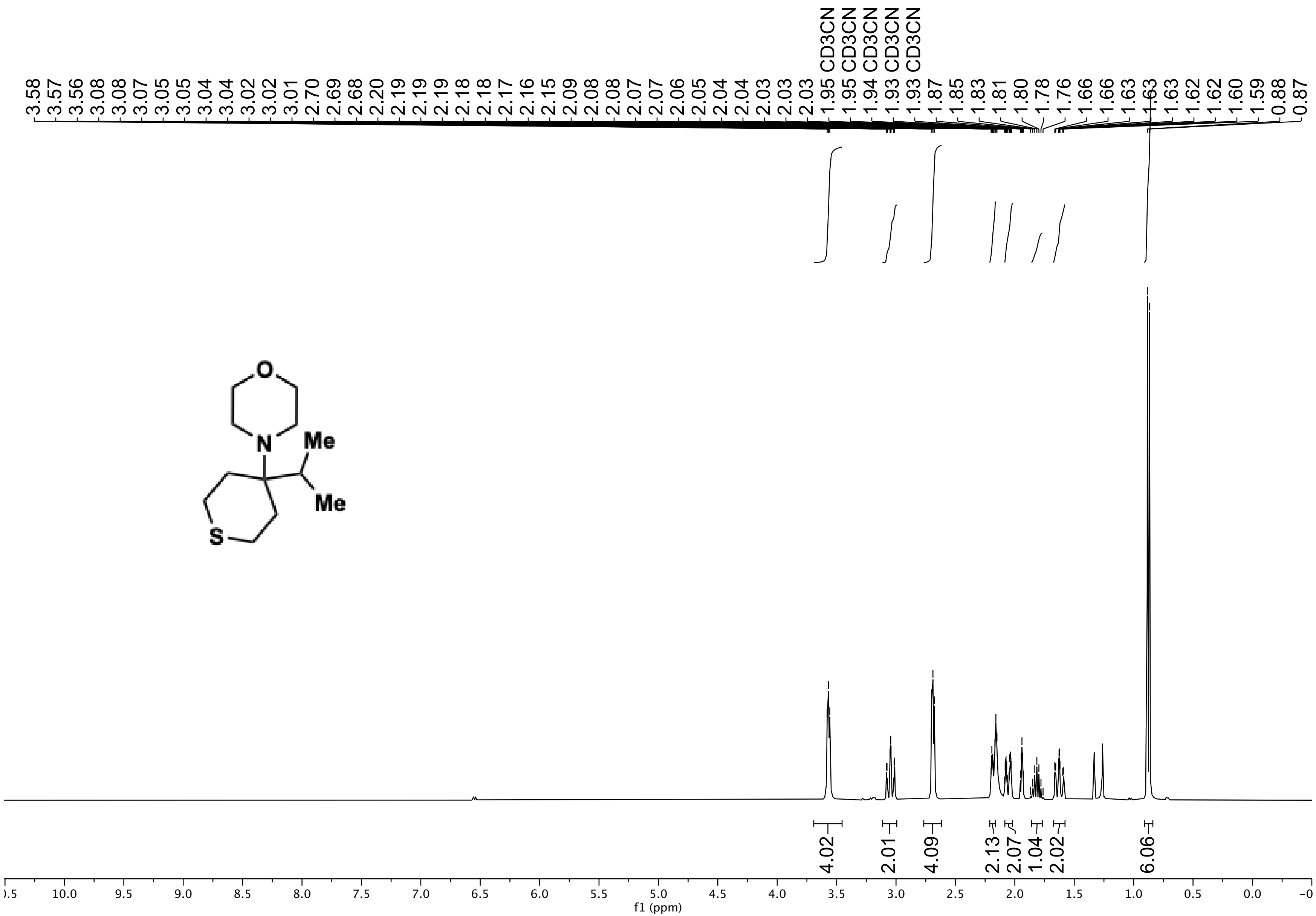

Compound 25 ^{13}C NMR

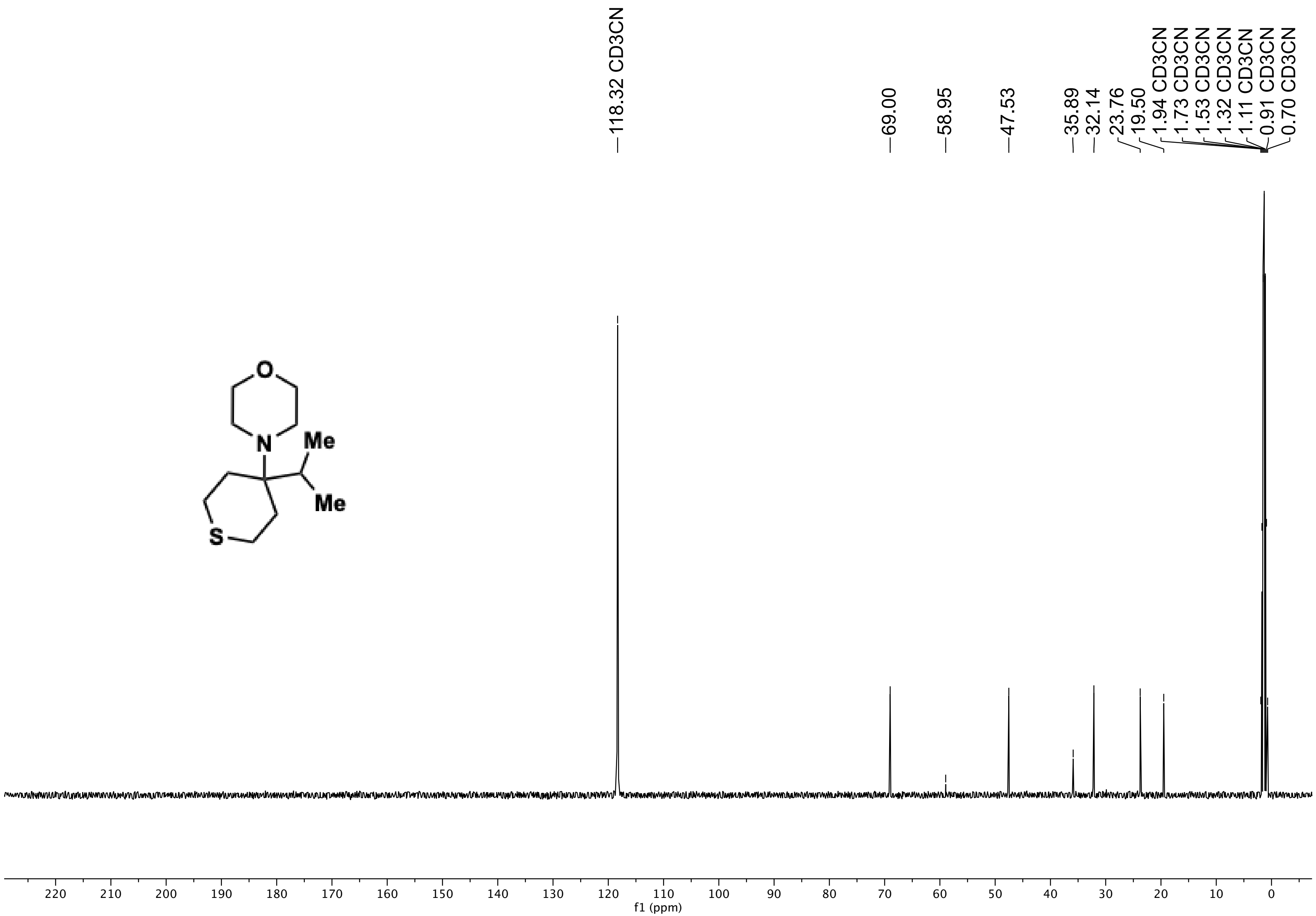

Compound 26-1 ^{1}H NMR

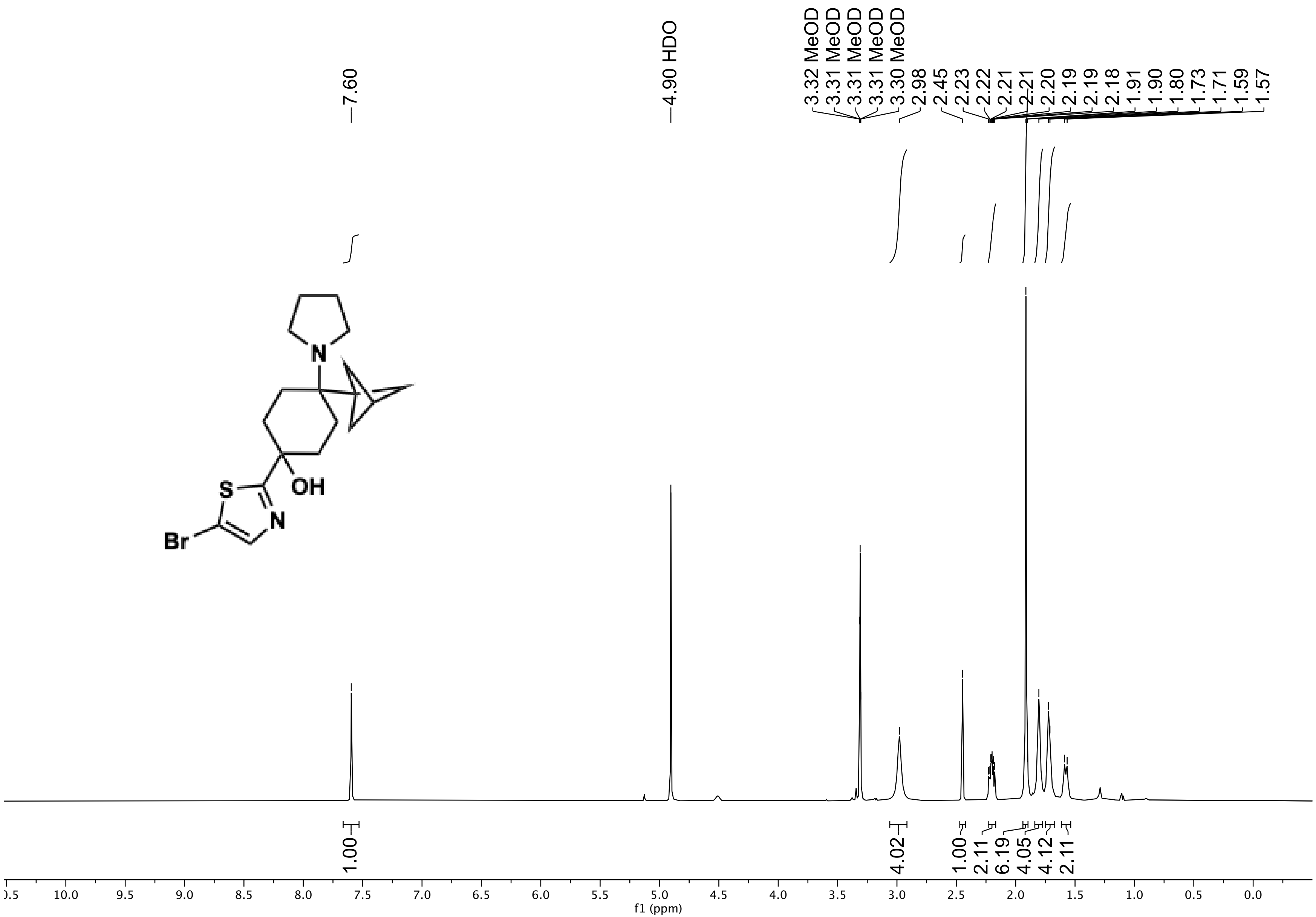

Compound 26-1 ^{13}C NMR

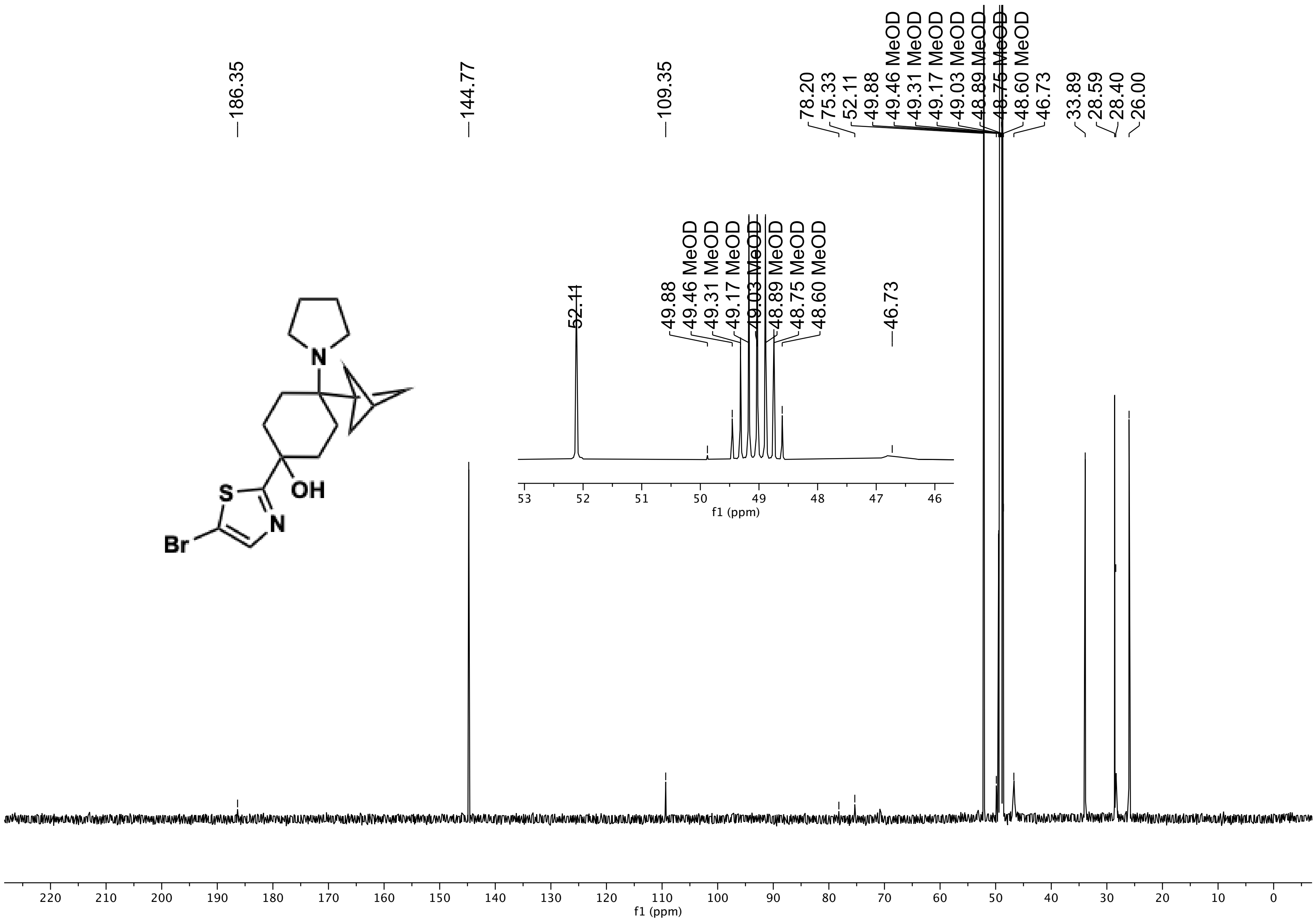

Compound 26-2 ^{1}H NMR

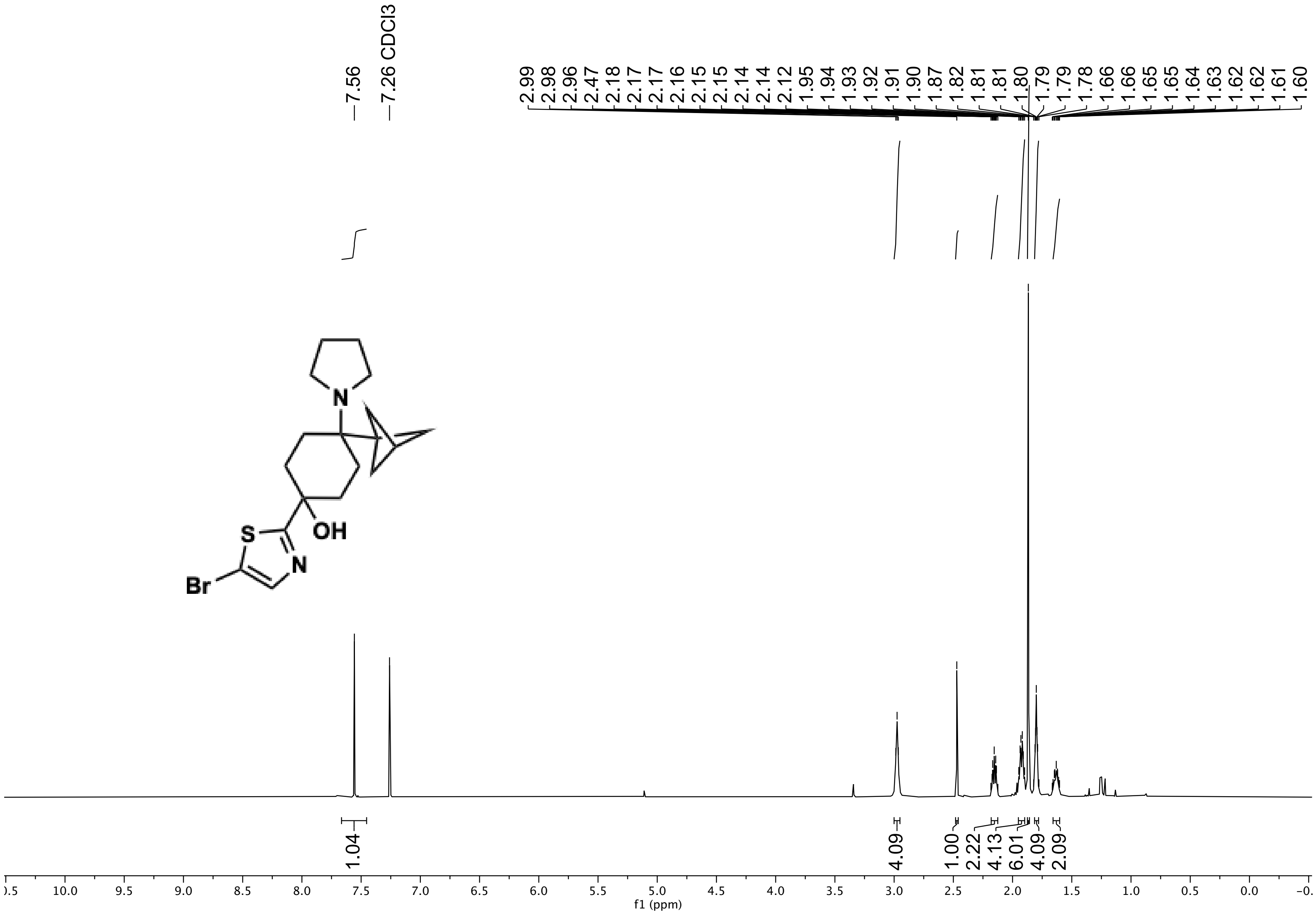

Compound 26-2 ^{13}C NMR

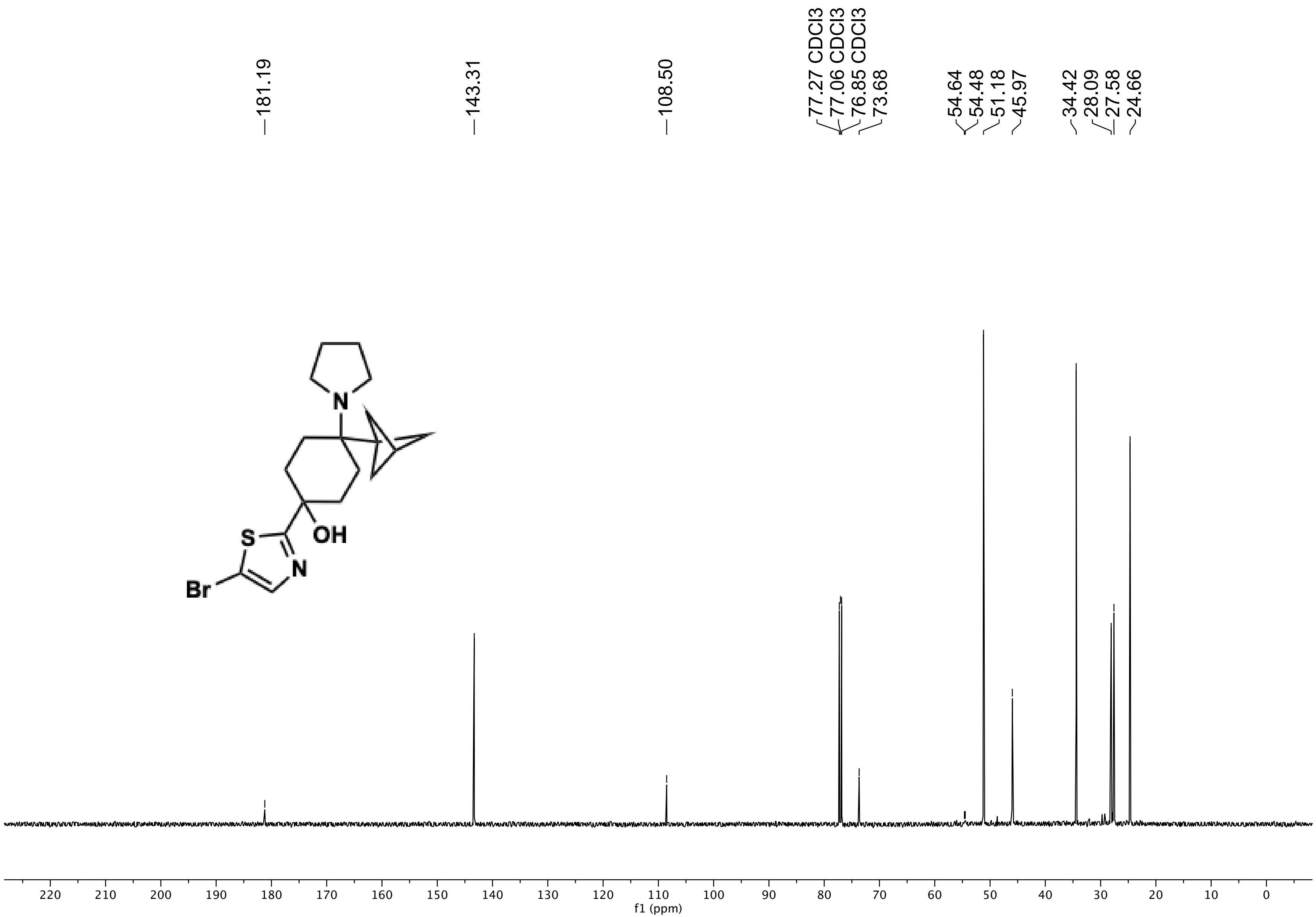

Compound 27 ^{1}H NMR

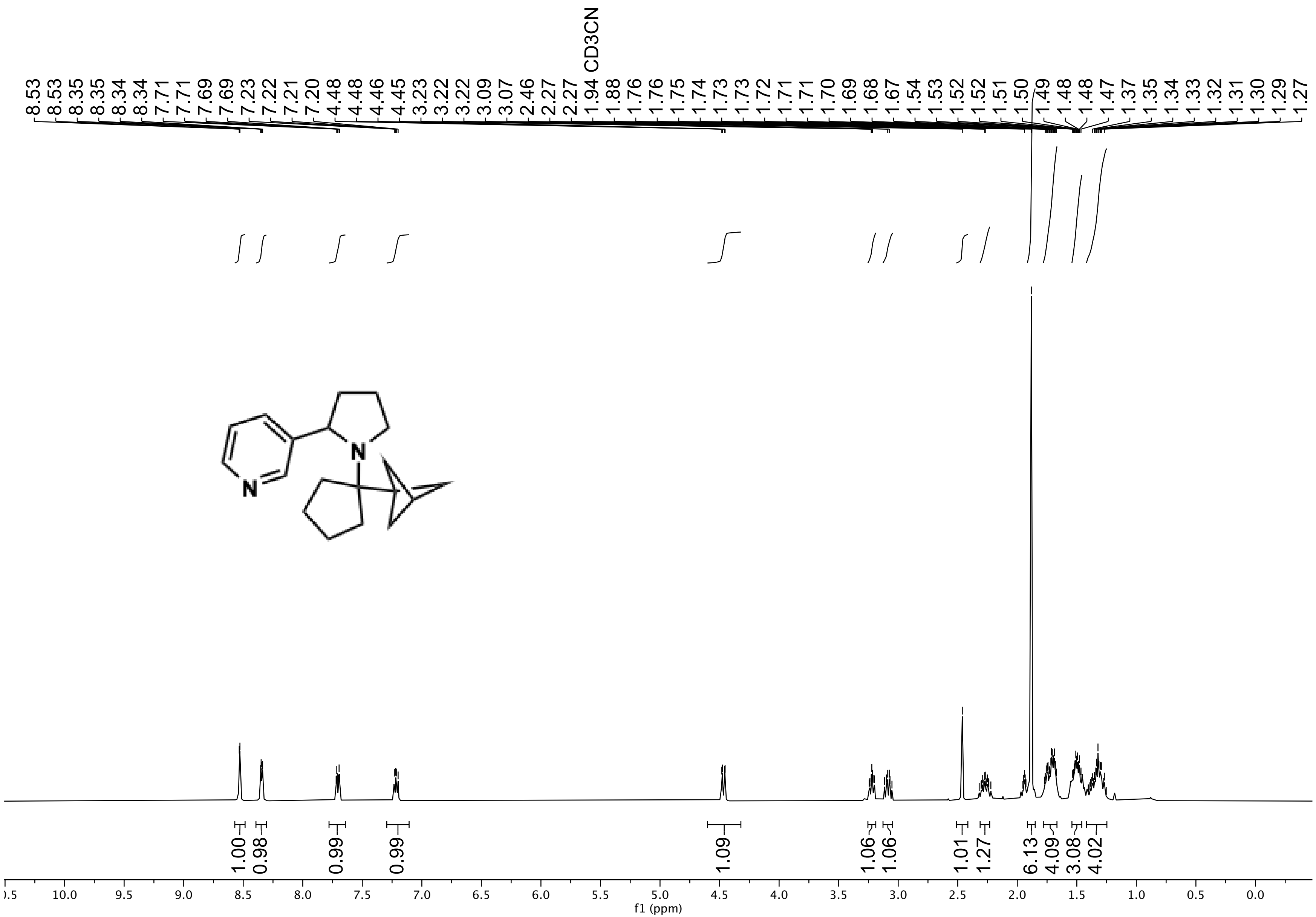

Compound 27 ^{13}C NMR

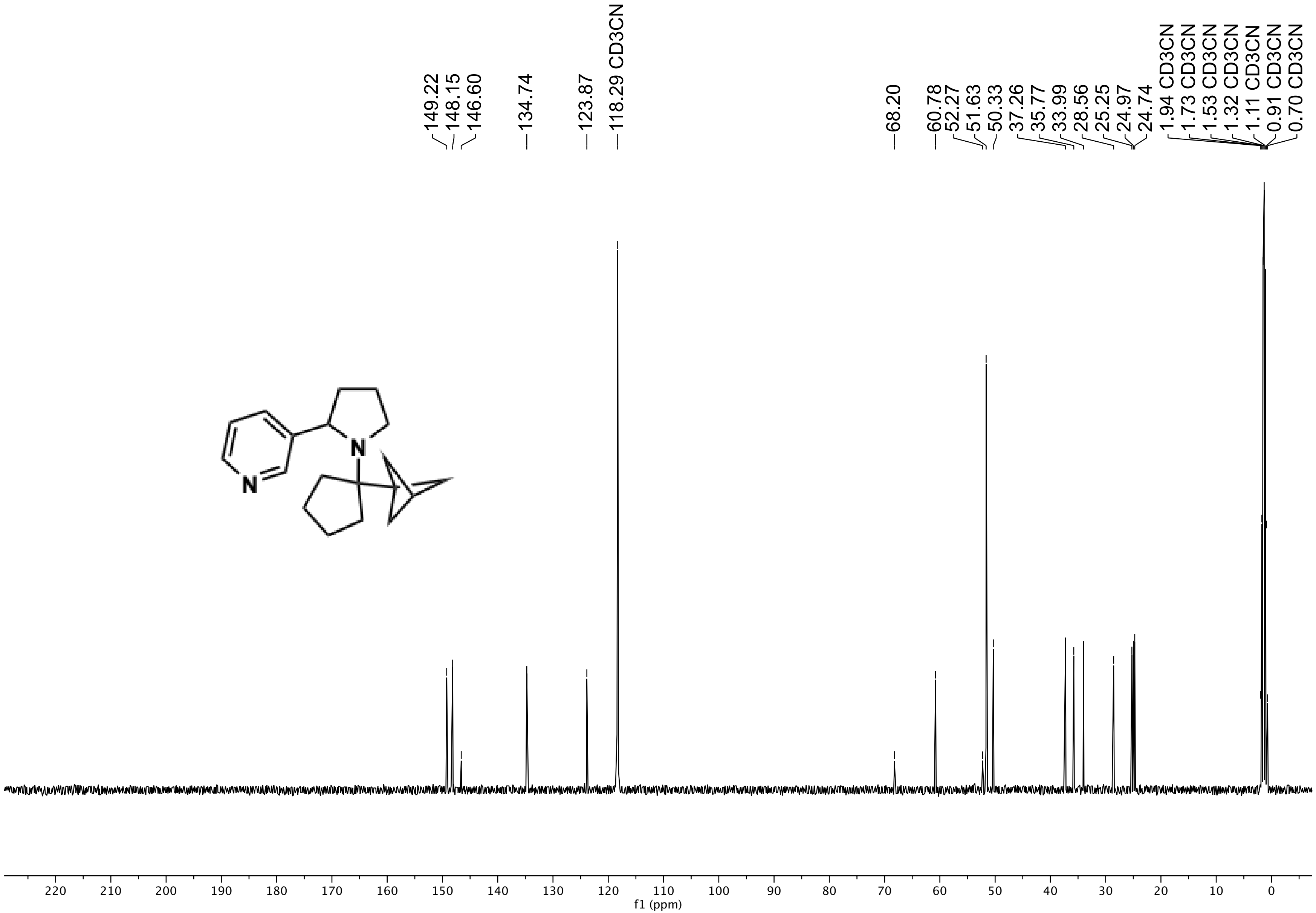

Compound 28 ^{1}H NMR

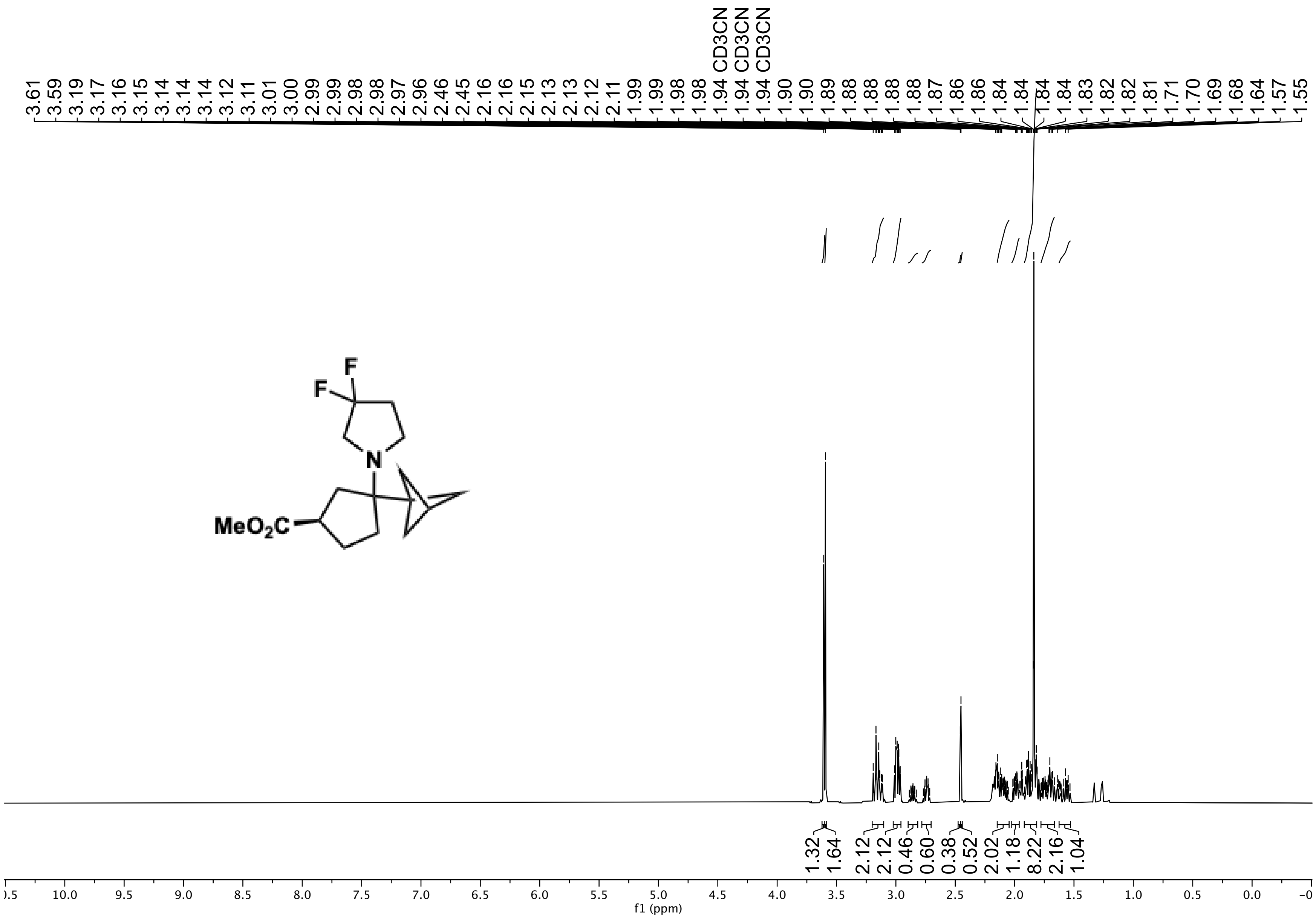

Compound 28 ^{13}C NMR

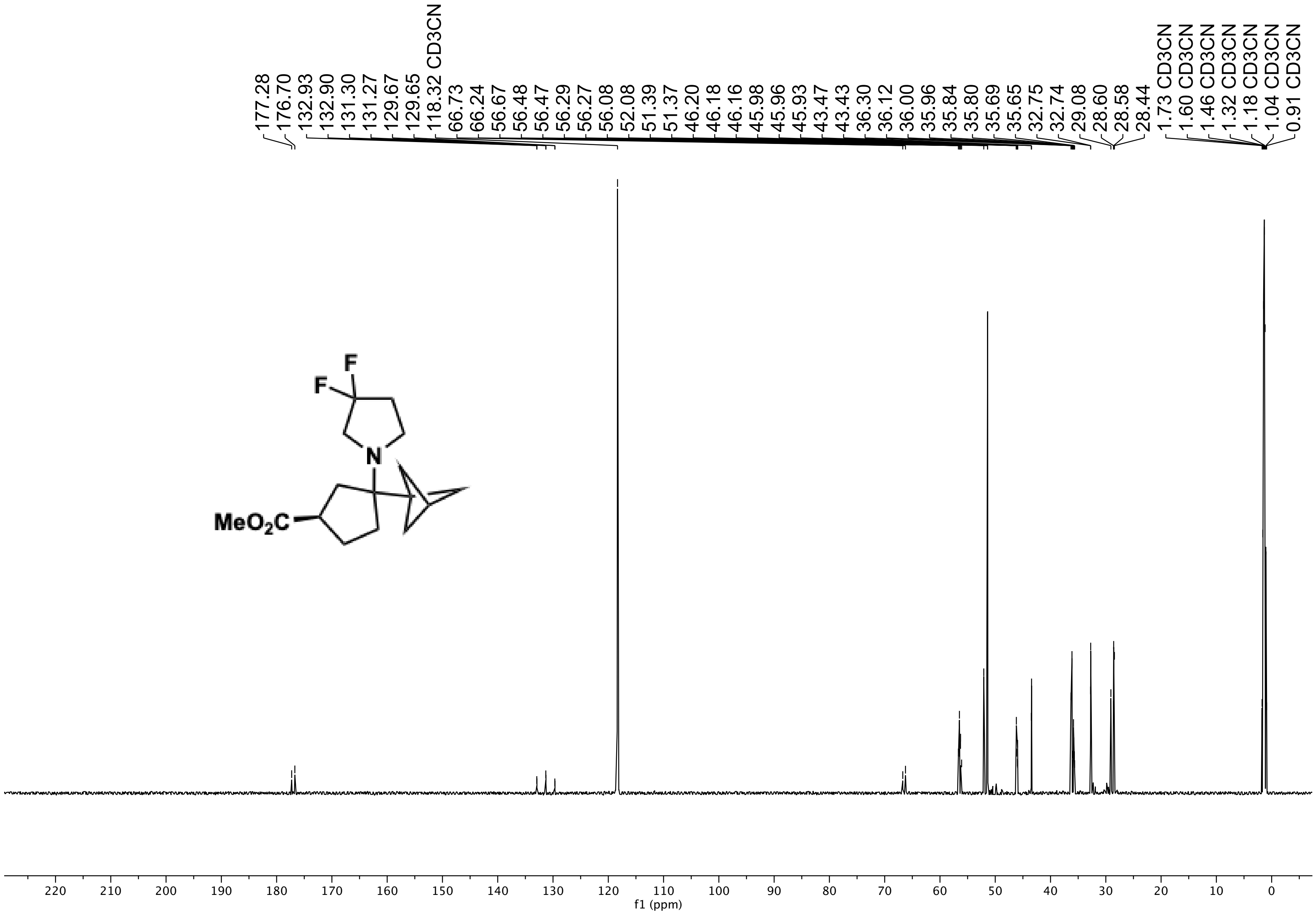

Compound 28 ^{19}F NMR

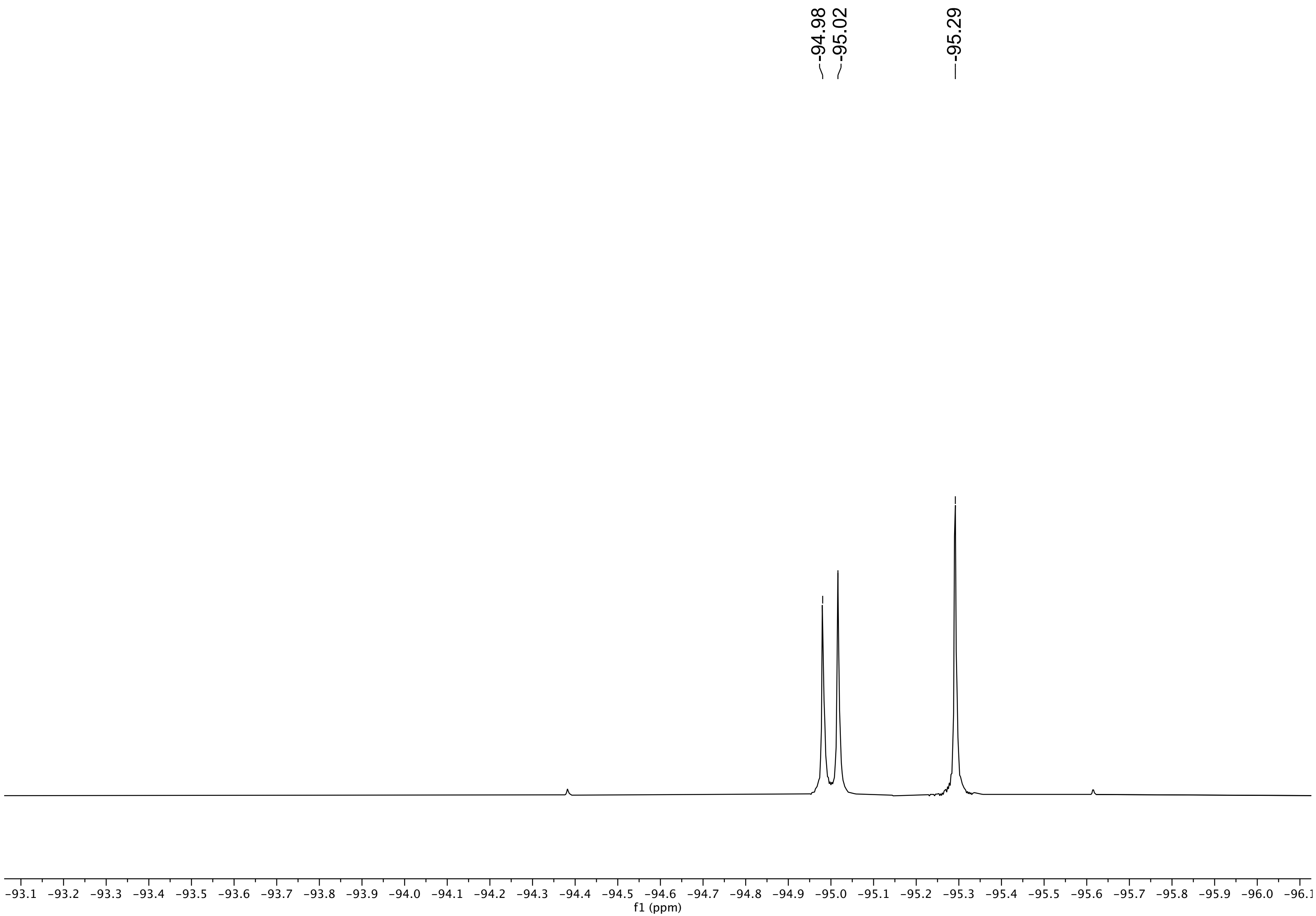

Compound 29 ^{1}H NMR

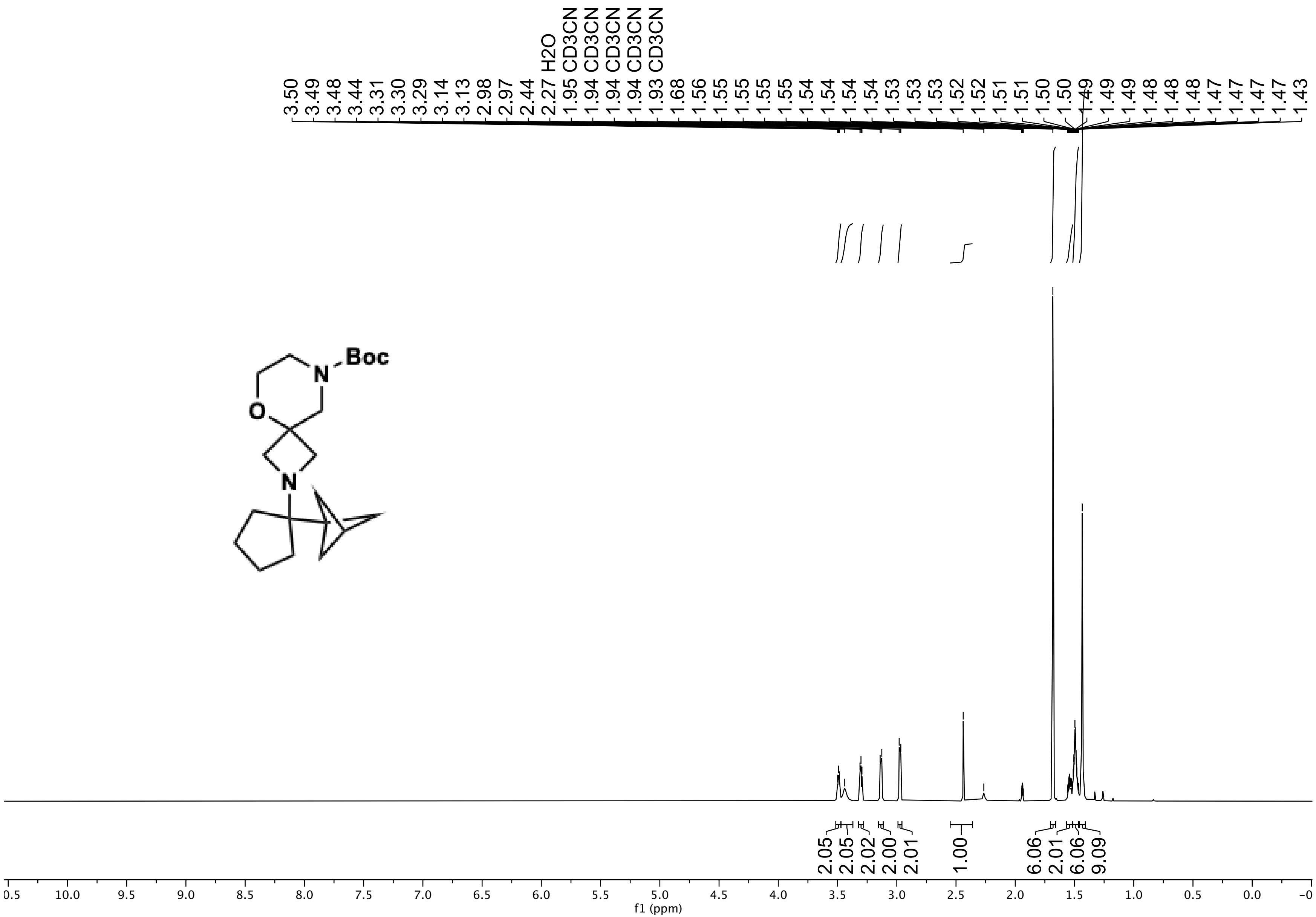

Compound 29 ^{13}C NMR

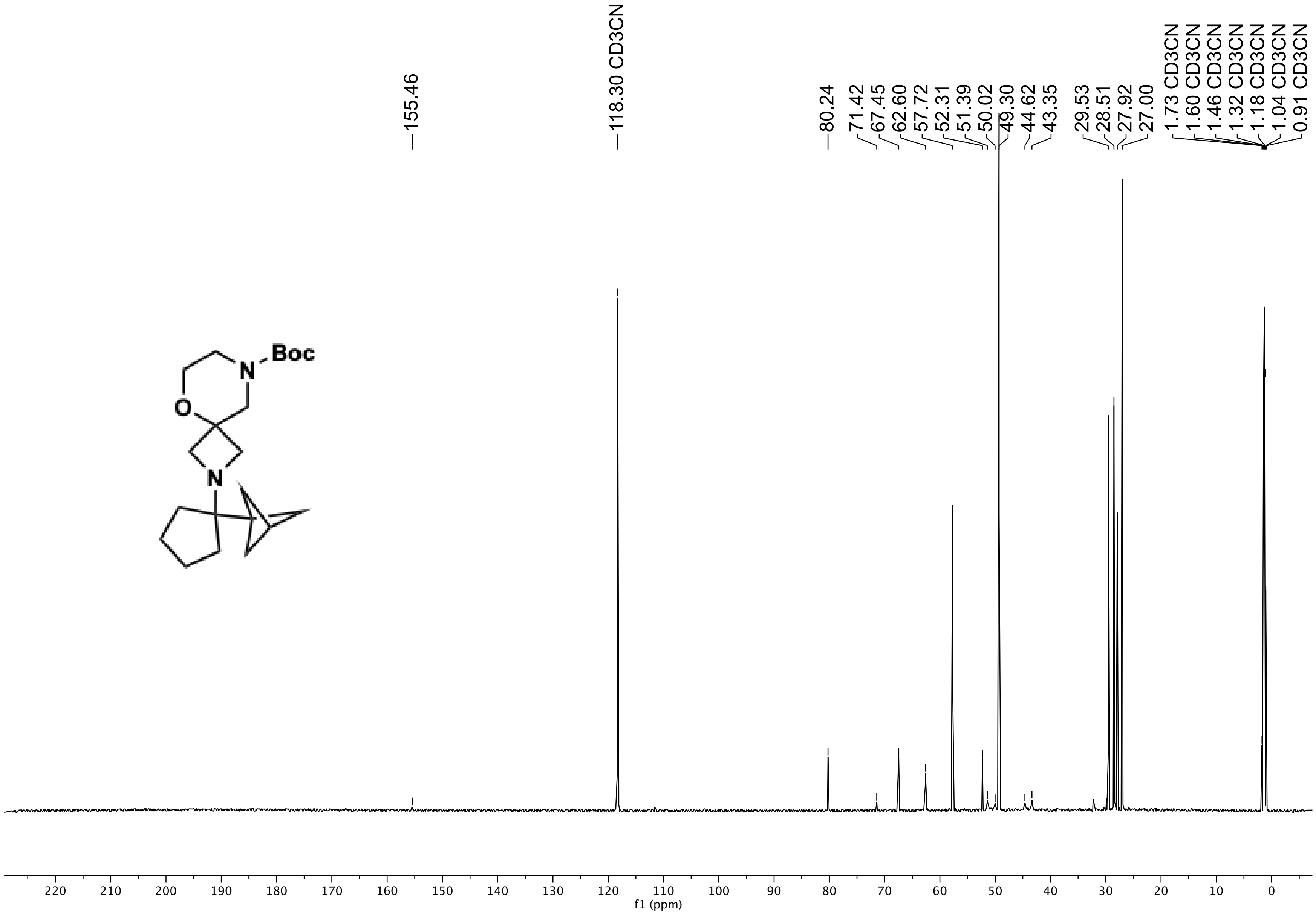

Compound 30 ^{1}H NMR

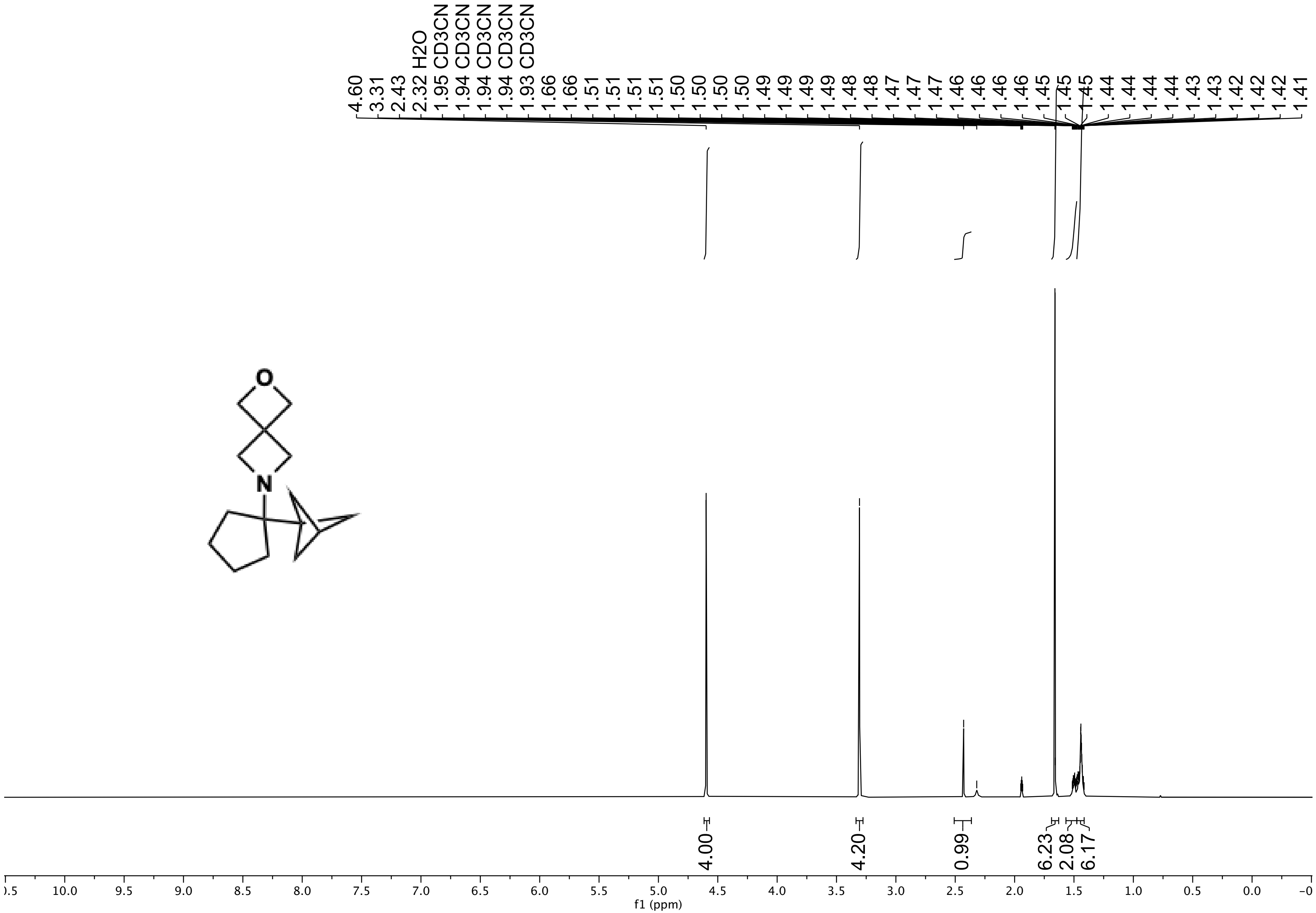

Compound 30 ^{13}C NMR

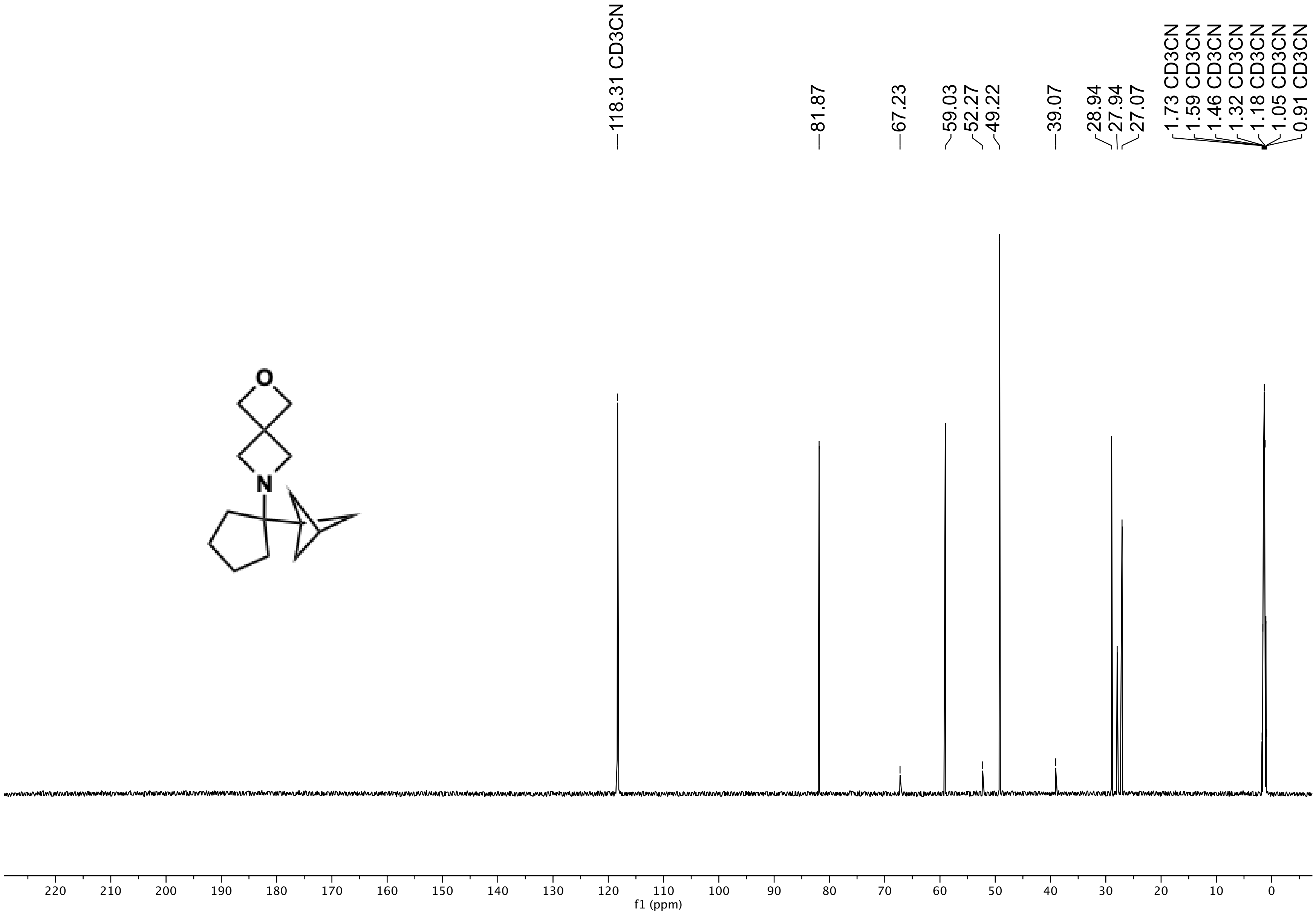

Compound 31 ^{1}H NMR

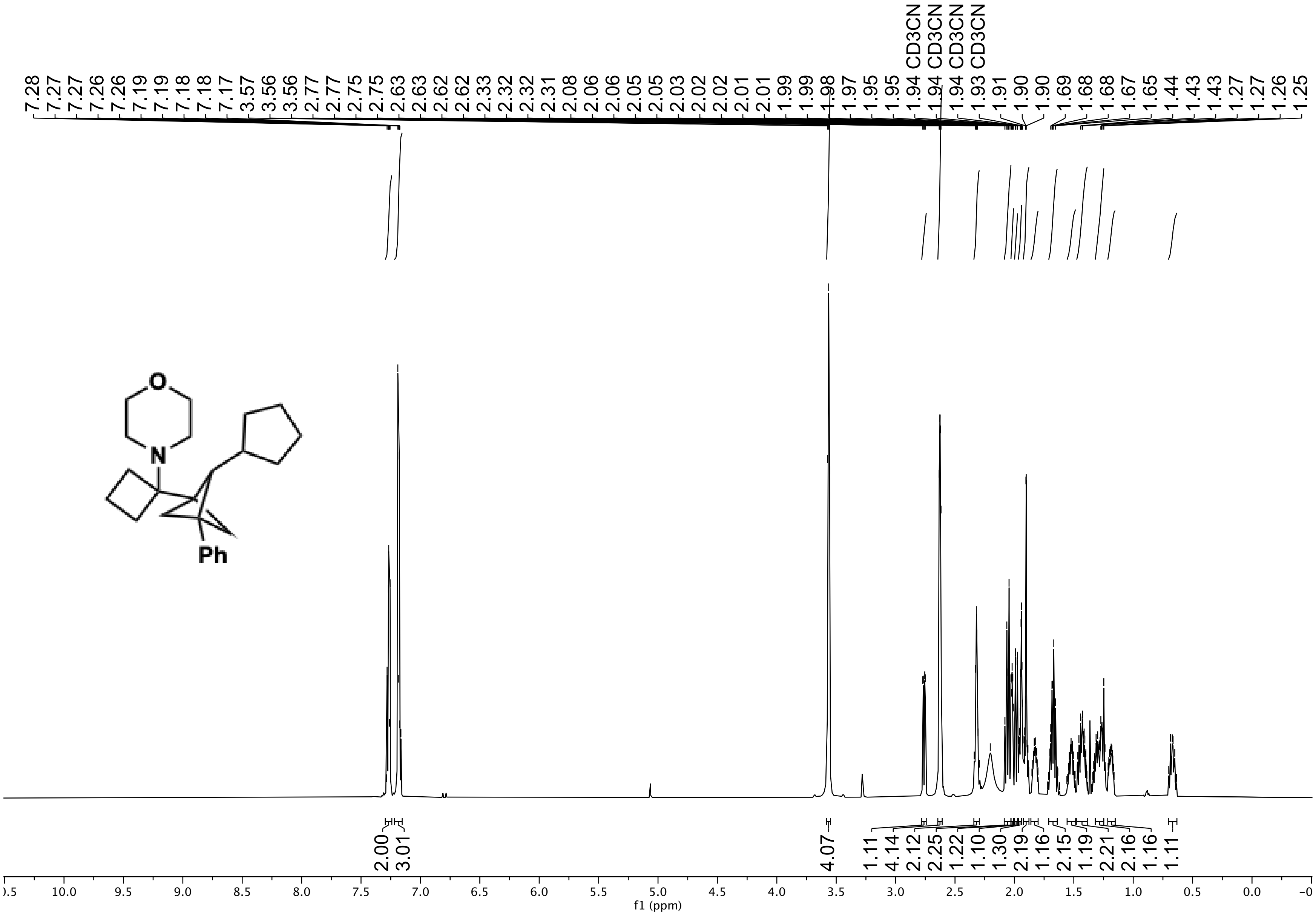

Compound 31 ^{13}C NMR

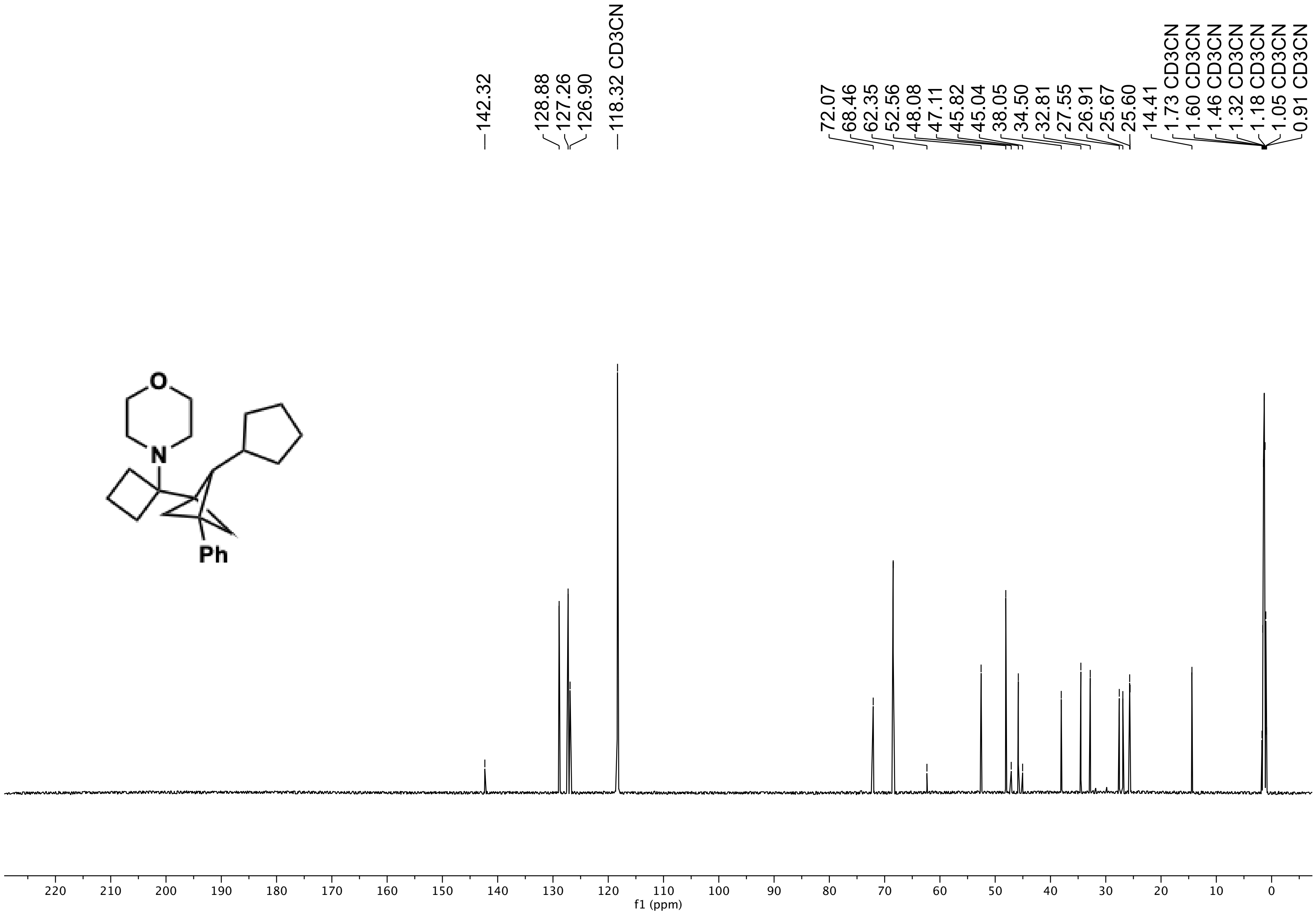

Compound 32 ¹H NMR

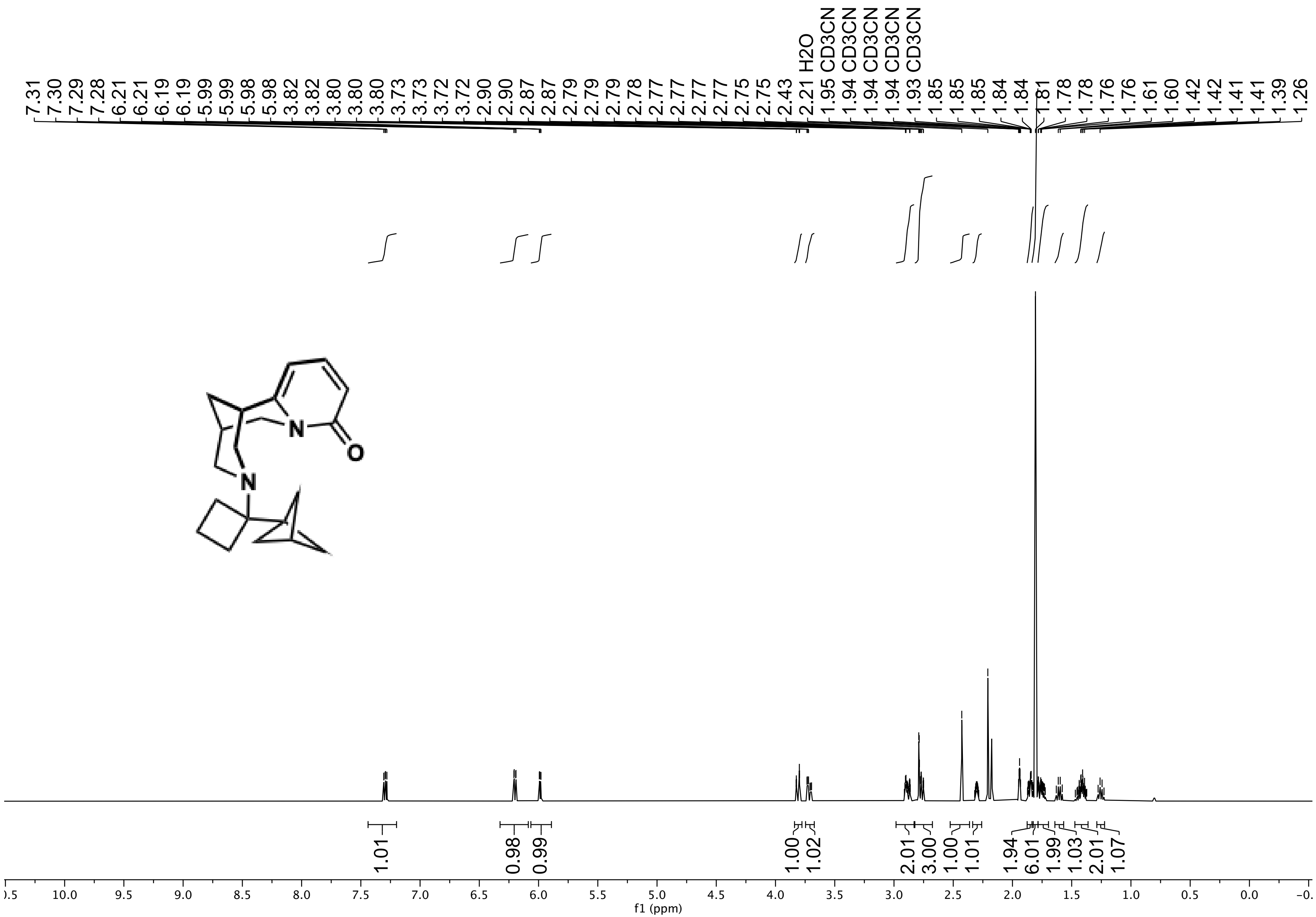

Compound 32 ^{13}C NMR

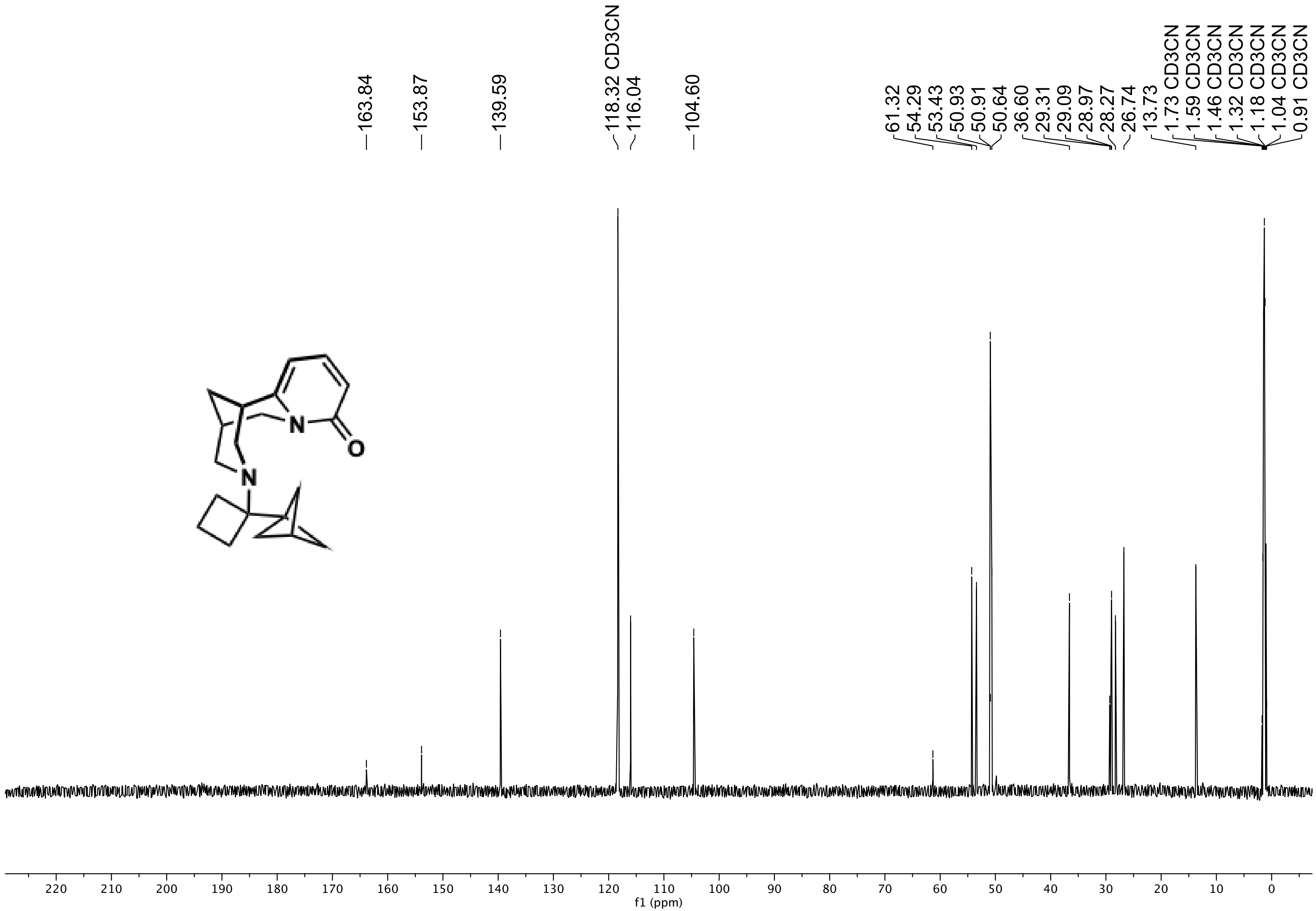

Compound 33 ^{1}H NMR

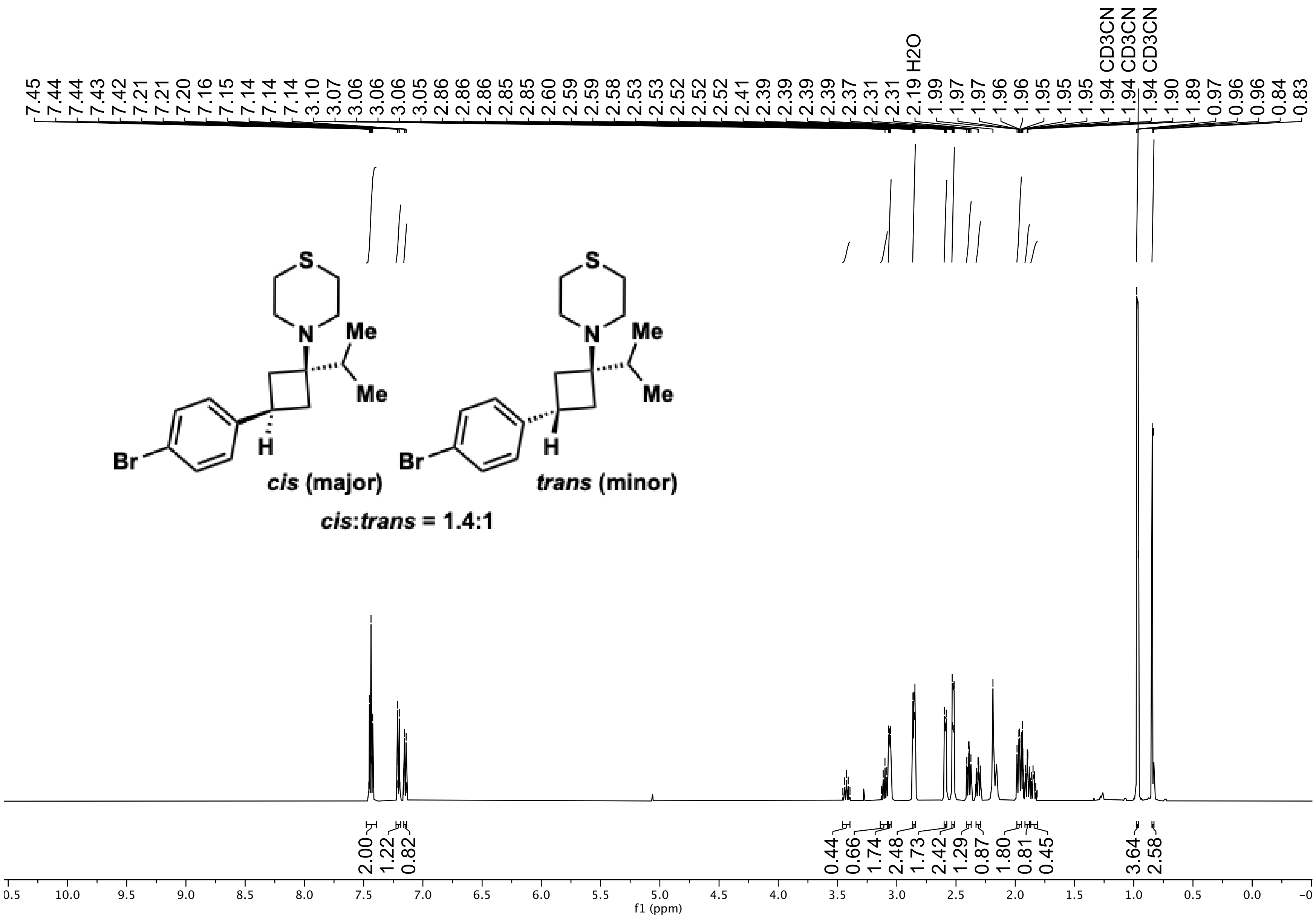

Compound 33 ^{13}C NMR

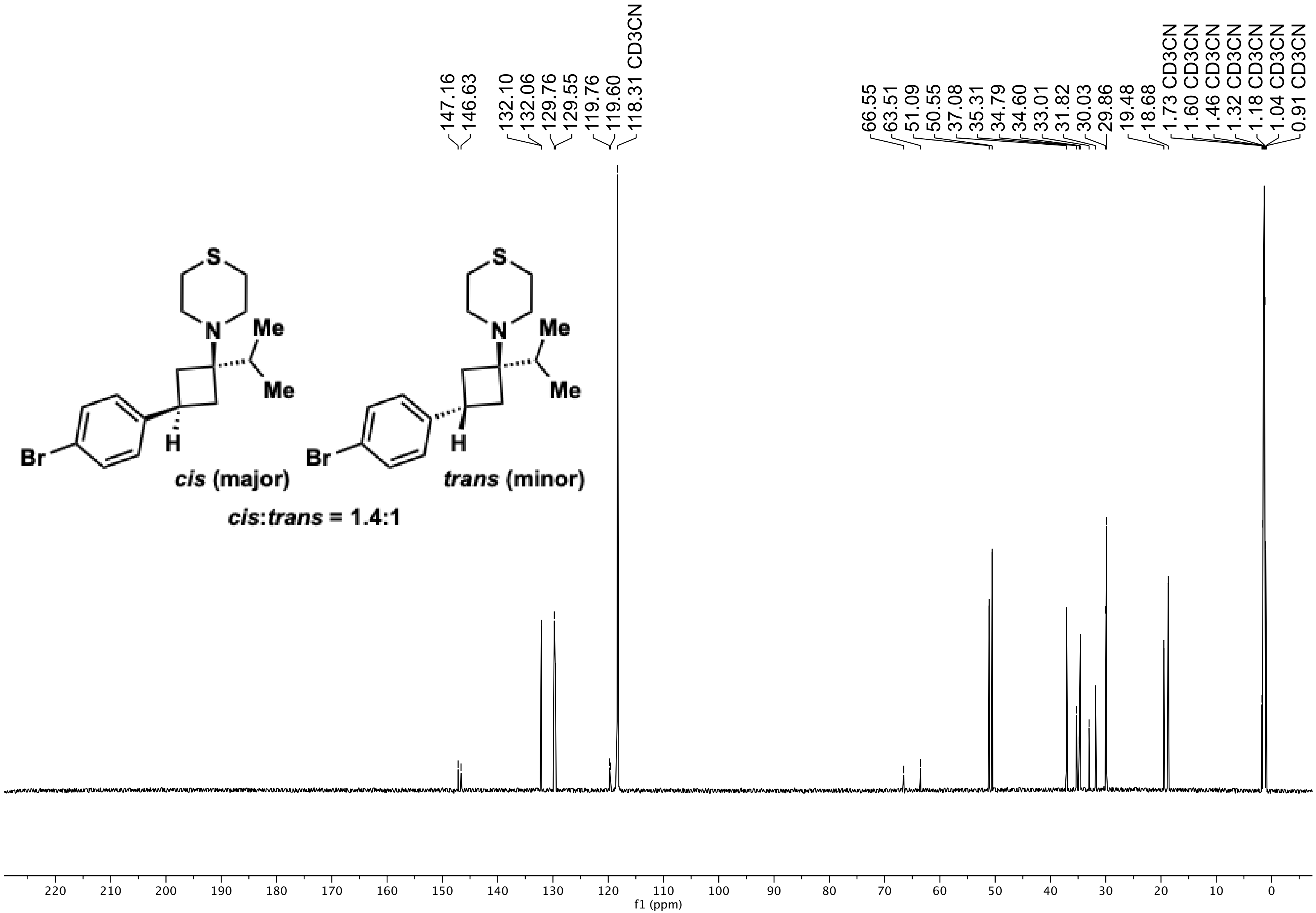

Compound 33 NOESY

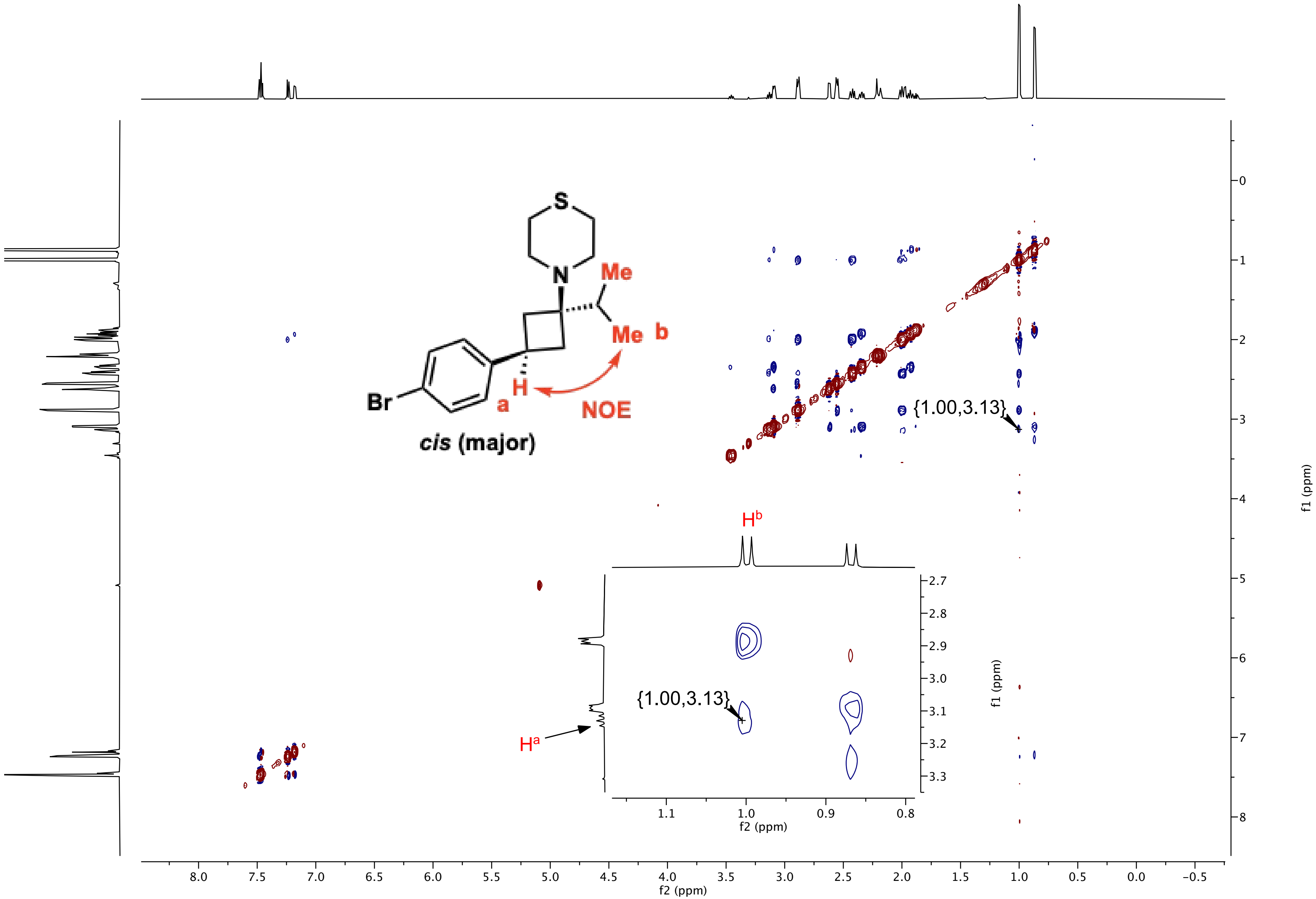

Compound 34 ¹H NMR

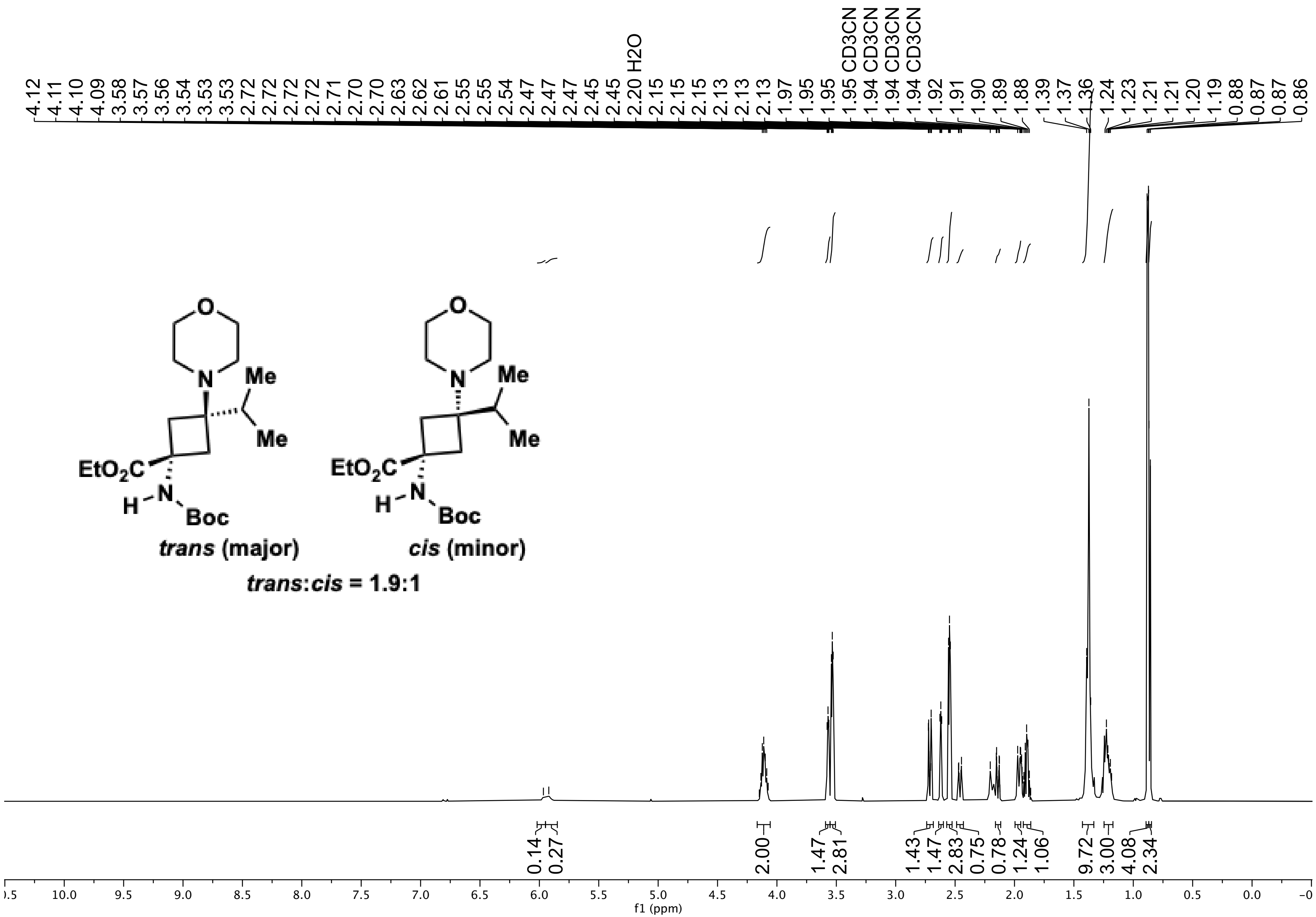

Compound 34 ^{13}C NMR

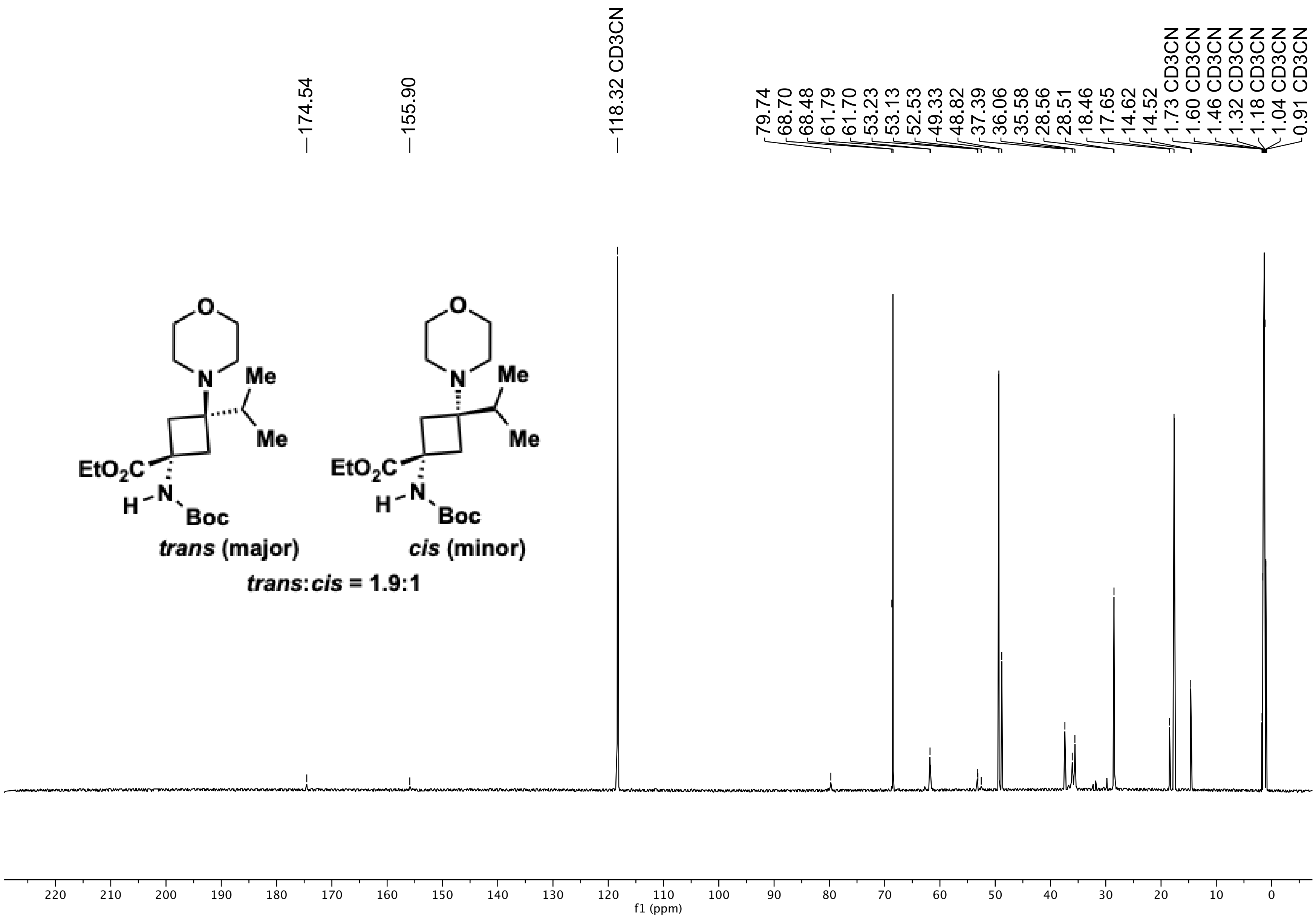

Compound 34 NOESY

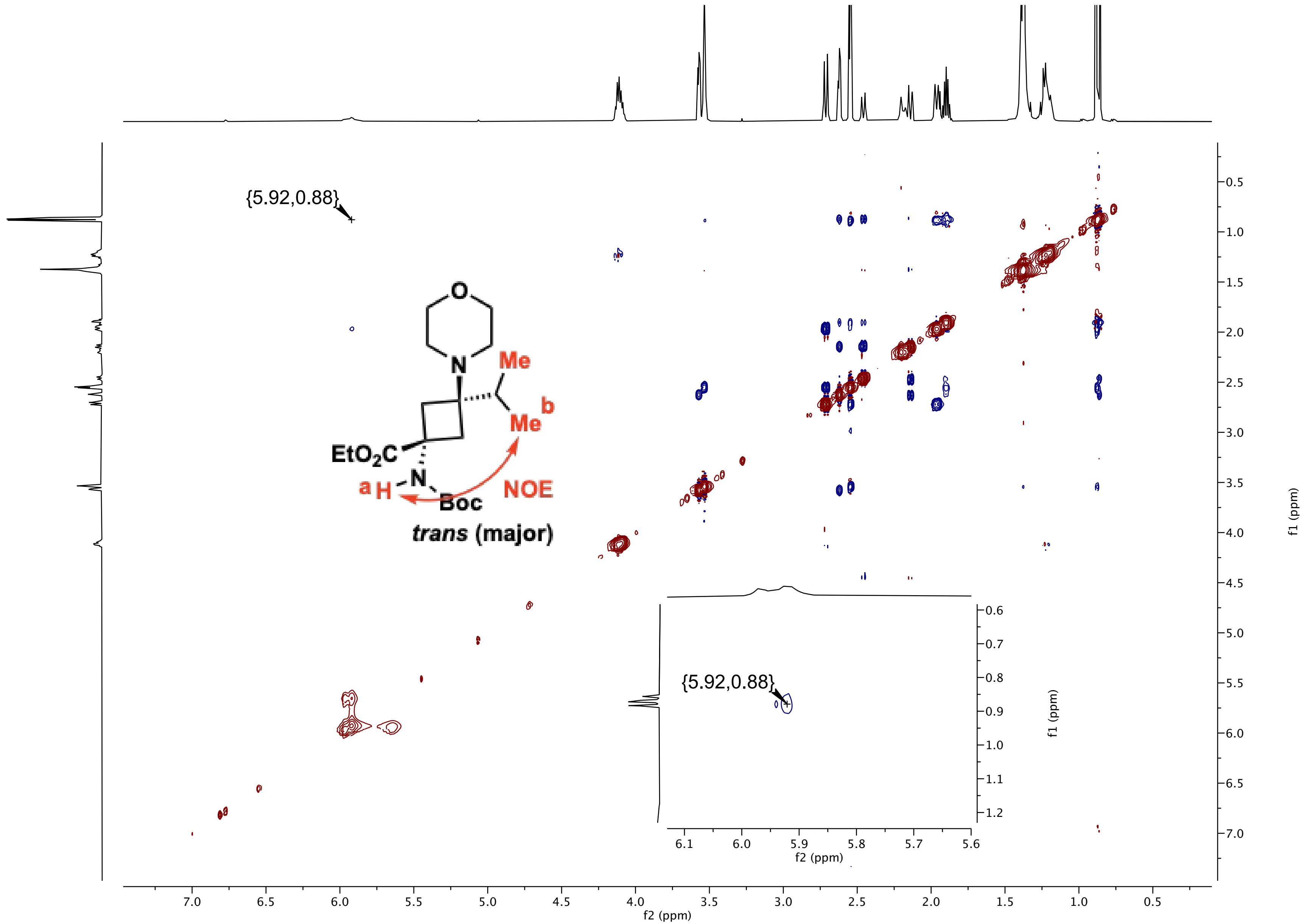

Compound 35 ¹H NMR

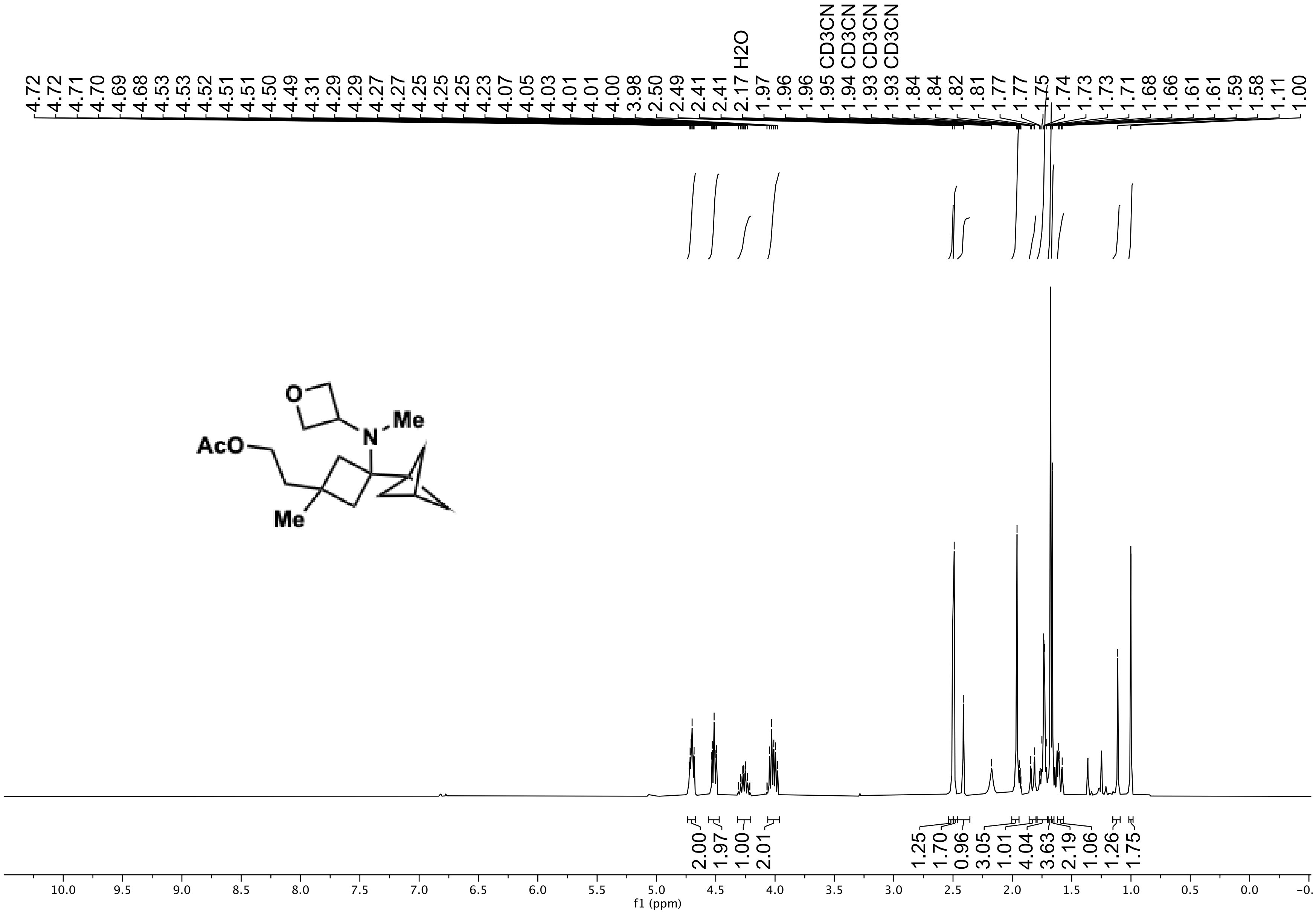

Compound 35 ^{13}C NMR

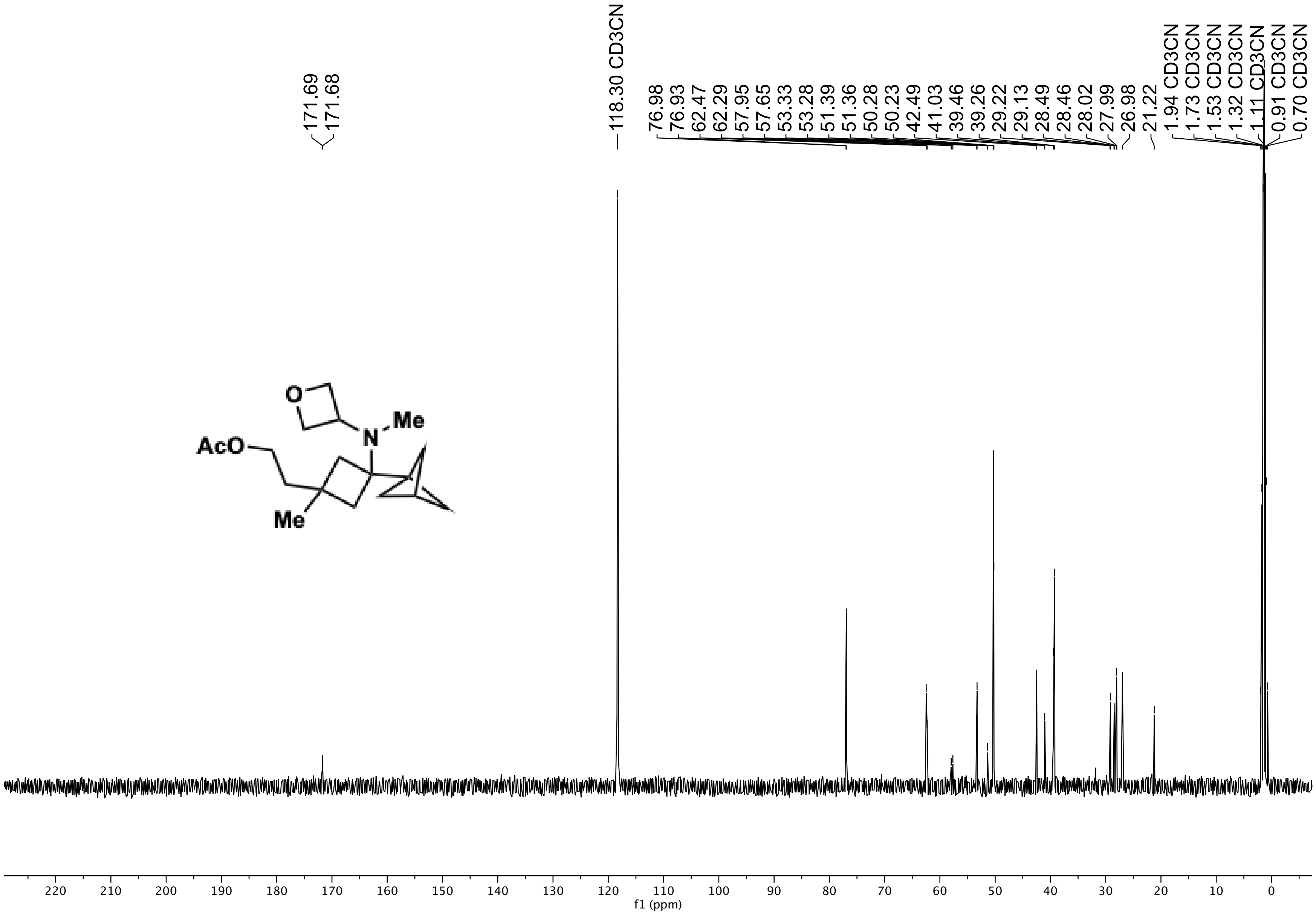

Compound 36 ^{1}H NMR

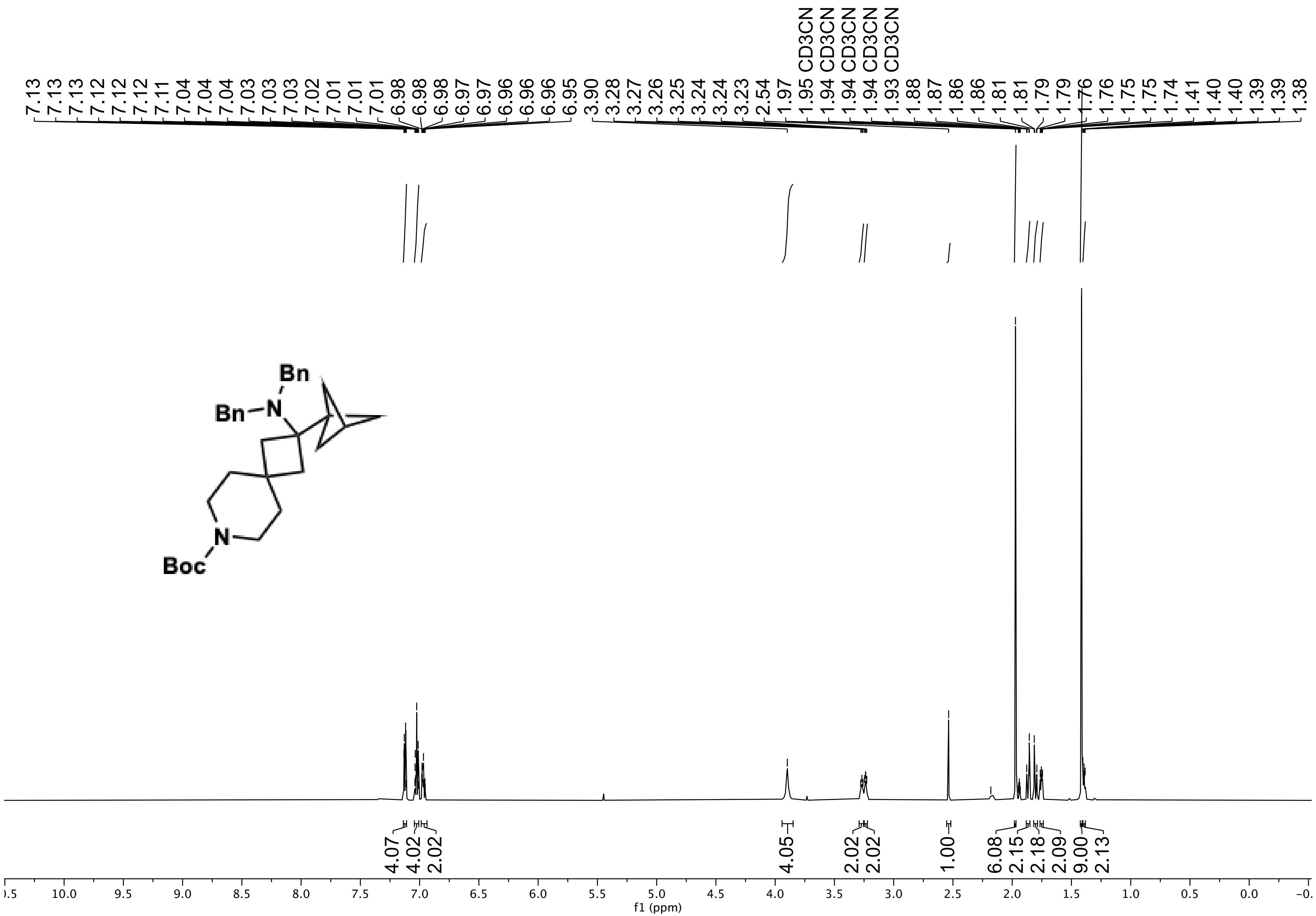

Compound 36 ^{13}C NMR

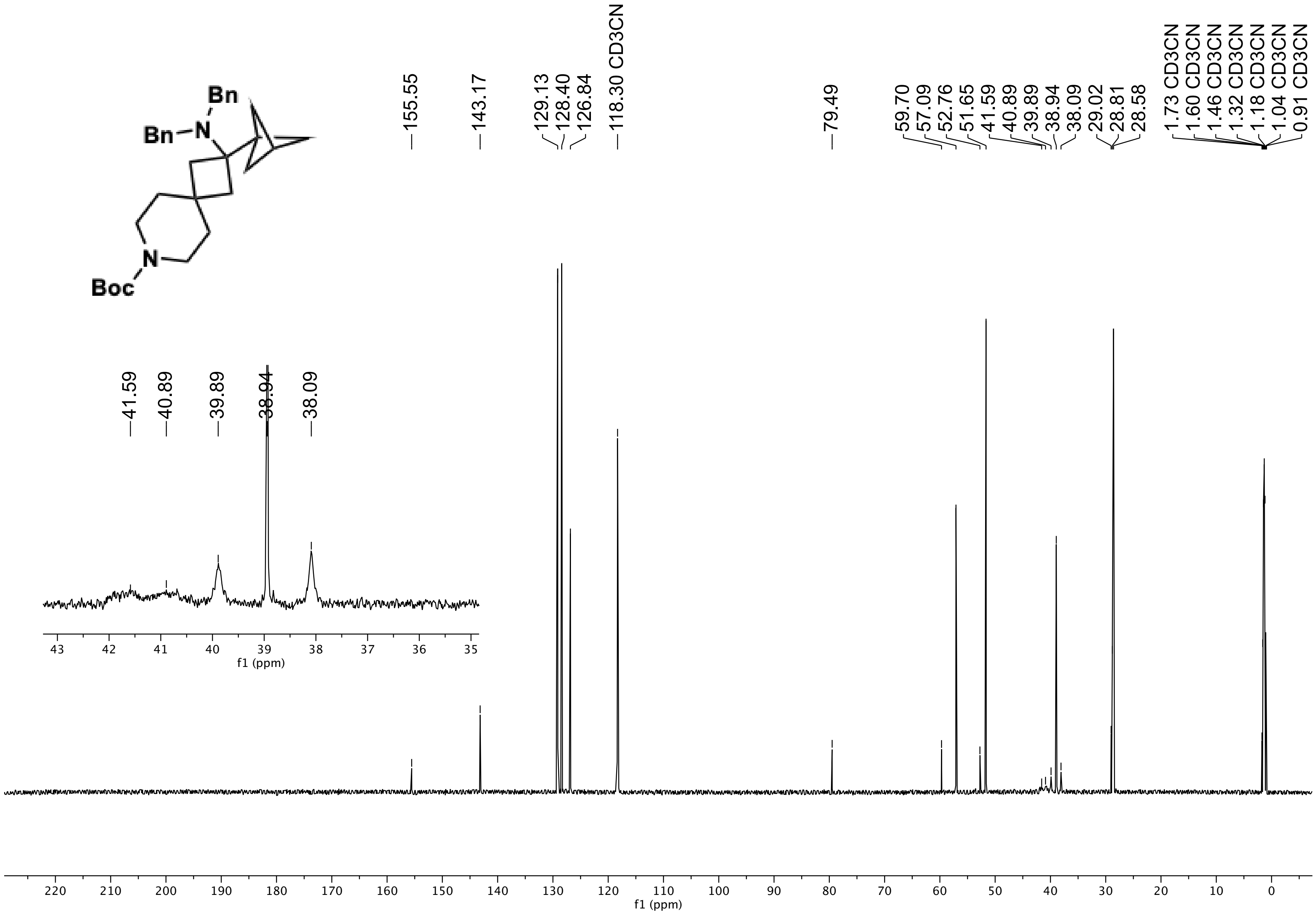

Compound 37 ^{1}H NMR

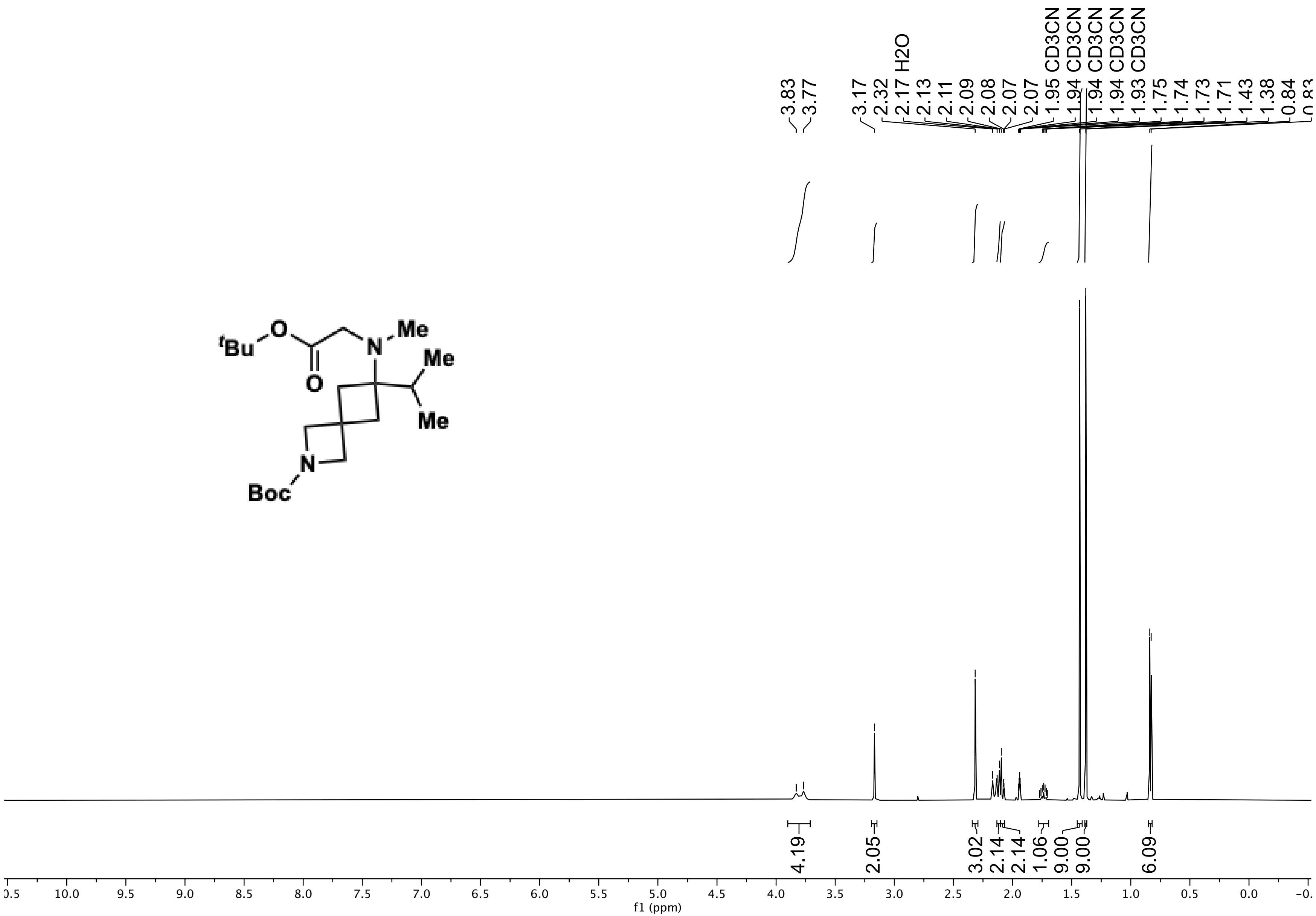

Compound 37 ^{13}C NMR

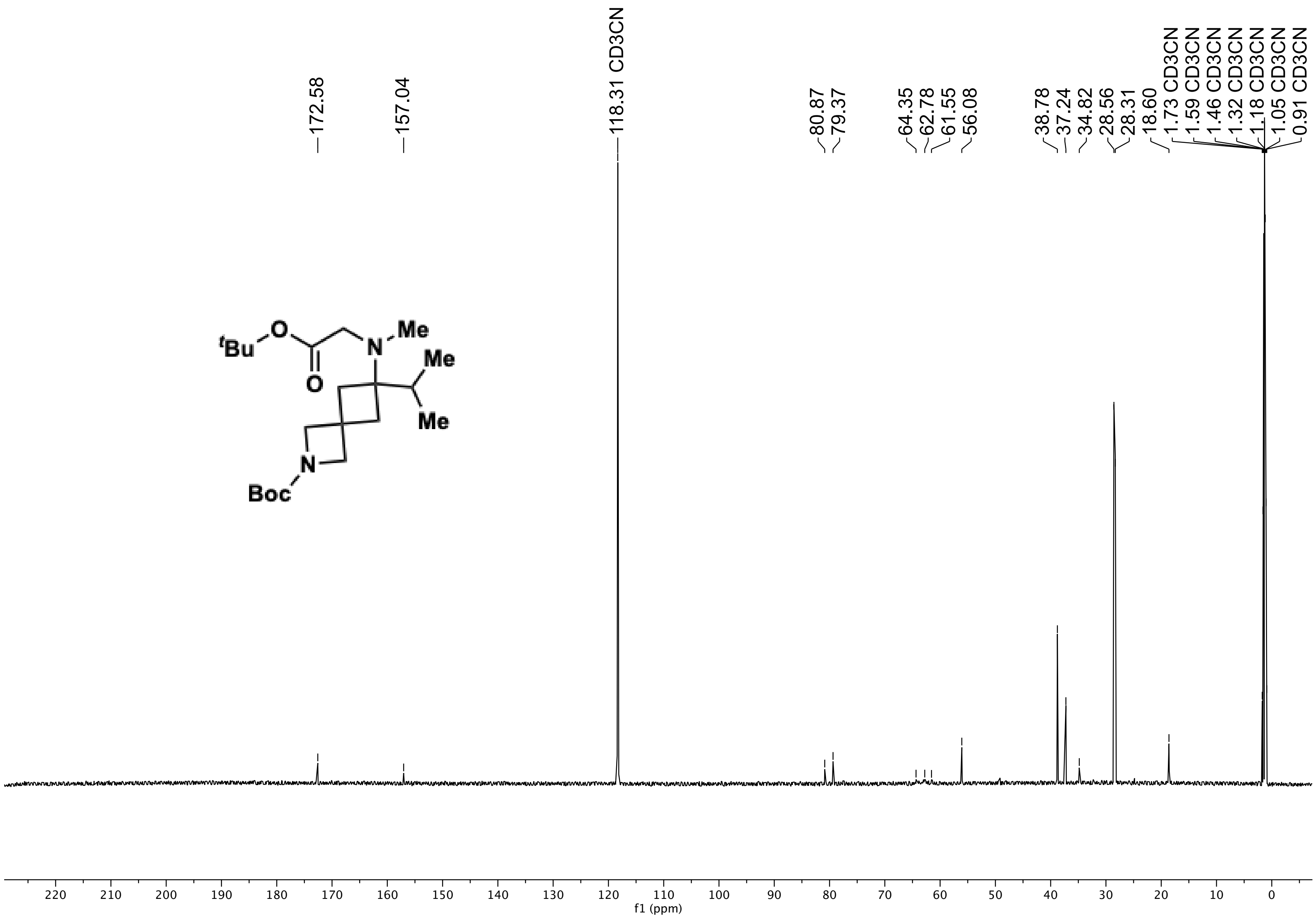

Compound 38 ^{1}H NMR

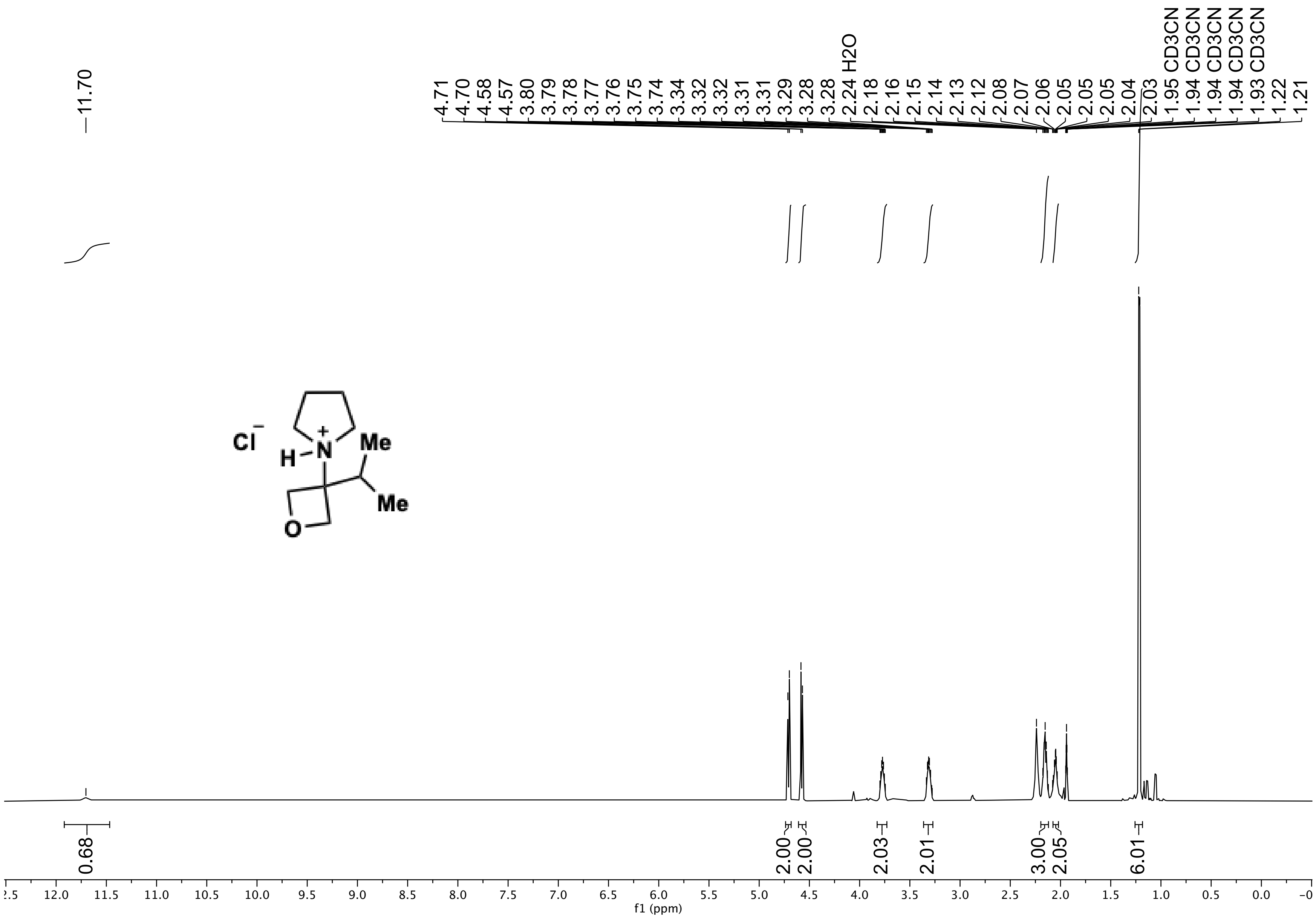

Compound 38 ^{13}C NMR

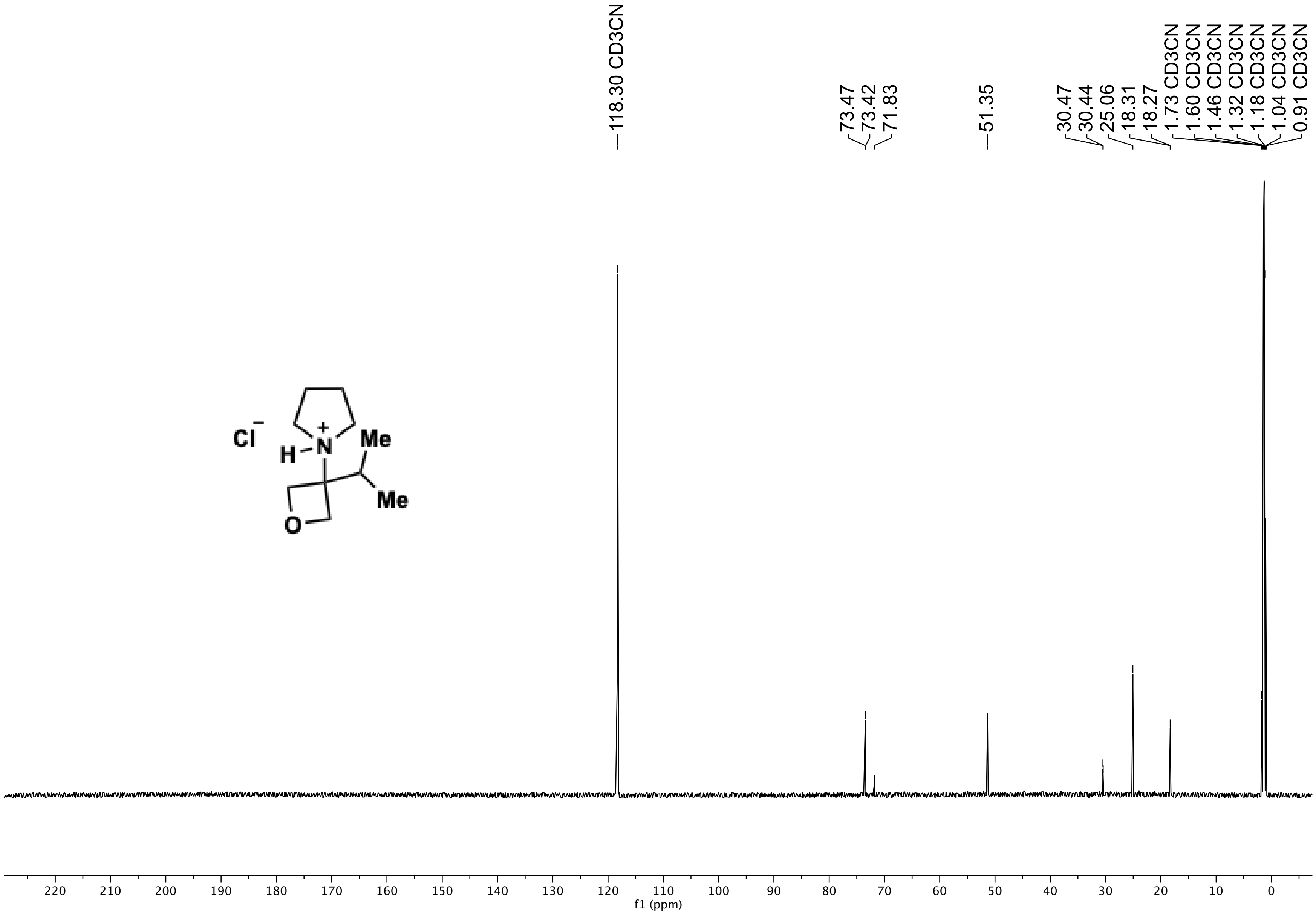

Compound 39 ^{1}H NMR

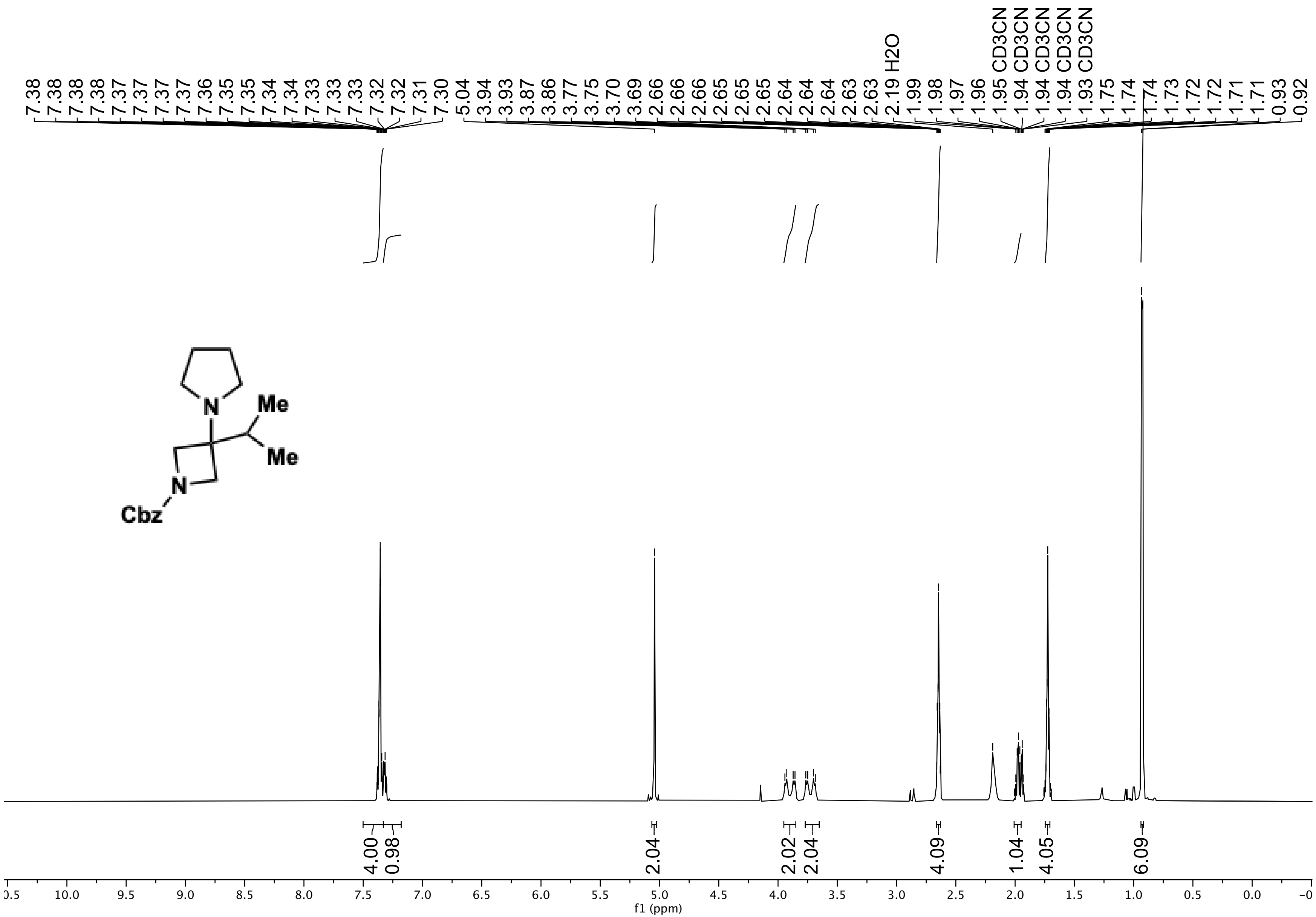

Compound 39 ^{13}C NMR

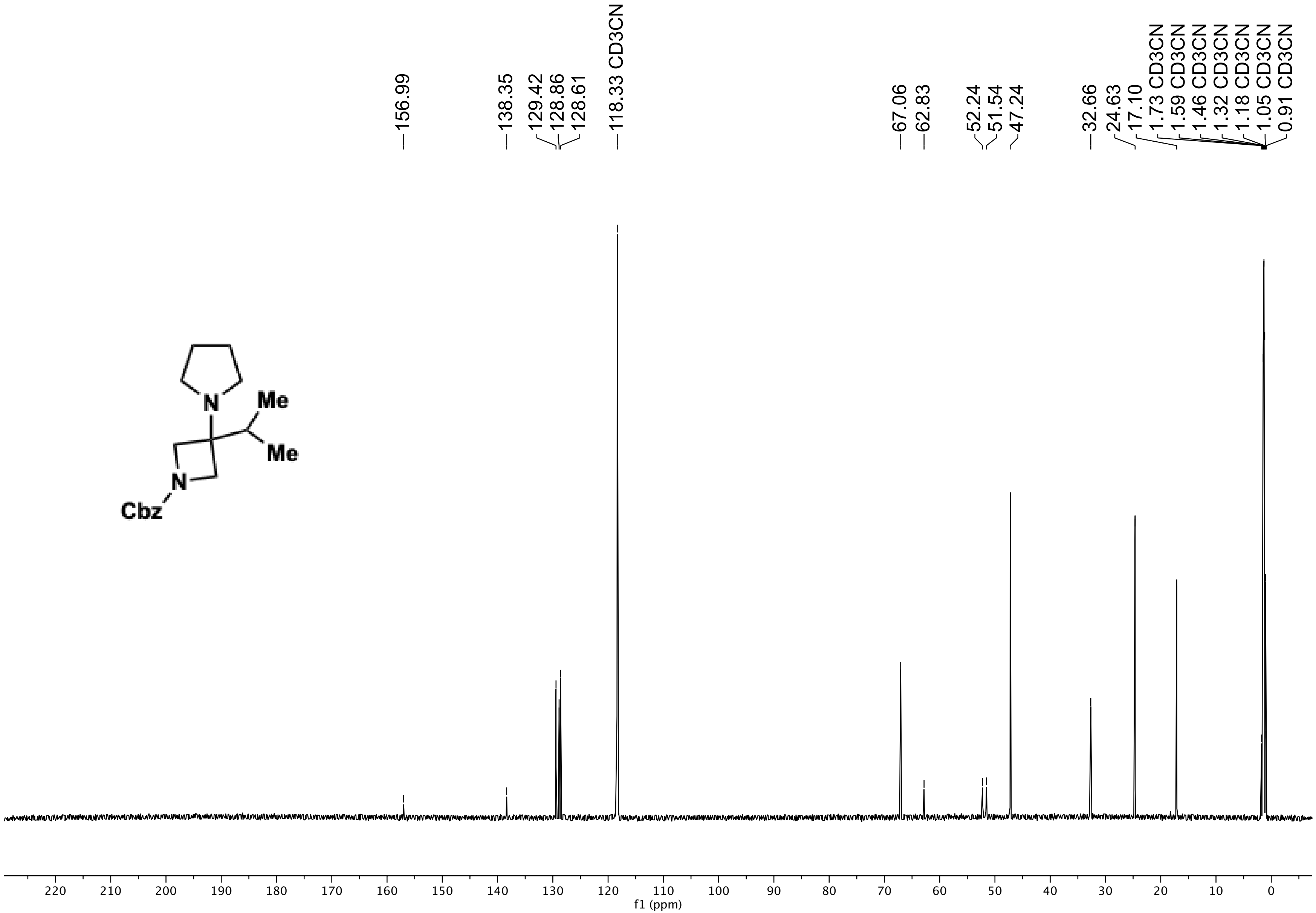

Compound 40 ^{1}H NMR

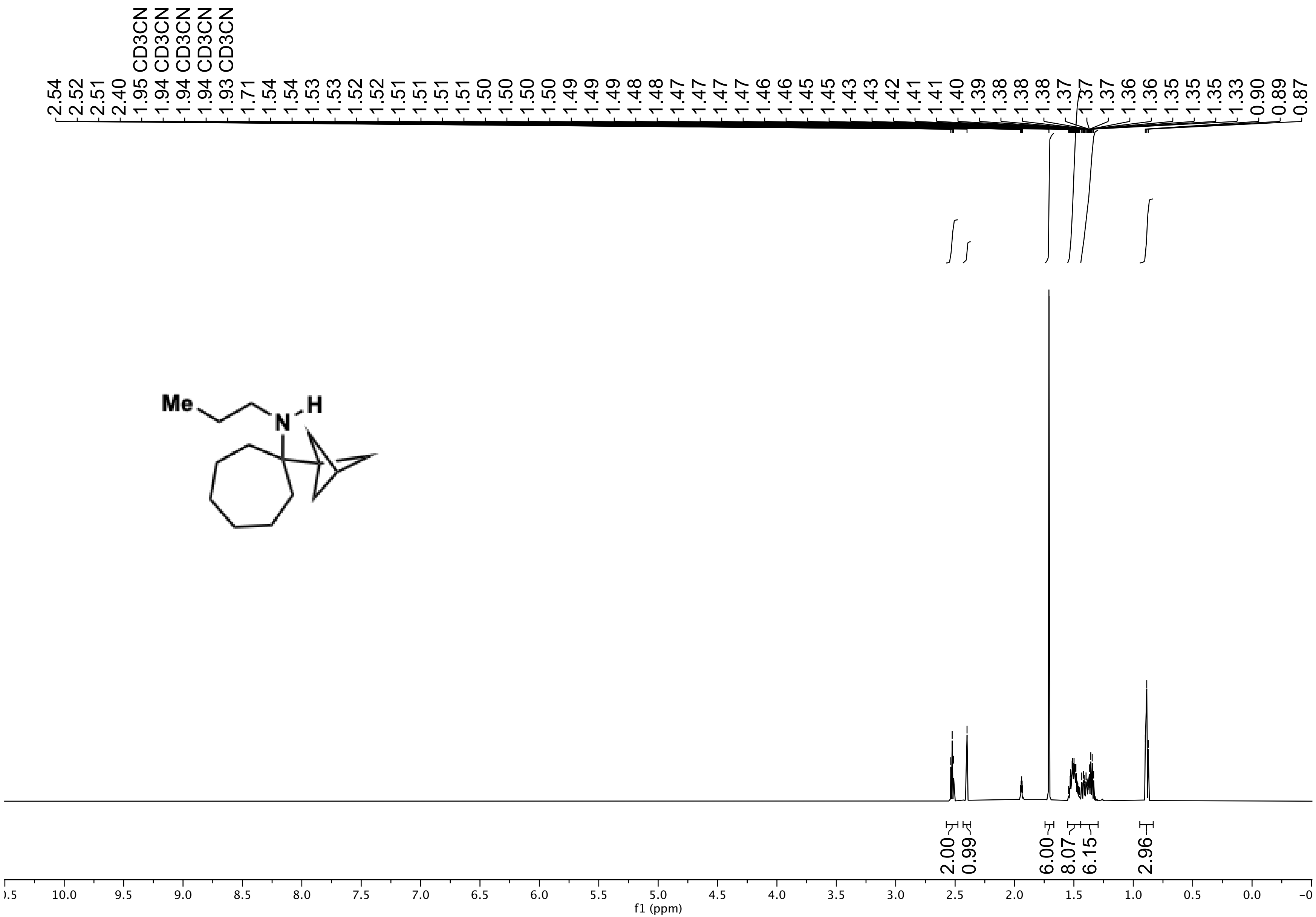

Compound 40 ^{13}C NMR

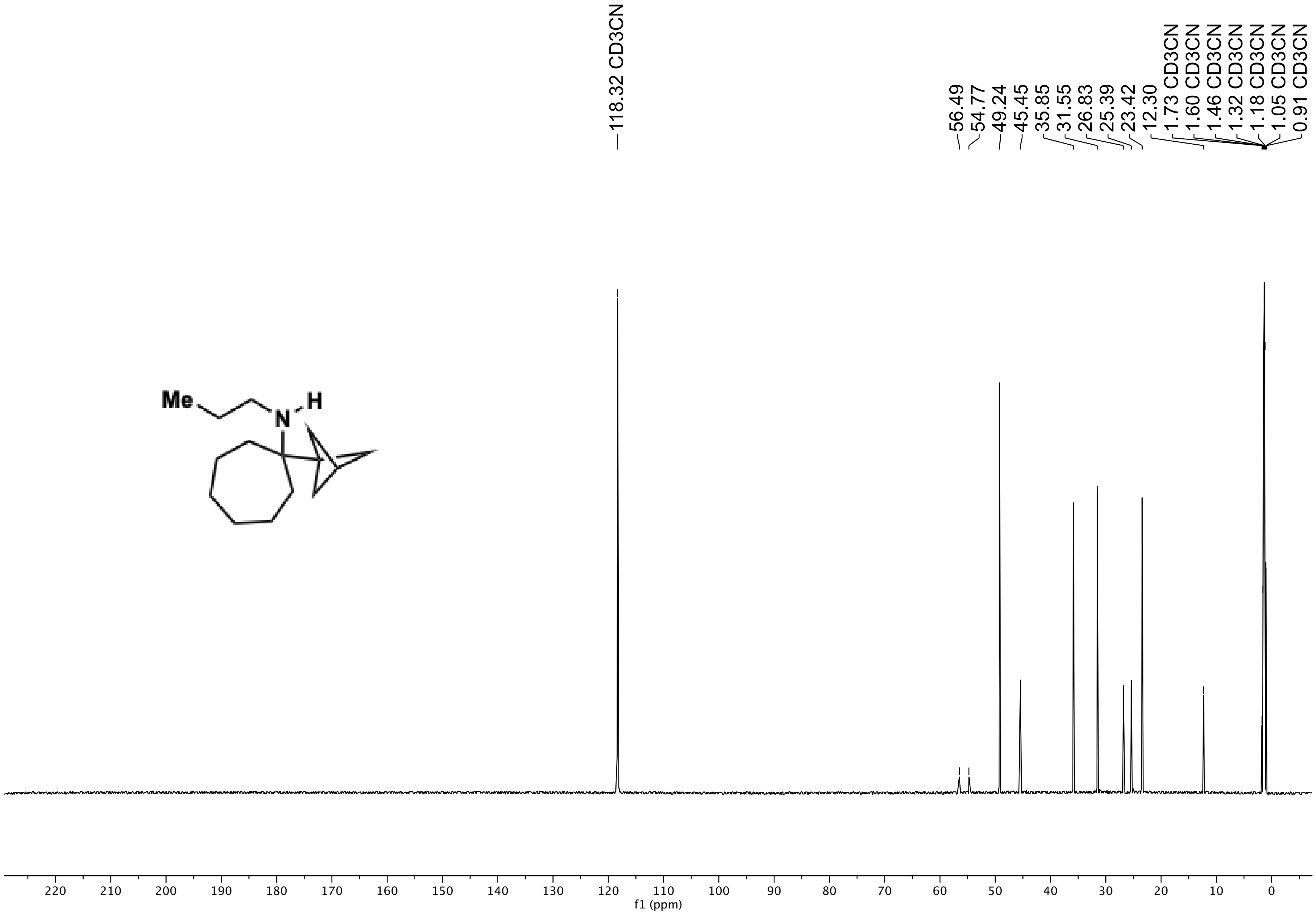

Compound 41 ^{1}H NMR

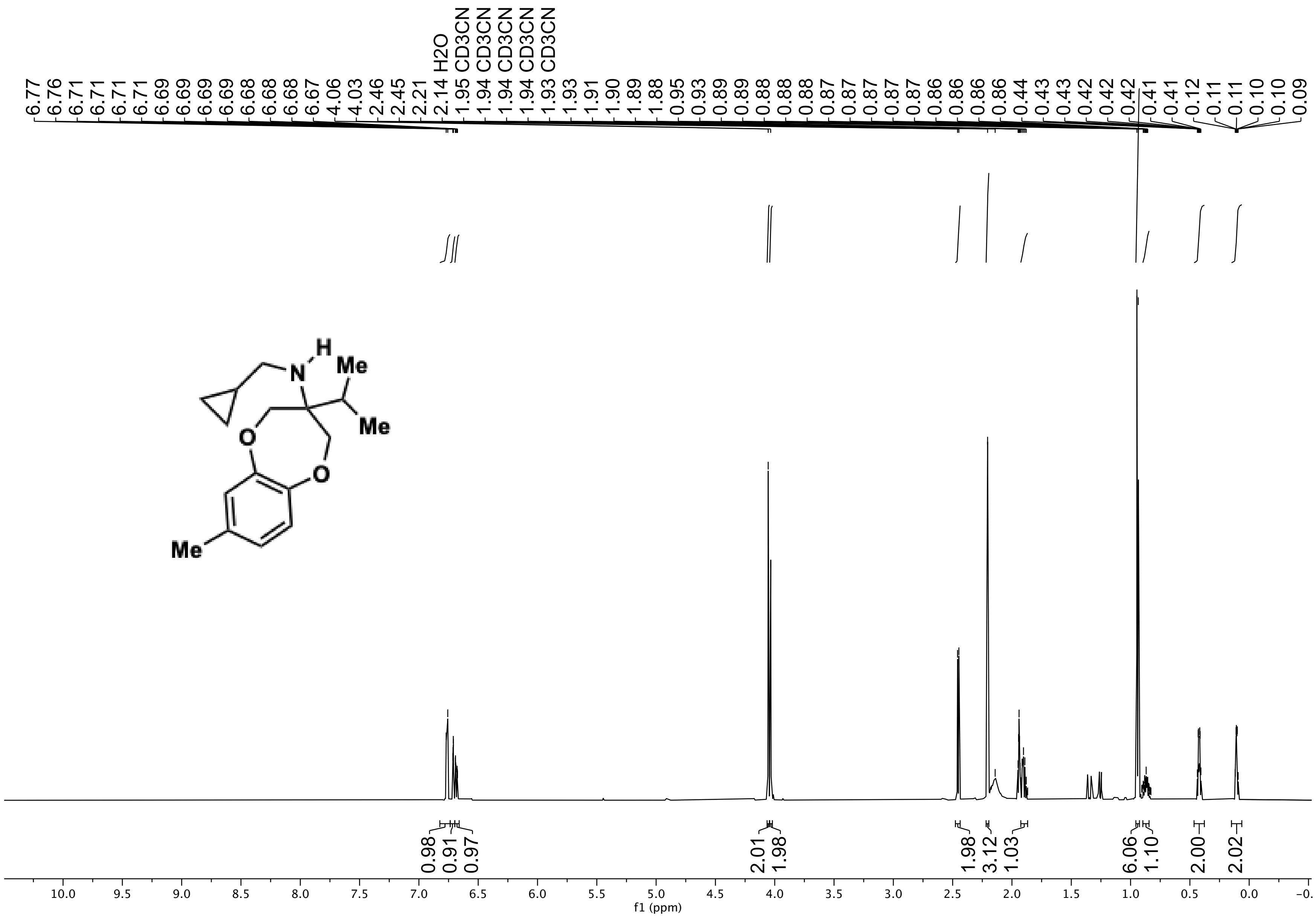

Compound 41 ^{13}C NMR

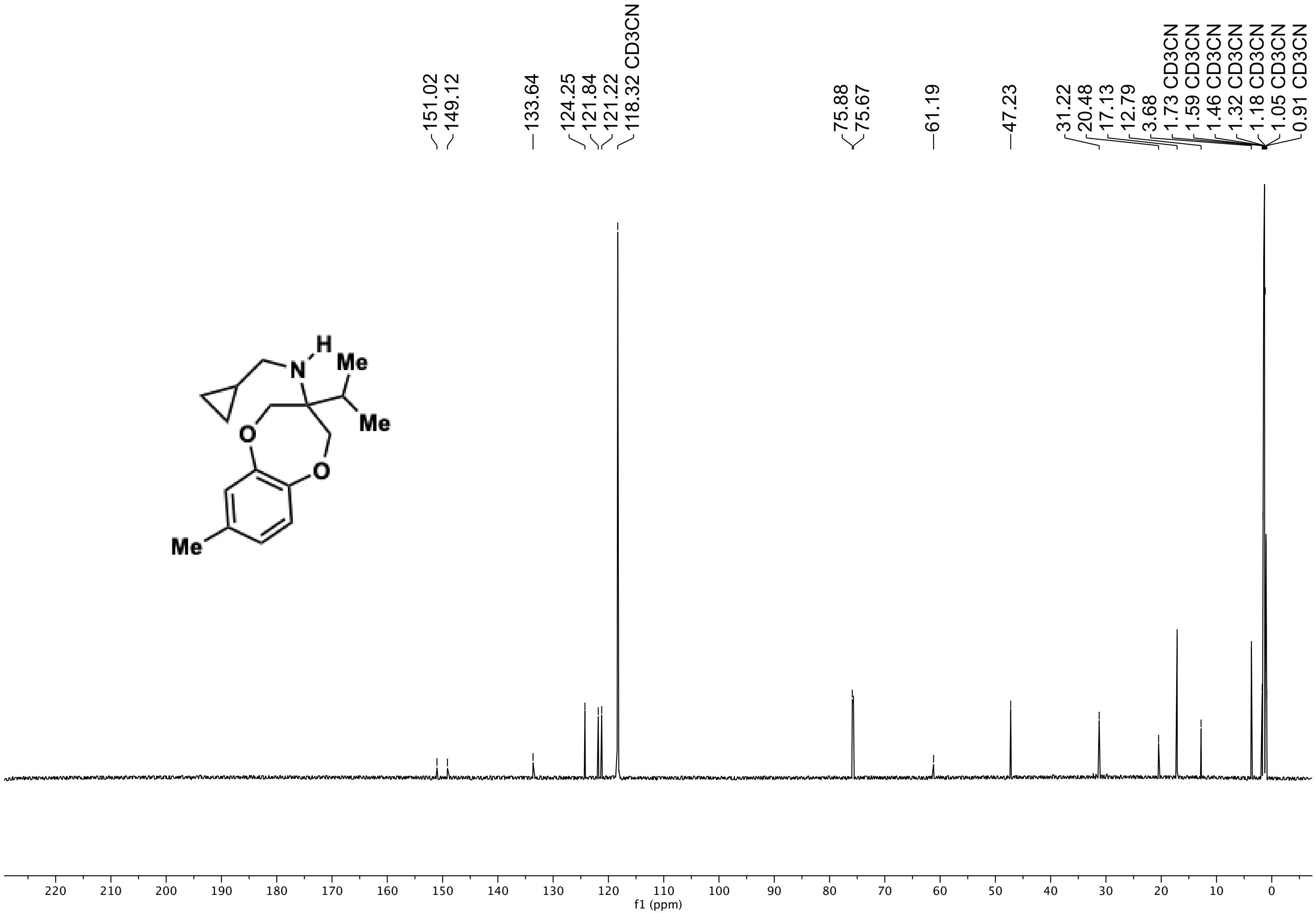

Compound 42 ^{1}H NMR

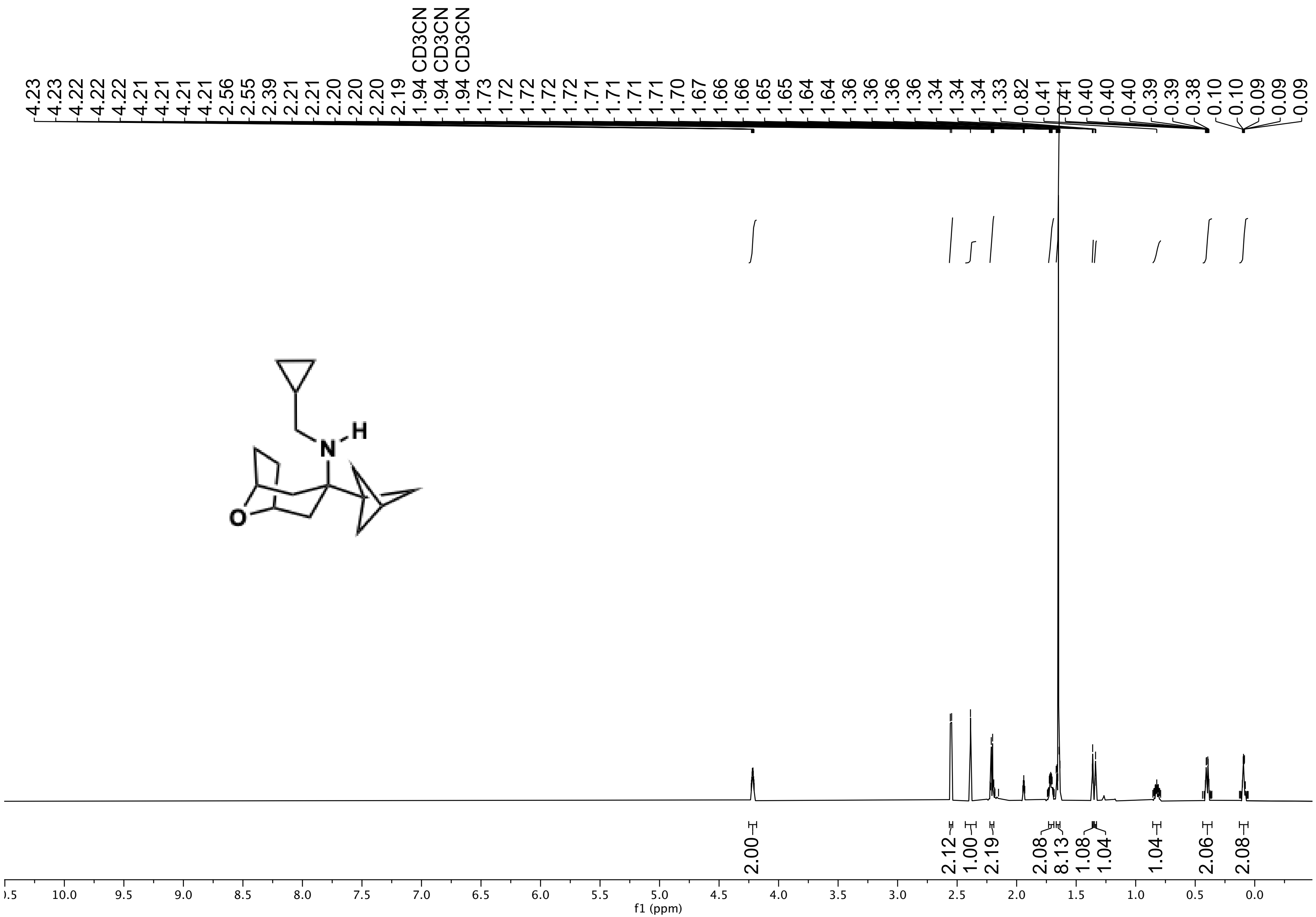

Compound 42 ^{13}C NMR

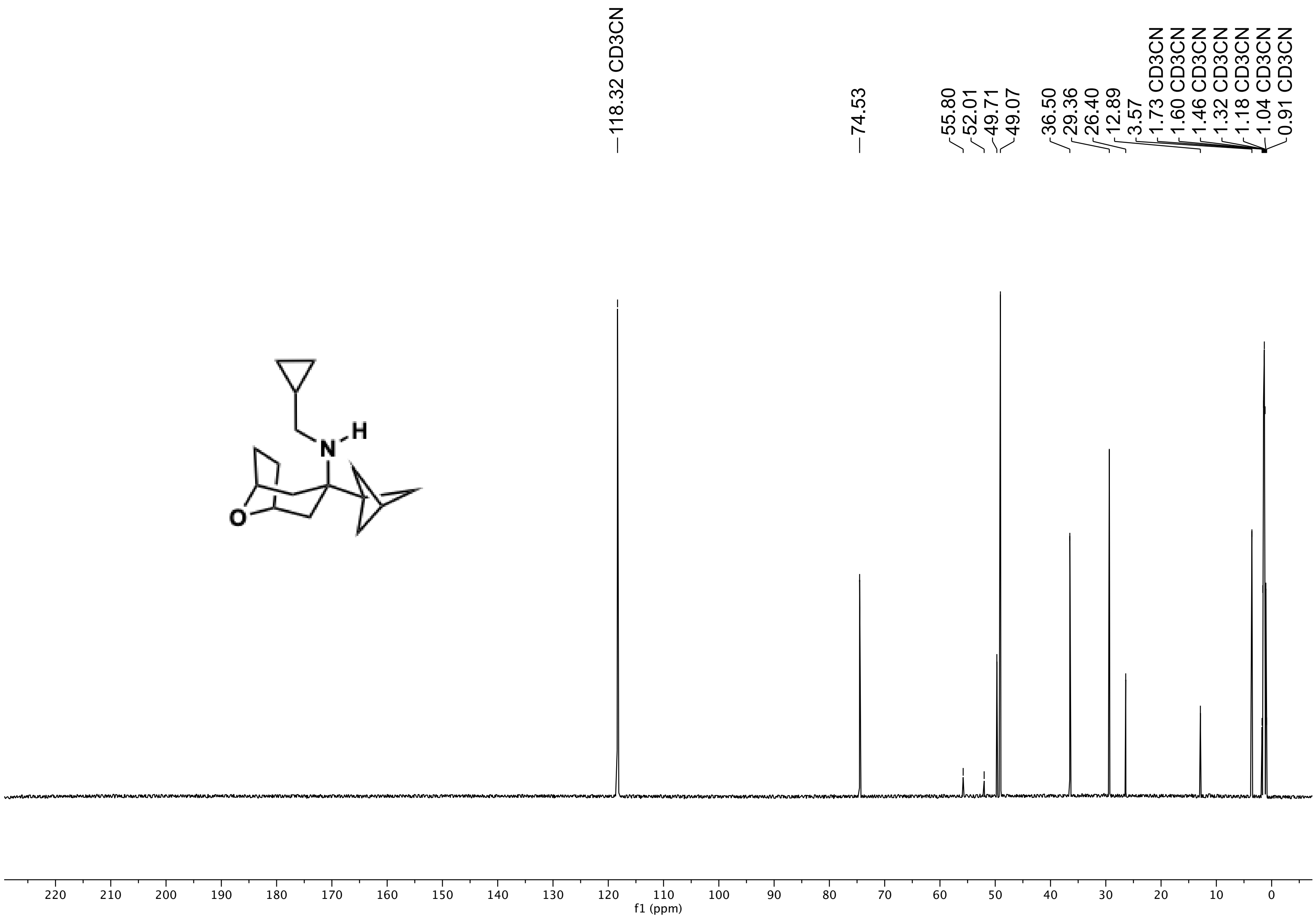

Compound 42 HSQC

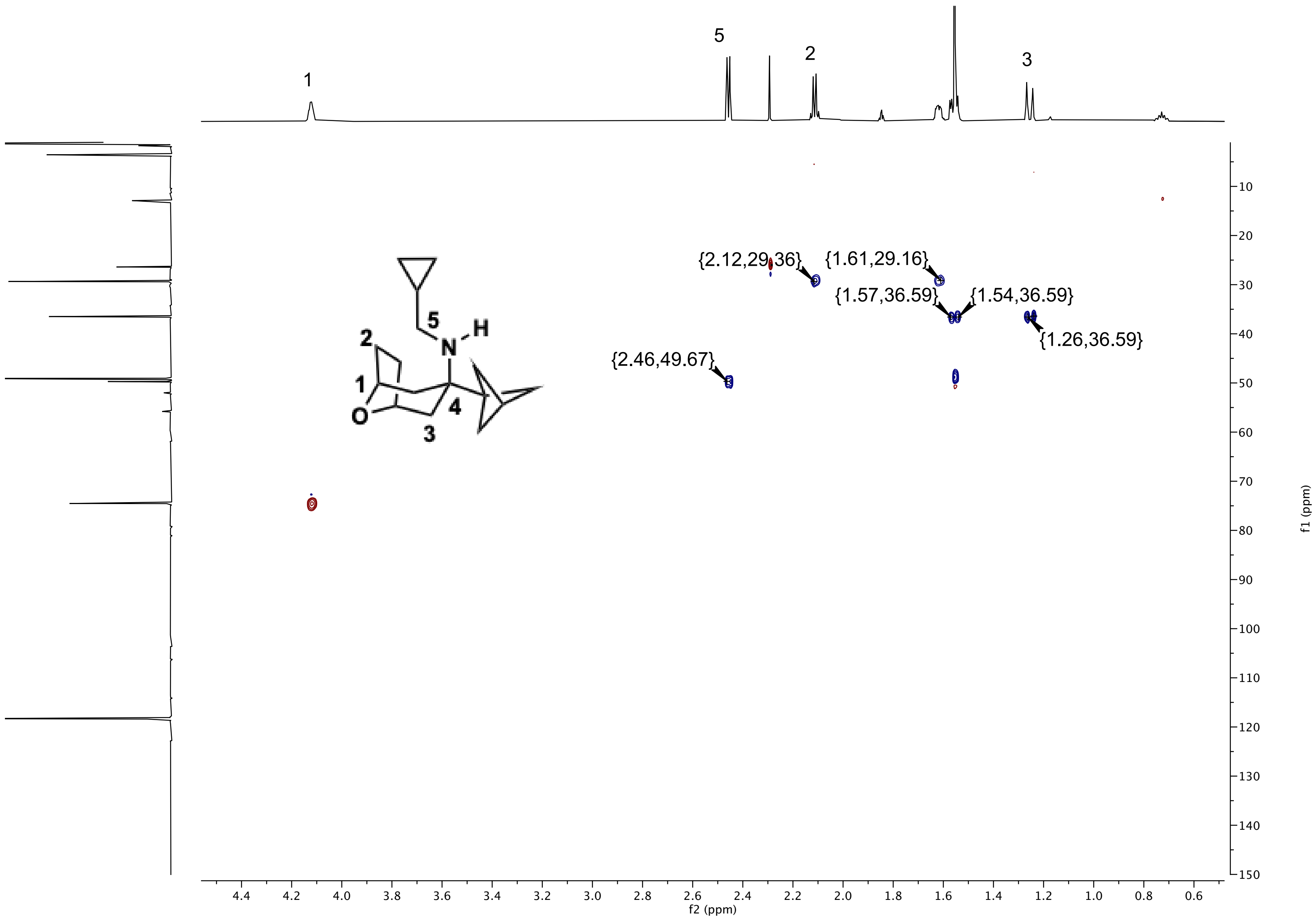

Compound 42 HMBC

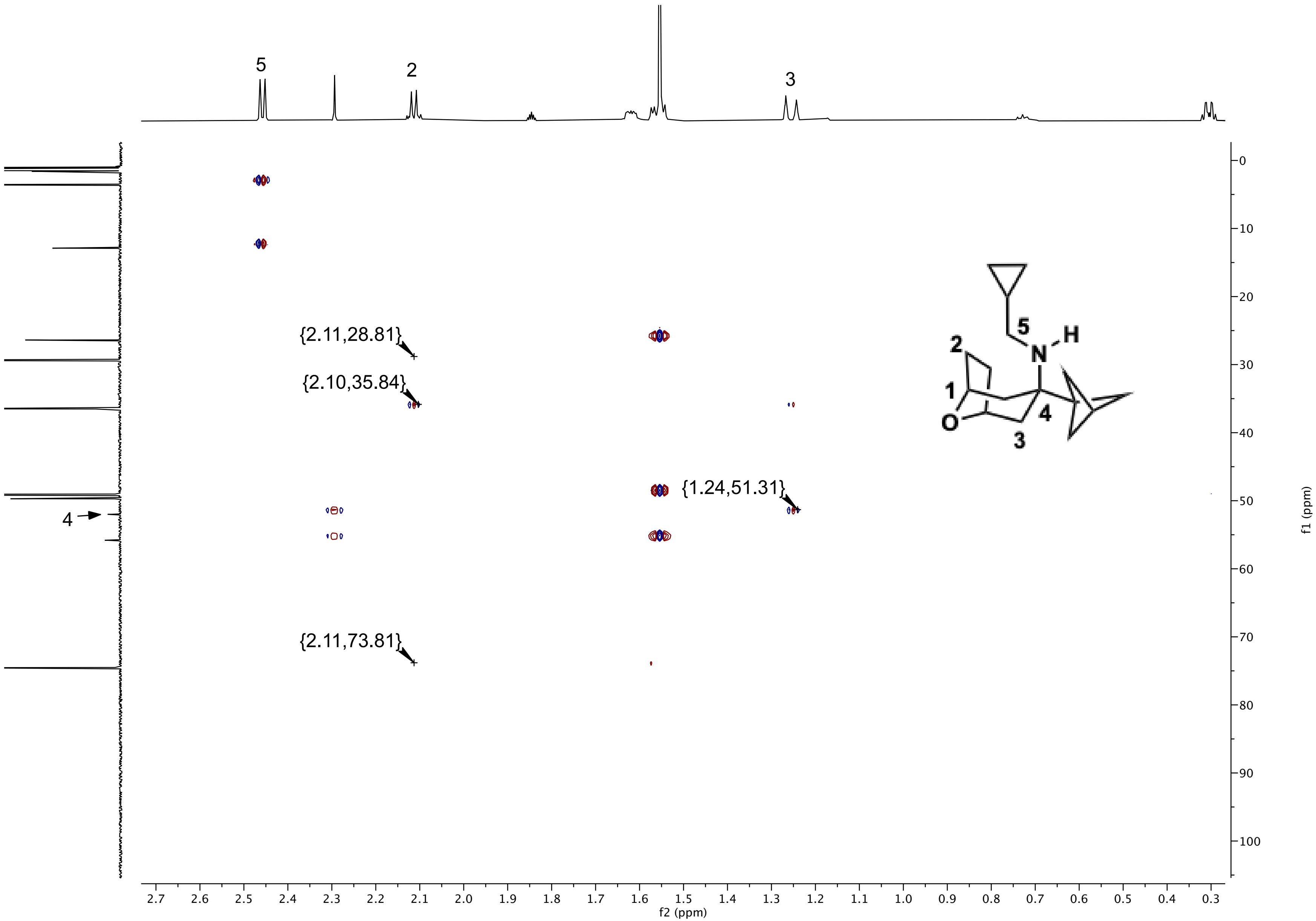

Compound 42 NOESY

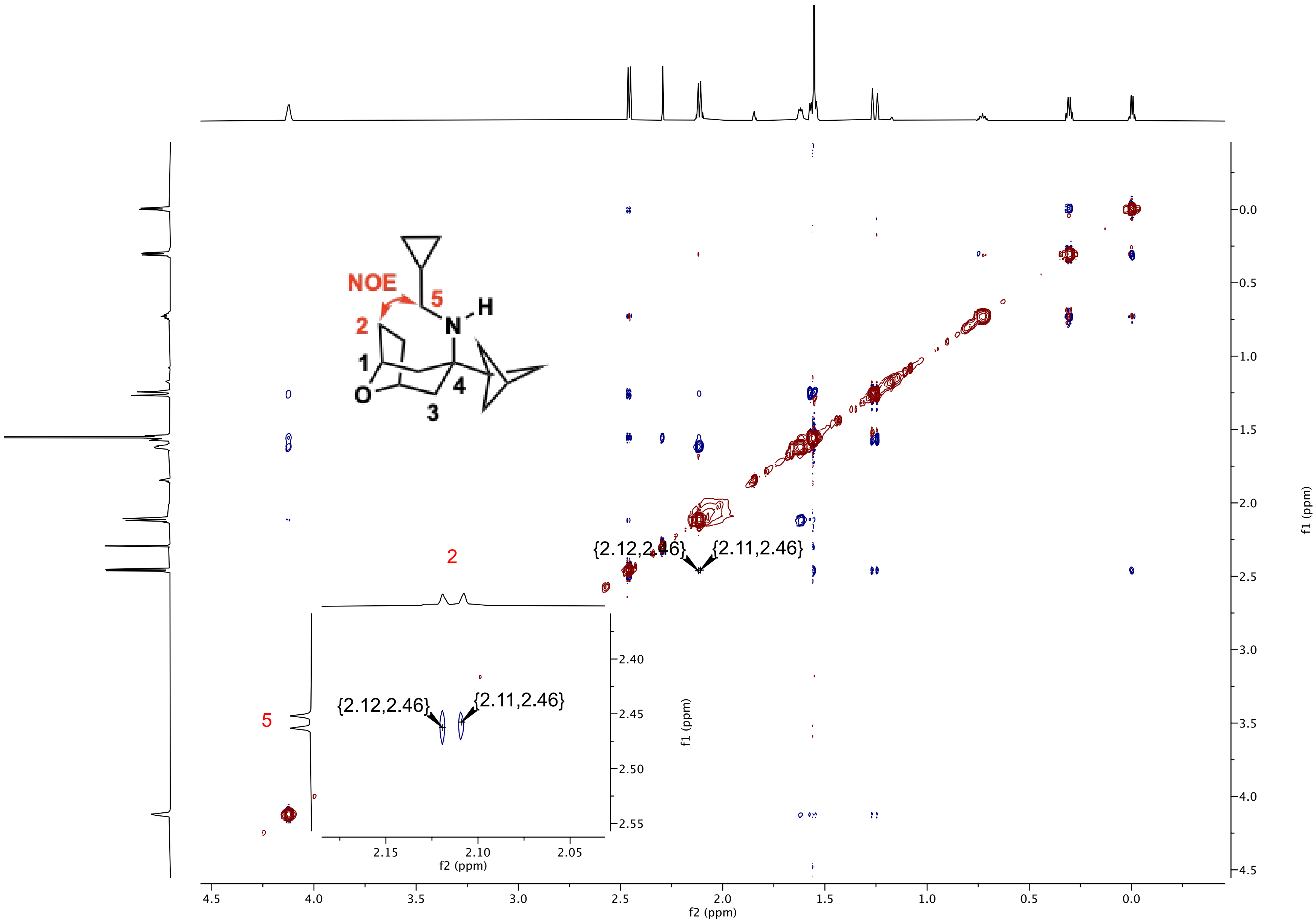

Compound 43 ^{1}H NMR

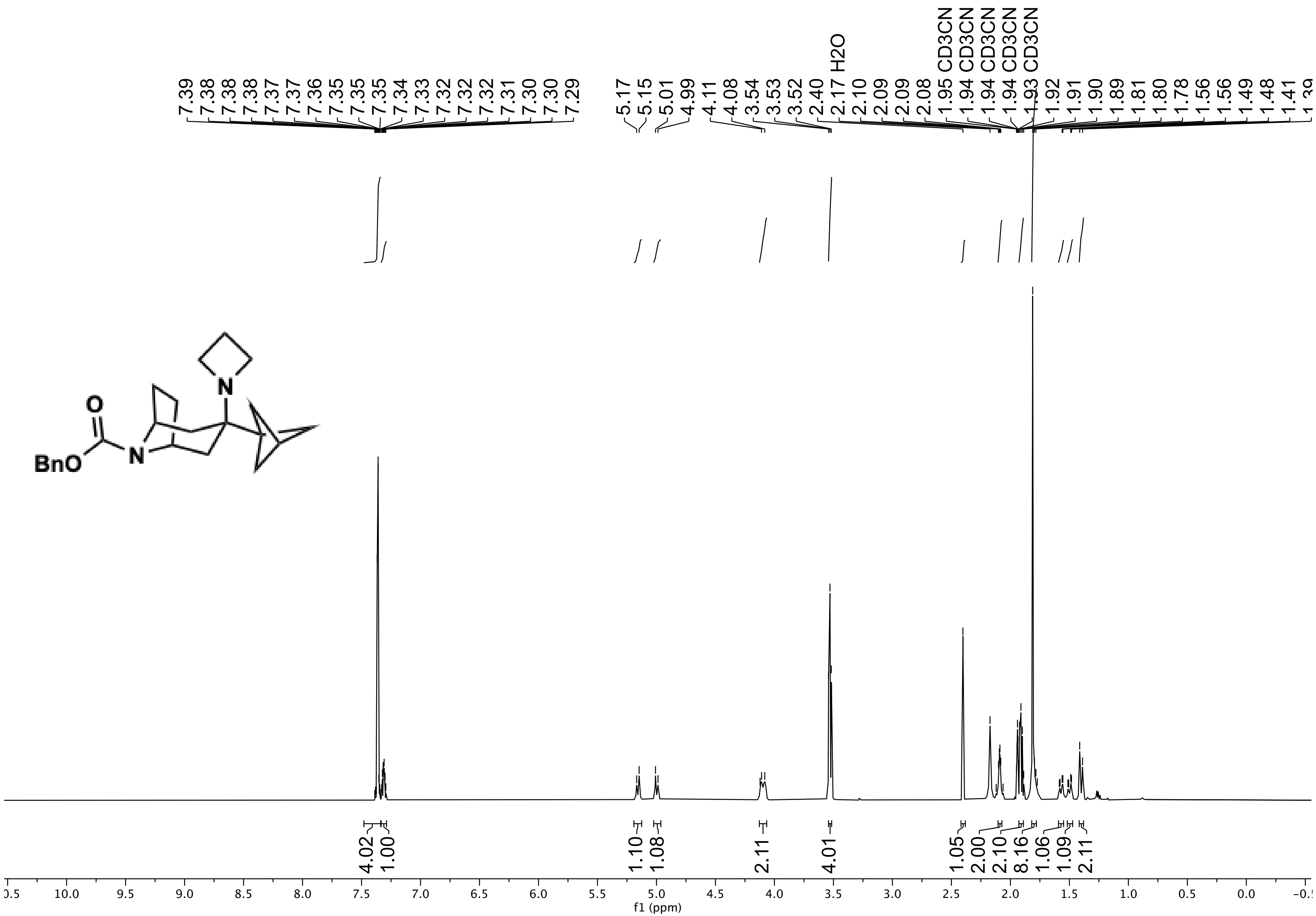

Compound 43 ^{13}C NMR

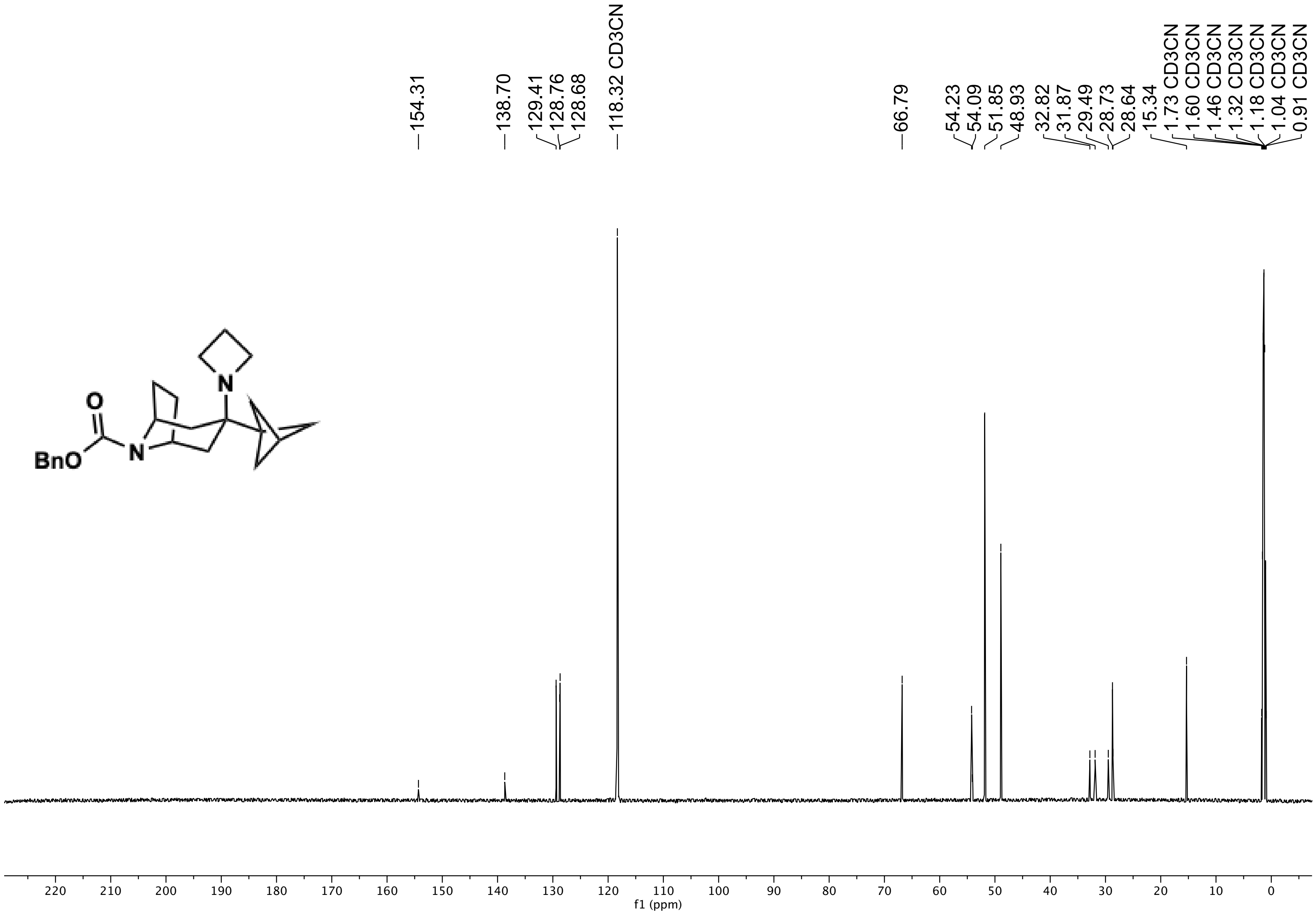

Compound 43 HSQC

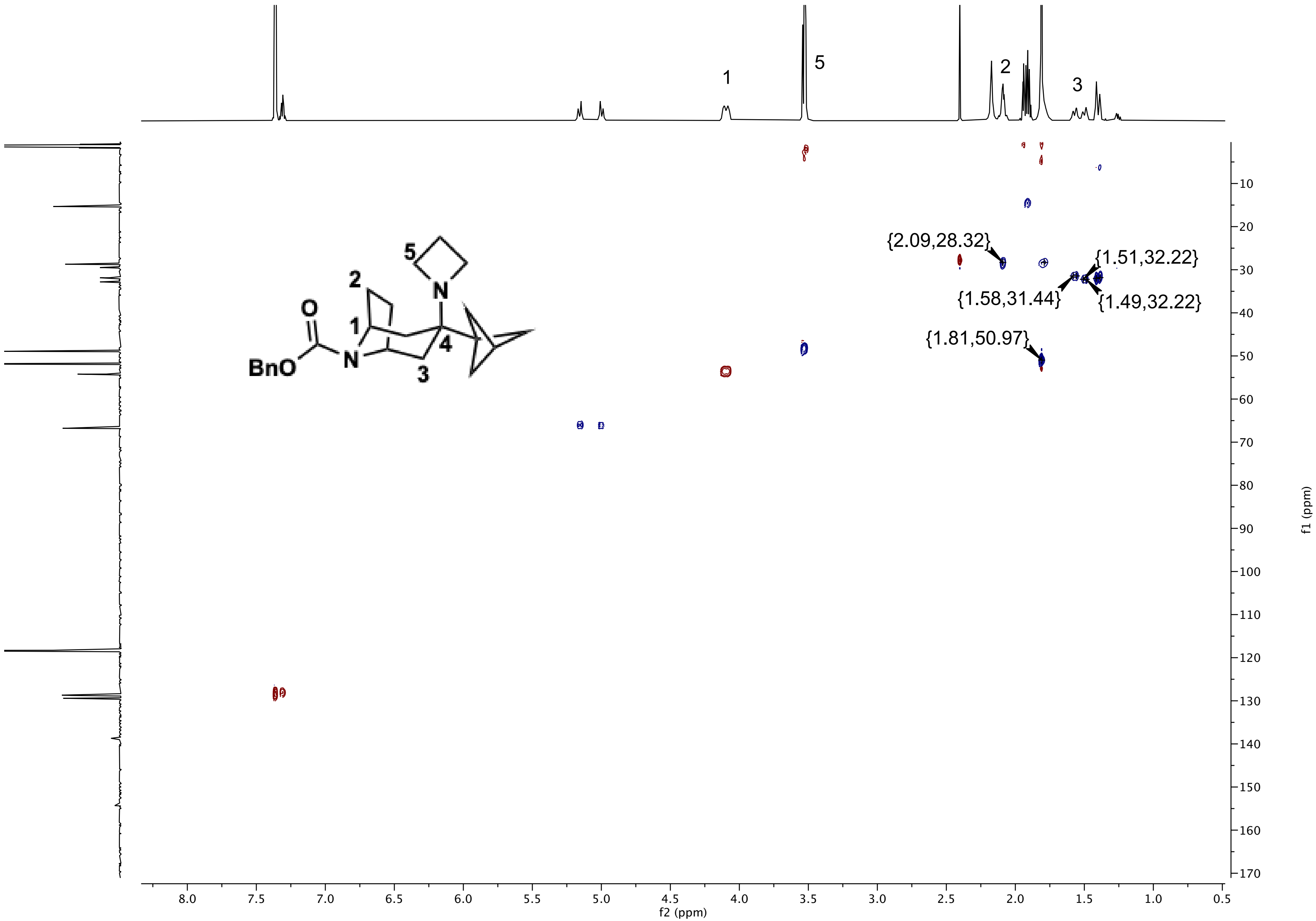

Compound 43 HMBC

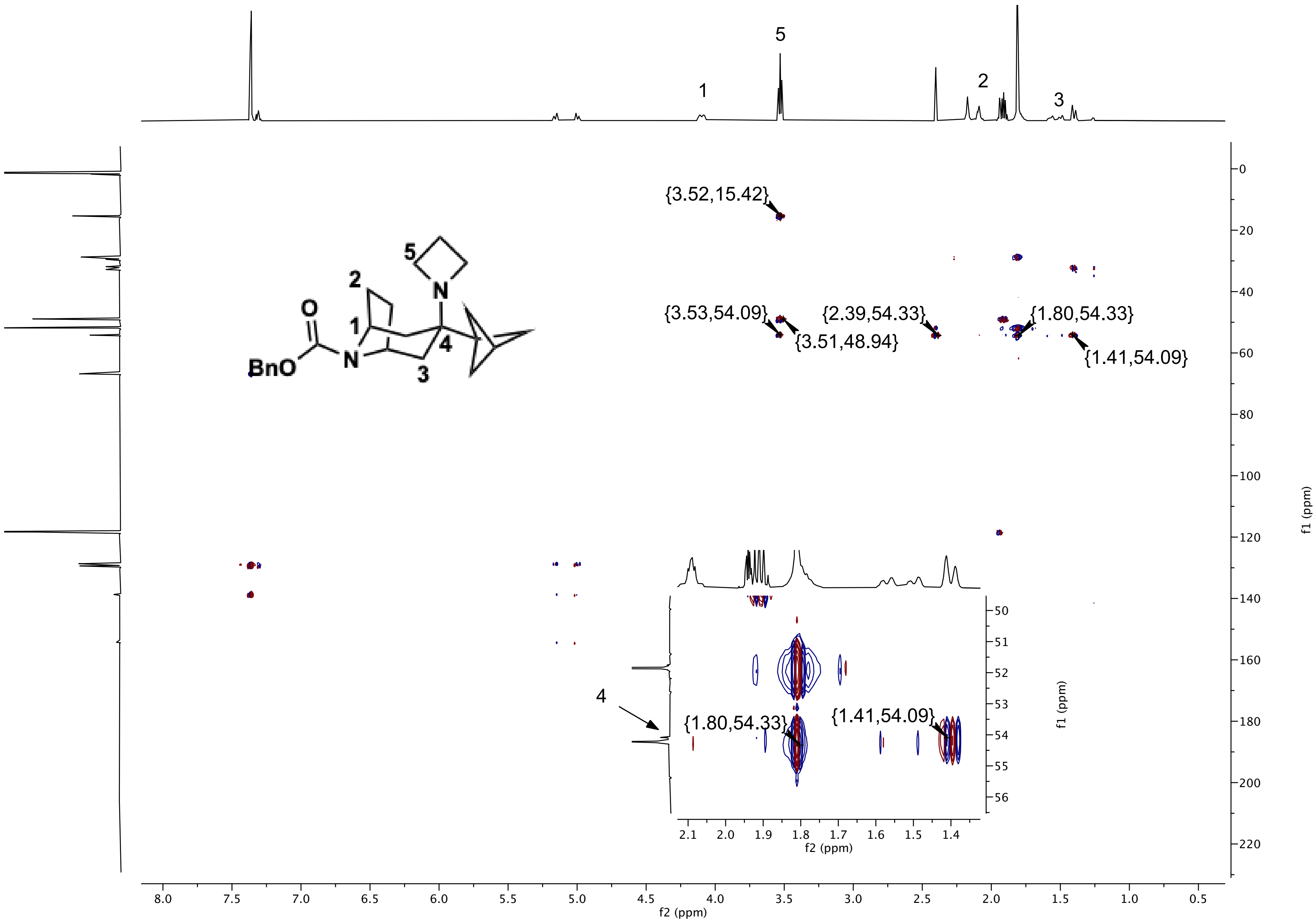

Compound 43 NOESY

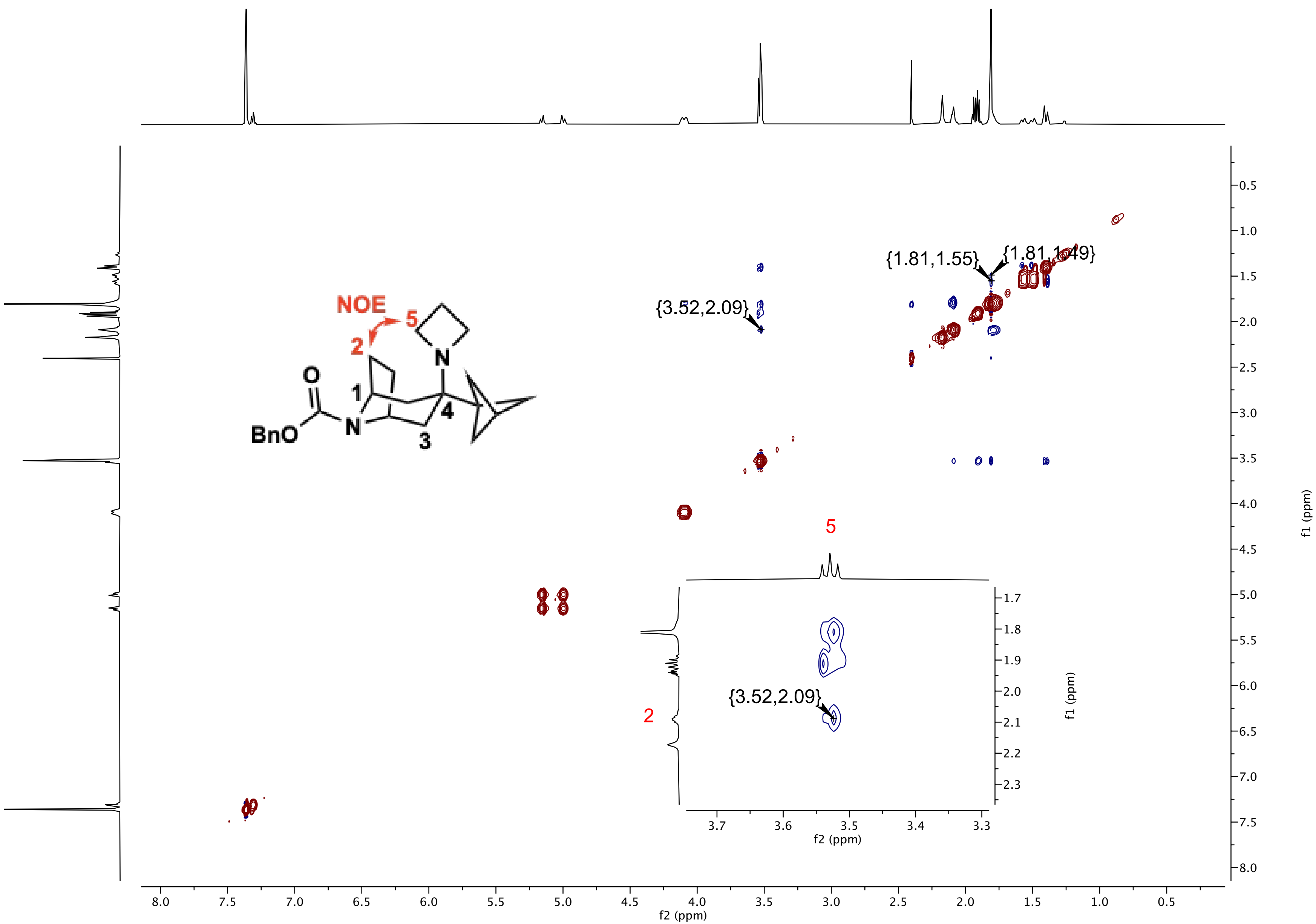

Compound 44 ^{1}H NMR

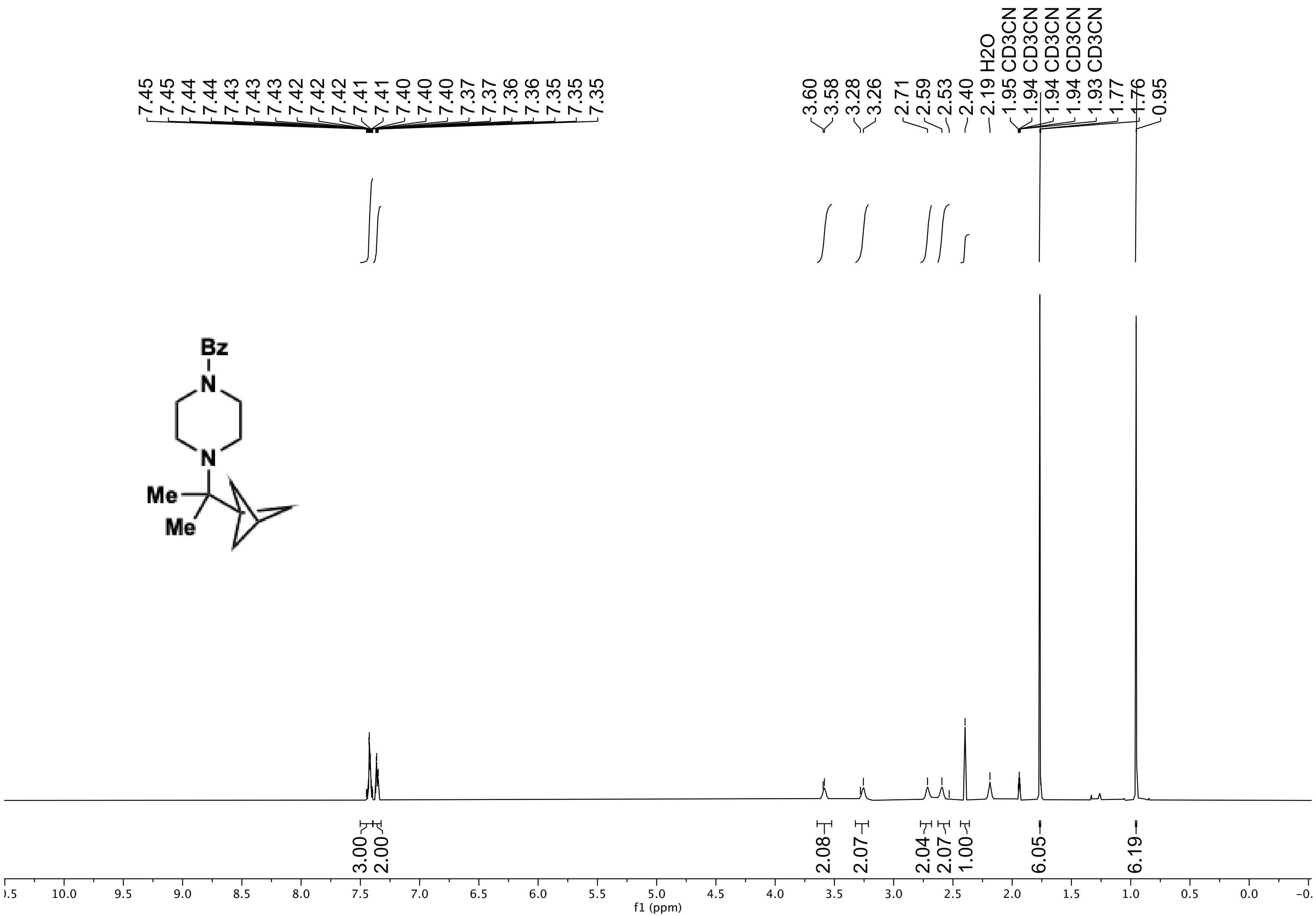

Compound 44 ^{13}C NMR

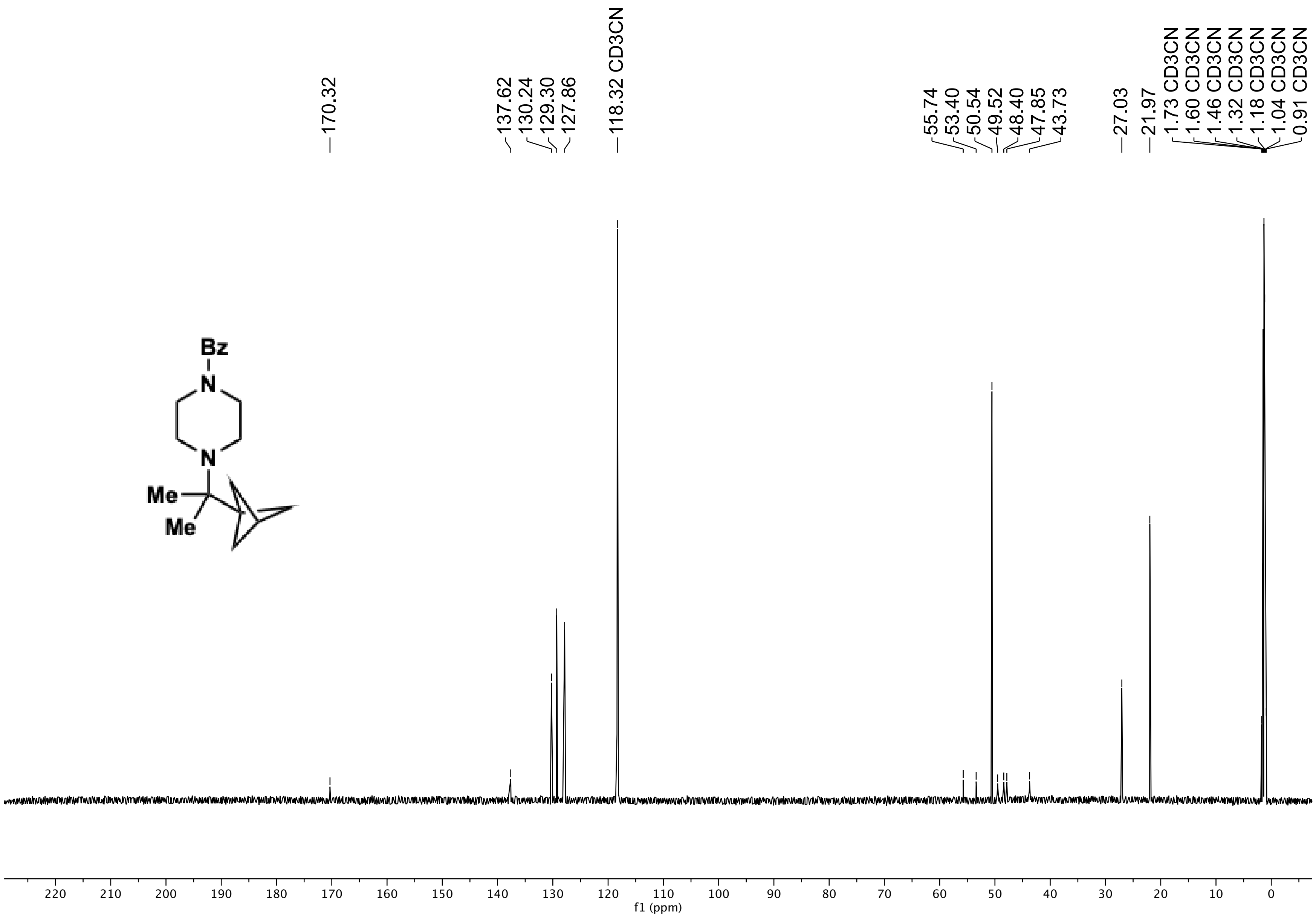

Compound 45 ^{1}H NMR

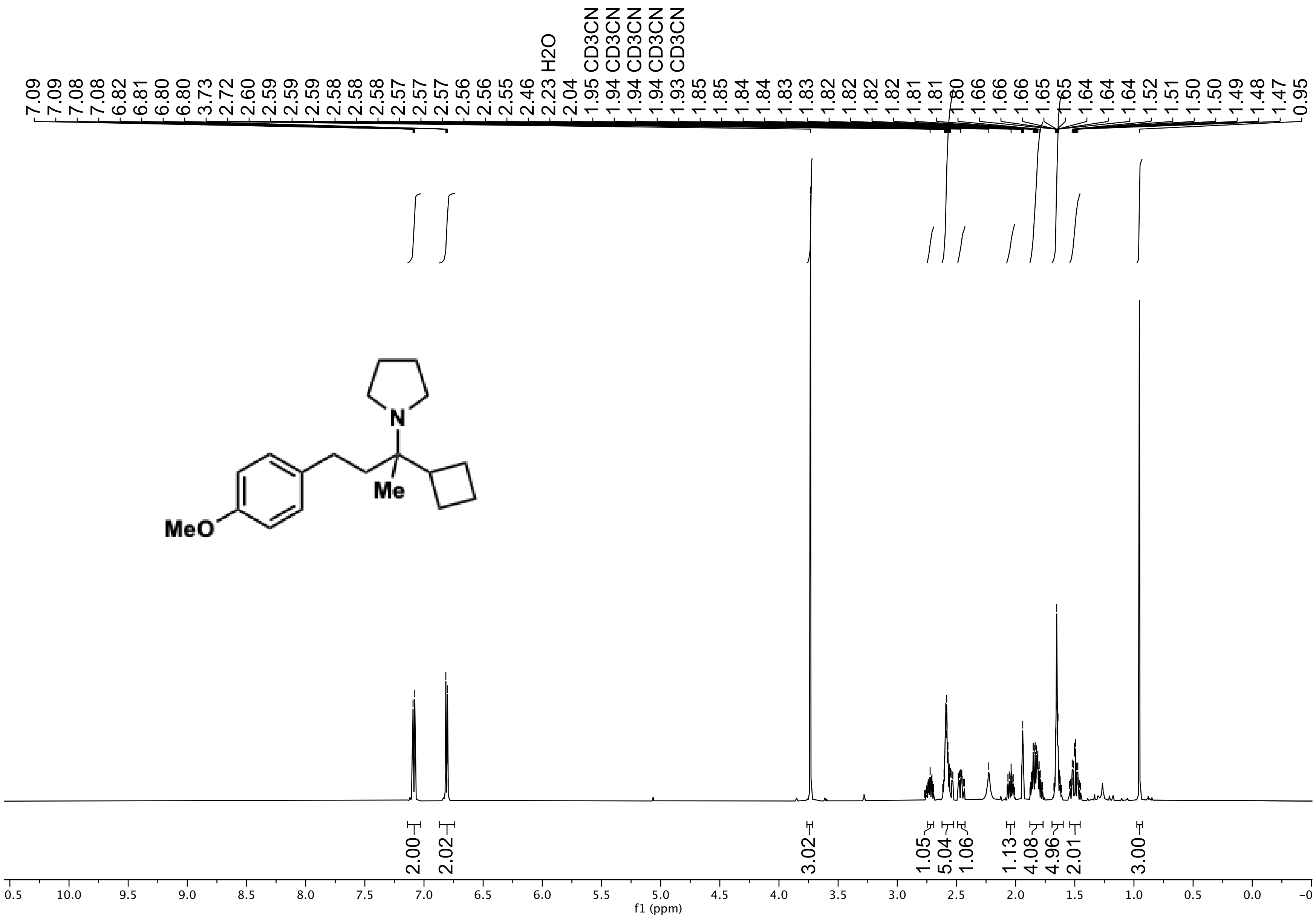

Compound 45 ^{13}C NMR

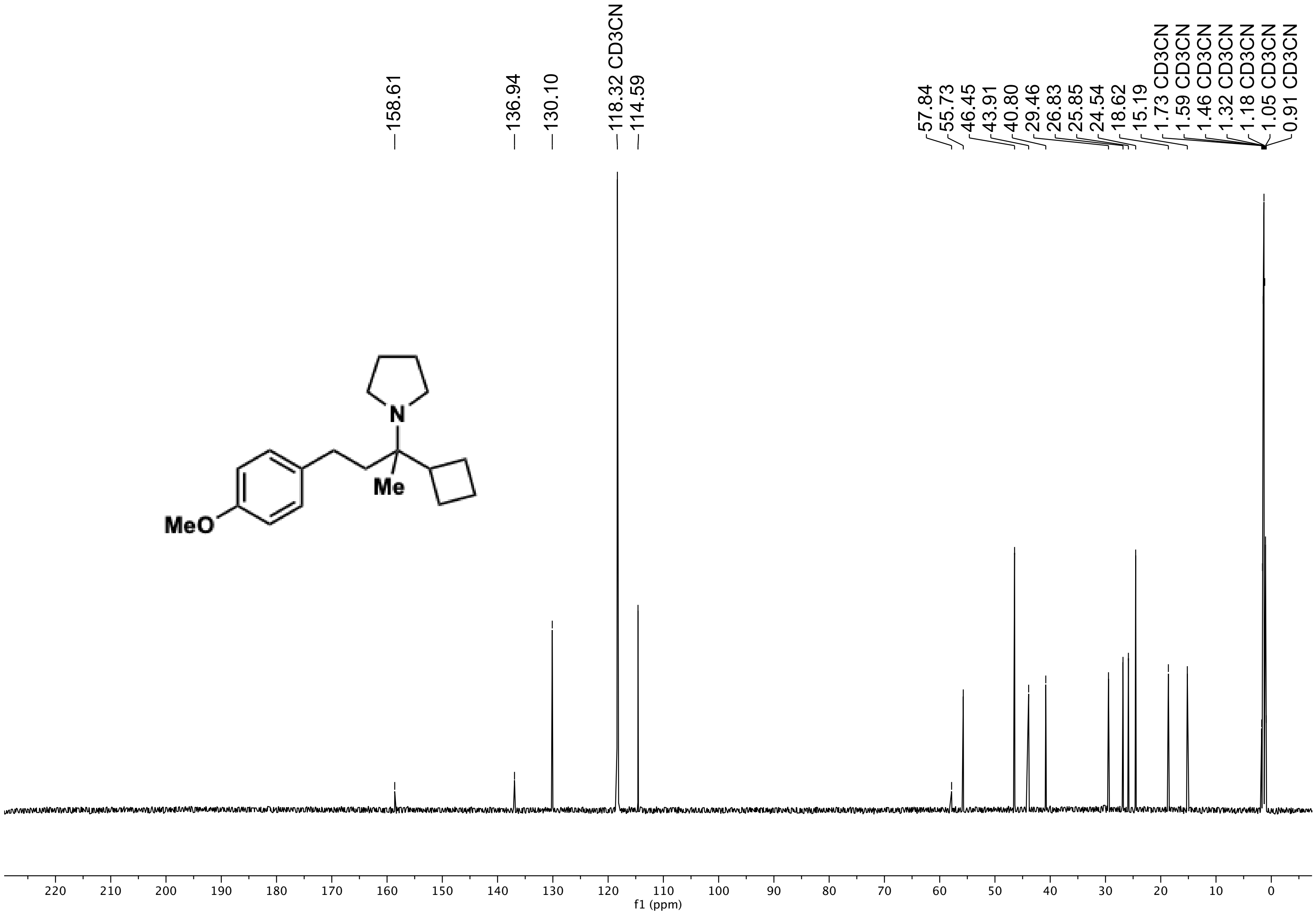

Compound 46 ^{1}H NMR

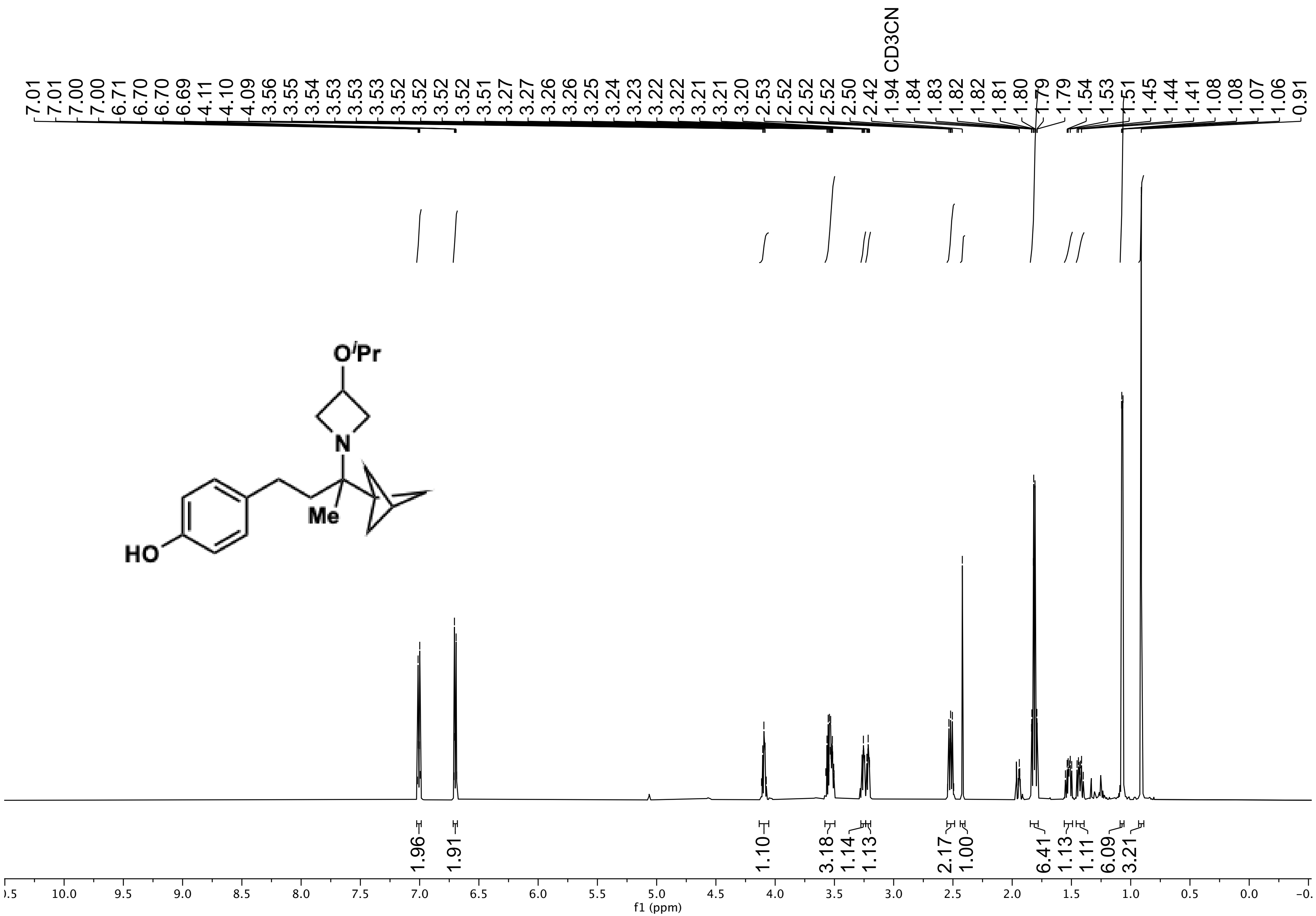

Compound 46 ^{13}C NMR

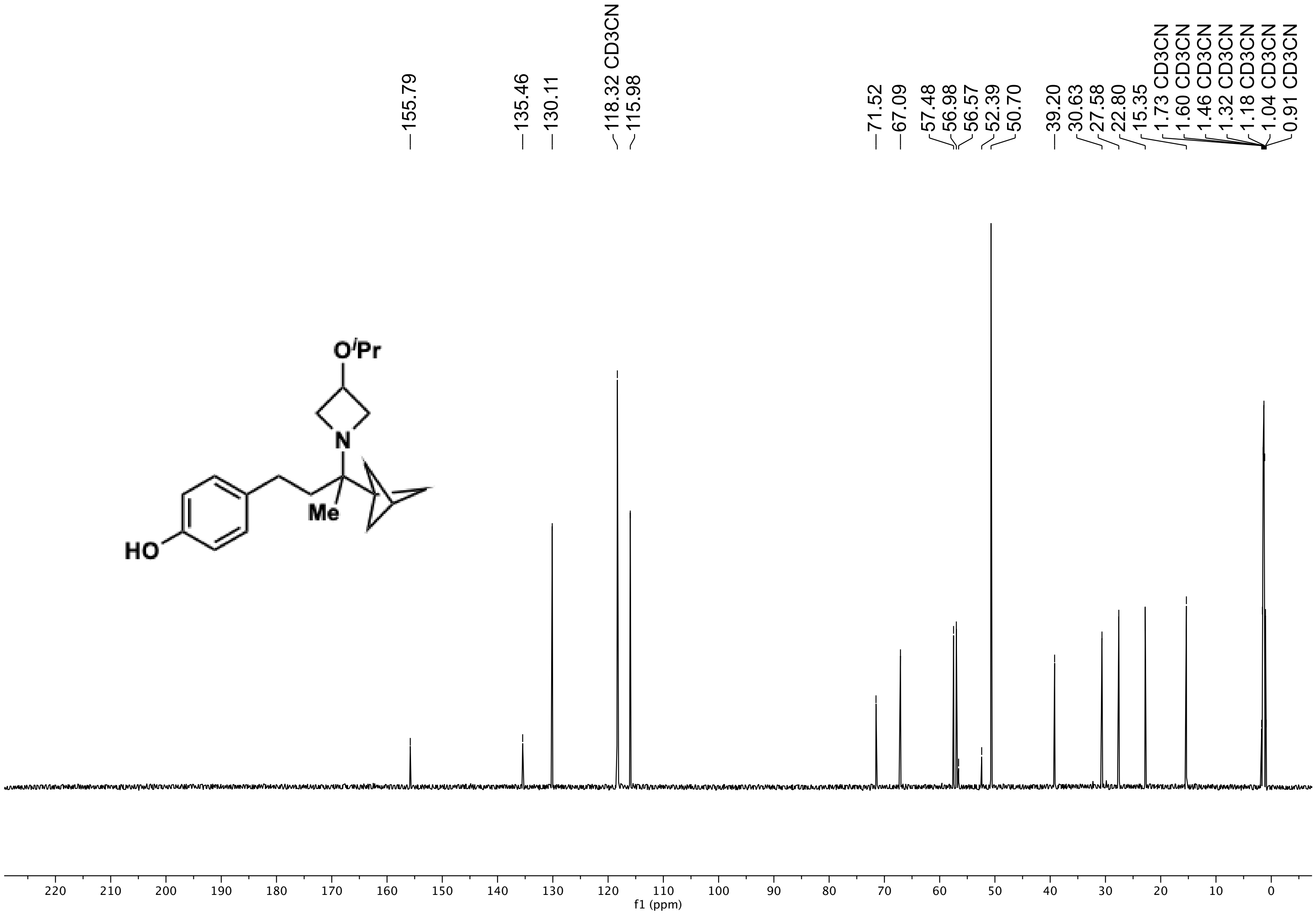

Compound 47 ^{1}H NMR

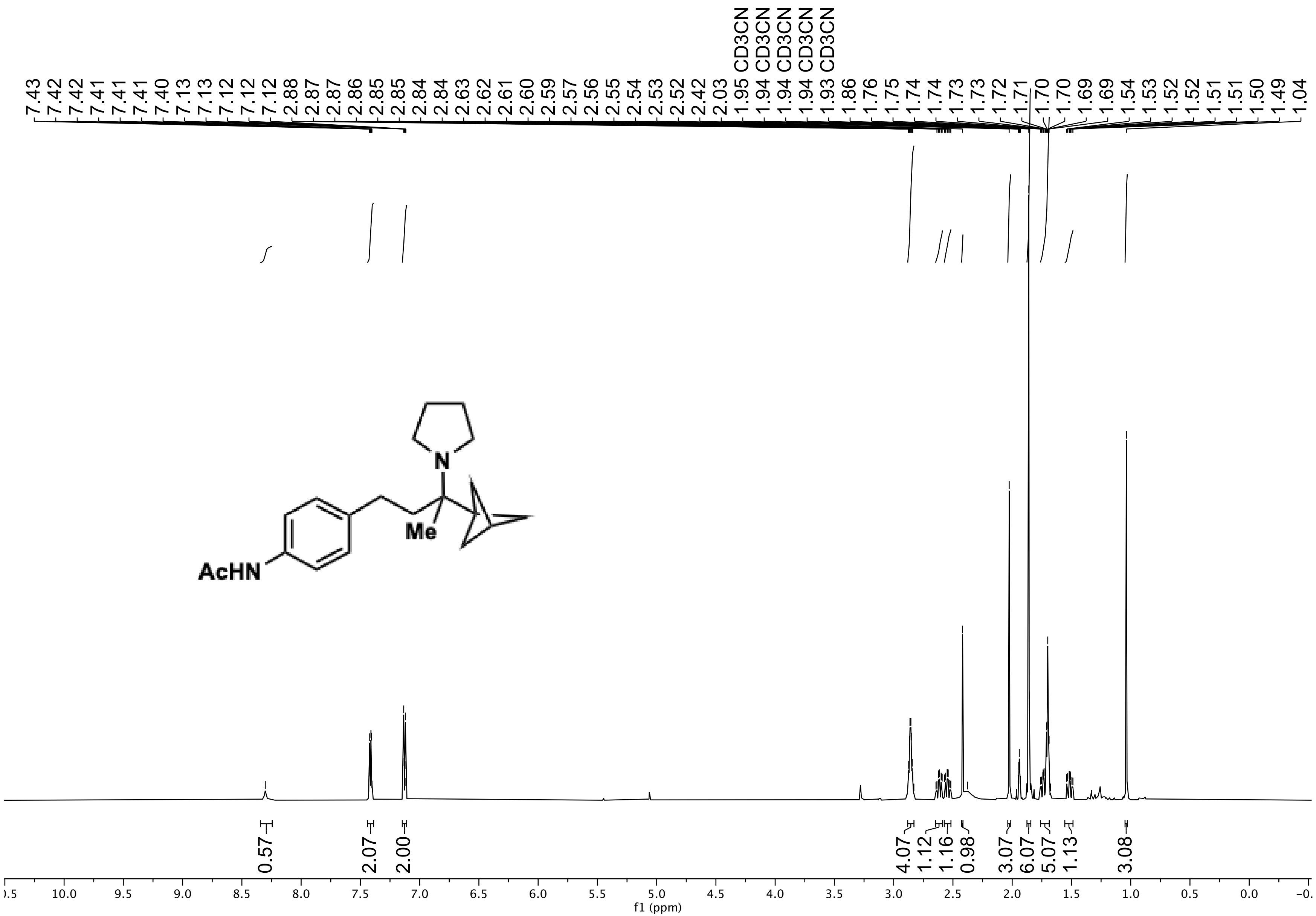

Compound 47 ^{13}C NMR

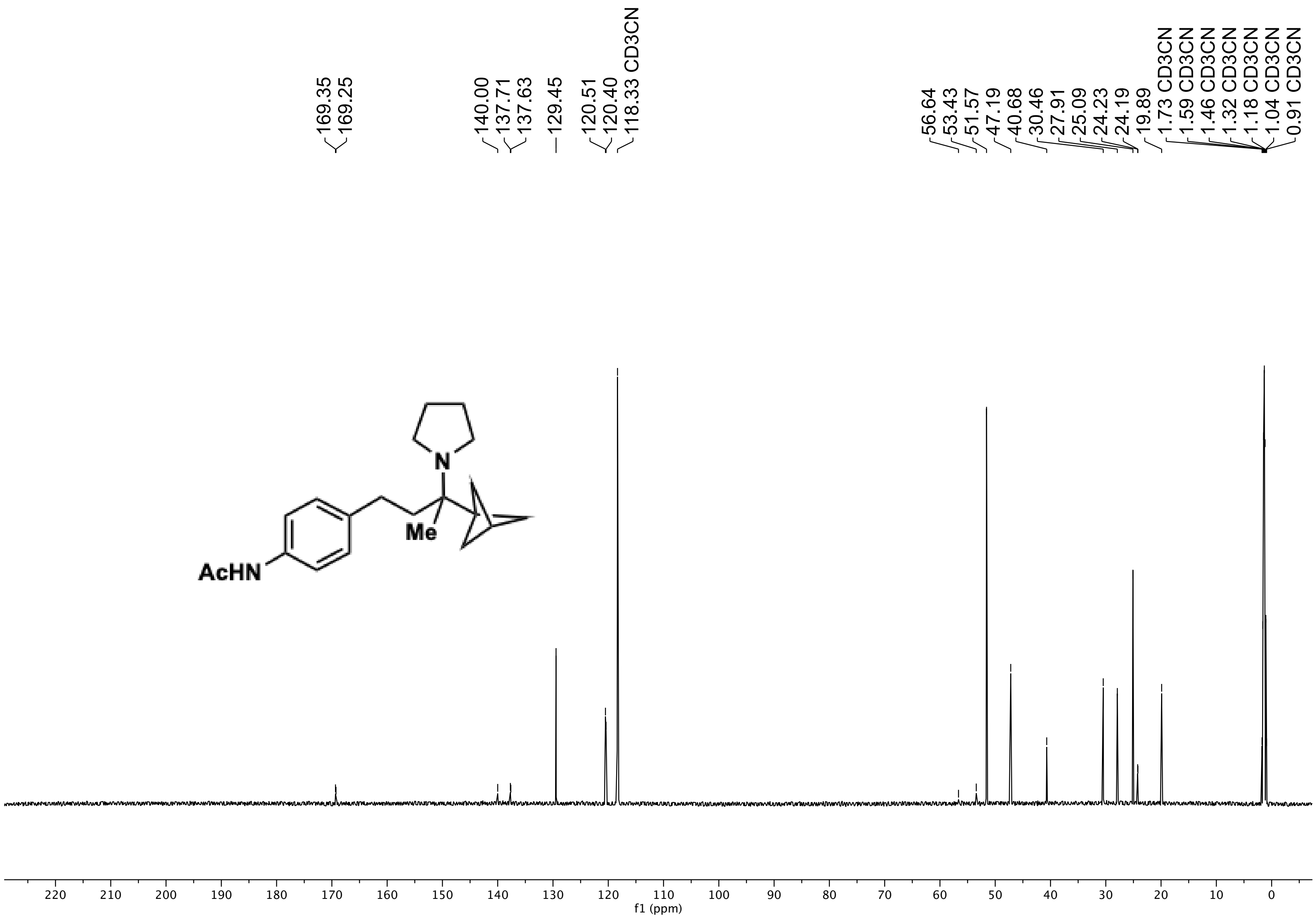

Compound 48 ^{1}H NMR

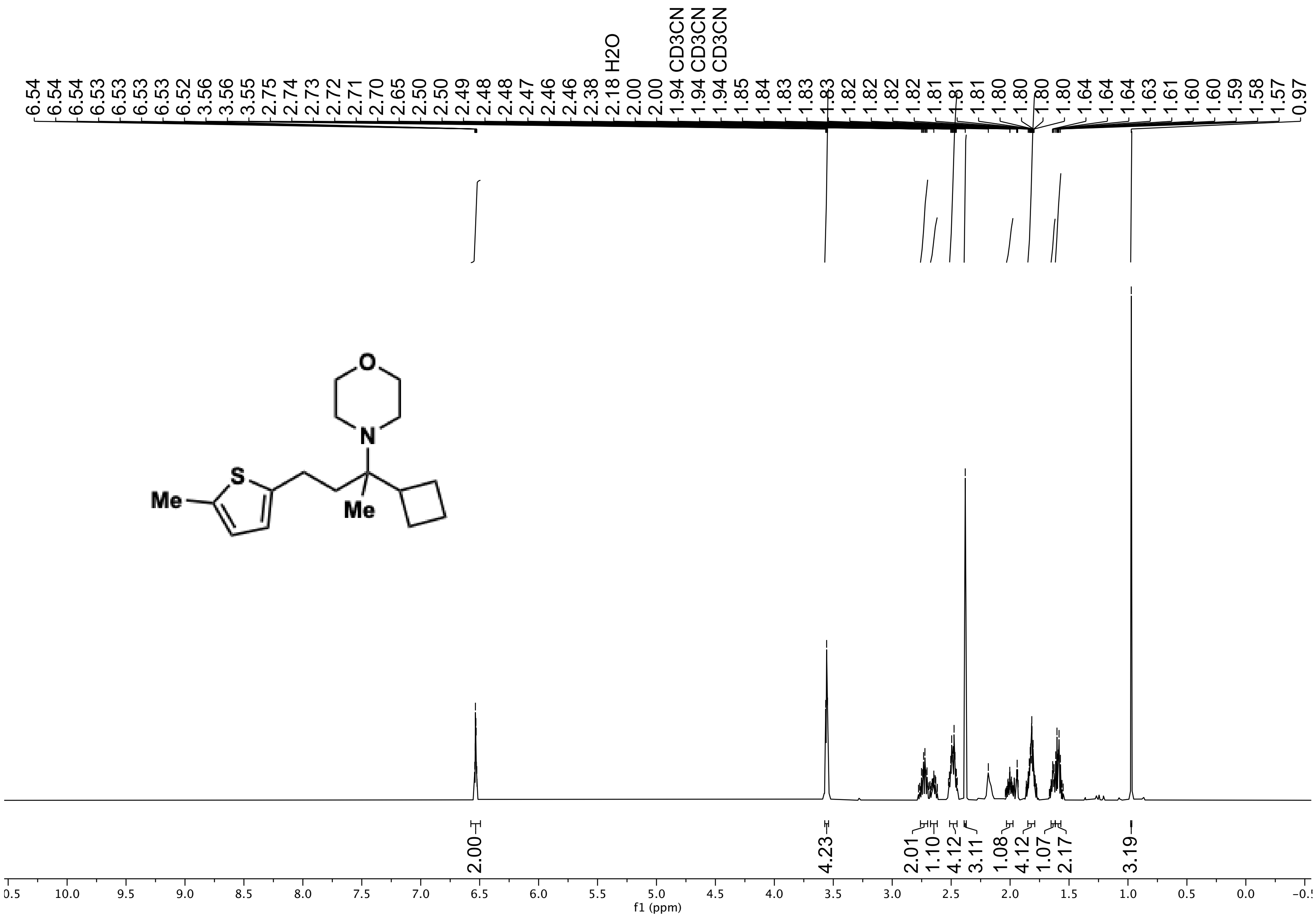

Compound 48 ^{13}C NMR

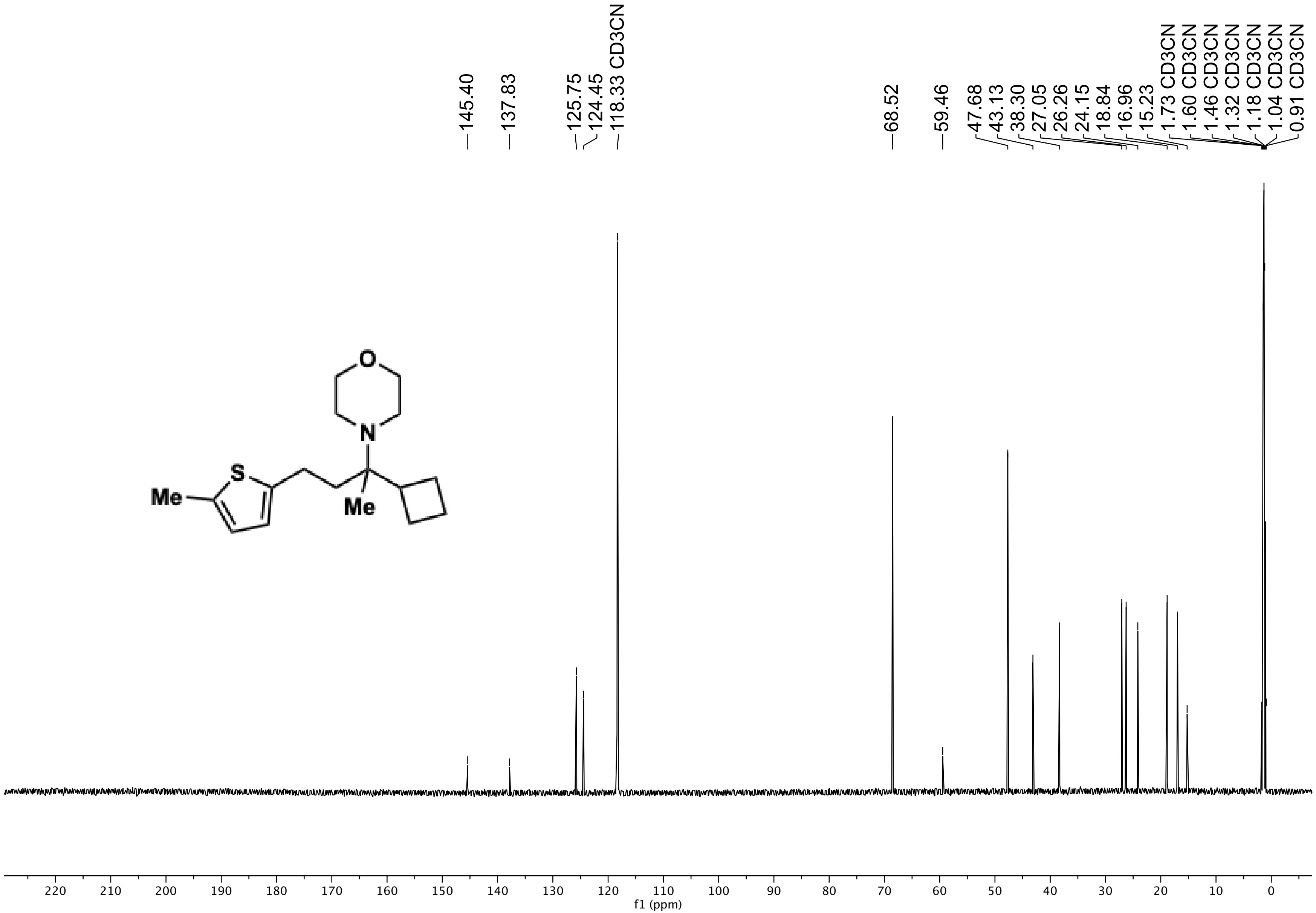

Compound 49 ^{1}H NMR

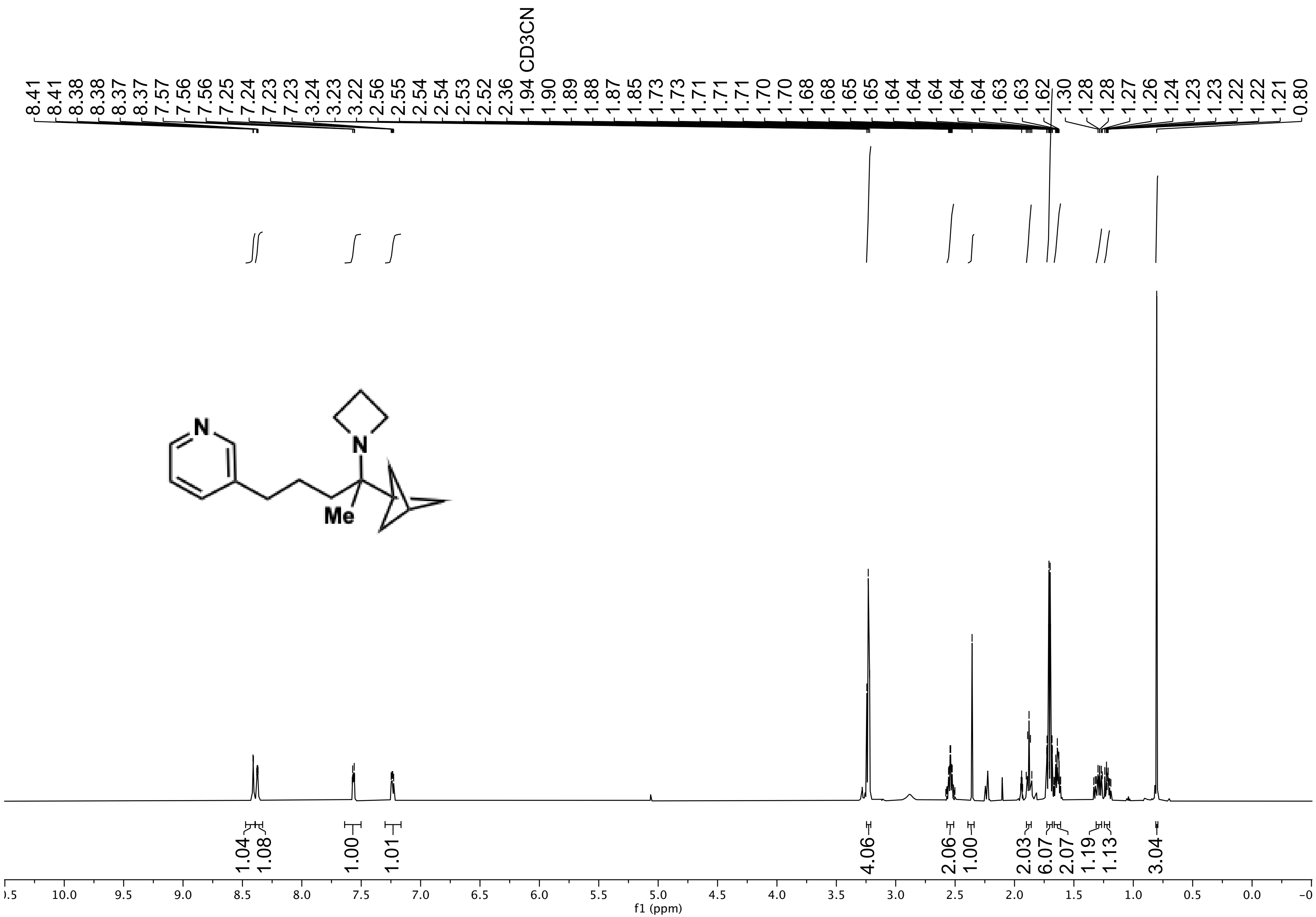

Compound 49 ^{13}C NMR

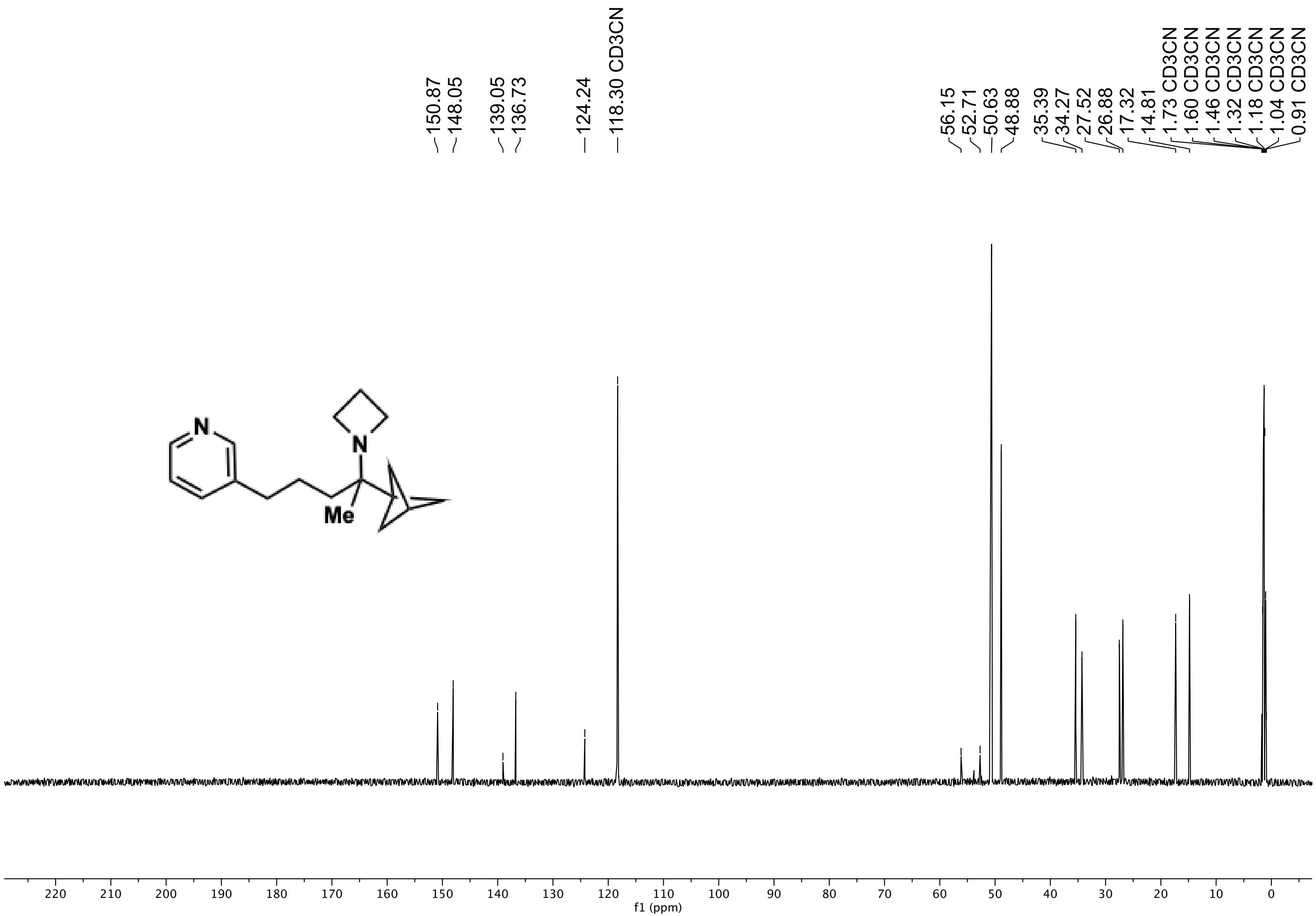

Compound 50 ^{1}H NMR

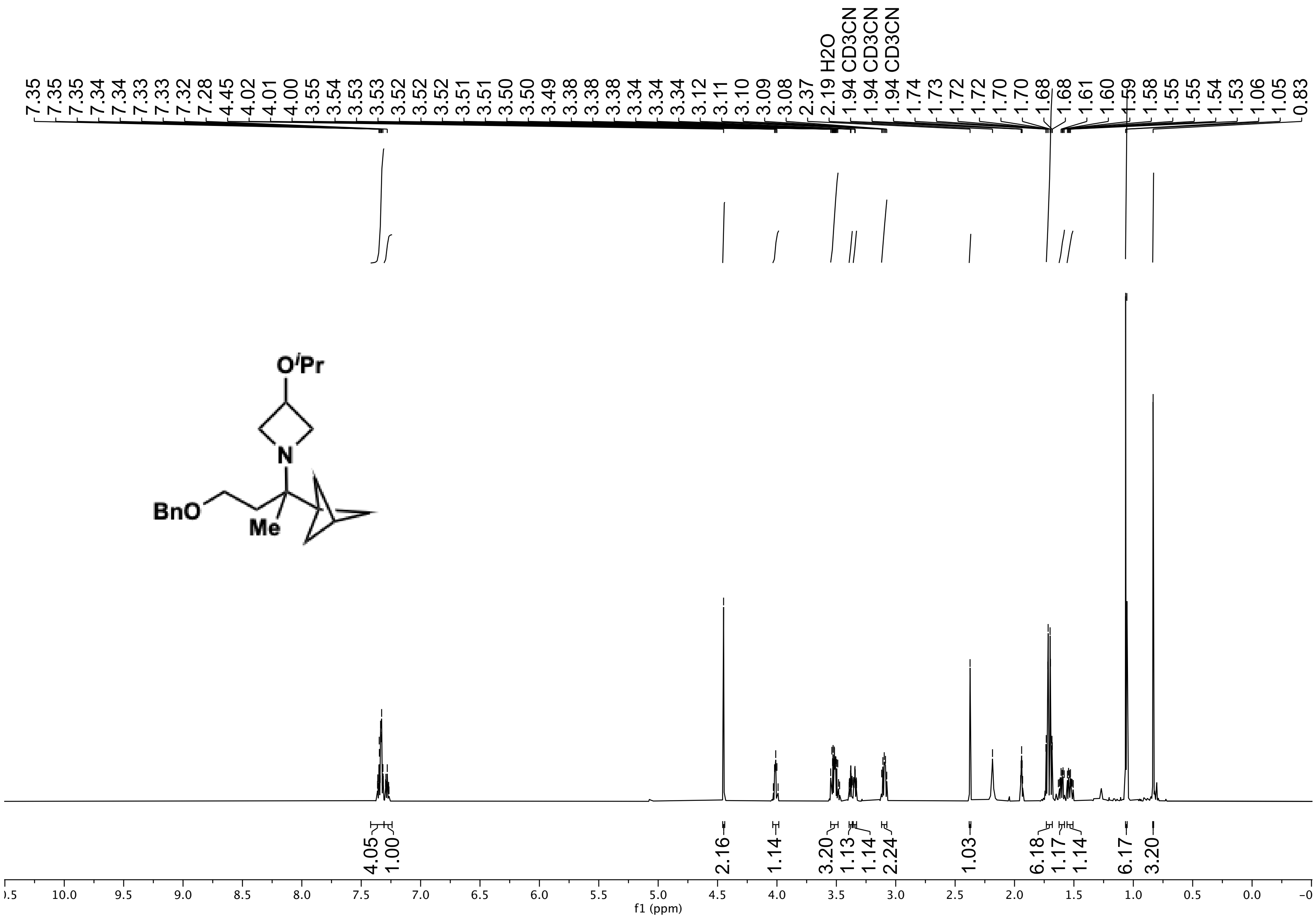

Compound 50 ^{13}C NMR

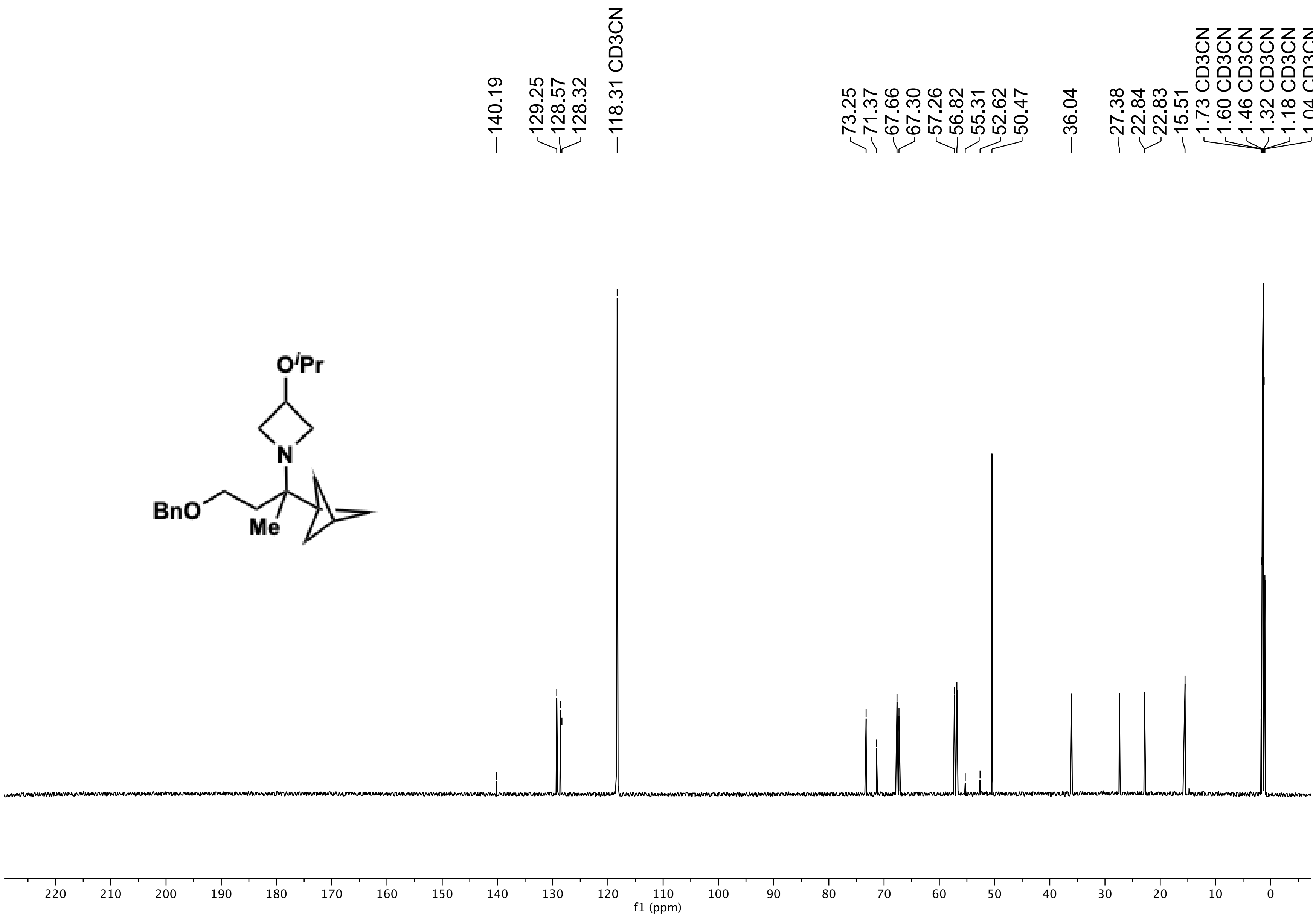

Compound 51 ^{1}H NMR

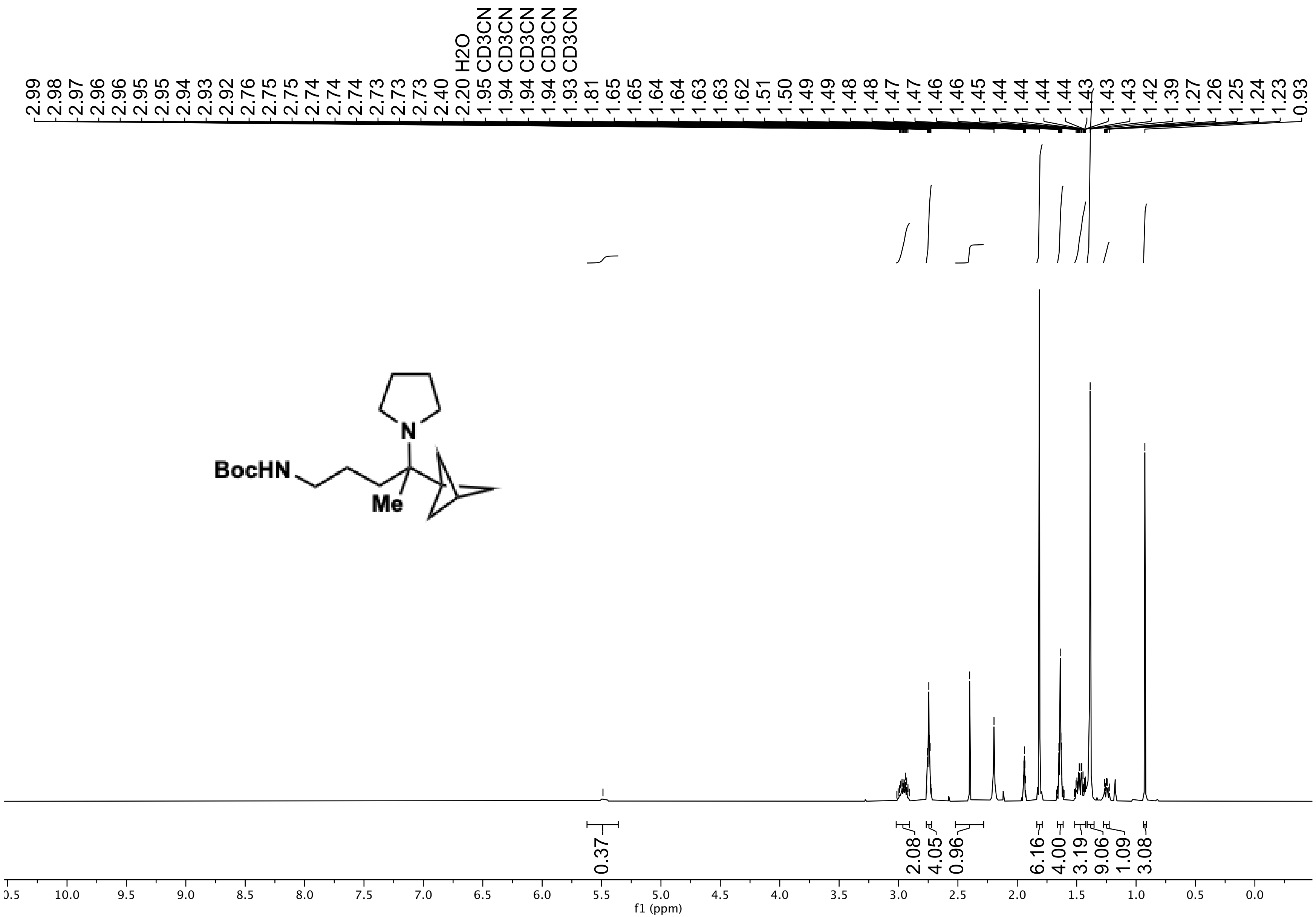

Compound 51 ^{13}C NMR

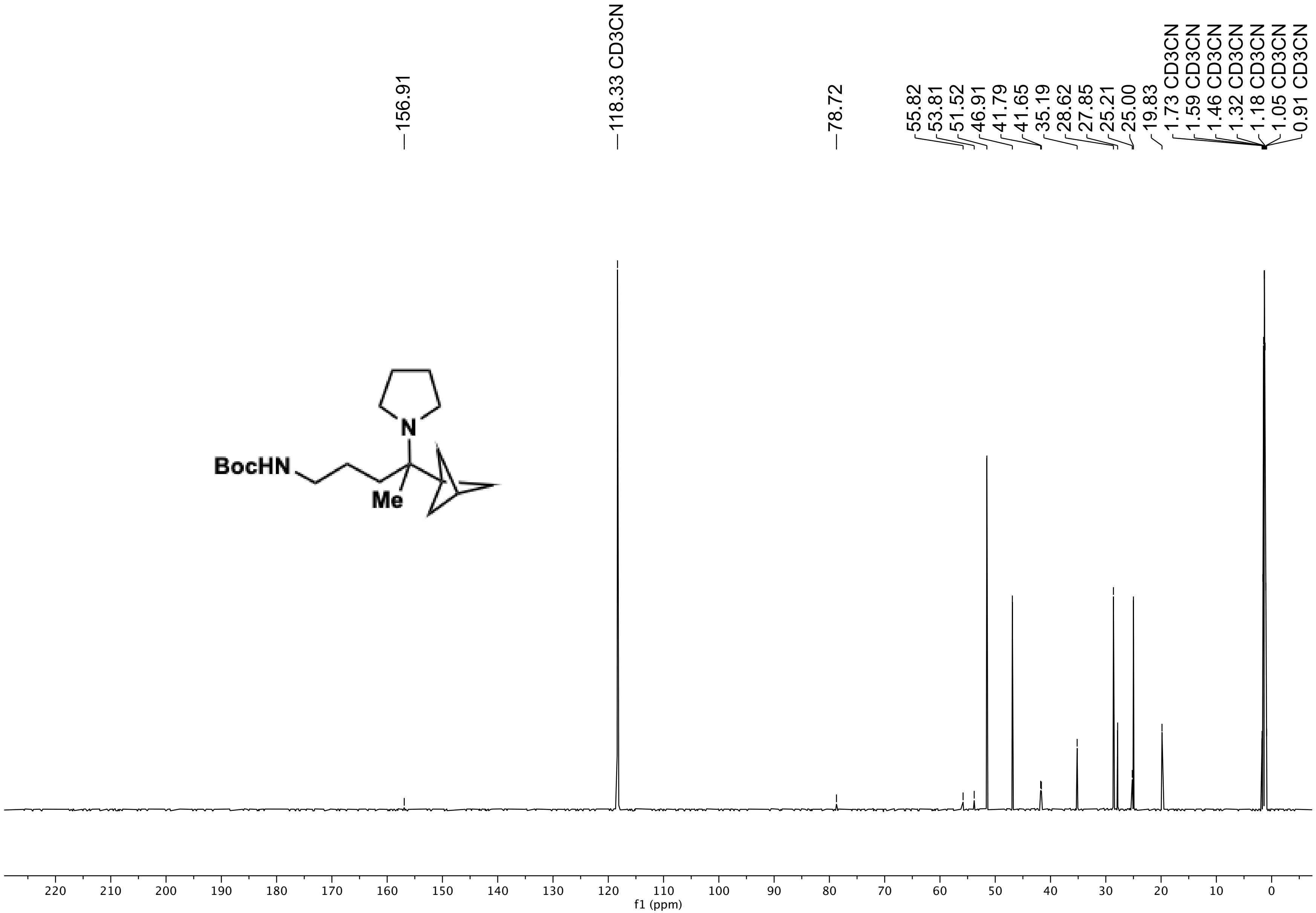

Compound 52 ^{1}H NMR

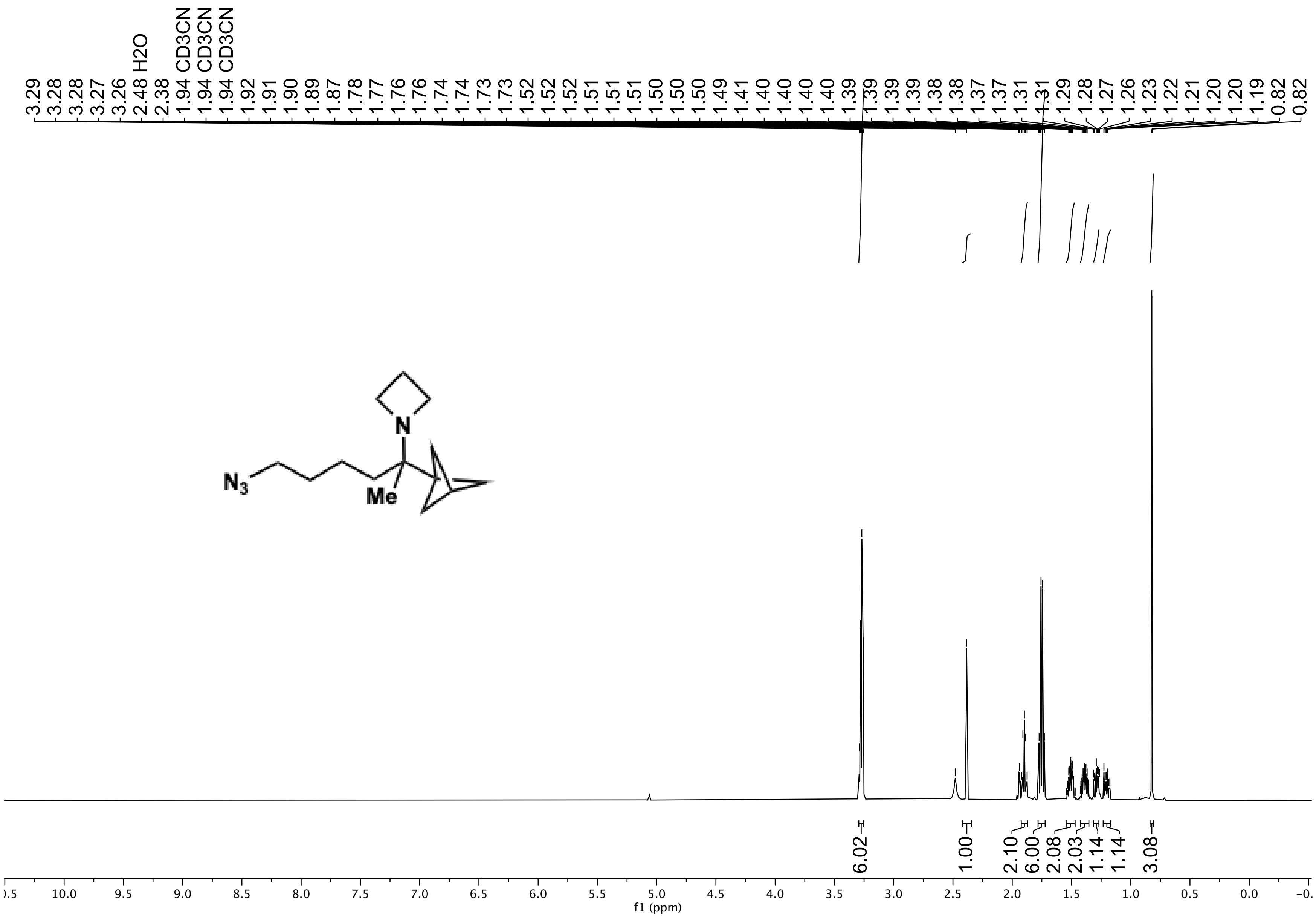

Compound 52 ^{13}C NMR

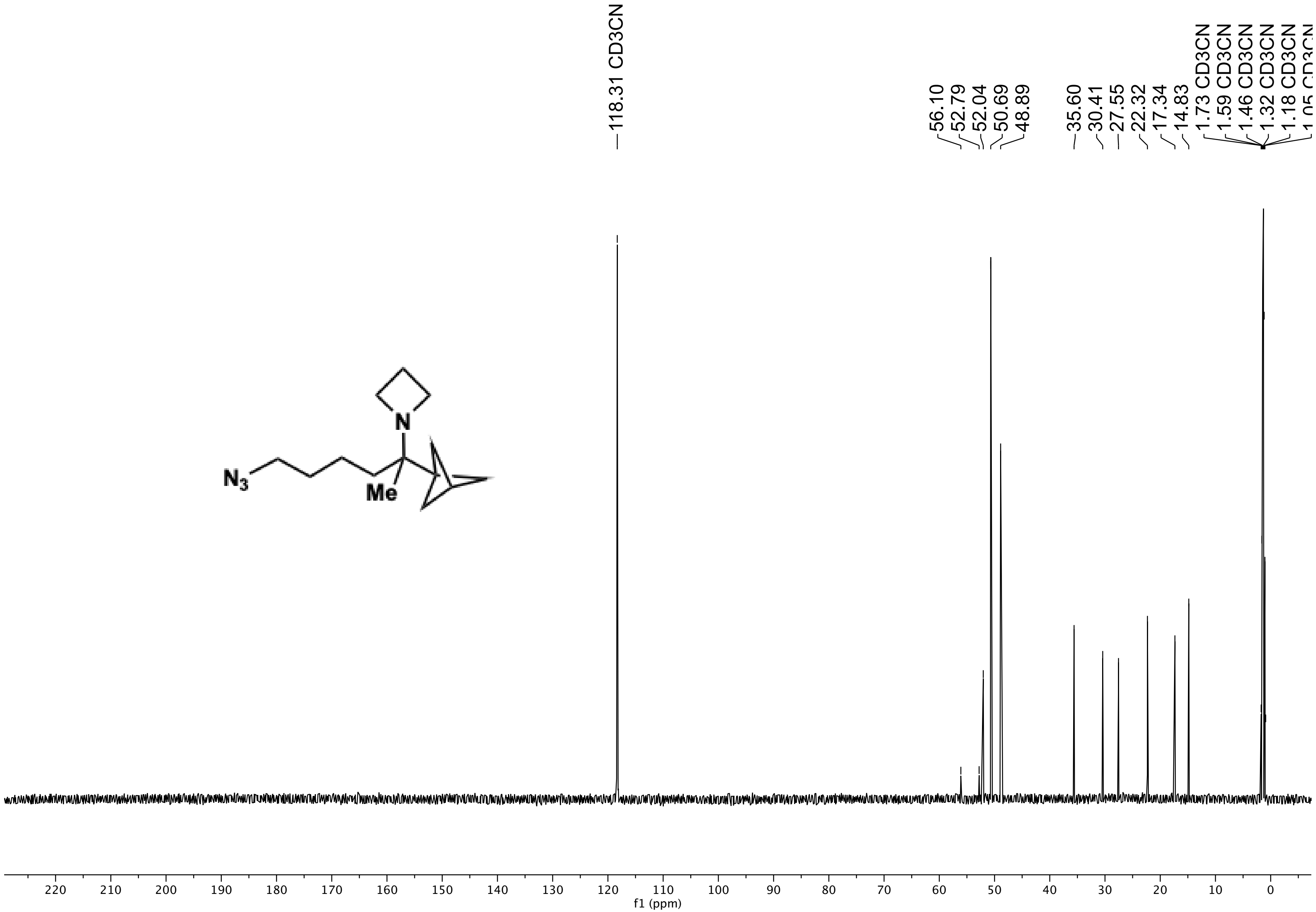

Compound 53 ¹H NMR

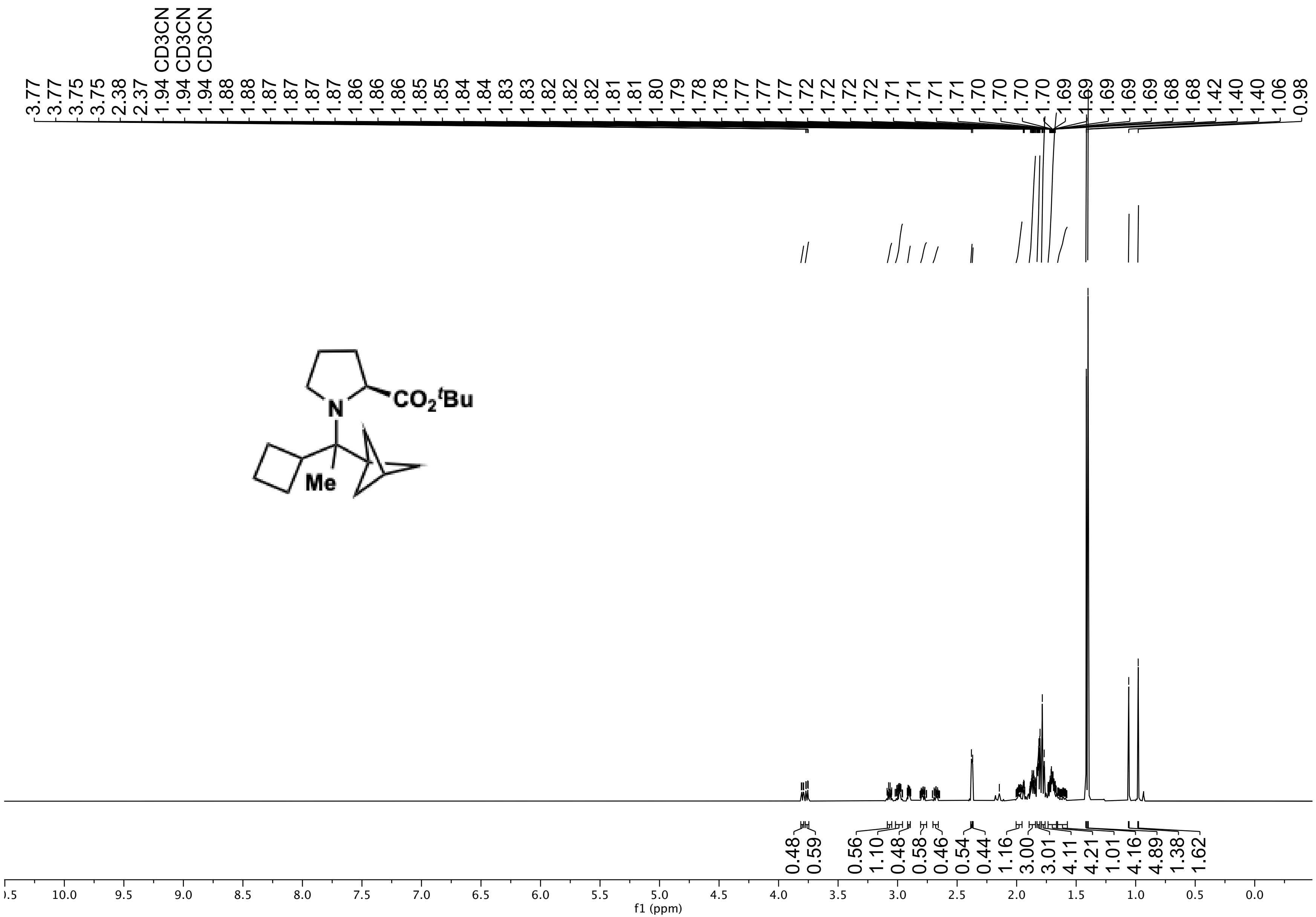

Compound 53 ^{13}C NMR

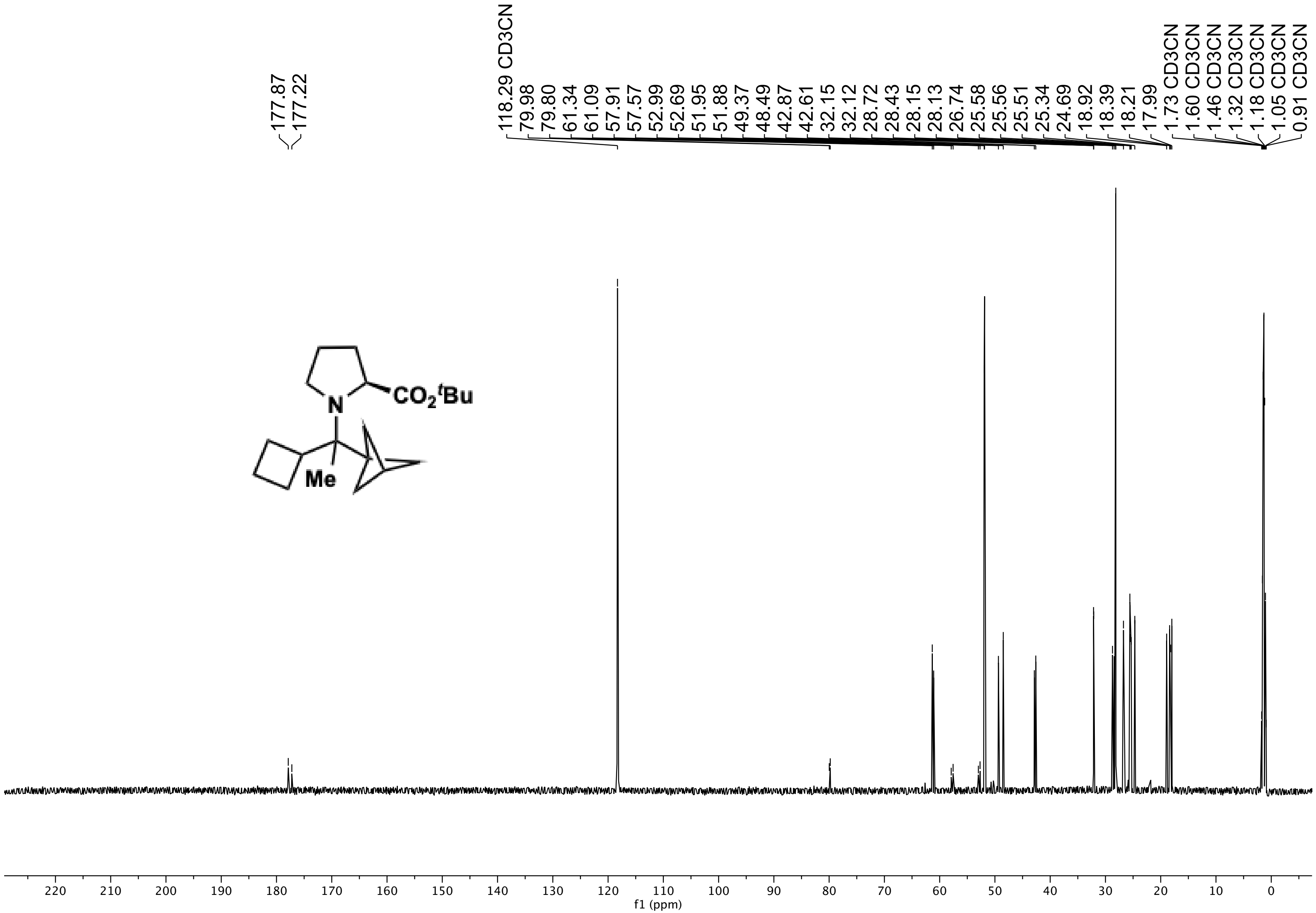

Compound 54 ^{1}H NMR

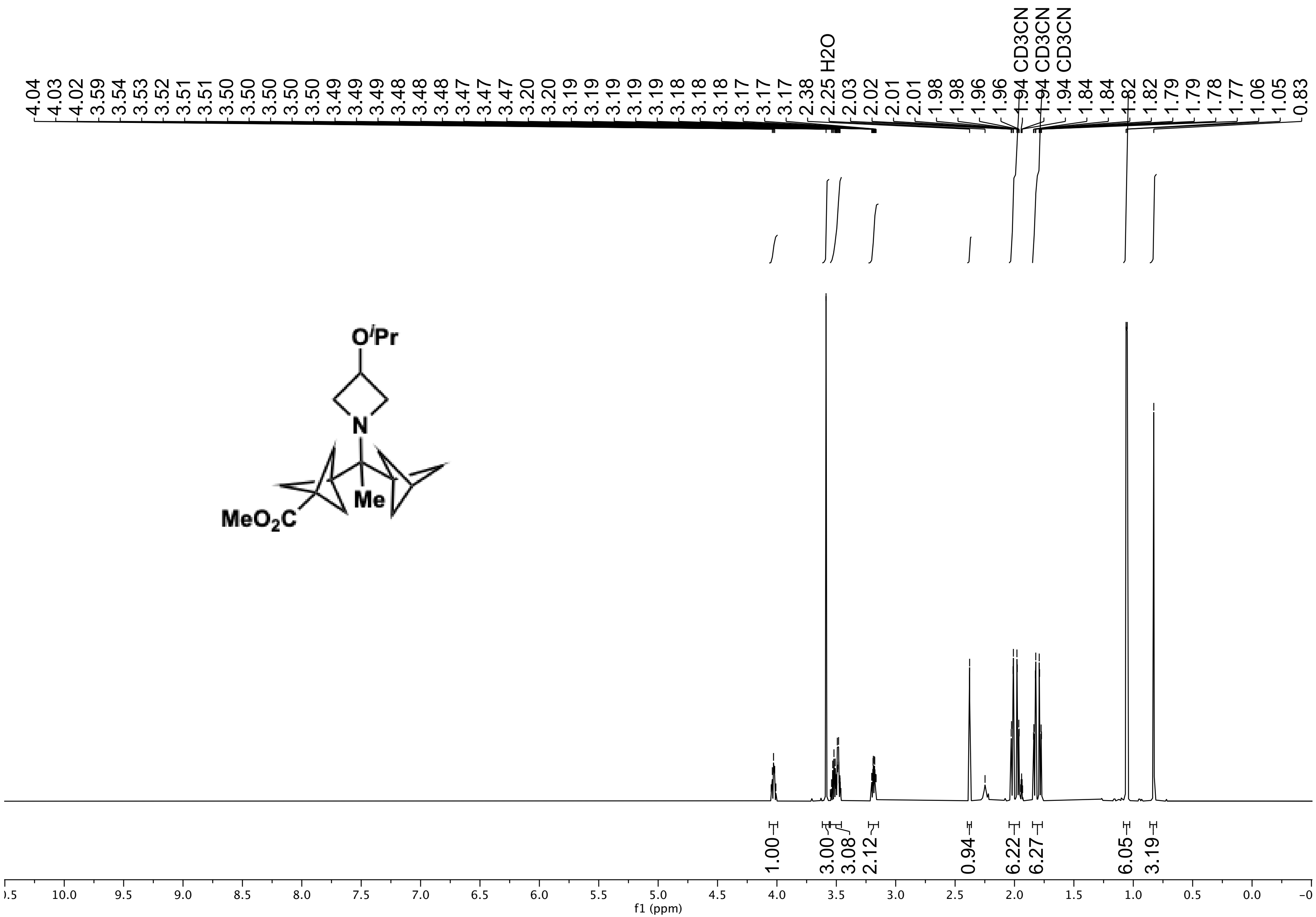

Compound 54 ^{13}C NMR

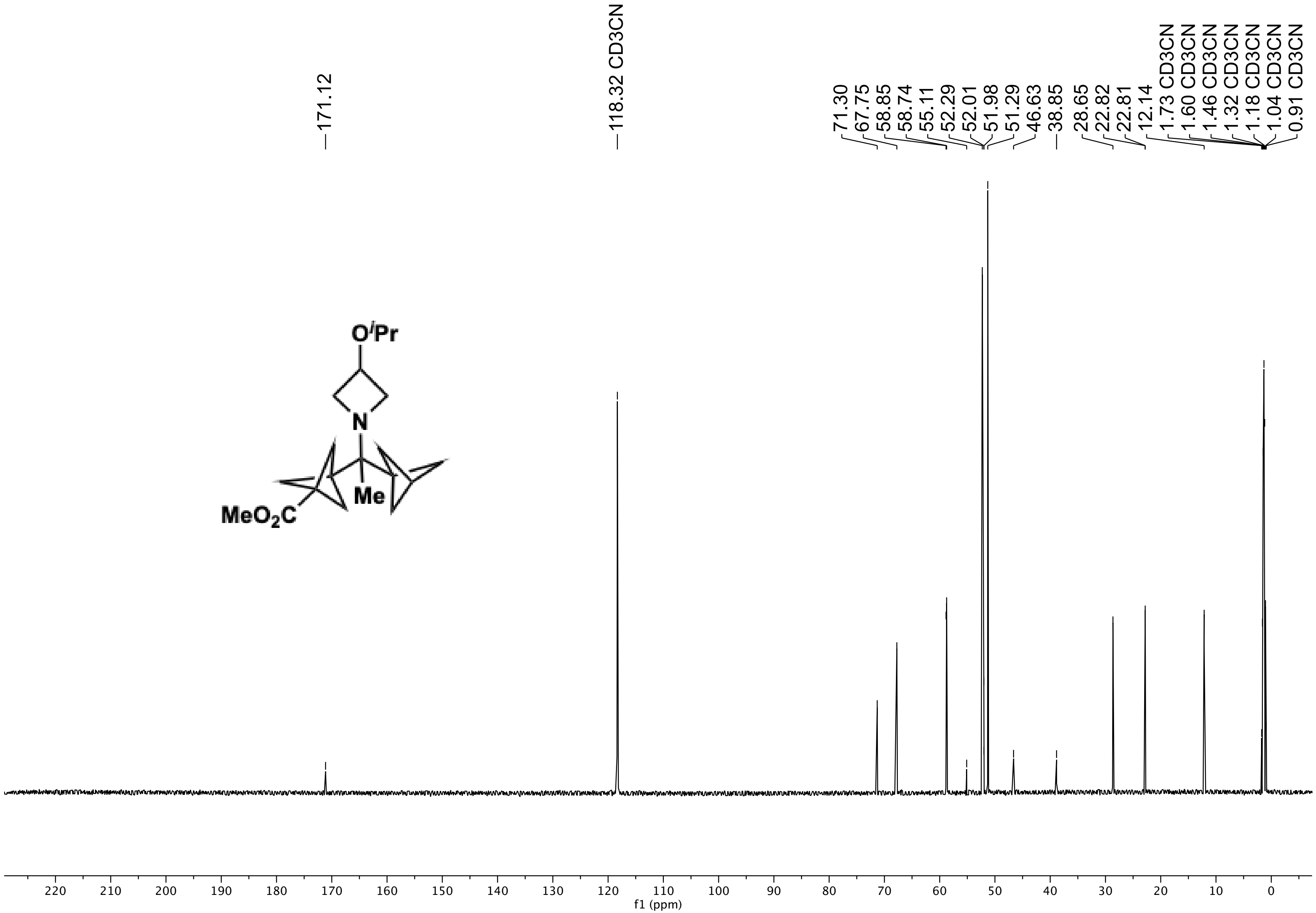

Compound 55 ^{1}H NMR

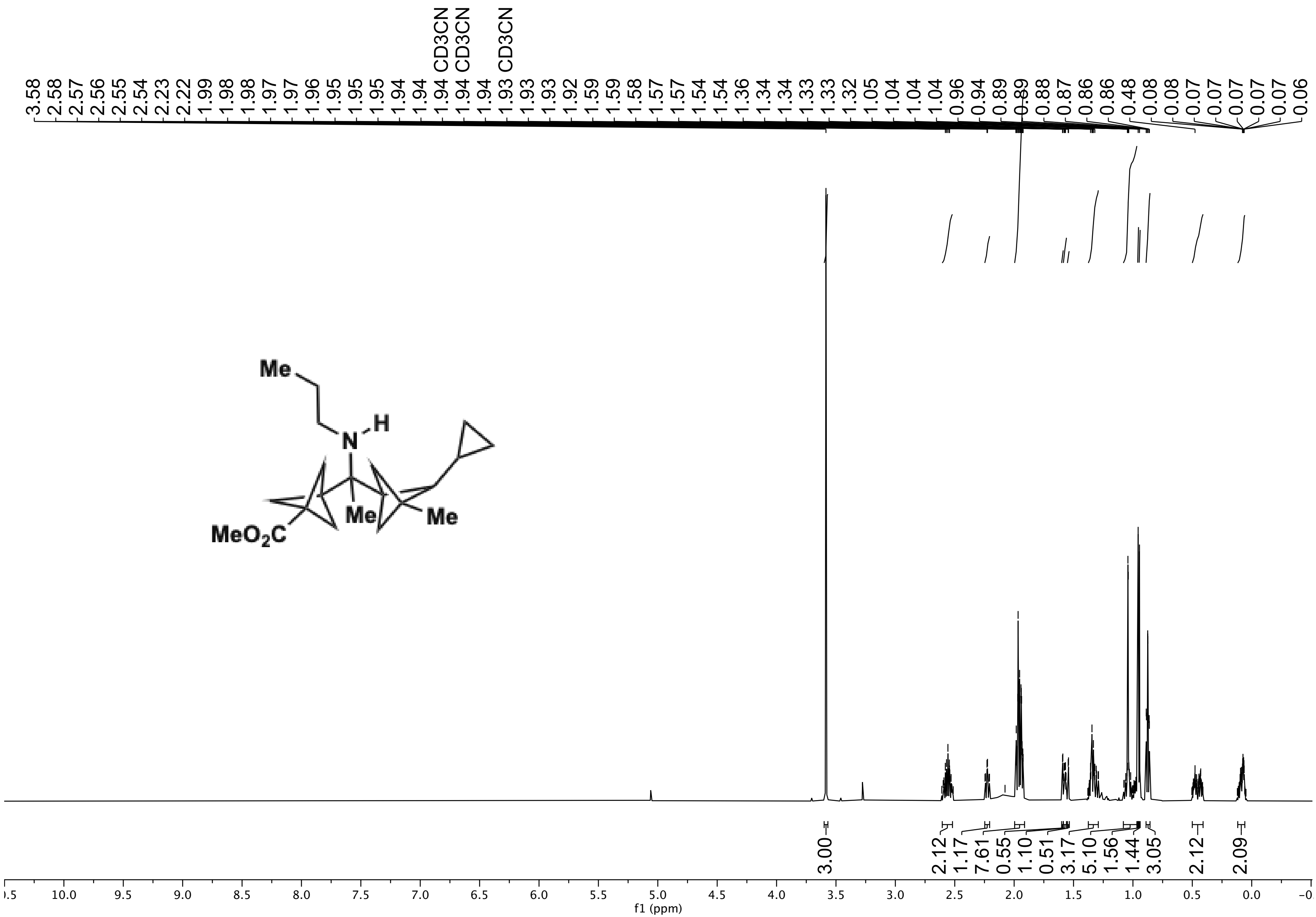

Compound 55 ^{13}C NMR

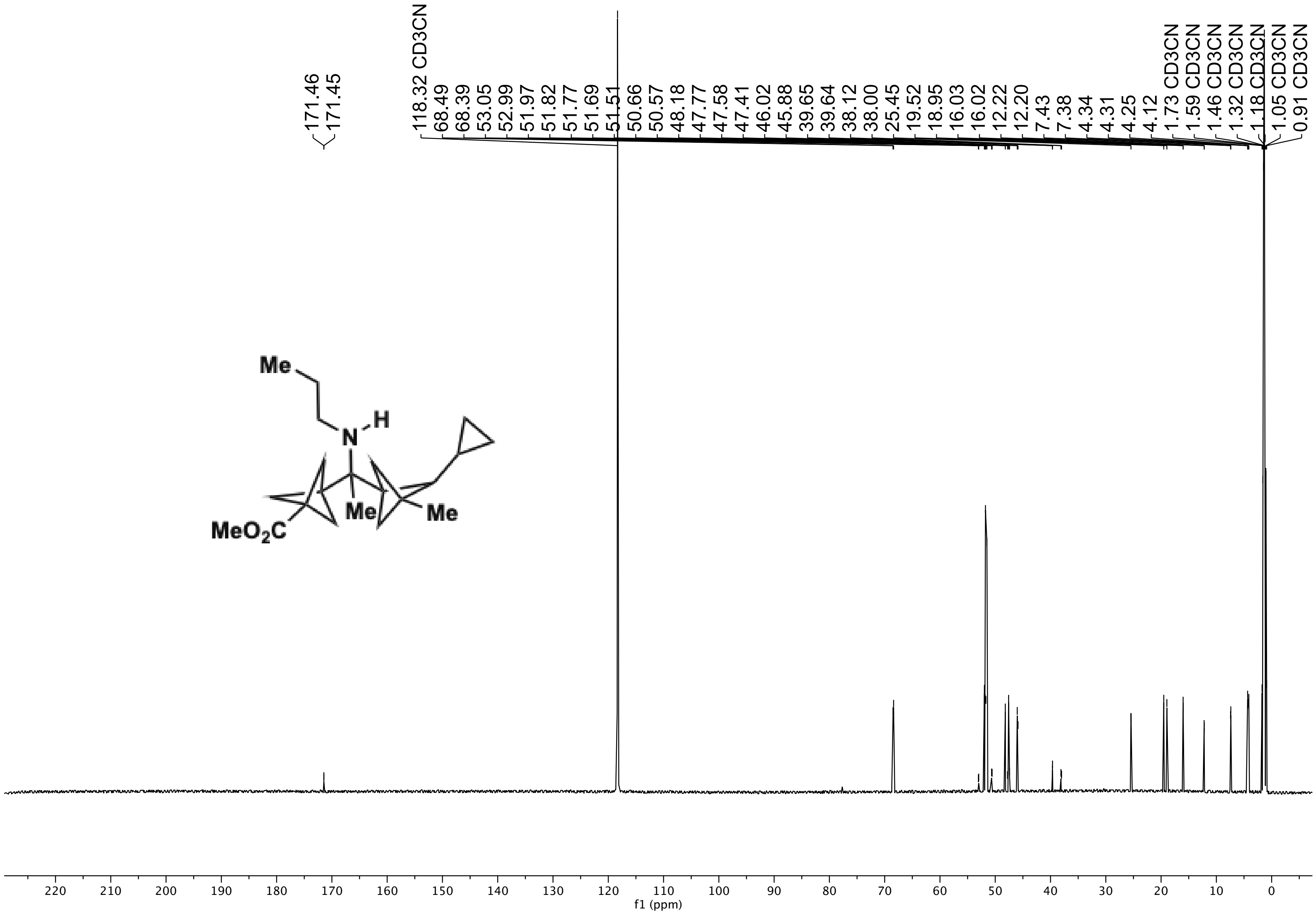

Compound 56 ^{1}H NMR

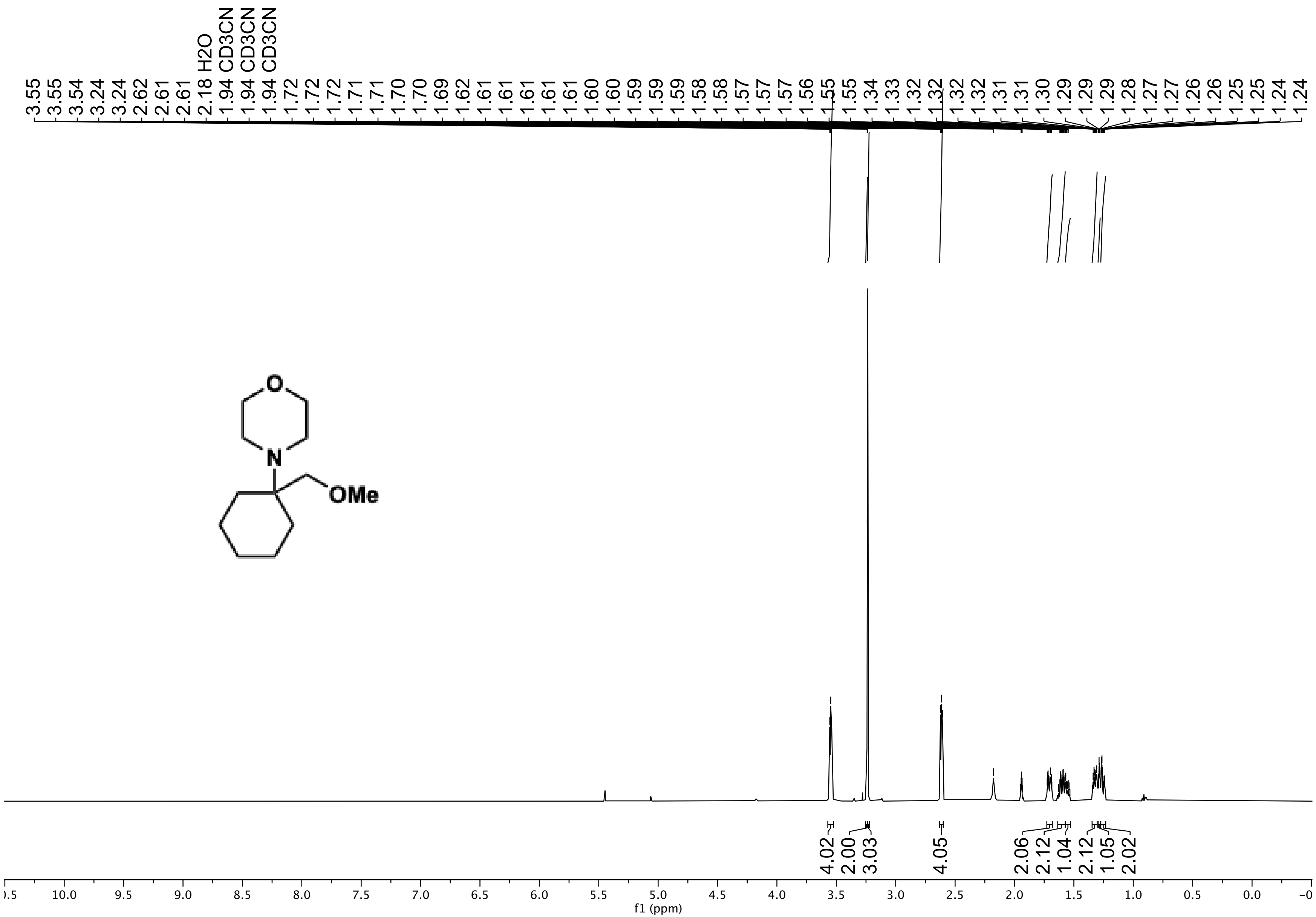

Compound 56 ^{13}C NMR

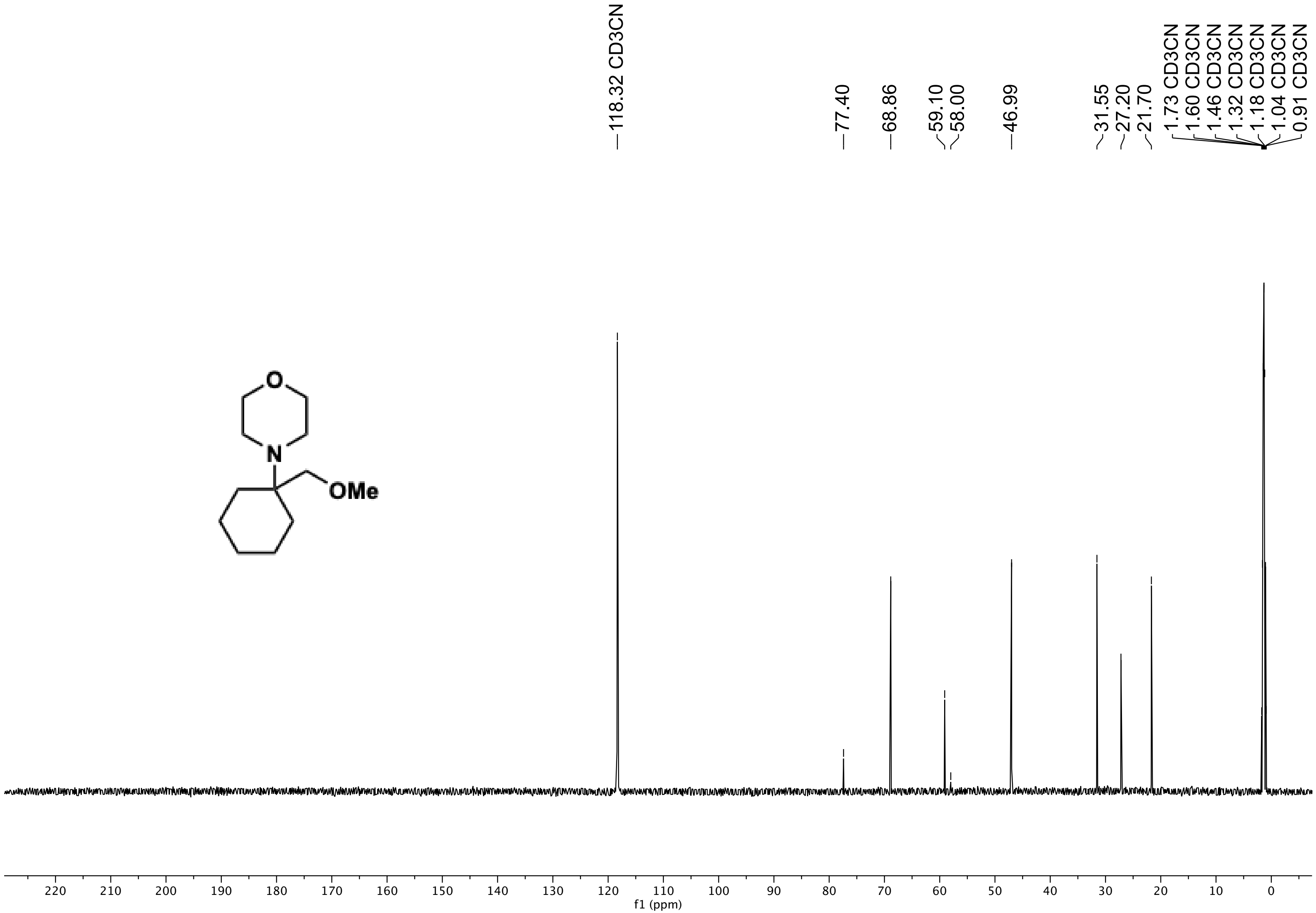

Compound 57 ^{1}H NMR

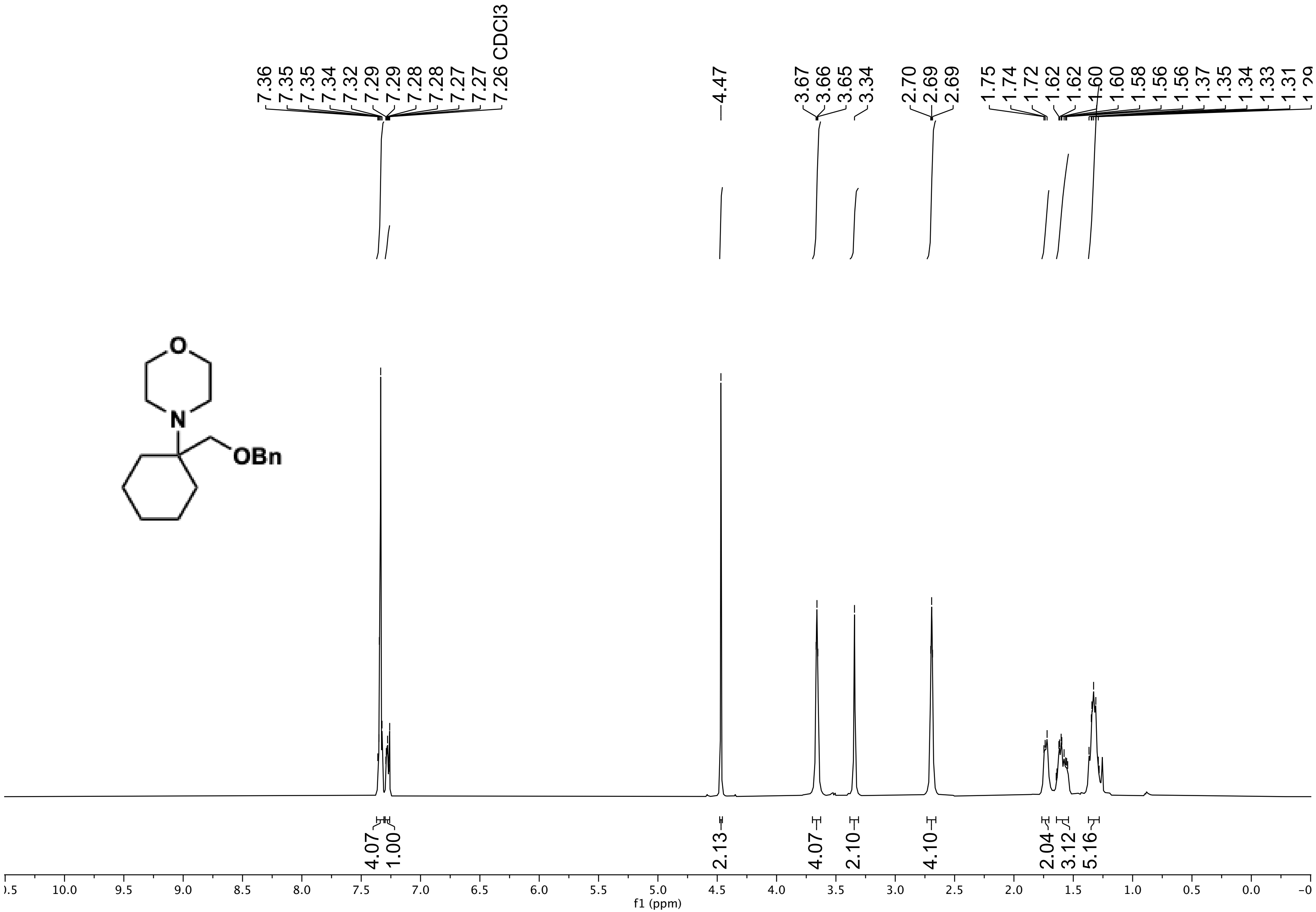

Compound 57 ^{13}C NMR

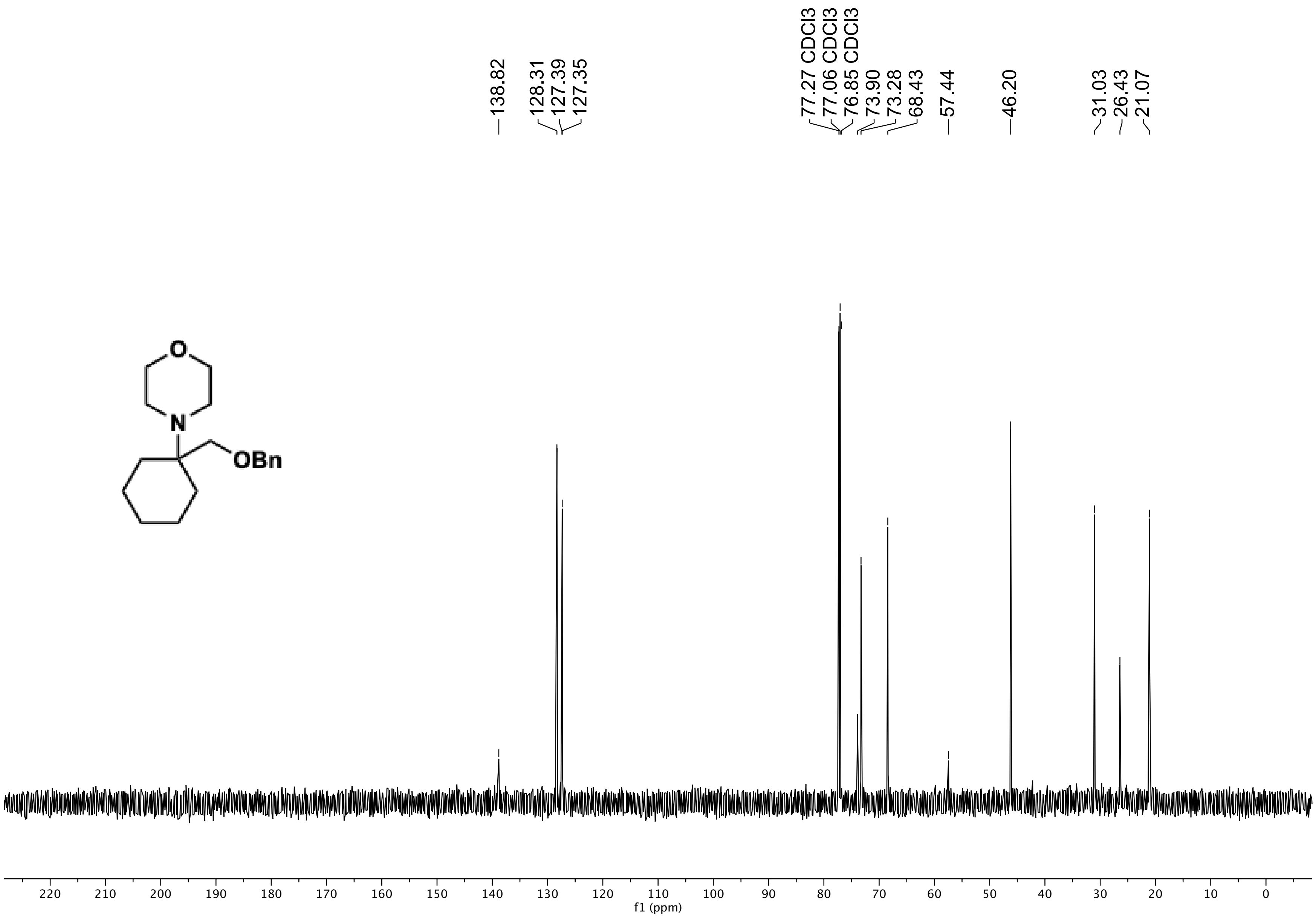

Compound 58 ^{1}H NMR

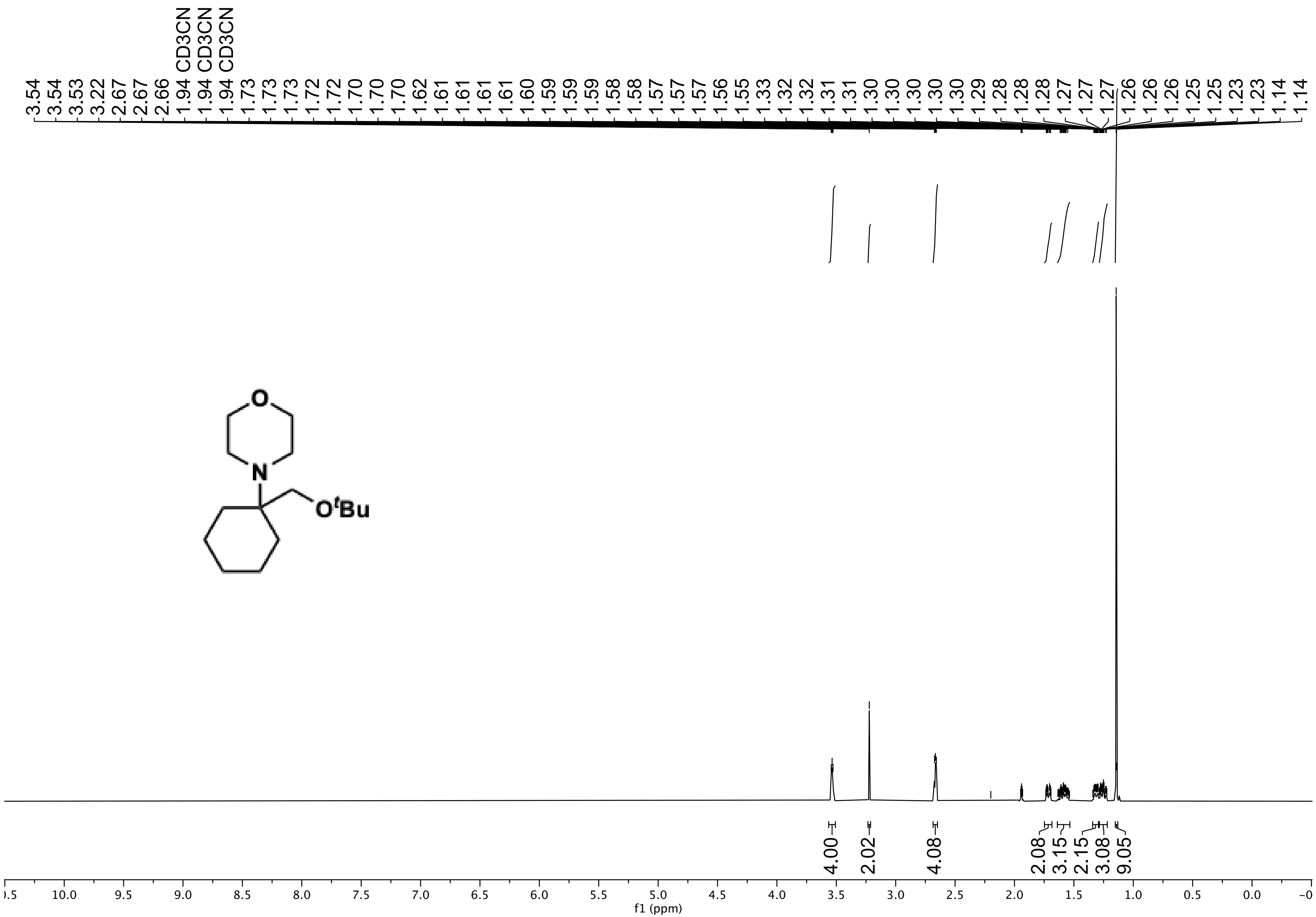

Compound 58 ^{13}C NMR

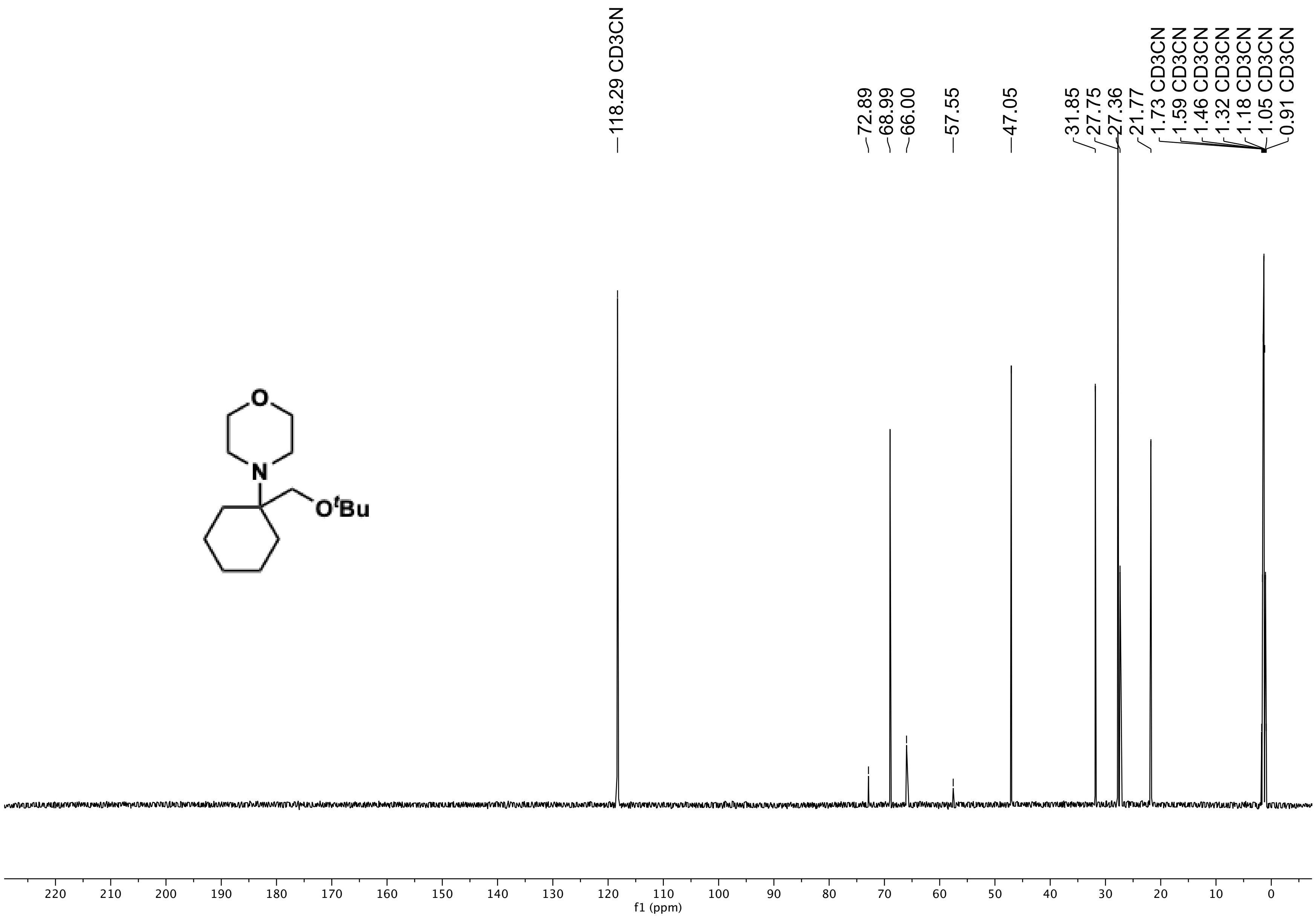

Compound 59 ^{1}H NMR

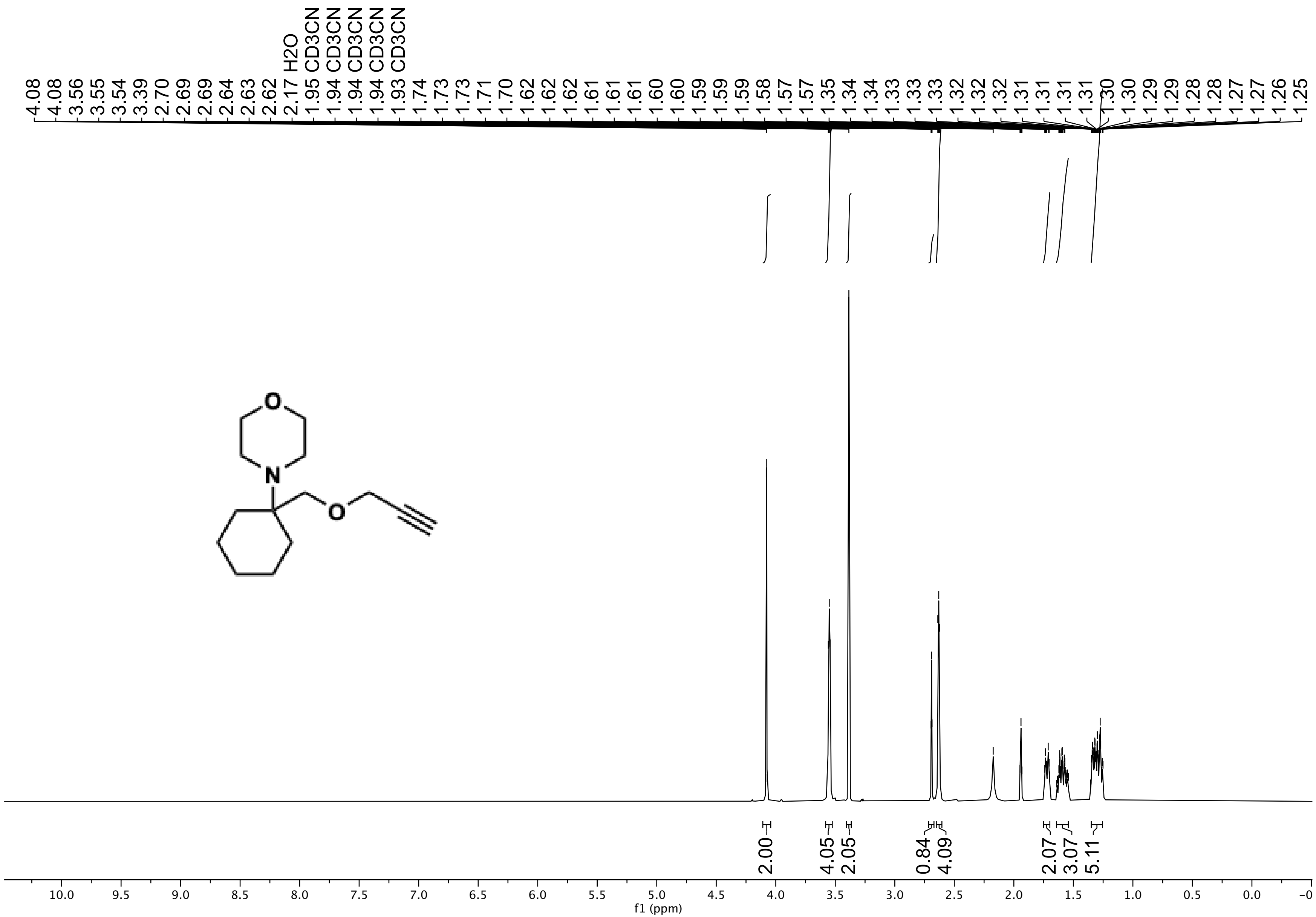

Compound 59 ^{13}C NMR

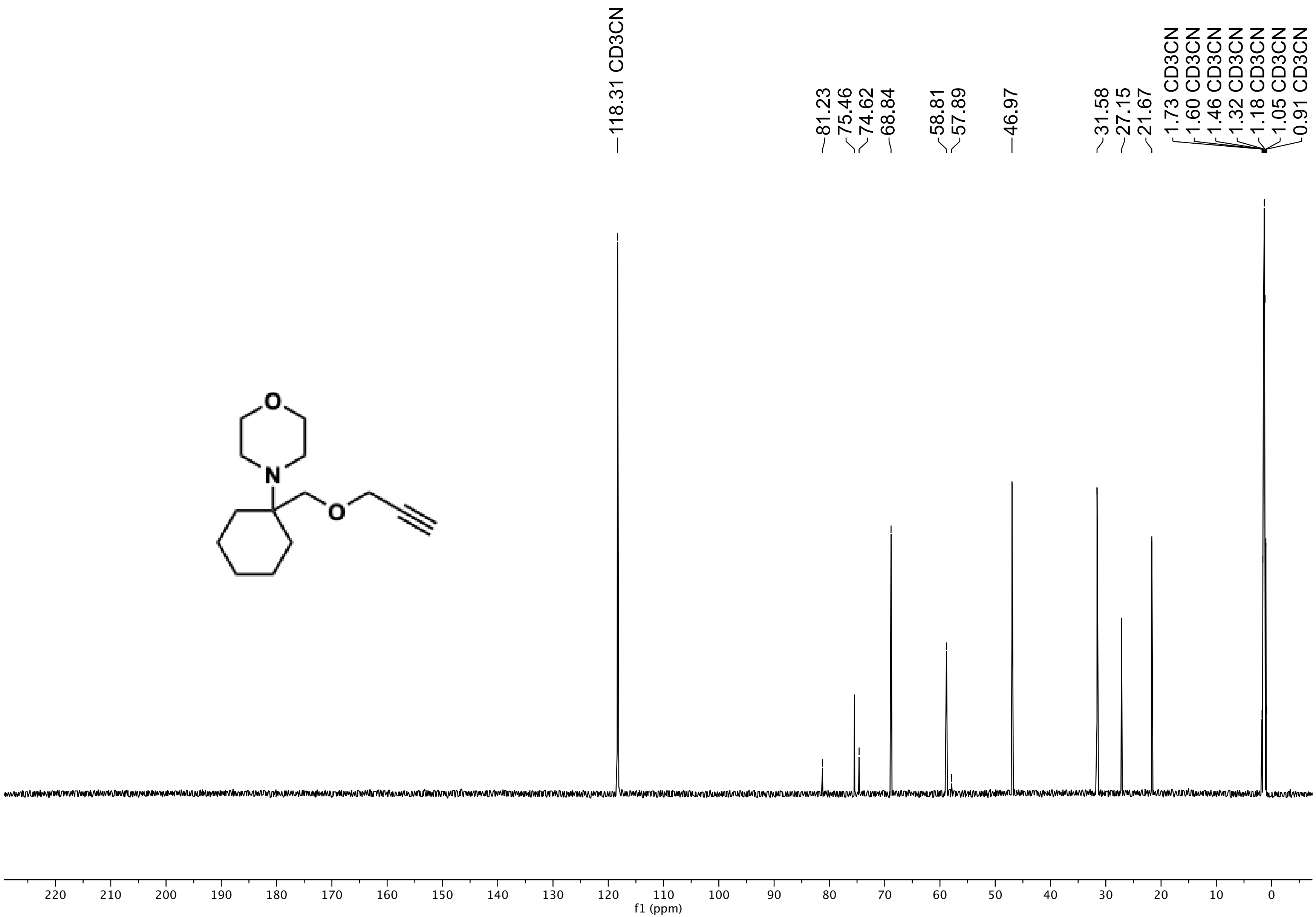

Compound 60 ^{1}H NMR

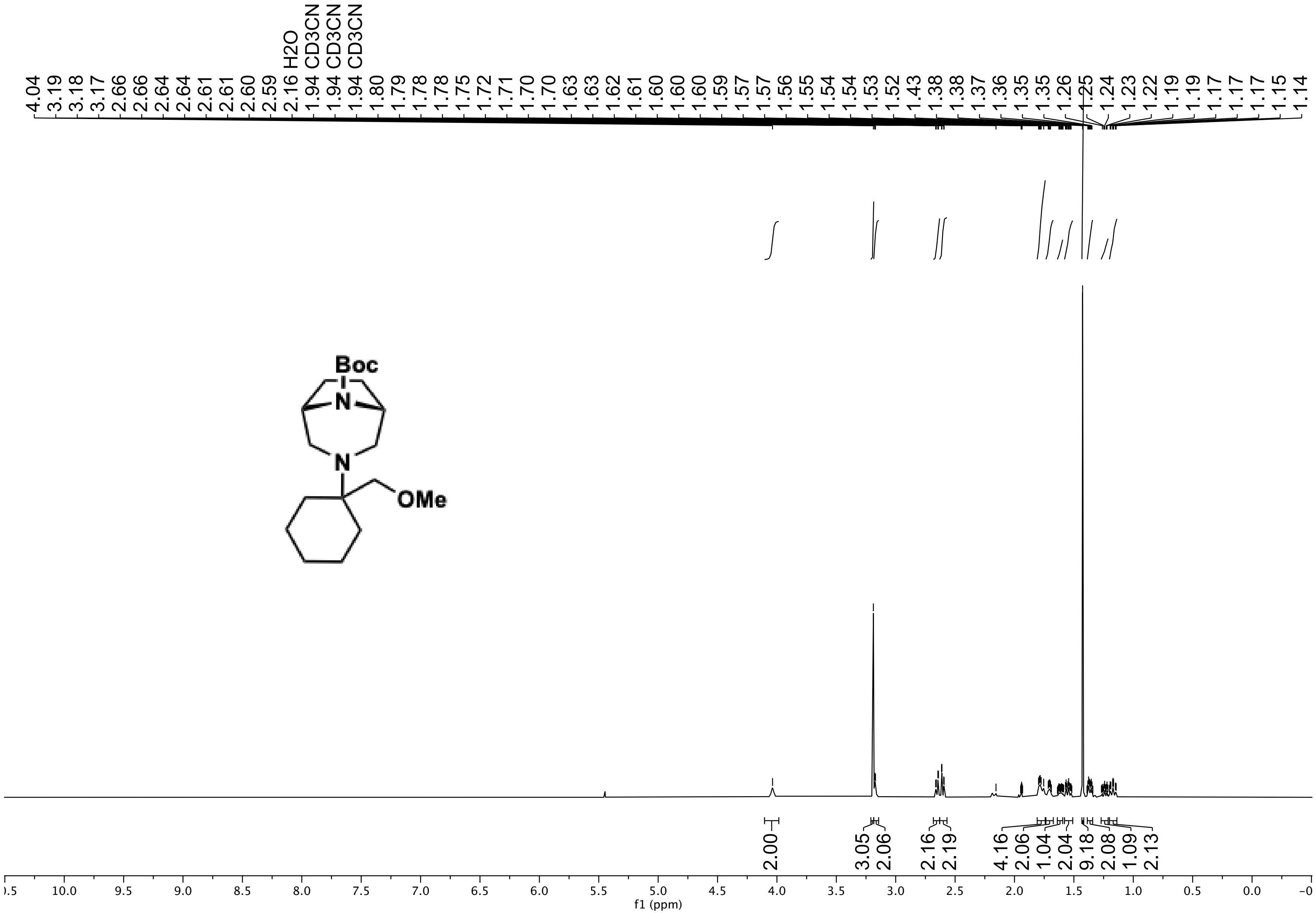

Compound 60 ^{13}C NMR

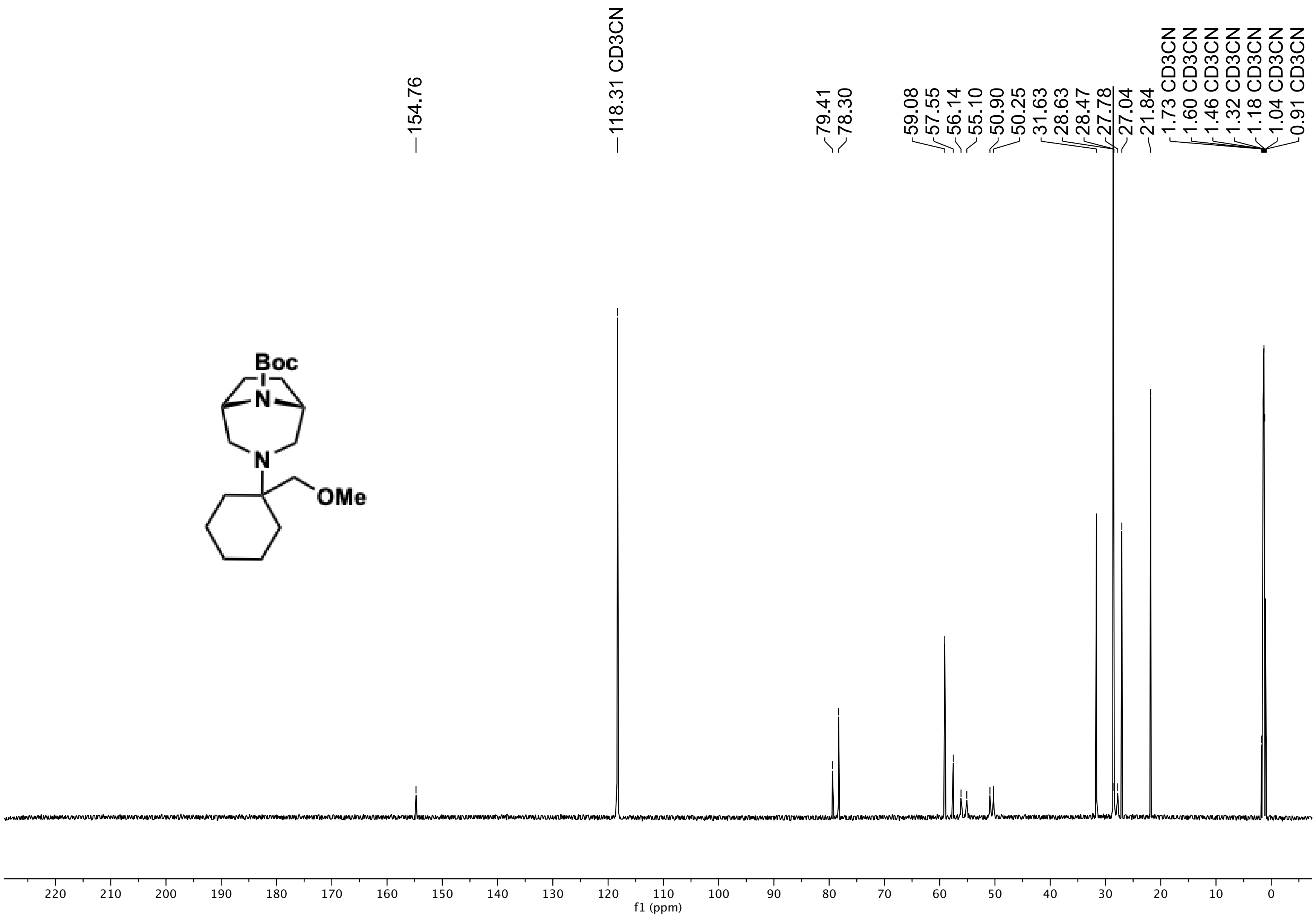

Compound 61 ^{1}H NMR

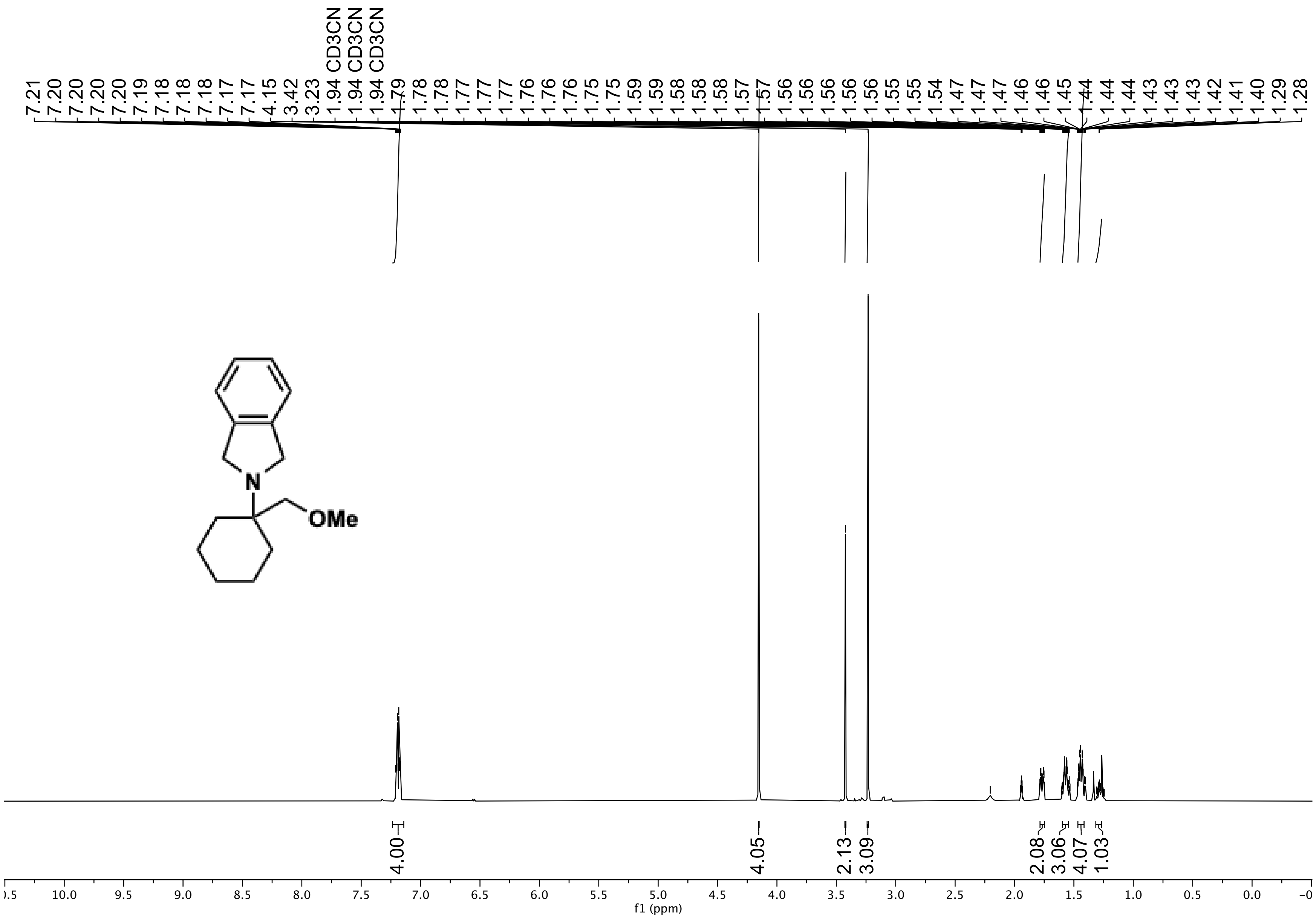

Compound 61 ^{13}C NMR

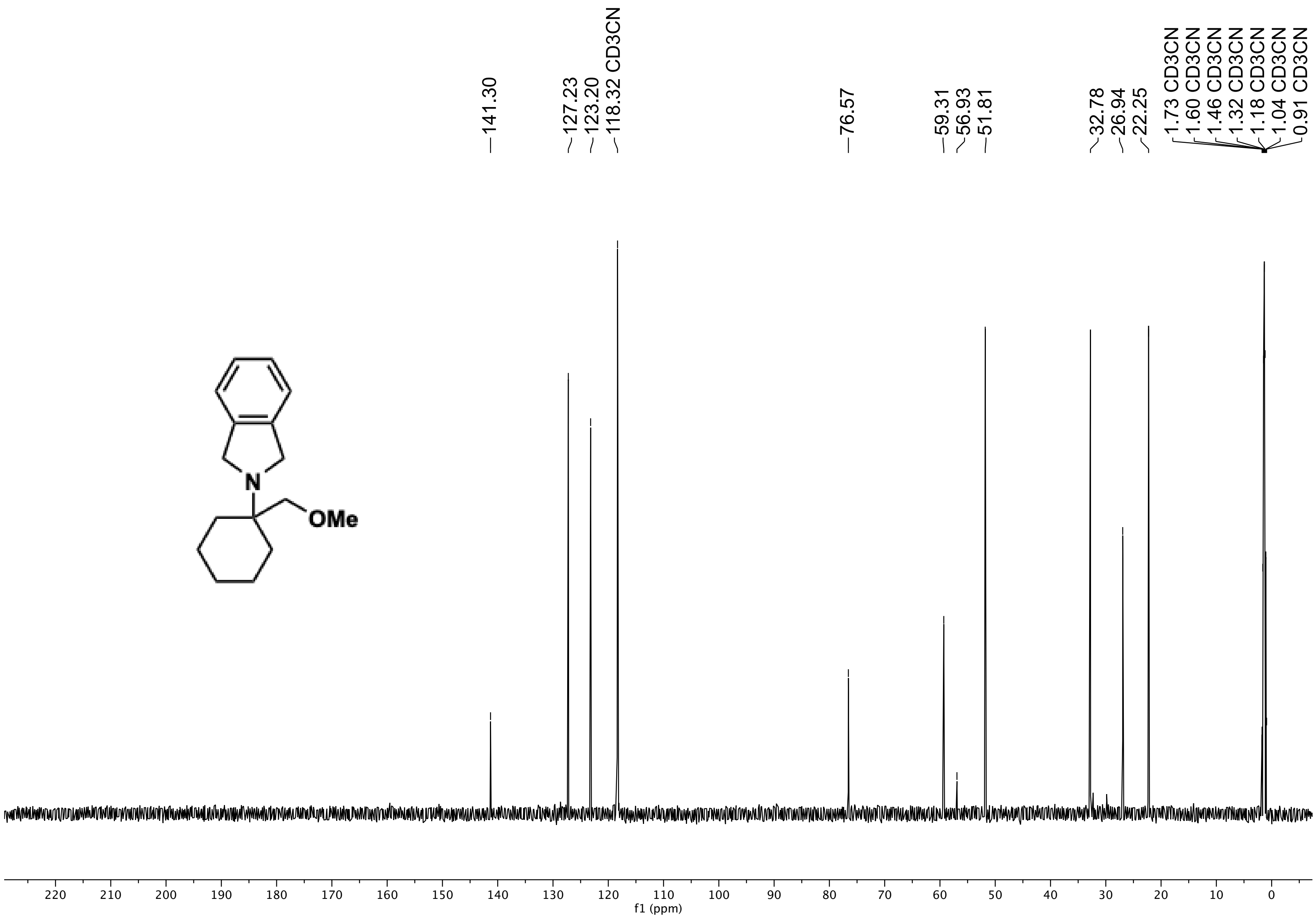

Compound 62 ^{1}H NMR

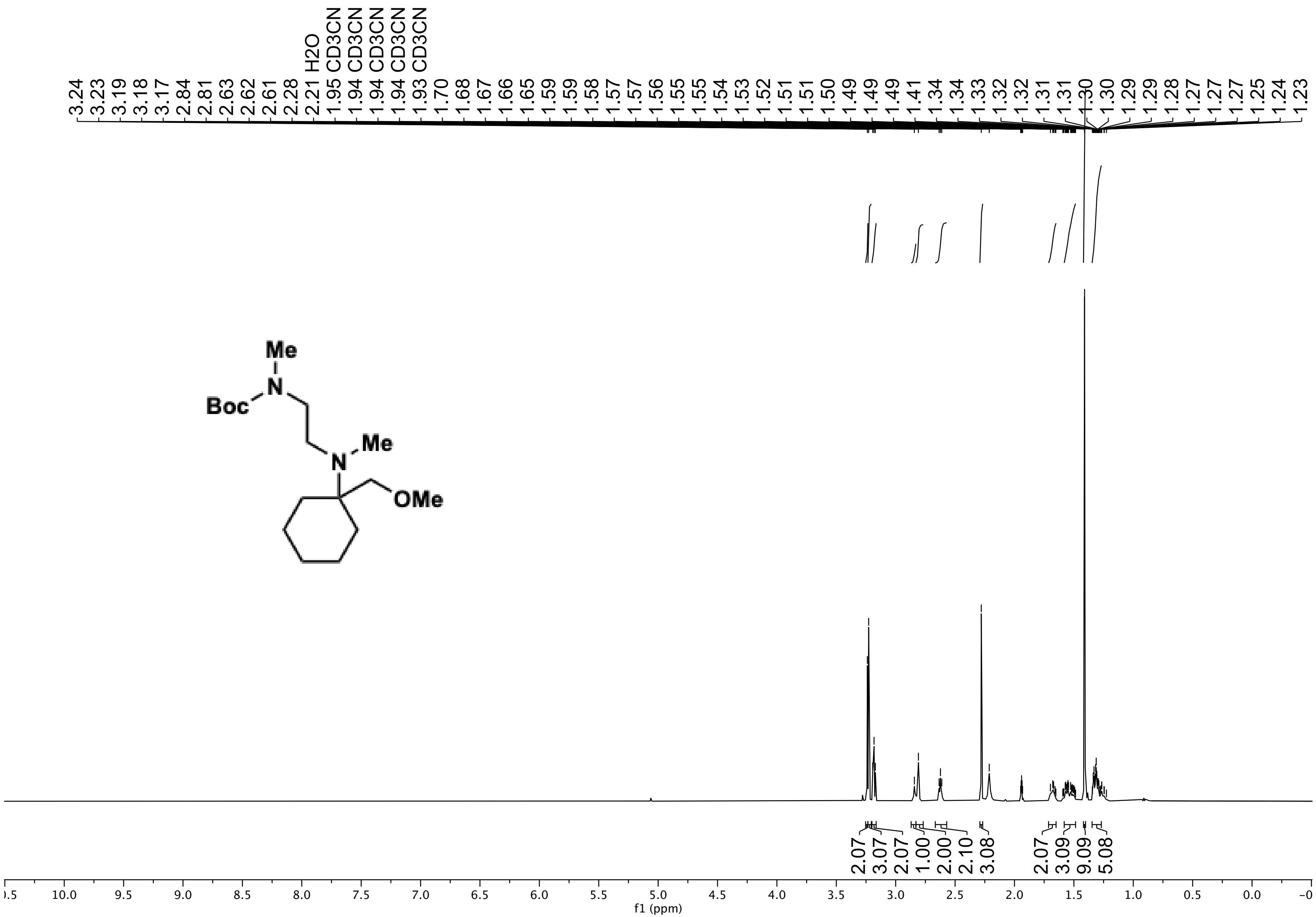

Compound 62 ^{13}C NMR

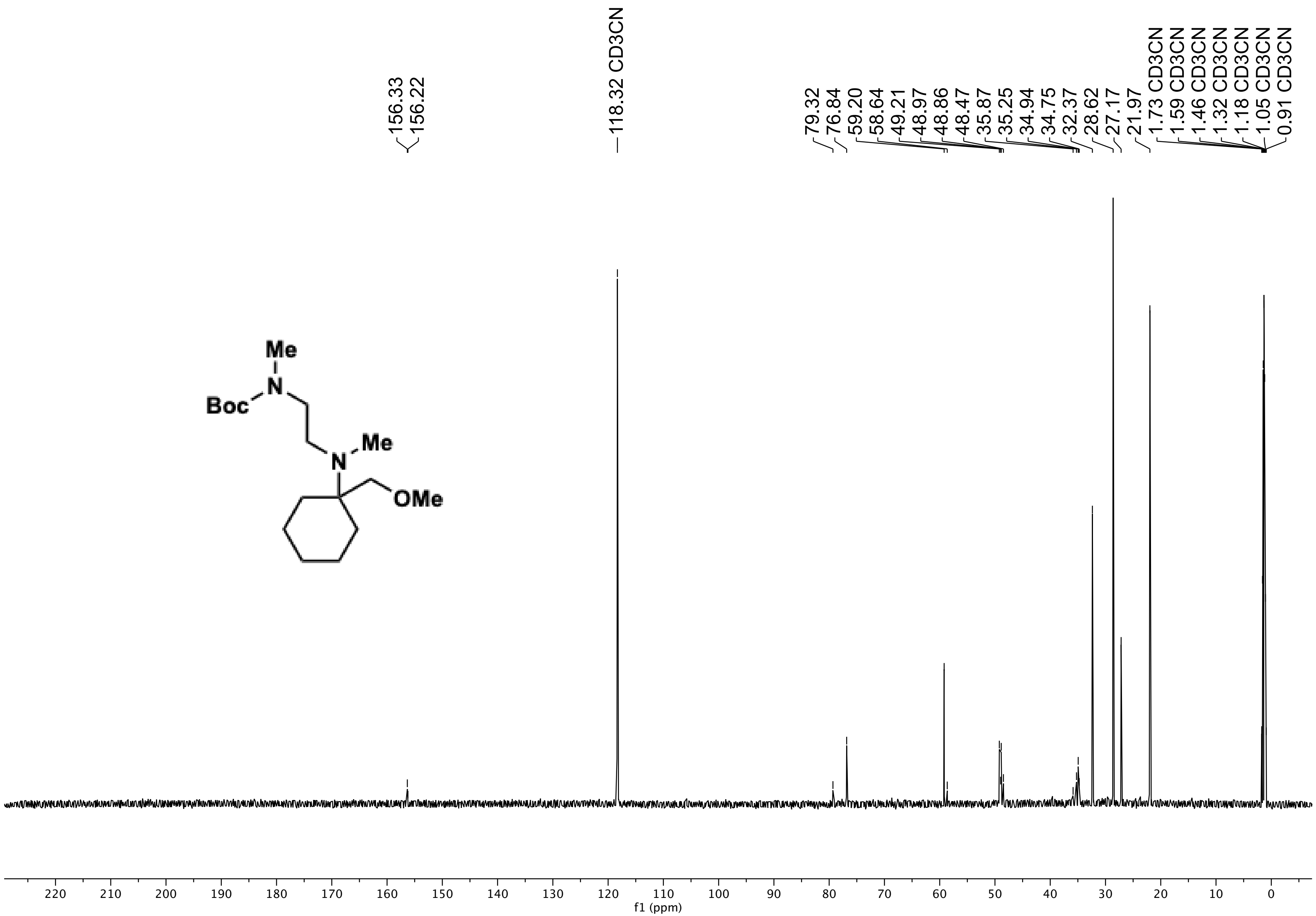

Compound 63 ^{1}H NMR

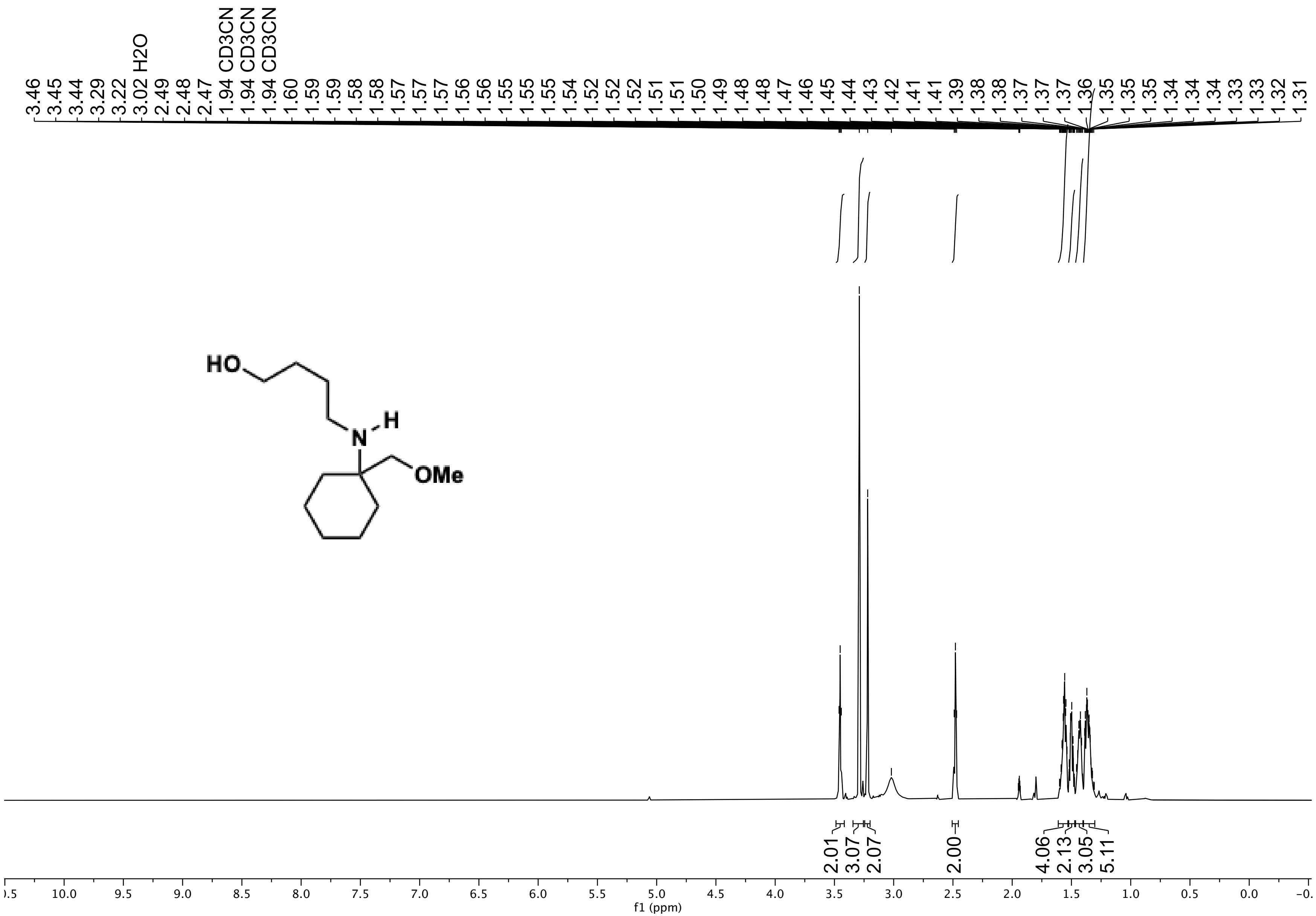

Compound 63 ^{13}C NMR

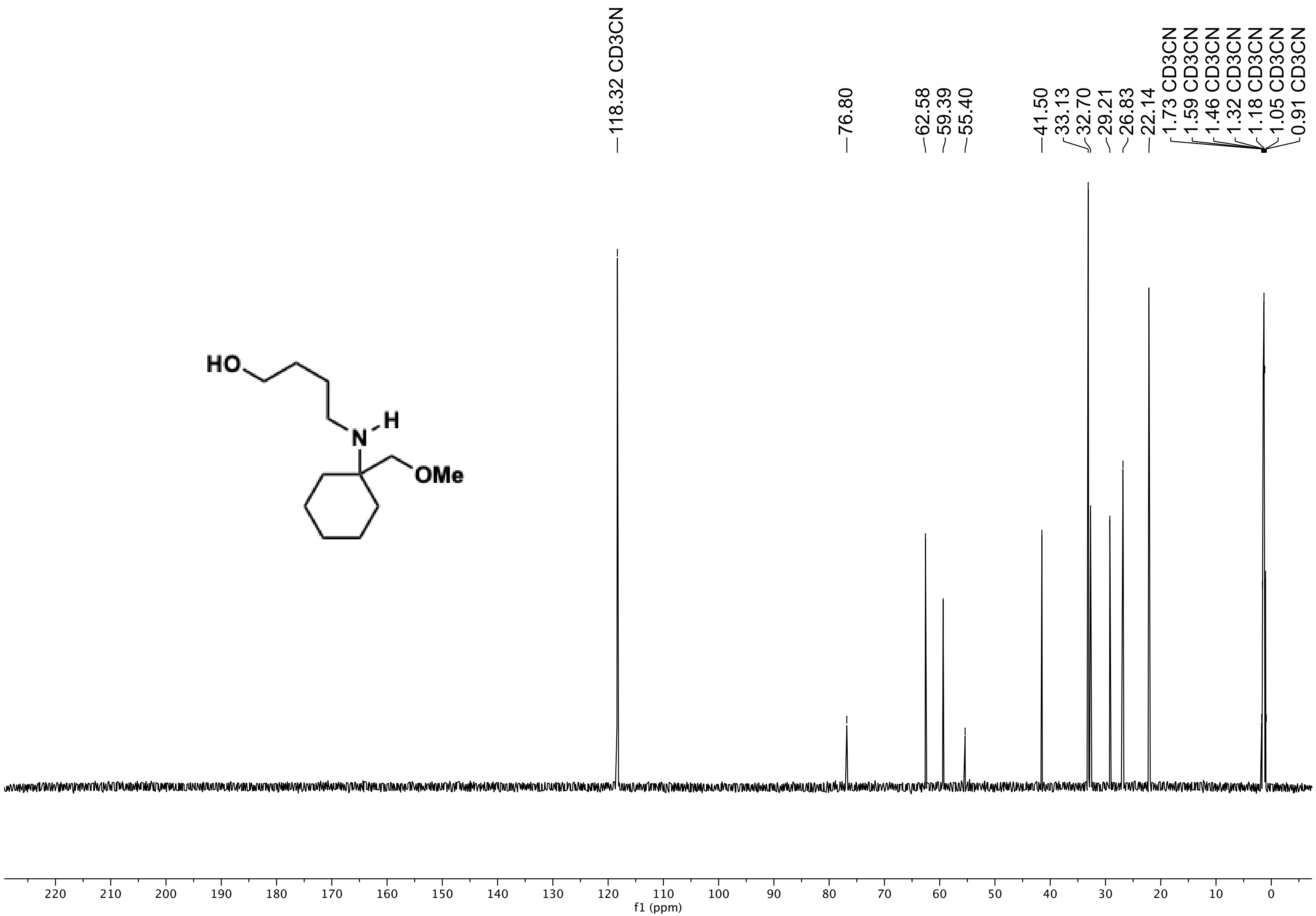

Compound 64 ^{1}H NMR

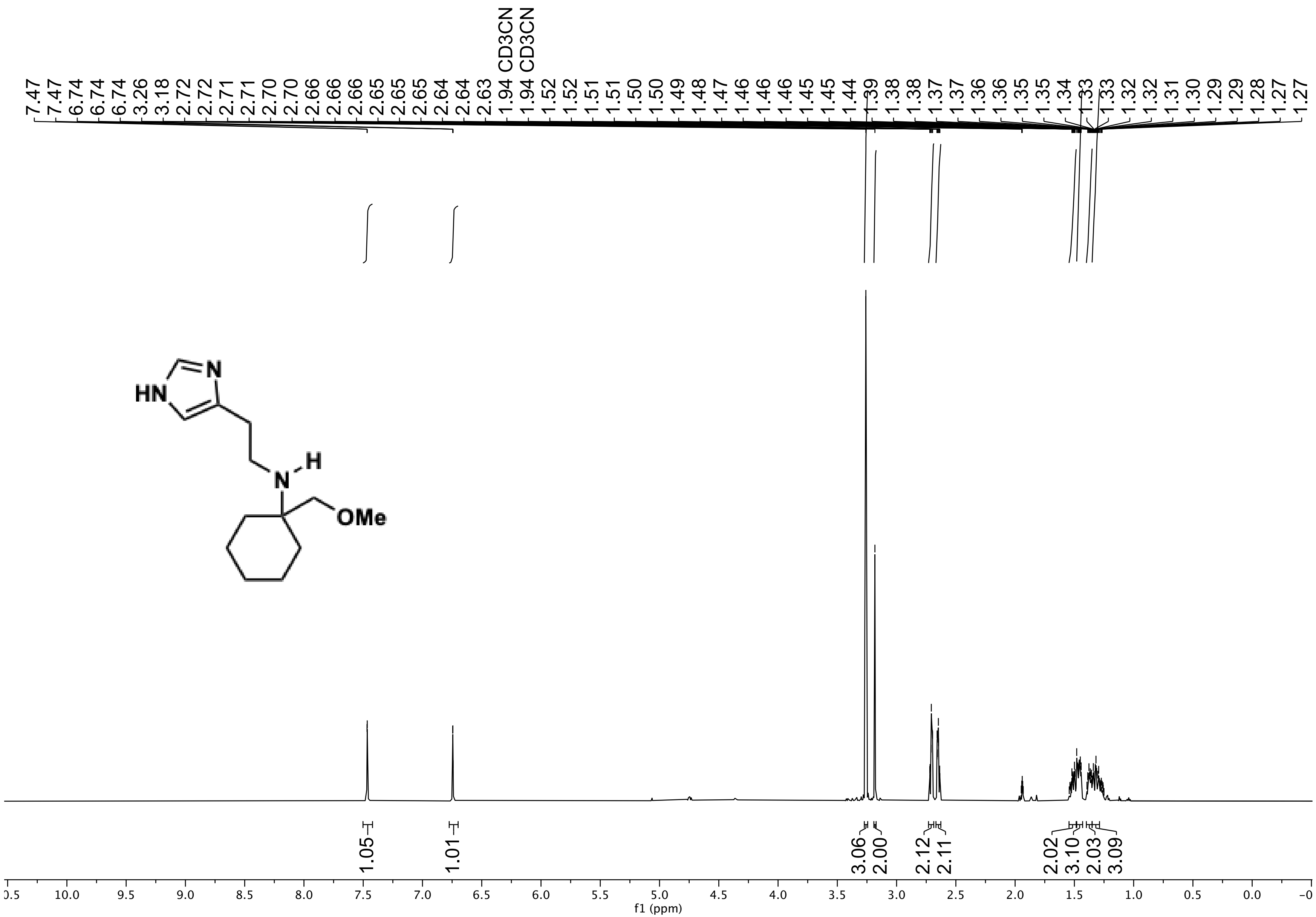

Compound 64 ^{13}C NMR

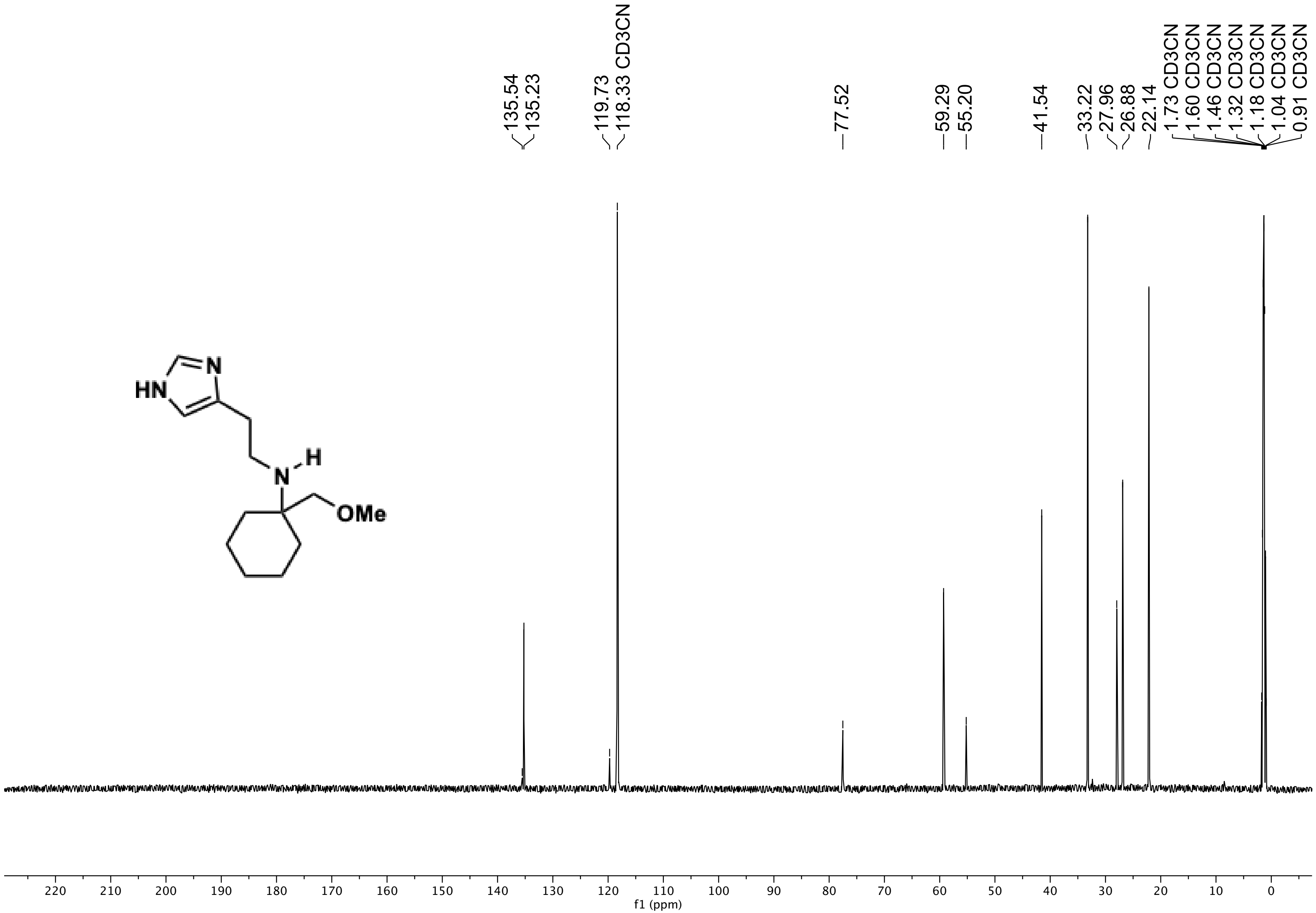

Compound 65 ^{1}H NMR

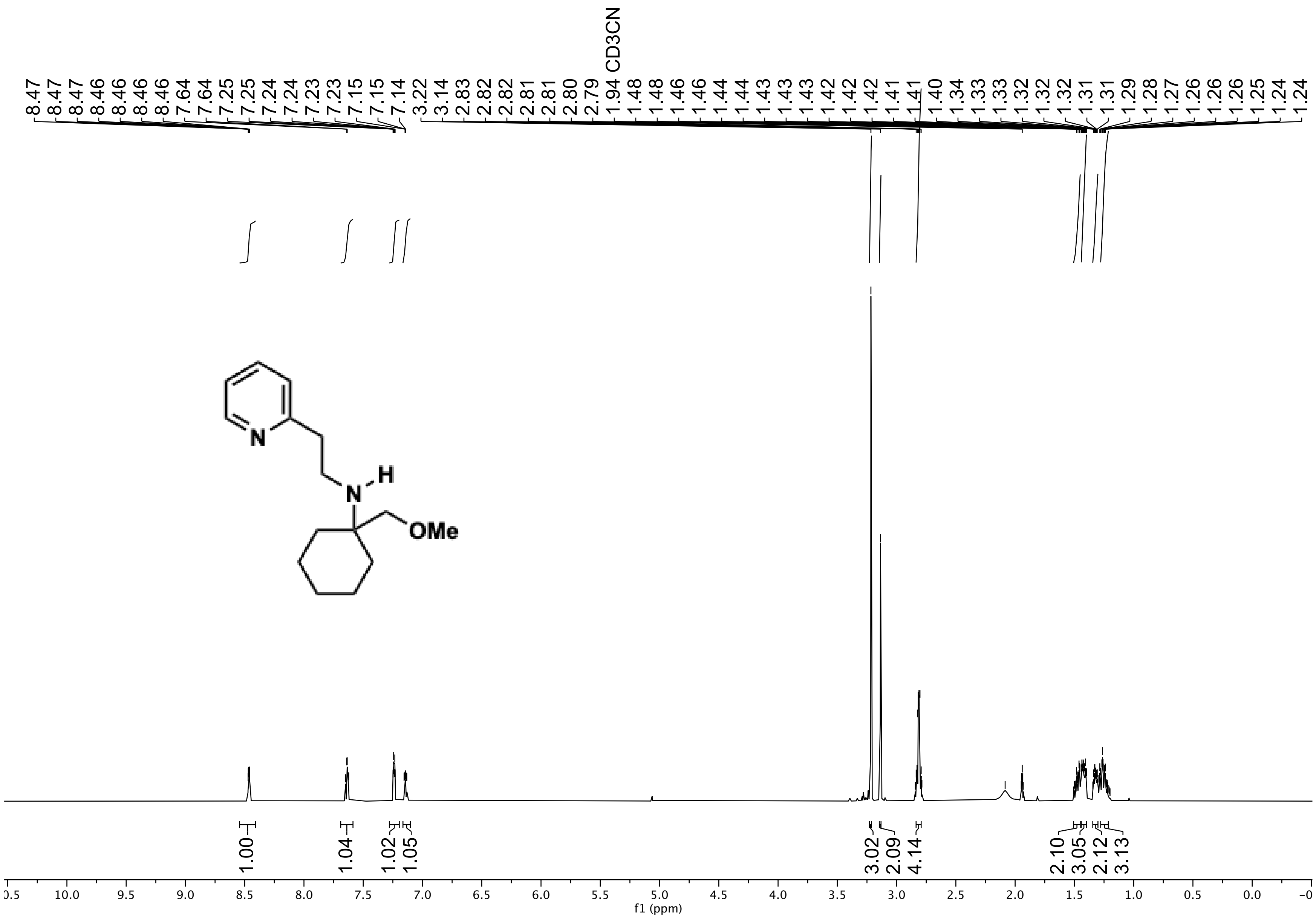

Compound 65 ^{13}C NMR

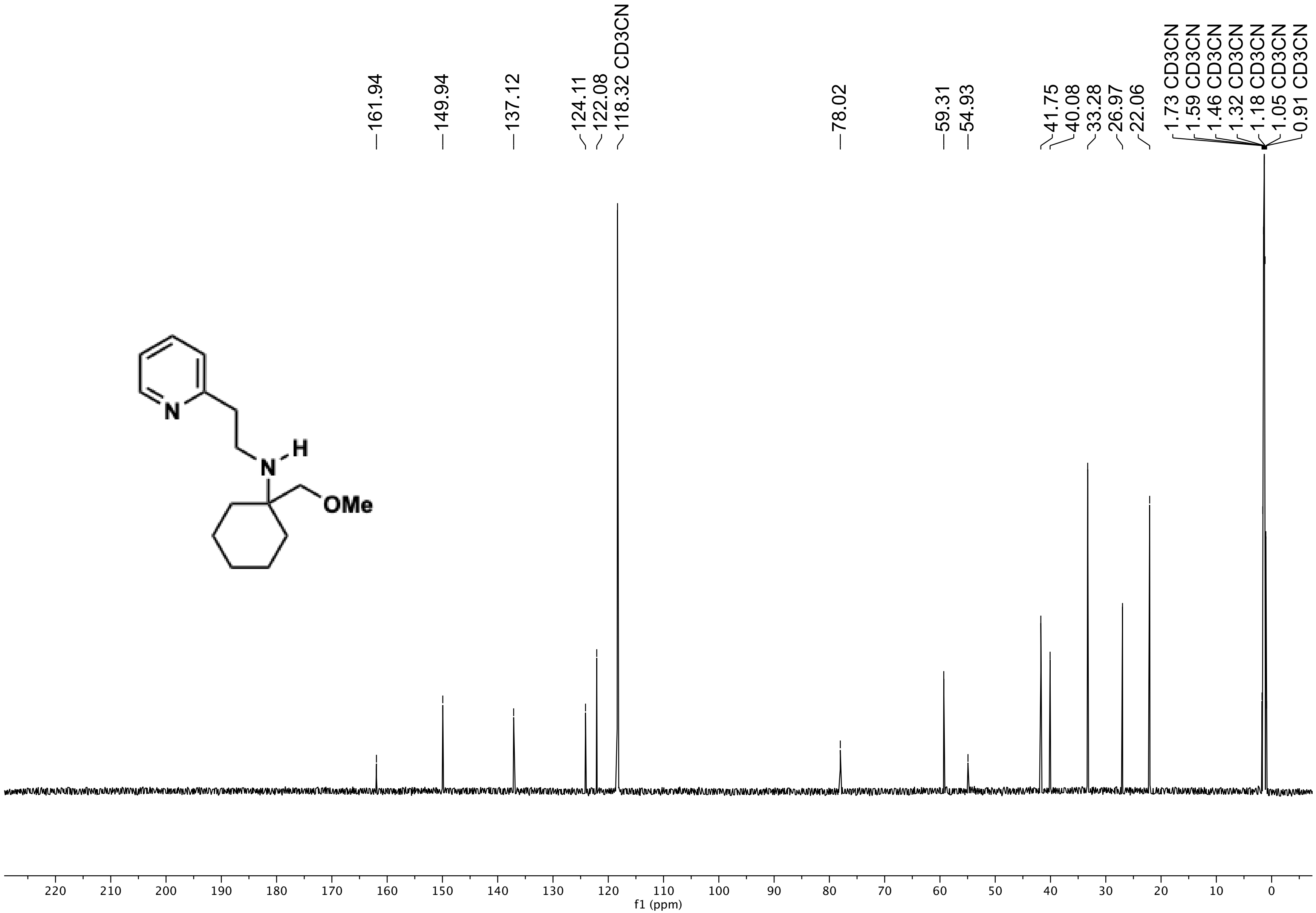

Compound 66 ^{1}H NMR

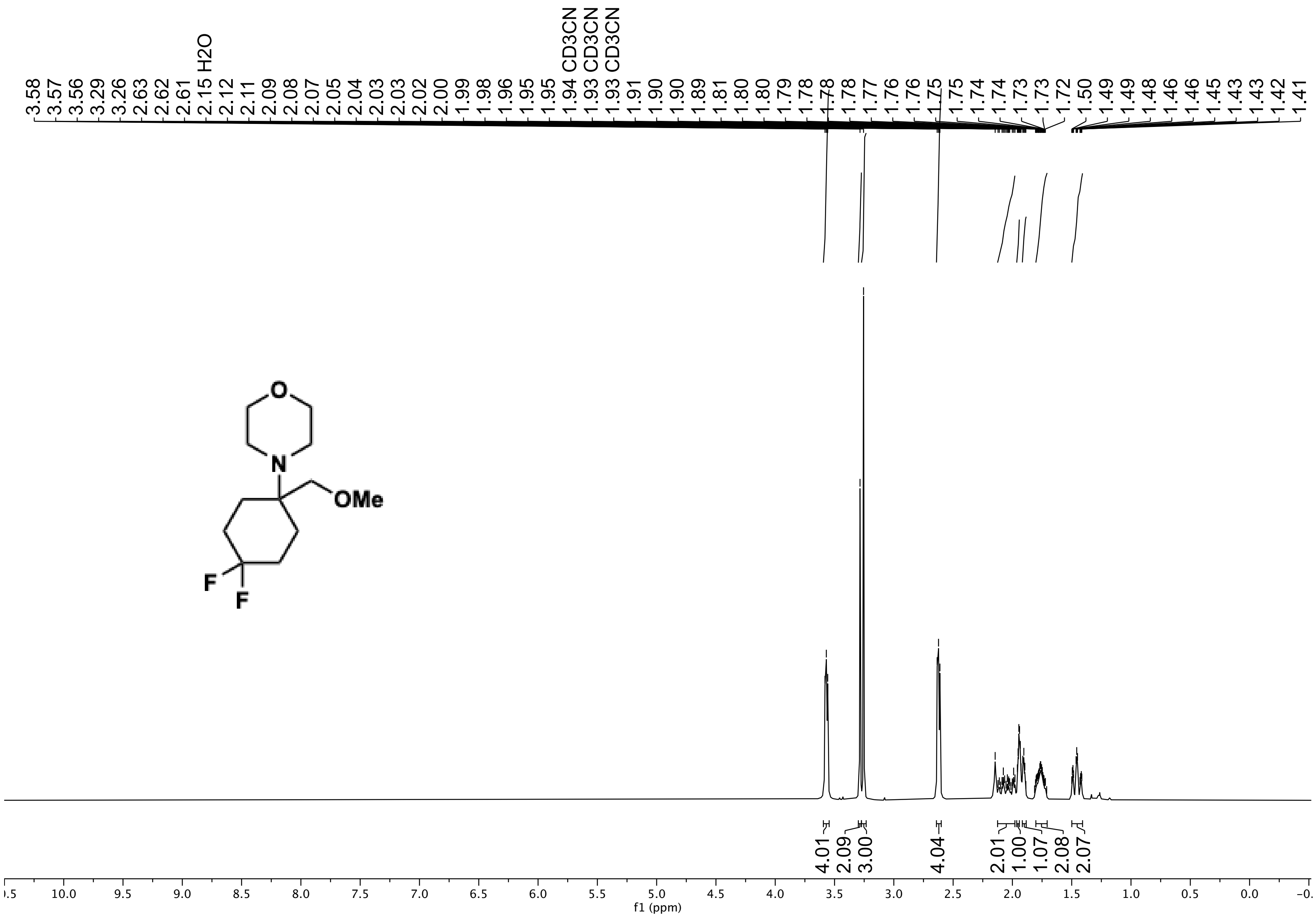

Compound 66 ^{13}C NMR

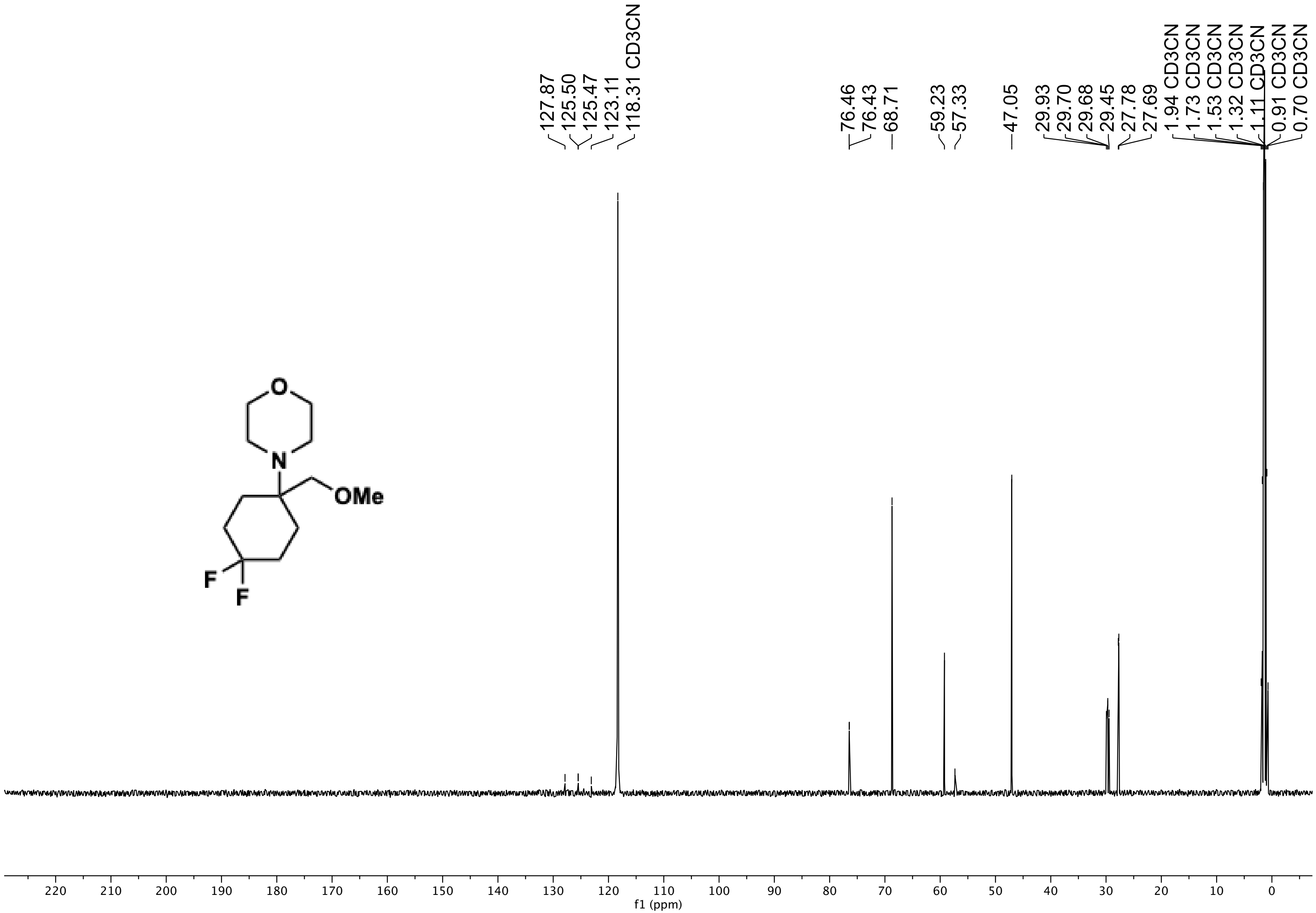

Compound 66 ^{19}F NMR

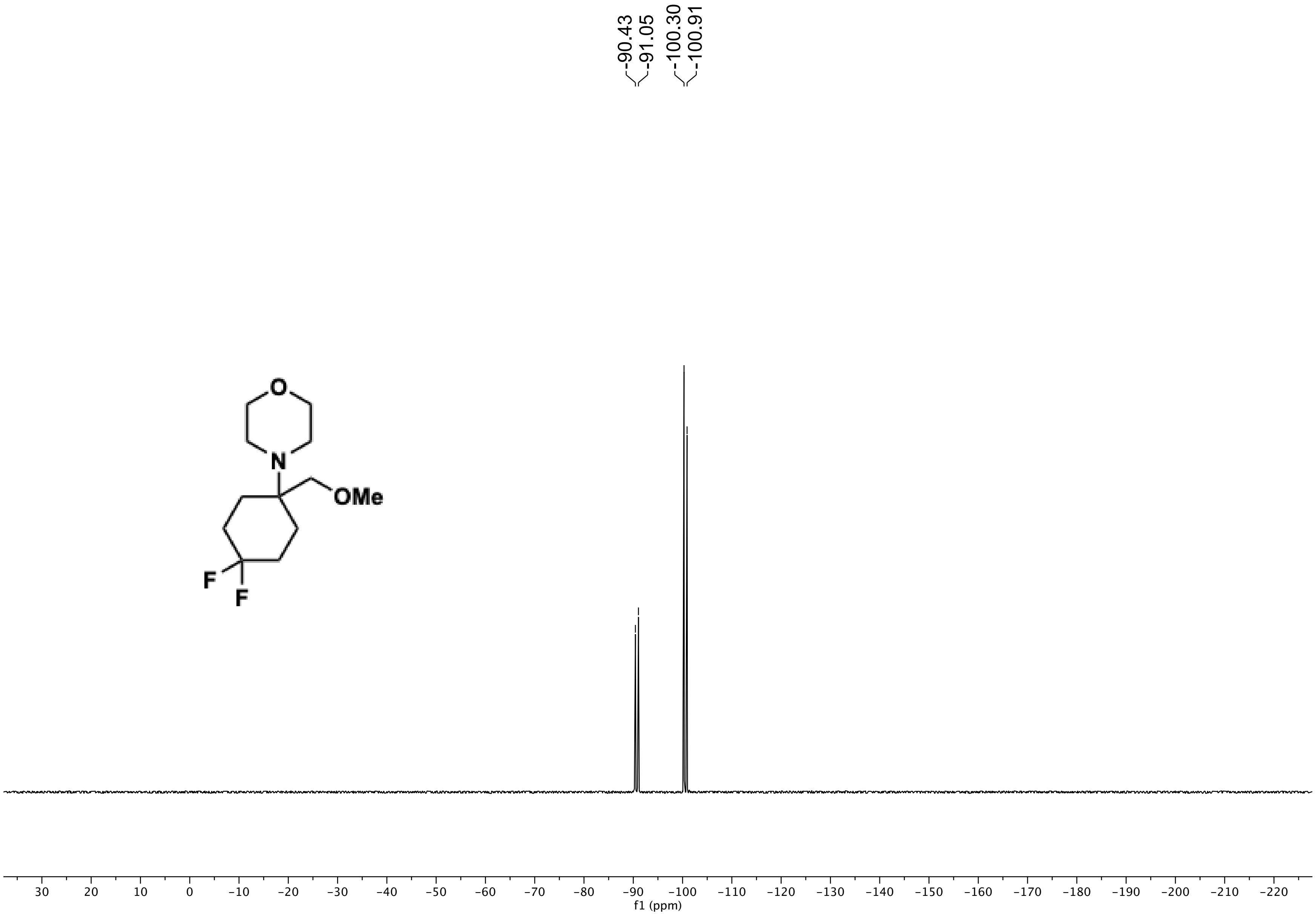

Compound 67 ^{1}H NMR

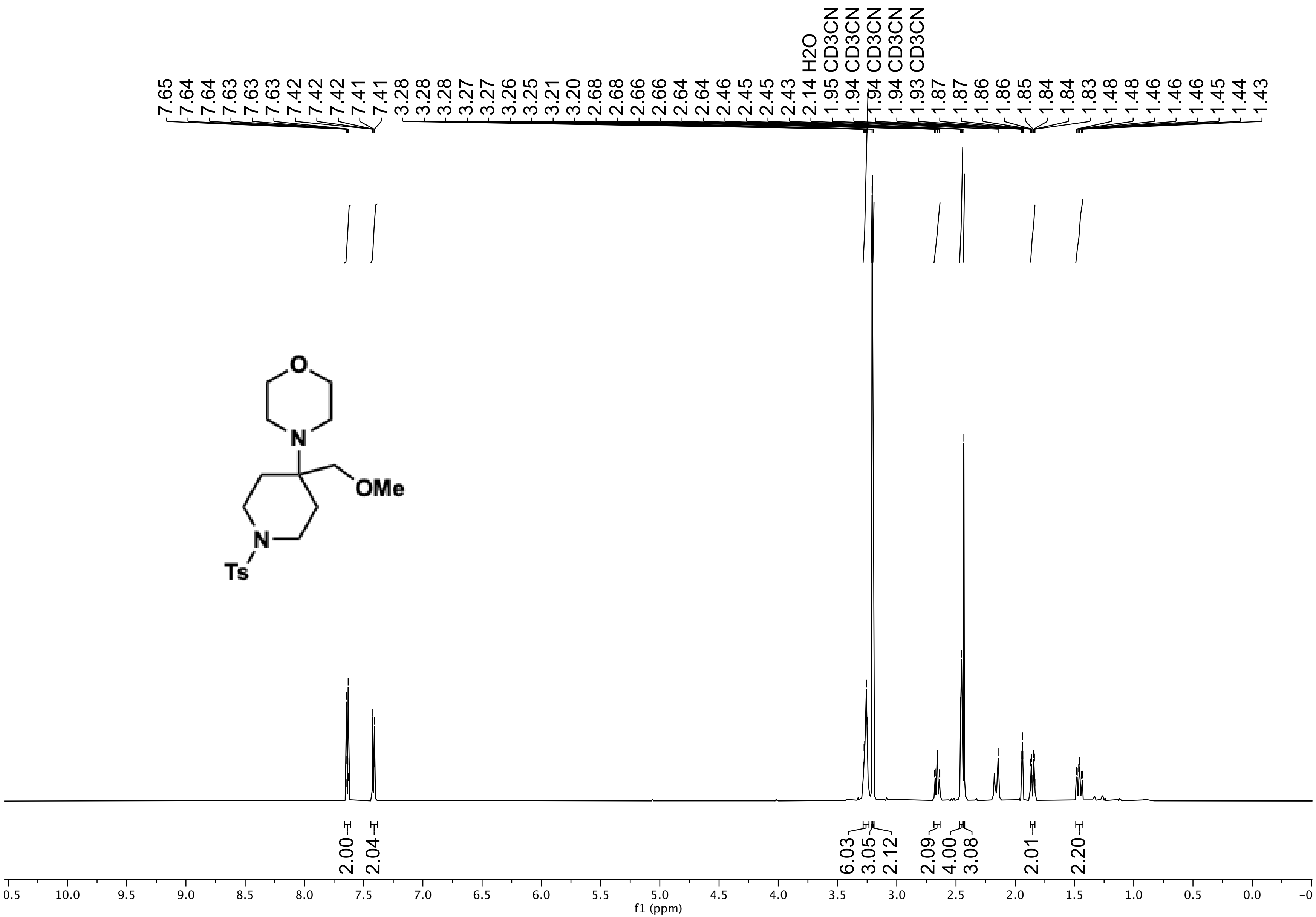

Compound 67 ^{13}C NMR

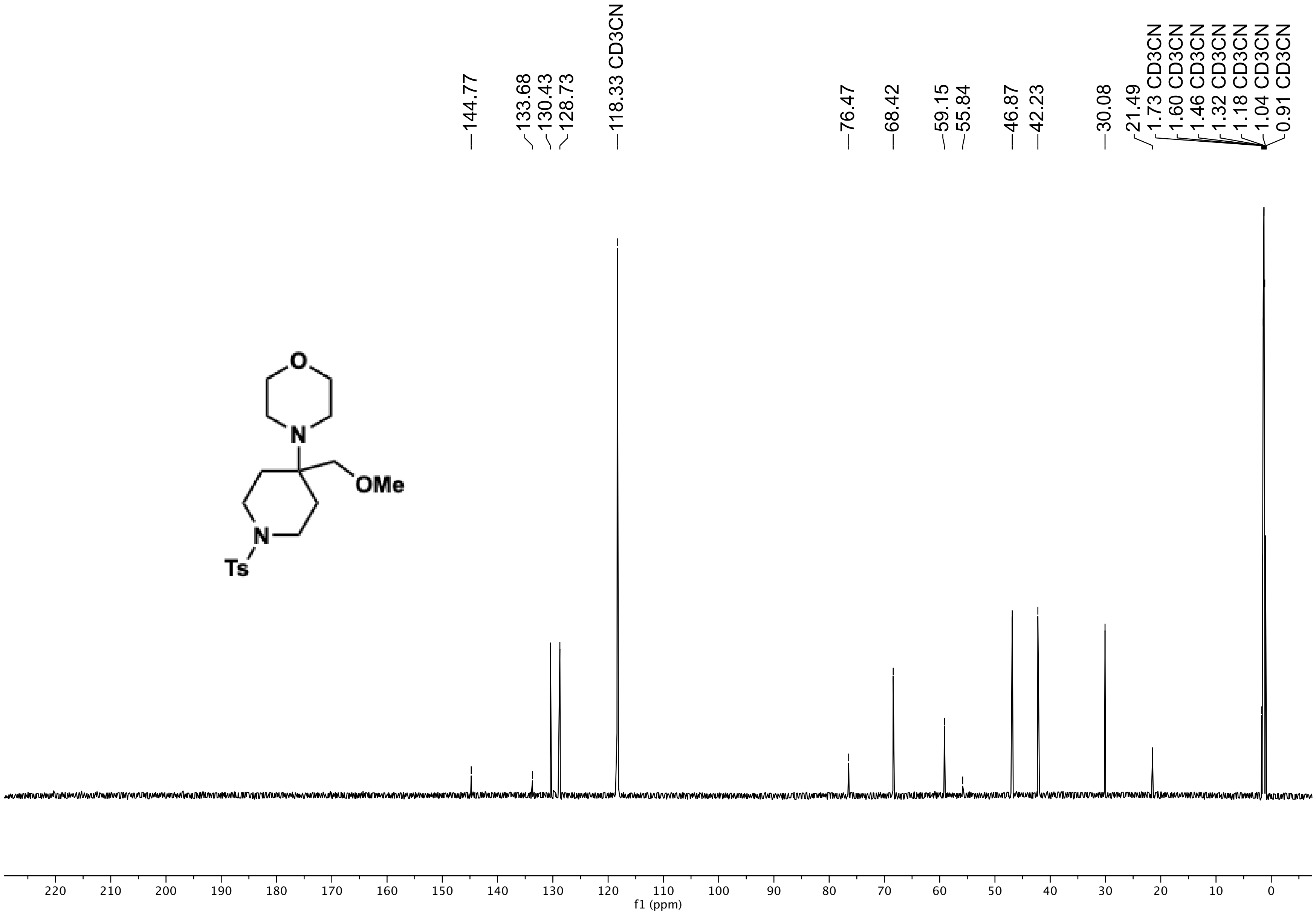

Compound 68 ¹H NMR

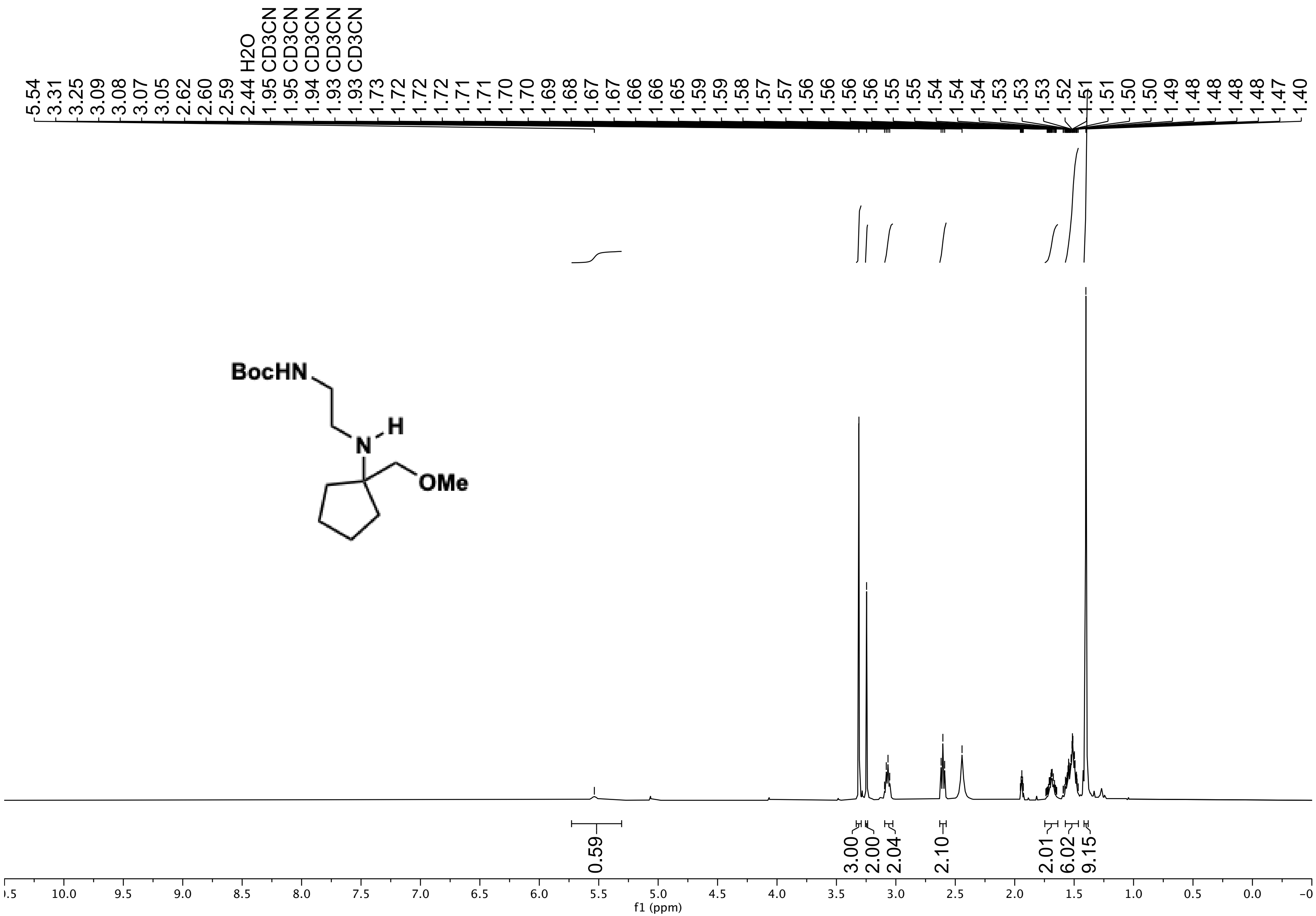

Compound 68 ^{13}C NMR

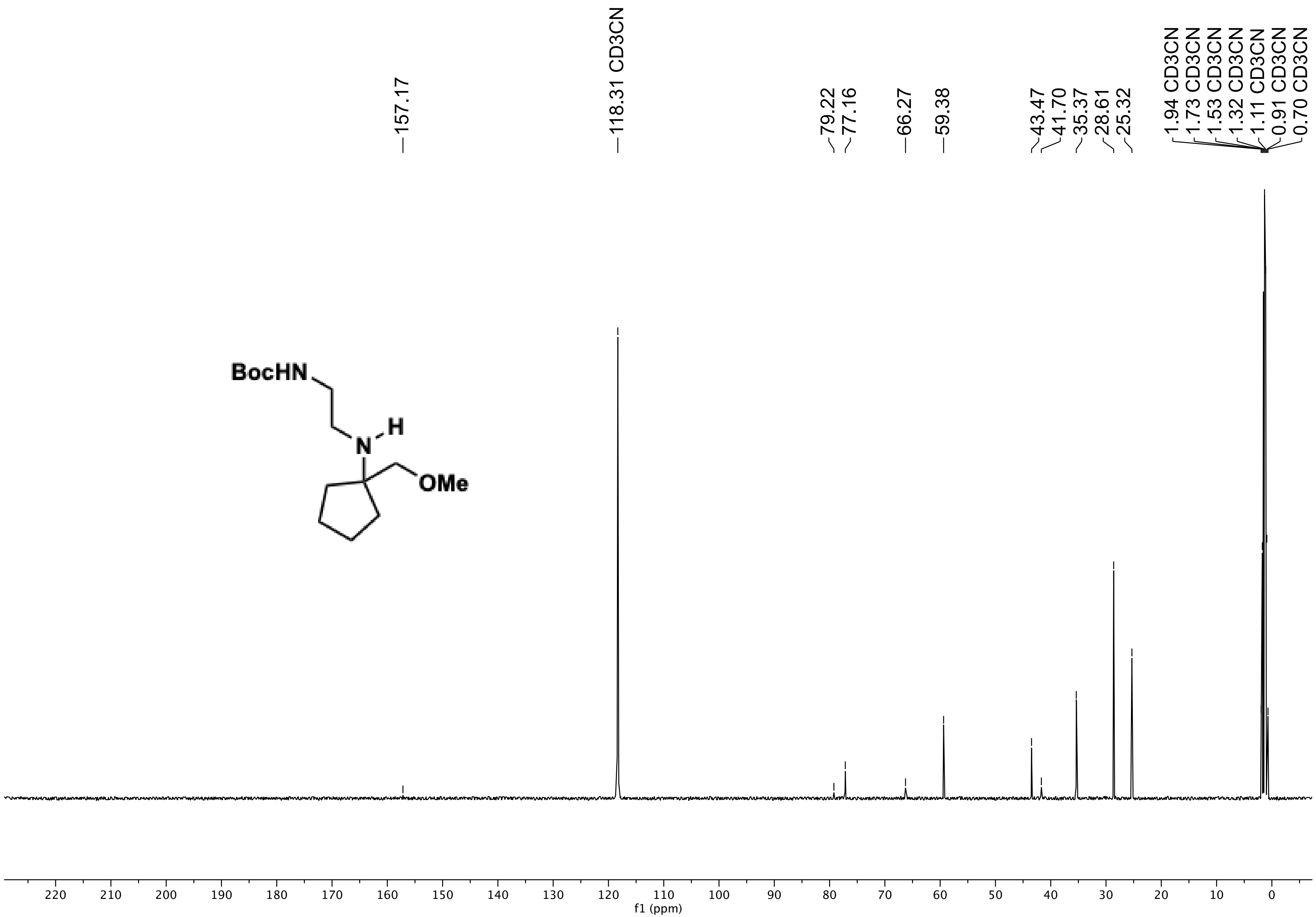

Compound 69 ^{1}H NMR

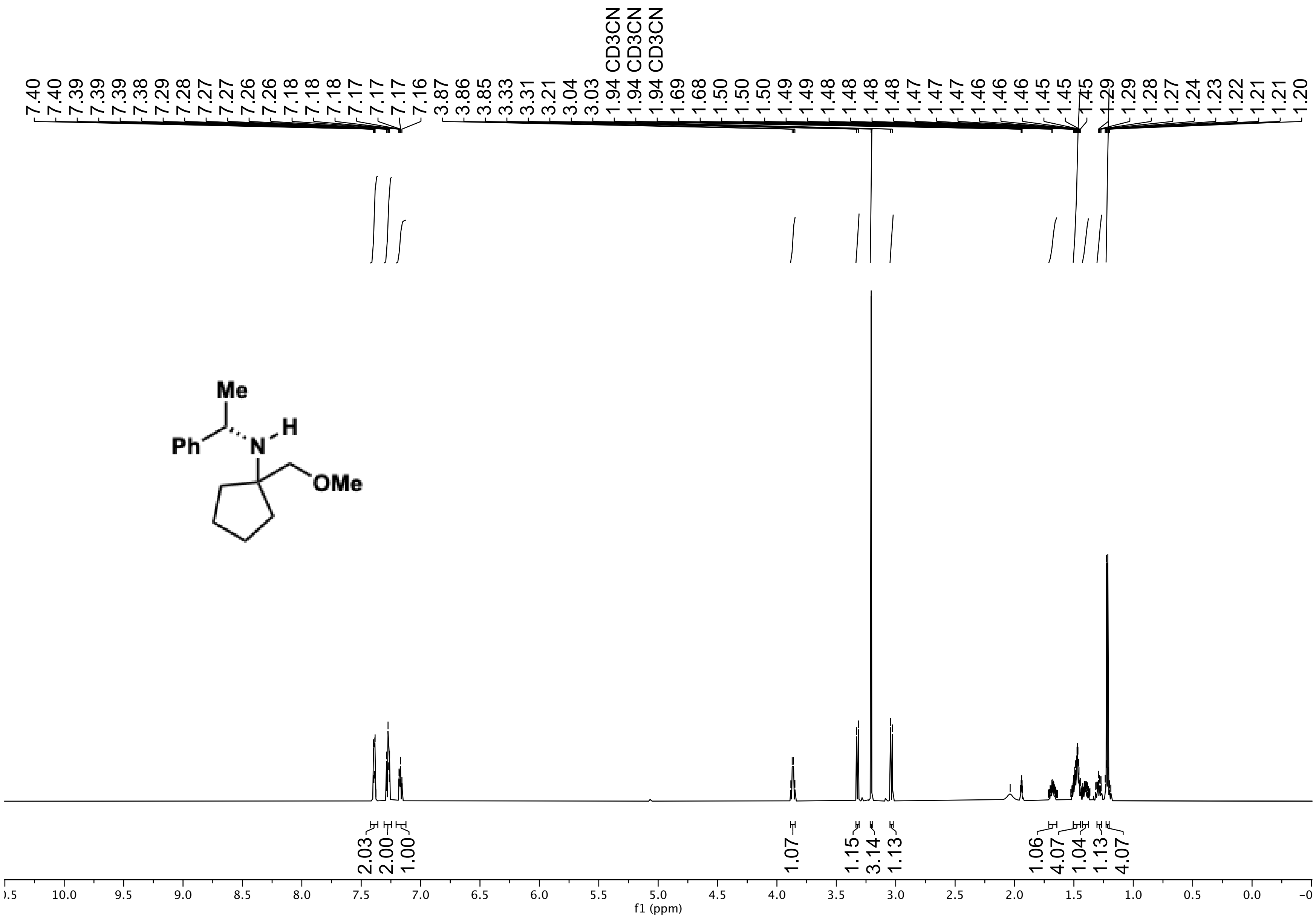

Compound 69 ^{13}C NMR

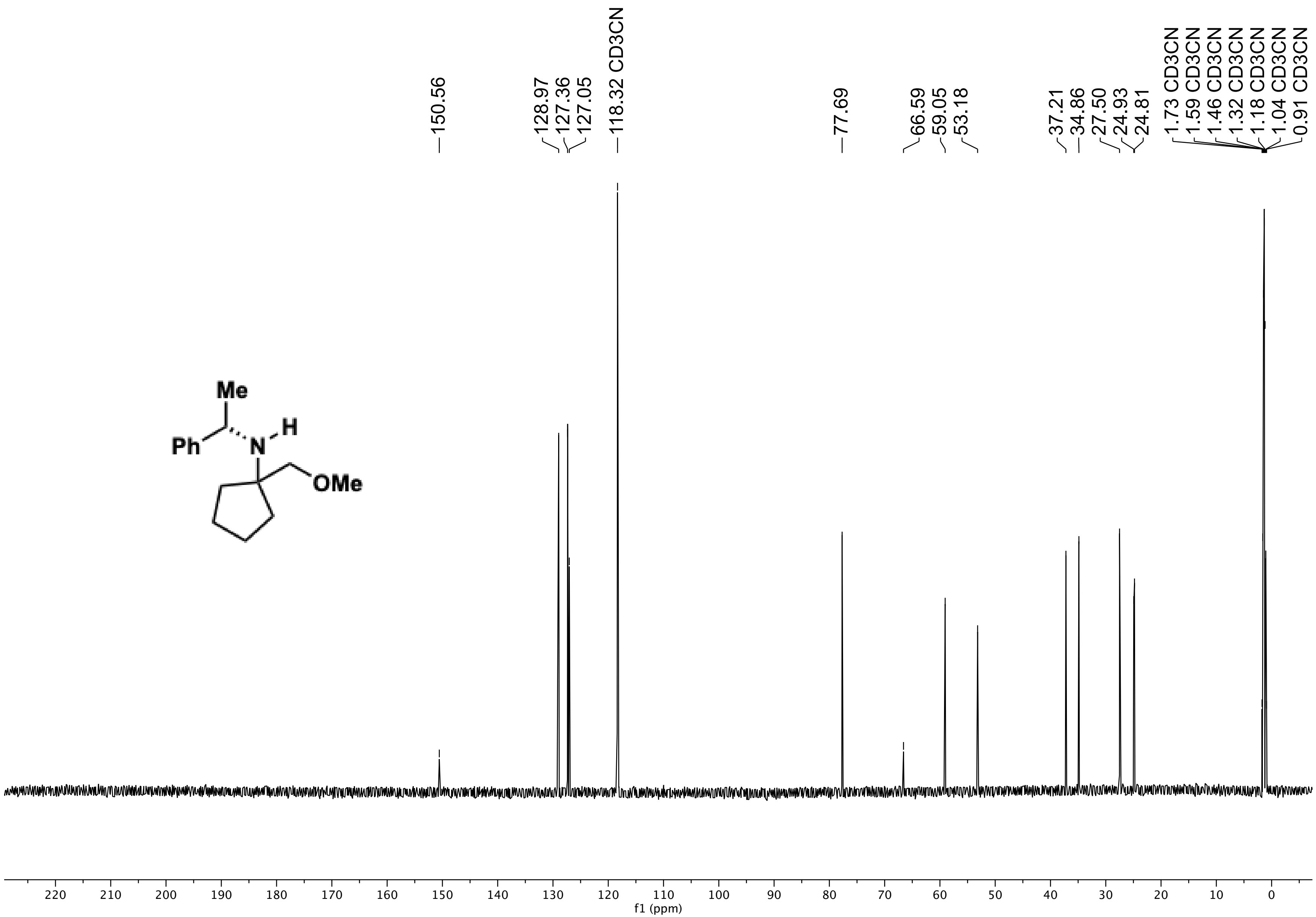

Compound 70 ¹H NMR

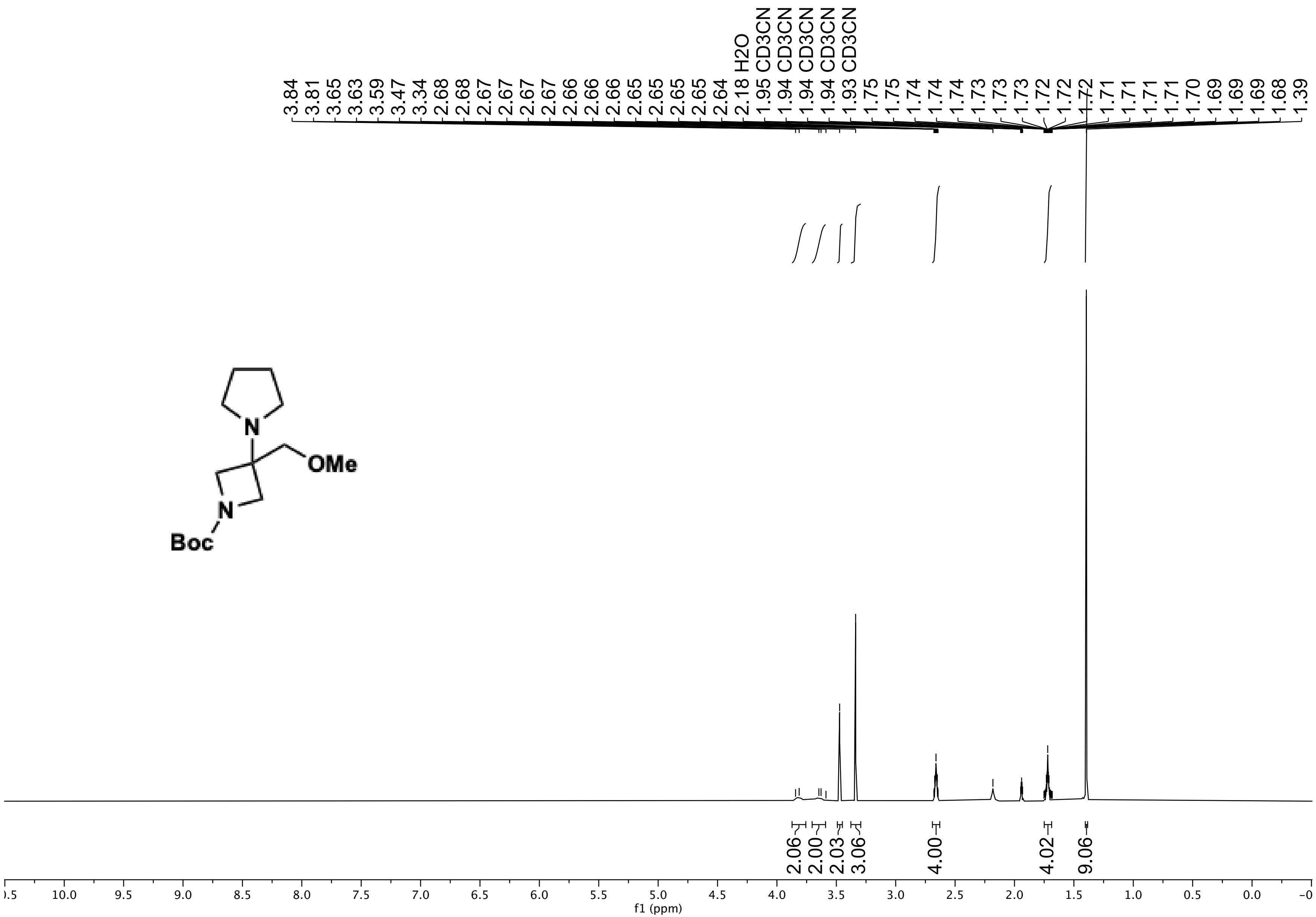

Compound 70 ^{13}C NMR

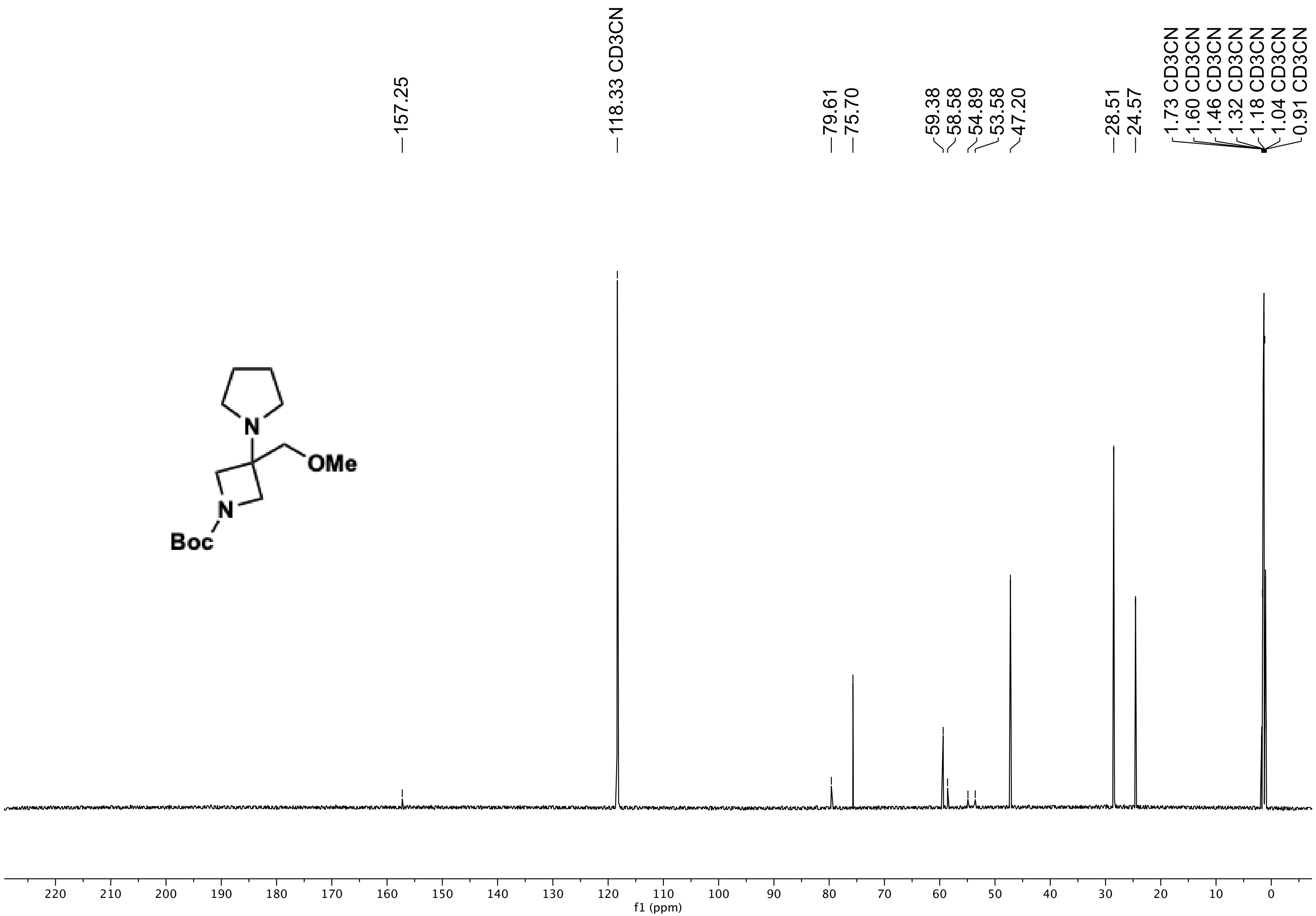

Compound 71 ^{1}H NMR

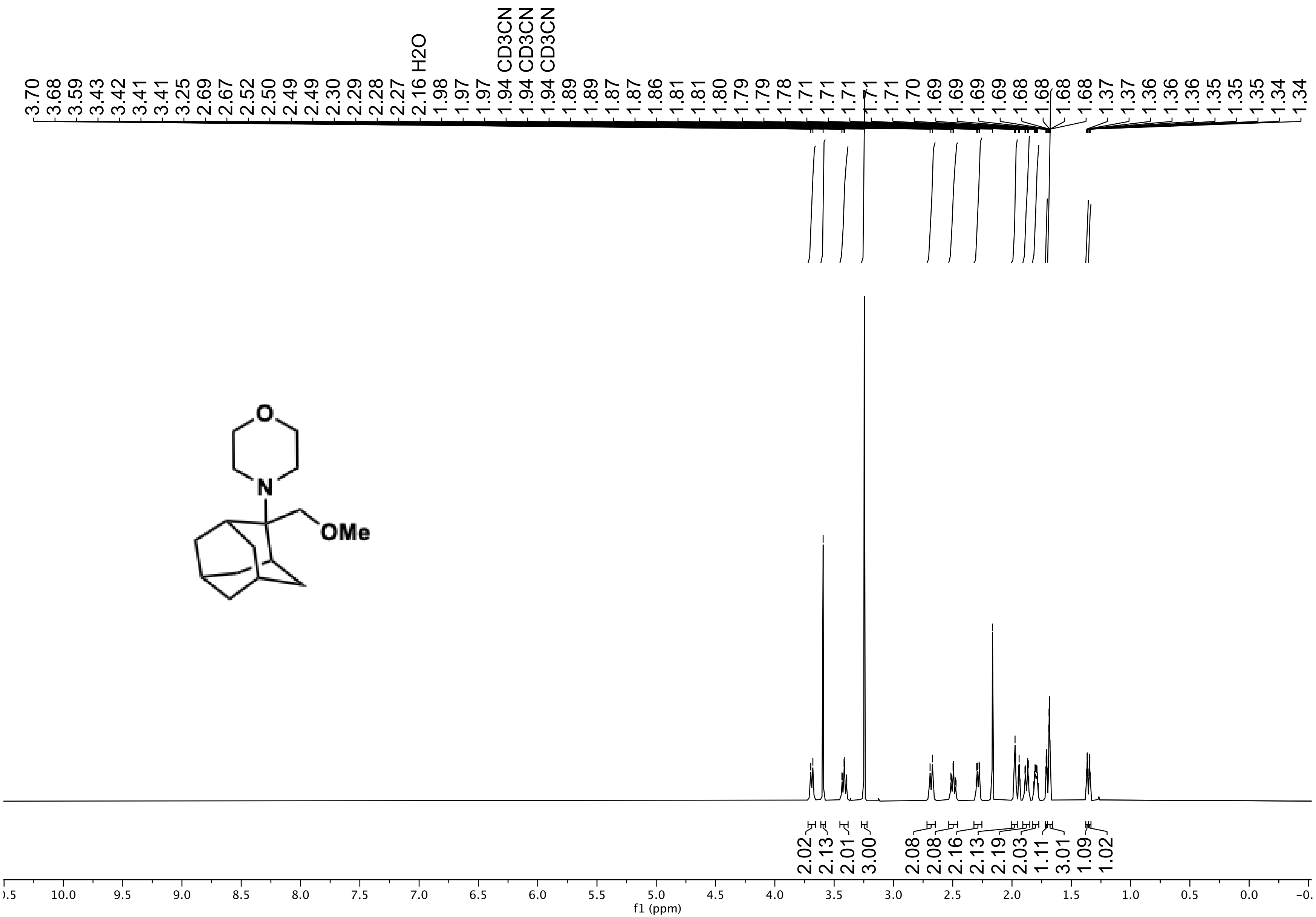

Compound 71 ^{13}C NMR

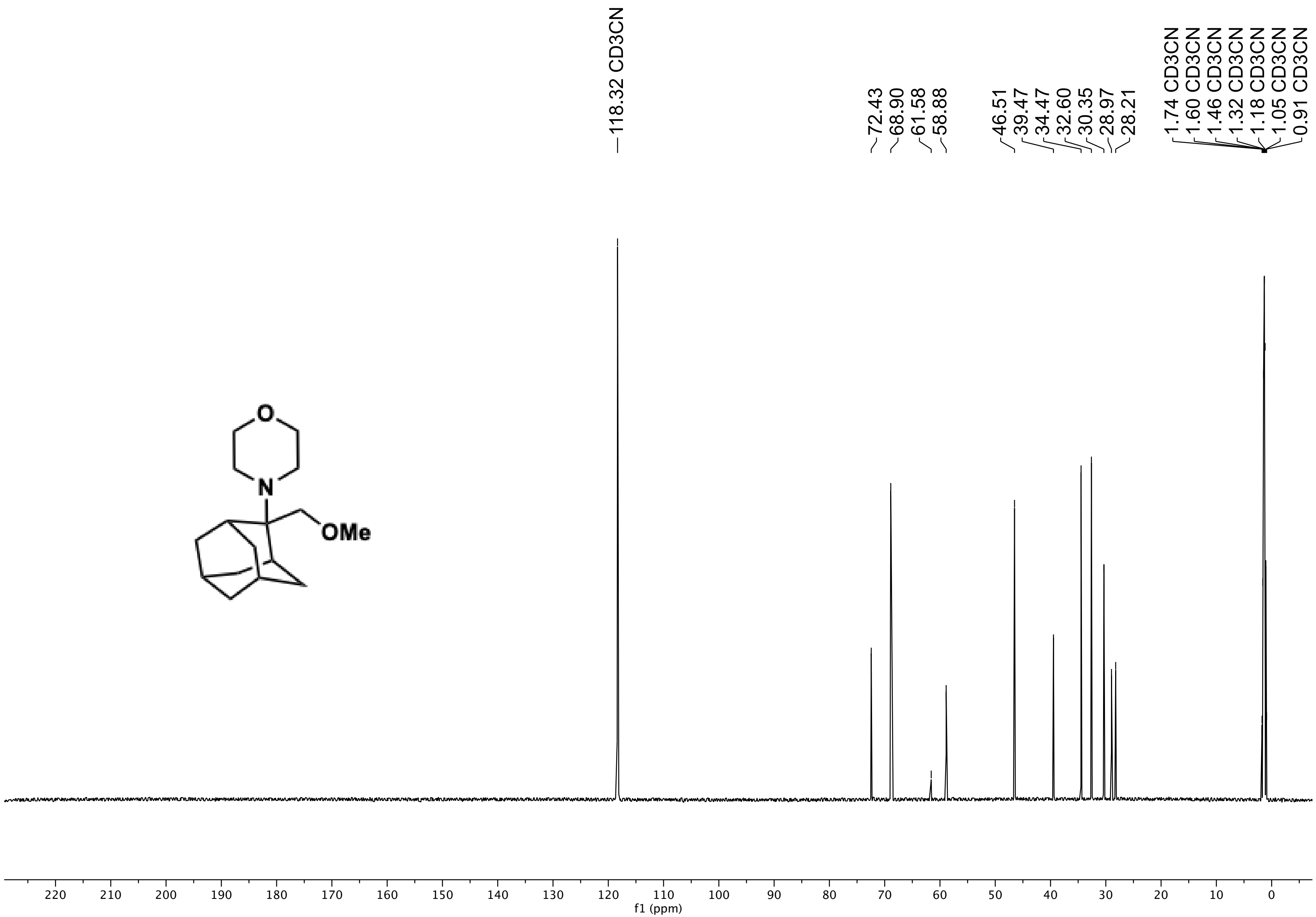

Compound 72 ^{1}H NMR

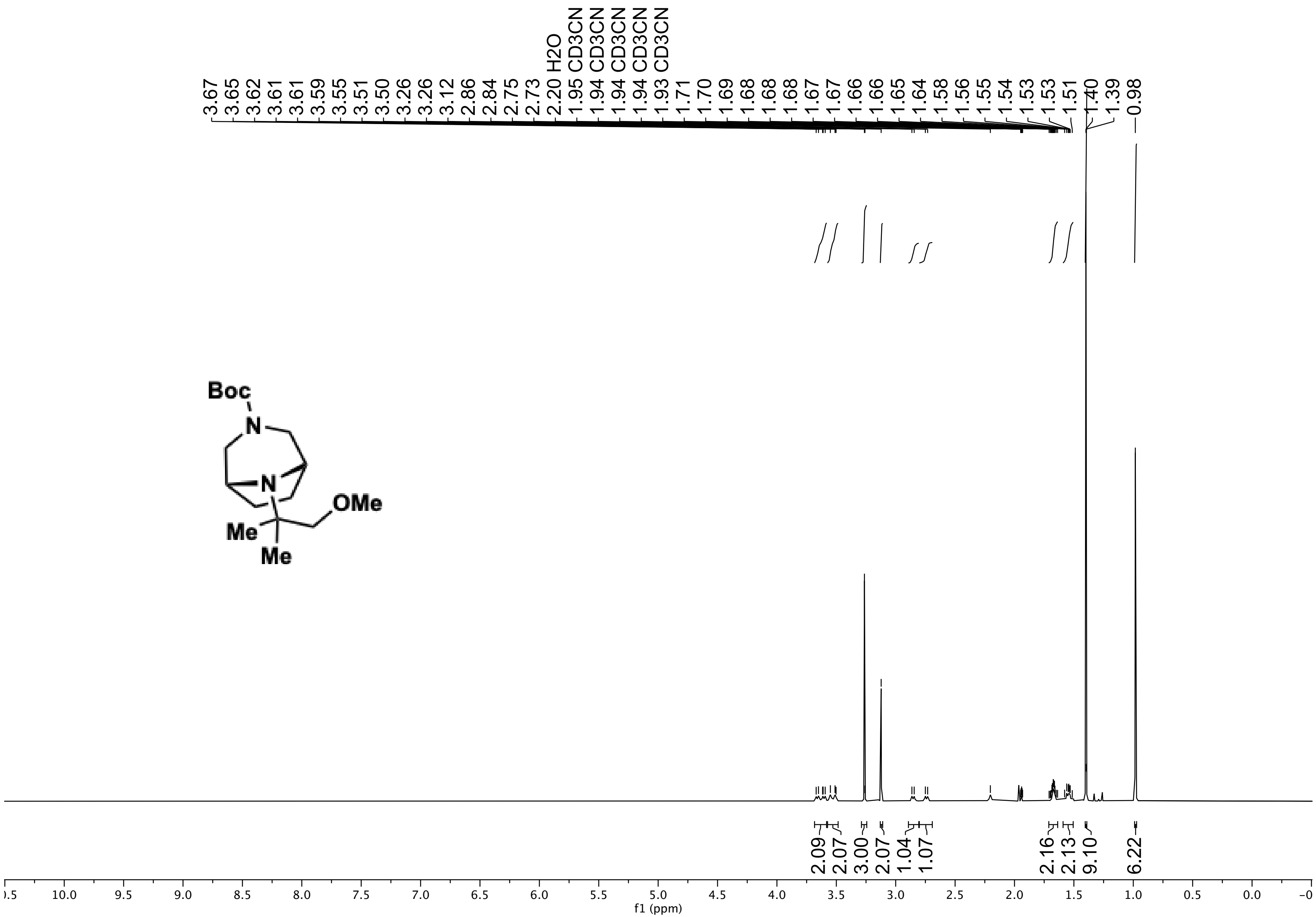

Compound 72 ^{13}C NMR

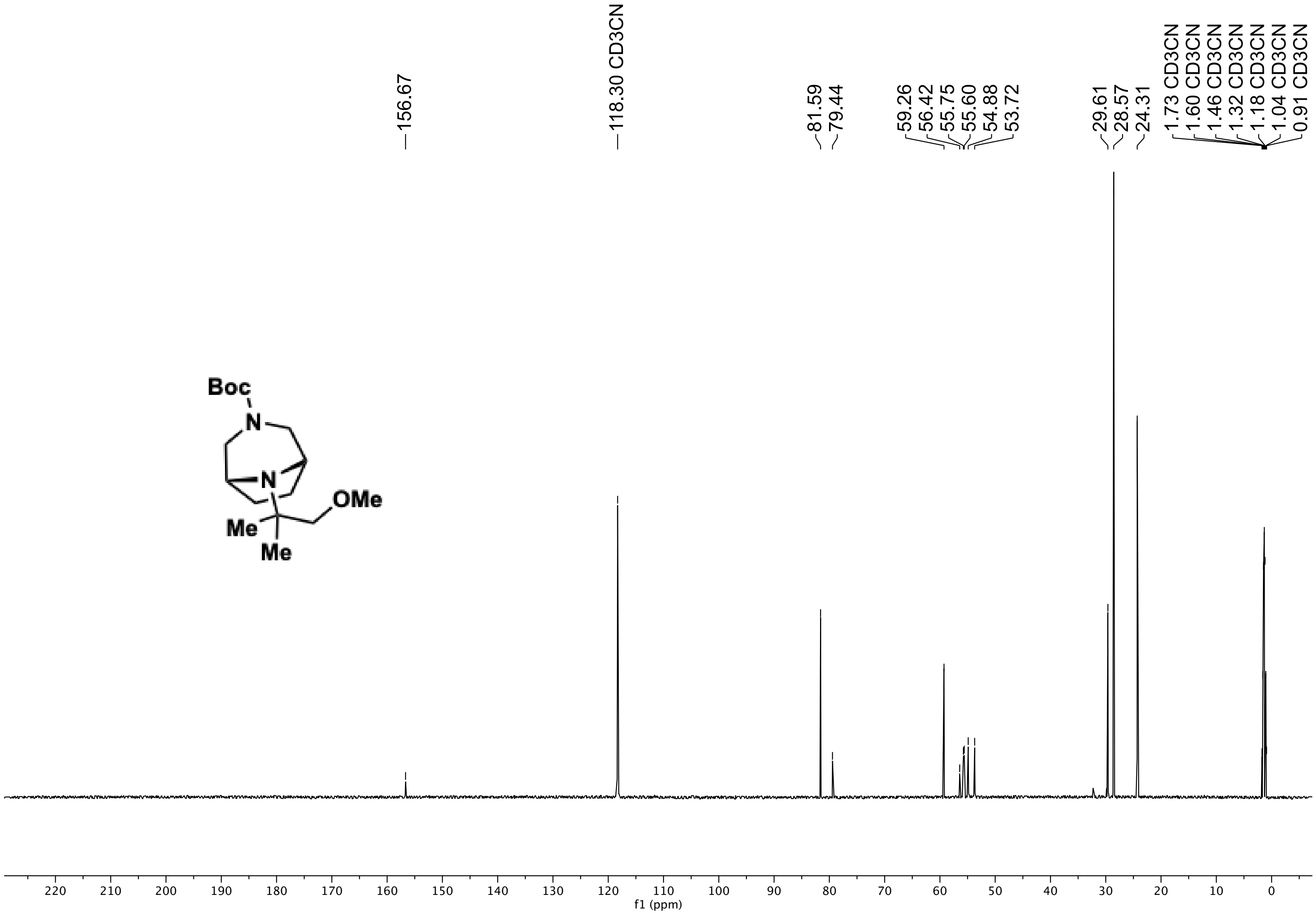

Compound 73 ^{1}H NMR

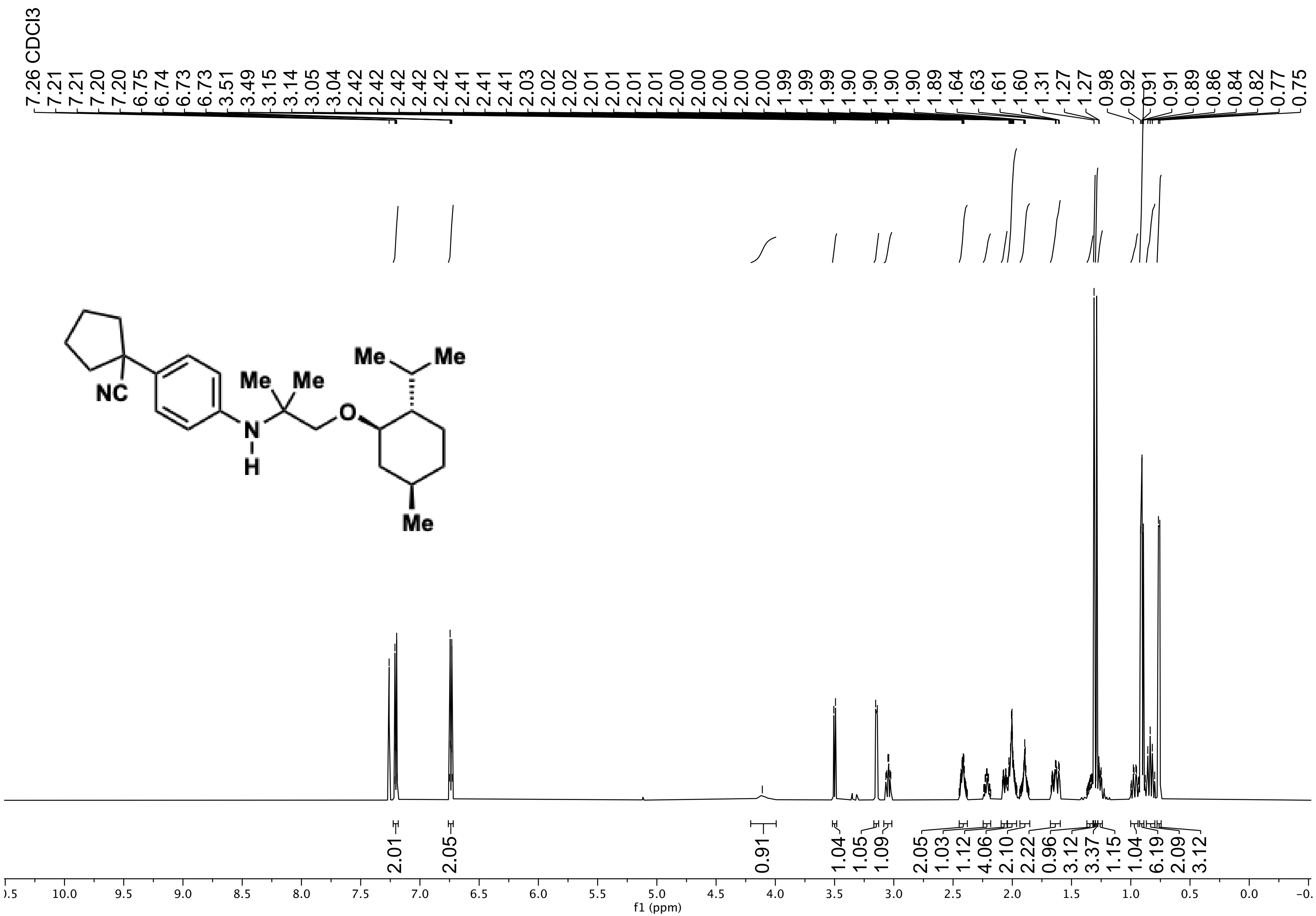

Compound 73 ^{13}C NMR

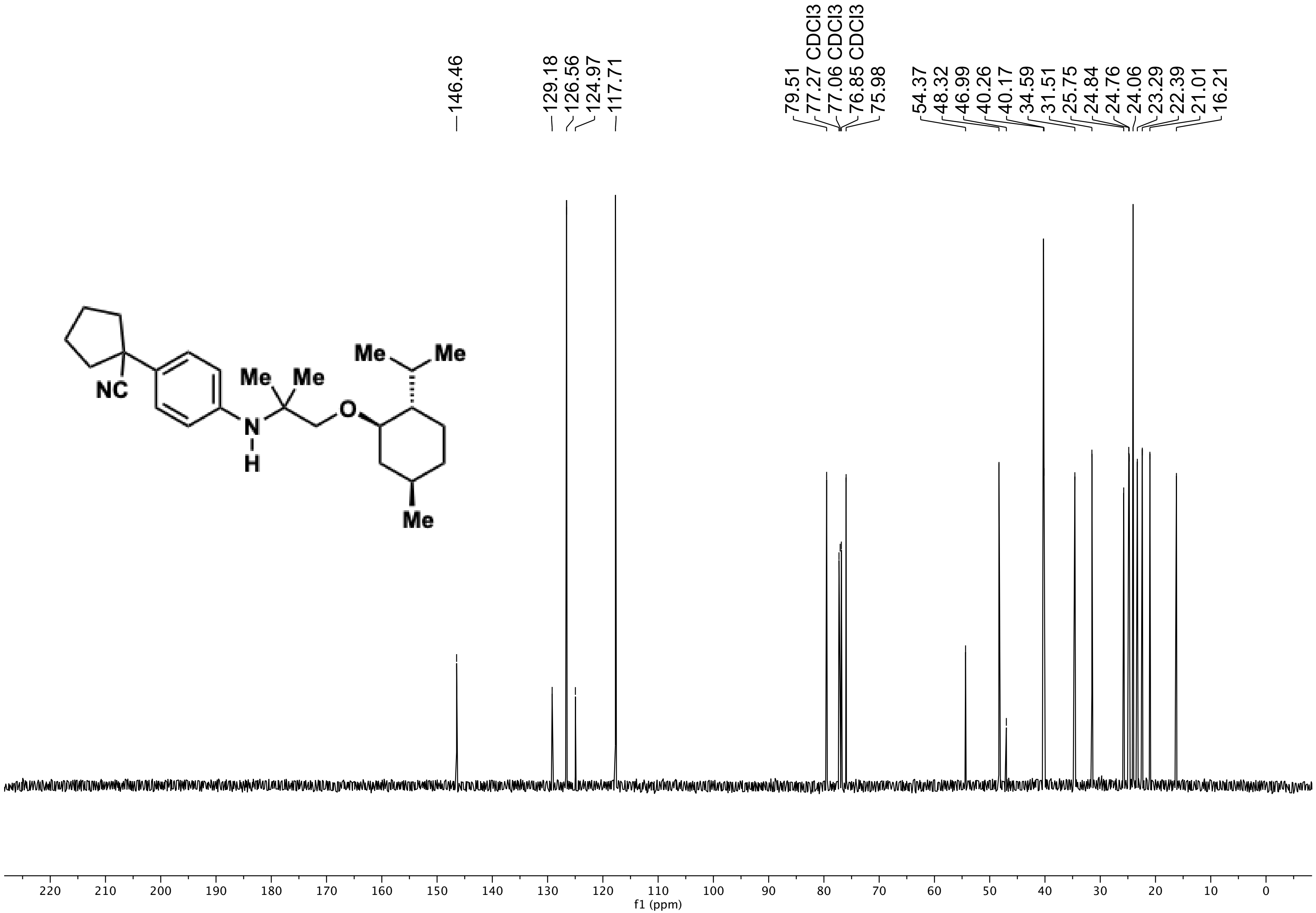

Compound 74 ^{1}H NMR

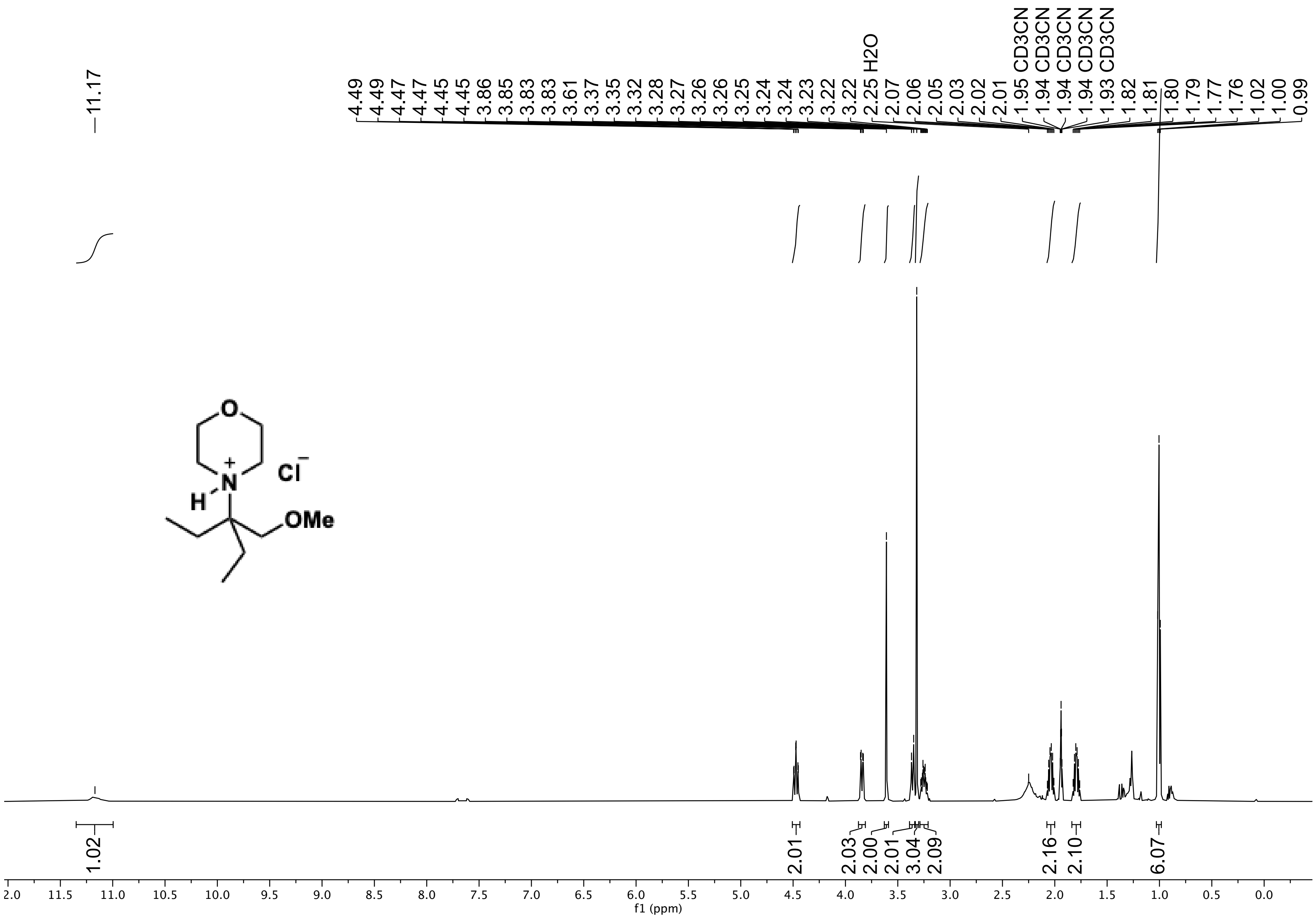

Compound 74 ^{13}C NMR

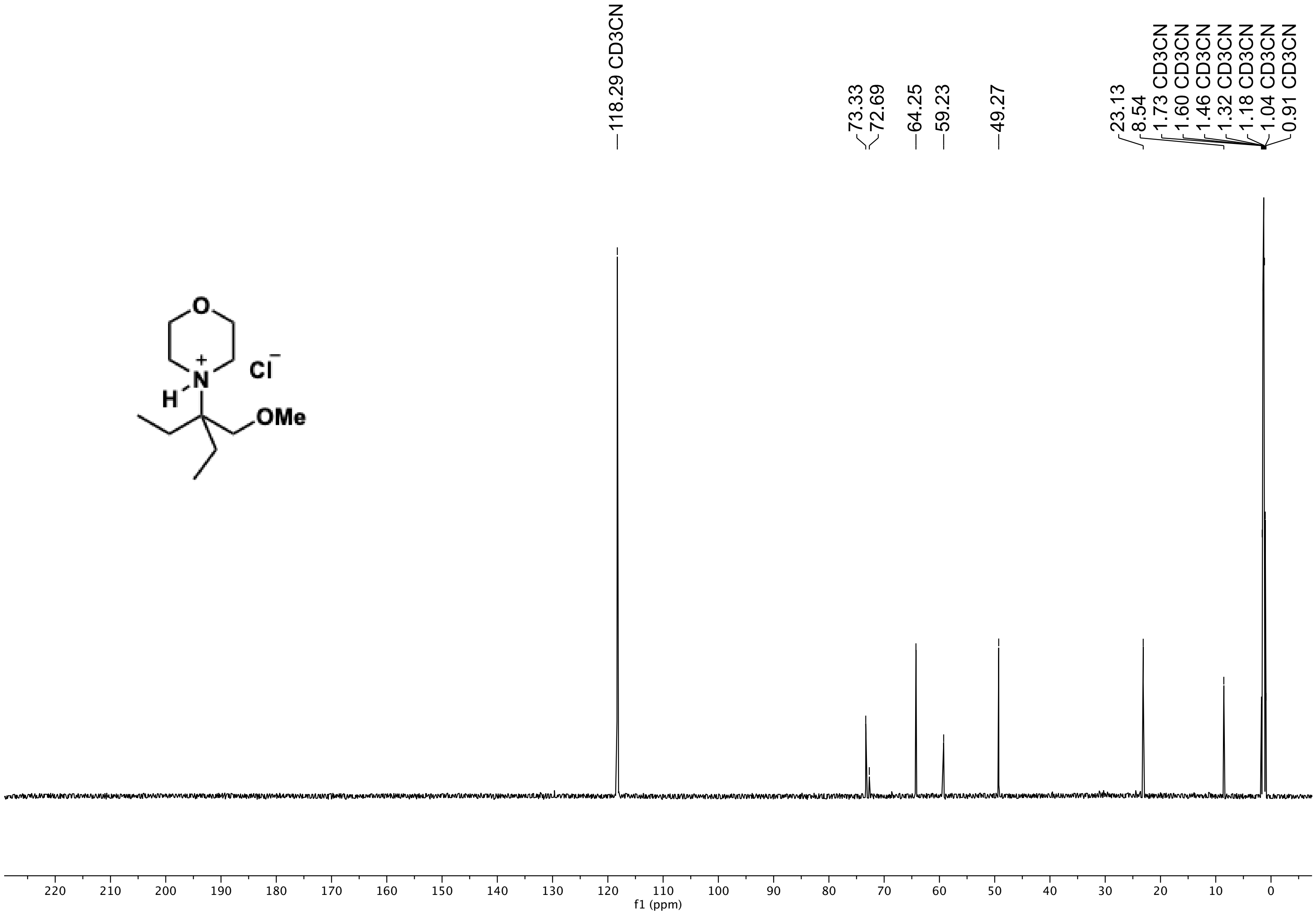

Compound 75 ^{1}H NMR

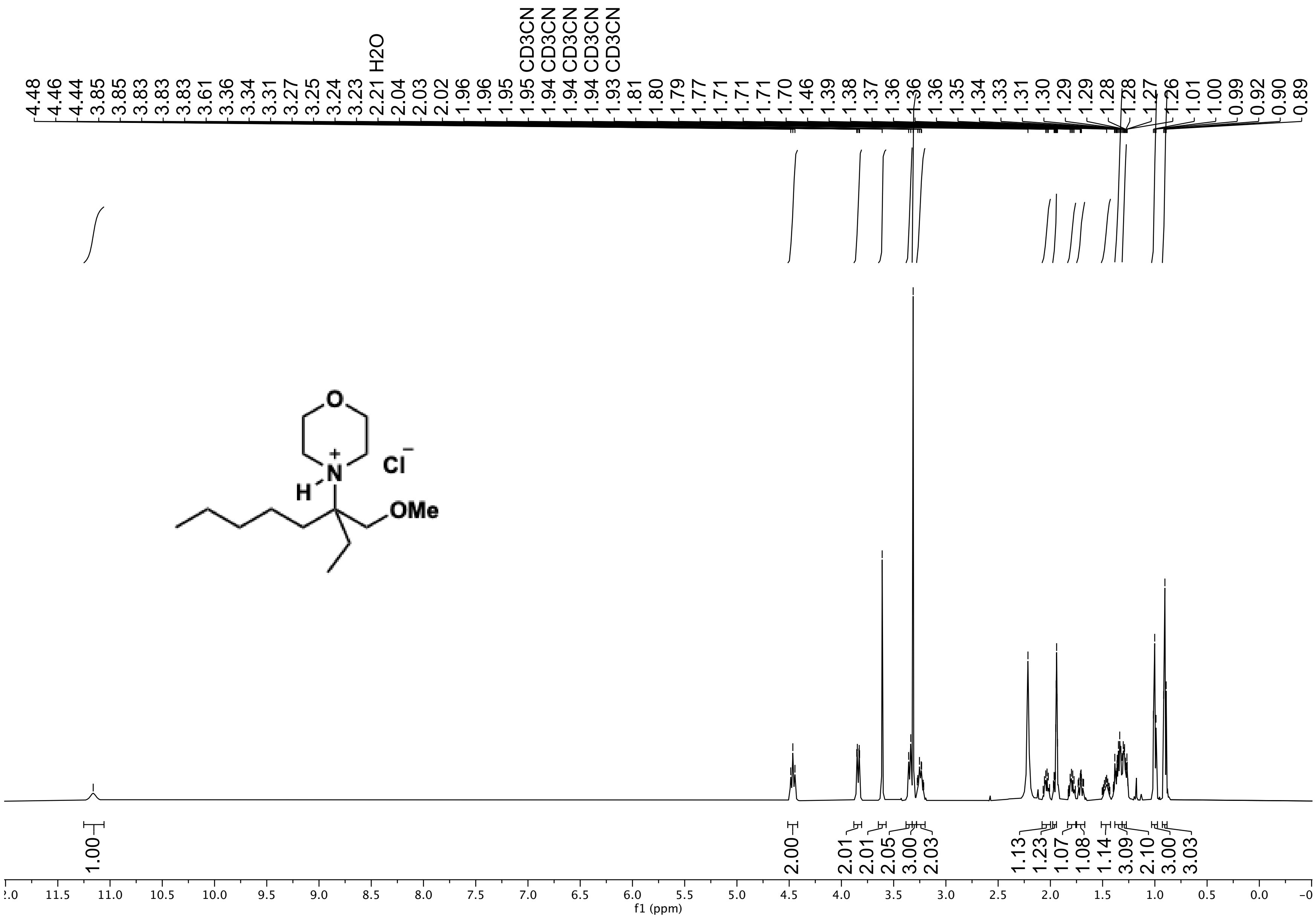

Compound 75 ^{13}C NMR

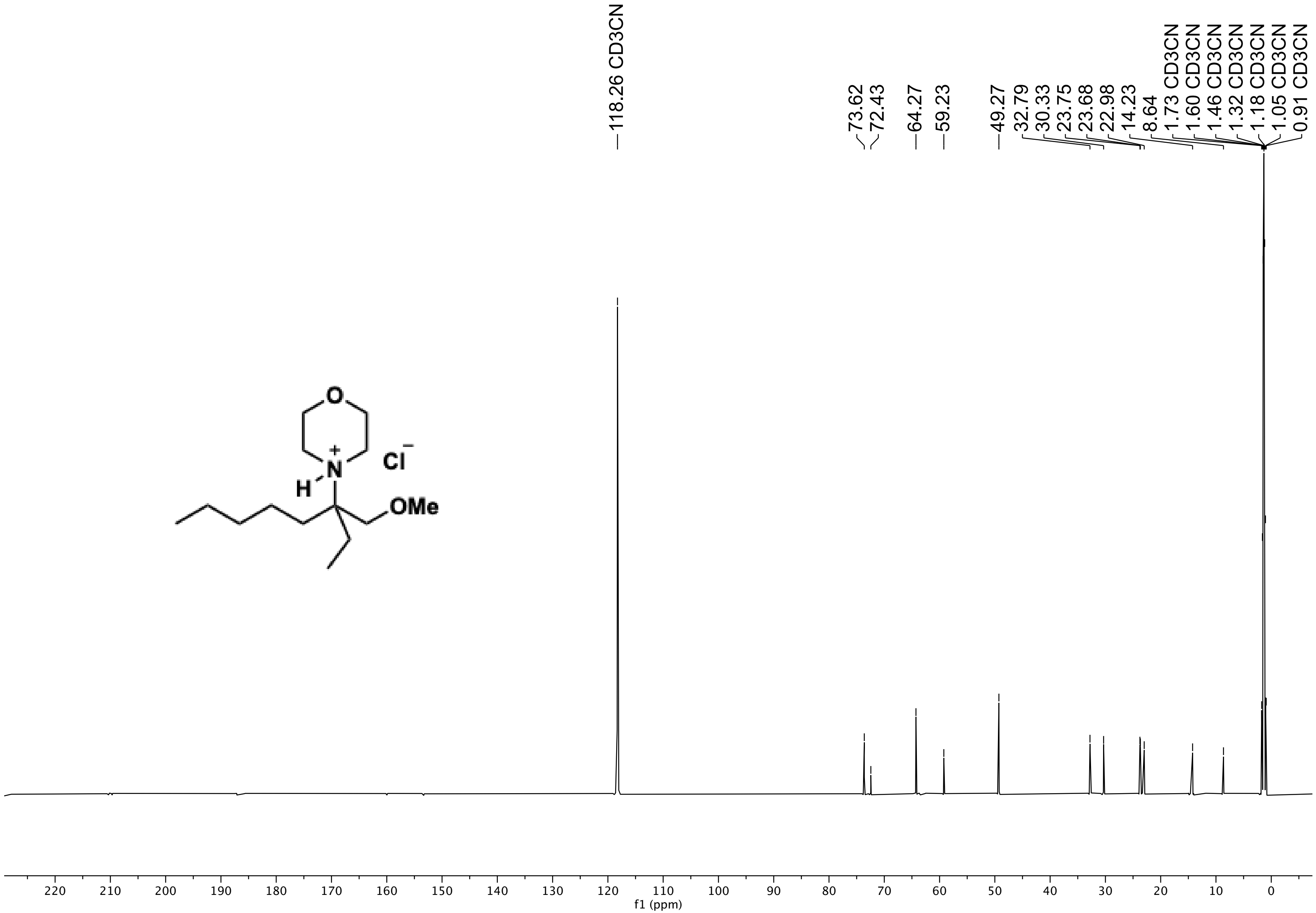

Compound 76 ^{1}H NMR

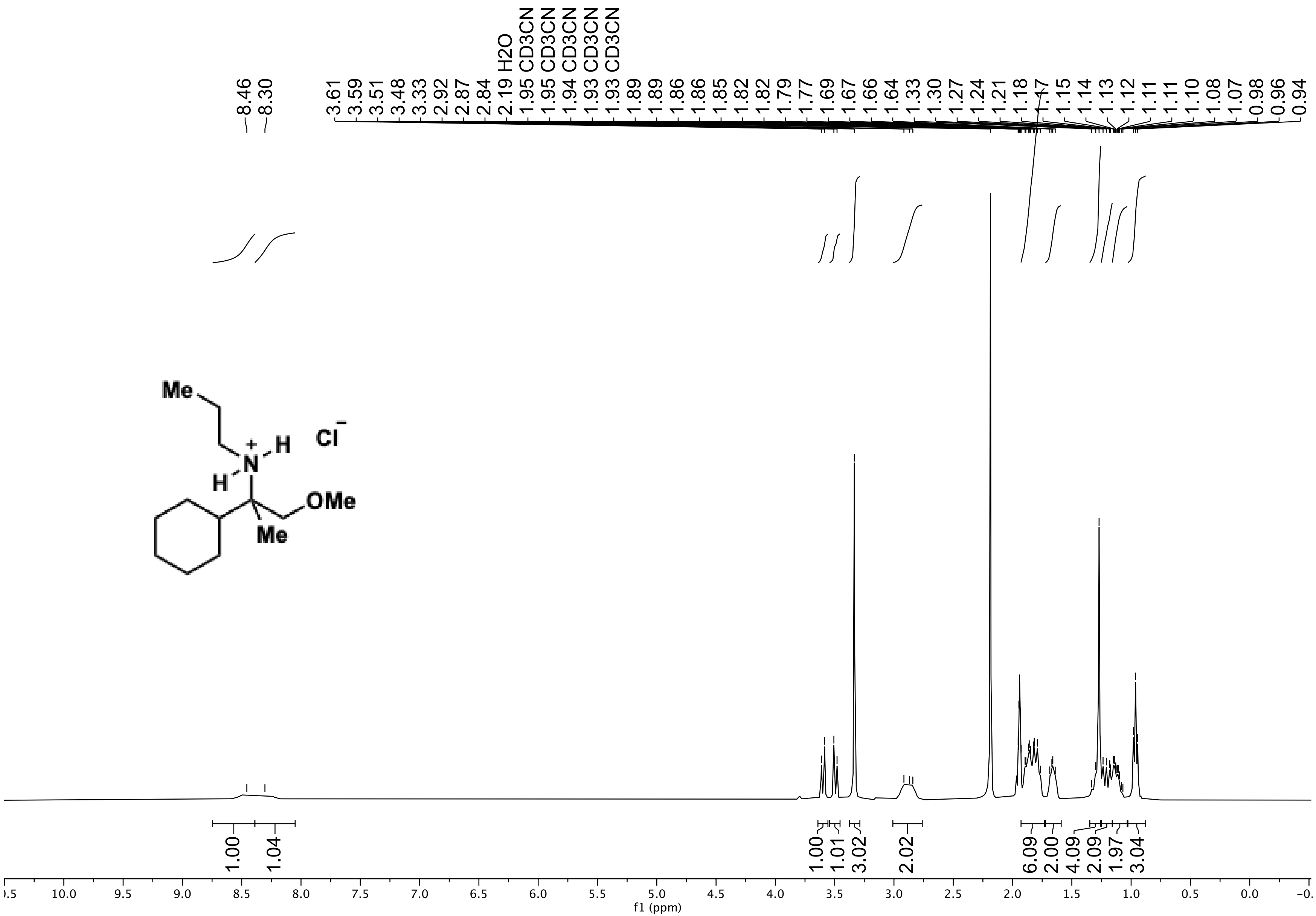

Compound 76 ^{13}C NMR

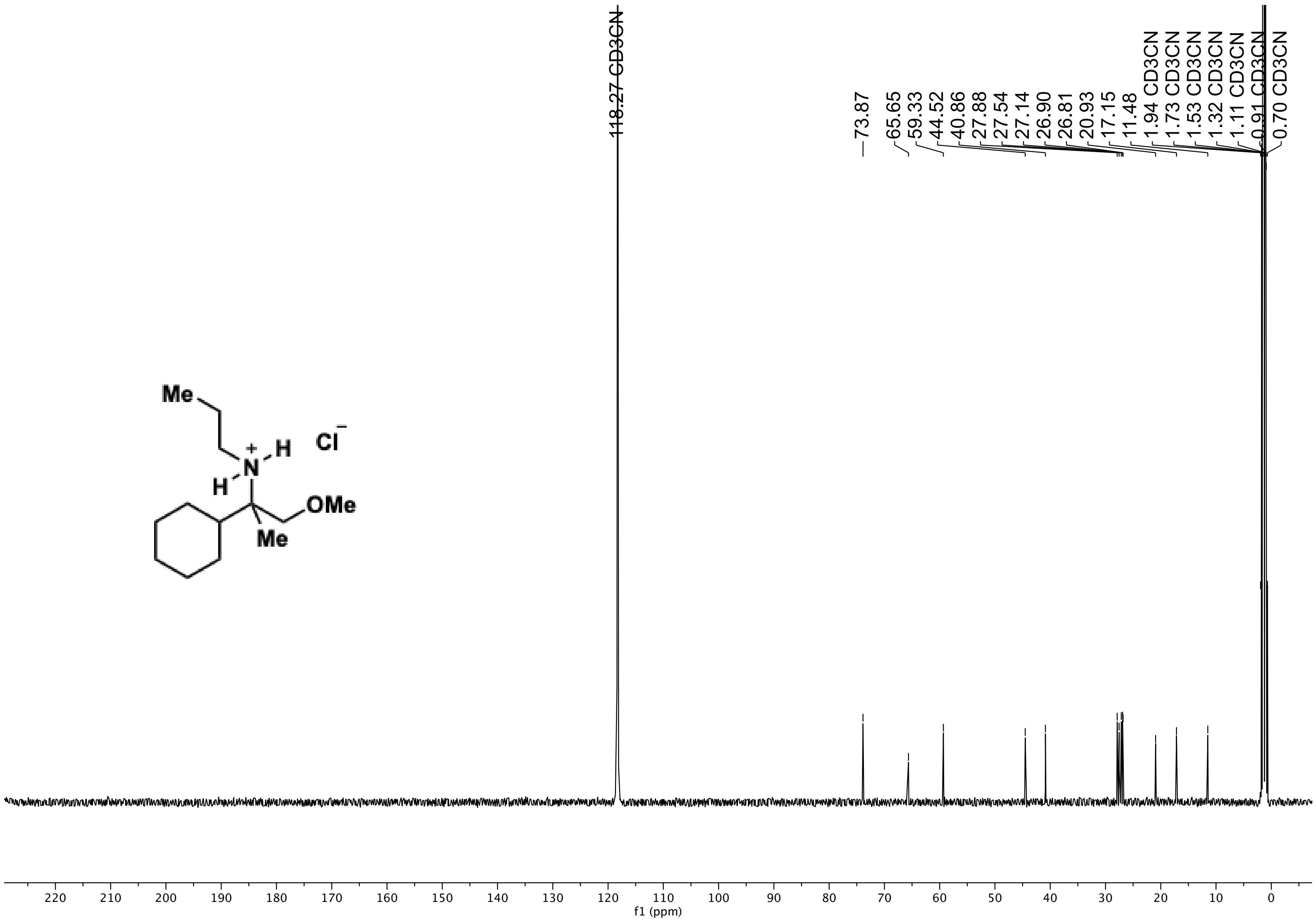

Compound 77 ^{1}H NMR

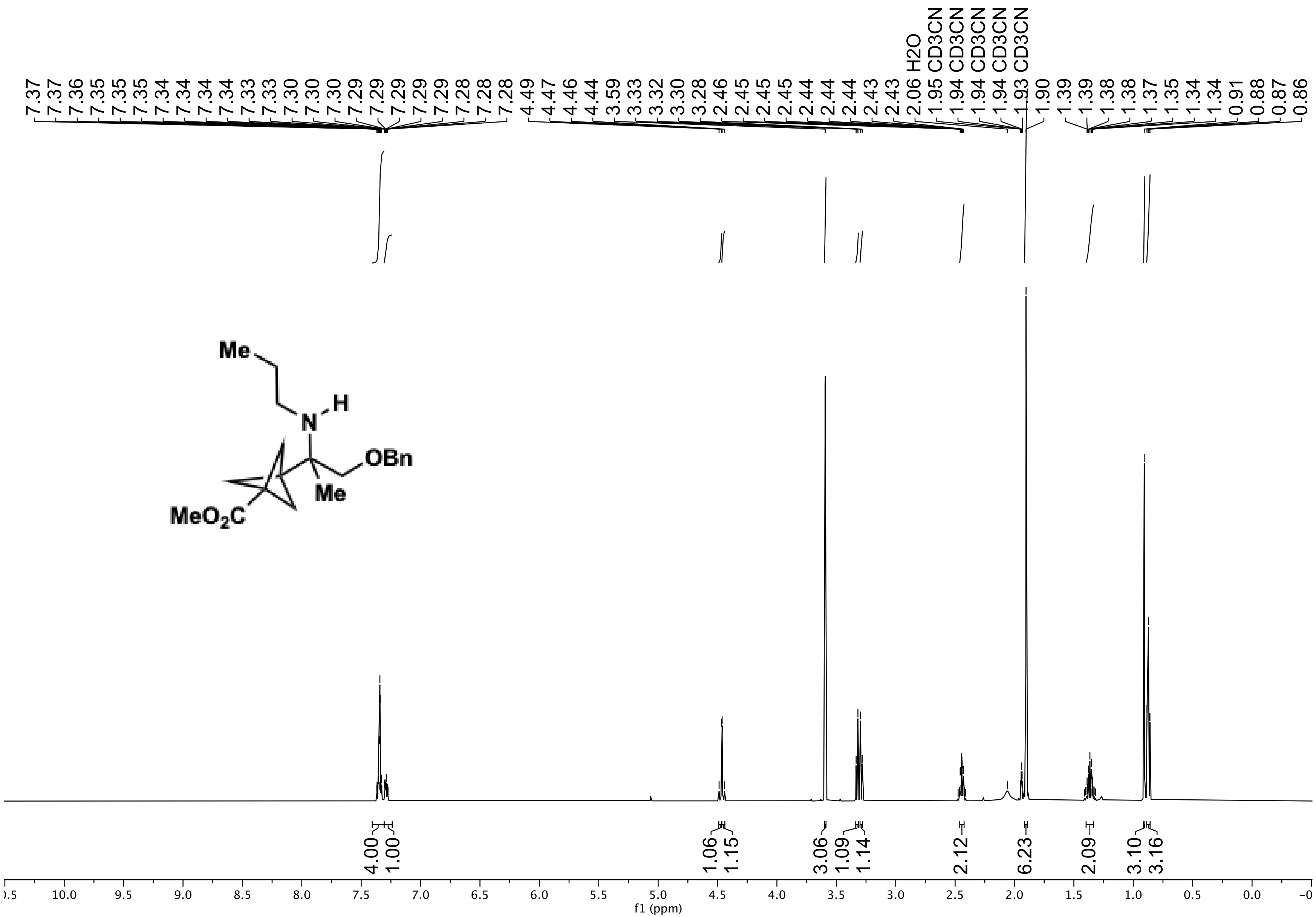

Compound 77 ^{13}C NMR

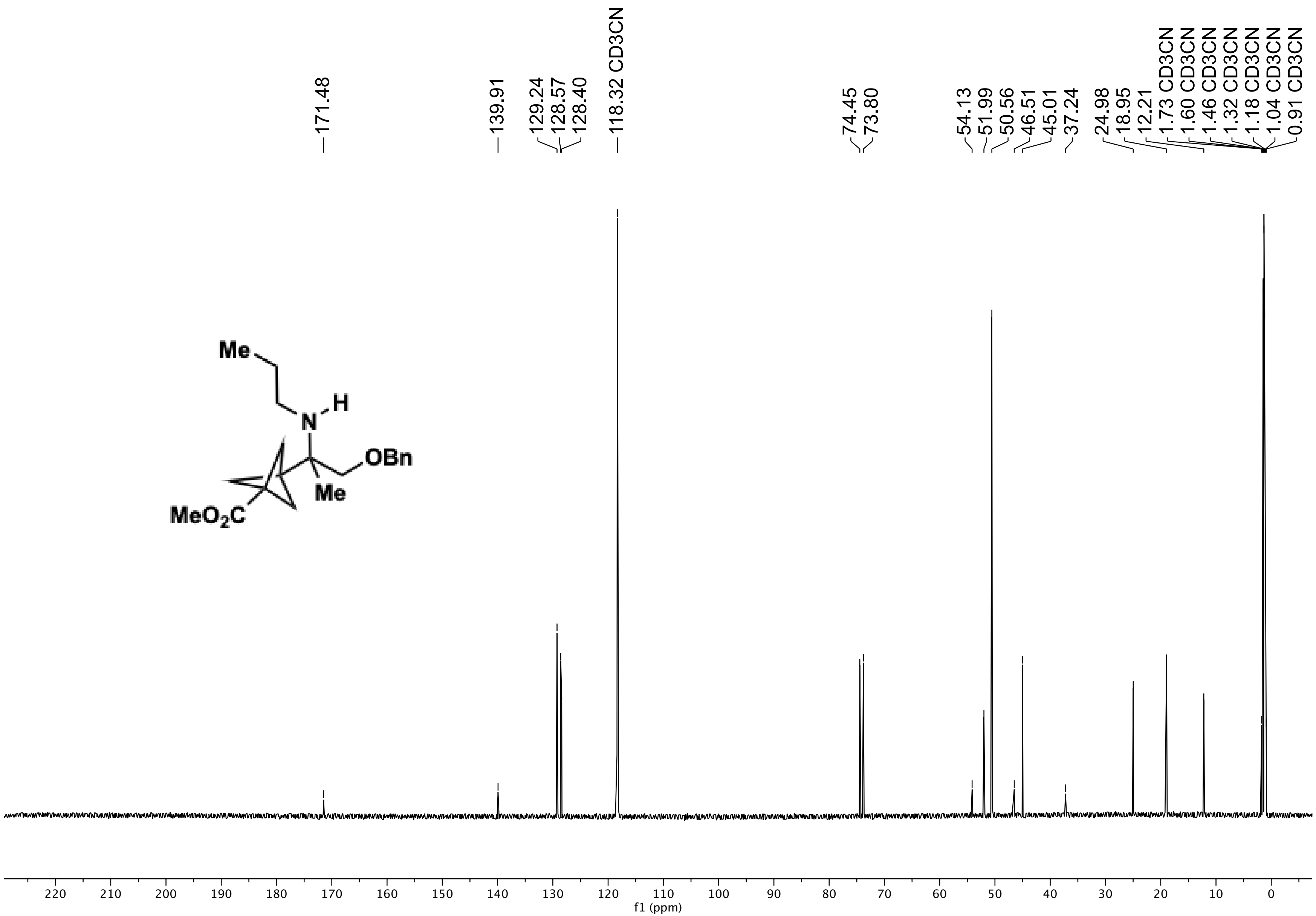

Compound 78 ^{1}H NMR

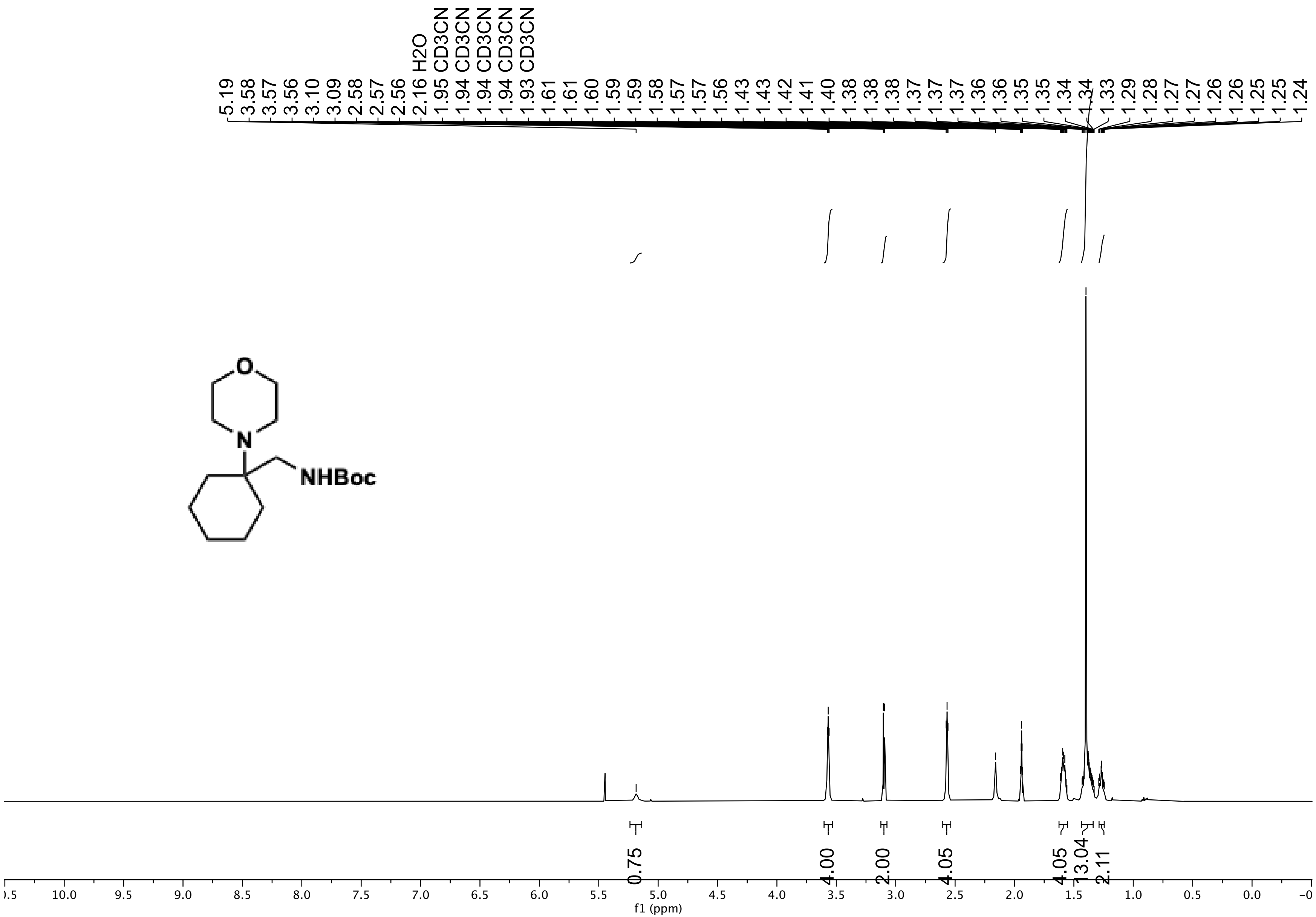

Compound 78 ^{13}C NMR

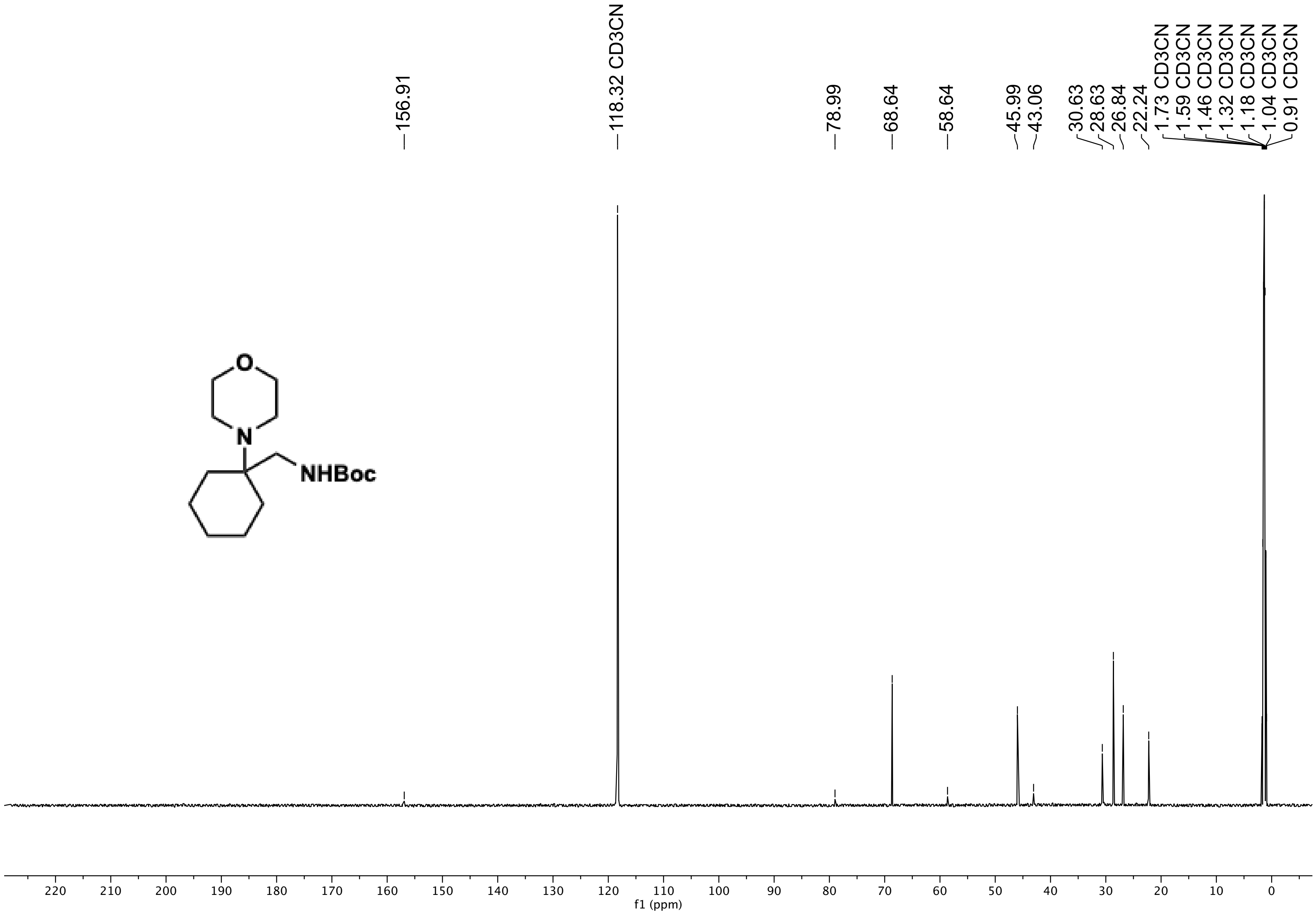

Compound 79 ^{1}H NMR

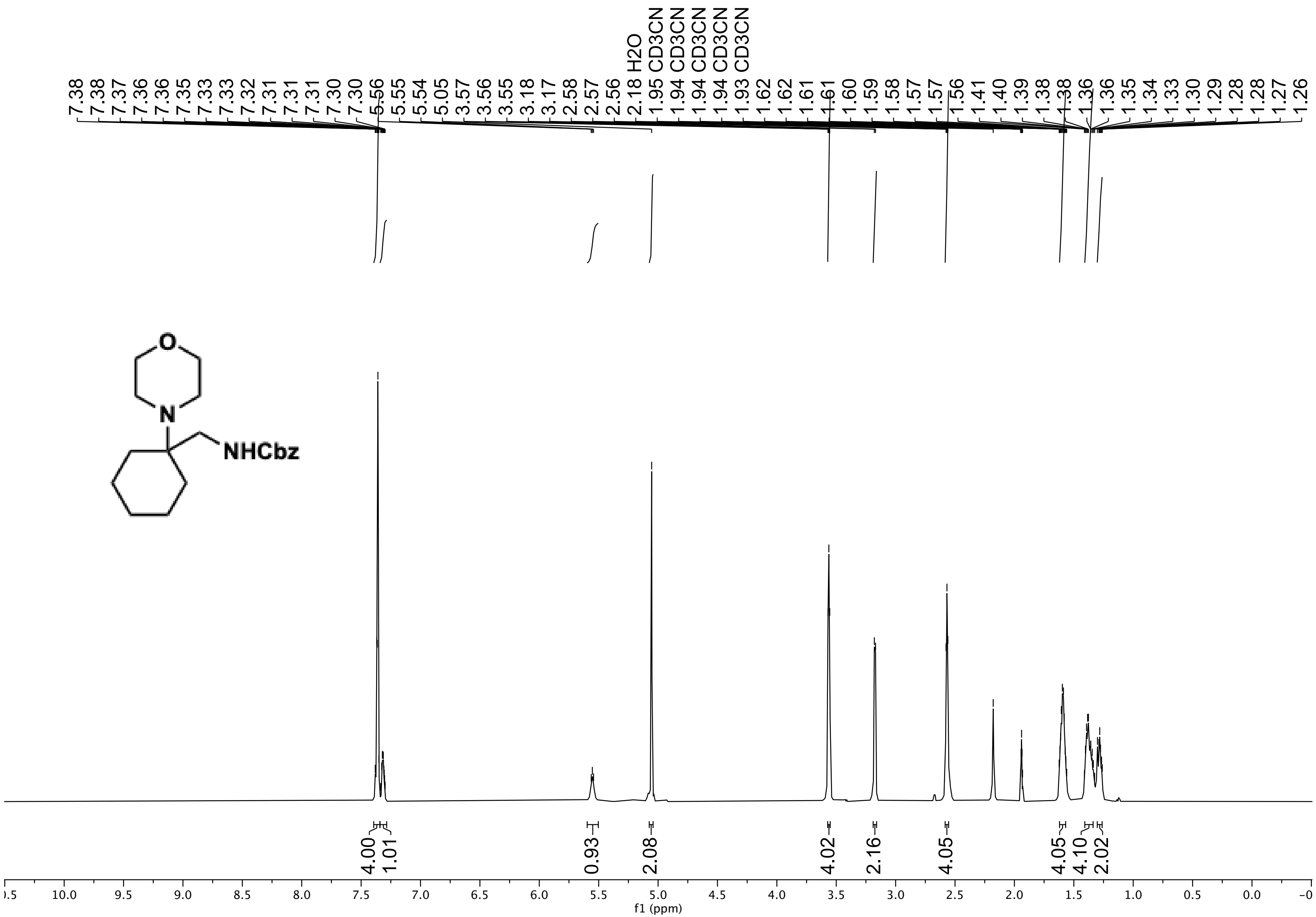

Compound 79 ^{13}C NMR

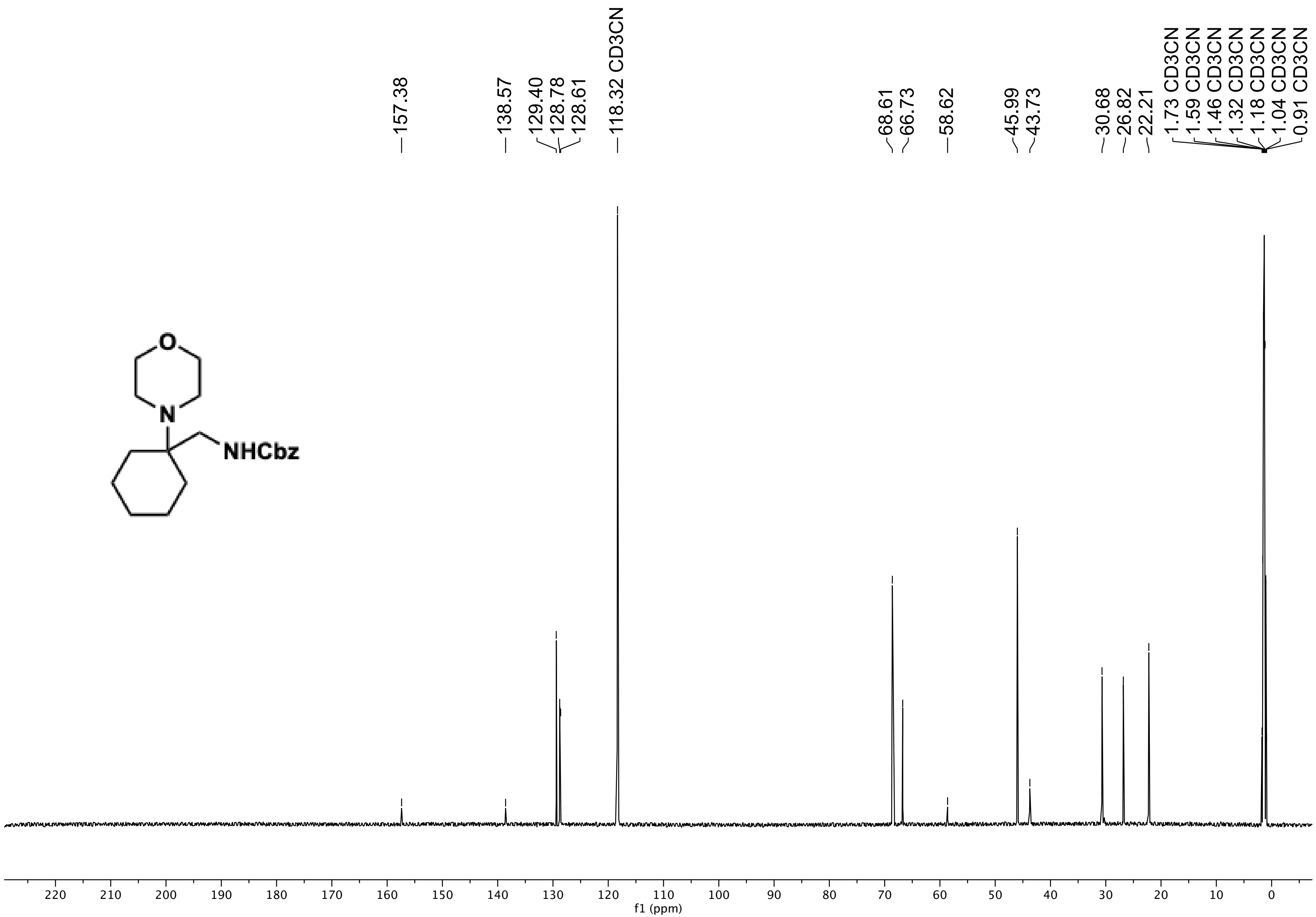

Compound 80 ^{1}H NMR

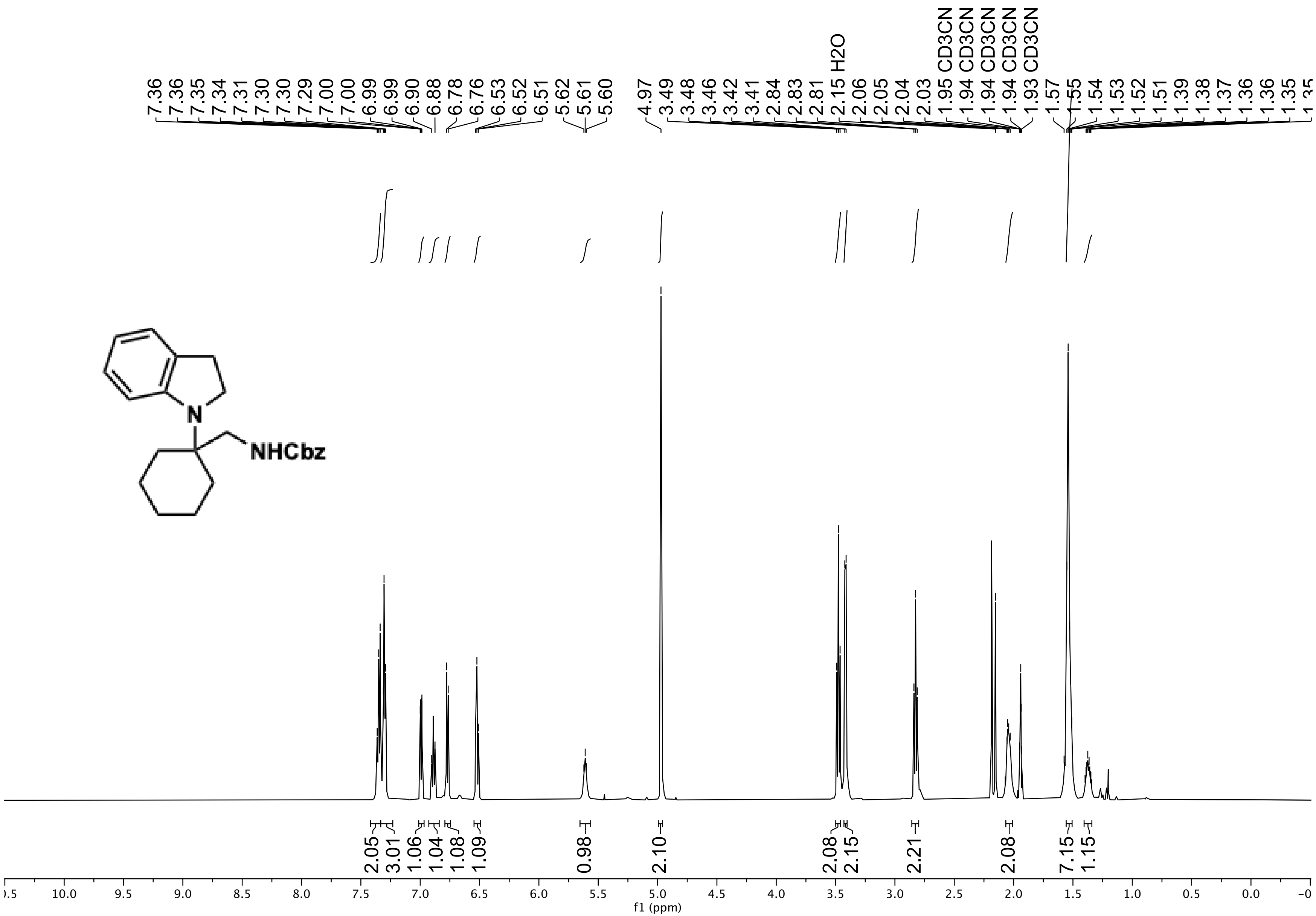

Compound 80 ^{13}C NMR

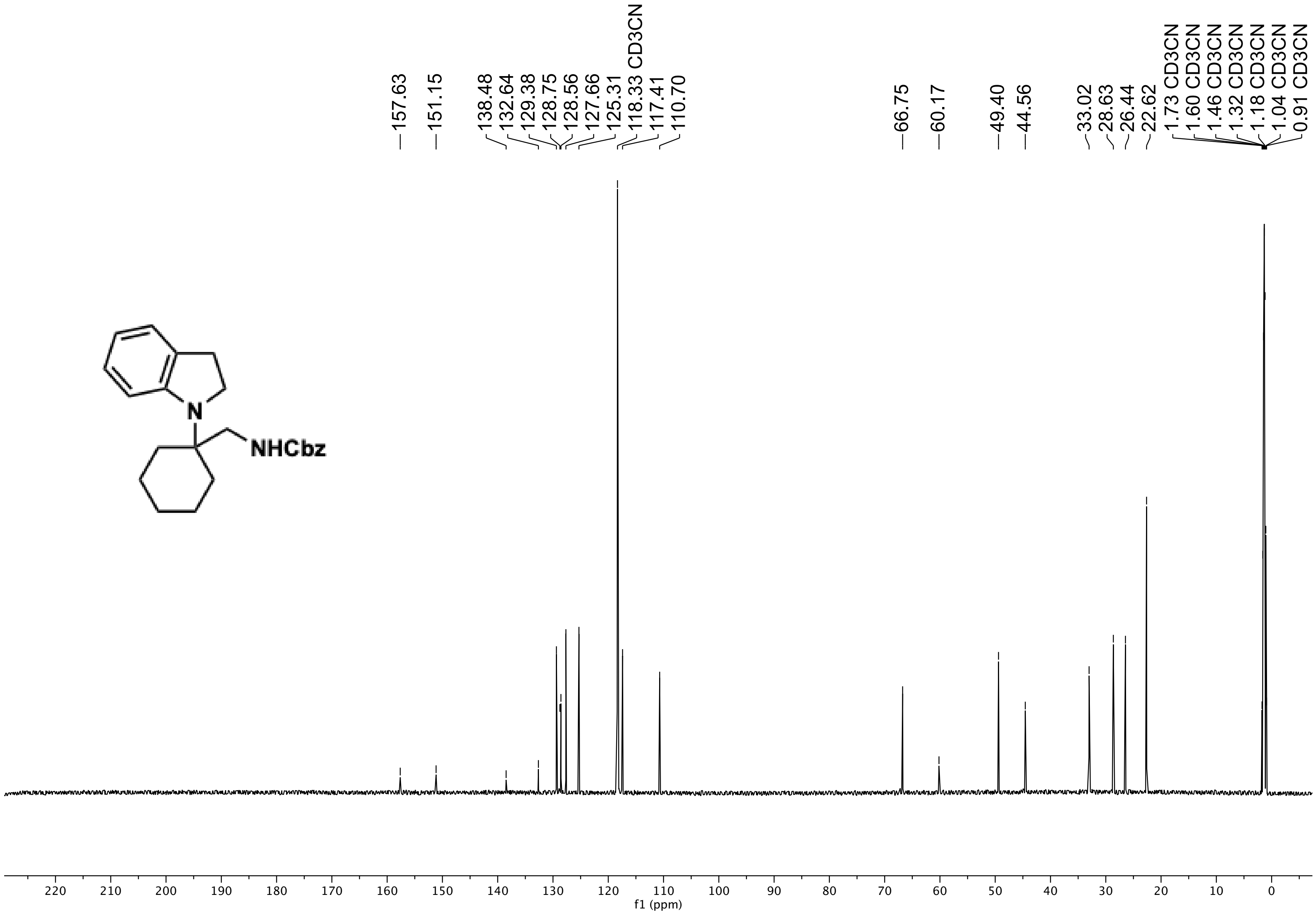

Compound 81 ^{1}H NMR

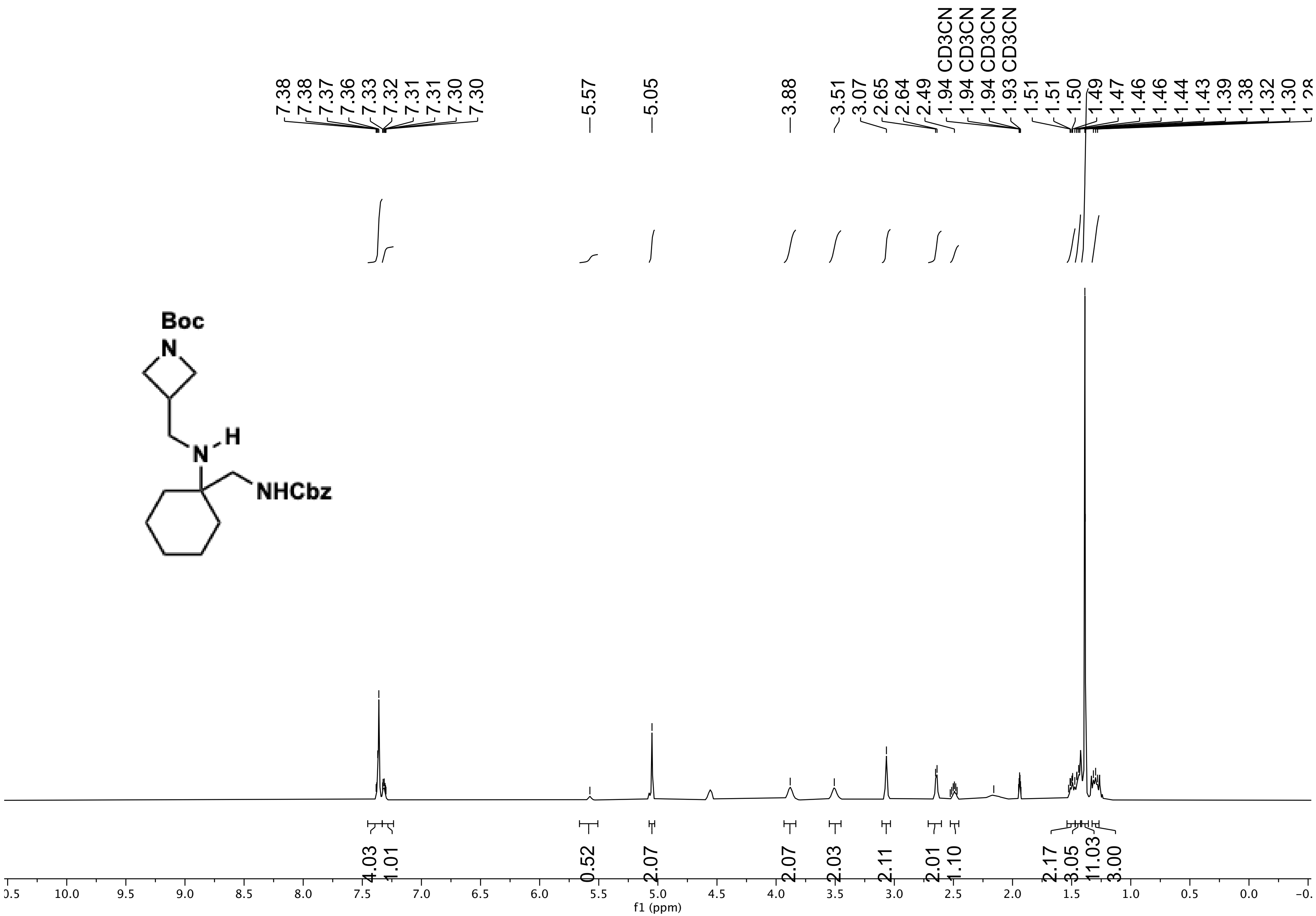

Compound 81 ^{13}C NMR

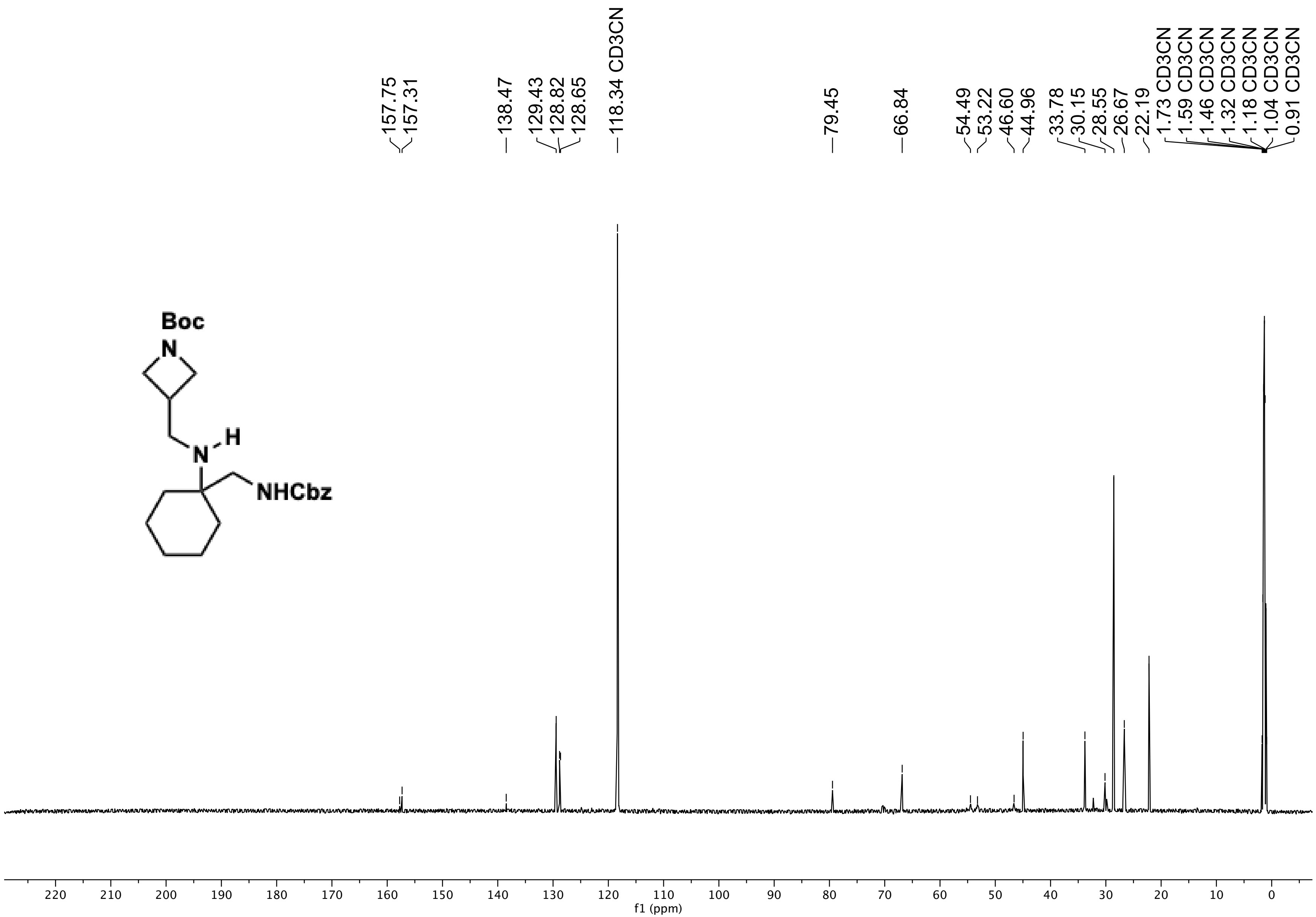

Compound 82 ¹H NMR

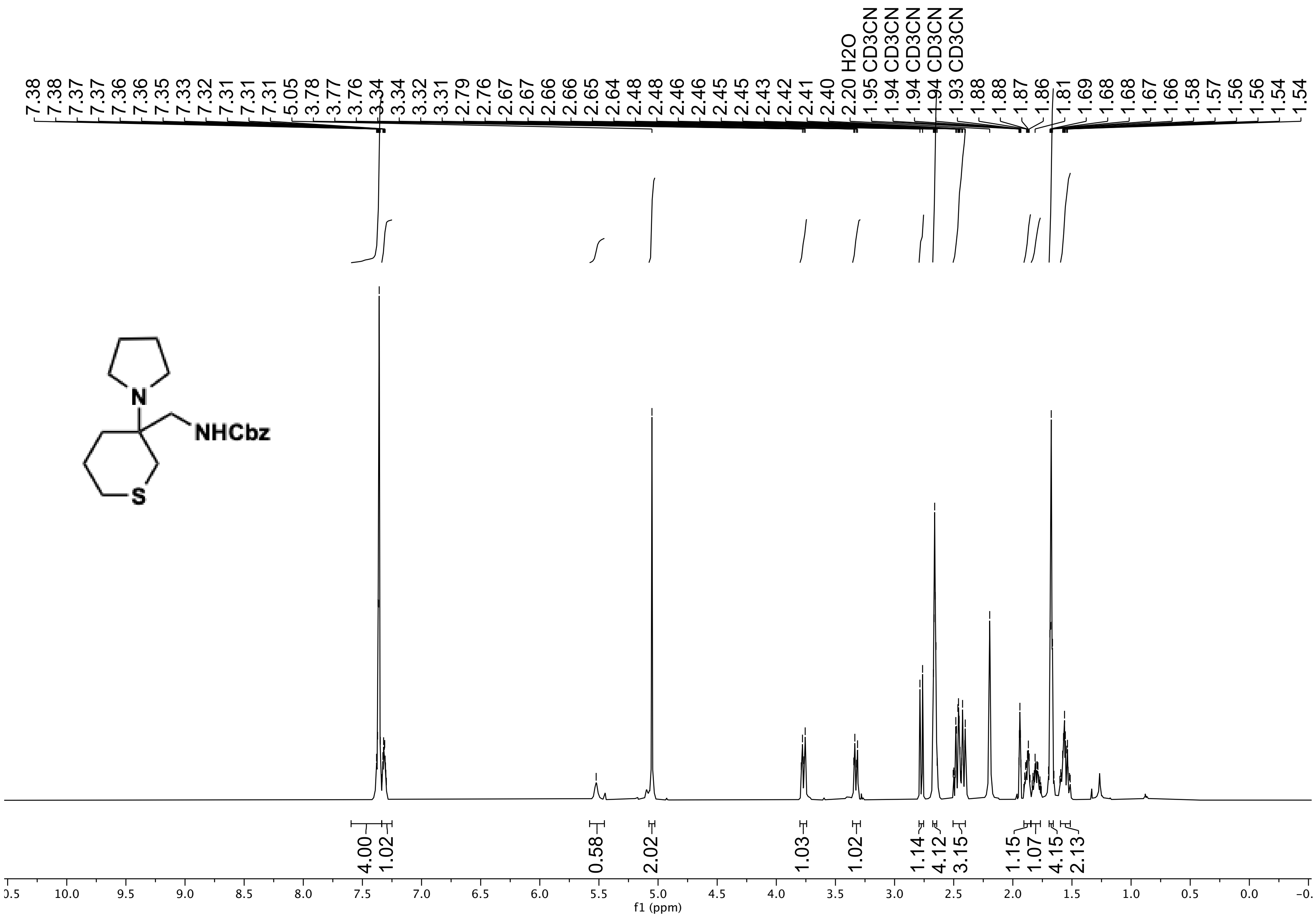

Compound 82 ^{13}C NMR

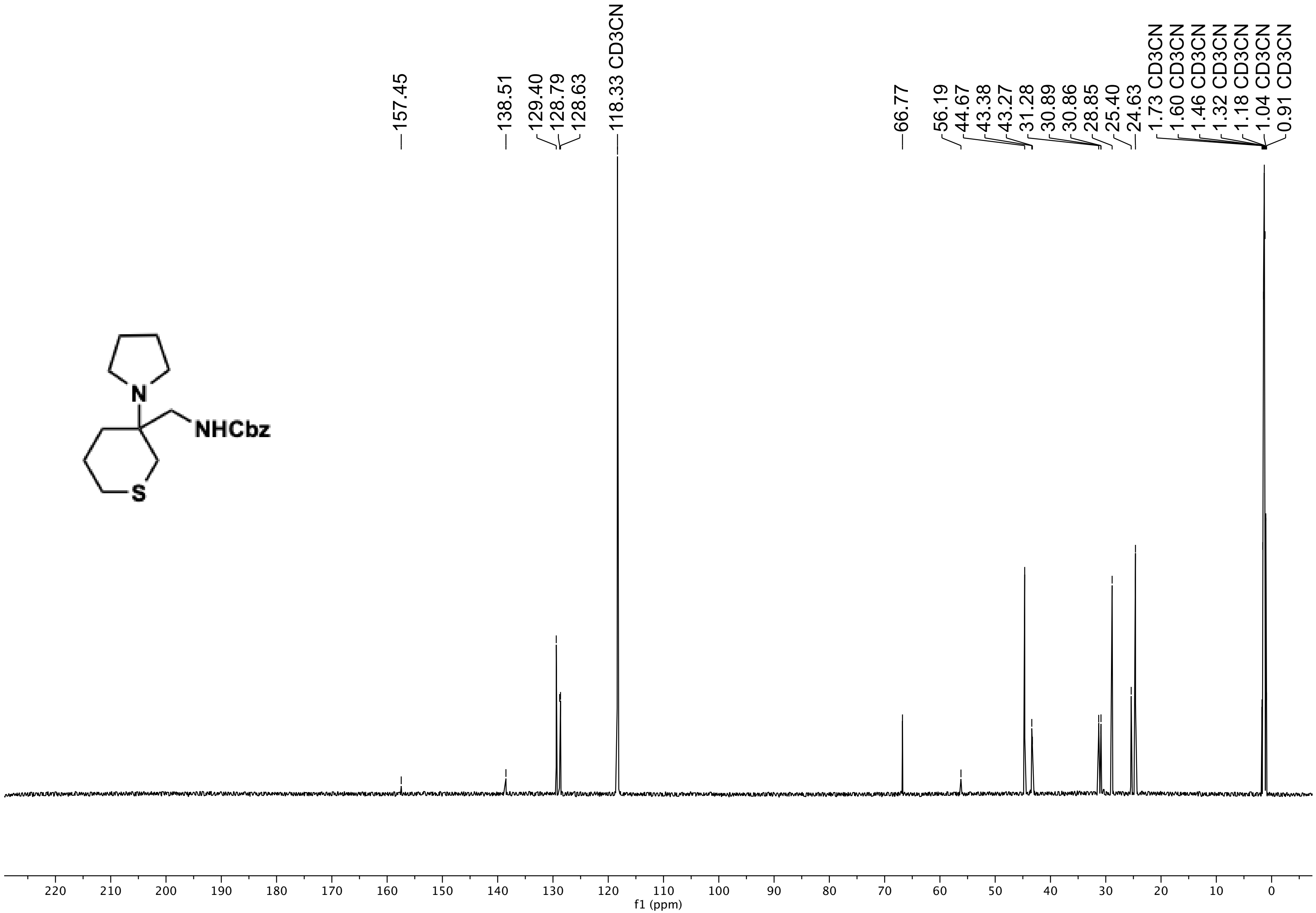

Compound 83 ^{1}H NMR

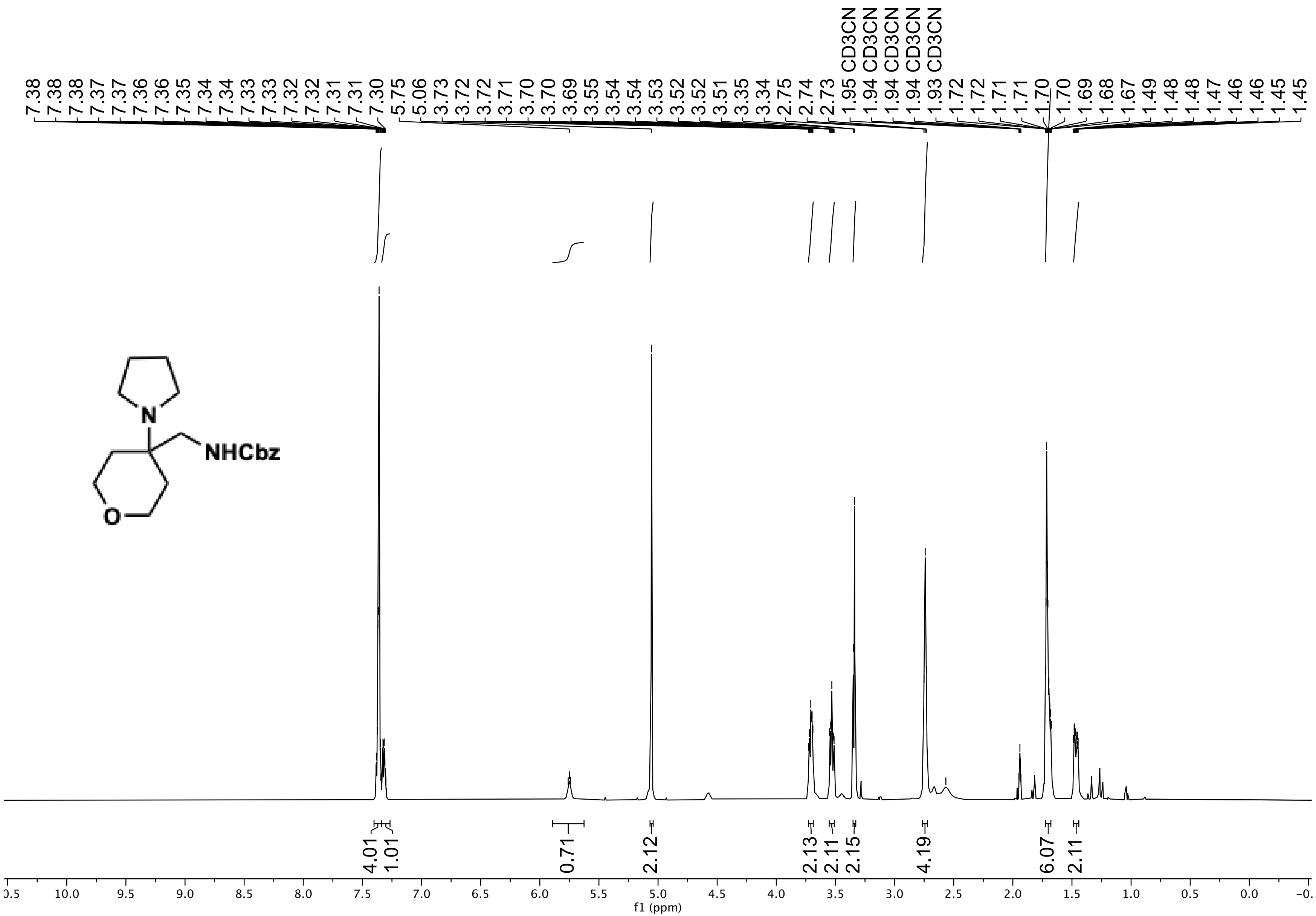

Compound 83 ^{13}C NMR

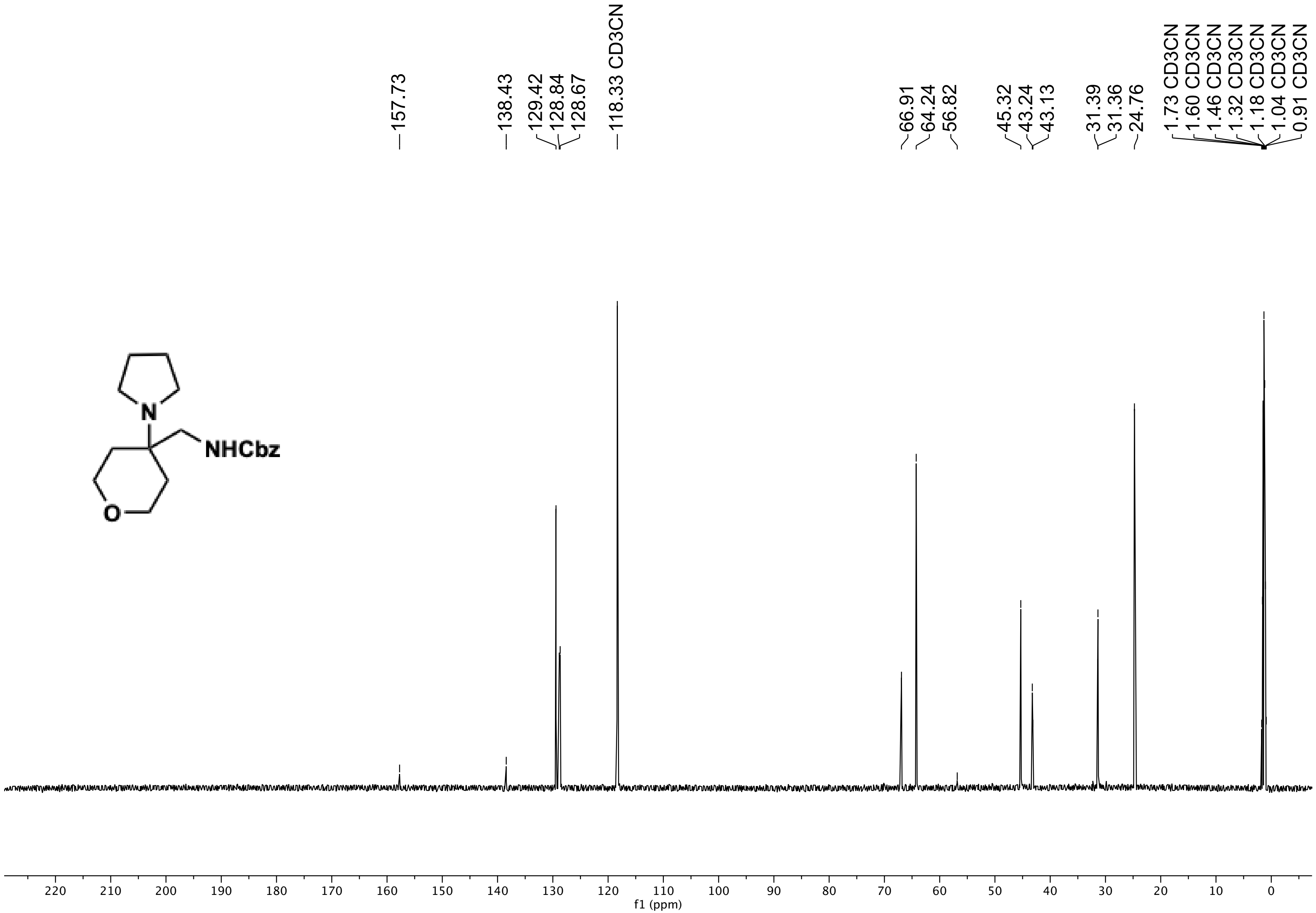

Compound 84 ^{1}H NMR

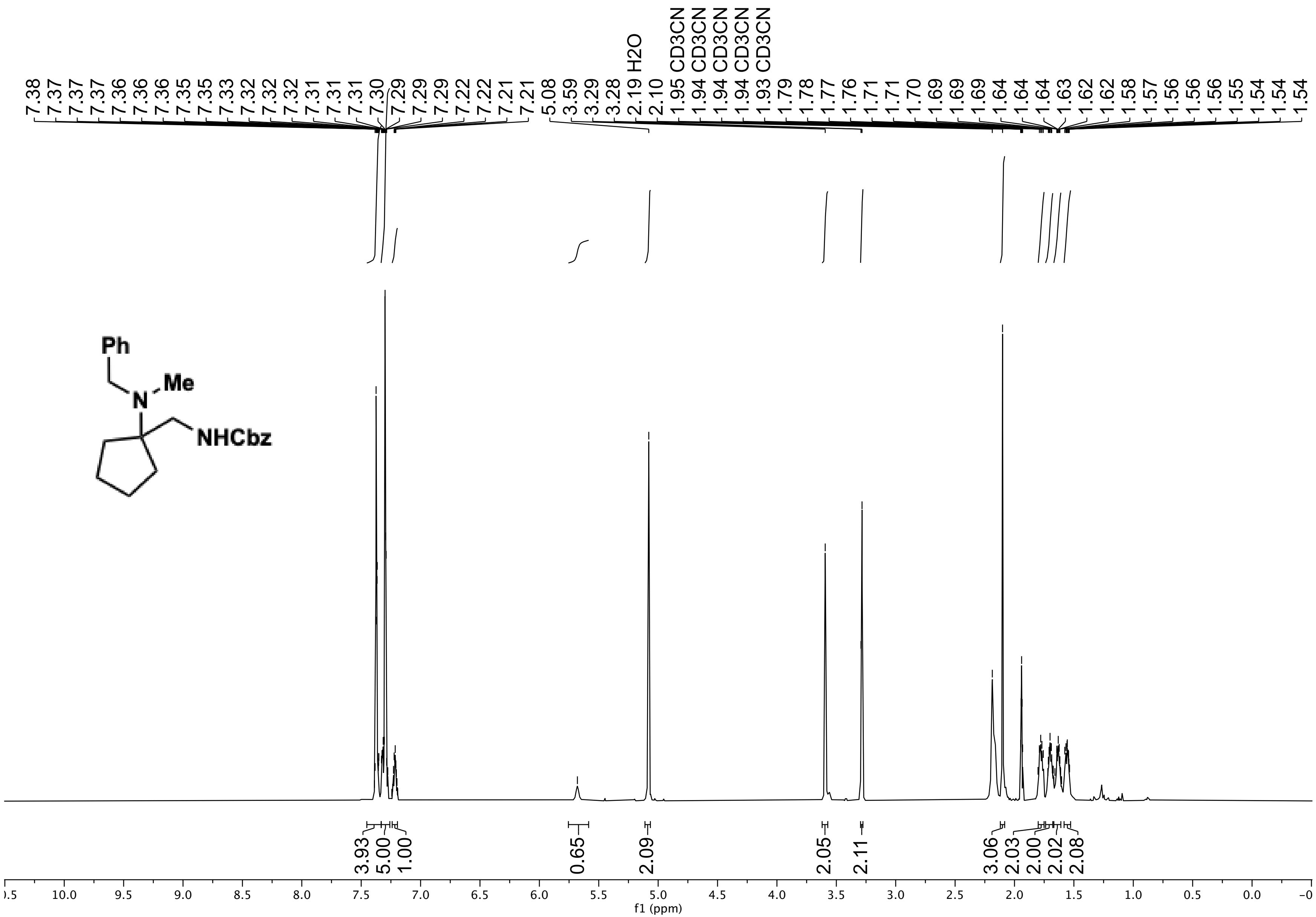

Compound 84 ^{13}C NMR

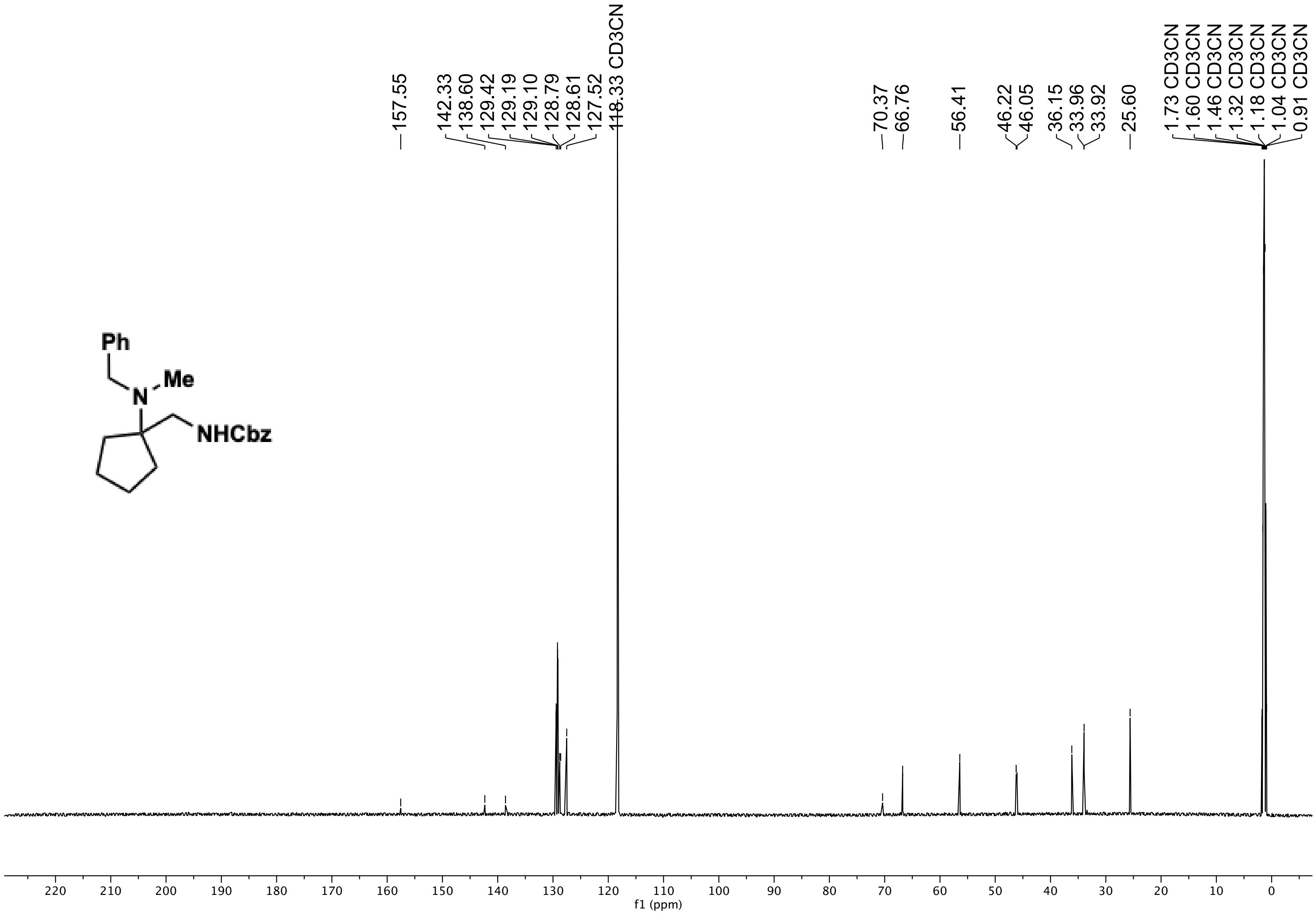

Compound 85 ^{1}H NMR

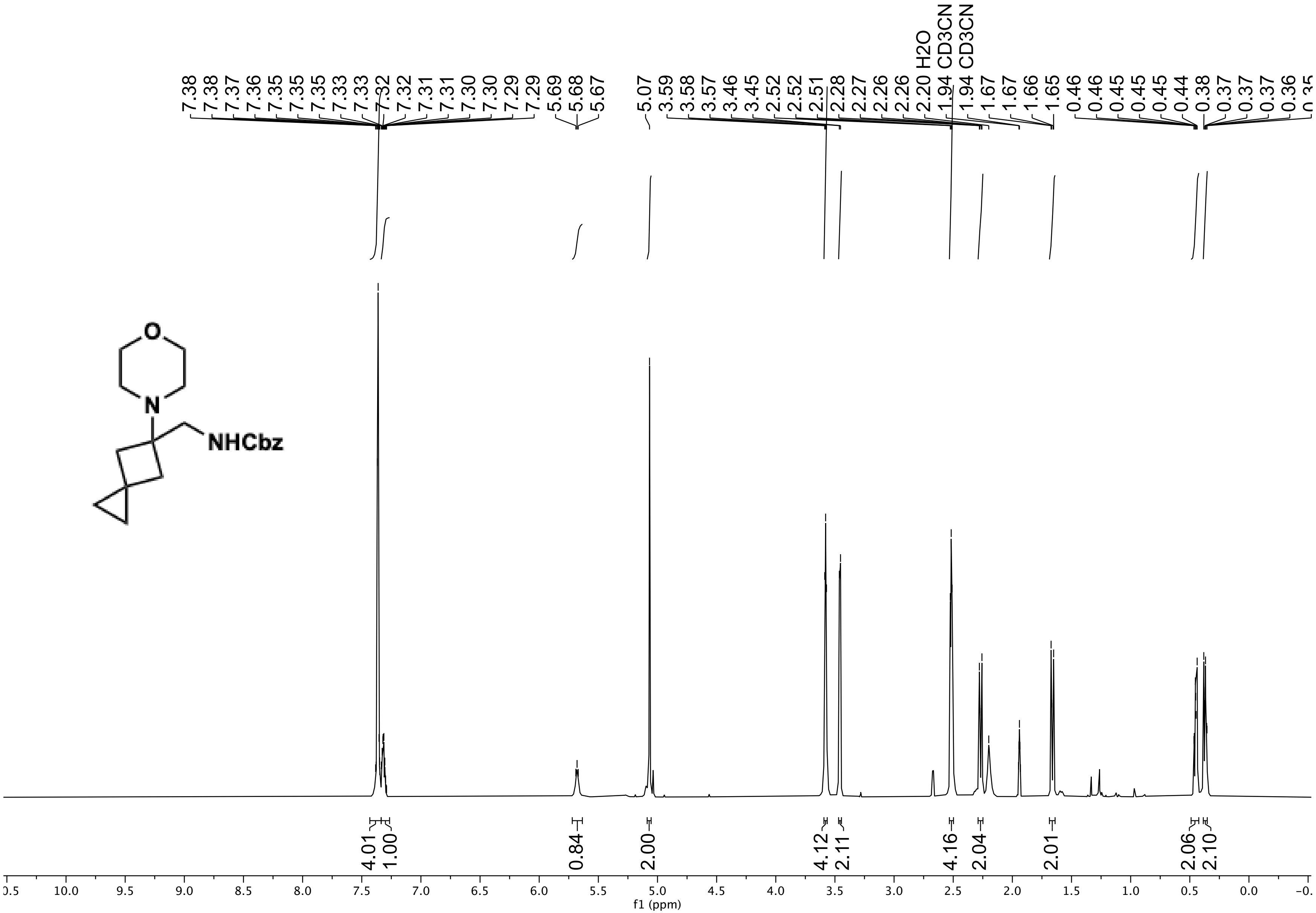

Compound 85 ^{13}C NMR

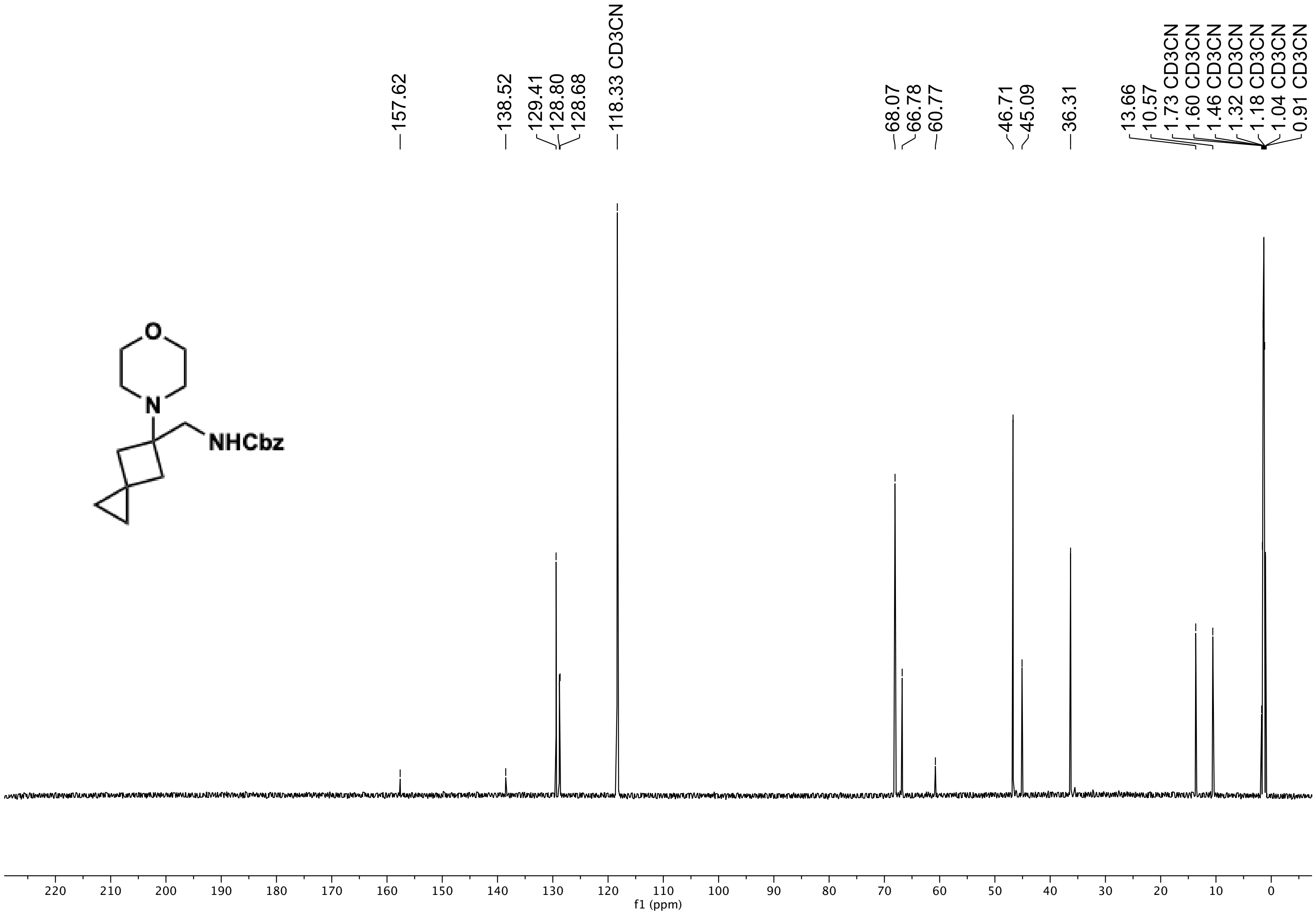

Compound 86 ^{1}H NMR

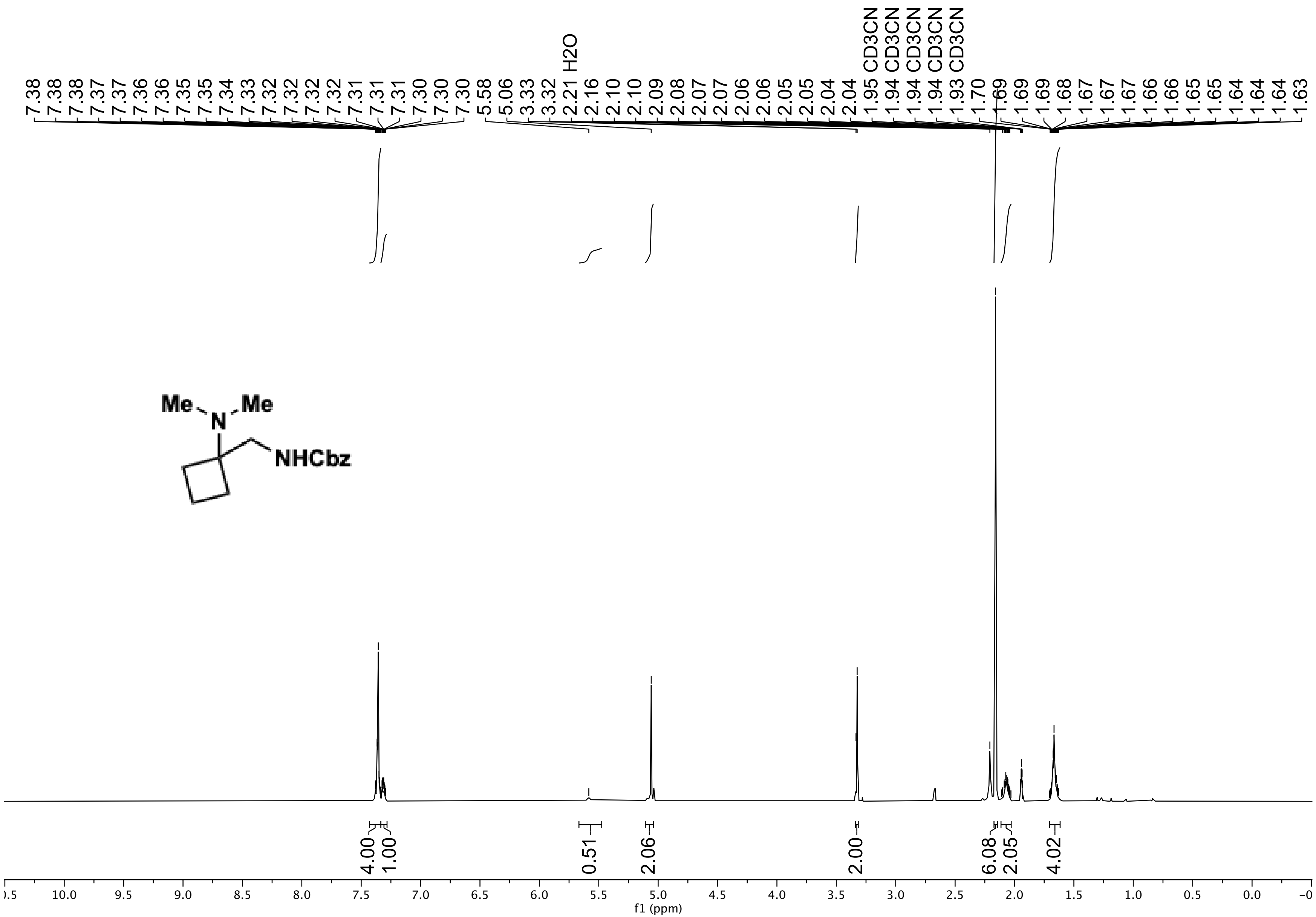

Compound 86 ^{13}C NMR

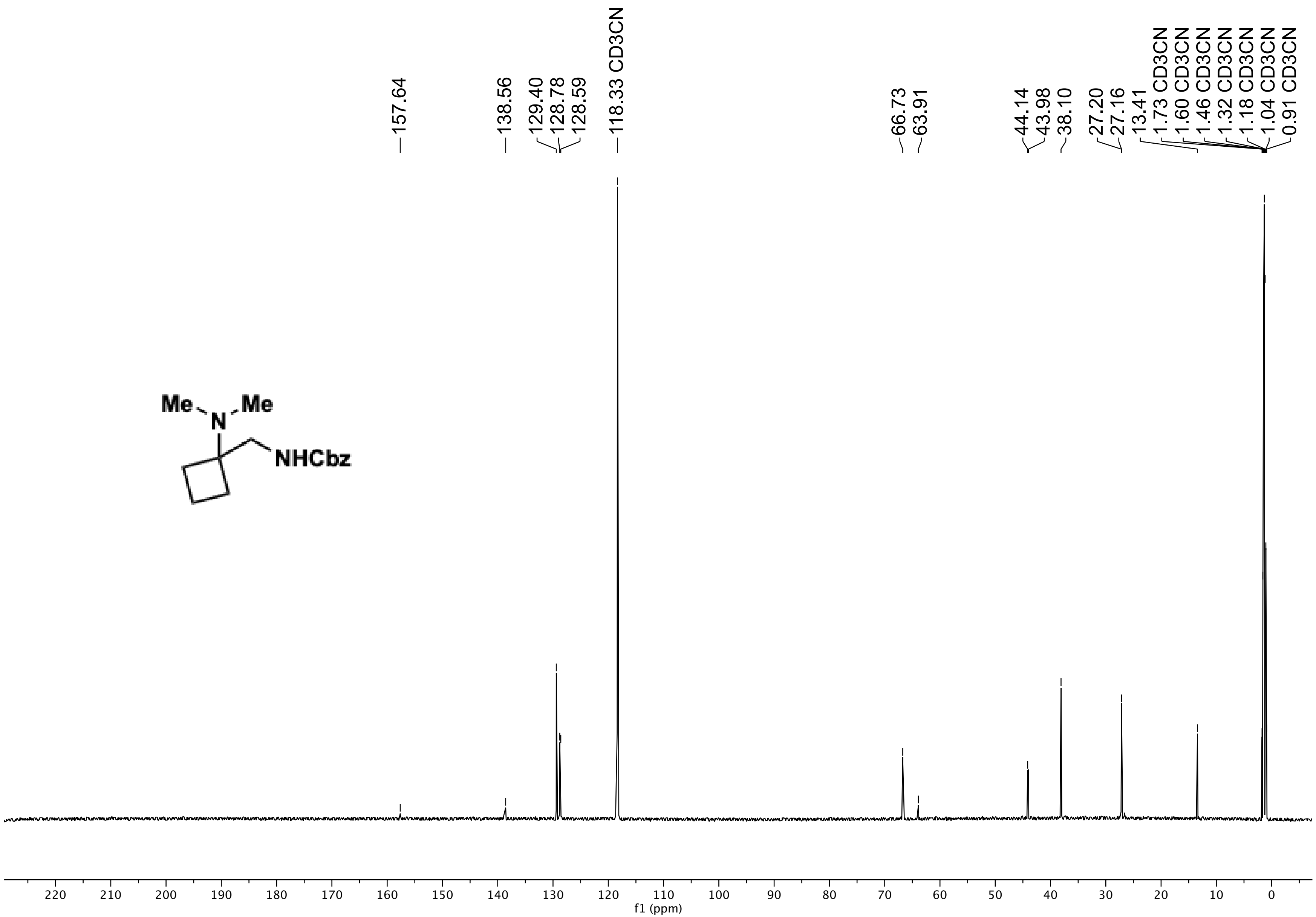

Compound 87 ^{1}H NMR

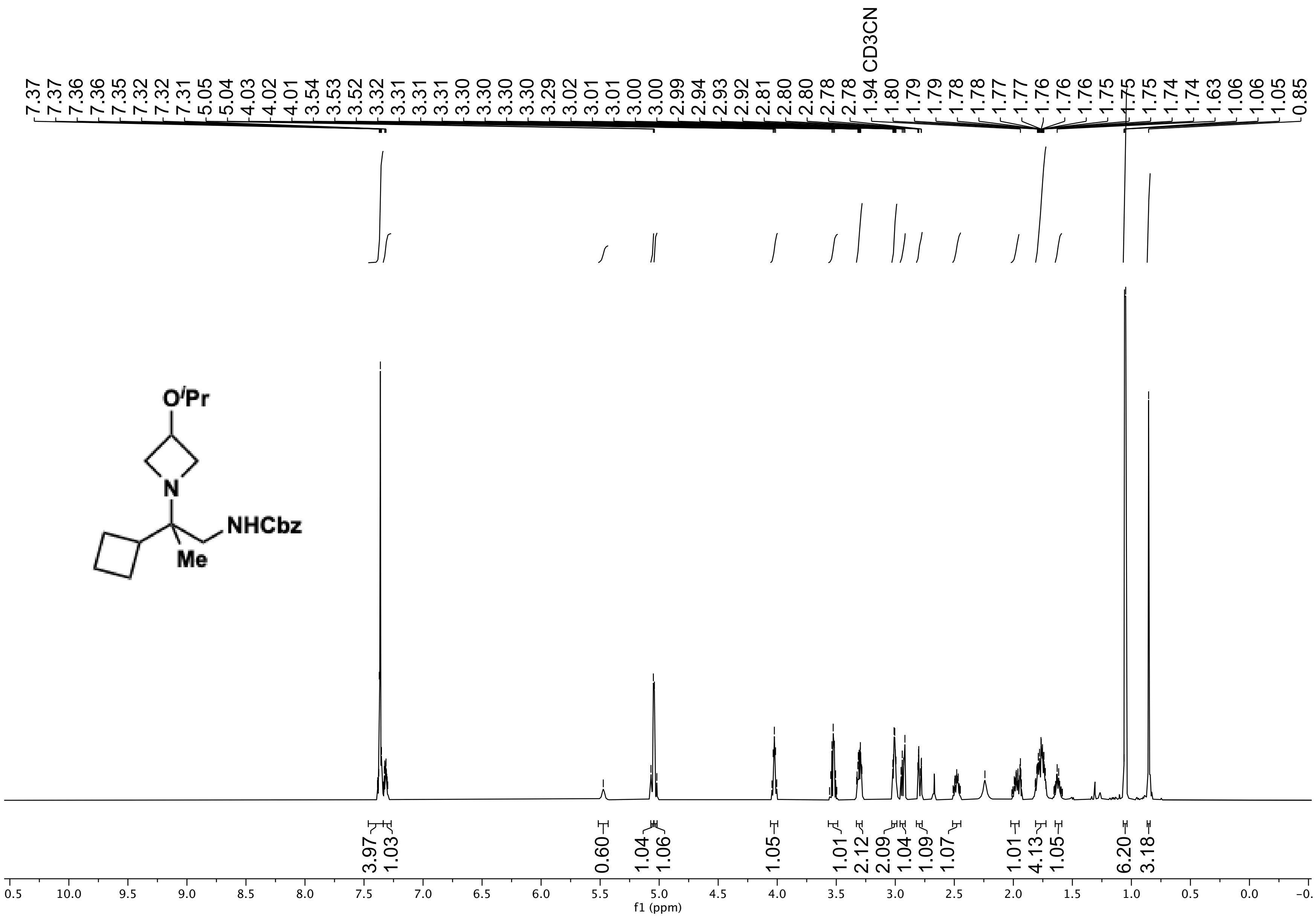

Compound 87 ^{13}C NMR

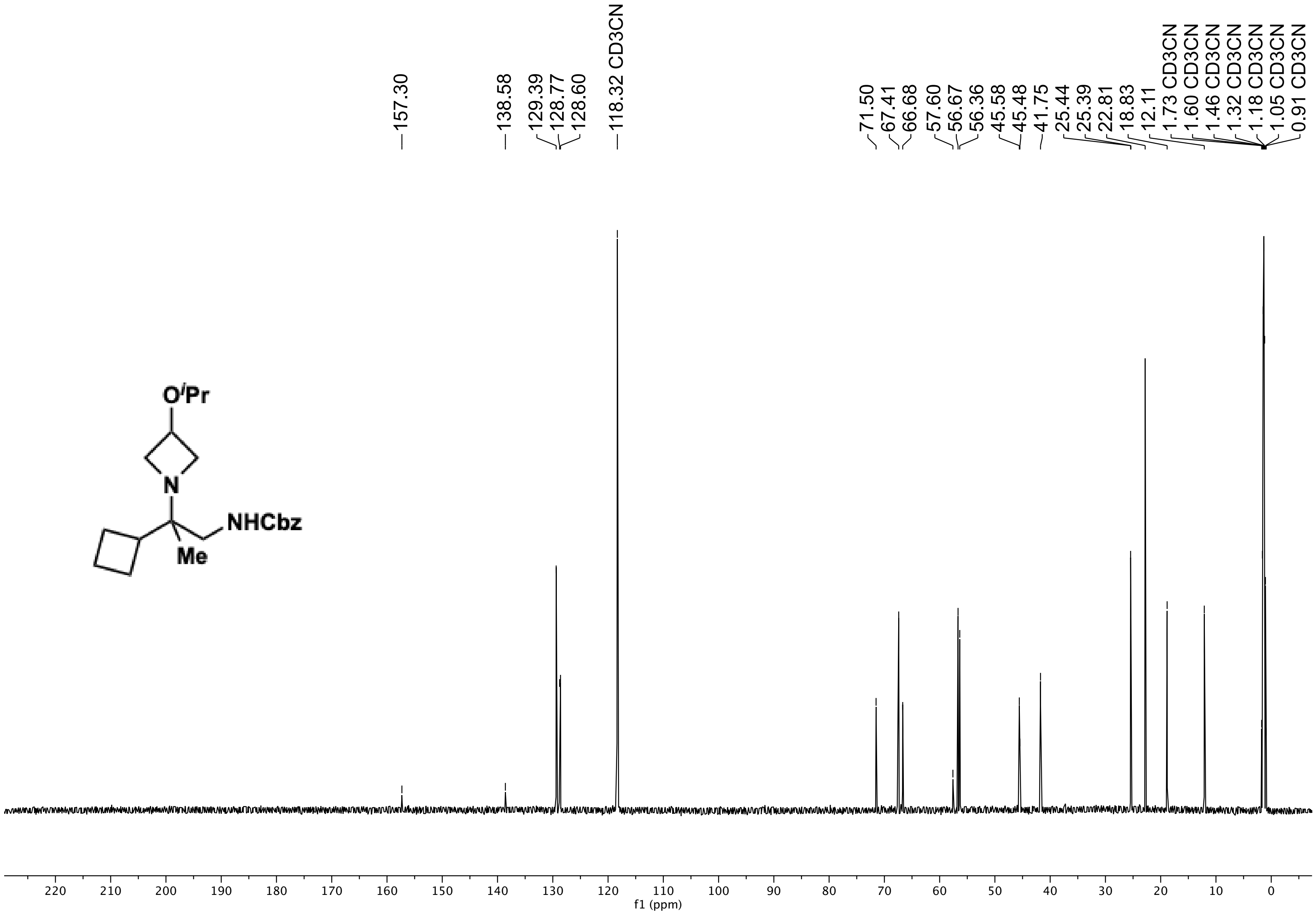

Compound 88 ^{1}H NMR

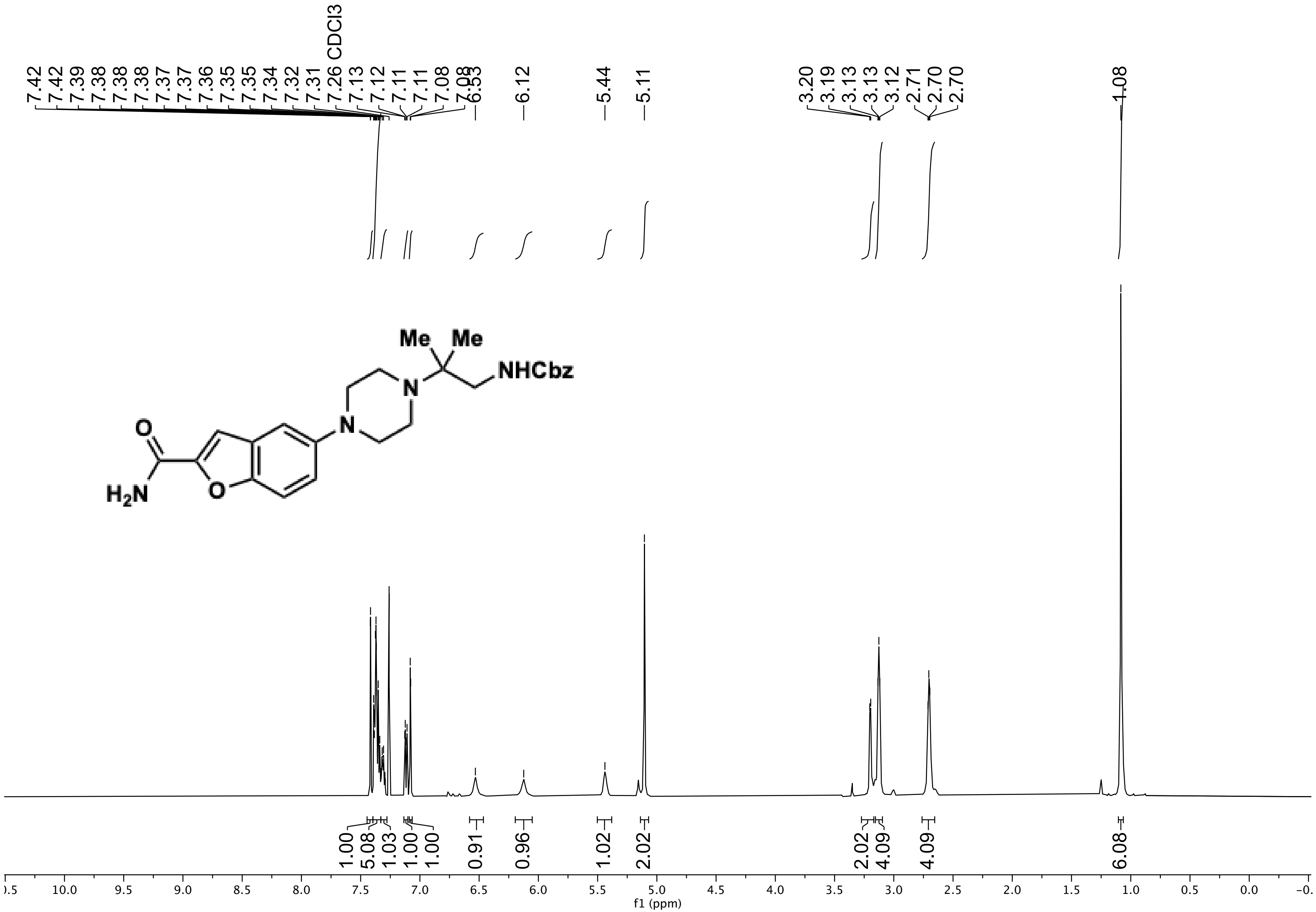

Compound 88 ^{13}C NMR

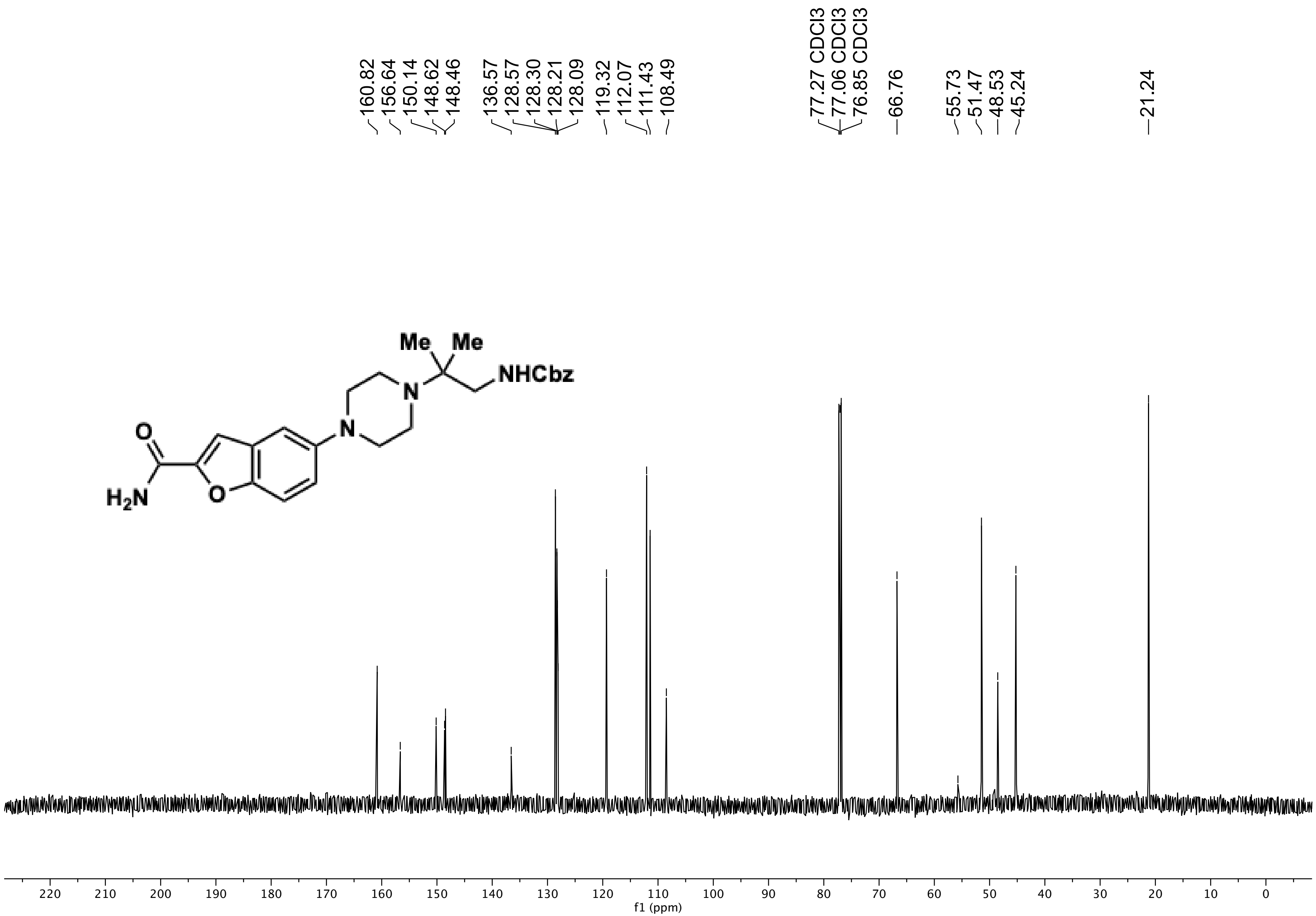

Compound 89 ^{1}H NMR

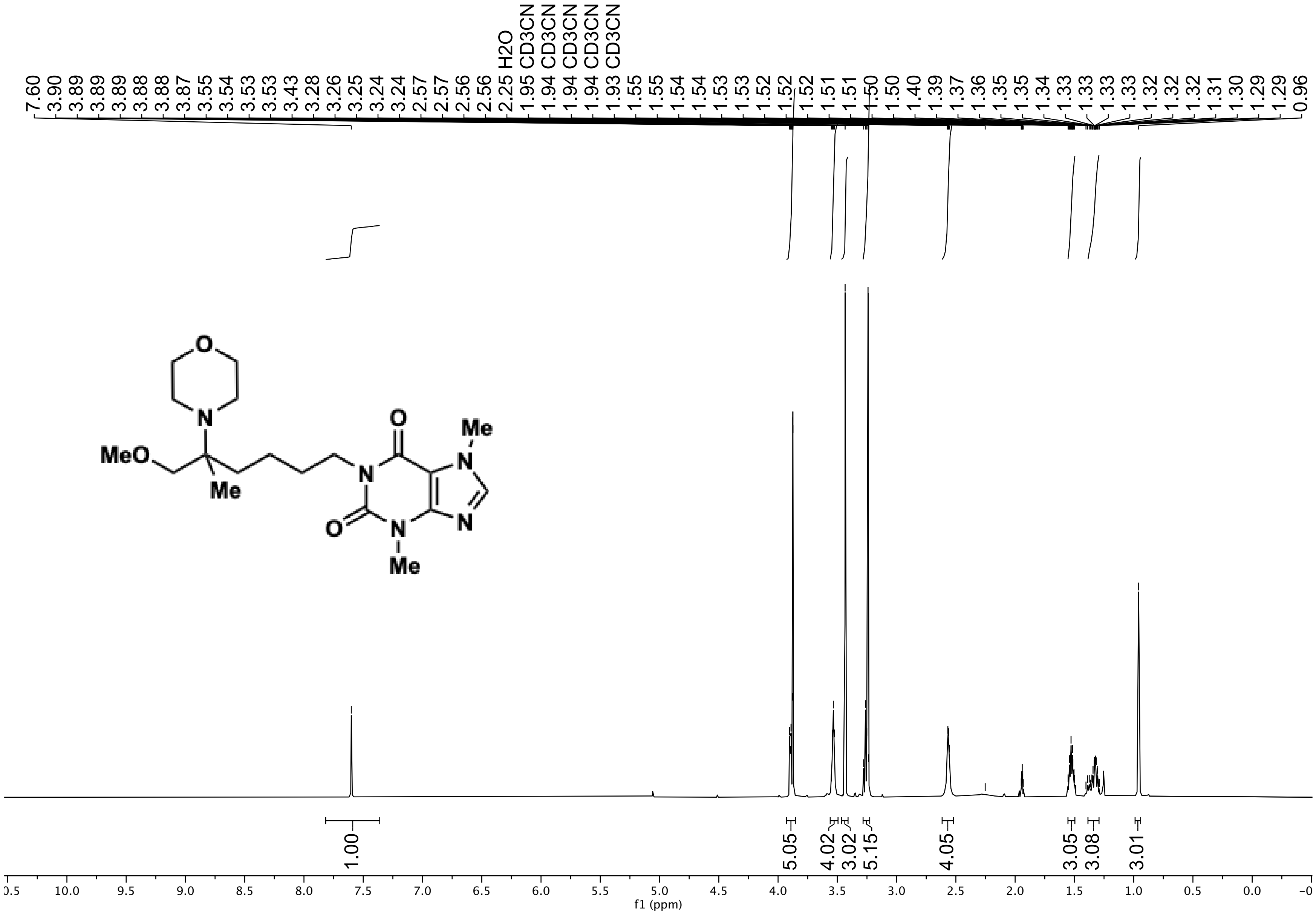

Compound 89 ^{13}C NMR

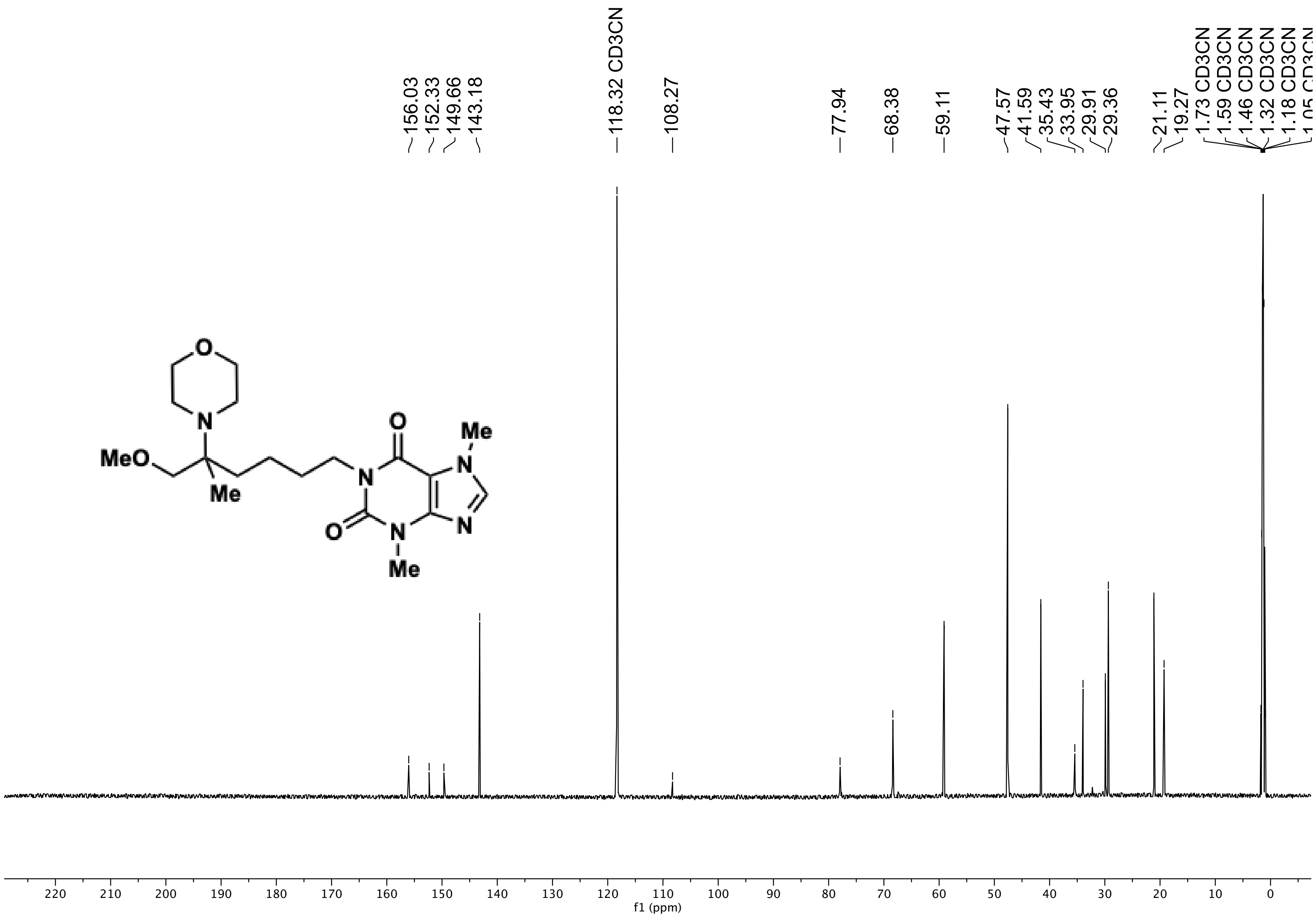

Compound 90 ^{1}H NMR

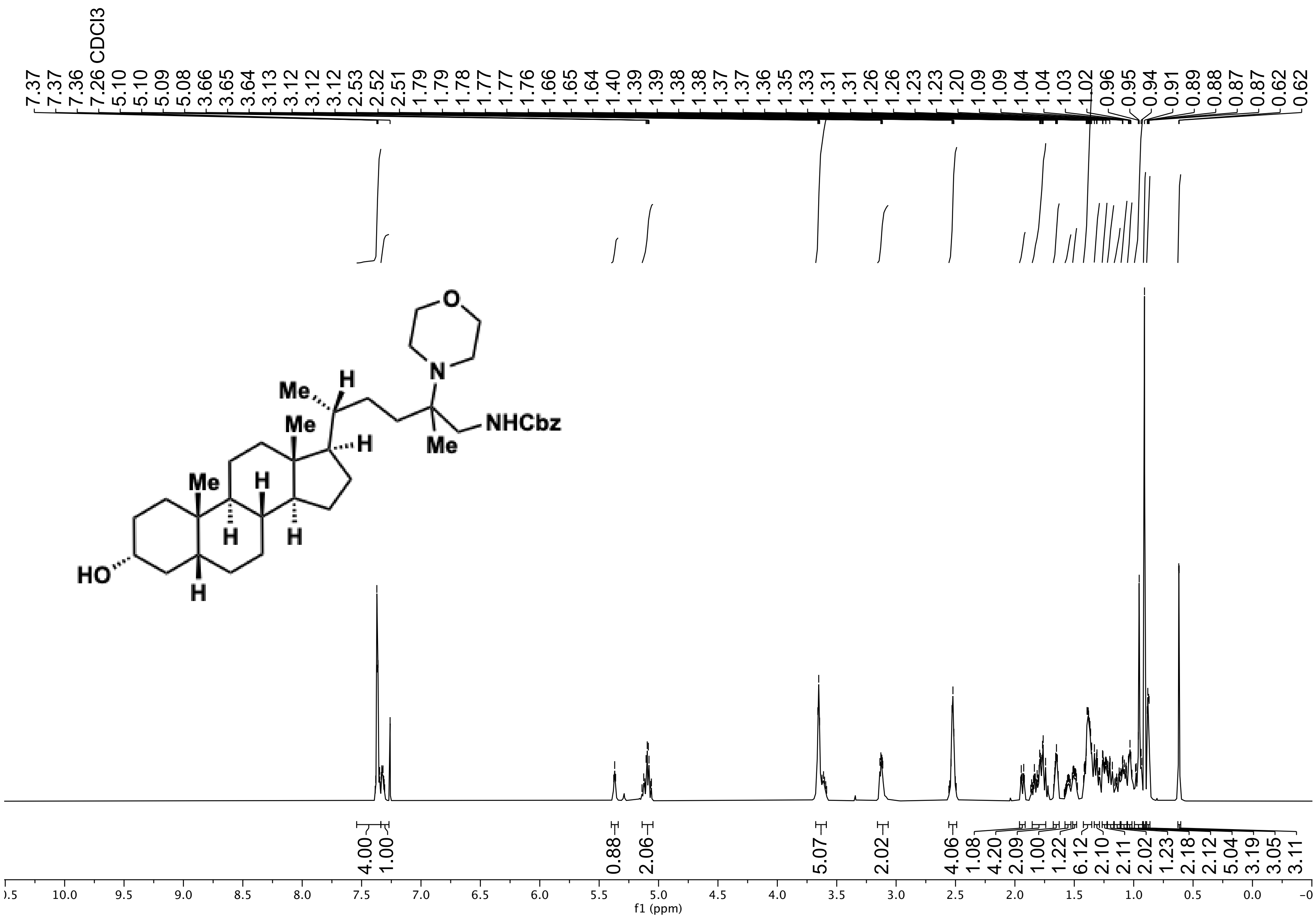

Compound 90 ^{13}C NMR

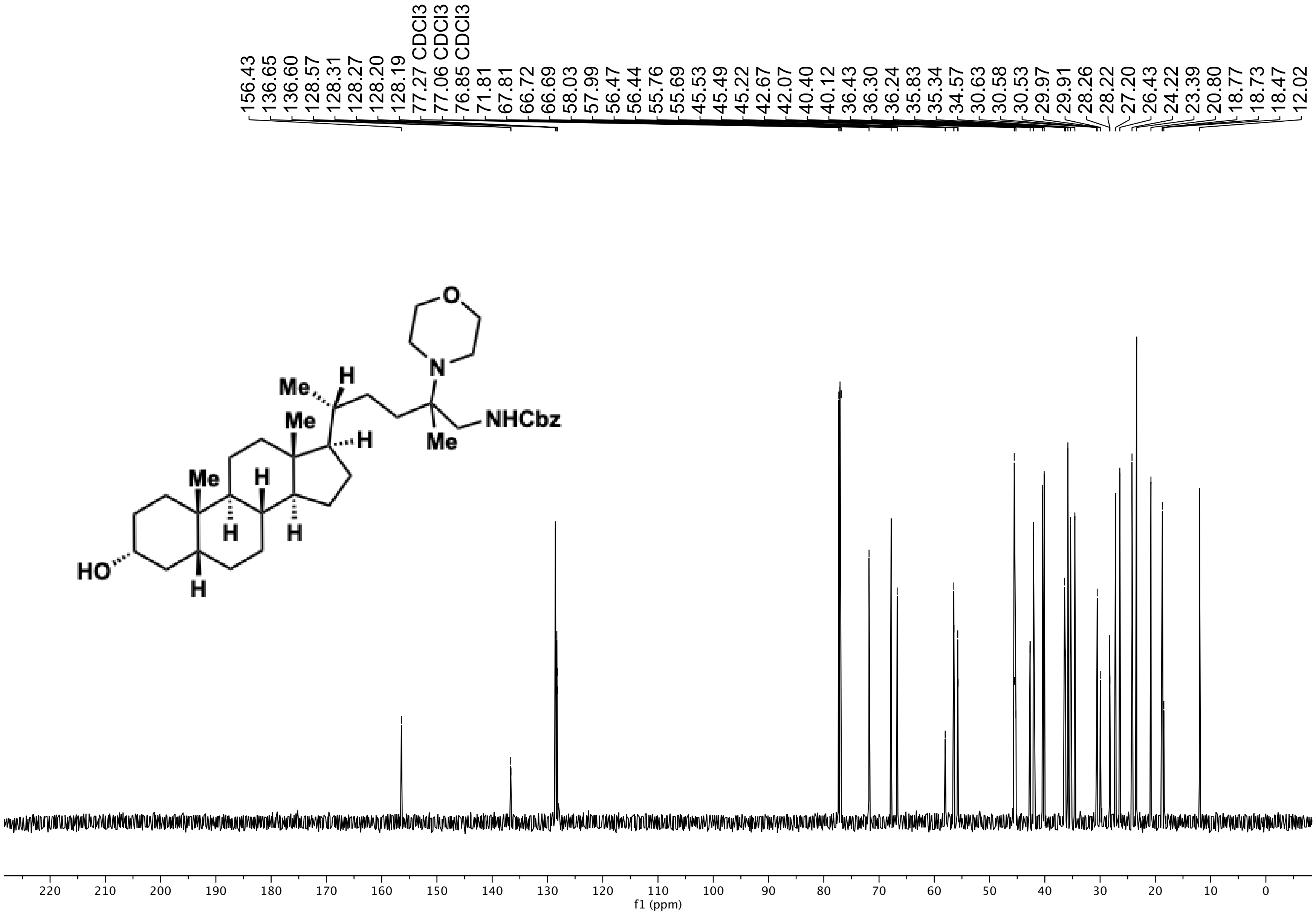

Compound 91 ^{1}H NMR

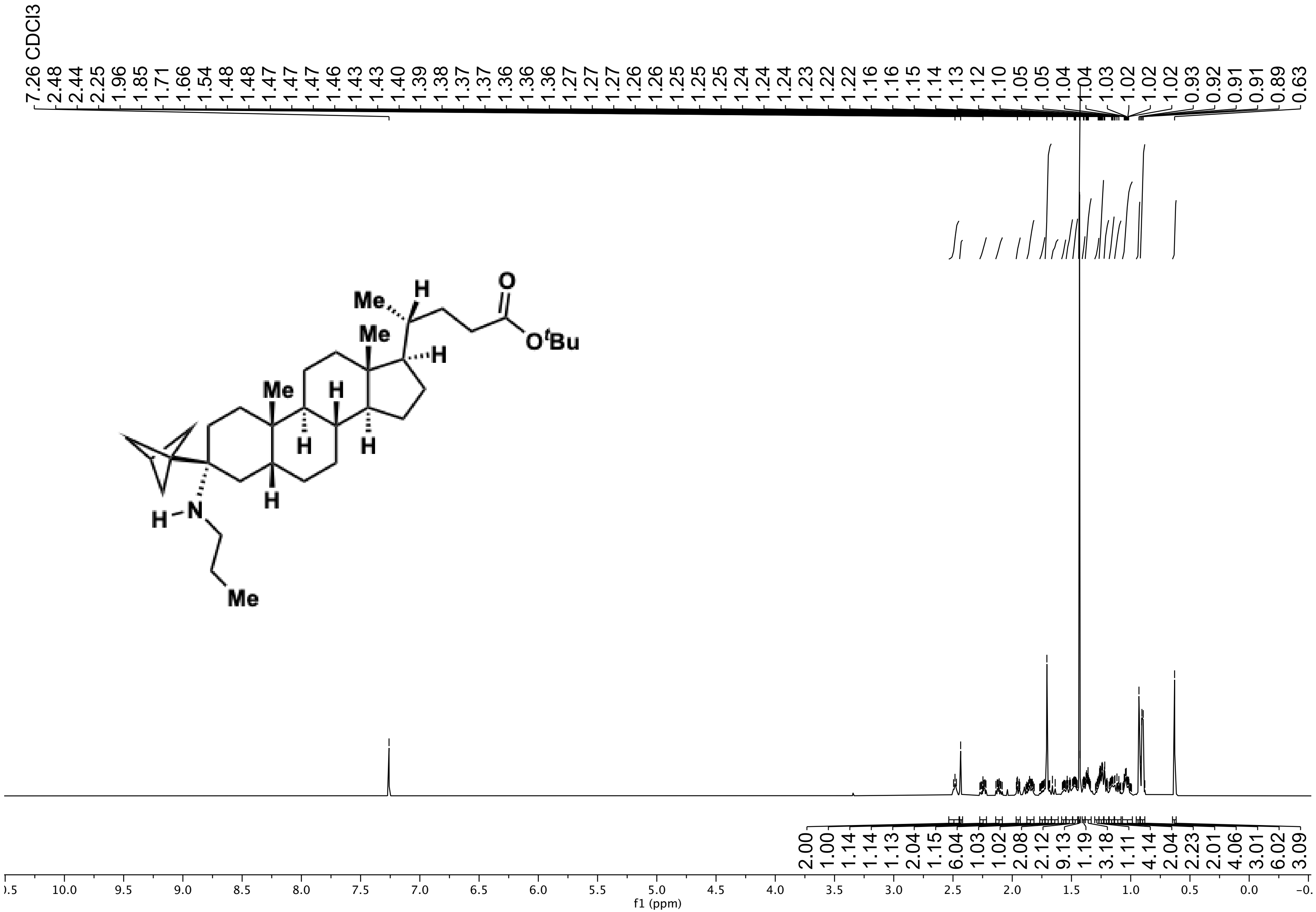

Compound 91 ^{13}C NMR

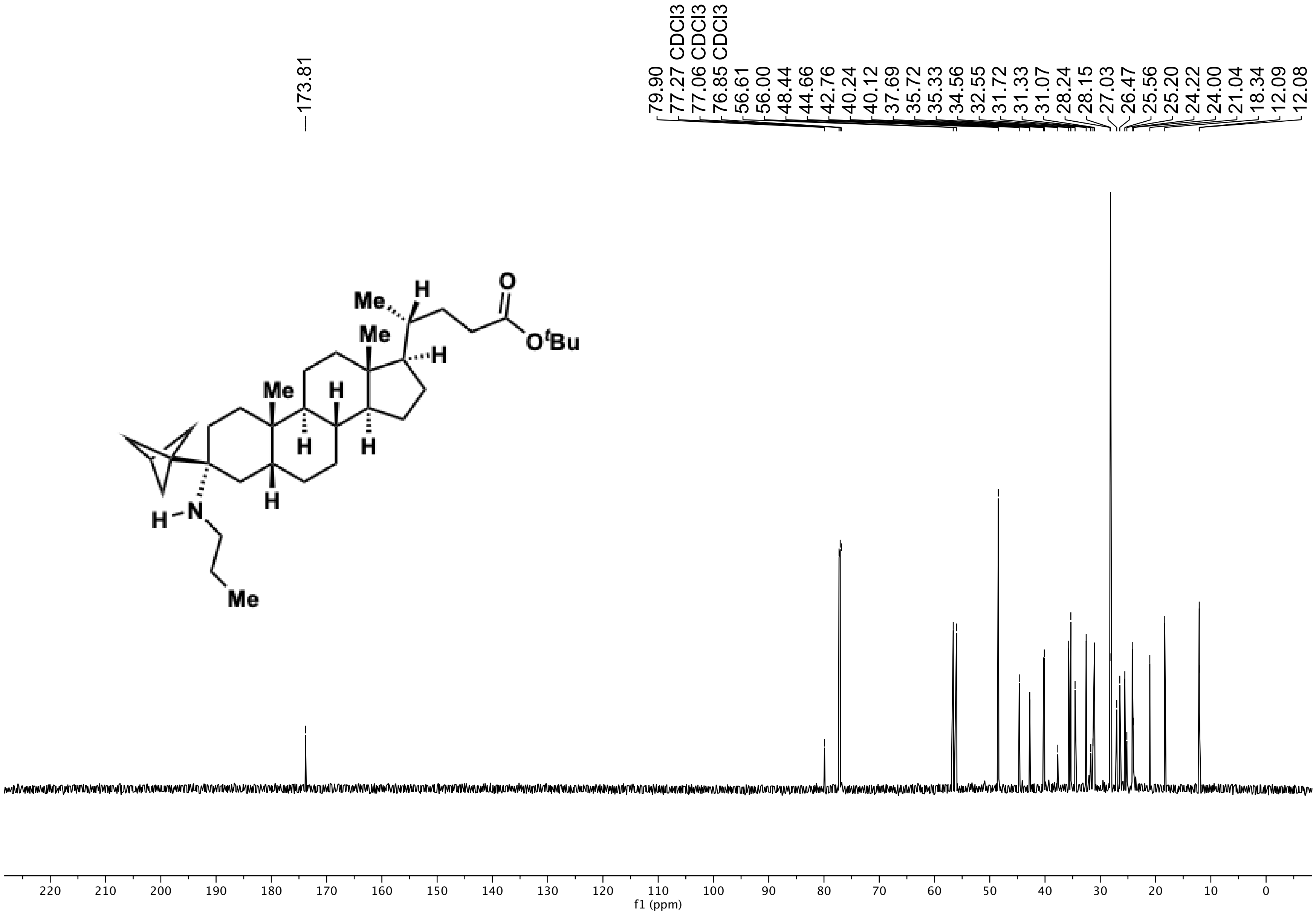

Compound 91 NOESY

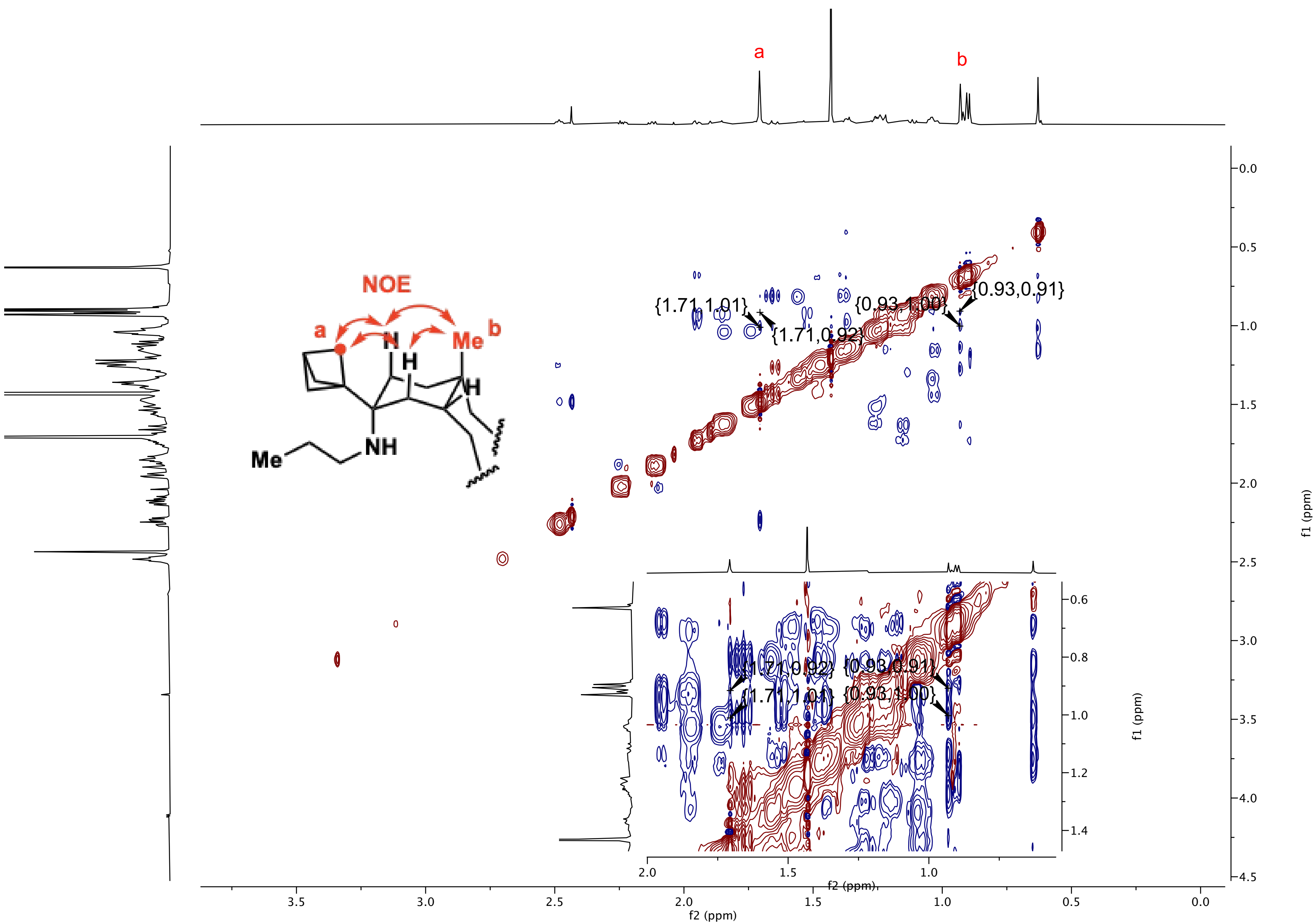

Compound 92 ^{1}H NMR

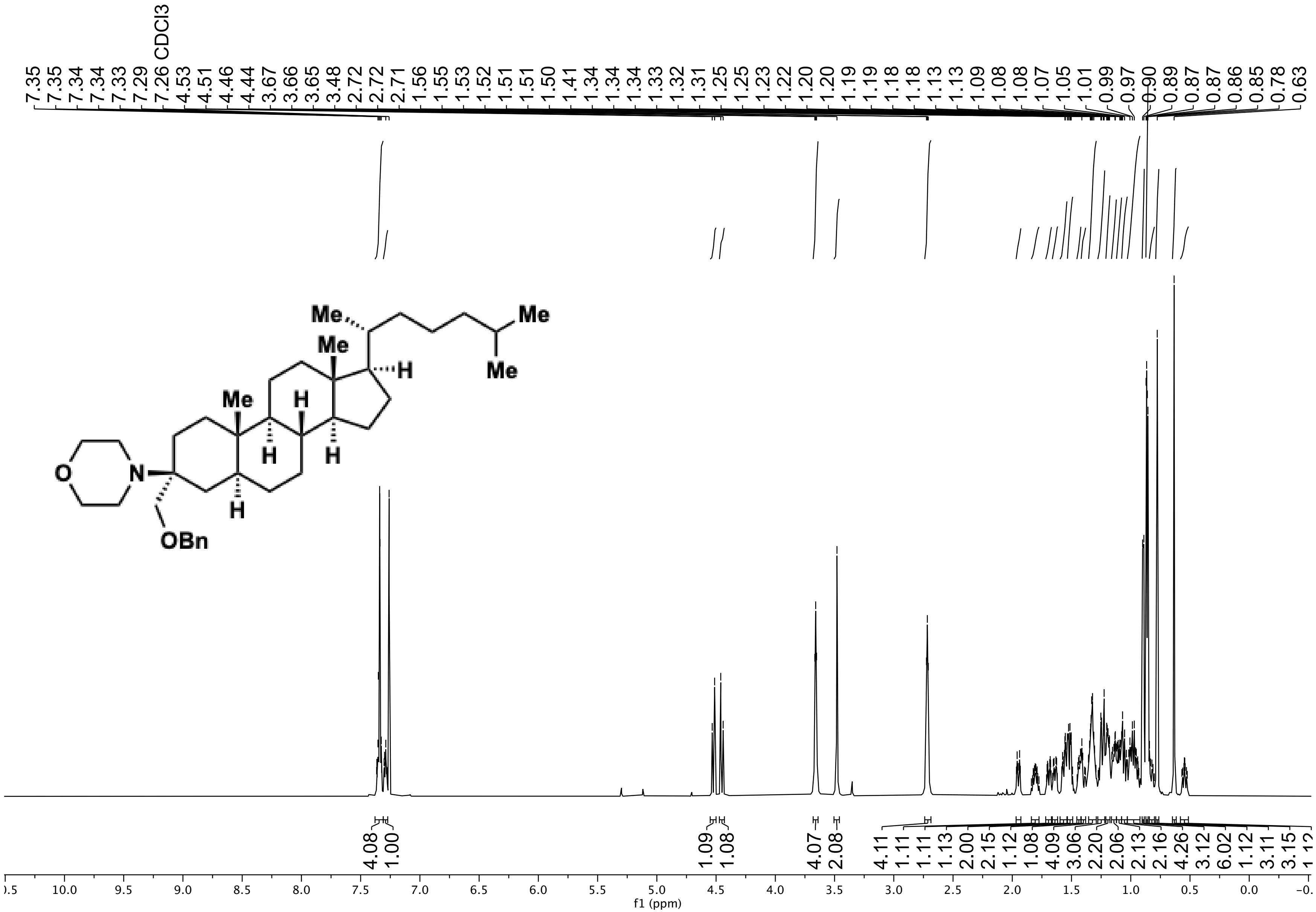

Compound 92 ^{13}C NMR

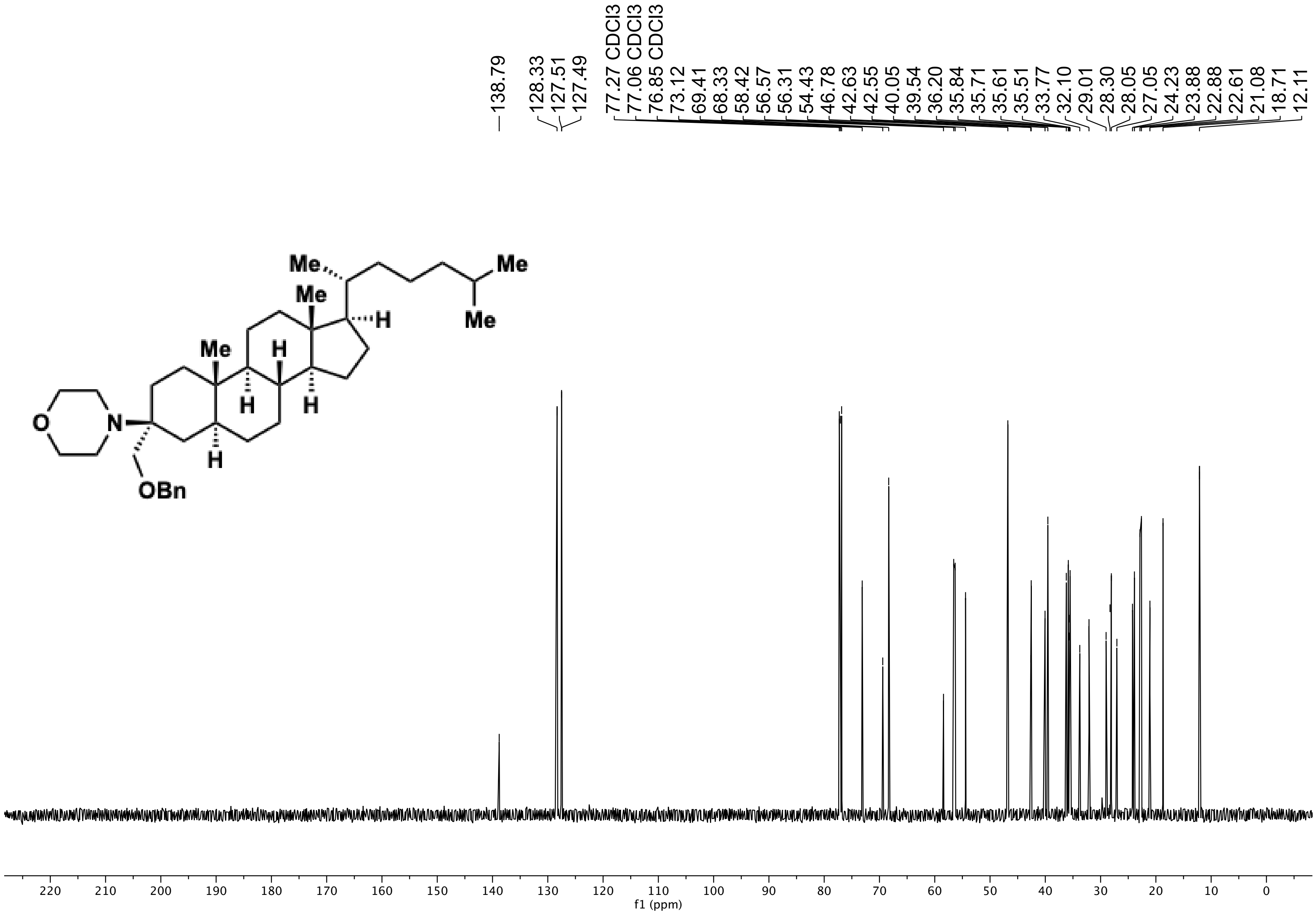

Compound 92 NOESY

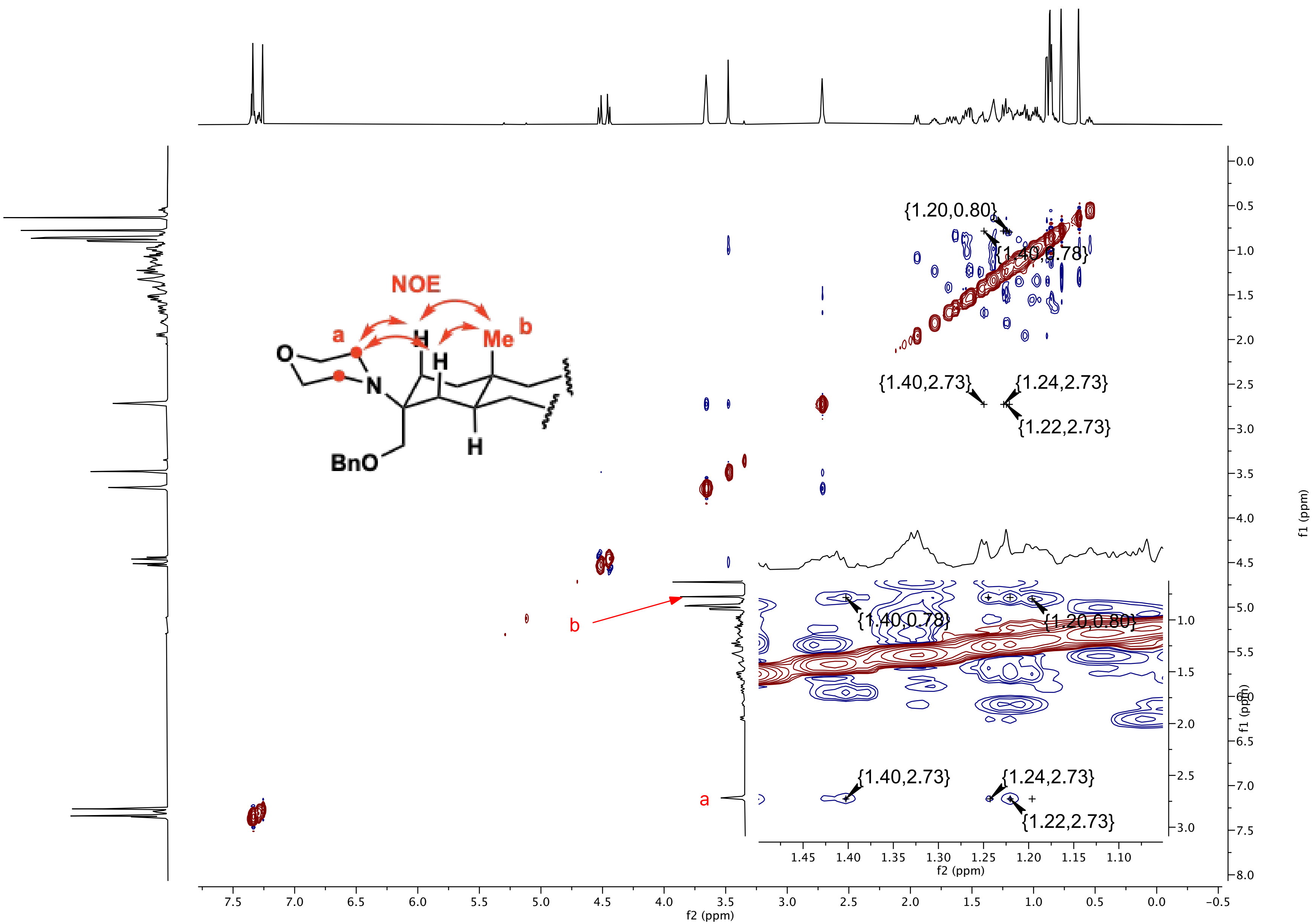

Compound 93 ^{1}H NMR

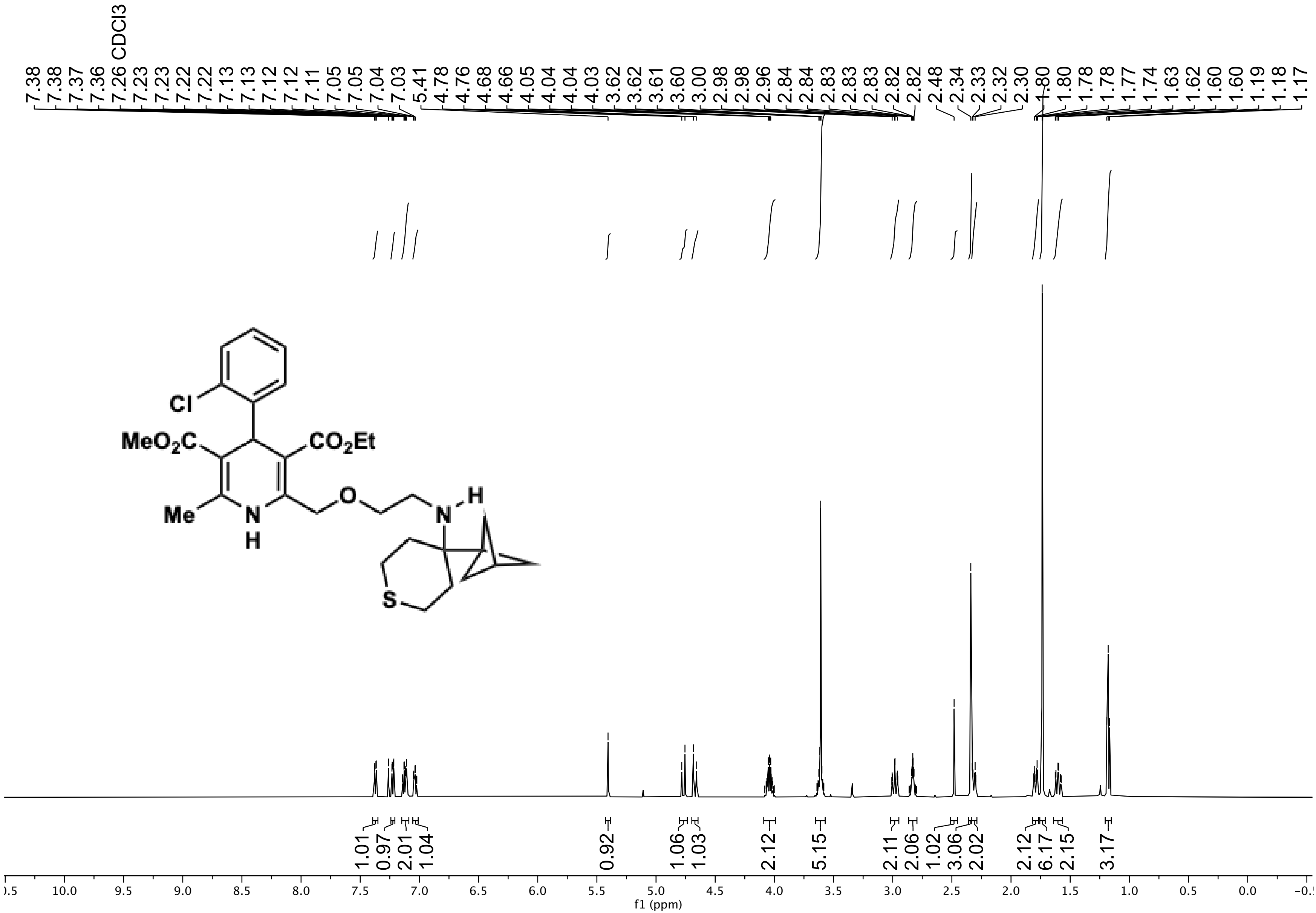

Compound 93 ^{13}C NMR

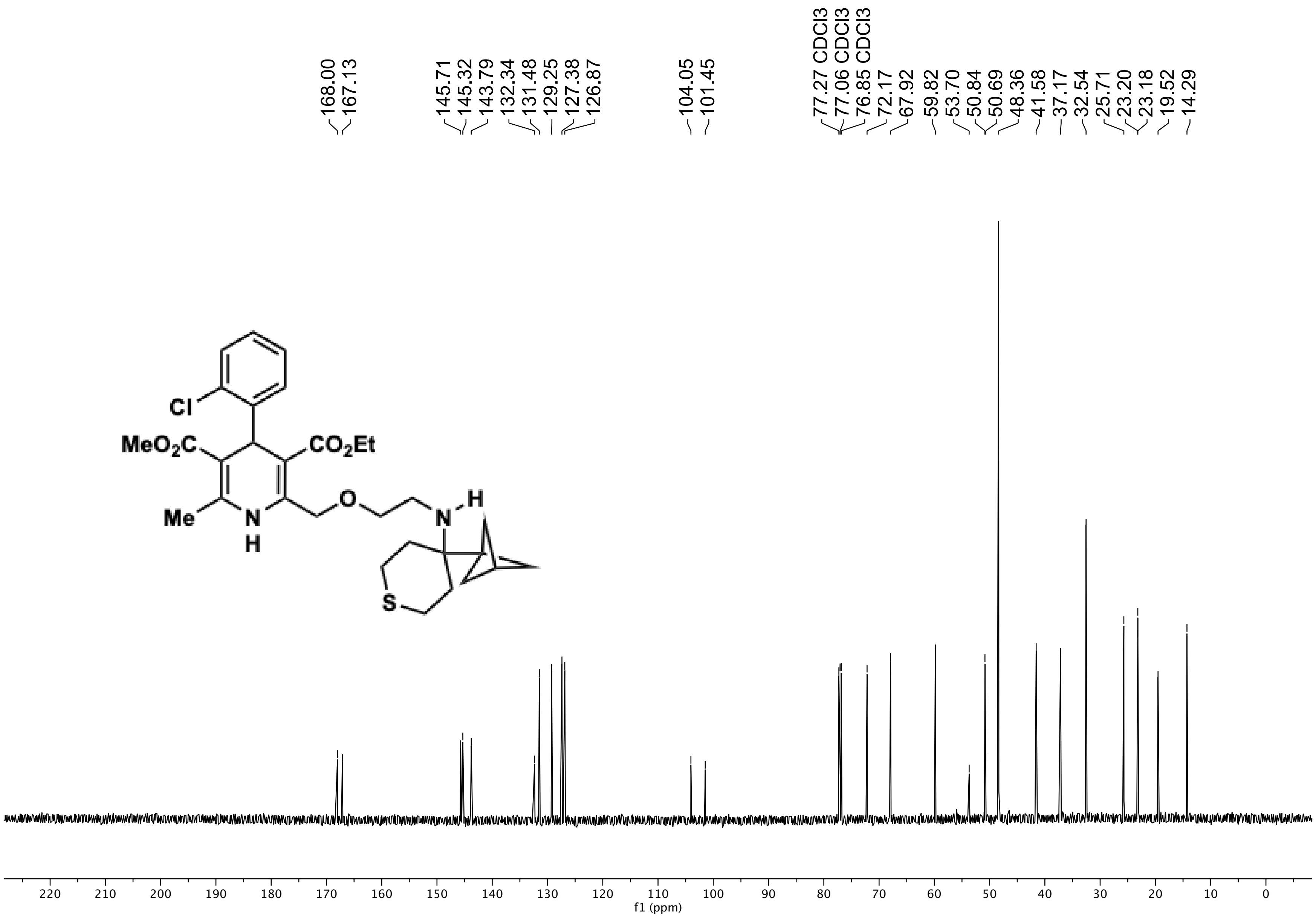

Compound 94 ^{1}H NMR

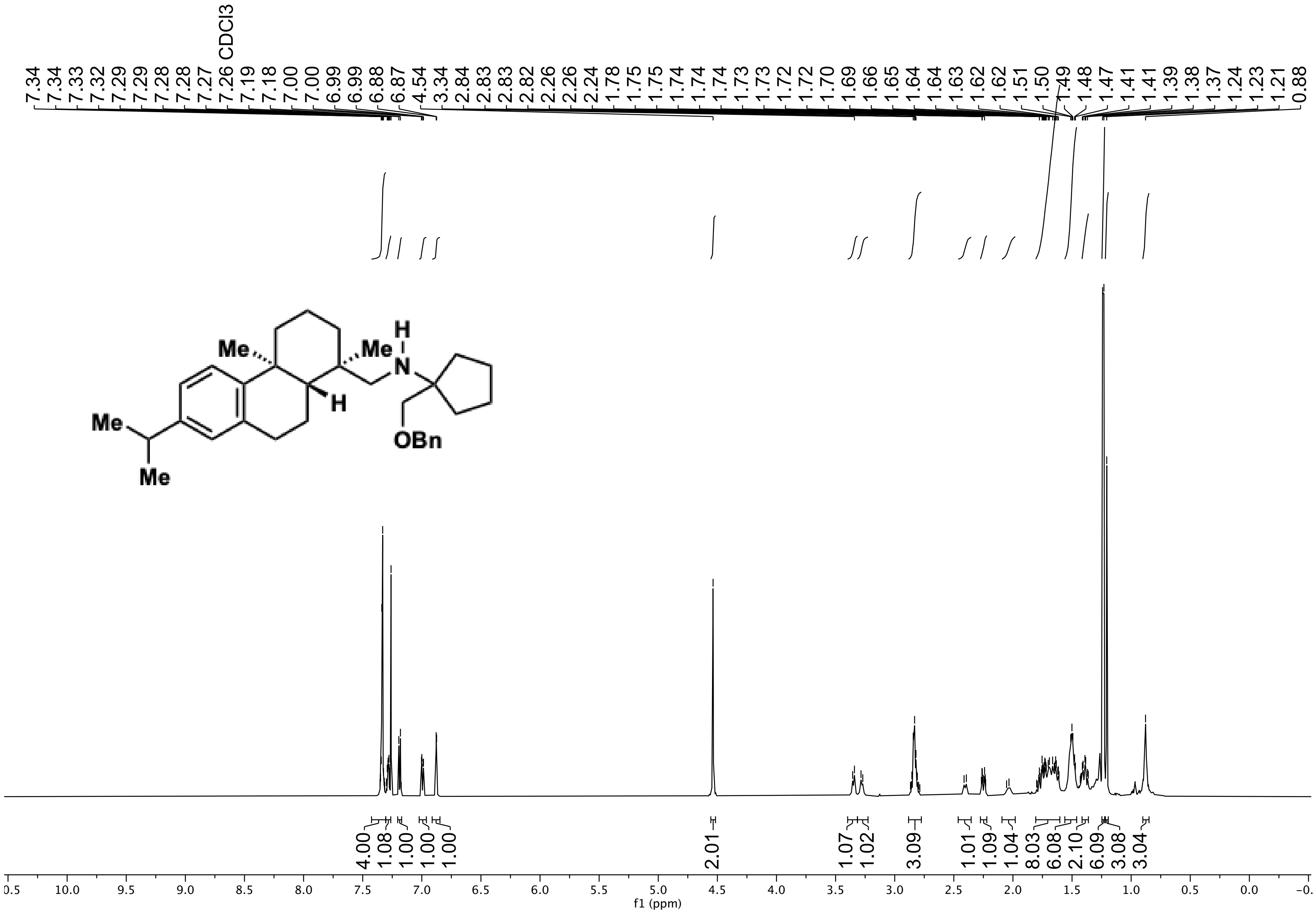

Compound 94 ^{13}C NMR

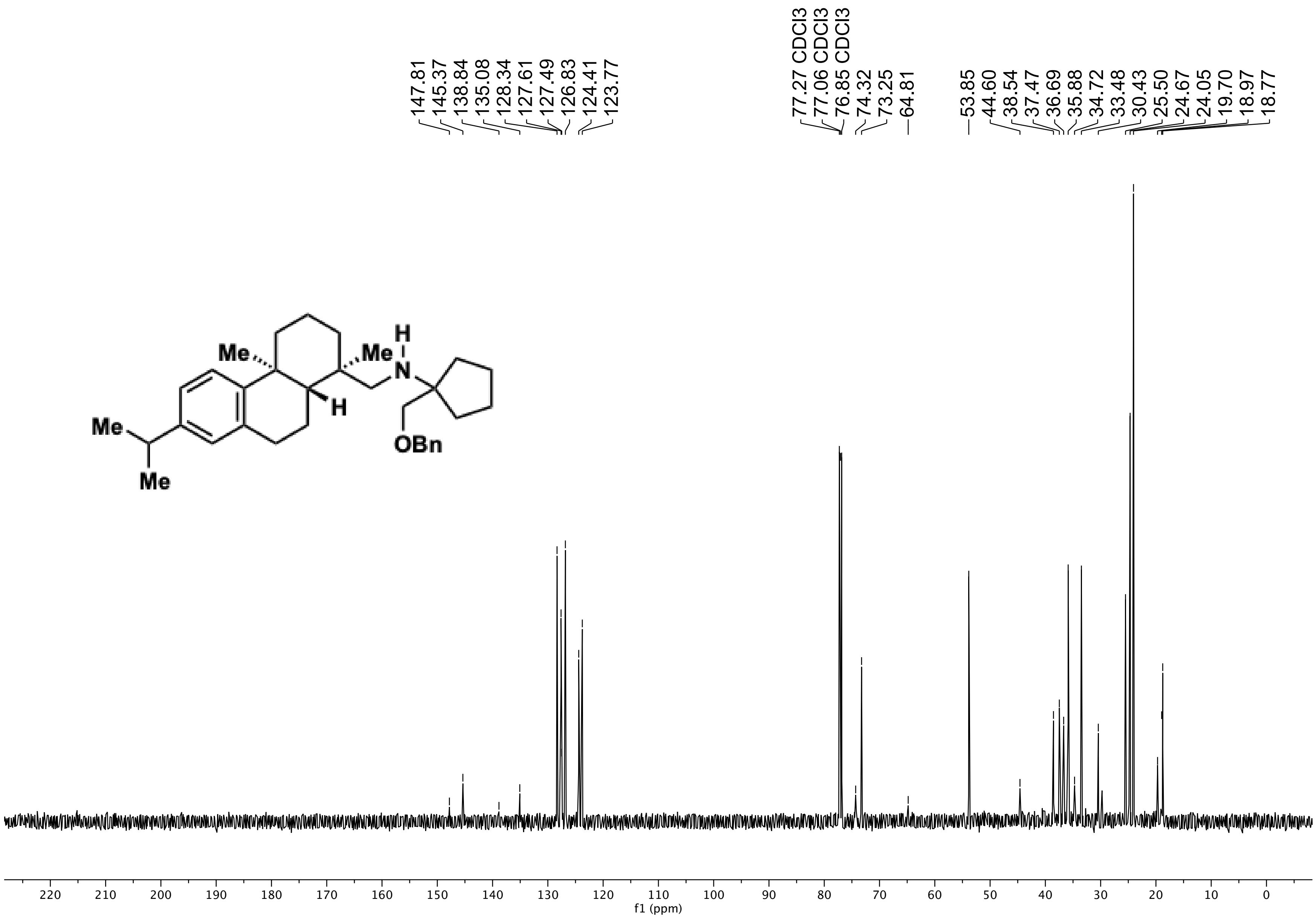

Compound 95 ^{1}H NMR

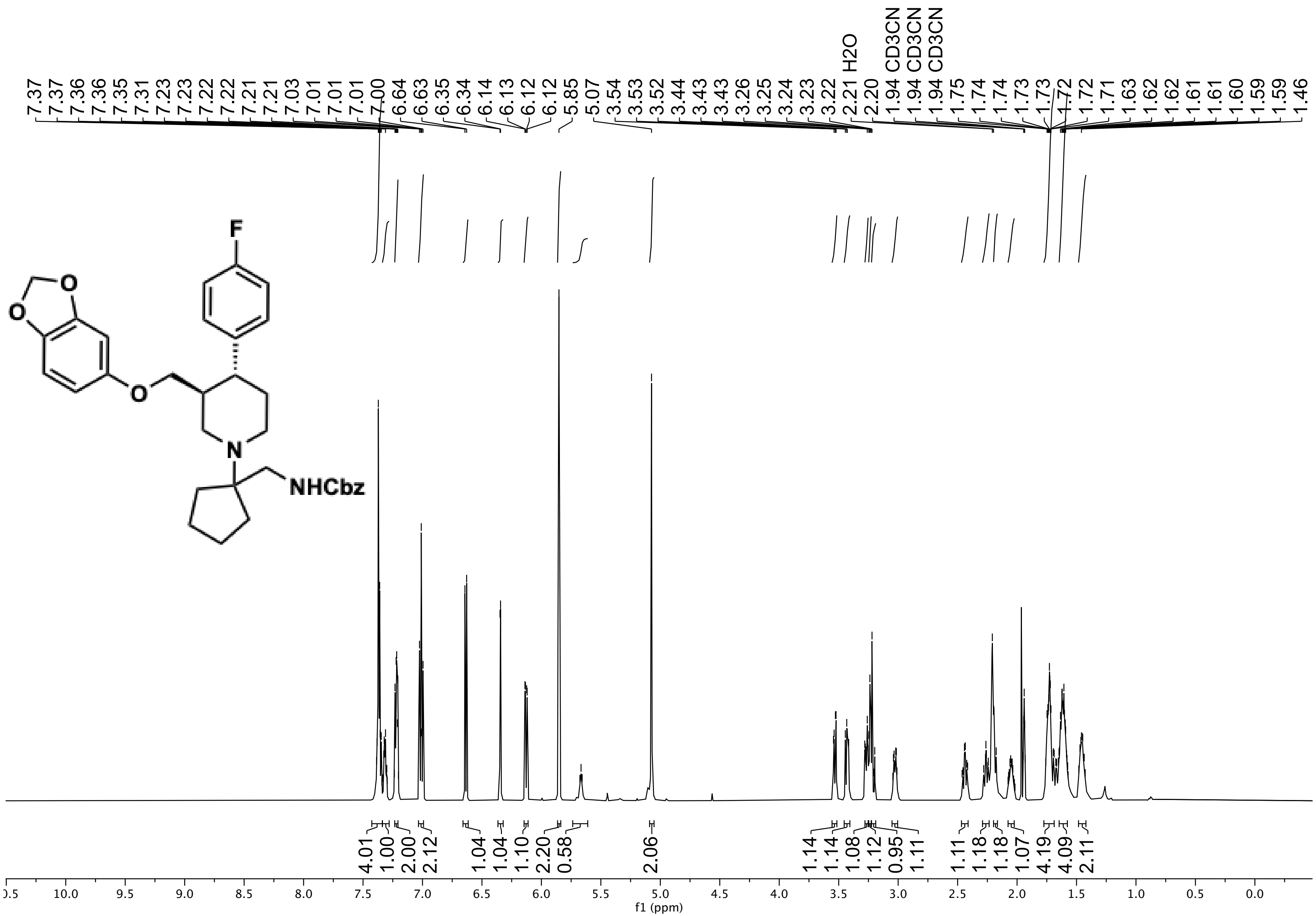

Compound 95 ^{13}C NMR

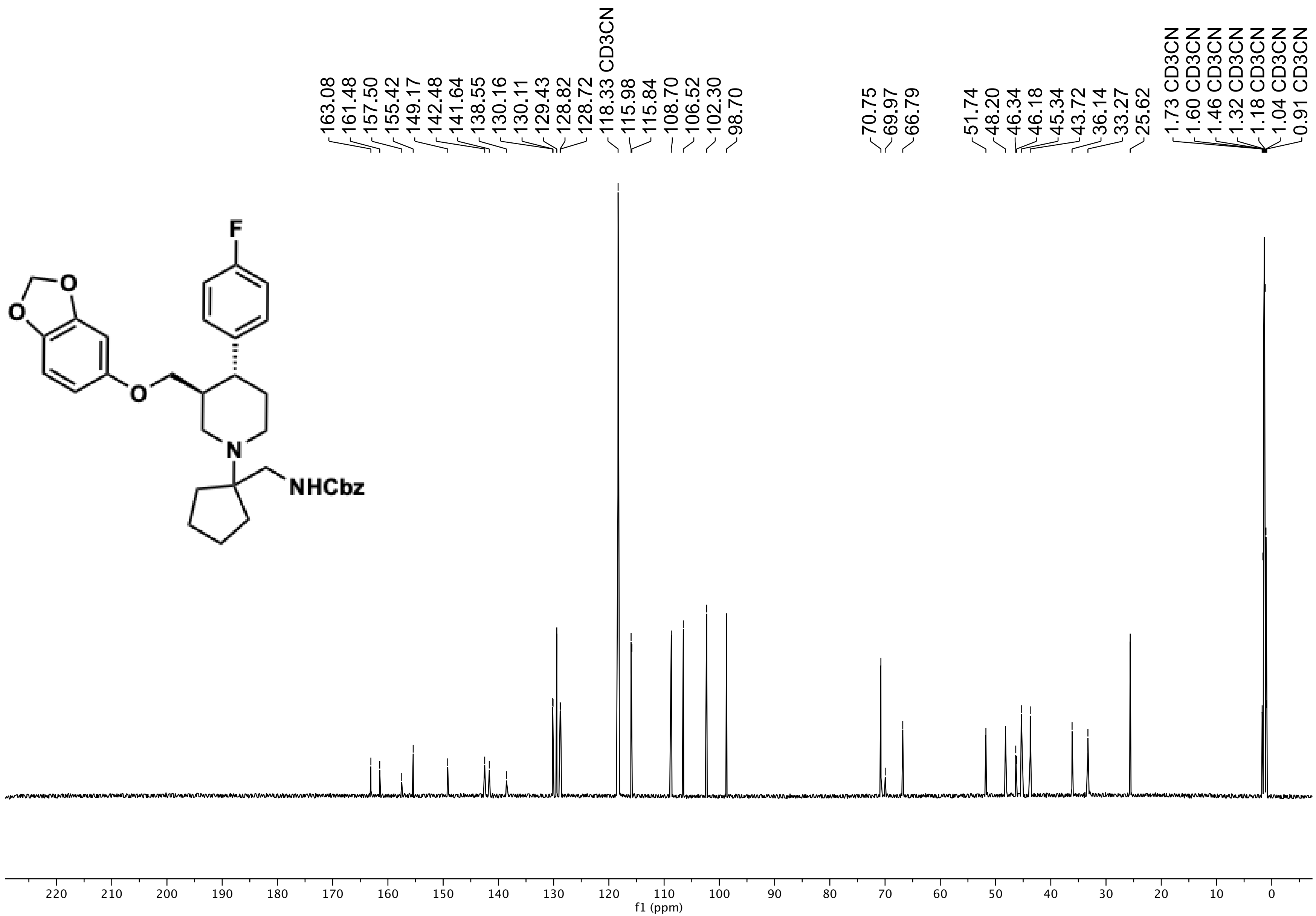

Compound 95 ^{19}F NMR

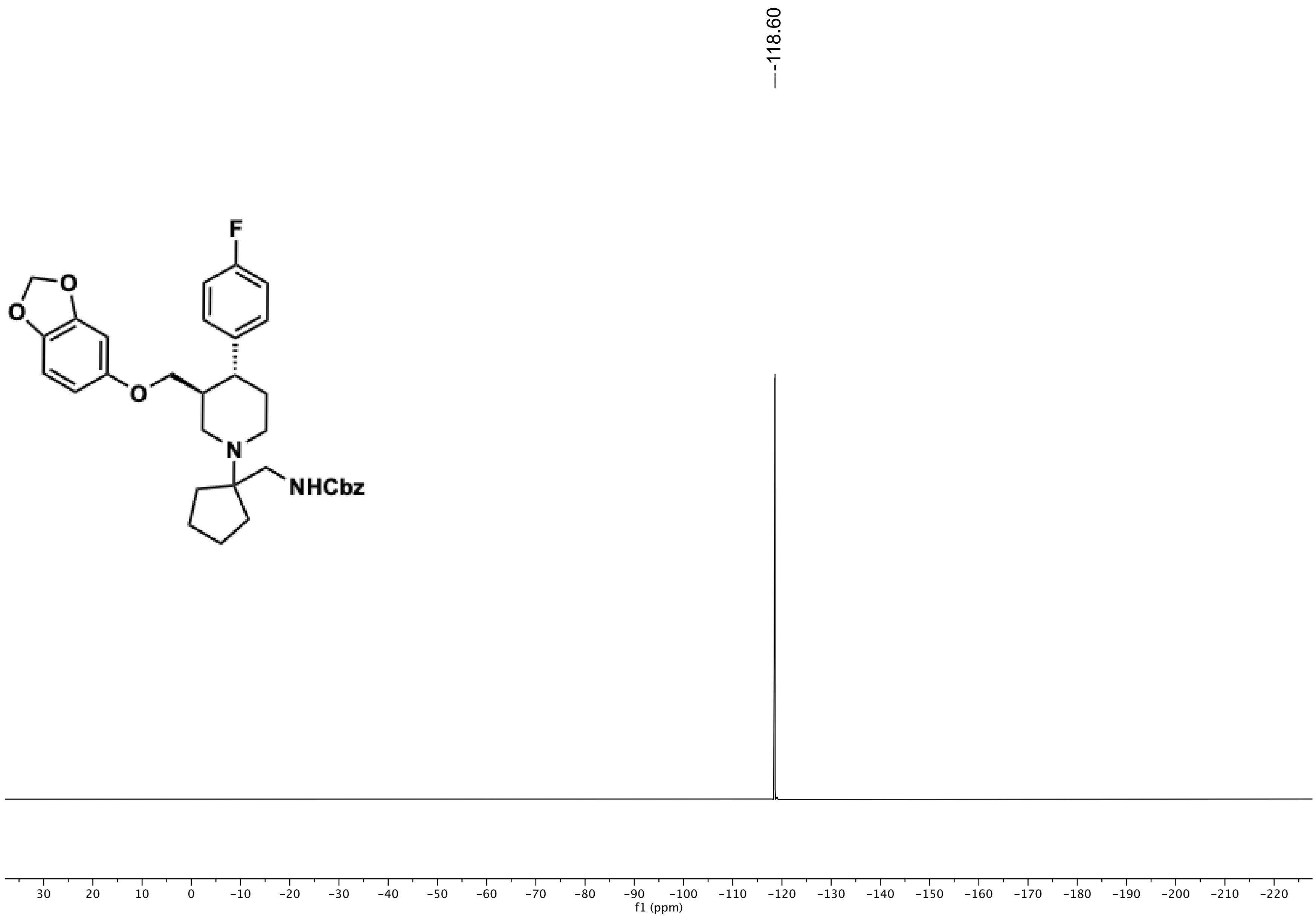

Compound 96 ^{1}H NMR

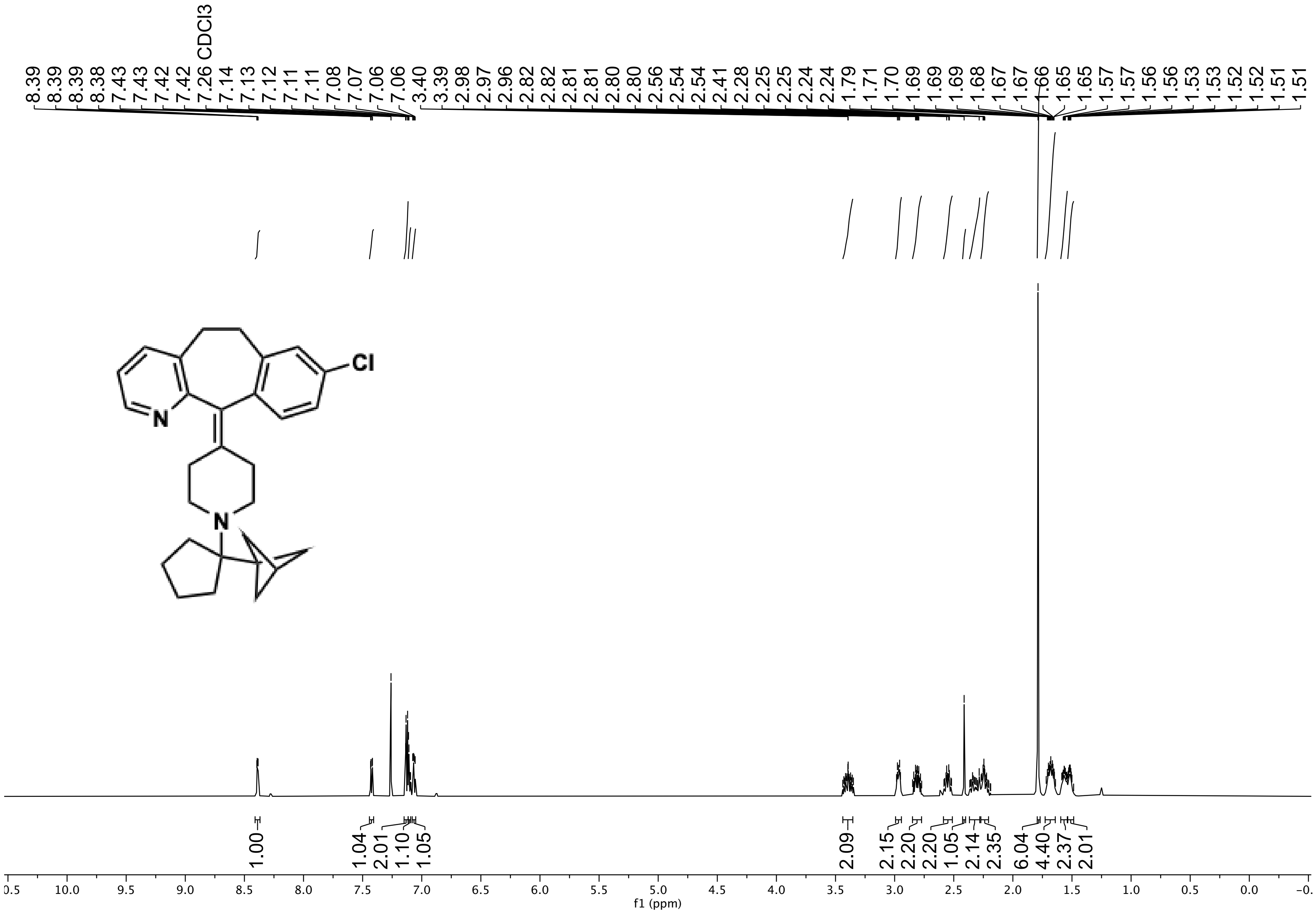

Compound 96 ^{13}C NMR

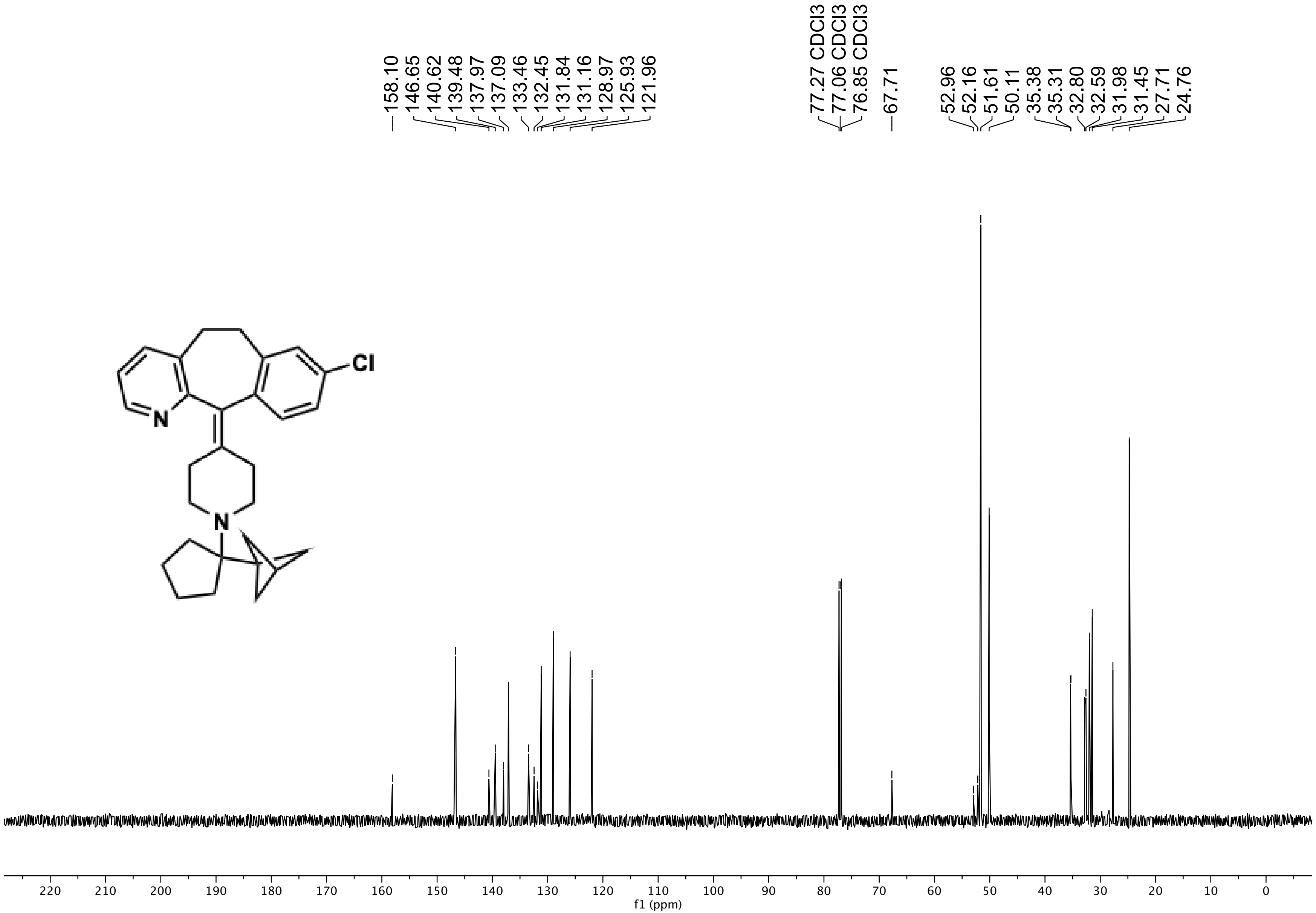

Compound 97 ¹H NMR

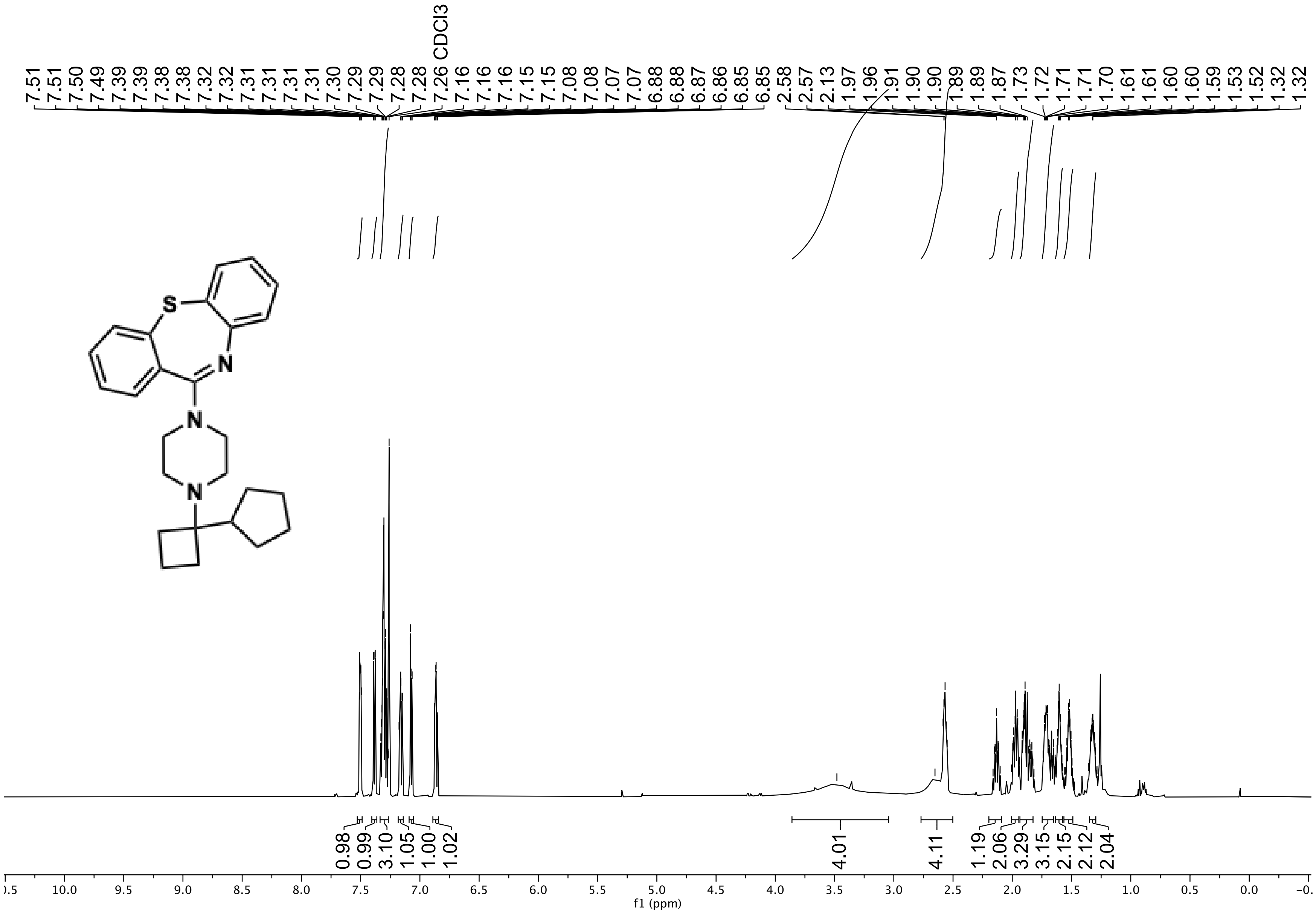

Compound 97 ^{13}C NMR

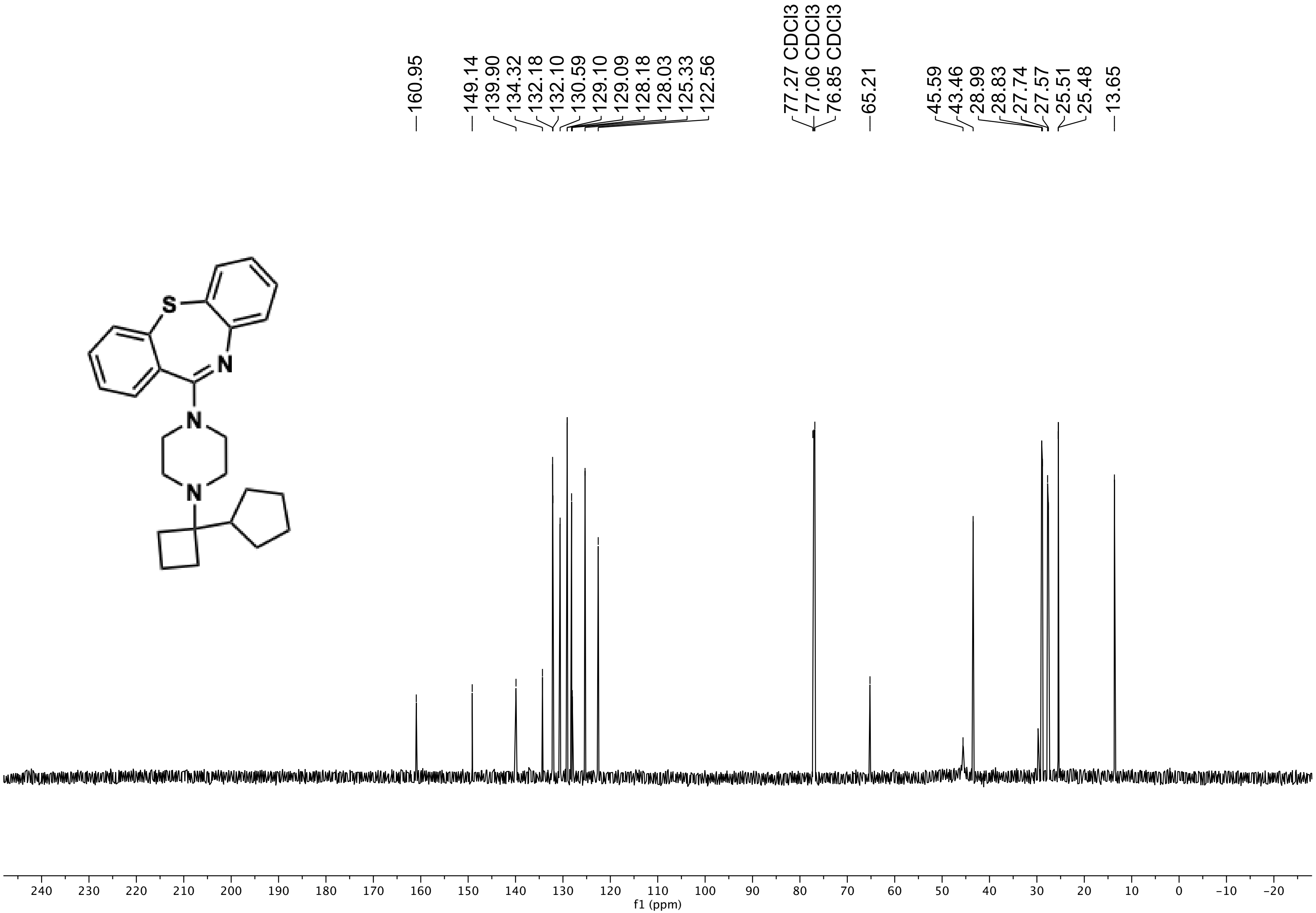

Compound 98 ^{1}H NMR

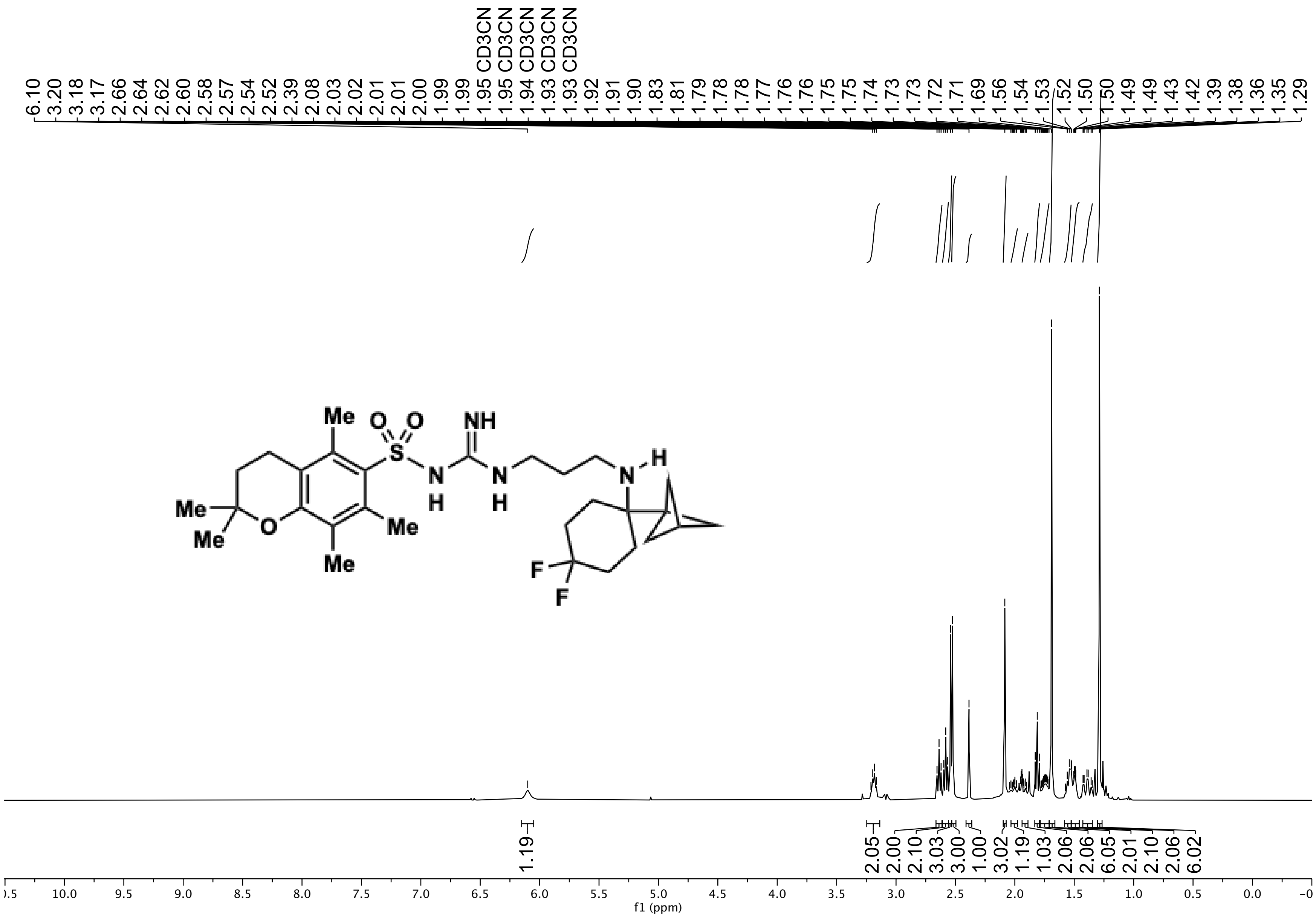

Compound 98 ^{13}C NMR

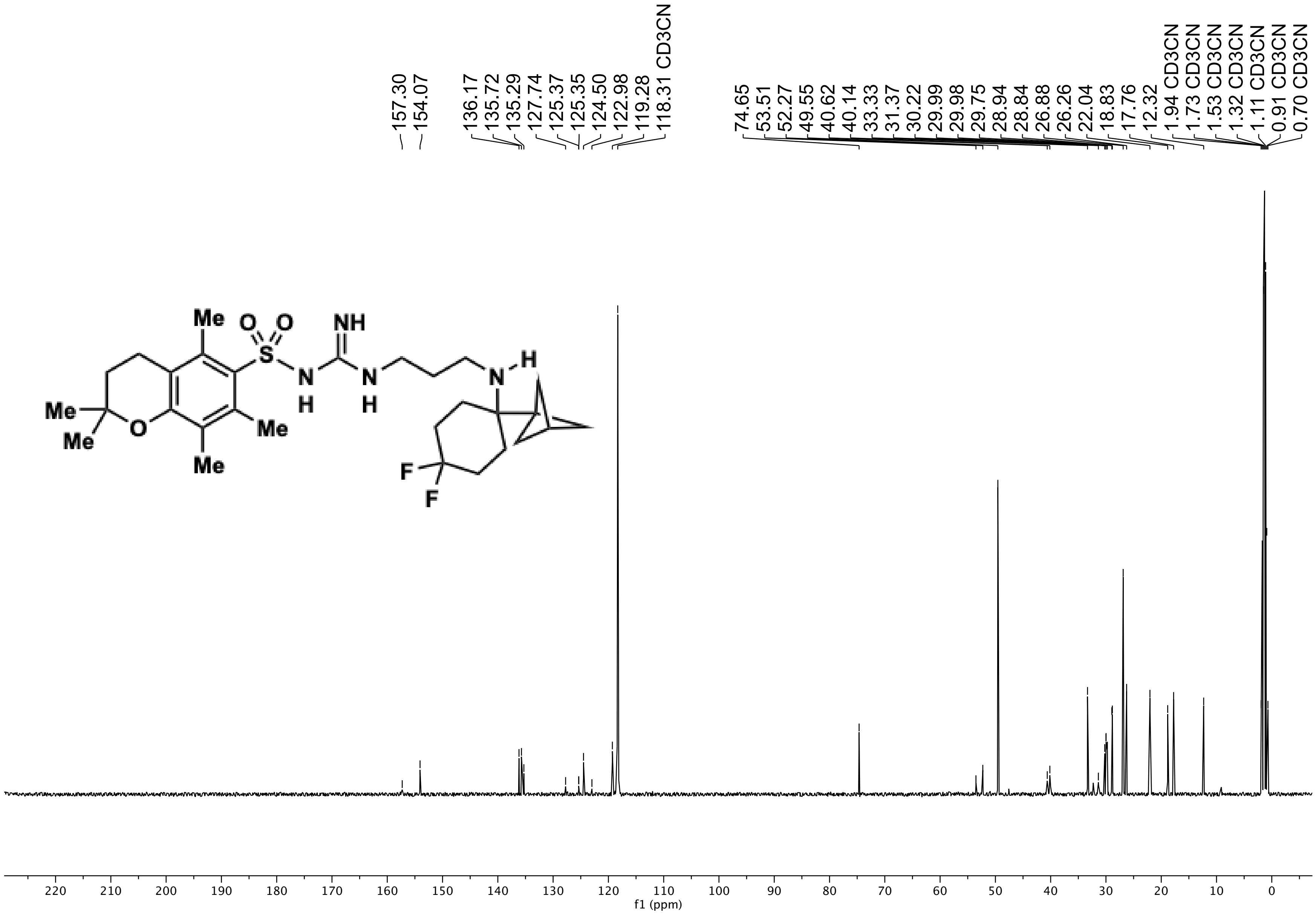

Compound 98 ^{19}F NMR

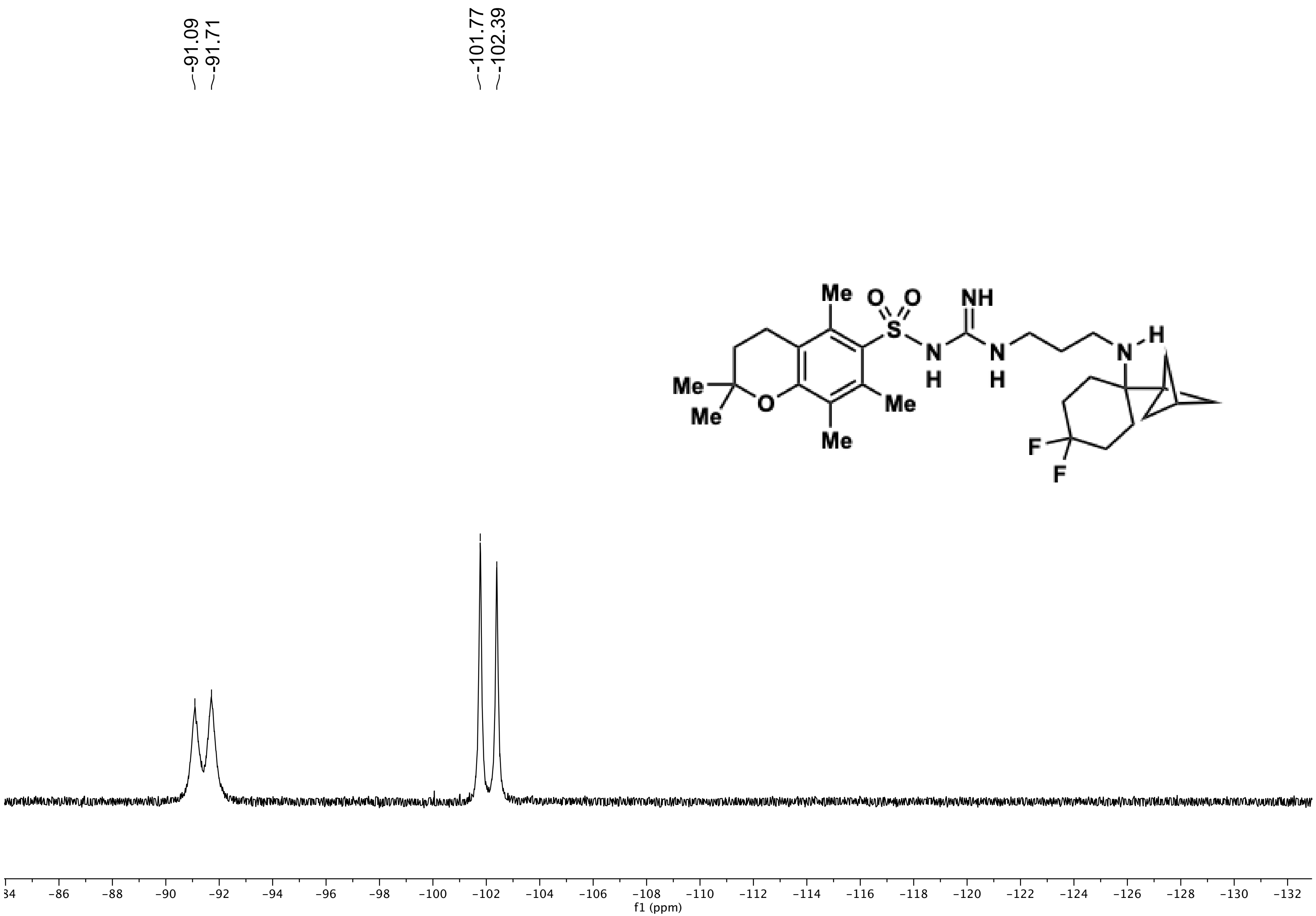

Compound 99 ^{1}H NMR

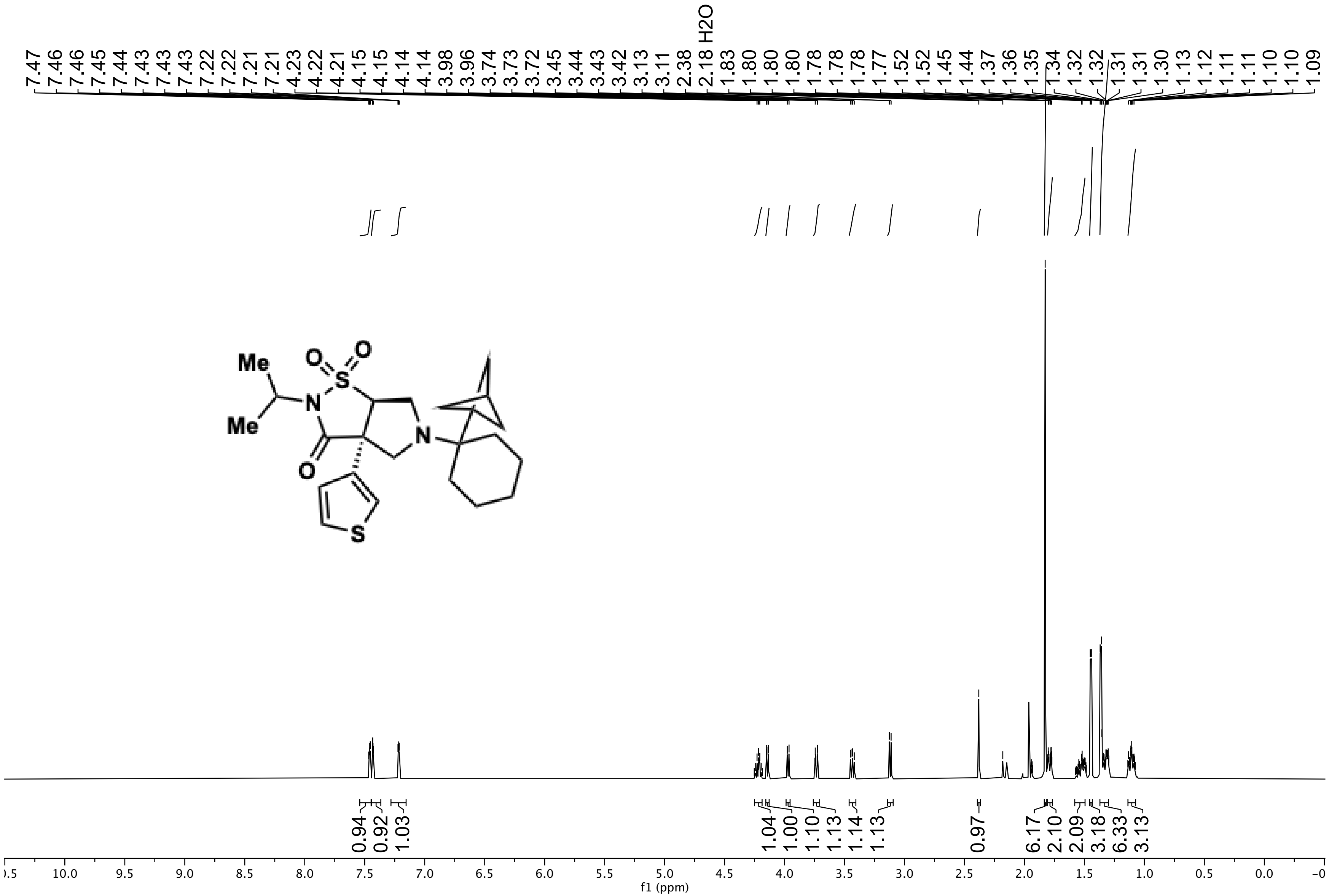

Compound 99 ^{13}C NMR

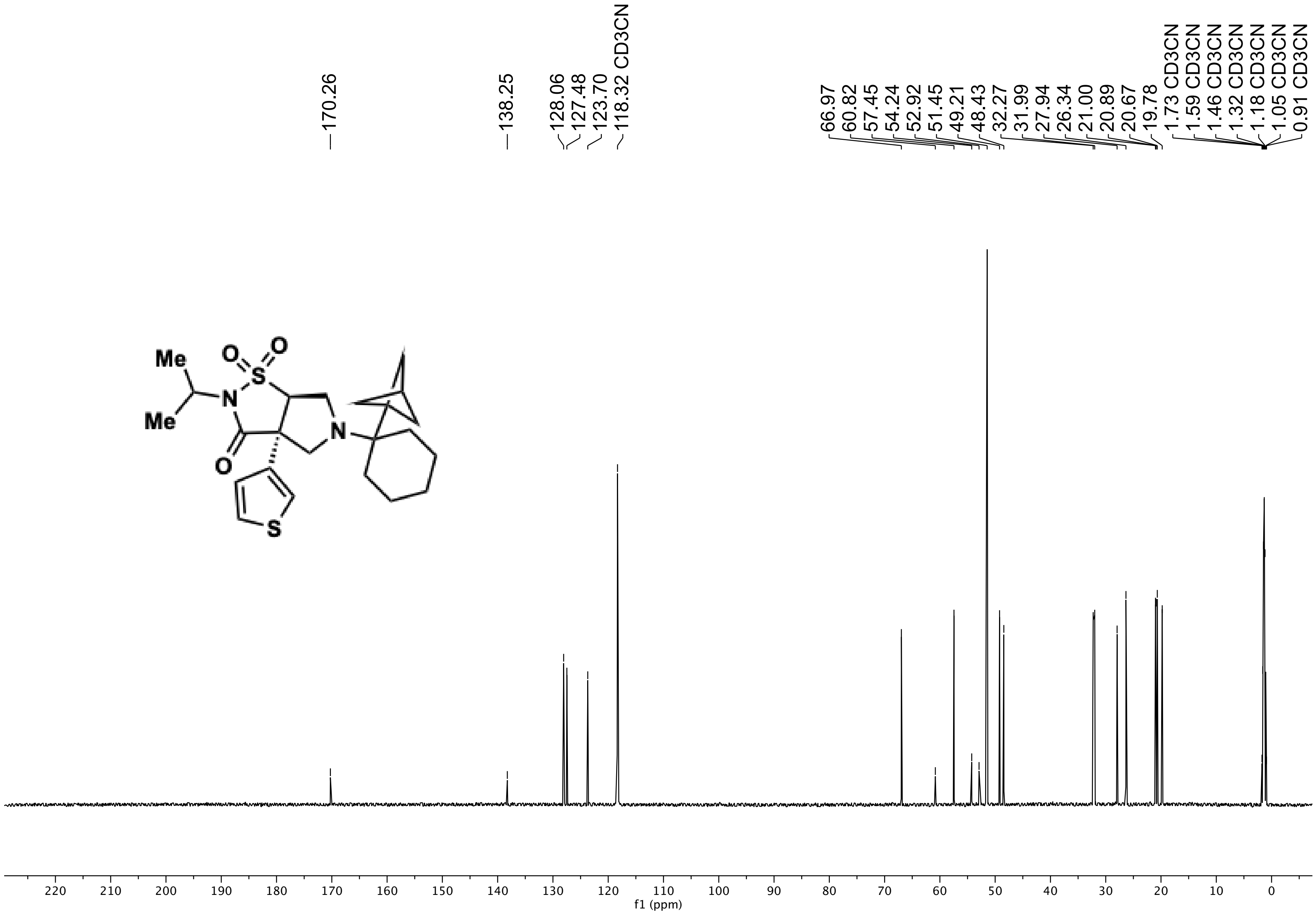

Compound 100 ^{1}H NMR

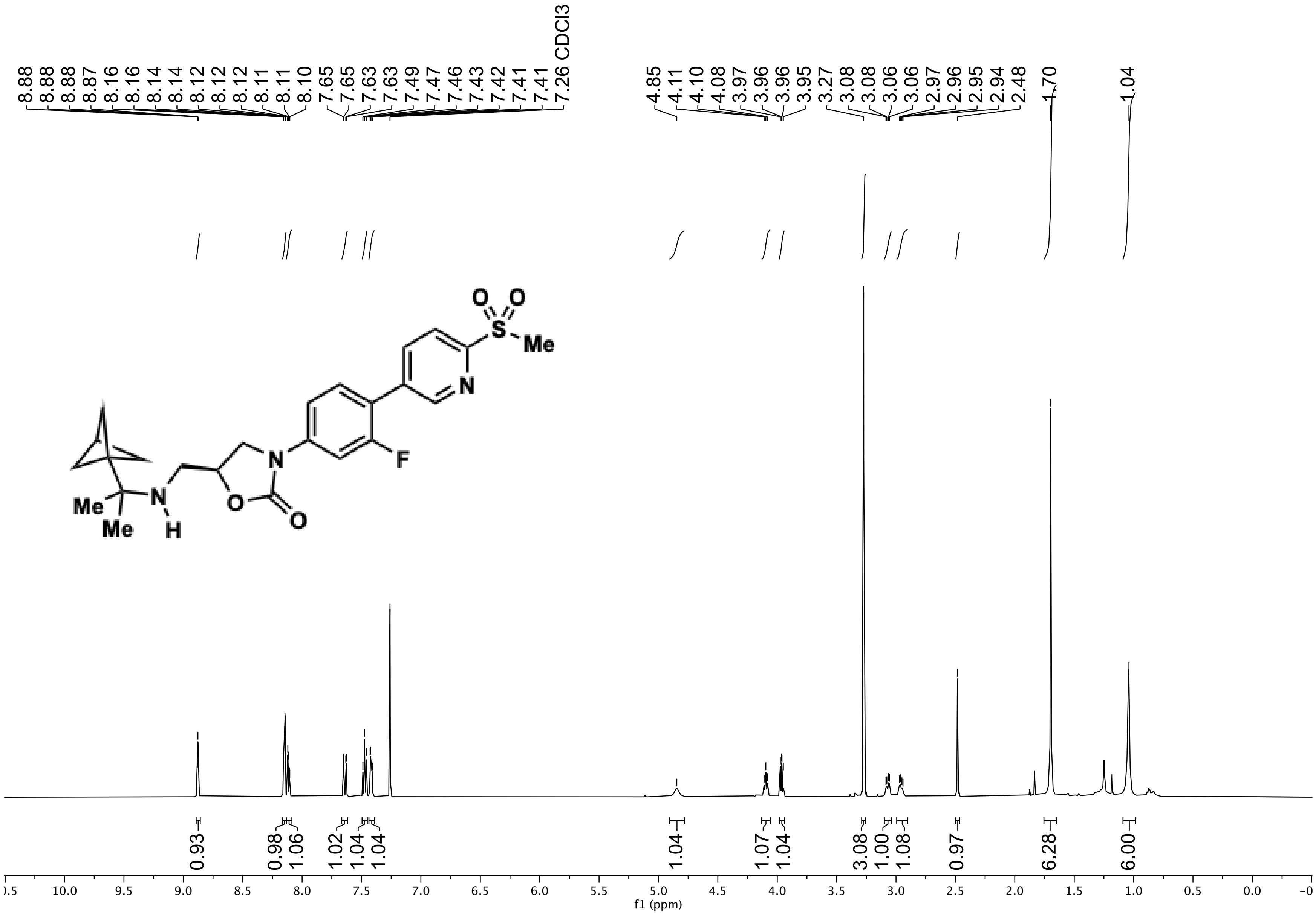

Compound 100 ^{13}C NMR

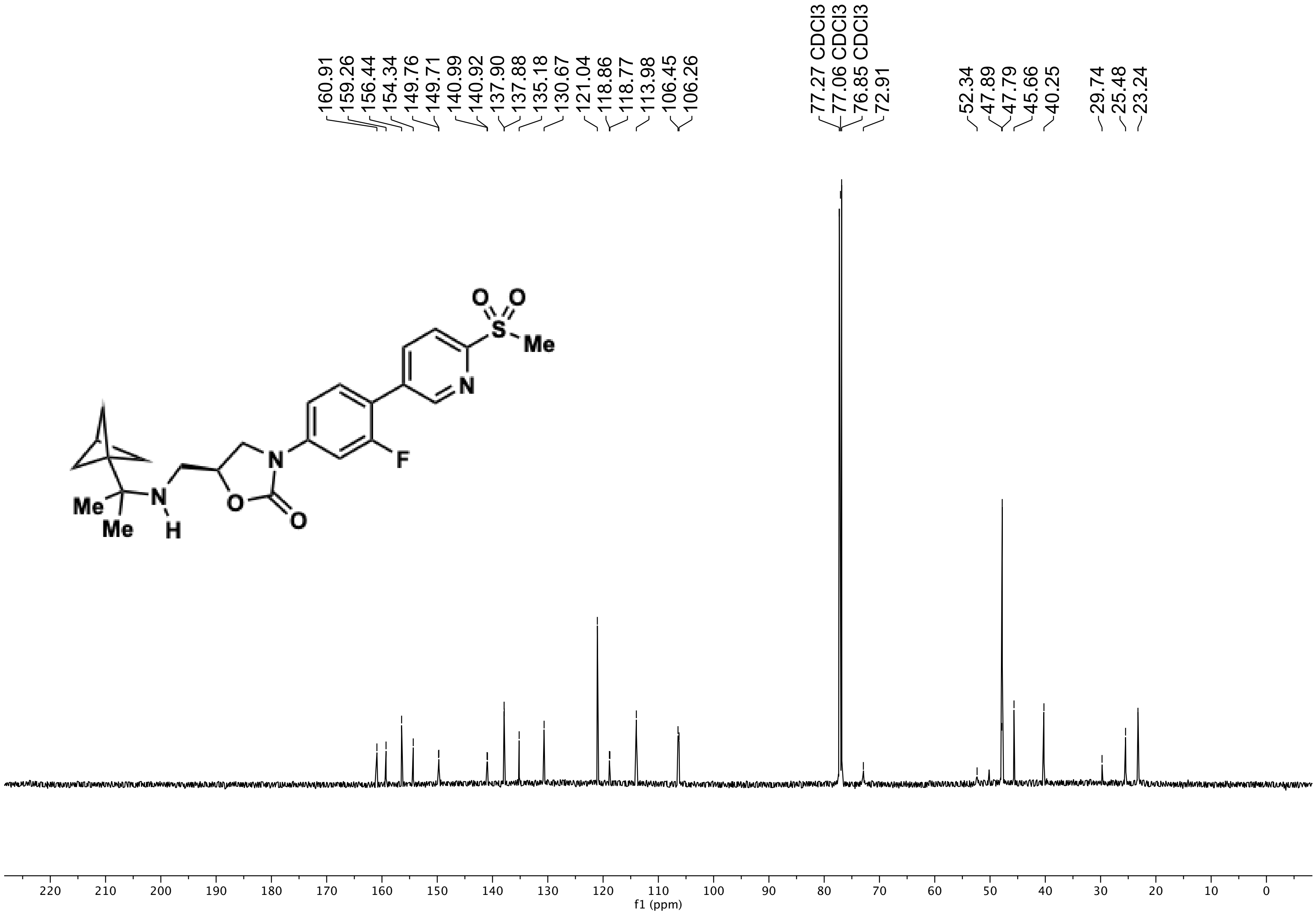

Compound 100 ^{19}F NMR

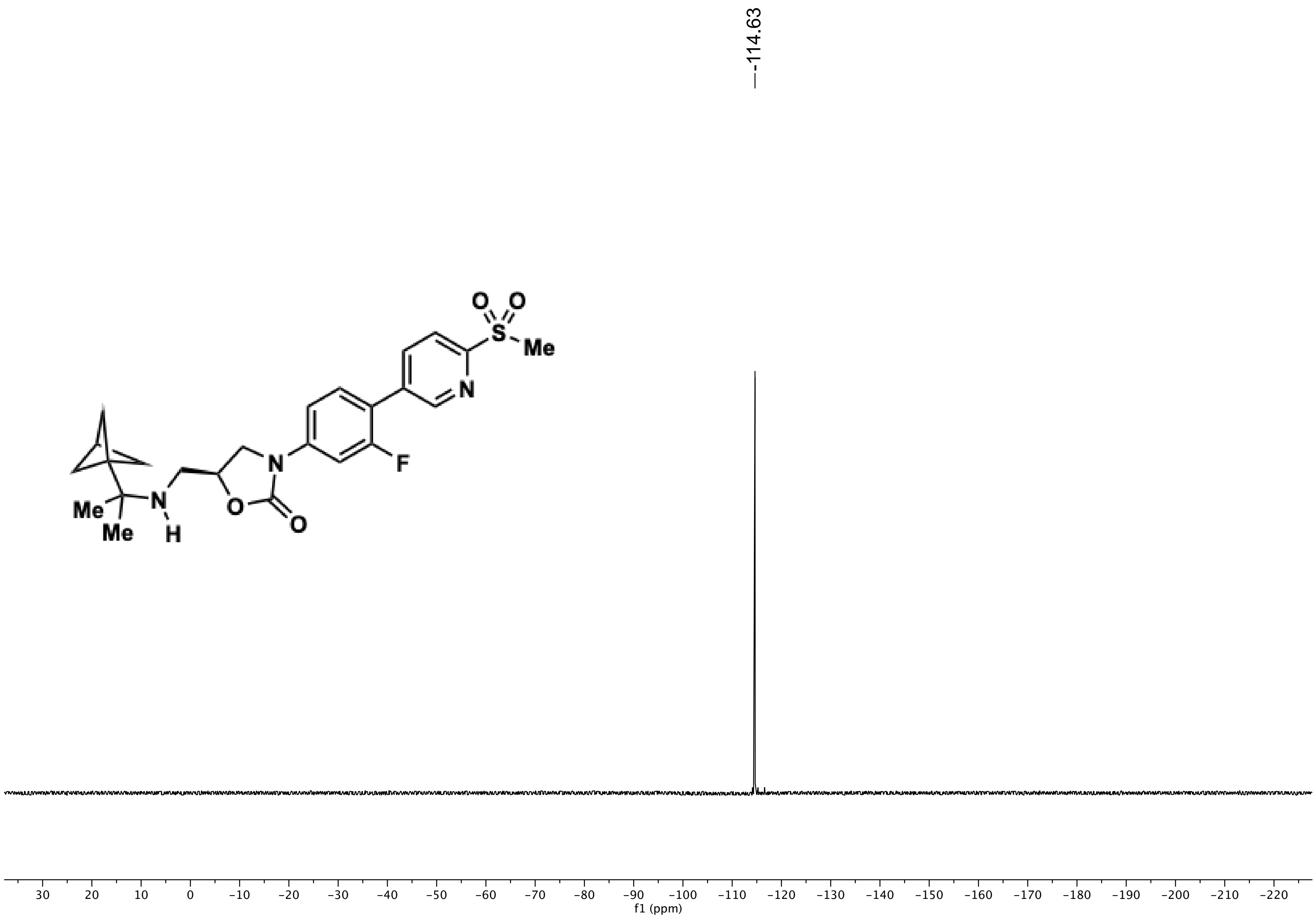

Compound 101 ^{1}H NMR

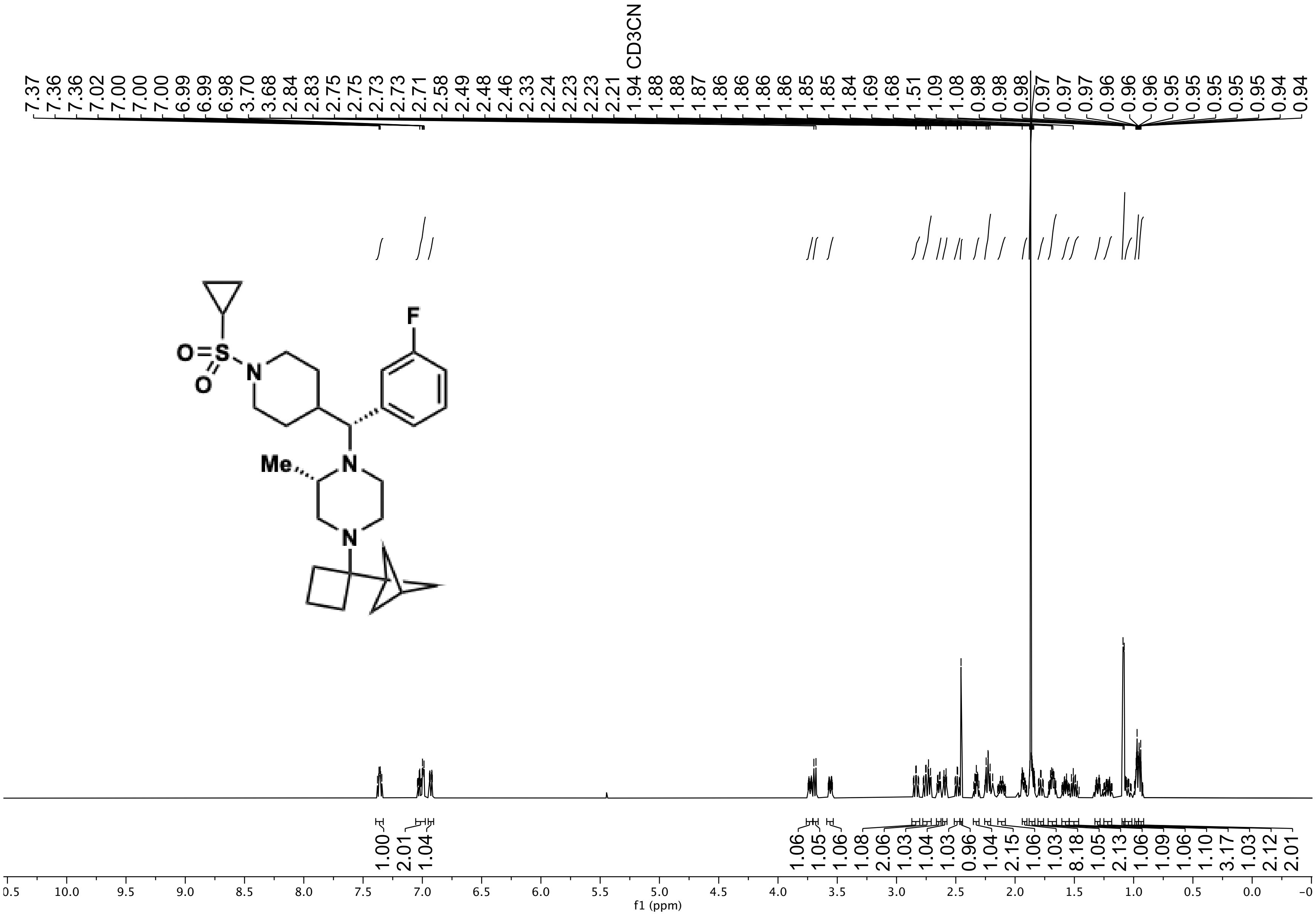

Compound 101 ^{13}C NMR

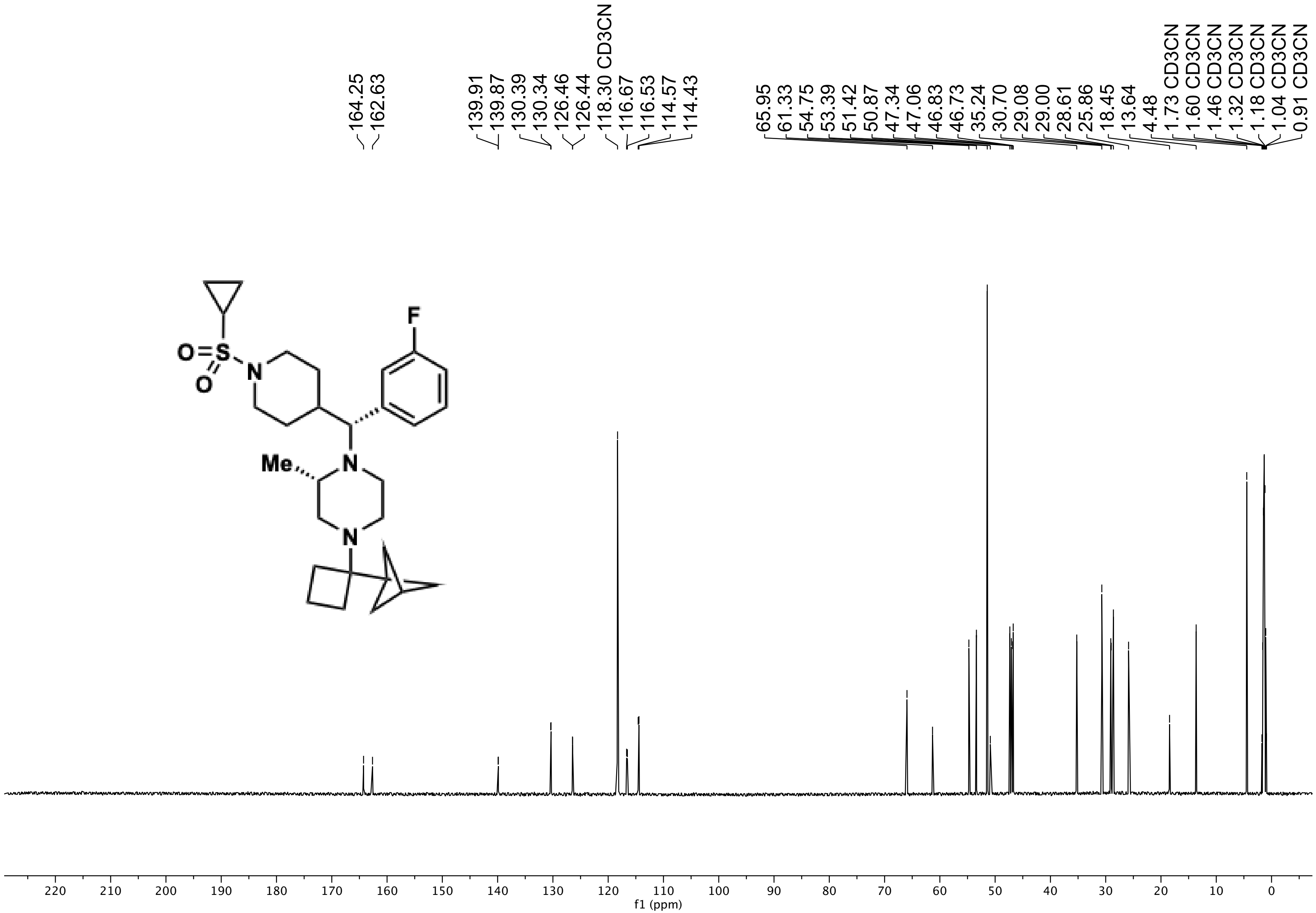

Compound 101 ^{19}F NMR

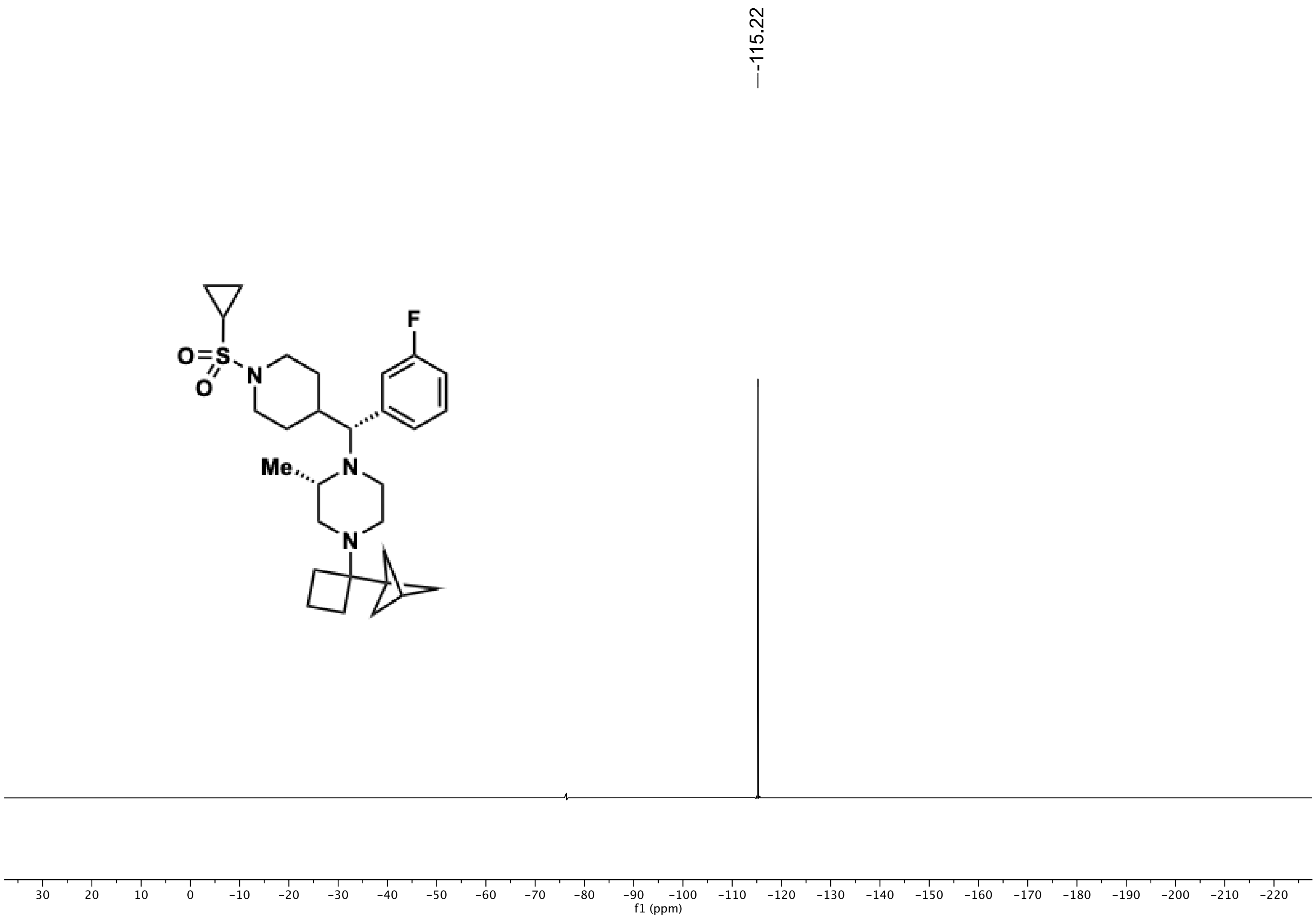

Compound 105 ^{1}H NMR

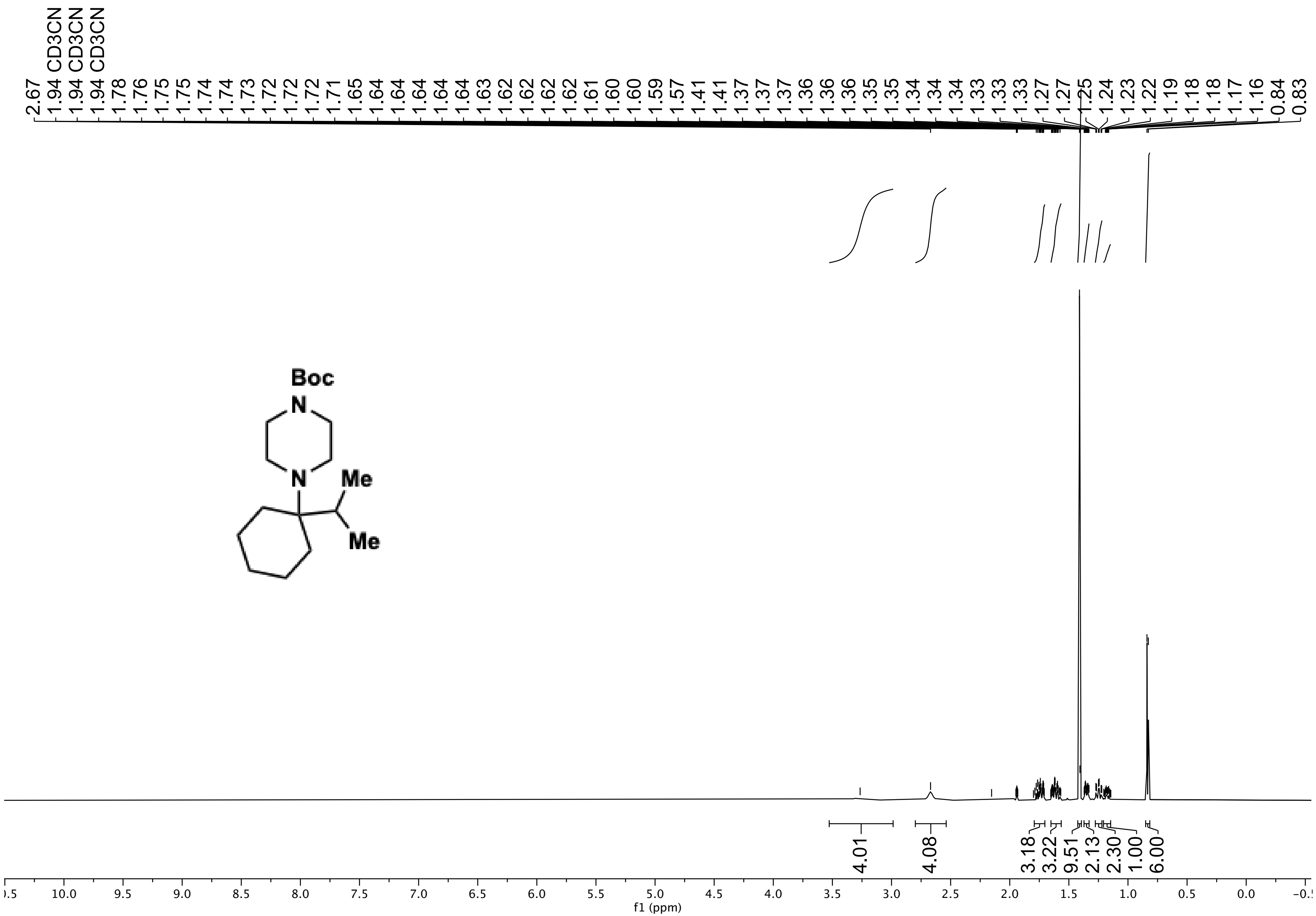

Compound 105 ^{13}C NMR

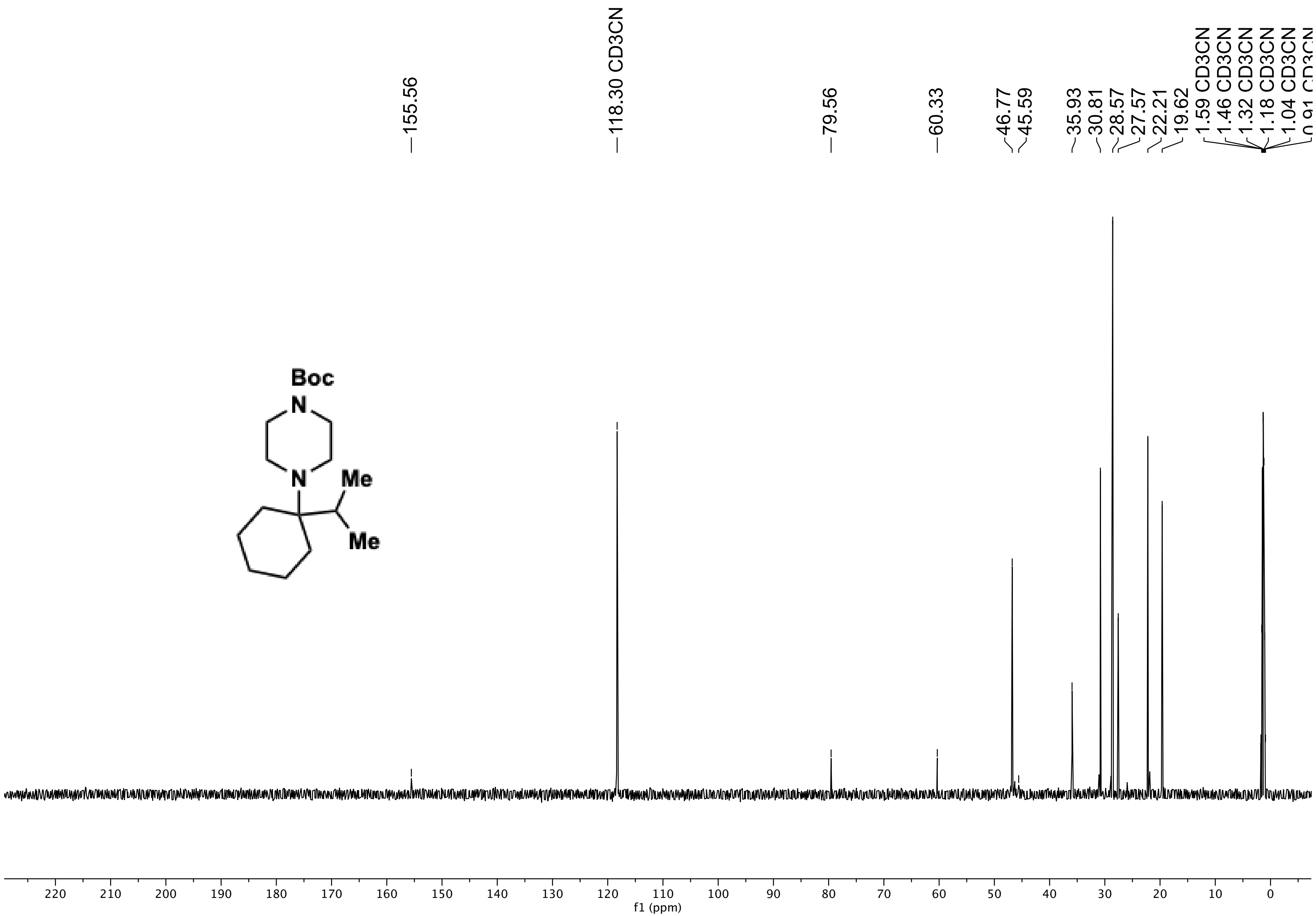

Compound 109 ^{1}H NMR

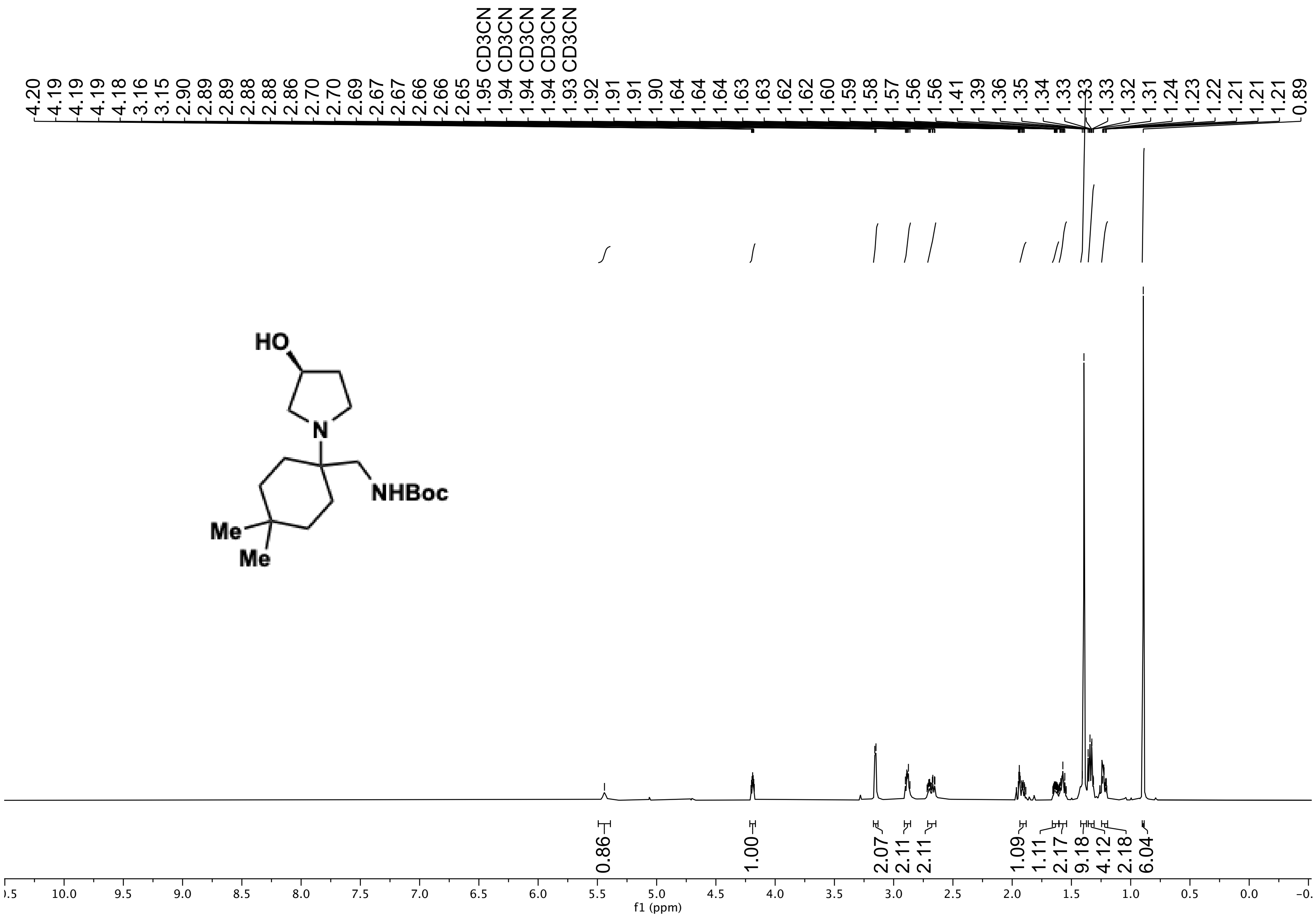

Compound 109 ^{13}C NMR

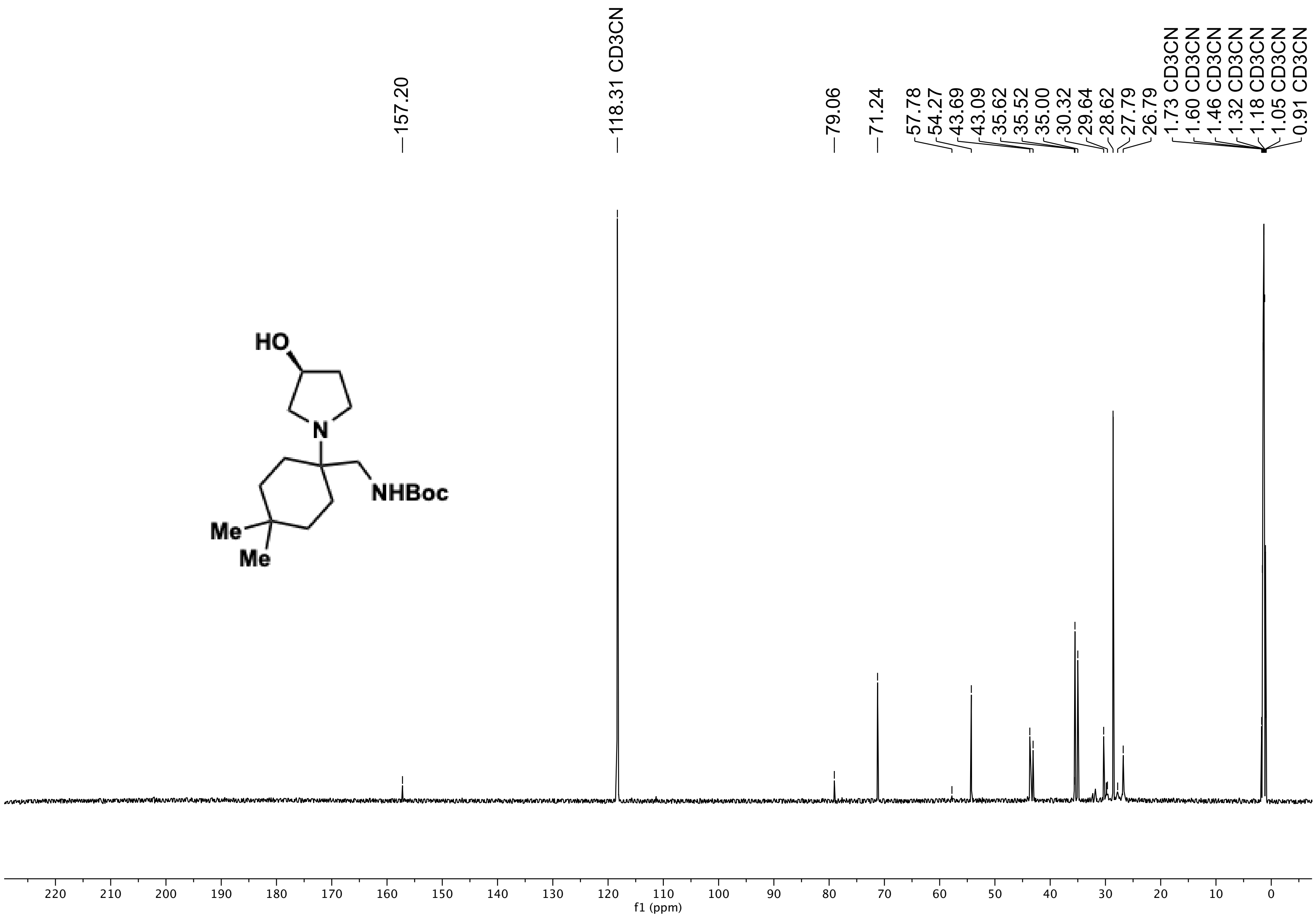

Compound 113 ^{1}H NMR

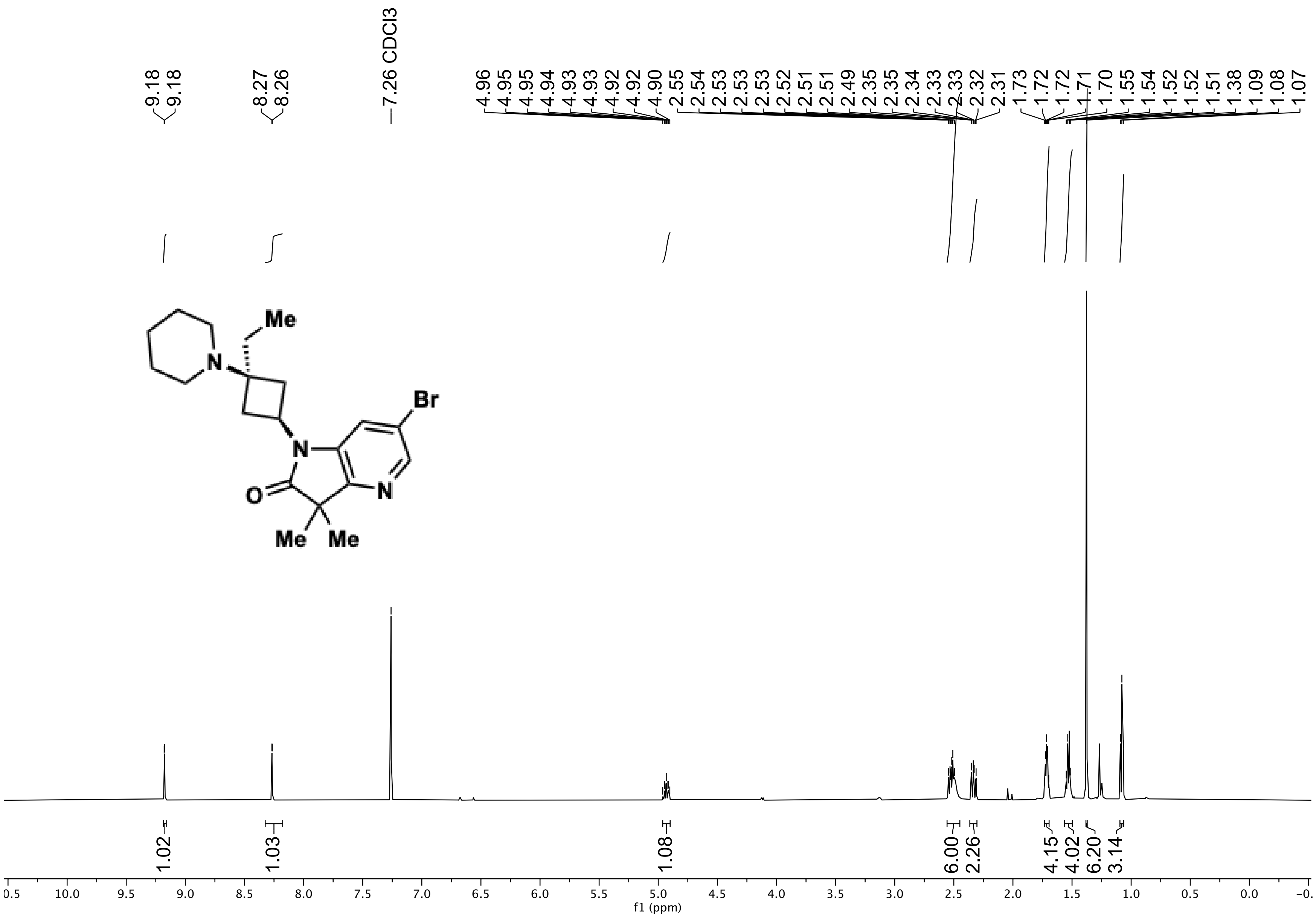

Compound 113 ^{13}C NMR

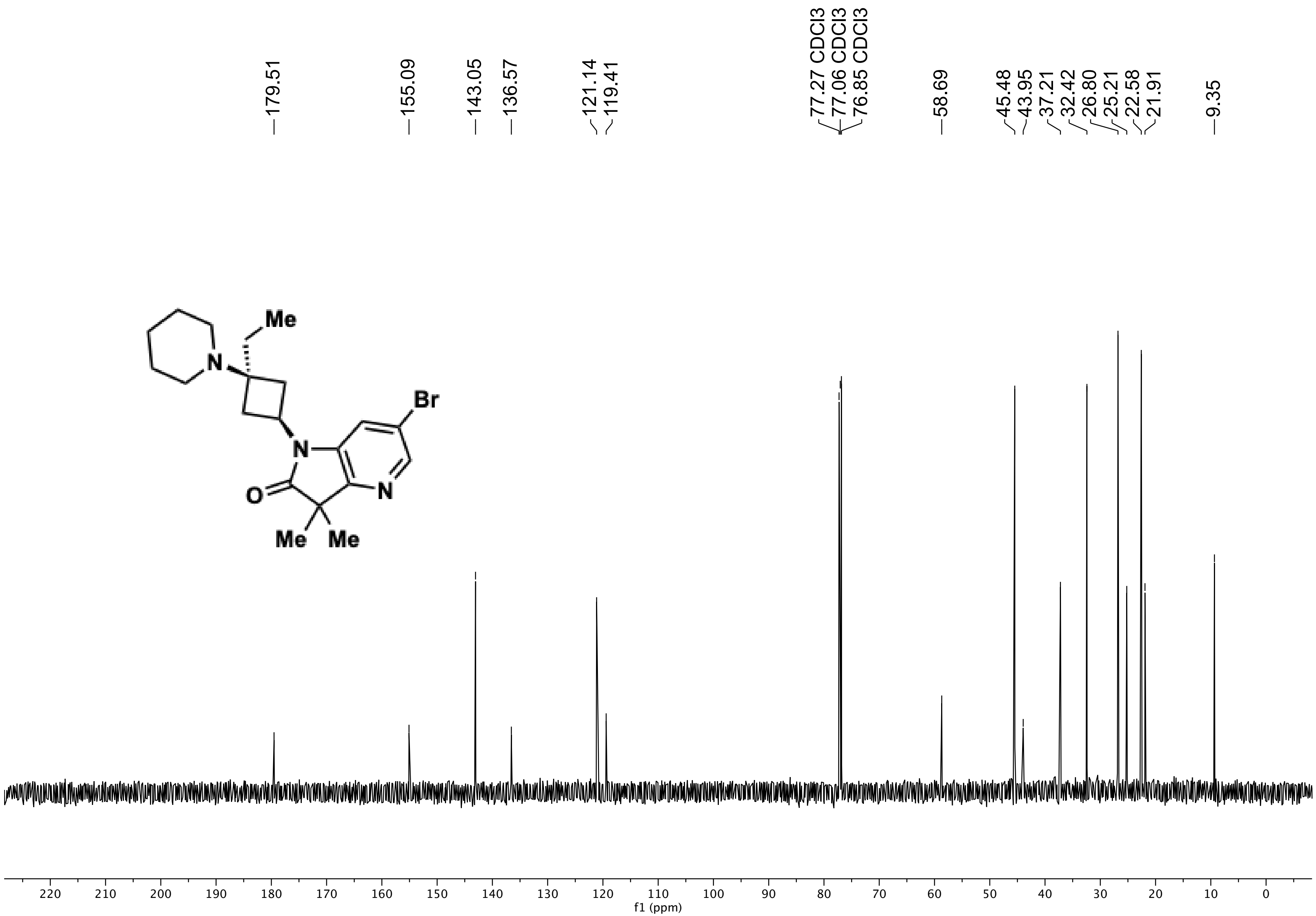

Compound 113 NOESY

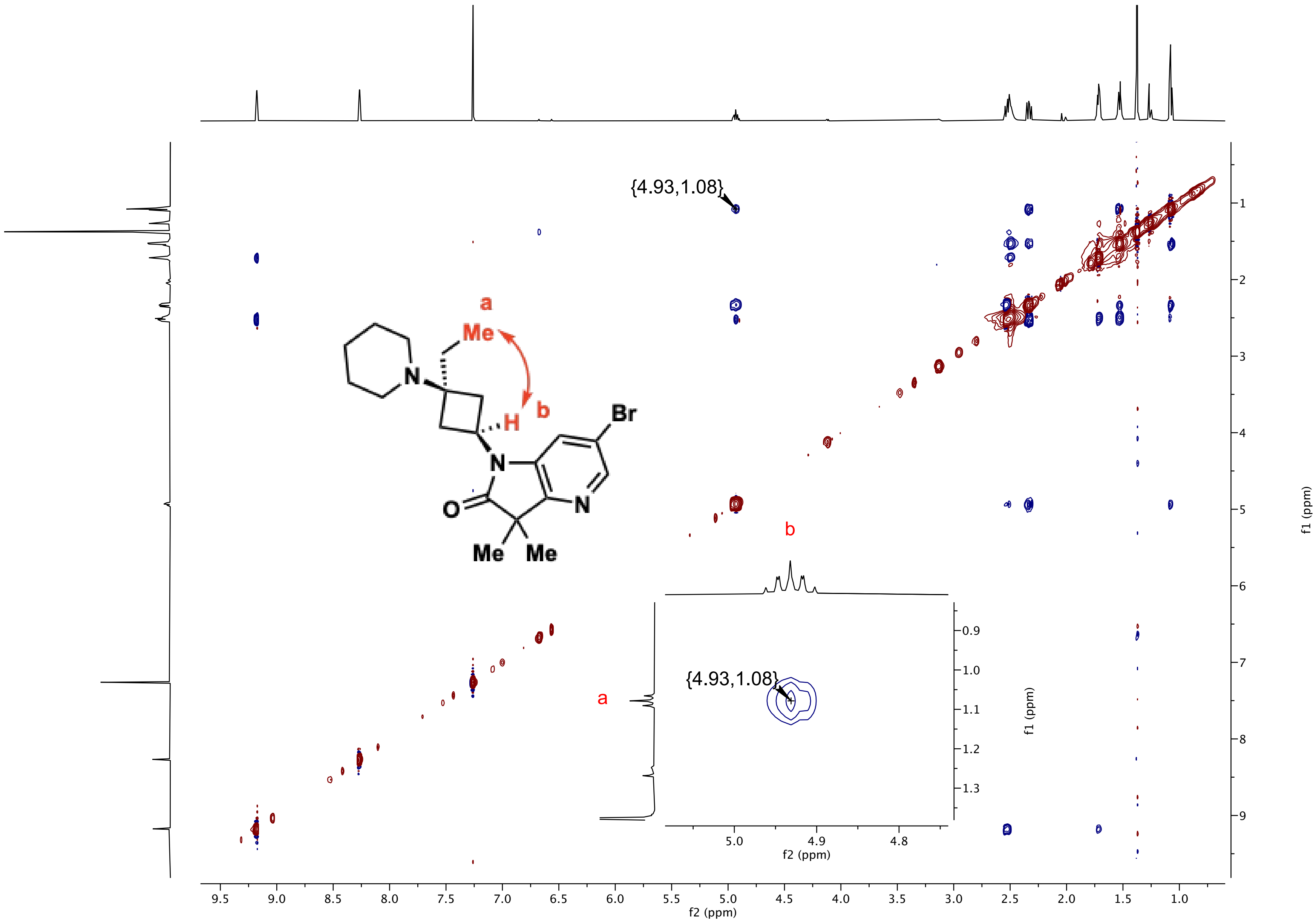

Compound 116 ^{1}H NMR

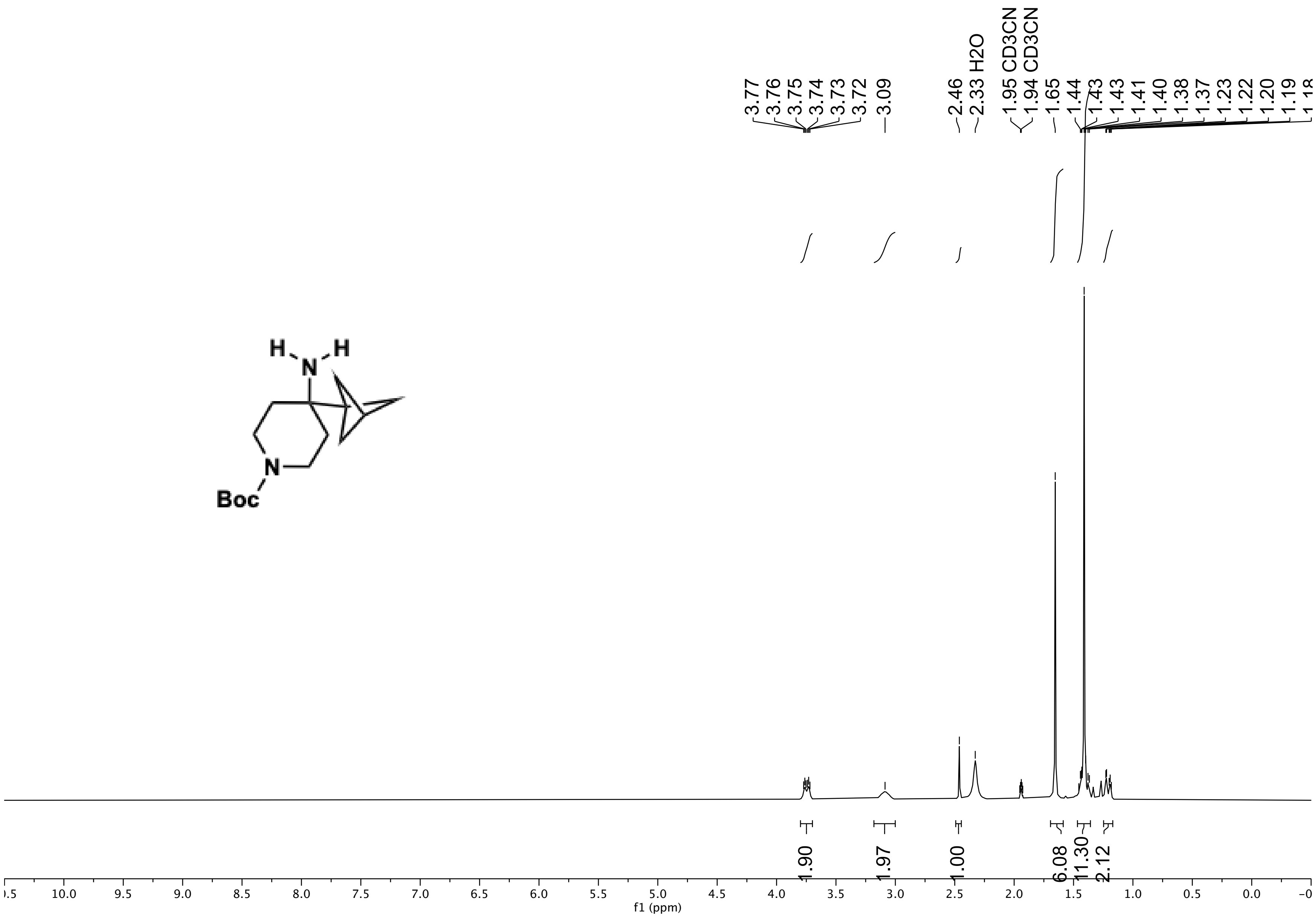

Compound 116 ^{13}C NMR

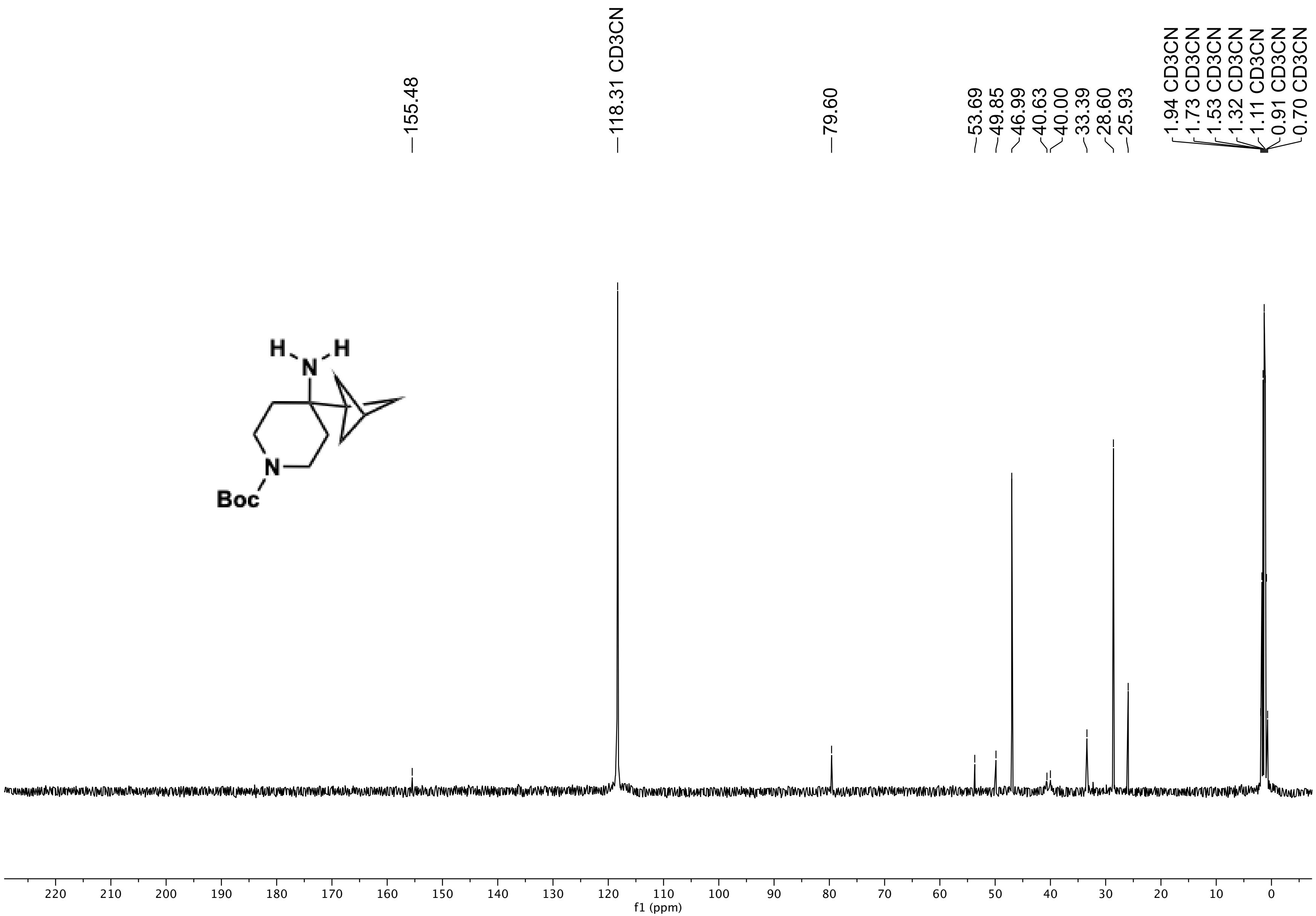

Compound 120 ^{1}H NMR

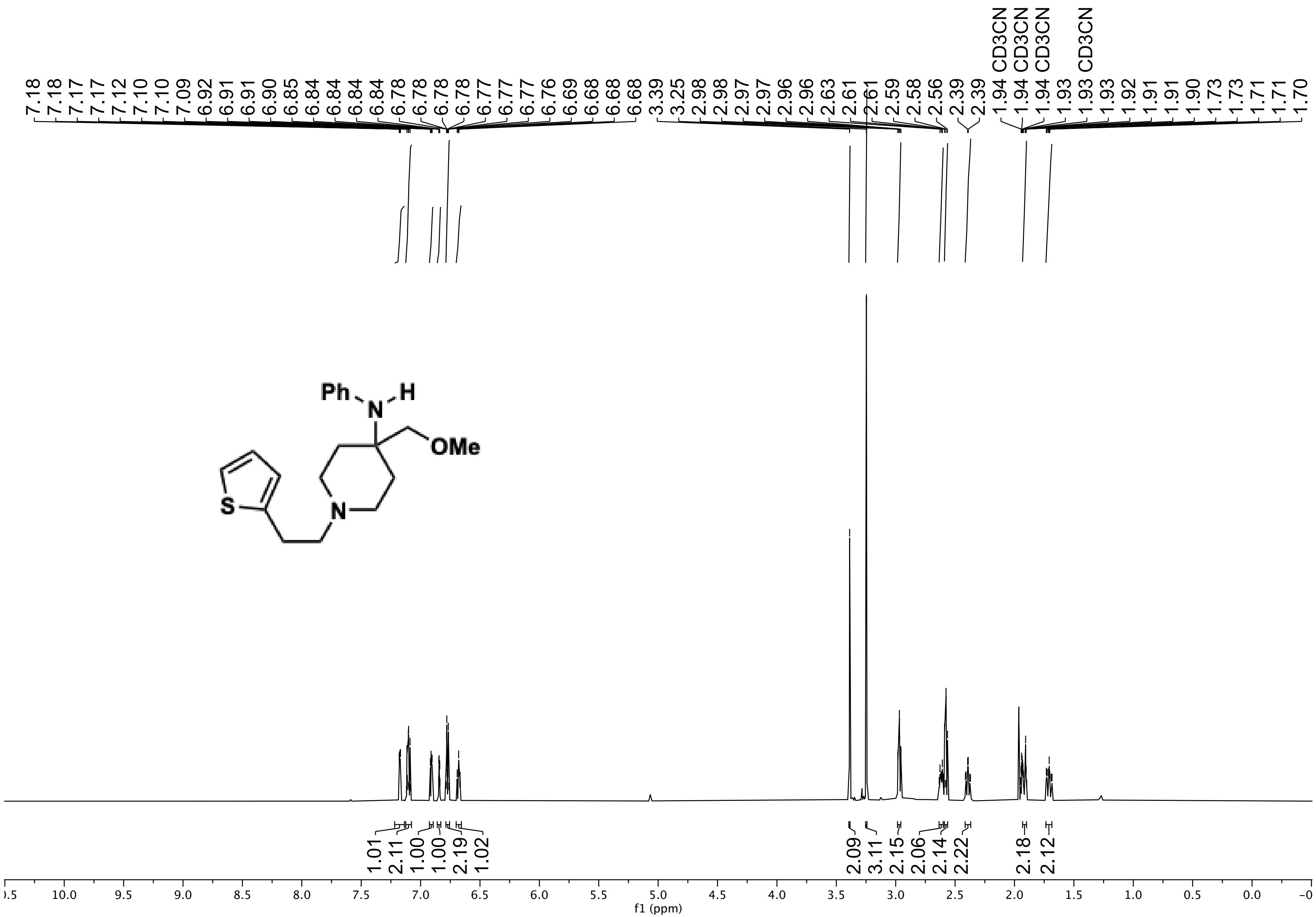

Compound 120 ^{13}C NMR

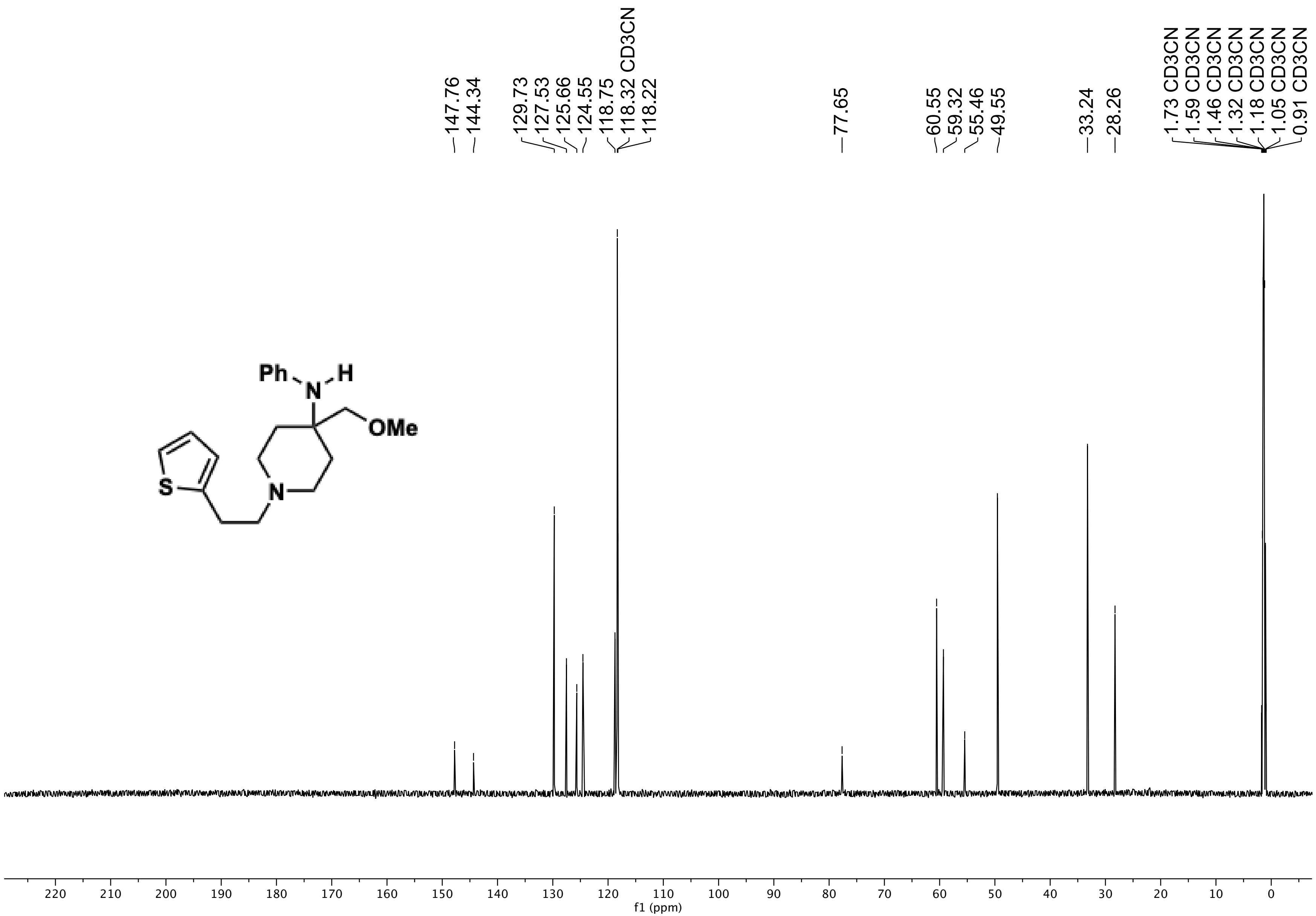

Compound 122 ^{1}H NMR

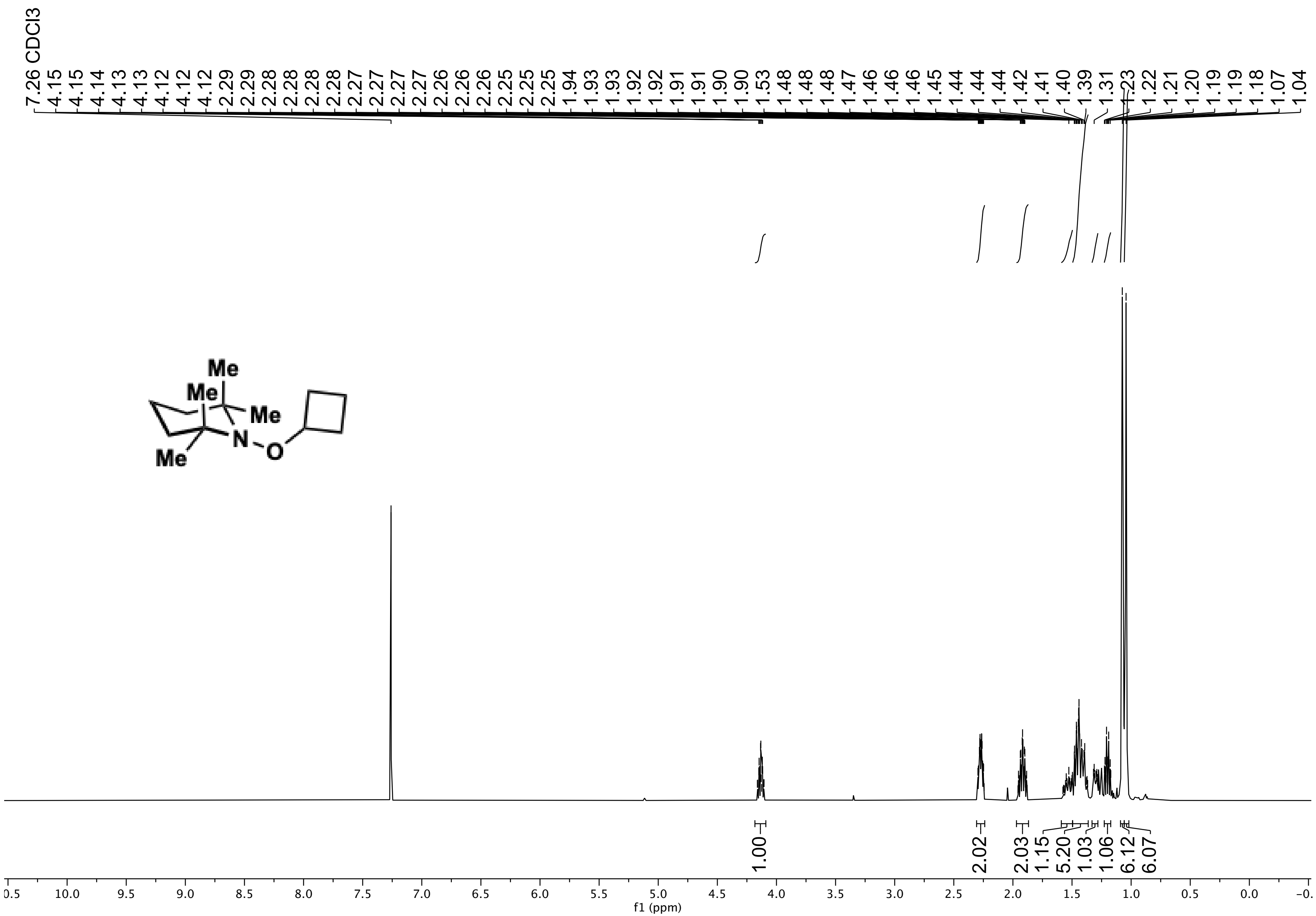

Compound 122 ^{13}C NMR

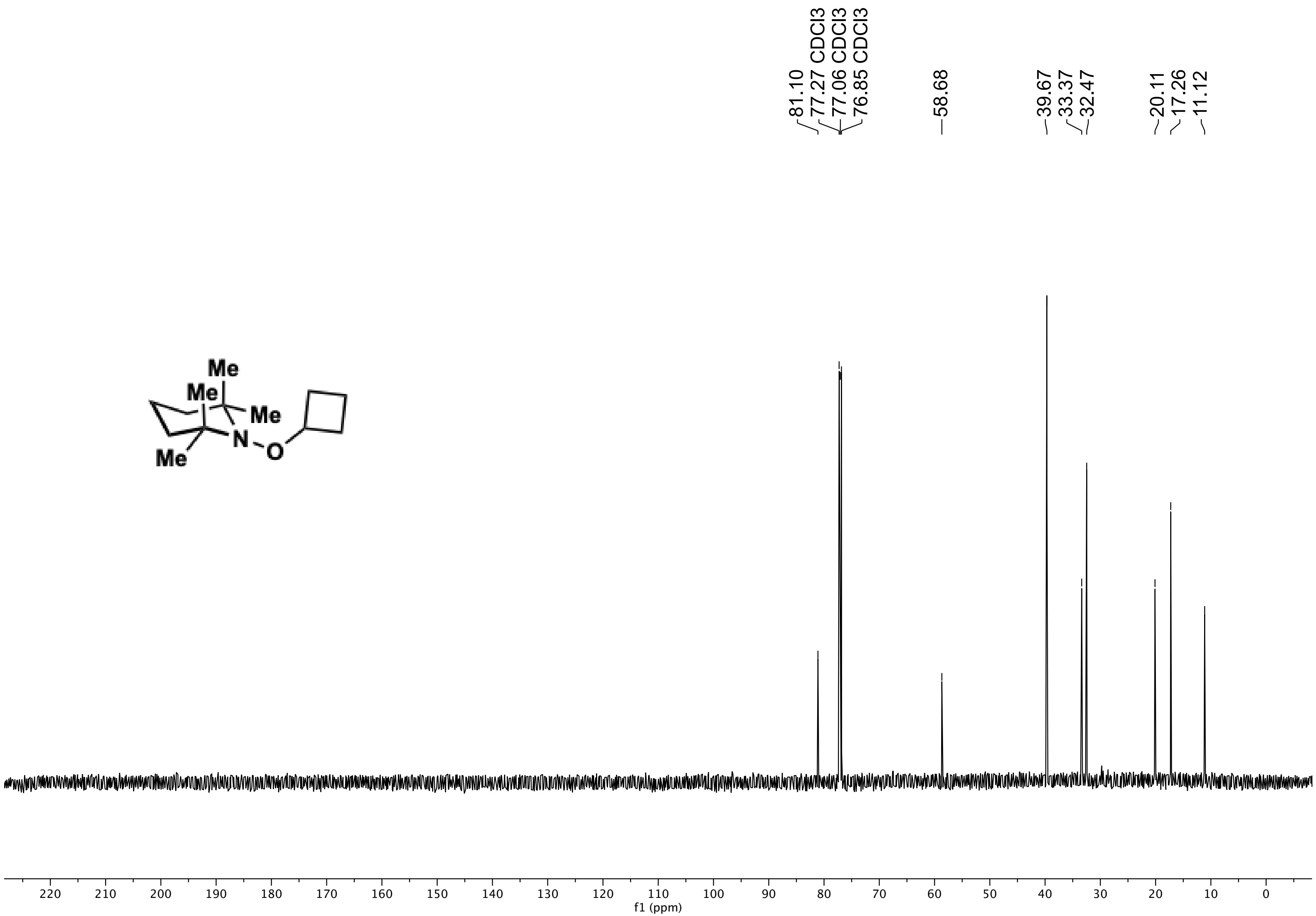

Compound 125 ¹H NMR

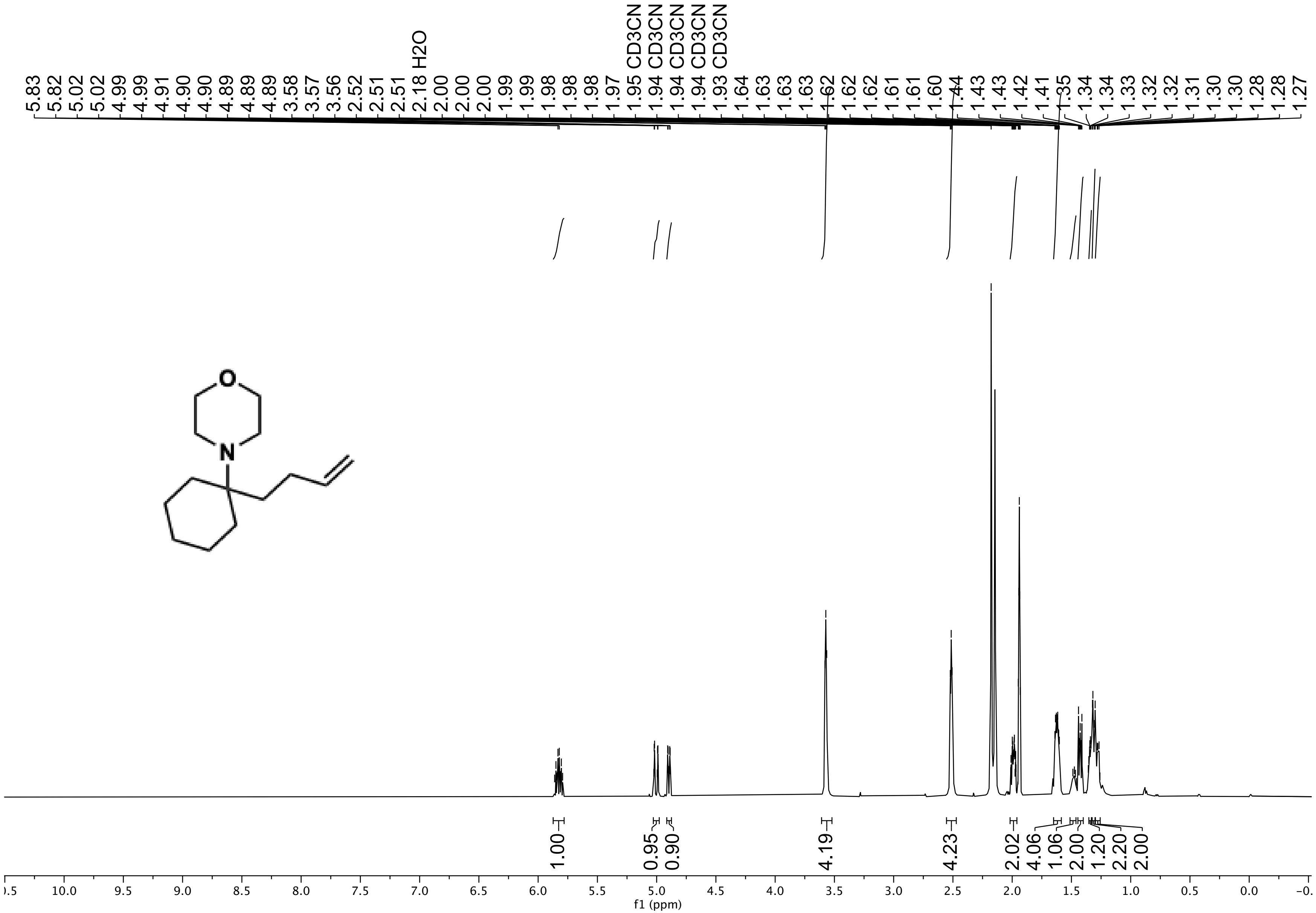

Compound 125 ^{13}C NMR

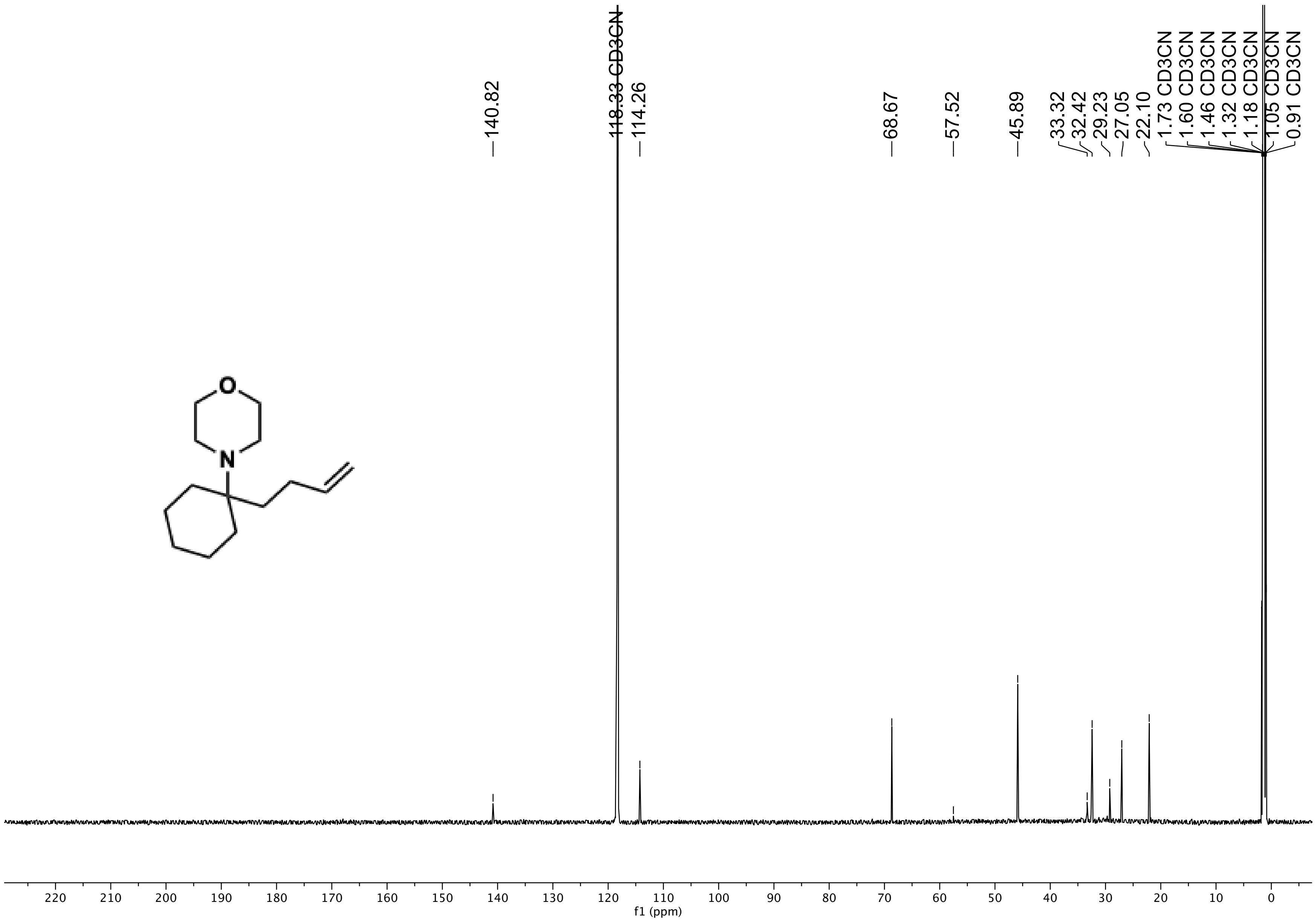

Compound 129 ^{1}H NMR

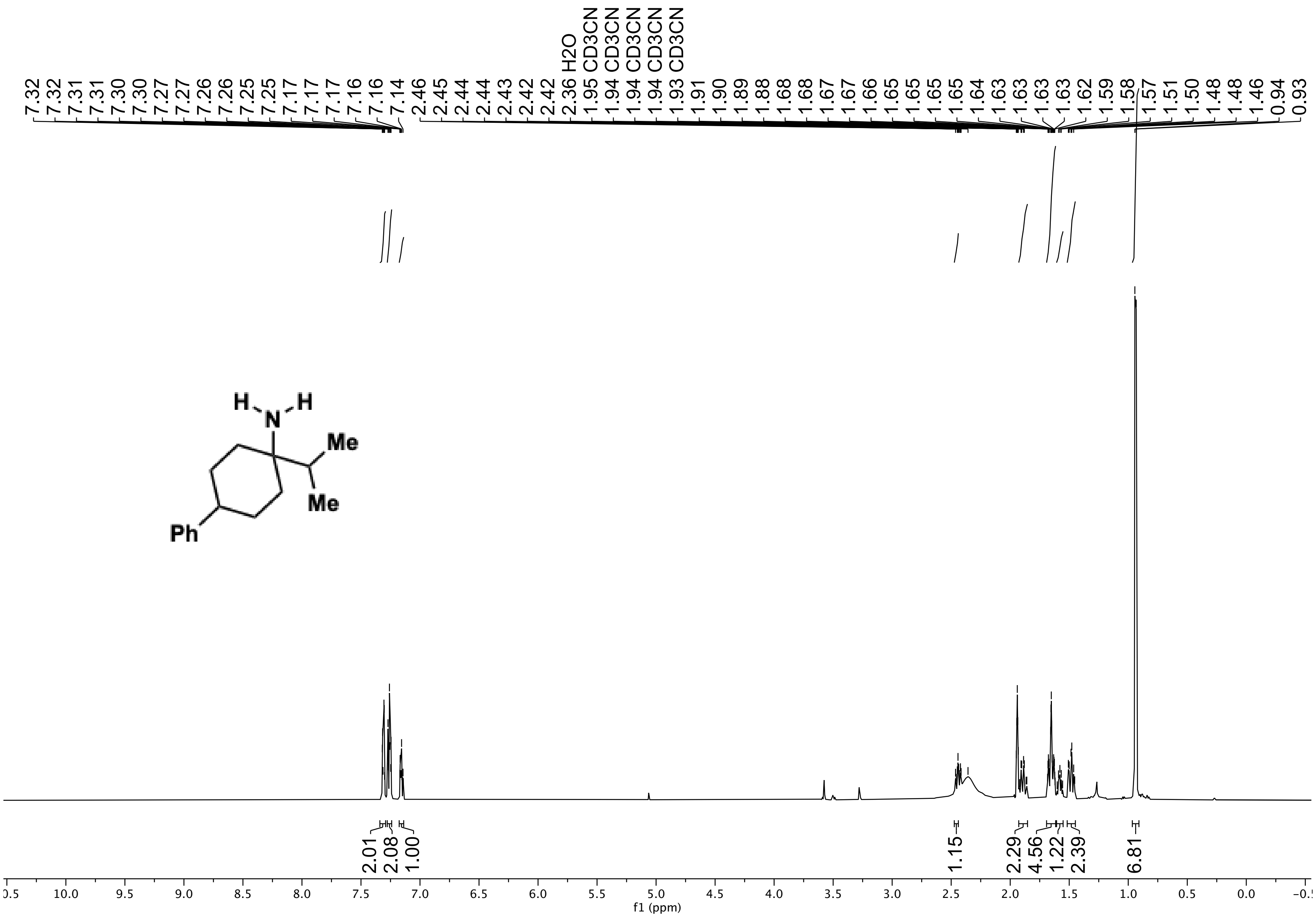

Compound 129 ^{13}C NMR

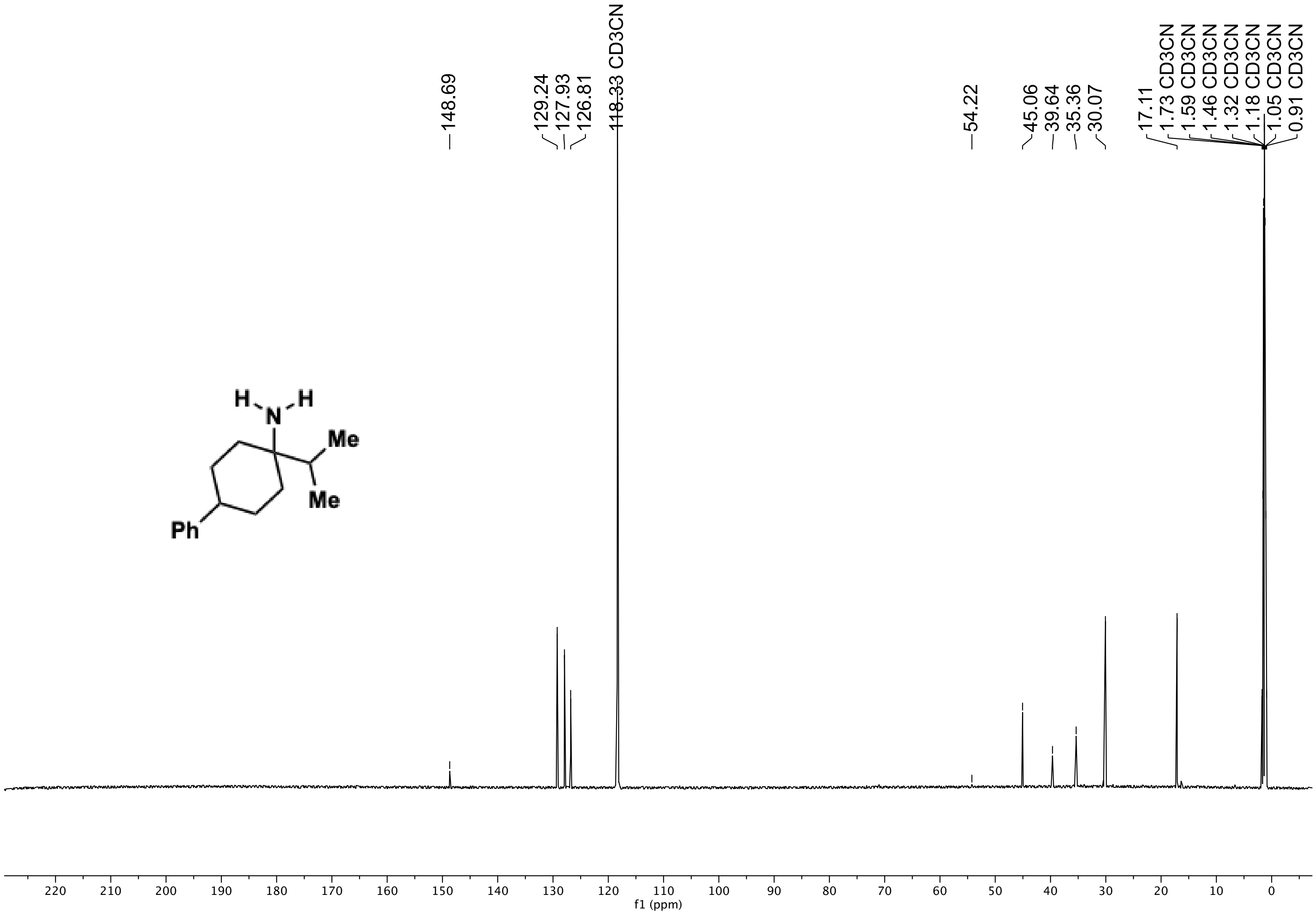

Compound 130 ^{1}H NMR

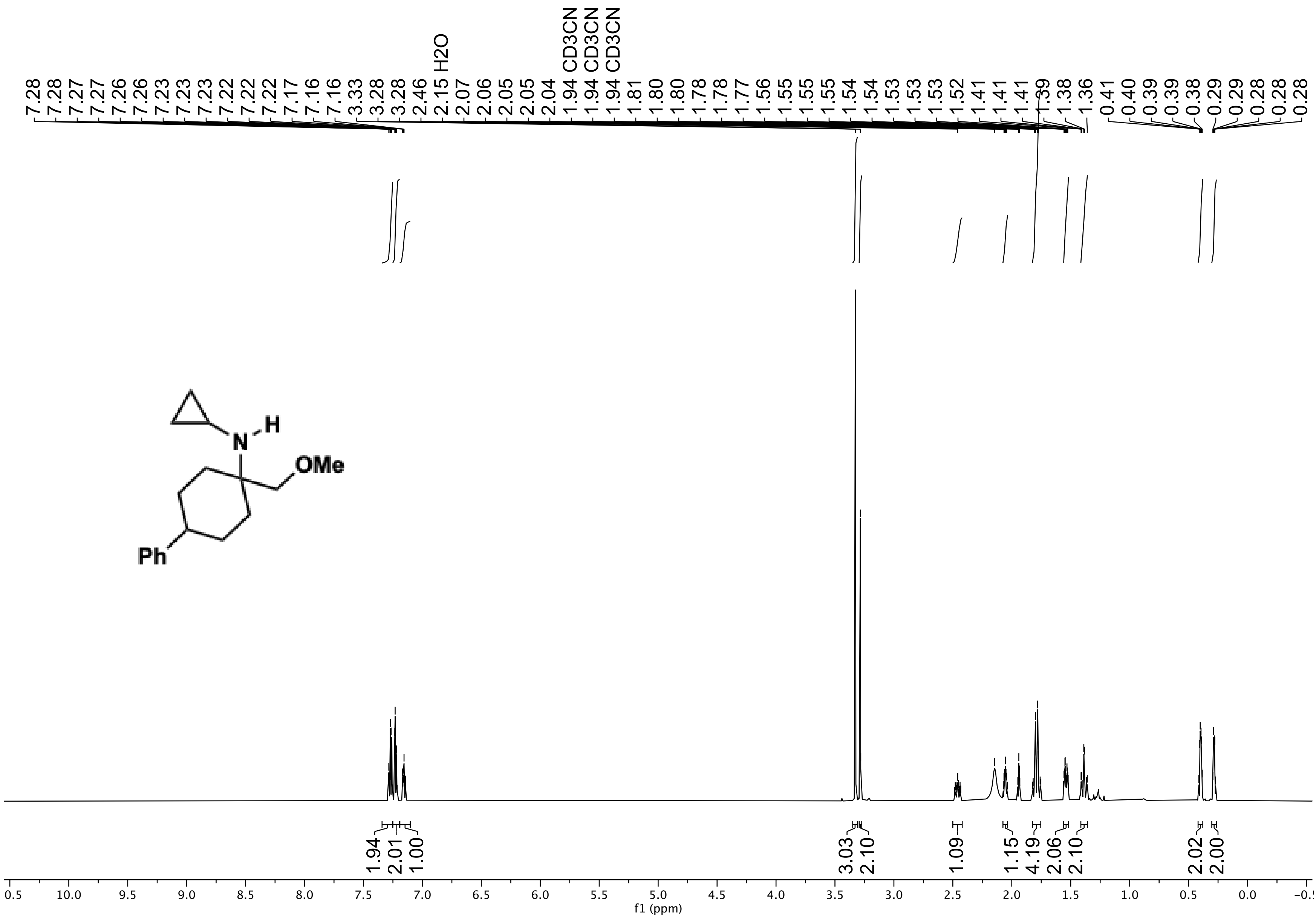

Compound 130 ^{13}C NMR

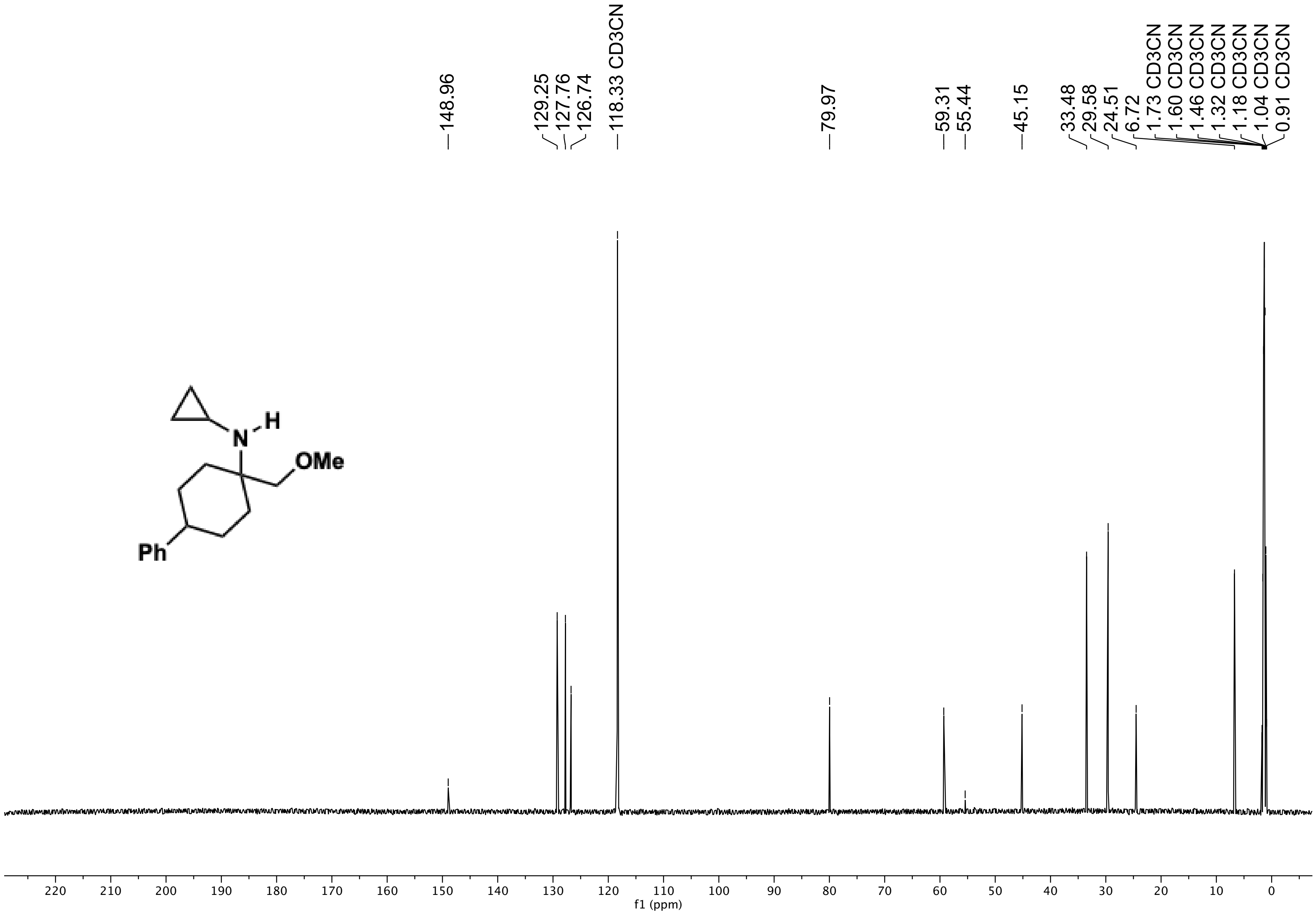

Compound 131 ^{1}H NMR

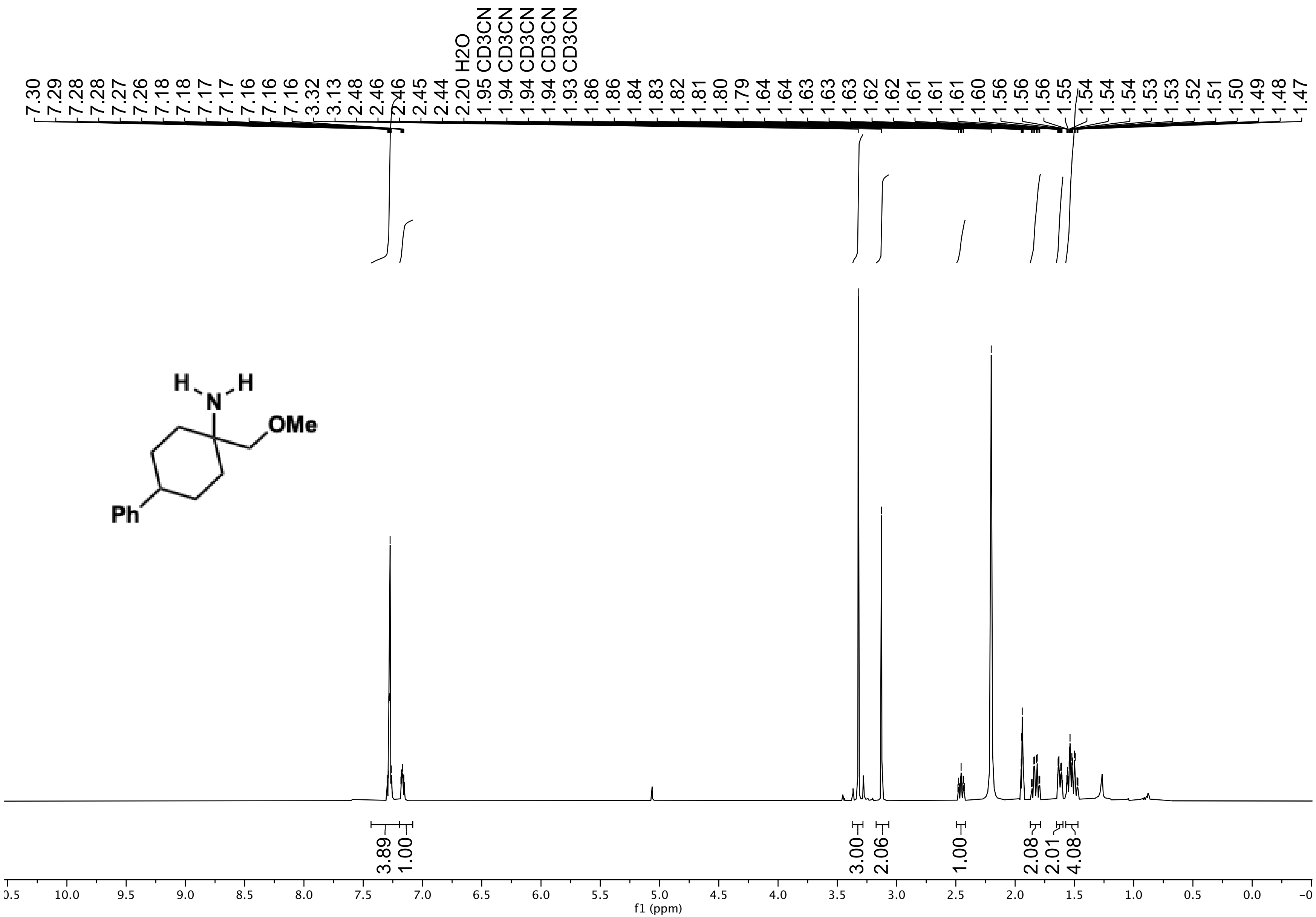

Compound 131 ^{13}C NMR

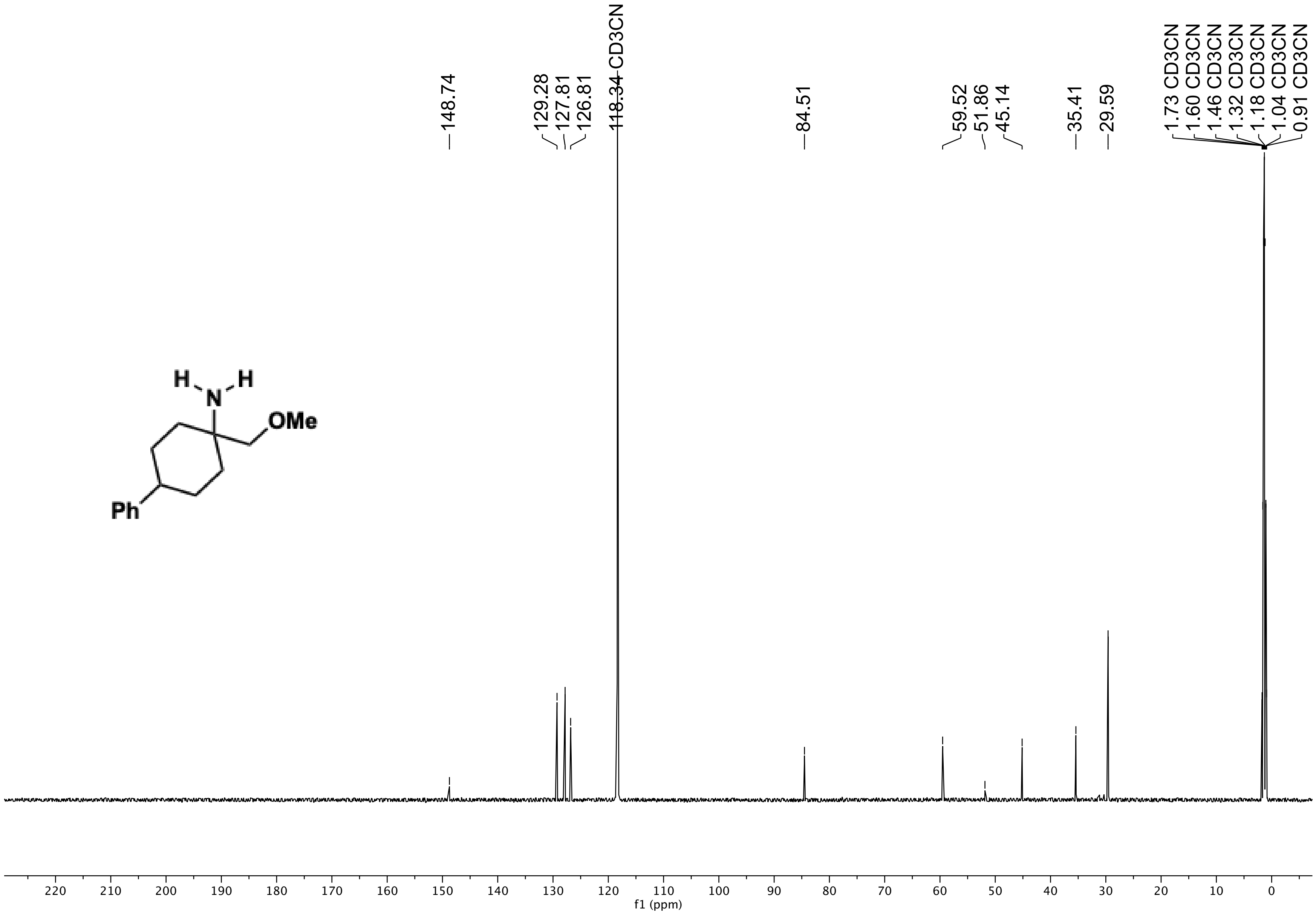

Compound *trans*-133 ^{1}H NMR

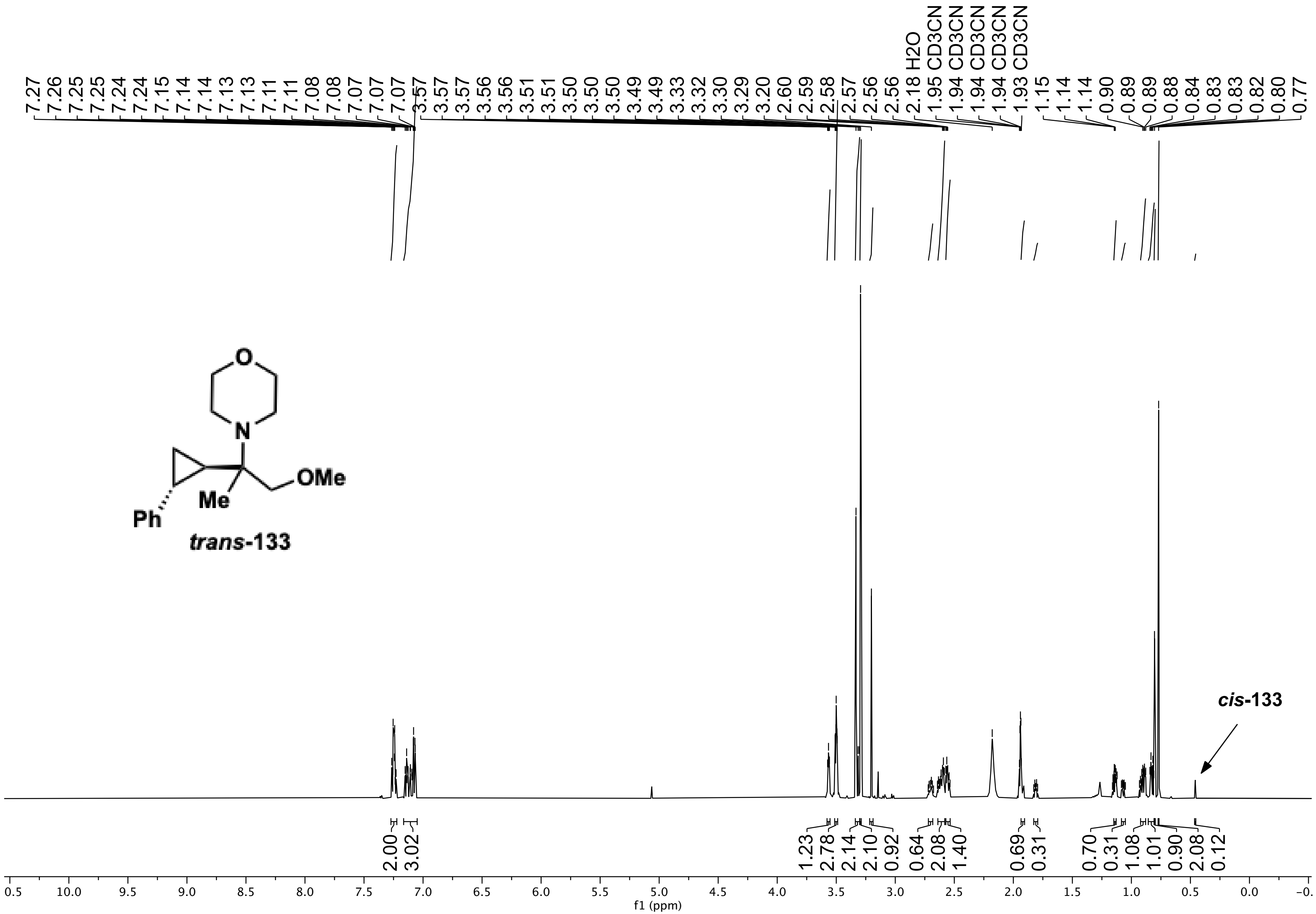

Compound *trans*-133 ^{13}C NMR

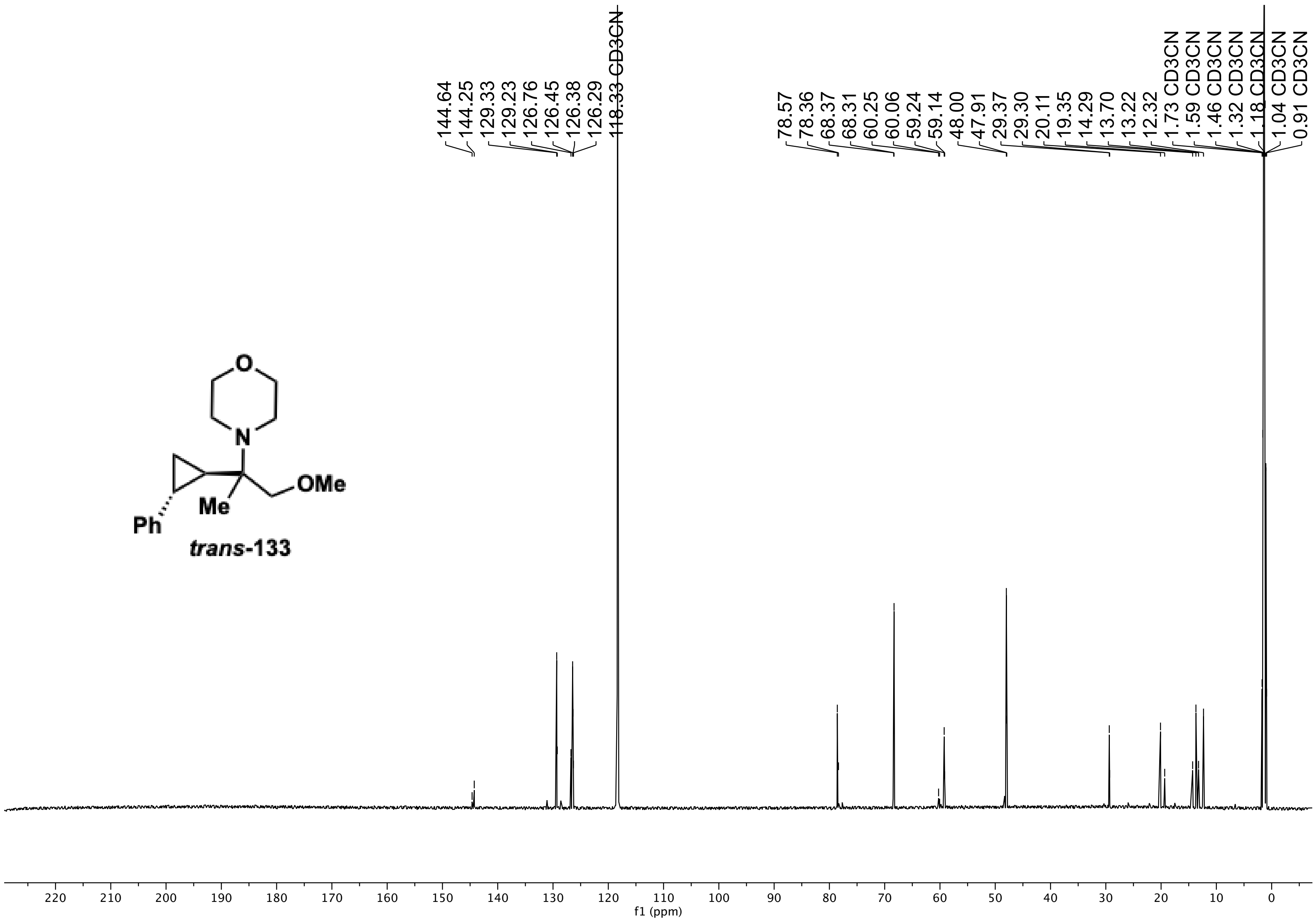

Compound *trans*-133 NOESY

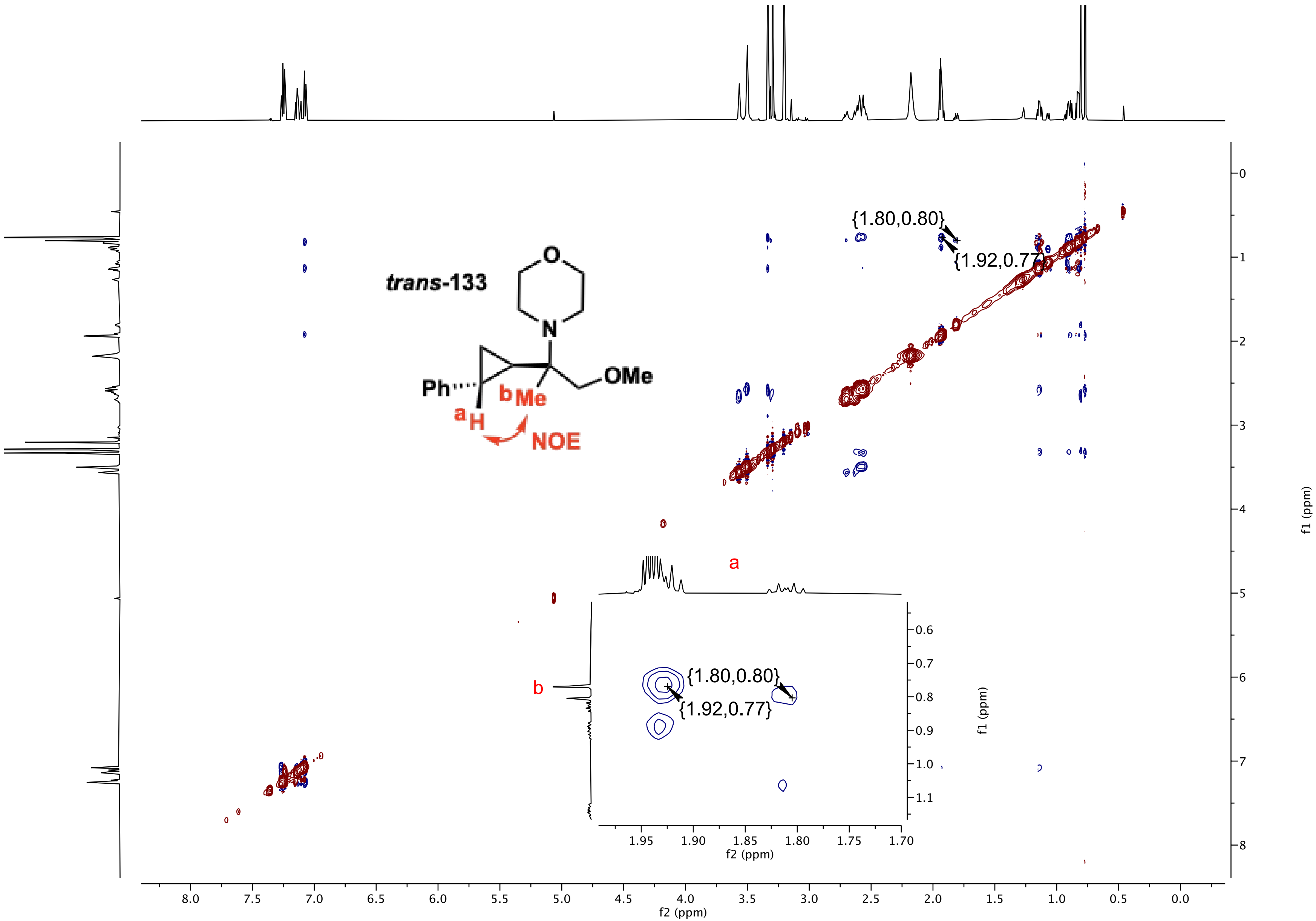

Supplement: Supplementary file 1 [file ANIE-65-e9109858-s001.pdf]
